# Reducing global inequities in medical oxygen access: the *Lancet Global Health* Commission on medical oxygen security

**DOI:** 10.1016/S2214-109X(24)00496-0

**Published:** 2025-02-17

**Authors:** Hamish R Graham, Carina King, Ahmed Ehsanur Rahman, Freddy Eric Kitutu, Leith Greenslade, Masooma Aqeel, Tim Baker, Lucio Flavio de Magalhães Brito, Harry Campbell, Karen Czischke, Mike English, Adegoke G Falade, Patricia J Garcia, Mireia Gil, Stephen M Graham, Amy Z Gray, Stephen R C Howie, Niranjan Kissoon, Ramanan Laxminarayan, Inês Li Lin, Michael S Lipnick, Dianne B Lowe, David Lowrance, Eric D McCollum, Tisungane Mvalo, Jacquie Oliwa, Stefan Swartling Peterson, Rediet Shimeles Workneh, Heather J Zar, Shams El Arifeen, Freddie Ssengooba

**Affiliations:** a Melbourne Children's Global Health, Murdoch Children's Research Institute, University of Melbourne, Melbourne, VIC, Australia; b Royal Children's Hospital, Melbourne, VIC, Australia; c Department of Paediatrics, University College Hospital Ibadan, Ibadan, Nigeria; d Department of Global Public Health, Karolinska Institutet, Stockholm, Sweden; e Maternal and Child Health Division, International Centre for Diarrhoeal Disease Research, Dhaka, Bangladesh; f Department of Pharmacy, School of Health Sciences, Makerere University, Kampala, Uganda; g International Maternal and Child Health, Department of Women's and Children's Health, Uppsala University, Uppsala, Sweden; h Every Breath Counts Coalition, New York, NY, USA; i Department of Medicine, Aga Khan University Hospital, Karachi, Pakistan; j Department of Emergency Medicine, Muhimbili University of Health and Allied Sciences, Dar es Salaam, Tanzania; k Pontifical Catholic University of São Paulo, São Paulo, Brazil; l Usher Institute, College of Medicine and Veterinary Medicine, University of Edinburgh, Edinburgh, UK; m Departamento de Neumología, Clínica Alemana de Santiago, Universidad del Desarrollo, Santiago, Chile; n Nuffield Department of Medicine, University of Oxford, Oxford, UK; o Department of Paediatrics, University of Ibadan, Ibadan, Nigeria; p School of Public Health, Cayetano Heredia University, Lima, Peru; q Azimut 360, Barcelona, Spain; r Faculty of Medical and Health Sciences, University of Auckland, Auckland, New Zealand; s College of Medicine, Nursing and Health Sciences, Fiji National University, Suva, Fiji; t Department of Pediatrics, University of British Columbia, Vancouver, BC, Canada; u One Health Trust, Washington, DC, USA; v UCL Institute for Global Health, University College London, London, UK; w Center for Health Equity in Surgery and Anesthesia, University of California, San Francisco, San Francisco, CA, USA; x International Child Health, Murdoch Children's Research Institute, Melbourne, VIC, Australia; y Pandemic Preparedness and Response, Global Fund, Geneva, Switzerland; z Global Program in Pediatric Respiratory Sciences, Department of Pediatrics, Eudowood Division of Pediatric Respiratory Sciences, Johns Hopkins School of Medicine, Baltimore, MD, USA; aa University of North Carolina Project Malawi, Lilongwe, Malawi; ab Health Services Unit, KEMRI-Wellcome Trust Research Programme, Nairobi, Kenya; ac Department of Public Health, Institute of Tropical Medicine, Antwerp, Belgium; ad Addis Continental Institute of Public Health, Addis Ababa, Ethiopia; ae Department of Pediatrics and Child Health, Red Cross Children's Hospital & South Africa-Medical Research Council Unit on Child and Adolescent Health, University of Cape Town, Cape Town, South Africa; af School of Public Health, Makerere University, Kampala, Uganda

## Executive summary

*“Oxygen delayed is life denied.”*

*—Person who survived COVID-19, Kenya*

Medical oxygen is an essential treatment that has been in clinical use for more than 150 years. It is required at every level of the health-care system for children and adults with a wide range of acute and chronic conditions and to ensure safe surgery and perioperative care, and thus must be available to everyone who needs it. The COVID-19 pandemic shone a spotlight on the long-standing inequities in access to medical oxygen globally, and the importance of this life-saving therapy to people of all ages in every part of the world. It was against this backdrop that the *Lancet Global Health* Commission on medical oxygen security was launched in 2022, with the aim of synthesising available evidence and harnessing expertise to produce concrete and actionable recommendations for governments, industry, global health agencies, donors, the health-care workforce, and researchers.

Our work emphasises that provision of oxygen is an essential public service, not just a commodity or commercial product, and that achieving equitable oxygen access will require a systems approach addressing multiple domains (production, storage, distribution, supply, clinical use, coordination, regulation, and financing) across the health, education, energy, industry, and transport sectors. Previous efforts, including major investments in response to the COVID-19 pandemic, largely focused on the delivery of equipment to produce more oxygen, but did not invest in the systems and people required to ensure equipment is distributed, maintained, and used safely and effectively. Key findings from this Commission show how future investment in strengthening oxygen systems could have a huge impact by saving millions of lives, accelerating progress towards the Sustainable Development Goals (SDGs), and preparing the world for future pandemics.

### The global need for medical oxygen is high

Each year, 374 million people need medical oxygen: 364 million in acute medical and surgical contexts, and 9 million with long-term oxygen needs due to chronic obstructive pulmonary disease. 306 million (82%) of the 374 million people who need oxygen live in low-income and middle-income countries (LMICs), 253 million (68%) in south Asia, east Asia and the Pacific, and sub-Saharan Africa. Patients with acute medical and surgical needs require a minimum of 1·2 billion normal cubic metres (Nm^3^) of medical oxygen annually. This need is rising, driven by population growth and unmet surgery and long-term oxygen therapy needs. Efforts to prevent oxygen need are critical, and include immunisation, smoking prevention and cessation, reductions in malnutrition and indoor and outdoor air pollution, and mitigation of climate change. During emergencies (eg, epidemics, natural disasters, war), the need for oxygen can increase exponentially, putting enormous pressure on health systems. In 2021, an additional 52 million patients needed 1·9 billion Nm^3^ of oxygen to treat COVID-19 globally.

### Global access to medical oxygen is highly inequitable

There are huge gaps in oxygen coverage in many LMICs despite investments since the COVID-19 pandemic. We found that more than 5 billion people—ie, more than 60% of the world's population—do not have access to safe, quality, and affordable medical oxygen services. In LMICs, only 89 million (30%) of the 299 million people who need oxygen for acute medical or surgical conditions receive adequate oxygen therapy, with the lowest access in sub-Saharan Africa. The coverage gap for medical oxygen in LMICs is thus 70%, which far exceeds those for HIV/AIDS (23%) and tuberculosis (25%) treatments. Major contributors to the oxygen coverage gap include people not reaching a health facility, facilities lacking basic oxygen service capacity, failure to identify oxygen need due to the unavailability of pulse oximetry, interrupted, unsafe, or otherwise low-quality oxygen care, and high costs for patients. In LMICs, pulse oximeters are available in only 54% of general hospitals and 83% of tertiary hospitals, and oxygen therapy is available in only 58% of general hospitals and 86% of tertiary hospitals. Frequent shortages and equipment breakdown result in the need to ration care, which is a source of moral distress for health-care workers. In primary health-care facilities in LMICs, pulse oximeters and oxygen are nearly entirely unavailable.

### The cost of filling the oxygen gap is high but oxygen is a highly cost-effective investment

We estimate that closing the large acute medical and surgical oxygen coverage gap in LMICs will require an additional US$6·8 billion annually, equating to $34·0 billion in the next 5 years. This estimate does not include the substantial costs of meeting the additional oxygen requirements caused by pandemics (for reference, the additional cost of COVID-19-related oxygen requirements in LMICs in 2021 was $6·8 billion) or costs for long-term oxygen therapy. The case for investing in medical oxygen is strong: it is as cost-effective as routine childhood immunisation, would enable governments to make progress on eight of the nine SDG 3 goals, and could reduce deaths during future pandemics.

### National medical oxygen plans are essential to facilitate investment and coordinate service delivery

Fewer than 30 countries have developed national oxygen plans as stipulated in WHO's 2023 increasing access to medical oxygen resolution. We encourage all governments to do so by 2030. To develop the plan, governments should bring together public and private sector partners with a stake in medical oxygen delivery—including the health, education, industry, energy, and transport sectors—to design the system and institute a governance structure that keeps all parties connected in system management. Oxygen systems should be integrated into broader national health plans and pandemic preparedness and response strategies.

### Pulse oximetry is crucial to safe, affordable oxygen care at all levels of health care

Pulse oximetry measures an essential vital sign—peripheral blood (haemoglobin) oxygen saturation—that should be routinely assessed in all patients at all levels of health care. However, health-care workers in LMICs are poorly equipped or supported to use pulse oximeters effectively. Furthermore, pulse oximetry and oxygen therapy are not included in many clinical guidelines and health curriculums. We found that pulse oximetry was done in only 19% of people presenting to general hospitals in LMICs, and almost never done at primary health-care facilities. Use of pulse oximetry was least common in small and rural government health facilities and across sub-Saharan Africa. We recognise an urgent need to make high-quality, robust pulse oximeters more affordable and to ensure that they are used more commonly, as well as a need to improve the accuracy of pulse oximeters in all populations, including people with darker skin and infants and young children.

### Oxygen systems should suit the context and be affordable to all patients

There is no one-size-fits-all national medical oxygen system. Rather, governments should define priorities and optimise their systems to suit local conditions. Most health systems and health facilities will benefit from a mixed-source oxygen supply (ie, liquid oxygen, oxygen plant, oxygen cylinders, and oxygen concentrator), including reliable back-ups in case of failure or to meet surges in demand during emergencies. Operational costs account for 50–80% of total system costs but have received little investment to date, with major consequences for the functioning, sustainability, and effective use of oxygen equipment. We particularly highlight the importance of investing in the clinical and engineering workforces. There are many different models for managing a national oxygen system, from fully government run to fully run by the private sector. Whatever the system, governments should ensure that costs are not shifted to patients. Patient and caregiver testimonies repeatedly describe huge out-of-pocket costs, and we strongly urge governments to include pulse oximetry and oxygen services in universal health coverage schemes and to pursue other strategies to minimise user fees.

### Close collaboration is needed between the medical oxygen industry, governments, and global health agencies

The medical oxygen industry, like the pharmaceutical industry, is an essential part of the public health and pandemic preparedness and response architecture. Governments are responsible for ensuring that medical oxygen markets function safely, competitively, and with price transparency, and for ensuring that national regulations defining medical oxygen quality and safety are aligned with the updated WHO *International Pharmacopoeia*. Companies should adopt specific oxygen access targets and publish progress. Global health agencies should regularly assess the medical oxygen industry's progress (as they assess the pharmaceutical industry's). We call on global health agencies and donors to maintain oxygen access as a global health priority, including supporting the new Global Oxygen Alliance and replenishing The Global Fund to Fight AIDS, Tuberculosis and Malaria with a strong oxygen-access mandate. Access to medical oxygen and related tools and therapies should be fully integrated into global pandemic preparedness and response architecture.

### Accurate, timely data for oxygen systems are essential

We found huge gaps in data about oxygen coverage and estimates of cost-effectiveness for different oxygen solutions and patient populations, as well as major deficiencies in the tools used to monitor oxygen systems and service delivery. We present two new tools to help governments, health facilities, and global health agencies: ten oxygen coverage indicators and a national Access to Medical Oxygen Scorecard, which governments should use to both plan their national oxygen systems and report progress. We also identify areas in which further research is needed to close the most crucial evidence gaps in medical oxygen access.

Finally, we note the robust discussions on the future of global health after the SDGs and the calls for an approach that delivers not only for human health but also for planetary health. By centring equity and sustainability through practical action, this Commission provides a path for making efforts to increase access to medical oxygen a global health exemplar. Integrating oxygen investments into national plans and health-systems strengthening efforts is likely to improve health services and benefit all patients, everywhere. Embracing oxygen systems and devices that are energy-efficient and powered by renewable energy would help to keep carbon emissions down, while investment in local maintenance and repair would reduce the financial, human, and environmental costs of device graveyards. National medical oxygen systems can be at the forefront of ensuring the long-term health and sustainability of people and the planet, but only with investment in closing the wide gaps in access to medical oxygen for all.

## Introduction

### Medical oxygen and the COVID-19 pandemic

*“There was always a need for oxygen. It was just exacerbated by COVID. In the past, we lost so many children to pneumonia and other childhood diseases that required oxygen—and we lost adults too.”*

*—Person who survived COVID-19, Kenya*

During the COVID-19 pandemic, which caused 15·9 million deaths in 2020–21,^1^ images and stories of oxygen shortages alerted the world to the crucial role of medical oxygen and to the tragic consequences that can result when basic oxygen services fail. Although the COVID-19 pandemic represented a discrete surge in oxygen need, poor access to oxygen has been a reality for most of the world for decades, resulting in preventable deaths, lifelong disabilities, and catastrophic health expenditures. The gap between the need for, and availability of, medical oxygen is greatest in low-income and middle-income countries (LMICs), and is especially pronounced in rural communities with poor access to health care. Health-care workers in these contexts have to make difficult decisions every day about who gets oxygen therapy, but often lack essentials such as pulse oximeters, oxygen delivery devices, clinical guidelines, and referral pathways to support good decision making. This unacceptable inequity in access to medical oxygen represents a catastrophic failure of governments, global health agencies, and donors, and should be addressed urgently for the sake of everyone who suffers and dies as a result of a lack of oxygen therapy—eg, sick newborns, children with pneumonia, adults with sepsis, patients requiring anaesthesia and surgery, and older people with chronic lung conditions. Stronger oxygen systems are essential to build resilient health systems that can meet everyday needs and handle future emergencies.

Medical oxygen has been an essential medicine for more than 100 years—the first reported clinical use was for treating pneumonia in 1885^2^—yet it was not until 2017 that it was added to WHO's Essential Medicines List as a treatment for hypoxaemia, in which it was described as “a life-saving essential medicine with no substitute”.^3^ In 2008, WHO had established guidelines for safe surgery and a safe surgery checklist, which highlighted the role of pulse oximetry.^4^ In 2010—the Year of the Lung—a call to action had captured growing concern that medical oxygen was being neglected, and urged international health policy makers, funders, and implementers to put medical oxygen on the global health agenda and expand access to all who need it.^5^ However, there were no major global health initiatives or investments in medical oxygen between 2010 and 2015, which undoubtedly contributed to 133 (68%) countries failing to meet the target of a two-thirds reduction in child mortality by 2015 set out by the Millennium Development Goals.^6^

Between 2015 and 2020, several initiatives related to pulse oximetry and medical oxygen took shape and were invested in.^7^, ^8^ However, these developments were inadequate to prepare health systems for the increased medical oxygen demand caused by the COVID-19 pandemic. In 2020, as hospitals struggled with medical oxygen shortages, groups working on oxygen joined forces with the Every Breath Counts coalition, which includes more than 50 global health, industry, and academic institutions focused on helping LMICs reduce deaths from pneumonia, to advocate for the inclusion of medical oxygen in the Access to COVID-19 Tools Accelerator (ACT-A) in response to the rapidly escalating COVID-19 oxygen shortages. In February, 2021, the ACT-A Oxygen Emergency Taskforce, co-chaired by Unitaid and the Wellcome Trust, was announced. By the end of 2022, this taskforce had mobilised over US$1 billion worth of medical oxygen equipment and supplies to more than 100 LMICs, with more than 60% of this support coming from The Global Fund to Fight AIDS, Tuberculosis and Malaria.^9^ The ACT-A Oxygen Emergency Taskforce was a success story in establishing medical oxygen as a global health priority with dedicated funding from major global health agencies, but had shortcomings,^10^, ^11^, ^12^ including a 12-month delay before including oxygen as a treatment in the ACT-A architecture, no representation of LMICs in governance structures, and weak engagement with LMIC governments, industry, and local stakeholders, which undermined the principle of equity that the initiative was aiming to promote. Ultimately, these shortcomings compromised the speed and impact of investments in oxygen therapy in LMICs.^13^, ^14^

There is a widespread recognition among national governments, global health agencies, and donors that major gaps remain in access to medical oxygen, and that an absence of data, accountability, and monitoring mechanisms is compromising the impact of investments during the COVID-19 pandemic. To accelerate progress, several LMIC governments, led by Uganda, proposed a resolution on medical oxygen that was unanimously adopted by all 194 member states at the 2023 World Health Assembly.^15^ At the same time, the Global Oxygen Alliance (GO_2_AL) was announced to continue the work of the ACT-A Oxygen Emergency Taskforce, with Unitaid and The Global Fund as co-chairs.^16^ In view of the criticisms of ACT-A, the Africa Centres for Disease Control and Prevention and the Pan American Health Organization were appointed vice-chairs of GO_2_AL, and membership was intentionally broad. With this leadership structure set for only 2 years, it will be important to reflect on whether GO_2_AL overcomes the shortfalls of its predecessor. With only 5 years left to reach the 2030 Sustainable Development Goal (SDG) targets—in which oxygen systems play a crucial role in eight of the nine health goals—and with a high risk of another respiratory pandemic, access to medical oxygen needs to be firmly embedded in the global health architecture.

### About the Commission

*“I hope that the Commission's work shines a light onto the urgent need for all health systems to provide medical oxygen, as no patient who needs it can survive more than 5 or 6 mins without it.”*

*—Doctor, Ethiopia*

The *Lancet Global Health* Commission on medical oxygen security emerged from the urgent need to understand the root causes of medical oxygen shortages and to provide concrete solutions to strengthen medical oxygen systems. Our goal was to prevent a disaster such as the COVID-19 shortages from occurring again and to accelerate achievement of the SDGs by improving equitable access to medical oxygen during routine care.^17^ In view of global inequities in access, we have focused on LMICs, and particularly on securing high-quality oxygen services for general clinical care in LMICs. Within this broad mandate, the scope of this Commission was targeted to maximise its impact and relevance to policy makers and health leaders at national and subnational levels, with particular emphasis on poor and marginalised populations.

The Commission was driven by 18 commissioners—multidisciplinary academics with clinical, economic, engineering, epidemiological, and public policy expertise—representing all regions of the world. An executive committee coordinated and led the work. Commissioners were supported by a group of 40 advisers from the UN, global health agencies, donors, academic institutions, and non-governmental organisations. A large global network, the Oxygen Access Collaborators, provided constant input to the Commission and included representatives from all sectors, including industry and ministries of health. In addition, special consultations were held with patients, caregivers, and clinicians to ensure that the voices and experiences of these groups shaped the Commission's recommendations. These experiences are included as quotations throughout this report.

## Framing of medical oxygen within health systems

### Defining medical oxygen as a system

*“Oxygen is an essential medicine. There is no substitute.”*

*—Biomedical Engineer, Rwanda*

In this Commission, we conceptualise medical oxygen as a system, as well as an essential medicine and essential clinical service for patients (panel 1). We also emphasise the importance of the integration of medical oxygen across services, rather than treating it as a standalone system or simply a commodity. In this section, we outline the key definitions and conceptualisations that we use throughout the Commission. The frameworks that have underlined this work are in appendix 1 (p 4).

Panel 1 **Glossary of key terms**

**Oxygen access or quality-adjusted oxygen coverage**

The proportion of people needing medical oxygen therapy who receive treatment safely and effectively. We adopt the effective coverage cascade to illustrate the barriers to access to medical oxygen (appendix 1 p 4).

**Medical oxygen**

Purified oxygen gas used in medical therapies and described by WHO as an essential medicine without substitute.^18^, ^19^

Although different methods of production result in different levels of oxygen purity, the *International Pharmacopoeia* accepts oxygen 93 (produced by pressure-swing or vacuum-swing adsorption) and oxygen 99.5 (produced by cryogenic distillation)—the two main types of medical oxygen—as equivalent.^20^

**Oxygen therapy**

Any application of medical oxygen for patients. Simple low-flow (ie, supplemental) oxygen is typically administered via nasal prongs or face masks, which provide a total oxygen flow rate that is considerably less than the total inspiratory flow rate, meaning that room air is breathed alongside supplemental oxygen, often without humidification. Advanced forms of respiratory support, such as high-flow nasal cannulas, continuous or bilevel positive airway pressure, and mechanical ventilation (via endotracheal tube), all require specialised delivery devices, humidification (preferably warm), and the ability to mix and humidify oxygen and air.

**Oxygen services**

The package of oxygen-related care provided by health-care facilities and providers. Depending on the context, services could include pulse oximetry, simple oxygen therapy, advanced oxygen therapies, and supportive service components required for safe and effective care (eg, monitoring, medication for apnoea, suctioning, etc).

**Medical oxygen system**

The multifaceted set of components required for health-care facilities and providers to deliver oxygen services to patients, including clinical and engineering health workforces, normative guidelines, education, equipment, devices, tools, electricity, fuel, markets, supply chains, effective procurement, sustainable financing, risk-pooling, service delivery across levels and different types of health care, policy, regulation, intersectoral governance, public awareness, and advocacy.

**Pulse oximeter**

A device that non-invasively measures peripheral blood (haemoglobin) oxygen saturation by measuring the passage of different wavelengths of light through tissues. Pulse oximeters vary in style, function, cost, and quality, from portable fingertip and handheld devices to multifunctional table-top monitors.

**Hypoxaemia**

Low level of oxygen in the blood. Blood oxygenation is usually assessed as the proportion of haemoglobin that is saturated by oxygen (functional oxygen saturation). Oxygen saturation readings can be obtained by pulse oximetry (which measures peripheral oxygen saturation) or blood gas analysis (which measures arterial oxygen saturation). Blood gas analysis can also measure the amount of dissolved oxygen in the blood (ie, the partial pressure of arterial oxygen [PaO_2_]). Hypoxaemia is poorly detected with clinical signs alone.^21^ Although there is no universal cutoff used to diagnose hypoxaemia,^22^ we broadly recognise an arterial blood oxygen saturation of at least 95% (PaO_2_ 75–100 mm Hg) at sea level as normal, arterial blood oxygen saturation of 90–94% (PaO_2_ 60–<75 mm Hg) as moderate hypoxaemia, and arterial blood oxygen saturation of less than 90% (PaO_2_ <60 mm Hg) as severe hypoxaemia.^23^, ^24^ Lower saturations are normal at higher altitude.^25^, ^26^

**Occult hypoxaemia**

When a pulse oximeter reading of functional oxygen saturation does not identify true hypoxaemia measured by blood gas co-oximetry.

**Hypoxia**

Inadequate tissue oxygenation, which frequently results in cell damage and organ injury and potentially leads to organ failure and death. Hypoxia can be caused by decreased blood oxygenation (hypoxaemic hypoxia), decreased arterial oxygen content (anaemic hypoxia), inadequate blood flow (ischaemic hypoxia), and abnormal cellular oxygen utilisation (cytotoxic hypoxia).

**Oxygen need**

The number of people needing oxygen therapy in a particular period. The oxygen need includes people who are not seeking or reaching care. This definition does not include the broader population who should undergo pulse oximetry as part of routine clinical assessment but do not require oxygen therapy.

**Minimum volume of oxygen required to meet need**

The amount of oxygen required to meet patient need, calculated based on recommended flow rates and duration for various conditions and assuming no inefficiencies or wastage in oxygen use or upstream production, supply, and distribution.

**Oxygen demand**

The oxygen services that people desire or are willing to pay for. Demand is related to perceived need and individual and societal preferences, ability to pay, and accessibility of services.

**Oxygen coverage gap**

The gap between the number of people needing oxygen therapy (oxygen need) and the number of people receiving high-quality oxygen therapy (quality-adjusted coverage).

As an essential medicine, oxygen is required for the stabilisation and treatment of severely ill patients of all ages with a wide range of conditions. This includes people with primary respiratory illnesses (eg, neonatal respiratory distress, respiratory tract infections, asthma, chronic obstructive pulmonary disease [COPD], and lung cancer), people with other systemic illnesses (eg, sepsis and other severe infections, heart disease, stroke, severe anaemia, sickle cell crises, obstetric complications, trauma, and poisoning), and people undergoing surgery or anaesthetic procedures.^5^ We affirm the use of pulse oximetry to measure oxygen concentration in peripheral blood (SpO_2_), an essential vital sign for identification and monitoring of all severely ill patients with particular importance for guiding oxygen therapy. This oxygen therapy can take various forms, from the administration of low-flow oxygen therapy via mask or nasal prongs to advanced forms of respiratory support including high-flow oxygen, non-invasive ventilation, and mechanical ventilation.

As an essential service, oxygen is required at every health facility that provides care for unwell newborns, children, or adults (including perinatal care, surgical care, and ambulatory care for chronic lung conditions) and by medical transport providers.^15^ Oxygen services range from basic pulse oximetry screening of blood oxygen levels in outpatient and triage facilities, to low-flow oxygen services in inpatient wards in general hospitals, to advanced respiratory support services in intensive care settings. Services need be integrated with other clinical services for the range of conditions in which medical oxygen is used. To address differences in oxygen service need and service access and delivery challenges across the health system, we used WHO and UNICEF's three-level classification of health facilities throughout the Commission (appendix 1 p 6).^27^, ^28^

Oxygen is not simply a set of products or devices that requires procurement and supply. Rather, oxygen is a complex, multifaceted system that requires thoughtful design and multi-sectoral coordination (eg, across health, education, energy, industry, and transport) to achieve and sustain universal coverage. Given the unique status of oxygen as a medicine that can be produced and stored by several different technologies, the production-to-treatment pathway is complex and interdependent, and requires a systems perspective.

### Clinical framing: medical oxygen as life-saving, life-sustaining, and life-enhancing

*“I carry the oxygen like a backpack and so I can go to school and get together with my friends, even exercise. [With oxygen] I can live a normal life with my illness.”*

*—Child living with a chronic lung disease, Chile*

Oxygen therapy is life-saving for people with acute illness, life-sustaining for people undergoing anaesthesia and surgical care, and life-enhancing for people with chronic respiratory failure. The common indication for oxygen therapy for all these populations is hypoxaemia, with the fundamental goal of restoring or maintaining adequate blood and tissue oxygenation.^23^, ^24^ Although arterial blood gas analysis is the gold standard method for assessing oxygen concentrations in blood (either arterial oxygen saturation [SaO_2_] or partial pressure of arterial oxygen [PaO_2_]), this method is invasive, painful, time-consuming, and requires special equipment and handling, and is primarily used in critical and tertiary respiratory care contexts.^23^ As a result, pulse oximetry, which is non-invasive, low cost, and simple to use, and provides rapid and continuous results, is more commonly used to assess hypoxaemia. Pulse oximetry is a cornerstone of safe and effective oxygen service provision, pulse oximeters are an essential medical device, and SpO_2_ is a core vital sign.^29^

Normal oxygen concentrations in the blood vary by altitude and, to a lesser extent, age. There are no universal cutoffs or definitions,^22^ but we broadly describe an SaO_2_ or SpO_2_ of at least 95% (PaO_2_ of 75–100 mm Hg) at sea level as normal.^25^, ^26^, ^30^ We use moderate hypoxaemia to refer to an SaO_2_ or SpO_2_ of 90–94% (PaO_2_ of 60–<75 mm Hg), and severe hypoxaemia to describe an SaO_2_ or SpO_2_ of less than 90% (a PaO_2_ <60 mm Hg).^23^, ^24^ In most clinical guidelines, an SpO_2_ of less than 90% is considered to suggest hypoxaemia severe enough to require admission to hospital for supplemental oxygen therapy or other respiratory support, or both.^23^, ^31^, ^32^ However, some patients can have more compromised tissue oxygenation with less severe hypoxaemia due to impaired perfusion, anaemia, or abnormal cellular oxygen utilisation.^33^ As a result, many guidelines recommend aiming for an SpO_2_ of 94–98% during acute resuscitation and in people with severe systemic illness or end-organ compromise (eg, sepsis, severe anaemia, meningitis, encephalitis, and heart failure).^23^, ^31^, ^33^

More recent guidelines^34^, ^35^, ^36^, ^37^, ^38^, ^39^, ^40^, ^41^, ^42^, ^43^, ^44^, ^45^, ^46^, ^47^, ^48^ are increasingly recognising that the optimal benefits of oxygen therapy are achieved through pulse oximetry measurement and monitoring and titration of oxygen therapy to avoid severe hypoxaemia without causing hyperoxic injury—particularly in at-risk populations (panel 2). These oxygen saturation targets are based on closely monitored clinical contexts in high-income countries and might not be generalisable to general hospitals in LMICs with low monitoring and response capacity and where the risk of undetected deterioration in oxygen saturation (and clinical deterioration more generally) is high. Whatever the context, oxygen therapy, like other treatments, should be given only when indicated, at the lowest effective dose, and for the shortest duration necessary, guided by pulse oximetry and clinical response.

Panel 2 **Oxygen saturation targets for oxygen therapy—what is the sweet spot?**

Data for optimal oxygen saturation targets mostly come from high-income countries and intensive care environments. Interpretation and practical implications could differ in other contexts.

**Neonates**

Strong evidence supports the use of target ranges (eg, SpO_2_ 90–94%) in extremely preterm infants on oxygen to prevent damage to their developing eyes (ie, retinopathy of prematurity) and lungs (ie, bronchopulmonary dysplasia).^34^ Restrictive use of oxygen during neonatal resuscitation is also recommended.^35^

**Children**

Trial data of alternative SpO_2_ targeting in infants with bronchiolitis have found equivalency in symptom resolution and adverse effects between groups in which an SpO_2_ of less than 90% was the target and groups in which an SpO_2_ of less than 94% was the target,^36^ with reduced frequency and duration of hospital admissions in the lower saturation target group.^37^, ^38^ In one trial in critically ill ventilated children in a well-resourced intensive care setting, equivalent clinical outcomes were noted between the group for whom an SpO_2_ of 88–92% was the target and the group in which an SpO_2_ of greater than 94% was the target.^39^ Minimal data are available for other hypoxaemic conditions of childhood.^40^

**Adults**

Guidelines for adults include a wide range of target SpO_2_, from 88% to 100%, with little consensus on the optimal target range. A review suggested a target of 90–94% in low-income and middle-income settings.^41^ A meta-analysis of 19 trials of acutely unwell adults with sepsis, stroke, myocardial infarction, trauma, and other critical illness suggested that the optimal target range spans SpO_2_ of 94–96%, (ie, the lower 95% CI limit and median baseline SpO_2_ in the liberal oxygen groups) because increased mortality in hospital was noted for people treated according to a liberal oxygen strategy compared with those treated according to a more conservative strategy (relative risk 1·21 [95% CI 1·03–1·43]).^42^ Results support an SpO_2_ target of 90–96% in critically unwell adults and giving oxygen only in instances of hypoxaemia.^43^ Subsequent trials of saturation targets for adults undergoing mechanical ventilation in intensive care units have mostly shown no difference in outcomes with higher or lower SpO_2_ targets, but results are mixed.^44^, ^45^, ^46^

Patients with chronic hypoxaemia due to conditions such as congenital heart disease or chronic obstructive pulmonary disease might require more individualised target SpO_2_ ranges to balance the risks of hypoxaemia with the risks of overtreatment (eg, hypercapnia).^24^, ^43^ Long-term oxygen therapy can improve and prolong life for some people with severe hypoxaemic chronic obstructive pulmonary disease.^47^ The benefits in other chronic respiratory conditions are less clear. Although more than 30 long-term oxygen therapy guidelines have been published, they vary greatly in recommendations and generally do not account for low-income and middle-income settings.^48^

SpO_2_= oxygen concentration in peripheral blood.

From a clinical perspective, oxygen saturation is a dynamic physiological marker that should be interpreted in the broader clinical context. An SpO_2_ of 90% will have vastly different clinical significance in an energetic infant with bronchiolitis during sleep or a stable patient with COPD than in a semi-conscious child with sepsis, an adult with head trauma, or a postoperative patient with previously normal oxygen saturations. SpO_2_ should be monitored over time, and interpretation of readings should account for the range of measurement uncertainty.^49^ For example, a spot-check SpO_2_ of 90% in a stable patient with acute respiratory infection could be regarded as representing potential concentrations of 88–92% and should be rechecked before any treatment decisions, whereas a downward SpO_2_ trend from 98% to 92% could be highly alarming in a deteriorating patient with asthma.

Emerging data suggest that hypoxaemia is a strong predictor of death in patients of all ages and various illnesses, and that the risk of death is proportional to the degree of hypoxaemia.^50^, ^51^, ^52^, ^53^, ^54^, ^55^ The strong association between hypoxaemia and poor outcomes means that pulse oximetry can be used to identify patients who need extra attention, care, and monitoring, making it a useful tool for risk stratification and decision making at all levels of the health system—particularly in patients with moderate hypoxaemia, who might not always meet criteria for admission or oxygen therapy but in whom careful reassessment and close follow-up are warranted.^56^

Pulse oximetry is therefore the key tool in guiding the initiation, monitoring, targeting, weaning, and cessation of oxygen therapy and has additional value in screening and triage (eg, risk stratification), diagnostic stratification (eg, of severe *vs* non-severe pneumonia), and prognostication (eg, of long-term mortality risk in COPD). From a broader programme, policy, and public health perspective, oxygen saturation data can inform estimates of population oxygen need and help to prioritise oxygen service actions across the health system. In this Commission, we use data for the population prevalence of hypoxaemia and risk of death to inform oxygen needs and coverage estimates and to discuss the role and value of pulse oximetry at different health service levels.

## Quantifying global medical oxygen need, the coverage gap, and associated costs

### Oxygen need

*“Even though I am a doctor, I never thought in my life that oxygen security is the mainstay of everything. It should be available every time, everywhere, in every hospital, small or large … COVID taught me that oxygen is a big issue. Without oxygen, no one can survive.”*

*—Doctor, Bangladesh*

Everyone needs access to quality oxygen services, including clinical assessment guided by pulse oximetry and appropriate oxygen therapy when they need it. In this section, we quantify the number of people who need medical oxygen annually (ie, the oxygen need), the number who need oxygen but do not get it (ie, the oxygen coverage gap), and the cost required t o close the gap between 2025 and 2030 (appendix 1 pp 33). Our estimates are intended to convey the magnitude of oxygen need for patients with various medical and surgical conditions, reveal systemic inequities and vulnerabilities in oxygen systems, and provide guidance on the funding needed to close the gaps. We also provide data on oxygen needs due to COVID-19 as an illustration of how oxygen needs during pandemics and other emergencies can rise exponentially and overload service capabilities. Our key findings are summarised in panel 3.

Panel 3 **Global medical oxygen need and the oxygen coverage gap—key findings**

• 5 billion people—ie, 60% of the world's population—do not have access to affordable, high-quality medical oxygen services. The oxygen coverage gap is greatest in LMICs, in rural and remote communities, and in low-income households.
• Around 364 million people need oxygen for acute medical and surgical conditions annually, including 25 million people with respiratory tract infections, 17 million people with traumatic injuries, 5·4 million neonates, and 259 million people undergoing surgery. An additional 9·2 million people with chronic obstructive pulmonary disease need long-term oxygen therapy. 82% of people needing oxygen therapy live in LMICs.
• Humanitarian emergencies can substantially increase oxygen need and further compromise oxygen systems, as exemplified by the 52 million additional people who needed oxygen for COVID-19 in 2021, a surge in demand that completely overwhelmed the oxygen service capacity in many countries.
• In LMICs, 70% of people who need oxygen for acute medical or surgical care do not receive it, and almost no one who needs long-term oxygen therapy receives it. The coverage gap for medical oxygen far exceeds the treatment gaps for HIV/AIDS (23%) and tuberculosis (25%).
• Major contributors to the oxygen coverage gap include people not reaching a health facility; facilities lacking basic oxygen service capacity; missed identification of oxygen need due to unavailability of pulse oximeters; interrupted, unsafe, or otherwise low-quality oxygen care; and the high costs of oxygen services borne by patients.
• Oxygen is a highly cost-effective intervention. Closing the oxygen coverage gap for acute medical and surgical needs in LMICs will require an additional investment of US$34·0 billion between 2025 and 2030, with the highest need in south Asia ($2·6 billion per year), east Asia and the Pacific ($1·8 billion per year), and sub-Saharan Africa ($1·7 billion per year).
• Medical oxygen needs are rising. Greater efforts should be put into public health prevention programmes that target major risks driving the demand for medical oxygen—eg, smoking prevention and cessation, road safety campaigns, improved diets, reducing indoor and outdoor air pollution, increased vaccine coverage, and curbing climate change.

LMICs=low-income and middle-income countries.

Estimation of acute medical, surgical, and long-term oxygen therapy needs involved systematic reviews of existing literature, integration of Global Burden of Disease (GBD) data, and expert consultation to inform model estimates. People with acute medical illness need oxygen therapy for a short period (days to weeks). In such patients, low-flow oxygen therapy is usually provided but treatment can also involve non-invasive and invasive forms of ventilation. We estimated acute medical oxygen need by establishing the incidence of hypoxaemia in key conditions based on previous systematic reviews.^57^, ^58^, ^59^, ^60^, ^61^ We then combined these data with the incidence of the corresponding conditions according to GBD data^62^ to calculate the number of people hospitalised with acute hypoxaemia annually. We estimated the surgical oxygen need based on data from the *Lancet* Commission on Global Surgery,^63^ which we updated with more recent GBD data.^62^ Our estimates accounted for how patients receiving sedative anaesthesia require short-term (minutes to hours) oxygen therapy as supplemental low-flow or invasive ventilation, or both. We estimated long-term oxygen therapy needs using GBD data for COPD prevalence and data from a targeted review of patients with chronic respiratory failure, who use low-flow oxygen therapy in the home and community setting for long periods of time (months to years). For each of these populations, we counted only people who would very likely need oxygen therapy, and thus our estimates of oxygen needs are conservative.

We then extrapolated each of these estimates to calculate the minimum volume of oxygen required to meet the unmet need, and used historical GBD datasets to show the change in need over time. Our assumptions were reviewed and revised iteratively with feedback from clinicians and epidemiologists among the Commissioners and Commission advisers (appendix 1 p 33).

#### Acute oxygen need

*“I remember when I got to the emergency room, my saturation was 80%. I had a blackout in front of my eyes. I thought I would die. I was sweating. I felt like there was no life in my hands or feet. I felt much better when I got on oxygen and my symptoms got better and I thought I would come out of it. It gave me hope.”*

*—Young patient with acute respiratory failure, Pakistan*

**Figure 1:**
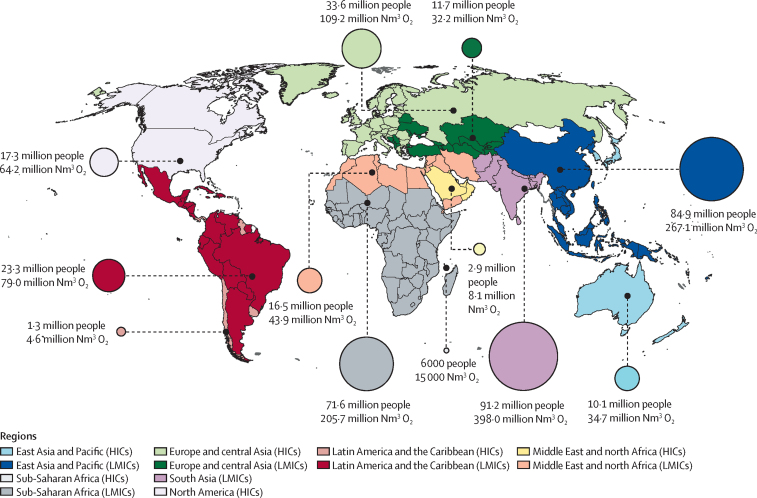
Location of people with acute medical and surgical oxygen needs in 2021, and minimum volume of oxygen required to meet need, by World Bank region Note that this figure excludes oxygen requirements related to COVID-19. Oxygen need is represented by the circles, the sizes of which are proportional to the number of people in that region who need medical oxygen therapy. Minimum volume of oxygen required to meet need was calculated using data for recommended and usual flow rates and duration for various conditions and assumes no inefficiencies in oxygen use and no wastage or inefficiencies in upstream oxygen production, supply, and distribution. HICs=high-income countries. LMICs=low-income and middle-income countries. Nm^3^=normal cubic metres.

We estimate that 364 million people needed a minimum of 1·2 billion m^3^ medical oxygen oxygen for acute medical and surgical conditions in 2021 (table 1). This estimate includes around 25 million children and adults with respiratory tract infections, 17 million patients with traumatic injury, 5 million neonates, and 259 million people undergoing surgery (Table 1, Table 2). 299 million [82%] of the 364 million people with an acute medical or surgical need for oxygen live in LMICSs. In keeping with overall population size, the acute oxygen need is highest in south Asia, east Asia and the Pacific, and sub-Saharan Africa (figure 1).

**Table 1:** Estimated global number of people needing acute, surgical, and long-term medical oxygen therapy, additional oxygen required due to COVID-19, and minimum volume of oxygen required to meet need, 2021

		**Acute medical need**		**Surgical need**		**Long-term need**		**COVID-19 need (2021)**	
		n, millions (uncertainty interval)	Volume of oxygen required, millions of Nm^3^ (uncertainty interval)	n, millions (uncertainty interval)	Volume of oxygen required, millions of Nm^3^ (uncertainty interval)	n, millions (uncertainty interval)	Volume of oxygen required, millions of Nm^3^ (uncertainty interval)	n, millions (uncertainty interval)	Volume of oxygen required, millions Nm^3^ (uncertainty interval)
Global		105·4 (39·6–211·0)	1132·0 (418·6–2264·2)	259·0 (230·0–288·4)	114·8 (101·9–127·8)	9·2 (5·9–13·5)	3178·8 (2044·2–4683·8)	52·4 (25·1–89·8)	1913·2 (910·8–3284·8)
High-income countries		18·3 (6·1–37·7)	200·1 (66·3–411·6)	46·8 (42·4–51·1)	20·7 (18·8–22·7)	2·9 (1·9–4·2)	1015·1 (664·7–1472·7)	8·7 (6·3–11·8)	319·5 (230·7–433·2)
Low-income and middle-income countries		87·1 (33·5–173·3)	931·9 (352·3–1852·5)	212·2 (187·6–237·3)	94·0 (83·1–105·1)	6·2 (4·0–9·3)	2163·6 (1379·5–3211·1)	43·7 (18·8–78·1)	1593·7 (680·1–2851·7)
World Bank region									
	East Asia and Pacific	23·9 (8·2–49·0)	270·4 (92·2–553·5)	71·1 (65·3–76·9)	31·5 (28·9–34·1)	3·2 (2·0–4·8)	1117·2 (707·4–1670·1)	4·7 (3·1–6·7)	170·6 (113·4–244·9)
	Europe and central Asia	11·8 (4·0–24·1)	126·6 (42·4–259·5)	33·6 (29·7–37·4)	14·9 (13·1–16·6)	1·8 (1·1–2·6)	619·3 (397·5–915·1)	9·2 (5·9–13·7)	339·6 (216·0–504·5)
	Latin America and the Caribbean	7·2 (2·4–14·9)	75·8 (25·6–156·5)	17·4 (15·3–19·4)	7·7 (6·8–8·6)	0·7 (0·4–1·0)	225·7 (142·6–338·0)	6·1 (3·6–9·2)	222·7 (132·8–339·1)
	Middle East and north Africa	4·6 (1·7–9·2)	45·5 (16·1–91·9)	14·8 (12·8–16·7)	6·5 (5·7–7·4)	0·3 (0·2–0·5)	110·6 (69·1–167·5)	3·9 (1·7–6·8)	141·5 (61·1–248·6)
	North America	5·3 (1·8–10·8)	58·8 (19·6–120·6)	12·0 (11·2–12·9)	5·3 (5·0–5·7)	1·0 (0·7–1·4)	340·6 (234·2–469·7)	1·9 (1·5–2·3)	68·4 (53·7–85·5)
	South Asia	32·1 (12·5–63·3)	371·8 (136·6–745·8)	59·1 (51·8–66·7)	26·2 (23·0–29·6)	1·8 (1·2–2·7)	639·7 (415·3–932·4)	15·4 (6·3–28·1)	562·3 (224·9–1028·9)
	Sub-Saharan Africa	20·6 (9·0–39·6)	183·1 (86·1–336·4)	51·1 (44·0–58·3)	22·6 (19·5–25·8)	0·4 (0·2–0·6)	125·6 (78·2–190·9)	11·3 (3·1–22·9)	408·1 (108·9–833·4)

The minimum volume of oxygen required to meet need was calculated using data for recommended and usual flow rates and duration for various conditions and assumes no inefficiencies in oxygen use and no wastage or inefficiencies in upstream oxygen production, supply, and distribution. We have converted the oxygen gaseous flow rates and duration that we calculated to volume in Nm^3^, but true volume will depend on actual pressure and temperature. Nm^3^=normal cubic metres.

**Table 2:** Estimated number of patients needing oxygen for acute medical conditions globally and minimum volume of oxygen required to meet need, 2021

	**People with acute hypoxaemia needing oxygen, millions (uncertainty interval)**	**Minimum oxygen volume to meet acute hypoxaemia need, millions of Nm^3^ (uncertainty interval)**
Neonatal encephalopathy	0·5 (0·2–0·8)	1·0 (0·5–1·7)
Neonatal lower respiratory infections	0·7 (0·1–1·6)	1·6 (0·3–3·6)
Preterm birth	3·2 (1·7–4·9)	12·6 (6·9–19·6)
Neonatal sepsis and other infections	1·0 (0·5–1·5)	2·2 (1·2–3·4)
Asthma	2·5 (0·6–5·6)	27·8 (8·2–60·8)
Diarrhoea	6·4 (1·7–15·0)	57·6 (15·3–133·4)
HIV/AIDS	1·1 (0·6–1·9)	19·5 (10·9–32·8)
Malaria	3·8 (1·4–8·6)	21·6 (8·4–47·7)
Nutritional deficiencies	1·3 (0·4–3·1)	5·2 (1·6–12·1)
Encephalitis	0·4 (0·2–0·7)	5·7 (2·7–10·1)
Meningitis	0·5 (0·2–1·0)	6·6 (2·8–13·2)
Lower respiratory infections	24·7 (11·5–44·4)	357·5 (147·1–676·1)
Trauma or injury	17·4 (5·8–36·5)	199·0 (63·5–421·5)
Tuberculosis	0·9 (0·3–1·8)	13·5 (4·7–29·7)
Typhoid	0·1 (0·0–0·3)	0·8 (0·3–1·7)
Dengue	0·7 (0·1–2·2)	6·5 (0·7–21·9)
Measles and pertussis	1·5 (0·7–3·0)	6·0 (2·6–11·8)
Cardiovascular disease	26·7 (8·4–56·3)	243·6 (76·4–513·3)
Sepsis (not otherwise classified)	4·7 (2·7–6·9)	70·6 (41·3–104·4)
COVID-19	52·4 (25·1–89·8)	1913·2 (910·8–3284·8)
Other conditions	7·6 (2·4–14·9)	73·0 (23·1–145·3)
Overall	157·8 (64·6–300·8)	3045·2 (1329·4–5549·0)

The minimum volume of oxygen required to meet need was calculated using data for recommended and usual flow rates and duration for various conditions and assumes no inefficiencies in oxygen use and no wastage or inefficiencies in upstream oxygen production, supply, and distribution. We have converted the oxygen gaseous flow rates and duration that we calculated to volume in Nm^3^, but true volume will depend on actual pressure and temperature. Nm^3^=normal cubic metres.

**Figure 2:**
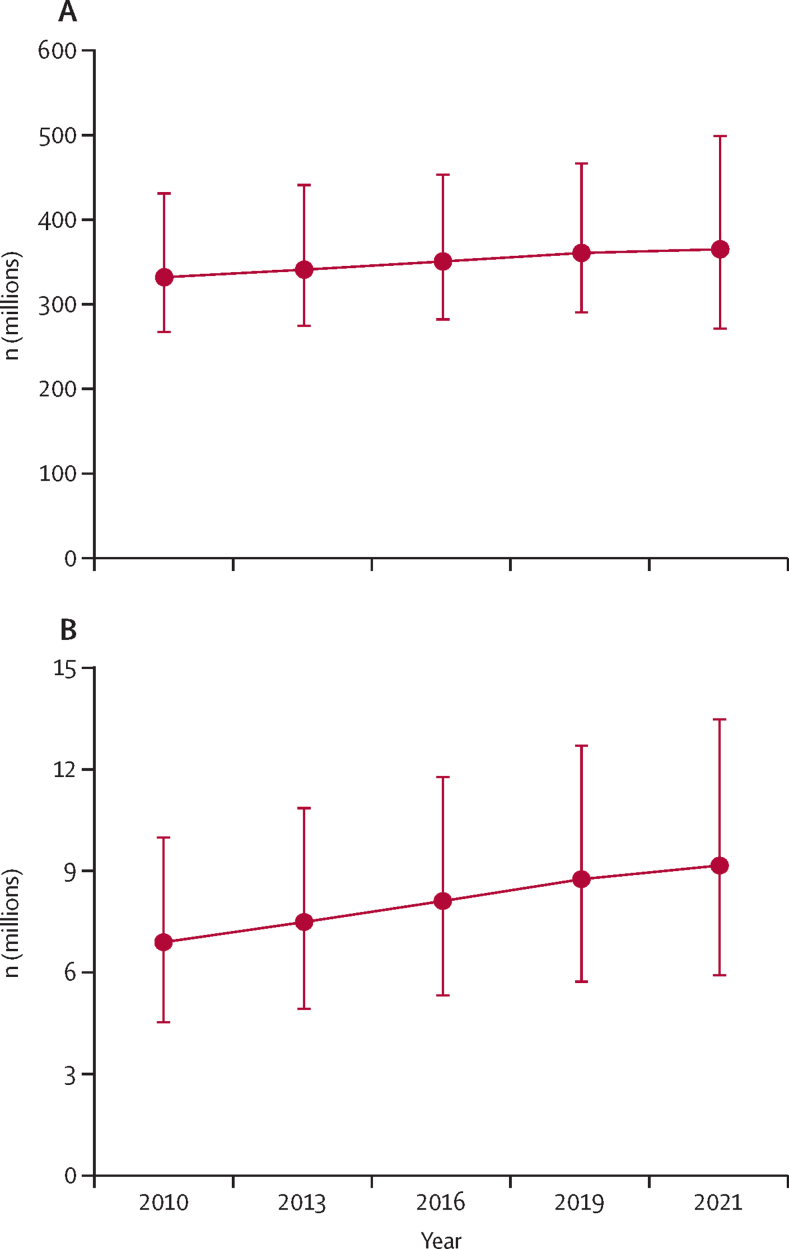
Trends in estimated global oxygen need (2010–21) for acute medical and surgical oxygen therapy (A) and long-term oxygen therapy (B) Data are from the Global Burden of Disease.^63^ Oxygen need related to COVID-19 is excluded. Error bars represent uncertainty intervals.

The number of people who needed medical oxygen for acute medical or surgical conditions increased from 331 million in 2010 to 364 million in 2021 (excluding COVID-19)—an increase of 10%. This rise has primarily been driven by increasing surgical volume, with the need for oxygen for acute medical conditions remaining broadly stable despite rising populations (figure 2A; appendix 1 p 115). The stability in oxygen need for acute medical conditions since 2010 can be largely attributed to prevention of communicable diseases, and of respiratory infections specifically, including widespread roll-out of pneumococcal vaccines for children.^64^ The introduction of these vaccines reduced hospitalisation for pneumonia in children by around 21% globally,^65^ and emerging data suggest similar reductions could be achieved with the new respiratory syncytial virus vaccines.^66^, ^67^ Opportunities for prevention of acute medical and surgical oxygen need include expanding coverage of pneumococcal vaccines from 60% to the global target of 90%, rapid and equitable roll-out of respiratory syncytial virus vaccines, preventing child malnutrition, reducing air pollution, and preventing injury though road safety initiatives and reducing alcohol, drug, and violence-related trauma.

Estimates of acute medical oxygen need are limited by a scarcity of data for medical oxygen need related to many non-respiratory conditions (particularly among adults) and of data from geographical regions outside sub-Saharan Africa.^61^ Global surgery needs estimates are based on data for surgical volumes by disease type from one high-income country (New Zealand) and might not translate to LMICs.^63^ Much of the global surgical need in LMICs is not met: every year, there are an estimated 150 million required surgical procedures that are not done.^63^

#### Long-term oxygen need

*“It is a Herculean task for a family caring for someone who needs oxygen at home. We are really on our own, with little support from the government.”*

*—Daughter of person on long-term oxygen therapy, Pakistan*

An estimated 9·2 million people with COPD meet the criteria for long-term oxygen therapy annually. Each of these people requires a continuous portable oxygen supply. 6·2 million (67%) of the 9·2 million people who need long-term oxygen therapy annually live in LMICs. Of these 9·2 million people, 3·2 million (35%) are in east Asia and the Pacific and 1·8 million (20%) are in south Asia.

The number of people who needed long-term oxygen increased by 33% (ie, from 6·9 million to 9·2 million) between 2010 and 2021 (figure 2B, appendix 1 p 115), driven by the long-term effects of smoking on an ageing population and exposure to indoor and outdoor air pollution, and exacerbated by climate change and urbanisation.^68^ The prevalence of COPD is projected to increase by 23% between 2020 and 2050,^68^ with disproportionately large increases in women and in LMICs, and so inequities in access to long-term oxygen therapy are likely to grow. Public health programmes that reduce smoking and burning of biomass indoors, improve diets and air quality, and protect ageing populations and overcrowded communities from the effects of extreme heat and climate change could help to reduce the anticipated growth in long-term oxygen therapy needs (see Sweden case study; appendix 2).^68^

Our long-term oxygen estimates focused on COPD, the condition for which the strongest data showing treatment benefit and the most consistent diagnostic classification and eligibility criteria are available. However, there is great variation in COPD guidelines and long-term oxygen therapy eligibility between and within countries and regions. Most guidelines come from high-income and upper-middle income countries and as a result they probably under-recognise chronic lung injuries from diseases such as tuberculosis and pneumonia.^48^

#### Oxygen needs during humanitarian emergencies

*“When we arrived, there were a lot of patients—it was very crowded—but they took us in and gave [my husband] an oxygen mask and big green cylinder. Five to six patients were sharing one cylinder.”*

*—Wife of elderly man with COVID-19, Philippines*

Medical oxygen needs can rise exponentially during emergencies and rapidly overwhelm oxygen service capacity. In the context of respiratory epidemics and pandemics, such as COVID-19 and severe acute respiratory syndrome (SARS), increased oxygen need was driven by sudden spikes in severe respiratory illness in adults, with patients often requiring more and longer oxygen support than usual respiratory infections. In 2021, 52 million people needed a minimum of 1·9 billion Nm^3^ medical oxygen due to COVID-19—more than the required volume for all other acute needs. Oxygen access during the pandemic was severely compromised even in previously high-functioning health facilities. In a study of 64 intensive care units (ICUs) in sub-Saharan Africa, 634 (45%) of the 1416 patients who died from COVID-19 never received oxygen.^69^ The COVID-19 pandemic resulted in an estimated 15·9 million excess deaths globally between Jan 1, 2020, and Dec 31, 2021, with most deaths in regions with the weakest health systems, including 4·4 million in south Asia, 2·4 million in sub-Saharan Africa, and 2·3 million in Latin America and the Caribbean.^1^

In conflict settings and natural disasters, increased oxygen need due to trauma and communicable diseases is often accompanied by destruction of health services and disruption of essential supply chains and electricity. Gaza, Palestine and Darfur, Sudan provide stark illustrations of these issues. Acute armed conflict in Gaza has destroyed most health facilities and disrupted fuel supplies, compromising the remaining oxygen plants,^70^ and protracted conflict in Darfur has weakened health services and made efforts to improve oxygen services much harder.^71^

### Oxygen coverage gaps in LMICs

*“The hospitals in western Kenya didn’t have the right equipment—they were basically just testing for malaria—and they didn’t have oxygen either.”*

*—Son of person who died from COVID-19, Kenya*

To understand the status of oxygen service coverage and quantify the coverage gap, we systematically reviewed published and grey literature from Jan 1, 2000 onwards, and obtained datasets for re-analysis (including all available Service Provision Assessment data). We focused on LMICs as defined by the World Bank (we assumed that high-income countries had negligible gaps in oxygen coverage) and extracted data for oxygen service readiness (eg, pulse oximeter and oxygen availability, functionality, and adequacy to meet need), the quality of oxygen service provision (eg, pulse oximetry coverage, oxygen coverage in people with hypoxaemia, and adherence to guidelines), and oxygen-related costs (eg out-of-pocket costs and costs to facilities). We calculated meta-estimates for key pulse oximetry and oxygen availability and coverage indicators. We then combined these data with estimates of oxygen need to map oxygen service coverage against the effective coverage cascade. Additional data for pulse oximeter and oxygen availability and coverage are in appendix 1 (p 116).

We focused our analysis on the medical oxygen gap for acute medical and surgical needs, in recognition of the fact that most LMICs do not have data, guidelines, or programmes for long-term oxygen therapy.^72^ Given the challenges to long-term oxygen therapy access even in the world's wealthiest countries,^48^ we expect that long-term therapy is available for very few of the 6·2 million people in LMICs who need it.

**Figure 3:**
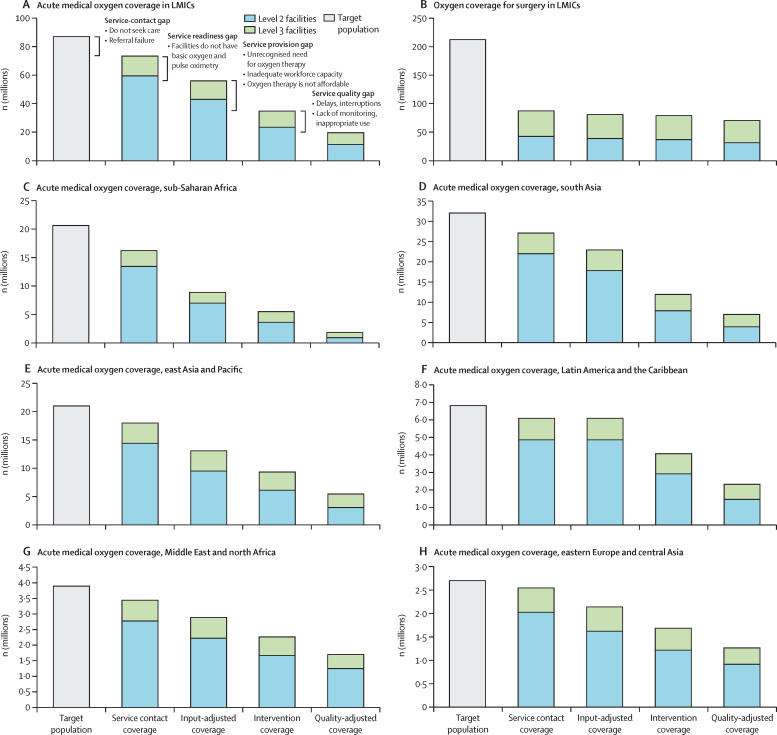
Oxygen coverage cascade for people with acute medical and surgical conditions This figure shows the effective oxygen coverage cascade for acute medical (A) and surgical (B) purposes in LMICs; and for acute medical purposes in LMICs in sub-Saharan Africa (C), south Asia (D), east Asia and the Pacific (E), Latin America and the Caribbean (F), the Middle East and north Africa (G), and eastern Europe and central Asia (H). The cascade depicts the number of people who need oxygen therapy (ie, the target population), who attend a health service that should offer oxygen services (ie, service contact coverage), who attend a health service that has oxygen available (ie, input-adjusted coverage), who receive oxygen therapy (ie, intervention coverage), and who receive oxygen therapy that is safe and appropriate (ie, quality-adjusted coverage or patient access). (A) details potential reasons for the oxygen coverage gap at each step of the cascade. Long-term oxygen need is not depicted. LMICs=low-income and middle-income countries.

Our analysis showed major gaps and inequities in oxygen service coverage for people in LMICs. Across all LMICs, we estimated that only 89·3 million (30%) of the 299·3 million people who need oxygen for acute medical or surgical conditions receive adequate oxygen therapy (figure 3). This medical oxygen coverage gap of 70% far exceeds treatment gaps for HIV/AIDS (23%) and tuberculosis (25%).^73^, ^74^

For people with acute medical conditions, oxygen coverage was lowest in sub-Saharan Africa (where 1·8 million [9%] of 20·6 million people who need oxygen receive it), followed by south Asia (7·0 million [22%] of 32·1 million), east Asia and the Pacific (5·5 million [26%] of 21·0 million), Latin America and the Caribbean (2·3 million [34%] of 6·8 million), the Middle East and north Africa (1·7 million [44%] of 3·9 million), and eastern Europe and central Asia (1·3 million [48%] of 2·7 million). The oxygen coverage gap is due to inter-related challenges that affect all parts of the oxygen coverage cascade, including people not reaching health facilities that should have oxygen service capacity (ie, the service-contact gap), attending facilities that do not have basic oxygen service capacity (ie, the service readiness gap), not receiving oxygen therapy when needed (ie, the service provision gap), and receiving inappropriate or unsafe oxygen therapy (ie, the service quality gap). The service-contact gap is largely due to delaying or not seeking care and lower-level facilities failing to refer patients appropriately. Among the 59·5 million people in LMICs who need oxygen therapy and reach a general hospital (ie, a level 2 facility), only 11·5 million (19%) receive adequate oxygen therapy. Most patients experience substantial deficits in service readiness, service provision, and service quality, particularly in sub-Saharan Africa where only 900 000 (7%) of the 13·4 million people who need medical oxygen receive adequate therapy in general hospitals. Of the 13·9 million people who attend tertiary hospitals (ie, level 3 facilities), 8·0 million (58%) receive adequate oxygen therapy. The most common reason for not receiving adequate therapy at these centres is low quality of oxygen care.

For people requiring surgery, the oxygen coverage gap is primarily due to the low numbers of people accessing surgery in the first place (ie, only 87·0 million [41%] of the 212·2 million people in LMICs estimated to need surgery each year access it). The *Lancet* Commission on Global Surgery estimated that 5 billion people globally do not have access to safe, affordable surgical and anaesthetic care, with only 6% of surgeries globally occurring in the lowest-income countries, where a third of the world's population resides.^63^ Among people undergoing surgery within a health facility in LMICs, we estimated that 69·7 million (80%) of 87·0 million received adequate oxygen therapy and pulse oximetry measurement and monitoring.

These oxygen service coverage estimates vary across and within regions, and prioritisation and planning should take into account local data and context. To address these gaps, oxygen service investment should prioritise small and remote health facilities serving low-income populations and go beyond oxygen supply and equipment to also address major gaps in referral pathways and quality of oxygen care throughout the patient journey.

### Costing the oxygen coverage gap

*“A COVID patient used about four cylinders per day so we ran out quickly and asked the health ministry to send more, but they didn’t have enough money.”*

*—Doctor, Sierra Leone*

To close the oxygen coverage gap and build sustainable oxygen systems that ensure oxygen access for all, national governments and global health funders need to increase investments. A crucial point for this investment case is that oxygen is a highly cost-effective intervention. For children with pneumonia, the median cost per disability-adjusted life year (DALY) averted is $59 (range $21–225), and $44 when the costs to install solar systems are excluded.^75^, ^76^, ^77^, ^78^ Oxygen therapy is thus on par with routine childhood immunisation programmes in terms of cost-effectiveness ($64 per healthy life year gained,^79^ <$100 per DALY averted).^80^ An analysis using the Lives Saved Tool for Bangladesh, Chad, and Ethiopia estimated that scaling up oxygen access to 90% would produce similar mortality reductions to oral antibiotics.^81^

Therefore, there should be no question as to whether investment in oxygen-system strengthening is value for money. Rather, the focus should be on how much funding is needed and how this money would be most effectively spent. We synthesised data from a rapid scoping review of academic and grey literature (appendix 1 p 19) and national oxygen plans, to estimate the average cost a facility pays for 1 L of oxygen in each region. We multiplied this cost by our estimate of the regional oxygen coverage gap (expressed in volume of oxygen required to meet need and adjusted for realistic inefficiencies in oxygen production, supply, distribution, and use; appendix 1 p 75).

**Figure 4:**
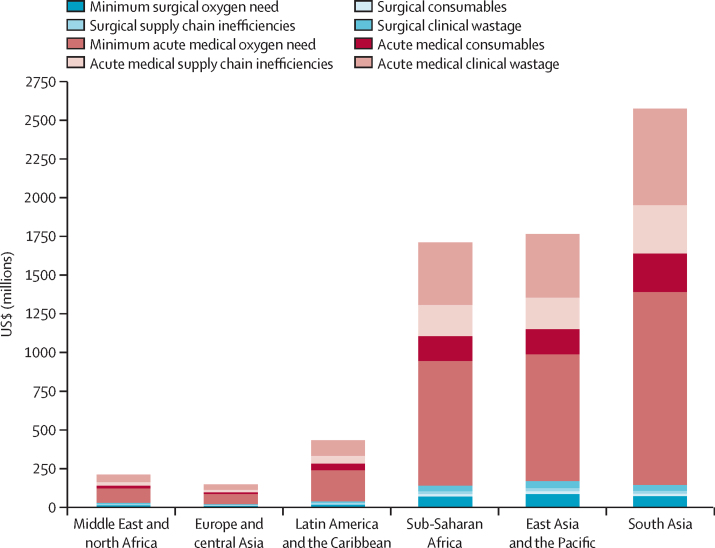
Annual cost to close the acute medical and surgical oxygen gaps in low-income and middle-income countries The minimum cost of the medical and surgical oxygen need is the cost to fill the oxygen coverage gap, based on recommended treatment. We inflated this cost to reflect actual practice and included inefficiencies in the system, clinical wastage, and additional consumables in our estimates (appendix 1 p 78). Supply chain inefficiencies refer to leakages in oxygen delivery systems and losses during production, distribution, and storage. Clinical wastage is the use of higher flow rates for longer periods than recommended, and treatment of patients without a clinical need for oxygen. Consumables includes the cost of pulse oximetry, nasal cannulas, masks, and staff time.

We estimated that the additional investment needed to close the gap in acute medical and surgical oxygen need is $6·8 billion a year—ie, $34·0 billion to 2030. This figure represents the shortfall in capital and operational costs for oxygen systems under realistic operating conditions, and incorporates the need to increase oxygen production capacity and to power, distribute, maintain, and repair existing oxygen systems.The annual cost gap is highest in south Asia ($2·6 billion), follwed by east Asia and the Pacific ($1·8 billion), sub-Saharan Africa ($1·7 billion), Latin American and the Carribean ($436 million), the Middle East and north Africa ($212 million), and Europe and central Asia ($148 million; figure 4). There are substantial opportunities to increase the cost-efficiency of these investments through improvements in clinical management practices. For example, a clinical quality-improvement project in India reduced oxygen consumption in a neonatal unit by more than 50%, and the proportion of the running budget of the facility dedicated to oxygen from 79% to 38%.^82^ Similar results were noted in a study of Nigerian paediatric wards.^83^ Additional cost-efficiencies could be achieved with oxygen-conserving devices, improved maintenance of oxygen piping systems,^83^, ^84^ use of conservative SpO_2_ targets,^85^, ^86^, ^87^ and strengthening of referral systems and emergency care more broadly.

Acute medical needs account for 92% of the cost gap ($6·3 billion). The smaller cost of closing the surgical need gap ($559 million per year) reflects the short duration of oxygen therapy received by these patients, and is driven by surgeries that should happen but do not, rather than gaps in oxygen provision for surgeries that are done. The *Lancet* Commission on Global Surgery estimated that it would cost $16–31 billion annually between 2012 and 2030 to achieve full surgical coverage, with oxygen accounting for a critical component of this provision.^63^

We estimate that an additional $6·8 billion (uncertainty interval 2·9 billion–12·2 billion) was needed in 2021 to fund the surge in oxygen need due to COVID-19 in LMICs. In terms of long-term oxygen therapy for COPD, evidence from high-income settings shows that systems based on mobile oxygen concentrators are more cost-efficient than oxygen cylinders, but still cost between $250 and $10 000 per patient annually.^88^, ^89^, ^90^, ^91^, ^92^ The USA spends more than $2 billion on long-term oxygen therapy each year^93^—more than the entire domestic health expenditure of more than 100 governments in 2021–22.^94^ When the need for a reliable home power source is factored in, there are additional and inequitable cost burdens to patients for the provision of long-term oxygen therapy.^95^, ^96^ 675 million people globally did not have electricity access in 2021,^97^ which would necessitate frequent oxygen cylinder refills (and high out-of-pocket costs as a result) for many patients on long-term oxygen therapy in low-income settings. Based on the scarce data from LMICs, we estimate that the annual cost to meet the long-term oxygen needs for patients with COPD is in the region of $3–10 billion a year—a cost that will be out of reach for many countries. Prevention of long-term oxygen need in LMICs should be an urgent public health priority, and the establishment of long-term oxygen therapy programmes should be considered alongside countries’ other essential health package priorities.

## Inequities in medical oxygen access in LMICs

*“The hospital was 80 km from home and we could not afford the 3000 taka to rent a private bus. So, we took the public bus, which cost 400 taka per person each way, but on the way the baby became listless and I was frightened he would die.”*

*— Parents of an unwell baby, Bangladesh*

SDG3, the SDG for health, seeks to “ensure healthy lives and promote well-being for all at all ages”.^98^ Health services play an essential role in promoting the wellbeing of individuals and communities by providing prevention, diagnostic, and treatment services. The core principle that connects health services with the wellbeing of populations is universal health coverage (UHC), which ensures that “all people have access to the full range of quality health services they need, when and where they need them, without financial hardship”.^99^ Governments have committed themselves to achieving UHC through SDG 3.8. Two broad elements are required to achieve UHC: the presence of accessible, service-ready health facilities and the provision of high-quality care to all patients without catastrophic costs.

Equitable oxygen provision is clearly an important component of UHC. In this section, we discuss both of the elements of UHC as they relate to oxygen services. We present evidence on oxygen service availability and readiness, and the quality of oxygen care provided to people (appendix 1 pp 74, 113), which is summarised in panel 4. We recognise that broad challenges to care-seeking and access to health care, such as geographical distance and transport, also apply to patients’ ability to access oxygen services and are also highly inequitable. A scarcity of data for care-seeking and accessibility to oxygen services prevented a detailed analysis of these issues, highlighting the importance of focusing on UHC^98^ and the need for more and better data. In recognition of the different international approaches to achieving UHC, we use the term UHC broadly to describe an inclusive approach to prioritisation and implementation of essential health service and benefits packages.

Panel 4 **Inequities in medical oxygen access in LMICs—key findings**

• Pulse oximeters are available in 54% of general hospitals (level 2) and 83% of tertiary hospitals (level 3), but only 10% of primary health-care facilities (level 1) in LMICs. Even when oximeters are available, pulse oximetry is performed infrequently in unwell patients attending general hospitals (19%) or tertiary hospitals (54%), and is almost never done in primary care settings (0%).
• Oxygen is available in 58% of general hospitals, 86% of tertiary hospitals, and 12% of primary health-care facilities in LMICs. However, oxygen stockouts and interruptions occur in 93% (95% CI 87–97) of primary health-care facilities, 45% (43–47) of general hospitals, and 25% (12–39) of tertiary hospitals. These estimates of the presence of oxygen overestimate oxygen availability given that functionality is rarely assessed (ie, oxygen might be present but not working), and that presence does not mean that what is available is sufficient to meet demand.
• Only around half of hospitalised patients with hypoxaemia receive oxygen therapy in LMICs (45% in general hospitals; 79% in tertiary hospitals). Patients who receive oxygen therapy often face delays, interruptions, inappropriate administration, and inadequate pulse oximetry measurement and monitoring, all of which substantially compromise the quality and effectiveness of oxygen care.
• The greatest inequities in pulse oximetry and oxygen service delivery are for people—particularly children—attending small health facilities in rural areas, especially in sub-Saharan Africa and south Asia. Estimates for facilities as a whole can cover up large inequities between wards within facilities. For example, availability of oxygen tends to be lower in paediatric and other ward areas than in operating theatres and critical care settings.
• Interventional studies have shown that pulse oximetry and oxygen care can be improved with low-cost capacity-building strategies. However, we note no substantial change in the availability or use of pulse oximetry and oxygen since 2000.
• Oxygen therapy is highly acceptable to patients, and fear or knowledge-related hesitancy about therapy is amenable to education and demonstration of effect. However, high out-of-pocket costs for oxygen therapy remain a major barrier to care.

LMICs=low-income and middle-income countries.

### Readiness of oxygen services at health facilities

*“I don’t want any future generations of doctors having to decide like God who lives and who dies, because that is what we had to do when there wasn’t enough oxygen.”*

*—Doctor, Ethiopia*

What does it mean for a health facility to be “oxygen service ready”? Clearly the availability of oxygen equipment is important, but availability is not enough to provide safe and effective oxygen services. Oxygen equipment also needs to be adequate, functional, and in the right location, and to have the requisite consumables and delivery interfaces appropriate for the patient. Facilities also need adequate staff capacity, oxygen to be included in clinical and equipment management protocols, and a fair pricing structure (panel 5; appendix 1 p 101). Medical transport settings will have similar requirements, with the added need for mobility. Critical care settings will have additional requirements, including the need for continuous monitoring, higher respiratory support, the ability to mix and humidify oxygen and medical air, and additional staffing and protocol requirements relative to the level of care provided. Importantly, all these resources will only function effectively when they are embedded in a high-quality care system that provides effective patient care from arrival at a health facility to discharge, and throughout all the transitions between.

Panel 5 **Key elements of oxygen-ready health facilities**

• Functional, good-quality pulse oximeters (or integrated monitoring) should be available in every area where patients are clinically assessed or admitted in sufficient quantities to meet need (considering variations in patient volume, age groups, and acuity of illness).
• Functional oxygen delivery points should be available in every area where patients are clinically stabilised or admitted, including reliable supply and appropriate delivery devices.
• A continuous, reliable oxygen source that supplies all oxygen outlets is needed, with a back-up oxygen source for unplanned malfunction or in times of extreme need. The ability to meet need should be based on peak patient numbers, not mean numbers, in recognition of daily and seasonal variations in admissions and oxygen need. Medical-grade oxygen needs to be available 24 h a day, even in extreme environmental conditions (eg, temperatures >40°C, 95% relative humidity).
• Oxygen delivery equipment and consumables should be available and adequate and appropriate for the population and clinical need. Such equipment includes age-appropriate nasal cannulae and masks, pressure regulators, and flow meters, and tools to enable warmed humidification, air–oxygen mixing, and non-invasive and mechanical ventilation in critical care settings.
• Adequately trained nurses (or similar) are needed to regularly monitor patients and trained doctors (or similar) are necessary for medical oversight.
• Prompt biomedical engineer or technician support should be available in case of major oxygen equipment failure and a routine equipment management plan that includes oxygen-related equipment should be in place.
• Clinical protocols containing guidance on oxygen therapy for all relevant age groups are required. These protocols should preferably be integrated into broader clinical guidelines (eg, emergency triage and treatment, pneumonia, and sepsis guidelines).
• A fair pricing strategy should be in place to avoid catastrophic health expenditure for patients and their families. This strategy need not be oxygen-specific and could include cost-sharing arrangements to avoid extreme costs to the most severely ill patients, debt relief for poor families, and broader insurance scheme arrangements.

In our analysis, we found that oxygen stockouts (ie, unavailability on a given day) occurred at 93% (95% CI 87–97) of primary health-care facilities (ie, level 1 facilities), 45% (43–47) of general hospitals (level 2), and 25% (12–39) of tertiary hospitals (level 3) in LMICs. Poor functionality of oximeters and oxygen sources was common in settings where preventive maintenance was lacking and substantially reduced the availability of oxygen therapy. Technical surveys of oxygen concentrators in hospitals in sub-Saharan Africa and south Asia showed that most concentrators were not functioning, with approximately half that produce gas simply blowing out air.^100^, ^101^, ^102^, ^103^, ^104^, ^105^, ^106^, ^107^ Oxygen cylinders were frequently empty or did not have the required regulatory apparatus for clinical use.^100^, ^101^, ^106^ Oxygen piping was often not used or was used restrictively due to leakages, faults, or incompatibility with bedside terminal connections.^108^ On-site oxygen plants were often working below capacity due to breakdown, power failures, or staffing shortages.^108^, ^109^ Oximeters, when available, were often poor quality, did not have batteries, were faulty or locked away, or did not have appropriately sized probes for neonatal and paediatric care.^100^, ^101^, ^110^, ^111^ Delivery devices were often not available or were inappropriate for the patient population (eg, oximeter probes that were too large for neonates, inappropriately sized nasal cannulae for children, and devices that could not mix oxygen and air for non-invasive ventilation).^100^, ^108^, ^112^, ^113^, ^114^, ^115^, ^116^, ^117^

#### Inequities in pulse oximeter and oxygen availability

*“Prior to COVID, only one public hospital had a functioning oxygen plant in the whole country.”*

*—Doctor, Sierra Leone*

Most data for the readiness of oxygen services came from surveys that focused on pulse oximeter or oxygen equipment availability and that used binary or Likert scores; very few studies provided insight into multiple elements of oxygen service readiness (appendix 1 p 107). These studies therefore systematically overestimated oxygen service readiness but still provide important insight into inequities in pulse oximeter and oxygen availability in LMICs.

**Figure 5:**
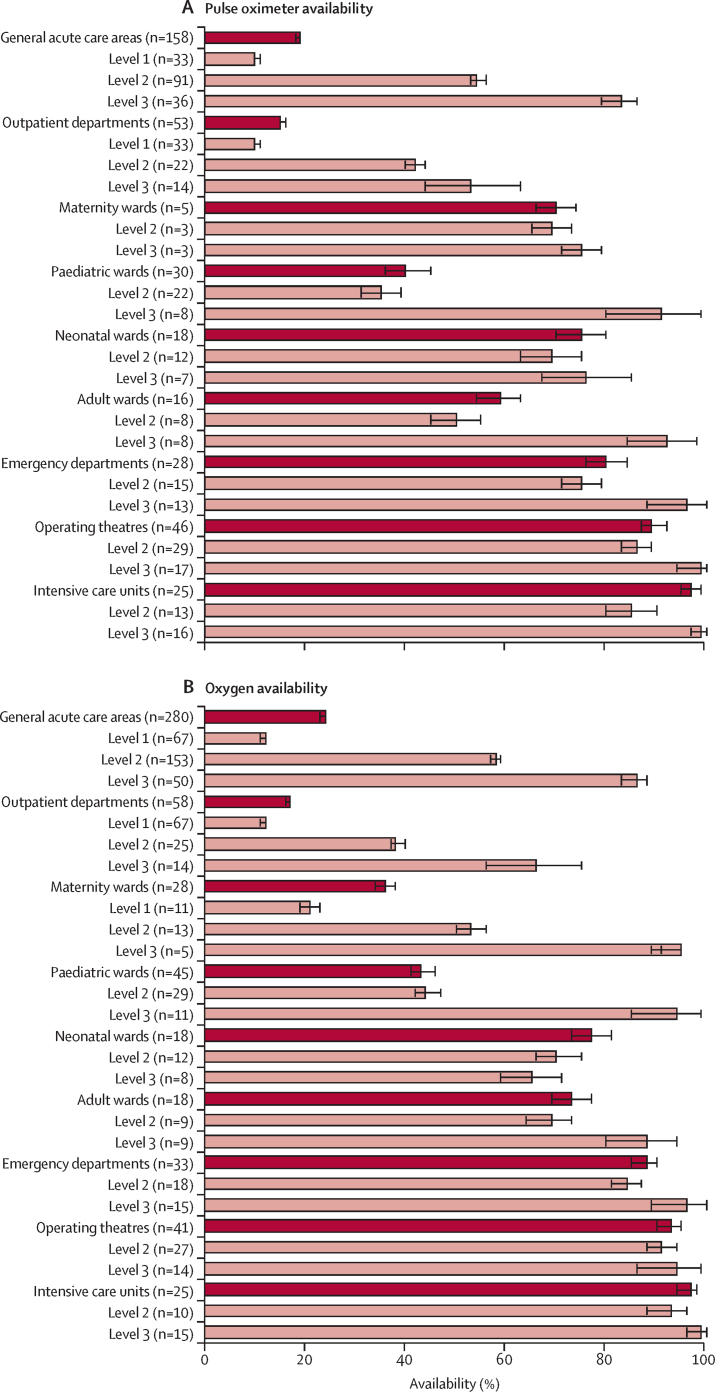
Meta-estimates of oxygen (A) and pulse oximeter (B) availability in health facilities in low-income and middle-income countries, by ward area and facility level Error bars represent 95% CIs. The n in parentheses details the number of datasets included in the meta-estimate. Level 1 describes primary health-care facilities, level 2 general hospitals, and level 3 tertiary hospitals. The darker shading represents overall estimates for a given department or ward. In (A), no data were available for oxygen availability for maternity care in level 1 facilities.

Our analysis of primary health-care facilities in LMICs showed that only 10% (95% CI 10–11) had pulse oximeters and only 12% (11–12) had medical oxygen (figure 5). In general hospitals in LMICs, the pooled prevalence of pulse oximeters was 54% (53–56) and of medical oxygen was 58% (57–59), whereas in tertiary hospitals the corresponding availabilities were 83% (79–86) and 86% (83–88), respectively. The availability of oximeters and oxygen for general acute care in general hospitals was lowest in sub-Saharan Africa (46% and 52%, respectively) and east Asia and the Pacific (70% and 66%, respectively). Availability was higher in south Asia (73% and 81%, respectively) and Latin America and the Caribbean (84% and 100%, respectively), although limited data were identified for these regions (appendix 1 p 116).

**Figure 6:**
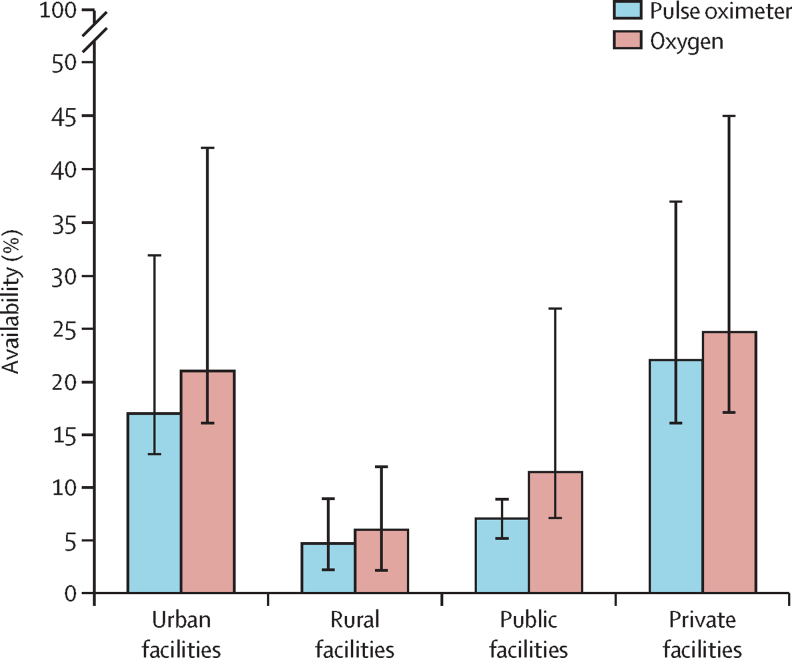
Median proportion of urban and rural health facilities and public and private health facilities in low-income and middle-income countries that have pulse oximeters and oxygen Data are from the Service Availability and Readiness Assessment and the Service Provision Assessment. Both surveys involve large-scale assessment of facility readiness from a representative sample of facilities and are repeated periodically. Data are not disaggregated by level of facility and over-represent level 1 facilities, thus reflecting the lower availability of pulse oximeters and oxygen in smaller facilities.

We found inequities in the availability of pulse oximeters and oxygen between rural and urban contexts, between public and private facilities, and between different wards within facilities. Urban facilities were better equipped with pulse oximeters and oxygen than were rural facilities, with large-scale survey data showing a three-times difference (figure 6). We noted similar differences between private and public government health facilities, with private facilities much more likely to have both pulse oximeters and oxygen. At the ward level, operating theatres and ICUs were more likely to have pulse oximeters (89% and 97%, respectively) and oxygen (93% and 97%, respectively) than were paediatric wards (only 40% had pulse oximeters and only 43% had oxygen; appendix 1 p 116).

We noted negligible improvement in oxygen availability in facilities over the past 25 years, even since the COVID-19 pandemic (appendix 1 p 128). Published reports and consultations with oxygen stakeholders suggested that the lack of improvement post-pandemic was due to multiple factors. Much oxygen-related support did not reach LMICs until very late in the COVID-19 pandemic (ie, from 2022 onwards) and it took a long time for this support to become fully operational.^12^, ^13^ Limited capacity in many LMICs to effectively commission equipment or to set up equipment management and maintenance systems has further delayed progress and raised concern about functionality.^118^, ^119^ Much of the oxygen equipment support went to designated COVID-19 facilities, which were typically larger, urban health facilities, and limited support was delivered to small, rural facilities, further increasing inequities. Encouragingly, pulse oximeter availability has improved since the early 2000s, although quality remains a major problem, especially for infants and children with unique device needs.

### Are patients who need oxygen getting it?

*“There were about 8000 cases of COVID in two and half years, and about 1000 needed oxygen treatment. Many did not get it.”*

*—Doctor, Sierra Leone*

Safe and effective oxygen therapy requires routine use of pulse oximetry to identify people who might need oxygen, timely administration of oxygen therapy via appropriate devices and flow rates, and monitoring to guide continuing treatment. Oxygen-related care is intimately related to broader aspects of care, including triage, resuscitation, documentation, continuity, patient-centredness, and rational use of drugs.

#### Pulse oximetry

*“The whole time in the hospital, we don’t remember our grandmother ever having her oxygen levels measured by a pulse oximeter.”*

*—Daughter of deceased elderly person (cause of death undetermined), Malawi*

**Figure 7:**
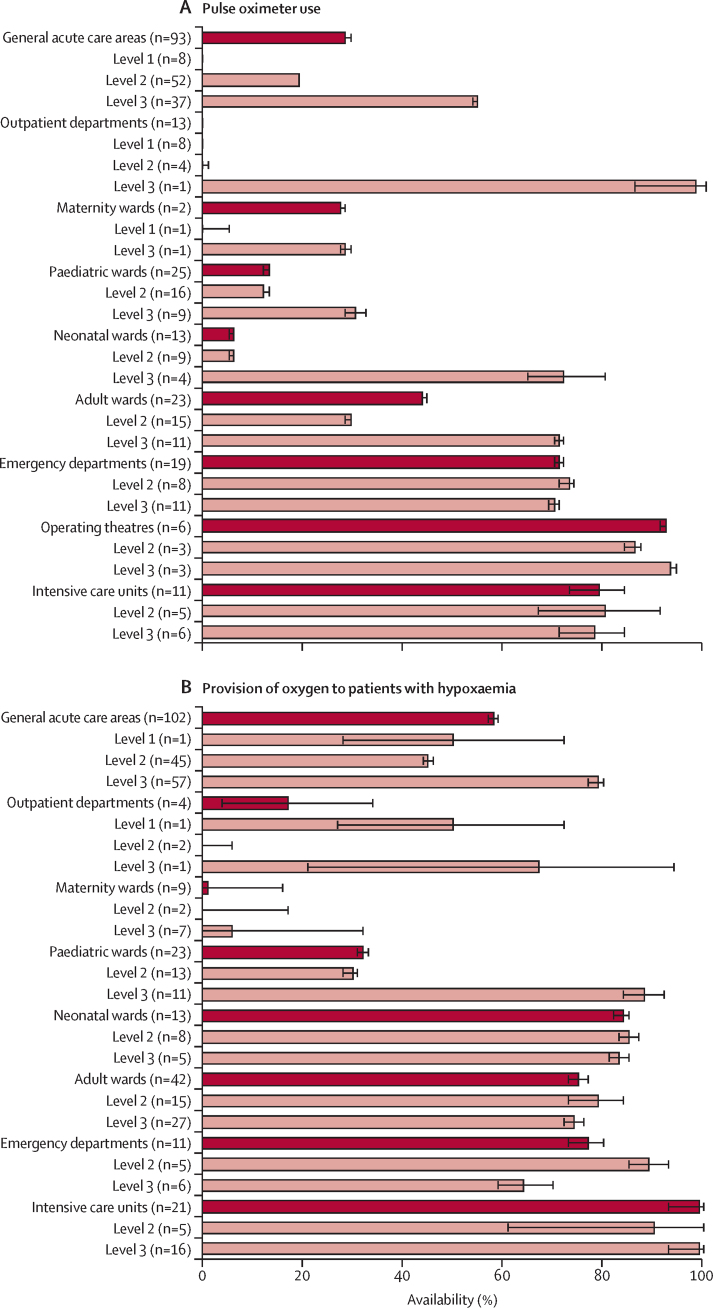
Pulse oximetry use in (A) and oxygen provision to patients with hypoxaemia (B) in health facilities in low-income and middle-income countries, by ward area and facility level Error bars represent 95% CIs. Data for general acute care areas do not include operating theatres and intensive care units. No data were available for oxygen provision to patients with hypoxaemia in operating theatre, for pulse oximetry on level 2 maternity wards, or for oxygen provision for hypoxaemia in level 1 maternity care. The n in parentheses details the number of datasets included in the meta-estimate. Level 1 describes primary health-care facilities, level 2 general hospitals, and level 3 tertiary hospitals. The darker shading represents overall estimates for a given department or ward.

We found that, in LMICs, pulse oximetry was almost never done in patients presenting to primary health-care facilities, and done in only 19% (95% CI 19–19) of patients admitted to general hospitals and 54% (53–54) admitted to tertiary hospitals (figure 7). Pulse oximetry use was low on neonatal (6% [5–6]), paediatric (13% [12–13]), and adult wards (43% [43–44]). Pulse oximetry use in emergency departments (70% [69–71]), ICUs (78% [72–83]), and operating theatres (91% [90–91]) was higher but still not universal. Pulse oximetry use was particularly low in general hospitals in sub-Saharan Africa (14% [13–14]) and south Asia (23% [22–23]).

#### Oxygen provision to patients with hypoxaemia

*“I remember we had 30 minutes of oxygen cylinders left on the ward. So we took those on less than 5 LPM [L per min] off oxygen and those using 5–10 LPM we put on concentrators. We saved the cylinders for the ICU patients. After that we had patients deteriorating rapidly.”*

*—Doctor, Ethiopia*

We found that oxygen was provided to only 45% (95% CI 44–46) of patients with hypoxaemia in general hospitals and only 79% (77–80) of those in tertiary hospitals (figure 7). Importantly, these figures overestimate patient access to oxygen in contexts where pulse oximetry is not routine (because most instances of hypoxaemia will be missed without pulse oximetry). Oxygen provision to patients with hypoxaemia tended to be higher on neonatal (84% [82–85]) and adult wards (75% [73–77]) than on paediatric (32% [31–33]) or maternity (1% [0–16]) wards. Although our analyses focused on facility-based care, some data show similar challenges in pulse oximetry and oxygen provision in medical transport contexts.^120^, ^121^, ^122^

Positive findings from medium-scale to large-scale interventional studies suggest that pulse oximetry and oxygen practices can be effectively improved through education, equipping, and mentorship, which can result in pulse oximetry and oxygen coverage rates of more than 80% in both primary health-care^123^, ^124^, ^125^, ^126^ and hospital settings.^83^, ^127^, ^128^, ^129^, ^130^, ^131^, ^132^, ^133^, ^134^, ^135^ However, our analysis suggests that clinical use of pulse oximetry and oxygen has changed little since the 2000s, despite improved availability of pulse oximetry, highlighting the urgent need to better educate and support health-care workers to use pulse oximetry and oxygen effectively.

Data from studies of the timeliness and appropriateness of oxygen-related care suggest that quality-adjusted oxygen service coverage is much lower than our analysis suggests. As many as a third of patients experience interruptions to oxygen therapy.^127^, ^136^ Data from a study^137^ of 249 hospitalised children who died from pneumonia in seven countries identified major gaps in timely oxygen service delivery: pulse oximetry was documented in only 83 children (33%), and only 127 (51%) had received oxygen therapy before death. Similar low rates of pulse oximetry and oxygen use were noted in death audits of newborns in Ghana,^138^ children in Malawi,^139^ and adult trauma patients in Cameroon.^140^

If oxygen therapy is not guided by pulse oximetry, both underuse and overuse of oxygen frequently occur. As a result, many patients who need oxygen therapy do not receive it and people on oxygen often receive excessive flow rates resulting in limited supplies being wasted.^83^, ^103^, ^114^, ^129^, ^134^, ^141^, ^142^, ^143^, ^144^, ^145^, ^146^, ^147^, ^148^ Oxygen therapy that is not guided by pulse oximetry can potentially lead to adverse hypoxaemic and hyperoxaemic events, particularly in at-risk patients such as preterm neonates. However, saturation targeting to prevent harm can be difficult, especially in contexts with low staff capacity to make frequent adjustments to oxygen therapy even when clear guidelines and continuous monitoring are in place.^146^, ^149^, ^150^, ^151^, ^152^ This issue is particularly challenging in neonatal care in sub-Saharan Africa, where an epidemic of retinopathy of prematurity is predicted to emerge during the 2020s due to unrestricted oxygen use in preterm neonates.^153^

### Patient and community perspectives on oxygen therapy

*“He kept trying to pull off the oxygen mask. He could not tolerate it. It really disturbed him.”*

*—Wife of patient who died from COVID-19, Philippines*

We synthesised information from a scoping review^154^ of patient perceptions of acute medical oxygen and published reviews on long-term oxygen treatment (panel 6), and patient testimonies. From this synthesis, two themes emerged as specific challenges to oxygen access: high out-of-pocket costs, and misconceptions about, and fear and poor understanding of, oxygen therapy. These oxygen-specific barriers interact with many other barriers to care-seeking and health service access on both the demand side (eg, geographical, social, and financial barriers) and supply side (eg, low quality of care and poor treatment by health service staff).^165^

Panel 6 **Patients’ and caregivers’ experiences and perspectives of medical oxygen therapy in academic literature**

Patients’ and caregivers’ experiences and perspectives of oxygen therapy can influence acceptance of, and adherence to, treatment for both acute and long-term oxygen therapy. We mapped academic literature onto the Theoretical Framework of Acceptability, which has seven domains that explore the acceptability of health-care interventions.^154^, ^155^

**Attitude towards oxygen**

Patients and caregivers commonly express fear related to the severe illnesses causing hypoxaemia and sometimes about the potential effects of oxygen use. Extreme fears and refusal of oxygen therapy have been reported due to community misconceptions that medical oxygen kills, sometimes resulting in delays to seeking care in the first place.^156^ However, usually these perceptions and attitudes do not result in treatment refusal and typically improve once the treatment begins to work, for both patients with acute conditions and those with long-term oxygen needs.^157^ People who need long-term oxygen therapy might have additional fears about their oxygen running out and the social stigma associated with the use of oxygen therapy, and might worry about their long-term prognosis.^157^, ^158^, ^159^, ^160^, ^161^, ^162^

**Burden on patients**

Patients and caregivers generally report low levels of perceived burden related to oxygen therapy for acute conditions, but have a clear preference for low-flow oxygen via minimally invasive nasal cannulae over high flow therapy, face masks, or higher levels of respiratory support (eg, continuous positive airway pressure devices or mechanical ventilation).^154^ However, patients requiring long-term therapy reported oxygen as a burden on both themselves and their family members,^157^, ^159^, ^160^ and that long-term oxygen use limited social participation and autonomy.^162^, ^163^, ^164^ In low-income and middle-income countries, oxygen therapy was frequently associated with high out-of-pocket costs,^157^ which could result in reluctance to commence oxygen therapy or premature cessation of treatment and discharge.

**Understanding of oxygen therapy**

Patient and caregiver perspectives of oxygen therapy were directly related to provider attitudes, practices, and education, and greater appreciation of the role of oxygen was associated with better acceptance and adherence. Patient and caregiver education about oxygen therapy was almost universally reported to be inadequate for recipients of short-term and long-term oxygen, and was the most common reason for non-adherence among people on long-term oxygen therapy.^157^, ^159^

**Perceived effectiveness**

The perceived effectiveness of oxygen is important in both acute oxygen therapy (which is generally perceived as life-saving) and long-term oxygen therapy (which is generally perceived as life-enhancing). For people with acute conditions necessitating medical oxygen, seeing evidence that the therapy reduces symptoms is reassuring and probably the most important factor in overcoming fears, worries, and financial concerns. For people with chronic respiratory failure, long-term oxygen therapy is understood as a complicated set of compromises. Patients and caregivers reported improvements in symptoms and functional status (meaning more social activity for some),^157^ and adherence is directly related to the magnitude of perceived symptom relief.^160^ However, treatment was also assocaited with drawbacks in terms of isolation, independence, and self-perception.

#### Cost of oxygen therapy

*“At that time [2021], oxygen cylinders cost about 20 000 taka [$180] each and you had to refill every 2–3 hours. It is almost impossible for patients who are not highly paid to afford medical oxygen.”*

*—Family of a patient with COVID-19, Bangladesh*

Multiple studies have shown the cost of oxygen is a barrier to oxygen access, leading to initial delays in seeking care and fear of being unable to pay, causing patients and caregivers to refuse referrals and treatment.^100^, ^156^, ^166^, ^167^ Cost barriers also came through strongly in patient testimonies provided to the Commission.

#### Fear of oxygen therapy

*“Initially I was scared about the oxygen for my child, but I didn’t say anything about it.”*

*—Mother of a sick child, Bangladesh*

The fear of oxygen therapy that has been reported^156^, ^166^, ^168^, ^169^, ^170^ is typically related to low understanding of, and access to, high-quality oxygen therapy and the high risk of death in people who receive oxygen therapy. In high-mortality settings with little oxygen access, only the very sickest patients will receive oxygen therapy, and a substantial proportion of those people will die. This dynamic can result in a community perception that oxygen is hastening or responsible for patients’ deaths, creating a negative feedback loop of delayed care-seeking, late provision of oxygen, and high mortality. Fear of oxygen can also be related to broader fears of death and loss of hope, with oxygen prescription making patients and their families realise the severity of the illness and the possibility that the patient might not survive.^166^

Importantly, in this context, oxygen-related fears are largely rational and are usually relieved once patients receive oxygen therapy and the beneficial effects are realised.^166^ Health-care workers have an important role to play in alleviating people's fears around oxygen through caring communication and demonstration of increasing oxygen levels in the blood by using pulse oximetry.

## Moving from challenges to solutions

*“We can’t ever be complacent and say it will not happen to us. We need to be prepared and have the systems in place so that lives are not lost unnecessarily for lack of medical oxygen.”*

*—Person with post-COVID-19 condition (also known as long COVID), India*

**Figure 8:**
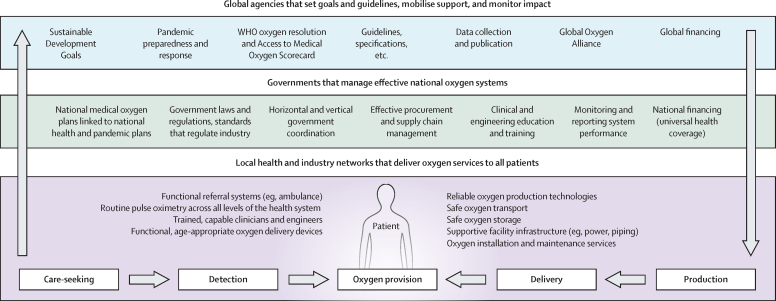
Key features of a resilient national medical oxygen system The arrows depict inter-related efforts and the direction of patient and medical oxygen flows required to provide treatment to a patient in hospital.

In the following sections, we propose solutions to overcome challenges in oxygen service provision and access. These solutions involve five broad thematic areas: use of high-quality, age-appropriate pulse oximetry at all levels of care, so patients in need of oxygen are identified and linked to services; establishing resilient oxygen production, storage, distribution, and delivery systems, which ensure that good-quality, safe, and affordable oxygen is available; coordination of the management of oxygen systems at the national and subnational levels; strengthening of medical oxygen markets, regulations, and standards; and establising robust monitoring and evaluation to track progress in oxygen system strengthening. (figure 8).

Pulse oximeters were invented 50 years ago but pulse oximetry is still not used in many health-care settings, which means that many severely ill patients do not receive the oxygen therapy they need even when oxygen is available. Challenges to pulse oximetry adoption have included the failure to include pulse oximetry in national health policies, medical device lists, and clinical guidelines; poor access to affordable, high-quality, and age-appropriate devices; a paucity of trained clinicians; and workplace cultures that do not recognise the crucial role of pulse oximetry.

Even if a patient arrives at an oxygen-ready facility, there are considerable barriers that can prevent uninterrupted access to medical oxygen, reflecting failures across oxygen planning, regulation, demand, supply, maintenance, and distribution. Each of these steps, from oxygen production to delivery, needs to be supported with biomedical engineering expertise, costed maintenance and repair plans, and, crucially, a plan for how to get oxygen to the point of care. Data about these steps are noticeably absent, which prohibits effective planning by facility managers and district health staff and means that stockouts are common and unpredictable.

Given the need for medical oxygen and pulse oximetry across all levels of the health system and multiple patient groups, oxygen often does not have a clear, specific place among government ministries, and as a result has often been left out of policies, programmes, and data collection systems. At the national level, different parts of the oxygen system often sit with different ministries and public agencies, and oversight of procurement and quality control of oxygen is fragmented and subject to inefficiencies. These issues are partly a result of the absence of coordinating structures that bring all national and subnational actors and regional and international stakeholders together.

These failures in oxygen supply, quality control, and system inefficiencies are linked to how the global oxygen market functions, and deficiencies in regulations, standards, and financing. In countries with mining, steel, and chemical industries, the industrial oxygen sectors have much larger market share than the medical sector.^171^ As a result, the health sector is disempowered as a purchaser of oxygen, particularly when procurement is fragmented and differs considerably from how the process works in other essential medicine markets. Additionally, the liquid oxygen market functions as an oligopoly, with a small number of large, multinational companies controlling most of the medical oxygen market and limiting purchaser power to negotiate fair and transparent prices.^172^, ^173^ Although considerable efforts were made during the COVID-19 pandemic to equip countries with a range of oxygen sources,^16^ much of this equipment is already non-functional or was never used.

To identify tangible solutions and highlight examples of innovations and best practice to tackle these issues, we drew on academic literature from a systematic review of enablers of, and barriers to, oxygen solutions (appendix 1 p 30); stakeholder consultations held with the oxygen industry, ministries of health, patients, caregivers, and regulatory agencies (appendix 1 p 131). We also conducted in-depth case studies on the political economy of oxygen in Malawi, Nigeria, Uganda, Bangladesh, India, Peru, and Sweden (appendix 2). Each case study took a different focus, and countries were selected to highlight both success stories and challenges with oxygen systems globally. Solutions need to consider how technological innovations, workforce capacity, market dynamics, and political systems intersect, with each solution as one piece of a bigger puzzle. Many of the solutions we present are not limited to oxygen systems: oxygen is part of the wider health system, and therefore solutions need to take a broader systems perspective.

## Linking patients to care: pulse oximetry at every health facility

*“Unfortunately, the health facility did not have the necessary equipment to test whether my father's oxygen level was optimal or not. They didn’t have a basic oximeter … and by that time my father was gasping.”*

*—Son of person who died from COVID-19, Kenya*

Pulse oximetry enables non-invasive assessment of blood oxygen level (SpO_2_) at the point of care by a range of health-care workers. Similar to the other core vital signs (ie, temperature, heart rate, respiratory rate, and blood pressure), SpO_2_ is non-invasive, measurable at the point of care by a range of health-care workers, and provides an objective assessment of physiological function. It can therefore assist in making confident diagnoses, assessing risk, and making treatment decisions that ultimately improve clinical care. We summarise key findings about pulse oximetry and how to link patient to effective oxygen care in panel 7.

Panel 7 **Pulse oximetry—key findings**

• Increasing uptake and appropriate use of pulse oximetry and oxygen requires a motivated, sufficiently capable health workforce supported by clear clinical guidelines.
• Oxygen saturation is an essential vital sign and should be used across all levels of health care for acutely unwell newborns, children, adolescents, and adults.
• The high cost of oxygen can deter patients from seeking care, and community misconceptions about oxygen can hinder acceptability.

### Empowering the clinical workforce

*“Right now, even after COVID, half of the clinical workforce doesn’t feel comfortable working with oxygen.”*

*—Doctor, Sierra Leone*

High-quality oxygen services, like high-quality health care more broadly, rely on a capable health workforce using evidence-based clinical guidelines to provide patient-centred care. Therefore, improving oxygen services starts with ensuring that pulse oximetry, oxygen, and SpO_2_ as an essential vital sign^174^, ^175^ are integrated into all relevant clinical guidelines and medical, nursing, and midwifery pre-service curriculums.

#### Clinical guidelines

**Figure 9:**
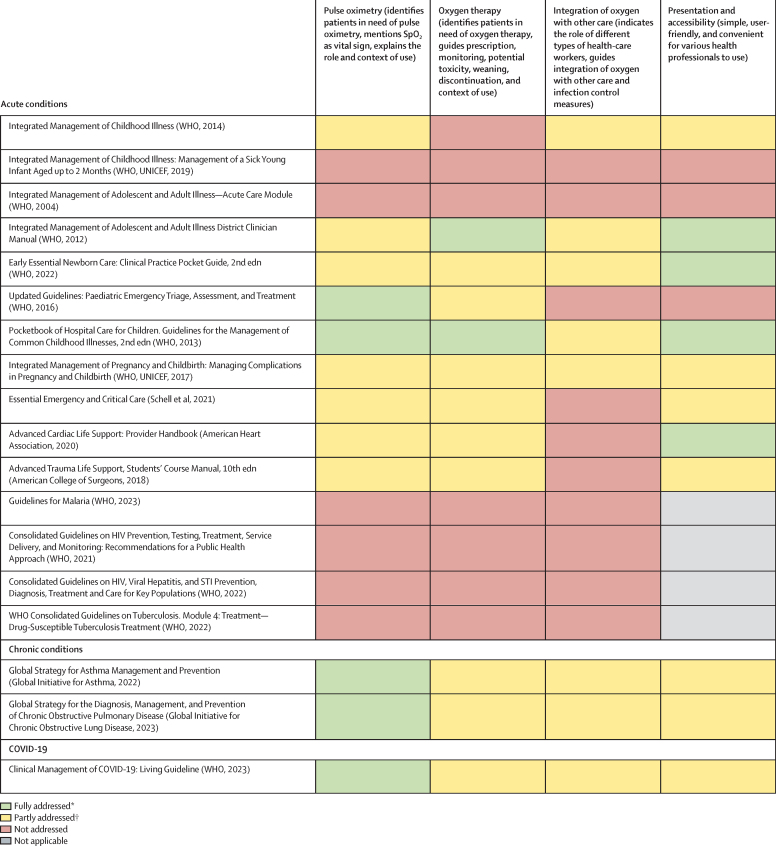
Inclusion of pulse oximetry and oxygen within key clinical guidelines SpO_2_=oxygen concentration in peripheral blood. *The treatment guideline or health-care package has incorporated all the medical oxygen service items assessed in each group. †The treatment guideline or health-care package has incorporated one or more of the medical oxygen service items assessed in each group.

Our review of major health-care packages and clinical guidelines for conditions associated with hypoxaemia (appendix 1 pp 14, 107) found that few adequately addressed pulse oximetry and oxygen therapy (figure 9). Of note, although pulse oximetry and oxygen therapy were substantively included in WHO's core hospital guidelines for children, adolescents, and adults,^31^, ^176^, ^177^ they were almost entirely absent in the corresponding primary health-care guidelines for low-income settings.^32^, ^178^ In terms of disease-specific guidelines, pulse oximetry and oxygen therapy were integrated into guidelines for asthma, COPD, and COVID-19,^179^, ^180^, ^181^ but were largely missing from guidelines for malaria, HIV/AIDS, and tuberculosis. They were also missing from guidelines related to childbirth. Broader inclusion of pulse oximetry and oxygen in clinical guidelines is achievable and should be an urgent priority for improving oxygen access to patients globally.

#### Clinical education

A 2023 review by Peterson and colleagues stated that “pulse oximetry training initiatives have been ongoing for decades for a variety of purposes, utilising a multitude of approaches to equip health care workers with tools to improve patient care”*.*^182^ This quotation highlights the problem with pulse oximetry training but also part of the solution. Given the persistent deficiencies we found in pulse oximetry practices, more training is clearly not enough. Indeed, poor availability of pulse oximeters and unclear messaging in clinical guidelines will continue to limit even the best attempts at training. A more holistic, integrated approach to pulse oximetry implementation is essential.

Many factors influence pulse oximetry use, but they are all broadly related to three key factors that influence behaviour: capability, opportunity, and motivation.^183^ Workforce capability needs to be addressed at pre-service and in-service levels simultaneously to ensure that pulse oximetry and oxygen are represented in curriculums for all major groupings of health workers. Training in delivery of high-level care (eg, non-invasive respiratory support) should be targeted to selected groups (such as intensive care staff). Health managers and clinical leaders need to recognise that high-quality oxygen therapy requires multiple professional groups, and particularly the empowerment of junior or low-level health workers, to recognise and act on the need for oxygen.^184^ Efforts to build capability should be evidence-based. Data for in-service training and continuous professional development suggest that education is most effective when it is practice-based, spaced, and repeated,^185^, ^186^ accompanied by reflection or audit of practice (with feedback to make apparent the gap between current and ideal practice),^187^ and reinforced by opinion leaders, guidelines, job aids, and support from professional associations.^185^ Ongoing supervision and mentorship are crucial.

Health-care workers also need the opportunity to use pulse oximetry in routine care. To do so requires time, reliable oximeters, space, and clinical workflows that encourage pulse oximetry use at the point of triage, in initial assessments, and for monitoring throughout the patient journey.^188^ Adoption of pulse oximetry is also helped by the development of social norms and expectations from peers and clinical leaders,^188^ and by addressing broader determinants of workplace satisfaction, such as economic incentives, job security, and workloads.

Motivation for health workers to consistently use pulse oximetry ultimately comes from health-care workers experiencing the benefits for themselves (eg, their job becomes easier) and their patients (eg, better outcomes).^188^ Motivation for good pulse oximetry and oxygen practices can be facilitated by reducing workloads and improving staffing capacity in overburdened settings,^189^, ^190^, ^191^, ^192^, ^193^ encouraging multidisciplinary cooperation,^194^, ^195^, ^196^, ^197^, ^198^, ^199^ and focusing on simple and trusted devices and therapies.^200^, ^201^, ^202^ However, motivation is lost when health workers are not able to act on a problem that they identify, such as identifying hypoxaemia that requires treatment but then not being able to adequately provide care or to get the patient to a referral destination.

### Pulse oximetry: the key to cost-effective oxygen systems

*“My husband's [pulse oximetry] reading was 73 and so we rushed to the regional hospital. Before this [COVID-19], we did not know what a pulse oximeter was.”*

*—Wife of patient who survived COVID-19, Philippines*

Clear evidence supports the routine use of pulse oximetry for patient assessment and monitoring in hospitals, with improved pulse oximetry practice emerging as the key driver of mortality benefit in oxygen-systems improvement programmes.^75^, ^83^ Pulse oximetry is also key to maximisation of the cost-efficiency of oxygen systems investments: it promotes high-quality oxygen use (ie, in the right patient at the right time in the right dose for the right duration) and sometimes reduces the overall volume of oxygen consumed.^83^ Emerging data for optimal oxygen saturation targets suggest that additional safety and efficiency gains are possible if specifically defined ranges^83^, ^151^—particularly for at-risk populations—are aimed for (panel 2).

Although pulse oximetry's role is clear for acutely unwell patients in hospital, its role in guiding risk assessment and referrals in outpatient and primary health-care settings has not been clear. Primary health-care guidelines provide little information about how to use pulse oximetry or interpret SpO_2_ readings. WHO's 2014 Integrated Management of Childhood Illness (IMCI) guidelines recommended measuring oxygen saturation in children aged 2–59 months with cough or difficulty breathing “if a pulse oximeter is available”, with referral to a higher-level facility if SpO_2_ is less than 90%.^32^, ^178^ WHO's 2024 revision of the pneumonia guidelines includes hypoxaemia as a danger sign for children with pneumonia, and suggests that in the absence of pulse oximetry, signs of respiratory distress should be assessed as an alternative.^203^ However, clinical signs do not effectively identify sick children with hypoxaemia at high risk of mortality.^54^, ^204^, ^205^, ^206^ The WHO revised guidelines did not adopt the guideline review group's advice that pulse oximetry should be available at primary health care; rather, more evidence about the feasibility and implementation challenges was requested. A potential explanation for why hypoxaemia was omitted from the guidelines is debate about whether pulse oximetry is cost-effective in primary health care, and in which populations. Some data suggest that including pulse oximetry in the IMCI guidelines would be highly cost-effective: a modelling study suggested cost-effectiveness as low as $2·97–52·92 per DALY averted,^207^ and in a trial in Ethiopia, pulse oximetry was associated with a cost of $25·72 per severe case of paediatric pneumonia detected.^208^ However, the cost-effectiveness of pulse oximetry against morbidity or mortality outcomes during real-world implementation in primary care has not been shown.

Small-scale and large-scale implementation activities have shown that pulse oximetry is feasible in many primary health-care settings,^209^, ^126^ but effects on clinical care are mixed.^125^, ^126^, ^210^, ^211^ We highlight four priority considerations to guide investment in, and implementation of, pulse oximetry in primary care.^56^ First, oxygen saturation should be recognised as a vital sign and hypoxaemia should be recognised as an important danger sign that should alert health-care workers to severe illness. Second, detection of hypoxaemia (including moderately low SpO_2_ of 90–93% or a failed SpO_2_ reading) should prompt reassessment, consideration of referral or admission, and close follow-up. Third, pulse oximetry should not be considered a standalone technological solution, and its introduction to primary health-care settings should take into account broader service provision capacities, priorities, and contexts. Finally, increased recognition of hypoxaemia at primary health-care facilities is likely to make little difference to outcomes if referral systems and hospital care are not of sufficient quality.

Pulse oximetry access is also the key to cost-effective oxygen services. The findings from the Thanzi la Onse model, which focused on the impact of scaling up oxygen and pulse oximetry use in childhood pneumonia in Malawi (panel 8),^212^ provide three key lessons. First, scaling pulse oximetry to primary health care is cost-effective. Second, pulse oximetry drives the efficiency of oxygen systems. The oxygen need detected and subsequent oxygen service provision doubles when pulse oximetry in outpatient settings covers all facility levels (compared with not using pulse oximetry). Finally, poor quality of care limits the cost-effectiveness of oxygen systems, but improved use of routine pulse oximetry is key to ensuring that people with hypoxaemia receive appropriate care, with oxygen therapy if available.

Panel 8 **The Thanzi la Onse model for scale-up of pulse oximetry and oxygen services for children with pneumonia in Malawi**

Thanzi la Onse,^212^ which means health for all, is a whole-system and all-disease model for Malawi. For this Commission, we used the model to estimate the cost-effectiveness of scaling up oxygen according to the scenarios set out in Malawi's national oxygen roadmap (2021–26),^213^ with added implementation of routine pulse oximetry at different levels of the health system, in terms of preventing paediatric pneumonia deaths.^214^ Additional details of the model are in appendix 1 (p 87).

The implementation of routine pulse oximetry alone at any scale averted DALYs at a cost below Malawi's cost-effectiveness threshold of US$80 per DALY averted, even in sensitivity analyes in which health-system conditions were varied. The scenario that yielded the greatest net health benefit was the one in which both pulse oximetry and oxygen services were implemented at scale (ie, implementation of routine pulse oximetry at all levels of the health system and oxygen scale-up reaching a service availability of 80%), with 42 400 net DALYs averted. This modelled scenario was associated with a 29% reduction in mortality and could have averted more than 73 000 DALYs in children younger than 5 years in 2024, at a cost of $34 per DALY averted and $894 per death averted.

If investments in effective operation of existing oxygen systems achieved an oxygen service availability of 40%, the introduction of routine oximetry at all facility levels could yield greater incremental net health benefit (ie, 28 000 net DALYs averted) than investing in the scale-up of oxygen production and delivery capacity without concurrent implementation of pulse oximetry further into primary care (15 100 net DALYs averted). Our modelling suggests that introduction of routine pulse oximetry across the health system could quintuple the net health benefit of oxygen implementation scenarios.

DALY=disability-adjusted life-year.

Although the Thanzi la Onse model is specific to Malawi, these findings provide valuable insight for other governments. They show that pulse oximetry is crucial, especially when overall implementation of the IMCI guidelines is poor. The model assumed pulse oximetry measurements in 90% of children, reflecting real-world experience from Malawi and other settings.^126^, ^215^ However, in many other settings, this level of adoption has not been achieved.^125^, ^126^, ^210^ Therefore, the critical evidence gap that remains is not whether pulse oximetry should be adopted across all levels of the health system, but how to incorporate pulse oximetry well enough to deliver on potential cost benefits.

Evidence around pulse oximetry is rapidly evolving: a greater number of relevant studies have been published in the past 3 years than in the three decades before. Emerging devices include non-invasive measurement of additional parameters such as respiratory rate and haemoglobin concentrations (so-called multimodal devices). Given the available data, two interventions seem likely to help to improve implementation and impact of pulse oximetry: updated clinical practice guidelines and accompanying education that recognises pulse oximetry as an essential part of routine clinical assessment (ie, as a vital sign), and careful scale-up of pulse oximetry to primary health-care settings. Scale-up should be prioritised in settings where it is most feasible (eg, high-volume facilities with high numbers of respiratory patients) and with the largest potential benefits (eg, remote facilities with some inpatient capacity and oxygen availability, and facilities with under-used referral pathways). We also recognise that improved regulatory standards to promote better and more equitable access to good quality oximeters are needed. We discuss these standards later in the Commission.

### Improving patient attitudes to oxygen therapy through high-quality, affordable care

*“I used to see some people being put off oxygen when they couldn’t pay because their money was all exhausted.”*

*—Person who survived COVID-19, Kenya*

Although oxygen is accepted by patients and caregivers once the benefits start to be felt or seen, initial fears and concerns about costs can delay care-seeking to the point when oxygen will no longer be effective. As access to high-quality oxygen services improves, oxygen therapy is more consistently recognised as beneficial, and community attitudes improve.^156^, ^166^, ^168^ Pulse oximeters are crucial to catalyse this positive shift in attitudes, by supporting effective communication between health-care workers and patients and caregivers, building trust around the need for oxygen therapy, and increasing adherence to referrals and treatment acceptance.^216^

As the benefits of strengthened medical oxygen systems materialise, we expect community narratives to evolve positively, resulting in more demand for these services. However, governments and global health agencies should be proactive in supporting trusted patient advocates and civil society organisations to lead medical oxygen awareness campaigns in communities and patient groups in which misconceptions persist and outcomes are poor. Building community awareness and knowledge could also increase the likelihood that communities will advocate for better services and report poor-quality services, strengthening accountability. We also encourage donors to invest in civil society organisations and advocacy groups so that these groups can become more active in oxygen advocacy. World Oxygen Day, which was launched on Oct 2, 2023, by the COPD Foundation (a patient advocacy group), is an example of a platform on which to build. Professional organisations should also do more to train health-care workers in patient-centred care and communication to dispel misconceptions about oxygen therapy when they talk to patients and their families.

Evidence shows that removal of user fees for health services increases care-seeking, and has a larger impact in low-income households.^217^ Governments should therefore lessen the financial burden on patients and their families by ensuring that medical oxygen services are included in the package of services covered by national UHC schemes and are included in essential packages of health services.^218^ Doing so is especially important for households on low incomes, for whom the cost of medical oxygen treatment can become a catastrophic health expenditure, plunging them further into poverty.

## Building resilient medical oxygen production, storage, distribution, and delivery systems

*“We can’t wait for an emergency to happen. We have to be ready. We must have the systems in place so we don’t lose lives unnecessarily.”*

*—Person with long COVID, India*

The medical oxygen system includes multiple steps from production to patient: generation of medical-grade oxygen, distribution of oxygen to health-care facilities, safe storage and distribution within facilities, and delivery to patients. We summarise key findings for how to build a resilient medical oxygen system in panel 9.

Panel 9 **Building resilient medical oxygen systems—key findings**

• Contextually appropriate models of oxygen production, distribution, and financing should be tailored to local needs and capacities. No one oxygen system will fit all use cases.
• Operational costs make up 50–80% of the total cost of ownership for different oxygen systems. Power is the largest variable factor for pressure swing adsorption, vacuum swing adsorption, and concentrator oxygen systems, whereas distribution logistics are the most important factor for cylinder-based systems.
• Biomedical engineers are a key part of the clinical workforce, and strategic investment is needed to strengthen capacity and promote the role of biomedical engineers during oxygen system planning.
• Reliable, affordable, and sustainable energy sources are needed for oxygen systems, but should be integrated into facility-wide electrification initiatives.

### Oxygen systems: not one-size-fits-all

*“We used the concentrators as bridges until the next oxygen cylinders came.”*

*—Doctor, Ethiopia*

Multiple technologies are available to enable each step of the medical oxygen system, from production to patient delivery, resulting in wide variation in what an oxygen system can look like. This variability is both an opportunity, in that it enables adaptation of systems to what best suits local needs, but also a challenge in terms of coordination and maximising efficiency.

Different technologies produce different concentrations of medical oxygen. Cryogenic air separation units produce liquid oxygen that has a concentration of greater than 99%. Pressure swing adsorption (PSA) and vacuum swing adsorption (VSA) plants—which use compressors or vacuum pumps, respectively, to pass air through an adsorbent material (such as zeolite) to remove nitrogen, allowing oxygen to be collected—produce oxygen concentrations of 93% from ambient air.^20^, ^28^ Similarly, oxygen concentrators are portable, self-contained, electrically powered oxygen sources that use PSA technology to concentrate oxygen from ambient air.^219^ The medical oxygen produced by both methods is safe and clinically equivalent, and is almost always combined with ambient or compressed air before reaching patients’ lungs.^20^ The choice of oxygen source should be guided by careful consideration of health facility size, use requirements, regulatory structures, workforce capacity, and cost and energy requirements (table 3).

**Table 3:** Sources of medical oxygen and typical uses

	**Description**	**Benefits**	**Challenges**	**Typical use case**
Bulk liquid oxygen	High-pressure liquid oxygen (99% purity)	Minimal power requirement at health-care facilitiesHigh production capacityEfficient to transport and store (ie, more oxygen in less volume)Typically cheaper per Nm^3^Can serve piped system via vacuum insulated evaporatorCan fill oxygen cylinders	High up-front capital costsProduction requires highly skilled techniciansRequires specialised storage and transport infrastructure and safety precautionsRequires expertise to install and maintain pipingRisk of leakage	Produced offsite at dedicated facilities for both industrial and medical industries and then delivered to tertiary or secondary hospitals, where oxygen is piped to the patient's bedside. Alternatively, liquid oxygen can be stored in cylinders and distributed to peripheral facilities (via a hub-and-spoke model).
Pressure or vacuum swing adsorption oxygen plant	Medium-pressure oxygen gas that can be compressed for cylinder filling (90–95% purity)	Continuous renewable supply of oxygenCan directly serve piped systemCan fill cylinders for bedside delivery via compressor	High up-front capital costsUninterrupted power supply is needed (with extra power required for filling cylinders)Requires expertise to install and maintain pipingRisk of leakage	Produced at a tertiary or secondary hospital and piped to the patient's bedside. Alternatively, the oxygen could be produced centrally, stored in cylinders, and distributed to peripheral facilities (via a hub-and-spoke model).
Oxygen concentrator	Low-pressure oxygen gas (90–95% purity)	Low up-front capital costsContinuous renewable supply of oxygen Portable	Not suitable for all patients (eg, patients who require very high flow rates or higher-pressure oxygen)Uninterrupted power supply is neededLow-volume outputFrequent preventive maintenance required	Home care or primary health-care facilities and small, remote secondary facilities.
Oxygen cylinder	Very-high-pressure oxygen gas (purity depends on source)	No power requirement at point of use (but high power needed to fill) Few maintenance requirements Portable	Limited exhaustible supply Frequent refills required High distribution costs Safety risks Cumbersome to move	Can be used as a back-up oxygen source that can be transported within and between facilities. Alternatively, can be a primary oxygen source for small or medium facilities at the bedside or as part of a manifold system.

Adapted from Smith et al (2020) and Asian Development Bank (2022).^220^, ^221^ Nm^3^=normal cubic metres.

For bulk liquid oxygen, the major barriers to entry are high up-front capital costs and technical requirements (table 3). As a result, liquid oxygen is only really a viable option at large secondary and tertiary health facilities with high patient volumes and biomedical engineering capacity. When implemented at an appropriate scale, liquid oxygen can be cost-effective for health systems—so long as it is subject to fair market conditions. The liquid oxygen market has been underdeveloped in many LMICs. The financial viability of production depends on guaranteeing a minimum level of demand, and whether health care alone is sufficient to sustain a domestic industry is unclear.

PSA and VSA plants are designed to produce and distribute oxygen directly within facilities, either through a central piped distribution system or by refilling portable cylinders for direct use at the bedside or via connection to a cylinder manifold (which then feeds into a piped distribution system). This dual capability allows for flexibility in delivery of medical oxygen to where it is needed. PSA and VSA plants are increasingly used in LMICs, given the appeal of offering a continuous supply of oxygen without the logistical challenges associated with procuring medical oxygen. The major challenges with these plants are running costs, substantial maintenance needs, and the need for an uninterrupted power supply; the responsibility for addressing these issues has generally fallen on facility managers.

Oxygen concentrators have the flexibility to serve one or more patients and are a low-maintenance, low-cost solution for settings with low-to-moderate oxygen demand. They can serve most, but not all, patient needs (there are flow rate and outlet pressure limitations) but can have a short lifespan if not properly cleaned or maintained, or if exposed to environmental pollution, humidity, or high temperatures. Most oxygen concentrator products on the market have been developed for home-care use for patients in high-income settings. It is therefore important for users to be aware of, and adhere to, the manufacturer's instructions for use to promote device longevity. Given the original use case for oxygen concentrators, their suitability in high-volume, low-resource health-care settings has been questioned.^222^ In response, a multi-stakeholder consultation led by UNICEF developed a target product profile for a more robust oxygen concentrator,^223^ which prompted improvements to available products and the release of a new concentrator meeting all specifications in 2024.

We have chosen not to recommend one specific oxygen source as the best for health systems. Instead, we conclude that there is no one-size-fits-all solution that will work in every health system. Rather, the two key lessons that have emerged are that mixed sources of oxygen should be embraced, and that back-up oxygen sources are essential. Panel 10 provides an example of a hospital in Uganda that has effectively incorporated multiple sources for medical oxygen to increase resilience against supply interruptions, challenges with maintenance and repair, power outages, and occupational hazards.

Panel 10 **Mixed oxygen sources in practice—the example of a regional hospital in Uganda**

In Uganda, use of mixed sources of medical oxygen is the adopted supply strategy. Oxygen concentrators and PSA plants with oxygen cylinders are the main storage and distribution devices, and use of liquid oxygen is limited. An estimated 75% of medical oxygen comes from cylinders that are refilled at PSA plants installed at regional referral hospitals or at central medical stores and procured from the steel manufacturing industry.^224^ The distribution of filled oxygen cylinders is based on a mix of hub-and-spoke (ie, regional distribution hubs supplying to facilities within their catchment area) and milkman (ie, central medical stores transporting and exchanging filled cylinders for empty cylinders) supply models.

Mbale Regional Referral Hospital in eastern Uganda serves a population of almost 5 million people across 17 districts. It provides an example of how these different sources of medical oxygen are used in a tertiary referral facility. The hospital has 470 inpatient beds, with intensive care, surgical, maternity, psychiatric, paediatric, neonatal, and internal medicine inpatient units. It has 16 medical specialists (ie, medical doctors with master's-level training or fellowship training in the specified medical specialisations and clinical work experience)—only 11% of the number it should have according to Ugandan Ministry of Health norms—164 nurses, 14 medical officers, eight anaesthetic officers, two pharmacists, and six pharmacy dispensers. Thus, Mbale Regional Referral Hospital is a busy regional referral hospital with an insufficient workforce that serves a large patient need with a range of oxygen requirements.

The hospital has 24 oxygen concentrators spread across ward areas and uses, on average, 200 cylinders every 2 weeks (approximately 4·3 Nm^3^). These cylinders are primarily intended to be refilled on site by a PSA plant, which has capacity to meet this need (when functional, the plant can produce enough oxygen to fill 25 cylinders per day). However, the PSA plant is often not functional, and instead the cylinders are refilled by a private steel company 250 km away, with the hospital responsible for driving them there and back. For distribution within the facility, the hospital has installed multiple 2 x 5 cylinder manifold systems (comprising two connected banks of five oxygen cylinders—one bank providing active supply, the other functioning as a back-up—that ensure a continuous oxygen supply by combining flow from all the cylinders in a bank into one pipeline, which is then distributed to patient-use areas or medical equipment) for acute inpatient wards, including the paediatric acute care, neonatal, medical, maternity, emergency, and surgical high-dependency (which also serves as a temporary intensive care unit) wards. As a secondary supply, the paediatric acute care unit has three oxygen concentrators, and the neonatal unit has seven oxygen concentrators that are in constant use. The use of manifold systems in several ward areas is an efficient solution compared with moving cylinders to individual patient beds, but ward areas that are not included in this system are served by portable 50 L (9 Nm^3^) oxygen cylinders that are moved from one bedside to another.

The hospital faces various challenges: the prohibitive cost of the journey to refill cylinders when the on-site PSA plant in not functioning; a low overall number of cylinders (as a result of wear and tear), which prevents buffer stocking; a shortage of the oxygen cylinder distributors needed to move cylinders to where they are needed; frequent electricity outages that interrupt oxygen concentrator supply; and insufficient biomedical engineers to service and maintain the oxygen equipment (including concentrators). Despite these issues, harnessing mixed oxygen sources has meant that the hospital has been largely able to ensure an uninterrupted supply of medical oxygen to patients in need.

PSA=pressure swing adsorption. Nm^3^=normal cubic metres.

Another crucial component in planning for a resilient oxygen system is the ability to rapidly increase supply in times of surge need. At a minimum, oxygen systems should be designed with an in-built buffer (ie, mass storage and increased production capacity) that can cope with normal seasonal fluctuations. In terms of preparation for larger surges, the COVID-19 pandemic proved the usefulness of large liquid oxygen storage capacity, prenegotiated agreements with industrial gas suppliers, and deployment of oxygen concentrators to decentralise care for low-risk patients.^225^, ^226^ For disaster and conflict zones where infrastructure is damaged, oxygen supplies can be completely interrupted at facilities and for home-care patients,^227^ and simultaneously casualties, trauma, and disease outbreaks can increase the need for medical oxygen. Managing patient flow between facilities where oxygen is readily available and accessible to immediate relief efforts is crucial.^228^ Oxygen concentrators coupled with a power solution, and on-site portable oxygen generators can be rapidly and flexibly deployed. Devices specifically for humanitarian emergency use cases (eg, Médecins Sans Frontières’ oxygen concentrator with in-built solar power) have been developed,^229^ but their application in a real-world humanitarian response has yet to be assessed.

### Planning for the total cost of ownership

*“Right now, most of our hospitals are cemeteries for broken medical equipment.”*

*—Doctor, Sierra Leone*

**Figure 10:**
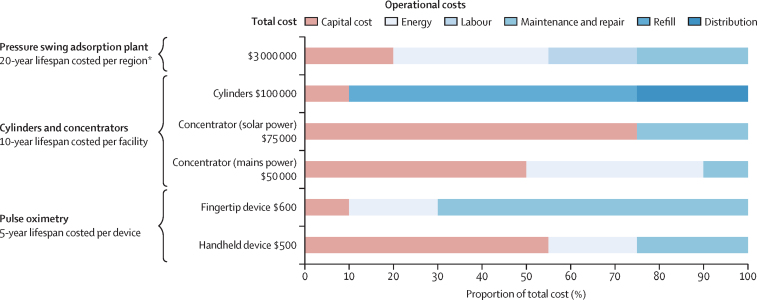
Capital and operation costs of different oxygen system components Costs are an estimated total cost of ownership. The breakdown of cost categories (in US$) is based on data from publications^83^, ^230^, ^231^, ^232^ and Open Oximetry related to projects in six countries (Nigeria, Papua New Guinea, The Gambia, Kenya, Rwanda, and Ethiopia), and is intended to support budget planning. The balance between categories will vary by setting, and this figure should not be used as a cost-comparison tool. *Based on regional hub-and-spoke models.

When choosing which oxygen system will be most suitable, understanding the total cost of ownership is key. Figure 10 provides guidance on planning for the total cost of ownership for different oxygen systems. For most systems, the operational costs exceed the capital costs over their lifetime. The main factor affecting the balance between capital and operational costs for oxygen concentrators and PSA plants is the cost of energy, which is typically incurred as regular payments for electricity or fuel to run generators (with the exception of locally generated solar power, which has high capital costs and lower relative operational costs).

Over the 20-year lifespan of a PSA plant, the operational costs, which can be more than $100 000 a year, make up 80% of the total cost of ownership.^230^ For cylinders, distribution costs are most important and are particularly relevant for rural and hard-to-reach facilities, for which distribution can often cost more than the oxygen itself.^84^, ^230^, ^233^ For pulse oximeters, procurement of the lowest-cost devices can be enticing, but masks potentially higher lifetime costs if devices malfunction and break easily, requiring regular replacement (as exemplified by Open Oximetry, a platform that openly publishes the results of independent pulse oximeter quality testing, purchase cost, and estimated total costs of ownership). There are also concerns for patient safety if cheap devices are of poor quality. Donors and national governments should consider the total cost of ownership when procuring equipment to ensure that there is a plan in place to effectively cover operational costs.

### Affordable, uninterrupted, and clean power

*“When the power went off, patients on the concentrators had to wait for the generator to kick in. Sometimes it took five minutes, and we had patients who died in that gap of time.”*

*—Doctor, Sierra Leone*

Experiences from various settings where uninterrupted power for medical oxygen has been established reveals four key lessons. First, governments and supporting agencies need to understand the local energy environment, in recognition of the crucial role of electricity in oxygen systems. Energy is one of the biggest costs related to oxygen production and distribution (figure 10), and the cost of the energy needed to generate oxygen influences whether oxygen concentrators, PSA and VSA plants, or liquid oxygen will be the most cost-effective technology.^84^, ^233^, ^234^ For example, a study in India showed that the cost of oxygen generated from PSA plants increased by a factor of 7·5 when the power supply shifted from 24-h mains electricity to a diesel generator.^234^

Second, all medical equipment intended for use in settings with unreliable electricity supplies should be equipped with surge and voltage fluctuation and variation protection. Brownouts (ie, a temporary drop in voltage) and voltage fluctuations damage equipment, resulting in shorter lifespans and therefore higher health-system costs. Indeed, WHO estimated that more than 50% of medical equipment in LMICs was not functional, and that nearly a third of medical device failures globally were caused by unreliable electricity supply.^235^ Many devices that provide power surge and voltage protection are available, and they should be included with any procurement of equipment that requires electricity. An early example of such an approach comes from the 42-bed Medical Research Council Hospital near Banjul in The Gambia, where dedicated sockets for oxygen concentrators that protected from voltage fluctuations were installed and benefits in terms of equipment longevity were apparent.^233^ Newer technologies should include in-built protections against poor power quality, such as the FREO2 PROTECT, a power-conditioning system that stabilises energy supply against voltage fluctuations.^236^

Third, solar electricity is a cost-effective, clean, sustainable, and flexible solution that is resilient to the challenges of a changing climate. Solar-powered oxygen concentrator systems at facilities in Nigeria, Papua New Guinea, Uganda, and Somalia have improved oxygen access and quality of care, and have been associated with reduced mortality.^77^, ^83^, ^237^, ^238^ Several initiatives have been launched to establish solar-powered PSA plants.^239^, ^240^, ^241^ Although the average price of solar panel modules declined by nearly 90% between 2010 and 2020,^242^ and battery costs have also decreased to a lesser extent, the up-front cost is still high. Rather than exclusively powering oxygen, solarisation solutions should consider the needs of entire facilities to find efficiencies and help to solve broader difficulties in the electrification of health.

Finally, new technologies should prioritise energy efficiency and robustness to harsh conditions—a point relevant for all settings as climate disasters and temperature extremes become more common globally. A key feature of UNICEF's target product profile for a resilient oxygen concentrator, published in 2022, was the requirement for solar-power compatibility and protections from voltage fluctuations and poor-quality power supply.^223^ Beyond simply meeting certification standards, new technologies need to be field-tested to establish functionality, usability, and durability in real-world harsh conditions.

Access to electricity is essential to the delivery of health services. However, nearly 1 billion people in low-income and lower-middle-income countries are estimated to be served by health-care facilities without reliable electricity access or with no electricity access at all.^243^ The requirement for reliable power is not unique to medical oxygen, but oxygen systems need a lot of energy, and present an opportunity to advocate more broadly for sustainable electricity in health systems.

### Biomedical engineers: essential health-care workers

*“The number of available biomedical engineers was not adequate to handle the oxygen needs and maintenance of the electrical equipment.”*

*—Doctor, Ethiopia*

A neglected aspect of sustaining national medical oxygen systems is the biomedical engineering workforce and the tools that this workforce needs to install, operate, and maintain equipment.^244^ During the COVID-19 pandemic, many LMICs did not have enough biomedical engineers or the required collaborations between engineers, health-care workers, and health-care managers in place to respond effectively to the surge in demand for medical oxygen. Biomedical engineers often lacked basic tools or access to spare parts or the requisite technical documentation to do their job. The biomedical engineering workforce in LMICs is overwhelmingly located in urban areas and tertiary facilities. These issues with biomedical engineering workforce capacity increase the risk that the oxygen equipment donated to LMICs during the COVID-19 pandemic could contribute to so-called equipment graveyards.^245^ Despite efforts to strengthen biomedical engineering capacity by global health agencies, donors, and professional organisations (eg, the International Federation of Medical and Biological Engineering and the Global Clinical Engineering Alliance), wide gaps remain.

The most recent WHO data for the number of biomedical engineers (defined broadly to include all health engineers and technicians) are from 2017 and cover only 139 countries. According to these data, the global average is 0·33 biomedical engineers per 10 000 population, ranging from 0·05 per 10 000 population in low-income countries to 0·60 per 10 000 population in high-income countries.^246^ For comparison, WHO estimates that globally in 2022 there were an average of 17·2 doctors, and 37·7 nurses and midwives per 10 000 population.^247^ WHO has recommended a minimum of 44·5 doctors, nurses, and midwives per 10 000 people as the minimum health workforce density needed to achieve UHC and the SDGs.^248^ There is no equivalent benchmark for biomedical engineers, and there is only one reference to biomedical engineers in the report Global Strategy on Human Resources for Health: Workforce 2030.^249^

We propose that a minimum threshold of 0·4 biomedical engineers (or equivalent) per 10 000 population is needed by 2030 for an effective health system (appendix 1 p 102), and posit that WHO should validate, adopt, and track this recommendation. This threshold equates to around one biomedical engineer for every 100 hospital beds, but we appreciate that as health system capacity develops to handle more complex diagnostics and procedures, the density and skills of biomedical staff will need to evolve. For oxygen, the biomedical engineering needs at a given facility will also depend on the oxygen supply solutions. For example, there are typically higher biomedical engineering needs at facilities that operate PSA or VSA plants or have extensive gas piping than at facilities that rely on delivered cylinders or a small number of oxygen concentrators, where maintenance and repairs are generally simple.^231^ Therefore, biomedical engineers need to be included in the design of national oxygen systems and work with educational establishments to ensure that curriculums match the needs of the oxygen system.

A specific target for biomedical engineering density will enable measurement of the WHO Oxygen Resolution requirement that member states “provide for adequate numbers of engineers to establish demand, select, set up, operate and maintain the equipment and all the infrastructure related to medical oxygen production, storage and uninterrupted distribution to patients”.^15^ Member states should report their progress using the dedicated domain within the Access to Medical Oxygen Scorecard (ATMO_2_S; appendix 1 pp 90–98), a tool we developed for national governments to track progress towards achieving the 2023 WHO Oxygen Resolution (discussed later in the Commission), and WHO should ensure that these data are reported alongside the ratios for other key health workers in the Global Health Workforce statistics database.^247^

Although increasing the absolute numbers of biomedical engineers is important, it is equally important to ensure that engineers are equitably distributed and able to service rural areas and small facilities. The largest gaps in biomedical engineering workforce capacity are in sub-Saharan Africa, where there are concrete opportunities to make rapid progress. One of the five pillars of the New Public Health Order led by the Africa Centres for Disease Control and Prevention is to build “the capacities and capabilities of all workers who contribute to public health in Africa”,^250^ and biomedical engineers have been highlighted as a crucial part of that workforce.^251^ Efforts can leverage existing initiatives, including the African Biomedical Engineering Consortium, the UBORA initiative, the African Biomedical Engineering Mobility programme, and efforts to build Rwanda's flagship East African Regional Center of Excellence in Biomedical Engineering and eHealth. Investing in these initiatives is a way in which global health agencies and donors can increase focus and funding to the operational costs of sustaining national medical oxygen equipment.

The new African Women in Biomedical Engineering Alliance is an opportunity to tackle the under-representation of women, who represent only 23% of the biomedical engineering workforce globally.^250^ We support calls for global action to strengthen the capacity of the biomedical engineering workforce,^252^ and encourage all medical oxygen stakeholders to work together to strengthen the profession, and particularly its role in national medical oxygen systems.

### Supporting innovations from production to patients

*“We prayed that this one concentrator that we all bandaged up by plaster—we basically Macgyver-ed it—would keep two patients alive. Human ingenuity during challenging times is amazing, and both patients lasted the whole weekend.”*

*—Doctor, Ethiopia*

In addition to ensuring the functionality of medical oxygen equipment, biomedical engineers working in these systems are also a source of innovation. There are few national programmes to support local medical oxygen innovators. Most international global oxygen support is granted to large, international non-governmental organisations. However, many of the solutions that stood out to the Commission were locally innovated and delivered, and had produced promising data in terms of cost-savings for health systems and potential benefits for patients.

In terms of repair and maintenance, OpenO2—a Malawian organisation—established a mobile unit that travelled from hospital to hospital refurbishing broken oxygen concentrators. During the pandemic, OpenO2 technicians repaired 649 mobile concentrators across 58 hospitals, enabling the use of 658 000 Nm^3^ of oxygen in the Malawian health system. They also manufactured an oxygen analyser that costs $40 (a fifth of the price of commercial products) and for which open-source specifications have been published, and they have also pioneered a method for reviving the zeolite in mobile oxygen concentrators.^253^ OpenO2 estimates that its local innovations have saved $4 million for the Malawian health system.

With regard to production and distribution, Hewatele, a Kenyan social enterprise, was a pioneer in the use of PSA technology to increase access to medical oxygen in Africa, in collaboration with several other partners.^230^ Hewatele's hub-and-spoke model uses centralised PSA plants to fill cylinders for several satellite health facilities. The organisation takes responsibility for both the operation and maintenance of the PSA plant, the logistics for distribution and refilling of cylinders, and training. Hewatele reports that it has reduced the cost of a 50 L oxygen cylinder by 39% for hospitals in the Siaya Region of Kenya compared with the prices charged by previous oxygen providers.^230^

These organisations are just two examples of the many promising oxygen access innovations we identified that originated in LMICs (table 4). We recommend that national governments, global health agencies, and donors collaborate to assess the effects of innovations in oxygen products and services on oxygen access in low-income settings and subsequently increase investment in high-impact models with the greatest likelihood of sustaining access cost-effectively over time. We echo calls for greater localisation in how global health agencies and donors provide support, as well as calls for greater investment in innovations and organisations that emerge from low-income settings.^255^, ^256^

**Table 4:** Priority areas for medical oxygen-related innovation

	**Examples**
**Pulse oximetry and oxygen use**	
Improve accuracy of pulse oximeters	Open Oximetry: a free online platform that reports pulse oximetry performance based on independent studies
Improve clinical and biomedical oxygen-related training	The Oxygen Series: an extensive series of free, online training videos and resources in multiple languages for clinicians in LMICs from Stanford Medicine, Assist International, and Lifebox
Develop better, more affordable oxygen delivery devices	Polite CPAP: low-cost neonatal CPAP device designed and built in Nigeria to replace the commonly used improvised CPAP devices^254^*
Strengthen professional associations	African Women in Biomedical Engineering Alliance: the first professional association for women working as biomedical engineers and technicians across Africa, with the aim of strengthening skills, networks, and opportunities for leadership, and closing the wide gender gaps in the profession*
**Oxygen supply systems**	
Develop more robust oxygen concentrators	PulmO2: a 10 L per min oxygen concentrator designed to the specifications of UNICEFs target product profile
Reduce graveyards of broken equipment	OpenO2: an organisation of mobile biomedical engineers who repair broken oxygen concentrators and related devices for a fraction of the cost of purchasing new equipment*
Improve oxygen service management models	Airbank: a social business delivering oxygen directly to hospitals in Nigeria and Kenya as part of the Oxygen Hub (which provides entrepreneurs in Kenya, Ethiopia, and Nigeria with financing, equipment leasing, and management support)*
Develop more affordable methods of oxygen generation	Medical ceramic oxygen generator: a new technology for generating medical oxygen in harsh operating environments based on ceramic ion transport membrane technology
Improve access to spare parts	Centralised procurement mechanism for oxygen compressor spare parts: a mechanism that provides fast access to affordable spare parts for oxygen plants designed by PATH and partners
Introduce power-outage-proof oxygen technologies	FREO2 low-pressure oxygen system: this system includes a reserve that holds excess oxygen from a concentrator; if the power cuts out, this oxygen is automatically released, providing a supply that lasts 8–10 h
Reduce energy costs of oxygen plants	Africa Infrastructure Relief and Support: an initiative providing installation and maintenance of solar-powered oxygen plants and biomedical engineering training at three sites in west Africa
**Coordination**	
Strengthen national government leadership	National medical oxygen plans: government plans outlining how a country will ensure access to pulse oximetry and medical oxygen*
Improve oxygen data generation and management	India's national medical oxygen grid: an online platform for hospitals to manage medical oxygen supplies and for governments to minimise stockouts at local, regional, and national levels*
Raise awareness about oxygen as an essential medicine	World Oxygen Day: a global effort to rally the world to advocate for access to medical oxygen held annually on Oct 2
Connect public and private oxygen sectors	Oxygen Alliance: a collaboration of public and private sector stakeholders for the repair and maintenance of biomedical devices to ensure the delivery of high-quality health care*
Better coordinate management of national oxygen systems	Oxygen desks, Nigeria: dedicated officers, based in federal and state ministries of health, who coordinate medical oxygen activities horizontally across national stakeholders and vertically with subnational stakeholders*
Better coordinate global oxygen support to LMICs	Global Oxygen Alliance: an alliance of 20 global health agencies and donors providing oxygen support to LMICs
**Oxygen markets and regulation**	
Reduce anti-competition practices in the oxygen industry	*WHO Pharmacopoeia*: This standard defines both 99% and 93% oxygen as safe for medical use and enables the mixing of oxygen from both sources, reducing the risk that health facilities will be locked in to one supplier
Increase manufacturing and supply chain management in LMICs	Hewatele's east Africa liquid oxygen plant: the first fully African-owned liquid oxygen facility with finance from donor governments, development finance institutions, and philanthropists*
Increase corporate oxygen access responsibility	Aire Liquide Access Oxygen: a corporate programme that involves company oxygen access targets, regular reporting, and flagship programmes in several LMICs to increase access to medical oxygen

The use of brand names or any mention of specific commercial products or services is solely for educational purposes and does not imply endorsement by the *Lancet Global Health* Commission on medical oxygen security. LMICs=low-income and middle-income countries. CPAP=continuous positive airway pressure.

* LMIC innovation.

In high-income countries, it is rare for governments to own or operate medical oxygen production or distribution systems. However, in LMICs it is common for health facilities to bear responsibility for maintaining oxygen supplies, with the piecemeal involvement of the private sector. To address this costly burden on health facilities, various groups have proposed alternative solutions that involve outsourcing oxygen supply under a range of different business models (eg, for profit, not-for-profit, and hybrid models). The oxygen-as-a-utility model proposes to produce, deliver, and bill oxygen services as a public utility (like water or electricity).^171^ Other models emphasise that oxygen supply is a service, rather than a commodity, and promote outsourcing of oxygen supply to private providers for a fixed price.^257^ This approach has been refined to describe an outsourced-oxygen-to-bedside model, characterised by “oxygen supply services provided by external entities that provide a minimum set of oxygen supply equipment, repair, maintenance, and training, and are contracted to guarantee continuous medical oxygen supply to the bedside”.^258^

These market-based models could improve oxygen access and are attractive to many governments and funders seeking to better utilise the private sector for oxygen supply. However, data on their efficacy, affordability, and financial sustainability are scarce, and public sector procurement systems that can contract them are needed. For not-for-profit models, securing donor support in perpetuity is unlikely, and the for-profit models are dependent on governments or individual facilities being willing and able to contract their services at the price needed for the business to be workable. For both, securing any necessary spare parts, retaining trained employees, and competing with much larger multinational companies are all barriers to viability.^258^, ^259^ We are also concerned that short-term subsidised models could crowd out the growth of local businesses, which cannot compete with non-governmental organisations that provide oxygen services free of charge to health facilities. Investment in local medical oxygen businesses with a mixture of grants, subsidies, loans, and equity, in partnership with development finance institutions, could be a more sustainable solution.

## Coordination of national medical oxygen systems

*“Investing in oxygen is one thing; ensuring access is another.”*

*—Person with long COVID, India*

For a functional national oxygen system, coordination and planning is needed across a wide and complex set of actors, with multiple government actors who must interface with the private sector and international stakeholders - particularly if they are reliant on external donor health financing. In panel 11, we highlight our key findings about coordination of national oxygen systems.

Panel 11 **Coordination of national medical oxygen systems—key findings**

• As of 2024, 27 low-income and middle-income countries have national oxygen plans; medical oxygen is largely absent from global emergency and pandemic preparedness architecture
• A national plan or strategy for medical oxygen is a powerful tool beyond planning and can be leveraged to improve coordination, advocacy, and fundraising
• Coordination structures need to work horizontally to connect stakeholder groups, and should ensure that there is a clear point of contact for oxygen systems within ministries of health at national and subnational levels, especially in countries where the management of the health system is devolved to lower levels of government
• Domestic and global support for oxygen systems has not been adequately driven by locally identified needs, priorities, and action plans, resulting in inappropriate investments but also opportunities for improving impact

### National oxygen plans as tools for planning, financing, and advocacy

*“We shouldn’t ever have to go through what we did with COVID. We didn’t have the things that we needed in place and at that time it was oxygen that was needed.”*

*—Person who survived COVID-19, India*

National oxygen plans are strategic national policy documents that describe the existing oxygen ecosystem, quantify gaps in current and future needs, and outline strategies to strengthen oxygen system capacity and performance. As of 2024, 27 LMICs have published a national oxygen plan. Most countries that have published national oxygen plans are in sub-Saharan Africa.^260^ Ethiopia was the first LMIC to publish a national medical oxygen and pulse oximetry scale-up roadmap, which was followed by Nigeria's national strategy for the scale-up of medical oxygen in health facilities (both countries are now onto the second iteration of their national oxygen plans).^261^, ^262^ These plans became essential tools when the COVID-19 pandemic hit, providing a degree of structure for the national COVID-19 response and helping to manage the sudden increase in donor requests. However, our Nigeria case study (appendix 2) highlighted the need for a dedicated budget to launch national oxygen plans and for advocacy to promote awareness among key stakeholders to ensure maximum impact.

Despite a rapid increase in the number of countries launching or developing national oxygen plans, only 14% of member states have plans. The 2023 WHO Oxygen Resolution urged all governments to “develop, as appropriate, costed national plans to increase access to quality assured, affordable medical oxygen systems and personnel to meet the identified needs of all patients in the context of national achievement of the health-related Sustainable Development Goals and universal health coverage”.^15^ In May, 2024, WHO convened a meeting, with 63 ministries of health in attendance, to co-create a national oxygen plan template to support national governments yet to roll out such a plan. A scoping review of existing plans provided the basis for developing a draft template, which participants then voted to modify. The initial review found that monitoring and research for impact was the weakest domain in existing plans, in line with previously highlighted research gaps in oxygen implementation.^263^ In the voting, participants opted to add an assessment of the environmental impact of oxygen systems, echoing themes throughout the Commission on the importance of energy and embracing green technologies. Governments should use this tool alongside ATMO_2_S to first identify their strengths and weaknesses and then draft a national oxygen plan that is responsive to their needs.

### Integration of oxygen into emergency preparedness and response efforts

*“We shouldn’t ever have to go through what we did with COVID. We didn’t have the things that we needed in place and at that time it was oxygen that was needed.”*

*—Person with long COVID, India*

Since the COVID-19 pandemic, substantial efforts have been underway to strengthen national and global architecture to better prepare for the next emergency that will require large quantities of medical oxygen. The world faces more humanitarian crises than ever before: climate change is increasing the frequency and severity of natural disasters, there are more active armed conflicts in process now than at any other time since World War 2, and more than 363 million people are displaced or in need of humanitarian assistance.^264^ Future respiratory epidemics and pandemics are highly likely, with some estimates suggesting a 66% chance of a COVID-like pandemic causing more than 10 million deaths in the next 25 years.^265^ These estimates predict that Africa and Asia would experience the highest mortality, with more than half of all deaths in sub-Saharan Africa (23%), India (18%), and China (14%) alone.^265^

To prepare, all governments should include medical oxygen and respiratory care in their national pandemic preparedness and response policies and operational frameworks, and conversely should include emergency preparedness and response in their medical oxygen plans. These plans should include estimates of both the quantity and cost of the medical oxygen that might be needed and outline measures to secure additional supply quickly. Governments need to ensure that all relevant contracts with industry include measures to secure surge capacity during emergencies, including diverting oxygen from industry customers to hospitals (eg, via force majeure provisions, which excuse contractual obligations when certain events beyond the control of the parties take place).

Global health agencies should provide support to governments, when needed, to develop and implement national oxygen emergency plans and to ensure that all relevant pandemic strategies and guidelines include medical oxygen. The inclusion of medical oxygen is especially important for WHO initiatives, including national action plans for health security, the Health Emergency Preparedness and Response framework, the state parties annual report, the Joint External Evaluation framework, benchmarks for international health regulations, and the Preparedness and Response to Emerging Threats initiative. Major pandemic preparedness financing channels, including the Pandemic Fund and The Global Fund, should keep investing in medical oxygen systems to strengthen pandemic preparedness and response across vulnerable LMICs, and to increase health system resilience for the future.

### Coordination of oxygen planning and programmes

*“Many, many people lost their lives for lack of oxygen and that is a national and global shame.”*

*—Person with long COVID, India*

**Figure 11:**
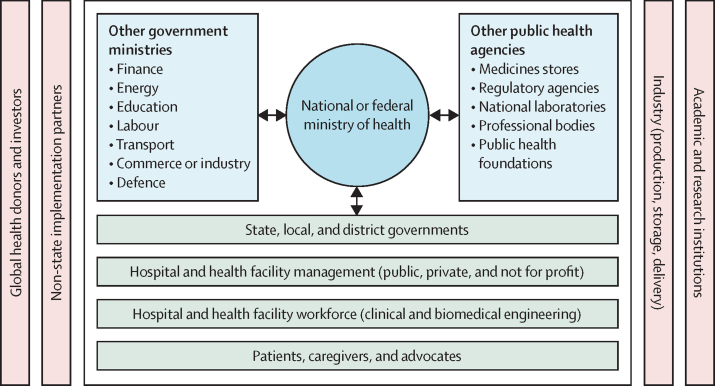
Key stakeholders in a national medical oxygen system Adapted from Mirza et al (2023).^266^

At the national level, key government ministries involved in the provision of medical oxygen include health, finance, industry, transportation, education, and energy (figure 11). National medicine stores, pharmaceutical and medical device regulatory agencies, higher education institutions, and health professional bodies also have roles (figure 11). National oxygen systems can be more complicated in countries with multiple levels of government, especially those with federal systems. In countries where the private or non-profit sectors are major providers of health care, different rules and regulations can apply to different health-care providers, adding yet more complexity. For example, in Sweden, hospital-level care is organised by region, whereas elderly care falls under the remit of municipalities, but municipalities are not permitted to employ doctors and therefore cannot deliver oxygen treatment (see Sweden case study; appendix 2). In Uganda, there are separate procurement systems for public and private health facilities, meaning that there are two systems for oversight of quality control (see Uganda case study; (appendix 2). In Peru, three different government ministries act as providers of medical care, alongside private providers, resulting in a fragmented market.^267^ These examples highlight different types of decentralised provision of oxygen that have resulted in inefficiencies and barriers to access.

The role of centralised but networked medical oxygen decision making has gained some traction. The example of oxygen desks, an approach developed in Nigeria, is worth highlighting as an example of how to assign responsibility for oversight of the oxygen system without establishing a vertical programme. In Nigeria, federal, state, and local governments are responsible for different aspects of the health system, with the federal ministry of health ultimately responsible for medical oxygen. Several external global health actors also support medical oxygen delivery (to different extents in different states). An oxygen desk was established in the federal ministry of health and tasked with coordinating the implementation of the Nigerian national oxygen strategy. At the same time, oxygen desks were established in each state to support the adaptation and adoption of the strategy at state level, in effect creating a national network of government decision makers. State oxygen desks are responsible for contextualising the national oxygen strategy and coordinating actions across stakeholders in their states, and report back to the federal ministry of health (see Nigeria case study; appendix 2).

National governments should consider transforming multisectoral oxygen taskforces that were assembled during the COVID-19 pandemic into sustained coordination structures with clear mandates to improve equitable access to medical oxygen. These efforts should also include mapping stakeholders and defining their roles and responsibilities. For countries without such taskforces, synergies and integration within existing programmes should be exploited, along with a focus on horizontal coordination mechanisms such as the oxygen desk approach.

### Aligning financing to need

*“We have to work on our oxygen reserves. We should never have to face this kind of shortage again.”*

*—Doctor, Ethiopia*

National governments are the primary party responsible for ensuring oxygen access for their populations, and should include oxygen in the package of services covered by national UHC schemes.^218^ In recognition of this obligation, the WHO Oxygen Resolution called on governments to develop fully costed national oxygen plans. However, many governments are struggling to prioritise and finance oxygen services.

We have identified four key lessons to ensure that oxygen financing is matched to need. First, government spending on oxygen services should be guided by national needs estimates and a comprehensive budget. As much as is possible, governments should source funds to fill oxygen coverage gaps by reallocating existing revenue, achieving efficiencies in current spending, or by increasing revenue (eg, through introducing so-called pro-health taxes on alcohol, tobacco, or sugary foods and beverages). Crucially, governments should not shift oxygen costs onto patients, which would exacerbate the already high out-of-pocket costs that have been reported for oxygen.^100^, ^154^, ^156^, ^166^, ^167^

Second, LMIC governments that need external support to close medical oxygen coverage gaps should coordinate external financing support to ensure that investments are tailored to the local context to minimise duplication and maximise efficiencies across programmes. Financing support could include funding to procure equipment but also to sustain the supply of medical oxygen, with budget lines for clinical, engineering, and administrative personnel and the operational costs of production, distribution, maintenance, and repair. Although several new funders stepped into the oxygen funding space during the COVID-19 pandemic (eg, The Global Fund and the World Bank), many governments found these funds challenging to access, with most of the funding going to countries that had high-level support from international non-governmental organisations. As the oxygen financing landscape continues to evolve, governments should be able to look towards GO_2_AL, which includes major global health agencies and funders, for assistance in accessing and coordinating external financing support.

Third, donors share responsibility to ensure that oxygen financing is directed towards priority needs and that their investments maximise impact and promote sustainability. During the COVID-19 pandemic, there were unprecedented investments in oxygen equipment by global health donors: The Global Fund alone provided $617 million to support 98 countries and six regional projects in increasing access to oxygen in 2020 and 2021.^268^ Between April, 2020, and November, 2023, WHO tracked oxygen investments in 141 countries worth $410·7 million by a subset of global health agencies (table 5). The largest spend, $114 million, was on ventilators—a decision that was questioned at the time and, with hindsight, was ill judged.^269^ $76·0 million was spent on PSA or VSA plants and components (equating to 247 plants for 55 countries), $81·4 million on concentrators, and $38·5 million on pulse oximeters. Less than 1% of the $410·7 million was spent on training, operational support, and power solutions combined, and a similarly low amount went towards spare parts. Consequently, many countries that received support, especially in sub-Saharan Africa, were left with an urgent need to finance set-up and recurrent operational costs. Unfortunately, one of the most common stories to emerge from the pandemic was of newly procured PSA plants never being turned on, breaking quickly, or being turned off because there was no plan for how to finance operations (panel 12).

**Table 5:** COVID-19 oxygen need, mortality, and international funding, by World Bank region, 2021

	**Proportion of global coverage gap for acute and surgical oxygen need (%)**	**Proportion of global COVID-19 oxygen need (%)**	Proportion of global COVID-19-related deaths (%)*	**Value of oxygen aid (millions of US$), 2021–23**†	**Proportion of total global oxygen aid (%)**
East Asia and Pacific	26%	9%	9%	59·4	14%
Europe and central Asia	4%	16%	22%	48·3	12%
Latin America and the Caribbean	7%	11%	17%	16·8	4%
Middle East and north Africa	3%	7%	3%	46·8	11%
North America	0	4%	7%	<0·1	0
South Asia	37%	29%	29%	41·9	10%
Sub-Saharan Africa	23%	24%	14%	197·6	48%

Because of rounding, percentages do not always sum to 100%.

* Global Burden of Disease data.

† WHO COVID-19 supply chain dashboard data (unpublished).

Panel 12 **The risks associated with establishing PSA plants in low-resource settings**

At the start of the COVID-19 pandemic, the race to increase oxygen production capacity was warranted, and a focus on procuring and installing PSA plants was logical: PSA plants are quicker and cheaper to install than air separation units or vacuum swing adsorption pressure plants, the cost of oxygen cylinders had increased substantially, and concentrators could not provide the flow-rate of oxygen needed for some patients with COVID-19. However, the promise of PSA plants has fallen short for many reasons, including the lack of appropriate infrastructure at facilities for them to be installed, insufficient biomedical engineering capacity to install, run, and maintain the plants, high energy requirements and a lack of public policies around energy pricing, making plants too expensive to run, and confusion surrounding ownership and responsibility for maintenance.

These issues were partly the result of geopolitics, with several global factors colliding to exacerbate running costs and disrupt supply chains. Many of the PSA plants were supplied after the initial peaks in severe cases of COVID-19 in unvaccinated people, as global energy prices rose (tripling between 2020 and 2022) partly as a result of the interruption of oil and gas supplies caused by Russia's war on Ukraine. Therefore, a lowering of oxygen demand as cases slowed coincided with a sharp increase in the cost to produce oxygen. The result was that many PSAs in low-income and middle-income countries were being turned off—or were never turned on. However, the emphasis on PSA plants was also a failure of planning. Despite the good intentions that motivated investment, there was a clear lack of consideration and coordination of who would be responsible for running, maintaining, and repairing the PSA plants.

In India, state governments and other stakeholders expressed concerns about the high costs of running PSA plants, describing them as white elephants, while acknowledging that allowing them to lie idle could lead to their becoming defunct—a waste of the estimated US$730 million invested in them (India case study; appendix 2). At Loayza Hospital in Peru, three oxygen plants were donated, including one by the Southern Mining company. However, because the machines were not formally owned by the Peruvian Government, government funds could not be invested in maintaining them.^267^ In Uganda, individual facilities became responsible for the running of PSA plants without the relevant managerial or medical expertise or dedicated budgets (see Uganda case study; appendix 2). In some cases, the operating costs of PSAs were more than facilities’ annual budgets.

Rather than solving oxygen supply issues, the provision of PSA plants has left many facilities with burdensome assets that they cannot effectively use. Establishment of models for the sustainable production of oxygen through already installed PSAs, such as external service contracts, is urgent. For PSAs that have not yet been installed, countries should seriously consider whether they will be a boon or burden.

PSA=pressure swing adsorption.

The issues introduced by donations or misguided procurement were not isolated to PSA or VSA plants, or to the COVID-19 pandemic response. For example, a national equipment survey in Malawi from 2022 showed that 50 unique concentrator and 49 pulse oximeter brands were in use.^270^ The consequences of this variety are dangerous and inefficient.^271^ Governments need to establish systems of oversight for medical equipment procurement and donations, and prospectively track these areas for future planning. However, global health agencies and donors need to take more responsibility for ensuring that they follow the principles set out by WHO for medical device donations.^272^ For oxygen, donors should consider whether the facilities to which they donate equipment have the technical capacity to run, maintain, repair, and use the technology, as well as the financial capacity to cover operational costs for the lifetime of use. Funders often work with short timelines that do not cover the 20-year lifespan of a PSA or VSA plant, and therefore up-front planning for long-term support, handover, and ownership is essential.

Finally, during pandemics and emergencies, investments should be balanced between the systems with the largest pre-existing health system gaps and those with the largest burden associated with the acute event. Analysis of how COVID-19 funds were allocated showed that international oxygen support was not well aligned with either priority (table 5). The region with the highest gap in volume in terms of acute oxygen need, COVID-19-related oxygen need, and COVID-19 mortality was south Asia. However, it received just 10% of funding—a lower proportion than only North America and Latin America and the Caribbean. In turn, Latin America and the Caribbean received only 4% of funding, despite having some of the highest mortality rates and the widest relative gap between COVID-19 oxygen need and mortality.^1^ The sub-Saharan Africa region received nearly half the funding, with 25% going to just Ethiopia, Nigeria, and Uganda—countries with well established national oxygen plans and pre-pandemic oxygen programmes. Because COVID-19 treatment centres were most often established in tertiary hospitals, even though coverage gaps were most prominent in small rural hospitals, this funding is unlikely to have had the effect it should have had on closing subnational gaps in oxygen access. Financing during emergencies should still be explicitly linked to national oxygen plans and pandemic preparedness and response efforts, but nuanced consideration of where the greatest need exists is crucial.

## Strengthening medical oxygen regulations and markets

*“Our hospital didn’t have a good supply of empty cylinders for the private companies to refill so we had to rent the cylinders plus pay to refill them and then pay to transport them to the hospital.”*

*—Doctor, Sierra Leone*

The medical oxygen industry has an essential role to play in increasing access to medical oxygen, and they should be recognised as part of the public health infrastructure. However, governments need foster fair market conditions and regulatory bodies ensure fit for purpose medical equipment. Our key findings for strenghtening medical oxygen markets are in panel 13.

Panel 13 **Strengthening medical oxygen regulations and markets—key findings**

• The medical oxygen industry, like the pharmaceutical industry, is an essential part of public health and pandemic preparedness and response architecture.
• There is little competition in the liquid oxygen sector, leading to high prices, particularly in LMICs, where monopolies, duopolies, and oligopolies are the norm. Legal frameworks that regulate markets, support new market entrants, and open tenders with price transparency are essential to increase competition.
• Government definitions of medical oxygen in national pharmacopoeias and other regulations are not aligned to the updated *WHO Pharmacopoeia*—which includes oxygen 93%—which has led to confusion around definitions and restricted oxygen sources.
• Regulations and standards fall short of ensuring that all pulse oximeters work for all patients, but there are opportunities to improve international regulations, which are undergoing revisions.
• Representation of LMICs on International Organization for Standardization committees is unacceptably low. Only six of the 31 members of the Anaesthetic and Respiratory Equipment Technical Committee are from LMICs.
• Partnerships between governments, global health actors, and industry can improve access to medical oxygen and are an opportunity for industry to expand their market. However, these partnerships need to be independently assessed to ensure that they are benefitting patients in a sustainable, cost-effective way.

LMICs=low-income and middle-income countries.

### Increasing competition

*“We have very few companies that make oxygen and most hospitals do not have the capacity to manufacture their own, so we have to rely on company monopolies, and this created the situation that we found ourselves in.”*

*—Son of person who died from COVID-19, Kenya*

Governments need to establish legal frameworks that prevent a single buyer or seller from affecting the market and that support buyers to choose from a selection of high-quality products to which there is no barrier to entry. When these conditions are not met, markets can become distorted, resulting in high prices, poor-quality products, and few players—characteristics that markets for medical oxygen have too often exhibited. The lack of competition in liquid oxygen markets specifically, for which national monopolies, duopolies, and oligopolies are the norm, has contributed to low access to medical oxygen in many LMICs. Three multinational companies headquartered in Europe and the USA dominate the global market for medical oxygen, and there is a well documented history of anti-competition practices and market manipulation, with multiple legal cases related to price-fixing, hindering of competitive tendering processes, and restrictive negotiation across multiple global regions.^273^

Industrial clients dominate the market for liquid oxygen. The health sector is a small, low-value sector in terms of oxygen in most countries. Although industrial demand for oxygen increases supply more broadly, it also limits health sector leverage and leaves the sector vulnerable to predatory practices, particularly when national and subnational procurement is fragmented. Governments can improve competition in their national oxygen markets by ensuring that national competition laws and regulations encourage a level playing field and fair trade practices, and by ensuring that independent arbitration (eg, trade commissions) is available for all parties to pursue grievances and appropriate penalties to discourage anti-competition practices. More specfically for medical oxygen, we present two solutions: open tenders and supporting new market entrants.

Governments and global health agencies can increase competition by conducting open, competitive tenders for procurement of oxygen equipment and contract services, and by publishing contractual terms and prices. Such efforts are particularly uncommon for liquid oxygen. UNICEF's oxygen market dashboard publishes prices for pulse oximeters, oxygen concentrators, and PSA or VSA plants, but it does not include liquid oxygen pricing.^274^ The lack of information about liquid oxygen means that governments and global health agencies cannot assess the most cost-effective mix of oxygen solutions and places health facilities at a disadvantage when negotiating contracts with the oxygen industry. In addition to governments publishing oxygen contract prices, global health agencies should collate samples of prices from different LMICs and regularly publish a medical oxygen market report, similar to the HIV market report produced by the Clinton Health Access Initiative.^275^

To address liquid oxygen oligopolies, new companies need to be supported to enter the market. Several strategies are available to help new companies to establish a foothold, and multiple efforts are underway to change the liquid oxygen market in sub-Saharan Africa. The first approach is to establish more domestic medical oxygen companies. If more production facilities are based in LMICs, health services are likely to benefit from reduced distribution costs, safety risks, and wait times compared with relying on international imports. A promising development is the first fully African-owned liquid oxygen plant, which is being developed in Kenya: this initiative was financed in 2023 with a $20 million loan and grants from development finance institutions and philanthropy organisations, with plans to serve the east African health sector exclusively, and reduce the cost of liquid oxygen in the region by 30%.^276^ There are questions about whether liquid oxygen production is financially viable without catering to industrial markets, so assessment of the sustainability of this initiative will be important.

Another noteworthy initiative is the Zambian Government's building of a cylinder filling station that liquid oxygen companies can use to fill medical oxygen cylinders.^277^ This approach enables small companies that do not have their own filling facilities to compete and requires all companies to use the same cylinder connectors, reducing lock-ins that tie health-care providers to specific manufacturers and oxygen providers. Finally, governments can increase competition by ensuring that health-care facilities own their oxygen piping and liquid oxygen storage infrastructure (which are often owned by private companies), which would allow them to use medical oxygen from several different suppliers and sources, including PSA or VSA plants.

### Updating national pharmacopoeias to include 93% oxygen

*“After a year or so we had an oxygen plant in the hospital compound, so the bottleneck that we used to face regarding oxygen was solved.”*

*—Doctor, Ethiopia*

Governments can improve competition between liquid oxygen and PSA or VSA plant companies by ensuring that their national definition of medical oxygen includes both cryogenically distilled liquid oxygen (ie, 99·5% oxygen) and PSA-generated or VSA-generated oxygen (ie, 93% oxygen), and that national guidelines state that both concentrations of oxygen can be safely mixed and delivered to the patient. Such a definition of medical oxygen reflects the 11th edition of WHO's *International Pharmacopoeia*, which was updated in 2022^20^ to address the problem of health facilities being restricted to one source of oxygen and dependent on one company. We are concerned that many national governments have not yet realigned their national pharmacopoeias with the new definition, and the belief that only 99·5% oxygen is safe for medical use remains pervasive. A case study from Peru shows an extreme consequence of this misconception (panel 14). We urge governments, industry, and global agencies to align their policies and operations with the new *International Pharmacopoeia* and ensure that they are universally understood by both the health sector (ie, clinicians, administrators, and civil servants) and industry in LMICs.

Panel 14 **The liquid oxygen policy trap in Peru**

In January, 2003, EsSalud—the social security system of Peru—claimed that two liquid oxygen companies had entered into agreements to restrict competition and fix prices during public tenders from 1999 to 2001. This complaint gave rise to the initiation of a sanctioning procedure by the National Institute for the Defence of Free Competition and the Protection of Intellectual Property. In 2010, the procedure was declared valid, and appealed by the liquid oxygen companies. In 2013, the Court for the Defence of Competition and Intellectual Property concluded that the two companies had incurred a very serious infringement—ie, restricting competitive market allocation during the national public procurement of medical oxygen from 1999 to 2004. The companies were fined the equivalent of almost US$7 million, but it was not until 2020 that the Peruvian Supreme Court ratified the sanction imposed—17 years after the complaint was raised.^278^ As of 2024, the fine has still not been paid.

This market dynamic, in which a duopoly thrived, was reinforced by a 2010 law that defined medical oxygen as having to contain oxygen in concentrations of greater than 99%, which created a policy trap in which the medical oxygen market was restricted to liquid oxygen. Liquid oxygen production is associated with high up-front capital investment costs, and in a context with two already dominant actors, the barrier to entry for new companies was unreasonably high. This policy trap not only enabled, but promoted, sub-optimal market dynamics.

When the COVID-19 pandemic reached Peru, the country had insufficient and inequitably distributed oxygen supplies. Multiple laws, policies, and regulations were enacted to undo this policy trap. In June, 2020, an emergency decree exceptionally authorised the use of 93% medical oxygen and established extraordinary measures to increase production and access to medical oxygen. However, it eventually took two laws, three ministerial resolutions, and 7 months to officially redefine medical oxygen to include 93% oxygen.

Peru had the highest COVID-19 mortality rate in 2020,^1^ partly as a result of several underlying and interlinked health system challenges and political instability (there were eight ministers of health in 2020 and 2021). However, the delays in diversifying and scaling up access to oxygen, which were rooted in economic and political interests of a liquid oxygen duopoly, were undoubtedly a key driver of this tragedy.Adapted from Garcia et al (2025).^267^

The International Organization for Standardization (ISO) is an independent, non-governmental organisation composed of representatives from the national standards organisations of member countries. It publishes more than 25 000 international standards, covering almost all aspects of technology and manufacturing. The Anaesthetic and Respiratory Equipment Technical Committee presides over medical oxygen supply and respiratory care devices and related matters. This group could play an essential role by recommending the harmonisation of national pharmacopoeias.

### Improved product regulations and standards

*“In the NICU [neonatal ICU] there are many ventilators we never got to use, oxygen concentrators that are dysfunctional… and the pulse oximeter probes don’t fit the newborns.”*

*—Neonatal ICU nurse, Ethiopia*

It is essential that regulations and standards governing oxygen-related products result in safe, high-quality products that work effectively for all patients across age groups and different medical oxygen systems. Ensuring that oxygen products are safe and effective is also important for manufacturers and for governments and global health agencies procuring these products to avoid legal liabilities and penalties that could arise from non-adherence.^20^, ^279^, ^280^, ^281^, ^282^ We highlight three specific issues on which further action is needed: low-quality pulse oximeters that do not work accurately when used in patients with darker skin pigmentation; the existence of multiple standards for oxygen cylinder connections, which inhibits interoperability; and under-representation of LMICs on global standards bodies, which led to standards that are not sensitive to LMIC contexts.

Pulse oximeter performance varies by manufacturer and model. Performance can be influenced by several patient factors, including age (with infants and children posing specific challenges in terms of movement artifact and probe-fit) and skin pigmentation. Concerns have been increasingly raised about the durability and accuracy of low-cost oximeters and the accuracy of pulse oximeters in patients with darker skin. Several studies have shown that low-cost oximeters, including some that claim to be in compliance with regulations, do not all meet performance standards for clinical use.^283^, ^284^ From the industry perspective, the prioritisation of low prices in government procurements acts as a barrier to investment in products designed specifically for LMIC markets. Governments, meanwhile, are often restricted by limited budgets and the need to ensure value for money (see Bangladesh case study; appendix 2). Given governments’ desire to purchase cheap equipment, there is a need for clear guidance on product performance in real-world settings to support public sector procurement of high-quality devices. Open Oximetry provides a solution for this information gap. Governments and global health agencies procuring pulse oximeters should use this resource (or similar initiatives) to ensure that quality, performance criteria, and lifetime rather than purchase cost are incorporated into procurement regulations.

Irrespective of price, several oximeters have been linked to potentially life-threatening inaccuracies when used in people with dark skin pigmentation.^285^, ^286^ Of the nearly 50 published studies that have examined the effectiveness of pulse oximetry in people with dark skin pigmentation, most show that pulse oximeters tend to overestimate actual SaO_2_ in this group, which means that people who might require oxygen therapy are not identified.^287^, ^288^ However, there are substantial limitations to these data.

**Figure 12:**
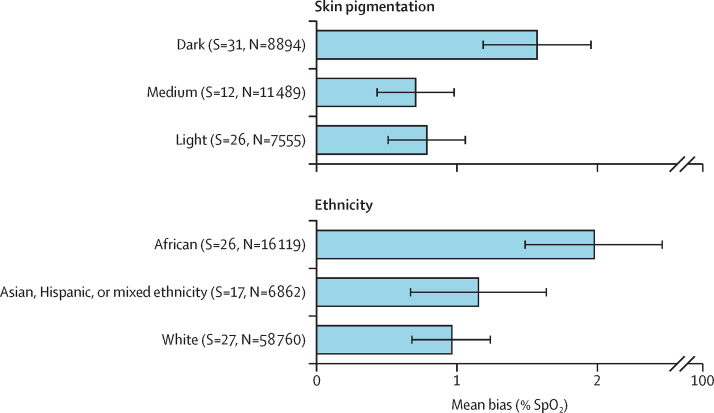
Mean pulse oximetry bias by skin pigmentation and ethnicity S indicates the number of studies that provided data for these estimates. Error bars show 95% CIs. SpO_2_=oxygen concentration in peripheral blood.

We did an updated review focused on the accuracy of SpO_2_ measurement with reflectance or transmissive pulse oximeters compared with SaO_2_. Our review included 33 studies, of which 30 were done in high-income countries (appendix 1 pp 21–29). We found that dark skin pigmentation was associated with a 1·58% overestimation of SpO_2_ (positive bias), whereas medium pigmentation was associated with a 0·71% overestimation and light pigmentation with a 0·79% overestimation (figure 12). However, some studies (detailed in appendix 1 pp 21–29) have reported negative bias or no bias for many pulse oximeters in people with dark skin pigmentation, and suggested that the direction and magnitude of bias is likely to depend on multiple patient-specific and device-specific factors. Better data are needed to understand these factors and their clinical implications (especially in LMICs) and to clarify why pulse oximeter accuracy in real-world practice differs from laboratory testing for regulatory approval.

There are several well intentioned standards and guidance documents that fall short of ensuring the safe and equitable performance of pulse oximeters.^289^, ^290^ Regulatory documents stipulate that transmittance pulse oximeters have some margin for error,^290^ but this potential variability in performance is not widely known by clinicians and is often inadequately accounted for in clinical practice, in which exact SpO_2_ cutoffs (eg, <90%) are used to direct care provision. In view of an accepted margin of imprecision for pulse oximeters, and variable performance due to patient factors, the role of absolute SpO_2_ cutoffs should be better addressed in clinical guidelines.

The 2013 guidance from the US Food and Drug Administration (FDA) introduced a requirement that at least 15% of participants in device performance validation studies had to have dark skin, but it did not detail what constituted dark skin, enabling subjective—and probably biased—interpretations. This guidance recommended only a small number of total participants in validation studies (around ten). As a result, only two people with dark skin tested would be likely to be tested per device, meaning that there would be insufficient power to detect differences due to skin pigmentation.^283^ Lack of harmonisation across existing standards, confusing and inconsistent terminology (eg, approved *vs* certified *vs* compliant *vs* clinically approved), non-intuitive statistical measures (eg, accuracy of root mean square error and non-disparate bias), and incomplete requirements for equitable performance across factors known to affect SpO_2_ measurement (eg, skin pigmentation diversity and low perfusion) are all opportunities for improving regulatory standards.

Both the FDA guidance and ISO standards for pulse oximeters have been under revision to better address these issues. Both should at a minimum include clear guidance to ensure that people with a diverse range of skin pigmentation are included in device development strategies. In early 2025, the FDA released a draft of their updated guidance, which recommends larger validation cohorts, more diversity of skin pigmentation (now defined by objective measures), and improved product labelling to inform clinicians and users of the limitations of pulse oximetry.^291^ Although this new guidance is a step in the right direction, regulatory changes alone are unlikely to eliminate skin pigment-related bias in pulse oximeters. As regulatory standards continue to evolve to address these issues and account for emerging data, we firmly advocate for the use of regulatory-compliant pulse oximeters that are intended for clinical use across all populations.

To ensure the safe and efficient delivery of oxygen from various sources in health facilities with many different types of respiratory tools and devices imported and donated from multiple jurisdictions, standards for oxygen systems in LMICs should be harmonised. A particular problem is the absence of universal standards for oxygen cylinder connectors, meaning oxygen often cannot be given to patients even when present in the facility.

The absence of cross-geography interoperability for oxygen equipment is part of a much larger problem with medical devices and indeed other technologies (eg, power outlets and plugs), but we note that progress is possible when governments act collectively. The EU directive regarding universal phone and tablet chargers provides inspiration for what collective political will can achieve. There are key parallels between these devices and oxygen devices. Different oxygen devices have different connectors, adaptors, and valves, which ties health-care providers into specific manufacturers and oxygen providers (ie, lock-in); incompatible accessories result in waste; and when an oxygen delivery system is faulty, all components rather than only the faulty parts might need to be replaced. All these issues increase consumer costs and produce unnecessary waste. The arguments against harmonisation also have parallels in the personal device industry, with the main opposing argument in both sectors being that forcing the industry to invest in modifying its products would lead to price increases.^292^ We encourage governments and their national standards bodies to advance discussions on how to develop universal standards for the equipment that is most essential to national medical oxygen systems, while also maintaining an environment for innovation.

The medical oxygen and respiratory care device industries, like most industries, are dominated by products designed and developed for patients in high-income countries. Despite the ISO Strategy 2030 goal to increase participation from “developing countries”,^293^ only six of the 31 participating members of the Anaesthetic and Respiratory Equipment Technical Committee are from LMICs. The most glaring issue is the absence of representation from sub-Saharan Africa: Uganda is the only participating member.^294^ Given that 134 of 195 countries are classed as LMICs by the World Bank, ISO should set targets that at the very least 50% of all participating members should be from LMICs by 2030. Membership in ISO is drawn from national standards bodies, so a concerted effort is required to engage national standards bodies from LMICs.

Similar to how low representation of LMICs on the ACT-A oxygen emergency taskforce weakened the COVID-19 oxygen response, the lack of LMIC involvement in global standard-setting bodies contributes to functionality issues with many oxygen-related devices in LMICs.^13^ Over time, greater LMIC representation in ISO should deliver better-quality oxygen products for LMICs. In the meantime, frustration with an opaque and inaccessible international medical device standard-setting and regulatory-approval architecture has contributed to the establishment of new regional entities, such as the African Medicines Agency, which has a mandate to establish its own standards and regulations.^295^ We note the concern that parallel regional standards systems could further fragment the medical device market and demotivate investment in products, but appreciate that the current medical device regulations and standards system is not working for many LMICs and is a barrier to local innovation and industrial development.

### Partnering with the medical oxygen industry

*“Companies should provide long-term [at least 3 years] guarantees to maintain the oxygen equipment when it breaks. Most of the warranties last just one year. They should also train our staff to fix the equipment and give them access to spare parts when needed.”*

*—Doctor, Sierra Leone*

The medical oxygen industry, like the pharmaceutical industry, is part of the public health infrastructure, and therefore collaboration is essential, with both small and large oxygen companies. To facilitate this collaboration, companies with a major stake in the medical oxygen industry should develop specific access goals and measure progress against them (eg, volume of oxygen delivered to LMICs). It is essential that these goals are part of company key performance indicators and annual reports, and there should be access teams that report to senior management. Companies should also consider access programmes in LMICs, with tailored products or services. For example, Masimo designed a pulse oximeter intended for paediatric patients in low-resource settings (funded through the Bill & Melinda Gates Foundation); Drive DeVilbiss, Sanrai International, and UNICEF collaborated on a mobile concentrator (with funding from the UK Foreign and Commonwealth Development Office), and Air Liquide has established an Access Oxygen programme (table 4) in Senegal, South Africa, and Kenya.^296^, ^297^, ^298^ To ensure that more companies embrace the access agenda, the Access to Medicine Foundation should be supported to engage the oxygen industry in the same way that it has worked with the pharmaceutical industry for more than 20 years.^299^

Governments and industry need to work closely together to ensure that LMICs have functional medical oxygen systems. There are encouraging examples of effective local public–private partnerships.^230^ At the global level, after three industry roundtables in 2020–21,^300^ Unitaid and the Clinton Health Access Initiative signed memorandums of understanding with the liquid oxygen companies Air Liquide, Linde, and, later, Global Gases. These agreements have led to a reported increase in access to liquid oxygen and a corresponding 43% reduction in the cost of cylinder refills in five countries (Eswatini, Lesotho, Mozambique, Zambia, and Zimbabwe).^301^ These are the first official partnership arrangements between global health agencies and the oxygen industry, and deserve recognition. Other initiatives across multiple LMICs have also shown the benefits of collaboration with industry.^118^, ^302^ Before these partnerships, many governments did not consider liquid oxygen to be a viable option, with hospitals lacking the infrastructure and technical capacity to deliver liquid oxygen. There is therefore an opportunity for the liquid oxygen industry to expand its market, and for governments to further diversify oxygen sources.

As promising as these public–private partnerships facilitated by global health agencies are, challenges remain. An audit of US Agency for International Development oxygen programmes showed delays in liquid oxygen installations in several countries.^303^ Another external review revealed concerns about equipment maintenance and financial sustainability beyond the grant period.^118^ The liquid oxygen industry had shown little interest in serving new health-care markets in these settings previously—it took independent, non-profit third parties to bring government and industry together to break down the barriers. We support greater investment in public–private models of oxygen provision, but with the caveat that these models should be independently assessed to measure their impact on access to medical oxygen and provide evidence on how to partner with industry most effectively.

#### The oxygen industry and pandemic preparedness and response

*“We should be prepared and not be surprised when we encounter the next respiratory pandemic.”*

*—Doctor, Ethiopia*

As an industry that produces an essential medicine during respiratory pandemics, the medical oxygen industry is also a crucial part of the global pandemic preparedness and response architecture. But unlike other industries, especially the vaccine industry, the medical oxygen industry does not yet have the internal policies and programmes or the external associations or relationships with global health agencies to deliver effectively on that role.^172^ During COVID-19, governments with leverage over oxygen companies serving large industrial clients took drastic actions to prohibit oxygen production for purposes other than use in the health sector and to cap prices (eg, India).^266^ Other governments required oxygen companies to trigger force majeure clauses in their contracts to divert all oxygen to the health sector (eg, South Africa).^266^ By contrast, governments with limited leverage largely failed to secure additional oxygen supplies from industry and hospitals ran out. In 2021, *Gasworld* published a reflection on the experience of the oxygen industry during the COVID-19 pandemic, calling on industry to work closely with public health actors to prevent oxygen shortages when the next pandemic hits.^304^

We urge the medical oxygen industry to fully engage with national, regional, and global pandemic bodies to ensure that oxygen is fully embedded in preparedness and response plans for the next pandemic and that the industry is ready to respond quickly in the case of future emergencies when large quantities of oxygen are urgently needed, especially in LMICs. GO_2_AL should make industry engagement with leading global pandemic preparedness and response bodies a priority.

## Monitoring progress in oxygen coverage

*“I think the lack of oxygen was a monumental failure that should not be allowed to happen again.”*

*—Son of deceased COVID-19 patient, Kenya*

Accurate and timely data are essential for effective evidence-informed decision making.^305^ Yet these data have been sorely lacking for medical oxygen systems, with the COVID-19 pandemic revealing major gaps in the tools used to measure oxygen access.^306^

**Figure 13:**
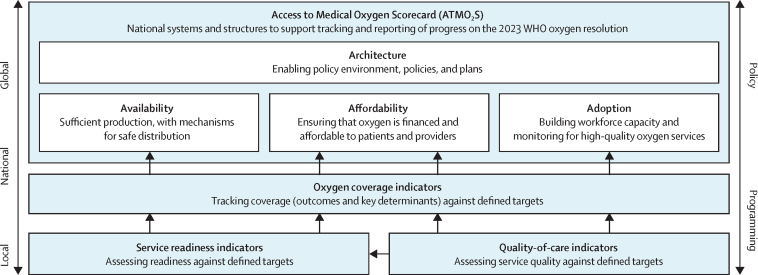
Proposed approach and indicators for a national medical oxygen monitoring framework

In this section we propose a new set of indicators for measurement of oxygen service coverage and present a new scorecard, ATMO_2_S, that governments can use to monitor progress towards universal oxygen access (figure 13). These indicators are intended to be used and adapted by health facilities, governments, and other stakeholders to drive continuous improvements in access to safe, affordable, and high-quality oxygen services, and by researchers to better understand the structures, processes, and outcomes of medical oxygen systems. ATMO_2_S provides a high-level framework for national governments and global health agencies to track progress on oxygen access against the 2023 WHO Oxygen Resolution.

To develop our approach to measuring the performance of national medical oxygen systems, we reviewed published and grey literature to identify measurement approaches and tools (appendix 1 pp 13–17), sought additional data and tools from our network of experts, and used the Commission's guiding theoretical frameworks (appendix 1 pp 3–4) and the 2023 WHO Oxygen Resolution^15^ to develop a set of ten core service indicators and a national scorecard. The indicators and scorecard were then revised with feedback from stakeholders, including WHO, governments, and implementing partners who had trialled or implemented these or similar sets of indicators. Panel 15 summarises our key findings.

Panel 15 **Monitoring progress towards universal oxygen coverage—key findings**

• Indicators used to measure medical oxygen service coverage are inadequate, rely too heavily on equipment availability, and do not sufficiently assess patient-level access or the appropriateness, affordability, or quality of service provision. In response, we developed ten core oxygen coverage indicators for monitoring coverage nationally and subnationally and the Access to Medical Oxygen Scorecard to track and report progress in implementing the 2023 WHO Oxygen Resolution.
• Clinicians, researchers, governments, implementing organisations, and funding agencies have shared responsibility to address the knowledge gaps in medical oxygen systems and to build a learning health system that can consistently generate and translate new learning into policy and practice.
• New generations of implementation and health-systems researchers, health economists, hospital management information systems data champions, data-literate clinical and policy leaders, and equitable multidisciplinary partnerships are needed.

### ATMO_2_S

*“Even the concept of oxygen as a medical therapy was not really understood by the main stakeholders—especially government officials.”*

*—Doctor, Ethiopia*

In response to the 2023 WHO Oxygen Resolution, ministries of health will need to report back to the World Health Assembly in 2026, 2028, and 2030 about 20 requests made of governments. To support this process, the Commission, in consultation with ministries of health and WHO advisors, developed ATMO_2_S (appendix 1 p 86). We mapped the WHO resolution to the four domains in the Frost and Reich framework^307^ for access to health technologies in LMICs to produce 19 indicators (table 6). ATMO_2_S is intended for national policy makers to qualitatively assess oxygen policy, strategy, and governance capability against commitments in the 2023 WHO Oxygen Resolution, to support oxygen system planning, and is directly informed by the core coverage indicators.^15^

**Table 6:** Domains and indicators in the Access to Medical Oxygen Scorecard (ATMO_2_S)

	**Indicator**	**2023 WHO Oxygen Resolution items**
**Architecture**		
1.1	We include medical oxygen in national essential medicine lists (or an equivalent—eg, a pharmacopoeia)	1, 5
1.2	We have included medical oxygen in national strategic and operational health plans (eg, as a national oxygen strategy), which includes a realistic costing and financing plan	2
1.3	We include medical oxygen in our approved national emergency and disaster preparedness and response plans and drills	8
1.4	We have coordination mechanisms in place across state and non-state actors to support effective partnerships, evidence use, advocacy, and leveraged capabilities to increase and sustain oxygen access	18, 19
**Availability**		
2.1	We can produce or acquire sufficient medical oxygen supply to meet need during routine conditions	11, 13
2.2	We can promptly increase medical oxygen supply to meet surges in demand at national and subnational levels (eg, outbreaks and disasters)	8
2.3	We have an up-to-date quantification of national medical oxygen need that is linked to production and procurement	4, 11
2.4	We have guidance documents and contracts to connect production (eg, gas companies, distributors) and end-user organisations (eg, hospitals, district health boards) at national and subnational levels	13
2.5	We have regulatory processes for ensuring medical oxygen and oxygen-related devices, including pulse oximeters, meet quality and safety standards, and accountability mechanisms for those responsible for supply	3, 17
2.6	We have mechanisms for safe distribution of medical oxygen that cover all facilities, to ensure minimal gaps in availability at the point of care for all patients in need of medical oxygen	13, 17
**Affordability**		
3.1	We have budget allocations for the key priorities within national medical oxygen plans and are able to access and release funds in a timely way	15
3.2	Our procurement and tendering processes for medical oxygen supplies, services, and devices, including pulse oximeters, are transparent and reflect a competitive market price	15
3.3	We have mechanisms in place that ensure that the provision of medical oxygen services at the point of care is affordable to providers and uses appropriate technologies (devices, spare parts, consumables, servicing)	16
3.4	We have mechanisms in place that ensure oxygen is affordable to all patients who need it (eg, insurance, financial assistance, subsidies)	11
**Adoption**		
4.1	We have sufficient trained and equipped health-care workers to provide medical oxygen services safely and effectively to patients	9
4.2	We have sufficient trained biomedical engineers or technicians, with access to appropriate equipment and supplies, to manage medical oxygen technology safely and effectively	10
4.3	We have indicators for monitoring and evaluating medical oxygen systems, including financing, supply, need, patient access, and quality of care (safety and appropriateness) at national, subnational, and health facility levels	4, 6, 7, 11, 20
4.4	We have reliable public health information being communicated to the population about medical oxygen services available	12
4.5	We have systems (eg, guidelines) in place to ensure appropriate use of oxygen among those who need it	6, 7

The “we” in each statement refers to the national government, and was framed as such to increase ownership and accountability.

A key challenge in conceptualising a national scorecard is the wide variation in the maturity of oxygen systems across countries. We have been intentional in designing indicators that can evolve as oxygen systems—and the data used to monitor them—evolve. Countries should therefore primarily use the scorecard to benchmark their own progress, and should avoid international comparisons and rankings. The complete scorecard, with instructions for how to complete and score each domain, is available in appendix 1 (p 88).

### Oxygen coverage indicators

*“I was sitting in the emergency room for close to two hours with no help, struggling to breathe.”*

*—Nurse who had severe COVID-19, South Africa*

The set of ten core oxygen coverage indicators that we devised is intended to help national and subnational programmes to track objective data on oxygen service accessibility, service readiness, quality of care, and affordability to inform policy and programme strategy (table 7). The supplemental facility readiness and quality-of-care indicators that we devised are intended to provide stakeholders with deeper understanding of oxygen services at a subnational and local level (appendix 1 p 101).

**Table 7:** Core indicators for monitoring universal access to safe, affordable, high-quality pulse oximetry and medical oxygen services

	**Definition**	**Target**
Pulse oximetry coverage*	Proportion of patients presenting to hospital with acute illness or undergoing surgery whose SpO_2_ is documented at triage or admission (or during non-emergency surgery)	>80%
Oxygen production and storage capacity*	Mean (and maximum) monthly production volume (in Nm^3^) of medical oxygen, and storage capacity, of each production facility (air separation units for cryogenic production of liquid oxygen or pressure-swing or vacuum-swing adsorption oxygen plants)	Individualised country targets
Pulse oximeter and oxygen availability*	Number and proportion of acute ward areas in health facilities with a functional pulse oximeter and an oxygen supply sufficient to meet patient need in the past month	100%
Pulse oximetry and oxygen service accessibility	Proportion of the population who can access, within 2 h, a health facility that provides low-flow oxygen services, including pulse oximetry measurement and monitoring	100%
Hypoxaemia prevalence	Proportion of patients attending a health facility who have hypoxaemia (ie, SpO_2_<90%) at triage or admission	None (reflects magnitude of oxygen need)
Oxygen coverage	Proportion of patients with hypoxaemia (ie, SpO_2_<90%) at triage or admission to a health facility who receive oxygen therapy within 1 h	>80%
Hypoxaemia-related mortality	Proportion of patients attending a health facility who have hypoxaemia (ie, SpO_2_<90%) and die before discharge or within 30 days	Individualised country targets
Clinical workforce	Number of doctors, nurses, and midwives per 10 000 population	≥44·5 clinicians per 10 000 population^249^
Biomedical engineering workforce	Number of biomedical engineers (defined broadly as per WHO^244^) per 10 000 population	≥0·4 biomedical engineers per 10 000 population†
Protection against catastrophic health expenditure	Proportion of patients receiving medical oxygen whose out-of-pocket expenditure on oxygen services is greater than 1% of their total annual household expenditure or income	<5% of patients experience catastrophic health expenditure

These indicators are most useful when used and interpreted collectively, because no one indicator in isolation provides an adequate representation of oxygen-related service provision. All targets should be adapted to the local context and given a timeline. SpO_2_=oxygen concentrations in peripheral blood. Nm^3^=normal cubic metres.

* Highest priority and most feasible indicators.

† In the absence of accepted global targets for biomedical engineering workforce, we propose a new target (appendix 1 p 102).

Selection of indicators required us to balance desirability with feasibility, in recognition of the fact that some of the ideal or preferred indicators would be difficult to obtain outside research projects, and that relevant proxies need to be used for routine programme monitoring and integration into health information systems. Most of the data needed for these indicators can be obtained from existing or adapted data collection tools or sources. Efforts to obtain these data should be integrated into existing facility readiness, disease incidence, and clinical care data systems (ie, to avoid duplication of effort or the unnecessary creation of separate data systems for oxygen).

Our proposed indicators are intended to be adapted to meet user needs and capacity, including specifying targets that reflect the local context. In keeping with the overall focus of the Commission, we focus on people who need basic oxygen services guided by pulse oximetry. Our indicators do not cover all the broader health systems requirements for provision of oxygen service coverage but capture some key aspects (eg, the health workforce and power supply). While these factors have been framed as indicators for oxygen services, many are equally relevant for other services. We encourage users to seek linkages, and to recognise oxygen-focused indicators as potentially useful signal functions to assess the readiness and quality of other services (eg, emergency, critical care, paediatric, neonatal, anaesthetic, and surgical services).

In the past 5 years, efforts have been made to integrate core oxygen service coverage indicators into routine health information systems in LMICs, particularly in countries with national oxygen plans. Although these efforts are still in their early stages, emerging lessons will be useful for other countries seeking to strengthen data systems tracking oxygen service coverage (panel 16).

Panel 16 **Best practice spotlight—integration of oxygen service coverage indicators into routine health information systems in Uganda and Rwanda**

As part of their national oxygen plans, the governments of Uganda and Rwanda sought to integrate oxygen service coverage indicators into routine health management information systems. In Uganda, the ministry of health has been tracking key clinical and oxygen supply indicators since October, 2022, and in Rwanda, the ministry of health started tracking indicators in January, 2024. Clinical indicators include pulse oximetry coverage (ie, the proportion of patients screened for hypoxaemia with pulse oximetry upon admission) and oxygen coverage (ie, proportion of patients with hypoxaemia who received oxygen therapy). Supply indicators capture data for oxygen consumption and stockouts (including days per month with stockout). Data are aggregated by facility-based data managers and reported monthly into the national electronic reporting platforms.

By systematically tracking these indicators, health-care authorities have been able to better understand the prevalence of hypoxaemia (and, by extension, oxygen need), track consumption and stock levels, and track utilisation of oxygen therapy, from the facility level to national level. However, integration of these indicators has been a long process and involved many challenges. Selection of indicators required extensive stakeholder engagement to arrive at agreement on what was prioritised and feasible. Critically, these discussions included ministry of health and programme managers, who would be using the data, and facility-based data managers, who would be aggregating and reporting the data. Reporting rates were initially low, clinical documentation was frequently missing (eg, records not kept on file), and clinical documentation (eg, vital signs charts) were often not standardised. Improvement of clinical documentation has been a key part of clinical quality-improvement activities and standardisation of reporting tools is underway. Efforts are also underway to create more user-friendly dashboards with real-time insights at the national level, to facilitate data-driven decision making, and to provide timely reports for facilities to support local quality-improvement efforts.

### Improving data quality and bridging knowledge gaps

*“There was a struggle to convince governments that oxygen therapy was the main mode of management for COVID.”*

*—Doctor, Ethiopia*

We found many gaps in the data available to understand and address oxygen access challenges. To improve data quality and bridge these gaps, we propose key steps to improve how data are collected and identify priority research areas.

#### Better tools

*“I saw babies on CPAP [continuous positive airway pressure] in the NICU die due to poor nursing care, typically a lack of monitoring.”*

*—Neonatal ICU nurse, Ethiopia*

Although many data collection tools that address aspects of oxygen coverage are available, very few address the core oxygen coverage indicators in adequate detail to inform decision making (appendix 1 p 107). The most comprehensive tools captured between six and eight of the ten core oxygen coverage indicators; common gaps include assessment of oxygen production and storage capacity, engineering health workforce, prevalence of, and mortality from, hypoxaemia, and oxygen coverage in people with hypoxaemia who needed it. We identified two broad categories of tools that provide data on oxygen services: facility readiness tools and clinical audit tools.

Facility readiness tools typically involve a facility visit, questions for managers, and direct inspection of ward areas (we excluded tools administered remotely). Many of the identified survey tools—including widely used tools such as WHO's Harmonized Health Facility Assessment^308^ and the Demographic and Health Survey's Service Provision Assessment, and most service-specific assessment tools—contained a yes-or-no question about the availability of oxygen equipment. The answers to this question reveal little about adequacy, functionality, technology management, production and storage capacity, or cost. However, a growing number of critical care and oxygen-focused assessment tools successfully integrate data for the adequacy of pulse oximeters and oxygen, stockouts, and the enabling facility environment, providing a more useful, holistic picture of oxygen readiness.^100^, ^101^, ^102^, ^107^, ^115^, ^309^, ^310^, ^311^, ^312^

Gathering clinical data about oxygen coverage typically involves review of clinical documentation (eg, patient case notes) to capture key oxygen-related clinical practice data. Very few quality-of-care tools capture even the most basic data for whether pulse oximetry was used or oxygen was provided to people who needed it and almost no tools addressed issues of the quality of oxygen services. Some oxygen-focused projects have successfully used clinical audit approaches to capture data for pulse oximetry and oxygen coverage, hypoxaemia-related mortality, and aspects of quality of care (eg, timeliness, appropriateness, monitoring, SpO_2_ targeting, and weaning).^100^, ^311^

We identified five opportunities to improve data collection tools and to provide better oxygen data for policy makers, facility managers, and the health workforce to action. First, facility readiness and clinical data should be disaggregated by level of facility and population (eg, age and ward type) to better understand gaps and inequity. Second, pulse oximetry data should be assessed and reported alongside oxygen data, given that one dataset cannot be understood without the other. Third, when assessing facility readiness, a measure of equipment adequacy to meet need (eg, per patient or per bed, stockouts), oxygen production or storage capacity, or both, and functionality of equipment should all be reported. Fourth, in assessments of oxygen-related quality of care, data for pulse oximetry, timeliness of care, and monitoring beyond the initial assessment period should be included. Finally, assessment of health-care and biomedical engineering worker capacity should be included. This assessment should include demonstrated competency and actual practice in addition to numbers of trained staff.

#### Better research

Research into oxygen-related topics has increased substantially in the wake of the COVID-19 pandemic, including studies on facility readiness, clinical use of oxygen, models of service provision, pulse oximetry implementation in primary health care, and new device development. To foster further research and close remaining evidence gaps, we have produced a non-exhaustive list of priority research areas (panel 17). We also make four general recommendations to researchers and research funders.

Panel 17 Priotity research areas for oxygen systems**These research priorities are drawn from the vast body of information studied by the Commission and testimony received from stakeholders. The list is not exhaustive, and we recommend a research priority-setting exercise similar to that done to establish research priorities for childhood pneumonia.263

**Description**

• Establish the burden of hypoxaemia in community and facility settings, by condition, age, gender, and socioeconomic status
• Identify and describe populations who need long-term oxygen therapy, by condition, age, gender, and socioeconomic status, and the cost of meeting this need
• Establish the oxygen coverage gap at national and subnational levels, disaggregated by facility type and ward level
• Explore systematic drivers of high oxygen service costs for different patient groups, across different contexts and levels of the health system
• Describe the extent, facilitators, and consequences of anti-competition practices in the medical oxygen market
• Describe the impact of smoking cessation, increased vaccination, reduction in occupational exposures, and improved air quality on demand for medical oxygen
• Understand the physical and psychological effects, experience, and consequences of hypoxaemia and oxygen therapy, by condition, age, gender, and socioeconomic status

**Delivery**

• Assess the effectiveness, reliability, and sustainability of different mixed-supply oxygen systems across diverse contexts to better understand the best use cases and the influence of local context and systems
• Assess the effectiveness, reliability, and sustainability of different models of oxygen system management (including production, distribution, maintenance, and repair) across diverse contexts and levels of the health system
• Establish the cost-effectiveness of different medical oxygen sources, across contexts and levels of the health system
• Develop and assess strategies to improve the rational and efficient use of oxygen, such as different SpO_2_ cutoffs and oxygen conservation devices
• Develop and assess strategies to improve the safety of oxygen therapy for at-risk populations, particularly preterm neonates (eg, different SpO_2_ targets, oxygen automation devices)
• Develop and assess strategies to reduce out-of-pocket costs for patients for medical oxygen services by level of health facility, condition, age, gender, and socioeconomic status, nationally and subnationally
• Assess the effectiveness of different oxygen delivery devices, including advanced respiratory devices, for neonates, children, and adults with different conditions and across different contexts
• Measure the effectiveness of different models for building workforce capability for medical oxygen therapy use in different contexts, and at scale
• Assess the implementation of pulse oximetry in primary health care, with particular emphasis on understanding population and contextual factors that influence adoption, sustainability, and impact on referral and clinical outcomes of pulse oximetry use
• Assess the effectiveness and cost-effectiveness in terms of mortality, morbidity, and quality of life of different SpO_2_ cutoffs for referral and treatment initiation across different patient conditions
• Establish the functionality, impact, and sustainability of the investments in medical oxygen systems made during the COVID-19 pandemic, including the current operational capacity of pressure swing adsorption oxygen plants, oxygen concentrators, and pulse oximeters.
• Assess the impact of oxygen support and related tools provided to low-income and middle-income countries by members of the Global Oxygen Alliance

**Development**

• Develop mathematical models to forecast the number of people who might need medical oxygen, the volumes of oxygen required for a range of future emergencies (including pandemics, natural disasters, climate change, and conflicts), and the capacity of oxygen systems to meet these needs
• Develop tools and methods to collect and integrate hypoxaemia and oxygen service data into routine health information systems and disease surveillance systems, including for epidemic preparedness and response
• Develop oxygen production, storage, and delivery technologies that are suitable for humanitarian and emergency contexts (eg, portable devices, devices with integrated power solution, devices that can tolerate heat and humidity)
• Develop measurement devices and tools that utilise artificial intelligence and the internet of things to better monitor medical oxygen production, demand, use, and quality
• Develop low-cost, high-quality pulse oximeters that work effectively across all age groups and skin pigmentations

SpO_2_=oxygen concentrations in peripheral blood.

First, health economics, implementation, and health systems research that approaches medical oxygen from a complex systems perspective and draws on methods that seek context-sensitive understanding of how oxygen services are provided and can be strengthened should be encouraged. Second, given that many priority research questions rely on complex multidisciplinary systems assessments, collaborative research with governments and implementers in LMICs and equitable partnerships between established and emerging research institutions should be supported. Third, research efforts should focus on the most pervasive challenges to medical oxygen access and the populations facing greatest disadvantage, including poor, rural, and humanitarian emergency contexts. Finally, core oxygen indicators should be integrated into broader health research agendas and studies, including service-readiness assessments, quality-of-care evaluations, and interventional studies involving neonates and children, emergency care, critical illness, surgical care, and acute primary health care.

In the coming years, we expect academic and research institutions, global health agencies, and research donors to collaborate to ensure that research gaps are closed and that the findings are used to shape policies and programmes to close medical oxygen coverage gaps.

#### Local evidence champions: building a learning health system

*“Among the hypoxaemic patients needing mechanical ventilation, none of them survived. This was my first experience of patients on ventilation not surviving.”*

*—Doctor caring for patients with COVID-19, Bangladesh*

The learning health system is a concept whereby health data are harnessed to inform evidence, and evidence is then fed back into practice in a continuous cycle to improve system efficiency, quality of care, and patient outcomes.^313^, ^314^ This approach relies on access to relevant data and the analytic capacity to use these data. Oxygen systems have the capacity to generate a wide range of valuable data, not only for patient-centred outcomes, but also for system efficiencies, from production to delivery—data that can be used to build a learning system.

The first step is to map and select core data components and key performance indicators (as described earlier in the Commission), which link to national oxygen plans. When possible, these indicators should be integrated into existing data platforms. Several countries have incorporated or are piloting inclusion of SpO_2_ and oxygen treatment measures within health management information systems, such as the District Health Information System (panel 11). Patient registers are another valuable tool. The Swedevox register for patients prescribed long-term oxygen therapy in Sweden shows the benefits of monitoring long-term trends for understanding policy impacts.^315^, ^316^

The next step is the use of end-user-friendly dashboards, in which multiple data streams are collated to support learning and action. The concept of a national medical oxygen grid emerged in India during the pandemic; it allowed individual health facilities and subnational and national governments to monitor data for oxygen use, sources of supply, and projections of upcoming need.^317^ This system, which can interface with other health management information systems, can be used to support patient management and national forecasting (see the India case study; appendix 2). Another example comes from Lesotho, where the ministry of health and the non-governmental organisation Jhpiego implemented a medical oxygen stock management dashboard to monitor real-time oxygen stock availability and use, forecast demand, and predict stockouts.^318^ The automated electronic dashboard was based on an oxygen stock and patient need checklist used by nurses to collect data regularly, and could be used to analyse supply and demand and generate stock status reports. Over a 6-week implementation period in 2022, the dashboard alert system identified that seven (58%) of the 12 secondary hospitals monitored were at risk of more than one oxygen shortage, and all potential oxygen shortages were prevented.

Central to the success of a learning health system is empowerment of decision makers to effectively use the information generated. To support these efforts, data staff and analysts need to be recognised as part of the essential health workforce, and capacity for using and applying evidence is needed across the system, from health-care workers to policy makers. Many of the solutions and innovations that stood out during the Commission were locally grown, contextually tailored, and driven by a small group of dedicated actors. In the case studies from Malawi and Bangladesh (appendix 2), the early adoption of oxygen solutions, such as the national scale-up of pulse oximetry in IMCI guidelines, was linked to the role of long-term research partnerships with collaborative oxygen and pulse oximetry research. We therefore encourage improved partnerships and mutual research priority setting, with support for local champions in advocating the need for robust evidence.

## Conclusion—looking beyond 2030

*“There is a need for global solidarity to ensure that everyone who needs oxygen regardless of where they come from is able to get it in a timely manner that saves lives.”*

*—Person who survived severe COVID-19, Kenya*

Oxygen is an essential part of care for patients of all ages and many different health conditions, yet remains poorly accessible to many people in LMICs. Our analyses showed that the oxygen coverage gap is enormous: around 70% of people in LMICs who need oxygen for acute medical or surgical conditions do not have oxygen access. Almost 400 million people need oxygen each year, and so investing in oxygen systems is a highly effective approach to reducing morbidity and mortality across a range of patient groups. Improved medical oxygen security will not only accelerate progress towards the SDGs, but also prepare for future humanitarian emergencies.

To ensure that the Commission's findings are translated into measurable actions, we make 52 recommendations for governments, the oxygen industry, global health actors, advocacy groups, academics, and professional bodies to work towards by 2030 (panel 18). We also recommend that an independent body assess progress against these recommendations in 2027, with the results of this assessment made publicly available.

Panel 18 **Recommendations for 2030**

**Governments**

• Develop and implement a costed national medical oxygen plan, with estimates of the quantities of pulse oximeters, medical oxygen, and supporting systems required to meet the needs of neonates, children, and adults at all levels of the national health system during routine service delivery and emergencies. Oxygen should be part of both national health plans and emergency preparedness plans, with progress monitored and reported using the core oxygen coverage indicators and ATMO_2_S.
• Increase domestic spending for national medical oxygen systems, with a line item for oxygen in government health budgets that separates capital and operating costs.
• Include pulse oximetry and medical oxygen services in national universal health coverage schemes so that patients do not face financial barriers to seeking care or catastrophic costs after treatment.
• Establish a focal point (eg, an oxygen desk) within federal and state ministries of health to coordinate activities across health, education, energy, transportation, industry, and other relevant ministries, and with the medical oxygen industry, global health agencies, and other stakeholders to improve the management of the national medical oxygen system.
• Adopt a national minimum target of at least 0·4 biomedical engineers or equivalent per 10 000 population (equivalent to one engineer per 100 hospital beds), and report progress in the ATMO_2_S.
• Include pulse oximetry and oxygen in pre-service medical, nursing, and allied health curriculums and in-service training for both integrated (eg, Integrated Management of Childhood Illness, Integrated Management of Adolescent and Adult Illness) and sector-specific (eg, tuberculosis, malaria, Comprehensive Emergency Management of Obstetric and Newborn Care, Basic Emergency Obstetric and Newborn Care) initiatives.
• Update all clinical guidelines, essential medicines and medical device lists, and related health policies to include pulse oximetry and medical oxygen, with a special focus on accelerating the use of pulse oximetry as a routine assessment tool in primary, secondary, and tertiary health-care facilities for newborns, children, and adults.
• Integrate core oxygen service coverage indicators into routine health and logistic information systems, especially the District Health Information System.
• Ensure that all relevant national laws, regulations, and standards mirror the WHO *International Pharmacopoeia* definition of medical oxygen to include both oxygen generated by cryogenic technology (ie, 99·5% oxygen) and oxygen generated by vacuum-swing or pressure-swing adsorption technology (93% oxygen). This process should involve emphasising that both forms of medical oxygen are safe and that medical grade oxygen from varying sources can be safely mixed and provided to patients.
• Ensure a competitive national market for medical oxygen by implementing laws that prohibit anti-competition practices and regulations that encourage transparent pricing and competitive tendering of oxygen contracts, and establish whistleblower and independent arbitration processes via which all parties can fairly pursue grievances.
• Negotiate contracts with private medical oxygen-related companies that deliver affordable, reliable installation and commissioning, clinical and engineer training, service contracts with multi-year warranties, and access to spare parts and technical documentation.
• Increase knowledge generation around oxygen systems through increased support to universities and other research and training institutions and work towards a learning health system committed to continuous learning and improvement.

**Oxygen industry**

• Adopt specific access to medical oxygen targets and implement flagship oxygen access programmes. Report progress in company annual reports.
• Collaborate with global health agencies (eg, GO_2_AL) and national governments to document progress towards oxygen access targets and to highlight best practice programmes.
• Commit to greater price transparency (especially for liquid oxygen) and ensure compliance with competition policy and medical oxygen-related regulations in all jurisdictions.
• Increase engagement with national governments to test public–private partnership models in which the private sector manages the oxygen system (or components of it) for national, state, or local governments.
• Accelerate investments in innovations that improve the cost-effectiveness of pulse oximetry, medical oxygen, and related therapies, including by reducing energy costs, and collaborate with academic institutions in LMICs to optimise products for use in these settings.
• Design products to meet the needs of patients and health facilities in low-resource settings (eg, pulse oximeters that perform equally well on all ages and skin pigmentations, devices that conserve oxygen) to facilitate access and address affordability, durability, and usability. Ensure end-users have a direct link to manufacturers for efficient troubleshooting and maintenance of equipment.
• Contribute to global efforts to increase manufacturing of key components of medical oxygen systems (eg, pulse oximeters, oxygen plants, oxygen concentrators, respiratory care devices, and spare parts) in LMICs, and to strengthen supply chains, especially across sub-Saharan Africa.

**Global health agencies**

• Ensure that all global clinical guidelines, essential medicines lists, and training materials— including both integrated and sector-specific initiatives—appropriately include pulse oximetry, oxygen, and related therapies for all patient populations.
• Ensure that health surveys, facility assessment tools, and related data tools appropriately include pulse oximetry, medical oxygen, and related therapies for all patient populations, especially the Demographic and Health Survey's Service Provision Assessments, Service Availability and Readiness Assessments, and Harmonized Health Facility Assessments. Champion the use of the Commission's core oxygen coverage and related indicators.
• Accelerate inclusion of pulse oximetry, medical oxygen, and related therapies in global emergency preparedness instruments, especially the State Parties Annual Report, the Joint External Evaluation framework, the Benchmarks for International Health Regulations, the guidelines for the National Action Plans for Health Security, and other initiatives related to the Pandemic Agreement.
• Increase support to national governments to develop national oxygen plans, leveraging global tools for planning, implementing, and monitoring, including the WHO oxygen plan template, ATMO_2_S, and related tools.
• Coordinate global oxygen-related activities within and across agencies; engage with the GO_2_AL to maximise impact and minimise fragmentation and duplication of effort, leverage the GO_2_AL strategic plan and investment case for agency resource mobilisation, and to facilitate stronger engagement with industry.
• Ensure that at least 50% of future global oxygen investments (eg, grants, loans, equity) are dedicated to supporting the operational costs of sustaining national medical oxygen systems, with a focus on the capability of the clinical and engineering workforce. Target the countries with the largest unmet needs, the least capacity to self-finance, and the greatest potential impact (in terms of number of lives saved) during routine service delivery and emergencies.
• Ensure that procurement processes for oxygen supplies align with national medical oxygen plans, assess prices based on total cost of ownership, and consider local maintenance capacity, availability of spare parts, energy sources, and other matters that may hamper the sustainability of the equipment. Funding agencies should use core oxygen service coverage data to make investment decisions and measure success, and should prioritise the areas with the greatest inequity.
• Increase access to high-quality global, national, and health facility data for medical oxygen needs, coverage, gaps, and support: include pulse oximetry and medical oxygen coverage as a routine indicator in UN databases (eg, the WHO Global Health Observatory, UNICEF child health coverage database), make medical oxygen data produced by GO_2_AL and its members public, and include hypoxaemia as a risk factor for death and disability in the next iteration of the Global Burden of Disease.

**Global health donors**

• Include oxygen-systems-strengthening components in all relevant funding offerings and promote integration across programmes and sectors in recognition of the essential role of oxygen services in addressing a wide range of health conditions and health investment priorities.
• Increase investments in medical oxygen to accelerate both achievement of the Sustainable Development Goals and pandemic preparedness by contributing to GO_2_AL's annual US$4 billion resource mobilisation target for 2025–30, which represents 12% of the annual estimated cost of financing the acute medical oxygen gap estimated by the Commission.
• Support the inclusion of medical oxygen in the eighth replenishment of The Global Fund to Fight AIDS, Tuberculosis and Malaria and invest in The Global Fund to ensure that it can continue to help eligible countries close medical oxygen coverage gaps.
• Announce a specific pandemic fund call to strengthen preparedness for respiratory pandemics, with a strong focus on surveillance and diagnostic tools and therapies, including pulse oximetry and medical oxygen and related therapies.
• Increase funding to improve the quality of oxygen care provided to patients, including funding for health-care worker training and professional institutions, quality of care, health management, and guideline development and implementation.
• Increase financing from development finance institutions to private sector oxygen providers, including small and medium enterprises in low-resource settings, and to encourage more large-scale public–private partnerships to manage national oxygen systems.
• Require grantees to apply best practices in the procurement of pulse oximeters, medical oxygen, and related equipment by requiring them to use total cost of ownership metrics to assess prices, including operational and energy costs, and to avoid distorting and displacing local actors, including private sector actors, in the provision of support.
• Encourage grantees to use the core oxygen coverage indicators and support the integration of pulse oximetry and oxygen data into routine health and logistic information systems.
• Increase funding for the repair and recommissioning of broken oxygen equipment—especially by increasing biomedical engineering workforce capacity. Stop perpetuating a throw-away culture by only funding and donating new equipment.
• Actively participate in the GO_2_AL to maximise the impact and efficiency of donor investments and to minimise duplication of effort, ensuring that there is open sharing of donor-funded data and tools, and an increase in the funding of open-source oxygen technologies.
• Increase funding for oxygen-related research, including implementation and health systems research in partnership with LMIC governments and research institutions.

**Civil society and patient advocacy**

• Integrate pulse oximetry and medical oxygen access into civil society health advocacy and programme activities, including efforts to hold health facilities, governments, industry, and global health agencies accountable for their oxygen access commitments.
• Engage with governments about the development and implementation of national oxygen plans and other relevant oxygen policies and programmes.
• Establish patient advocacy groups to ensure that the voices of patients who need oxygen for acute medical and surgical procedures and for long-term oxygen therapy (and their caregivers) are heard by all stakeholders, especially governments, industry, and global health agencies.
• Engage the GO_2_AL to support civil society and patient pulse oximetry and medical oxygen advocacy and other activities in LMICs.
• Mobilise civil society organisations and patient advocacy groups globally to increase the impact of World Oxygen Day.

**Academic and research institutions**

• Increase oxygen-related research, with particular emphasis on funding and training implementation science, health systems, and health economics researchers. Encourage collaborative, respectful, and equitable research partnerships.
• Partner with government and industry to generate and apply oxygen implementation and health systems data in a timely way to contribute to a broader learning health systems culture and environment. Champion the use of the Commission's core oxygen coverage and related indicators.
• Identify gaps in workforce capability that require the creation of new programmes or certifications to meet local needs for sustainable oxygen systems.
• Embed theoretical and practical content on pulse oximetry and medical oxygen into curriculums for clinical and biomedical professionals to create sustainable workforce capability.
• Research the major gaps in access to oxygen, especially in the research priority areas we outlined (panel 17).

**International Organization for Standardization**

• Review all standards relating to the provision of medical oxygen and related therapies to assess alignment with WHO's increasing access to medical oxygen resolution and make recommendations, including on how to reduce fragmentation across regions in medical oxygen-related standards.
• Ensure that at least half of all participating members of the Anaesthetic and Respiratory Equipment Committee (ISO/TC 121) and related sub-committees are representatives from the national standards bodies of LMICs.

**Professional organisations**

• Formalise national biomedical engineering professional associations or societies in each country and establish or fortify regional bodies to improve the capacity of the profession to increase access to medical oxygen.
• Ensure that relevant clinical societies are supporting the clinical workforce in the delivery of high-quality oxygen services, including the use of pulse oximetry at all levels of the health system.

ATMO_2_S=Access to Medical Oxygen Scorecard. LMICs=low-income and middle-income countries. GO_2_AL=Global Oxygen Alliance.

As we enter the final 5 years of the SDG era, multiple forces are rapidly reshaping population health. Poor diet, smoking, and ageing are likely to continue to contribute to rising numbers of patients with non-communicable conditions, and climate change is exacerbating infectious disease, air-pollution, and extreme-heat related deaths.^319^ At the same time, slow economic recovery from the COVID-19 pandemic and rising debt levels are putting pressure on national health budgets and international health financing. Governments and global health agencies are struggling to meet these challenges and at the same time to prepare for the ever-present threat of another pandemic. The UN reports that, at current rates of progress, none of the health SDGs will be achieved by 2030.^320^

The punishing experience of many LMICs during the pandemic has led to calls for a total transformation of the field of global health, culminating in the Lusaka Agenda.^255^ Rather than fund and implement disease-specific initiatives, the Lusaka Agenda recommends that global health agencies should support integrated delivery of services through investing in resilient health systems. In recognition of the role of poverty, discrimination, and other social injustices in creating and sustaining health inequities,^321^ global health actors should put health justice and equity at the centre of everything they do.^322^ In response to the Lusaka Agenda, there are calls for a shift in focus to sustainable health or planetary health care in shaping the global agenda beyond 2030, with the aim to not only protect human health, but also to restore the health of the planet.^323^, ^324^

We welcome this new direction and the centring of equity and sustainability in global discourse and action. We also welcome the Global Health 2050 report^325^ (the third report from the *Lancet* Commission on Investing in Health), which set a new target of a 50% reduction in the risk of premature death by 2050. Oxygen is a core treatment for 12 of the 15 priority health conditions for greater country investment highlighted in Global Health 2050. With this Commission, and the recommendations we put forward, increasing access to medical oxygen can be a global health exemplar. Centring oxygen investments around national plans and health-systems strengthening will ensure that oxygen is integrated into all health services, benefitting all patients, everywhere. Embracing oxygen systems and respiratory care devices that are energy-efficient and oxygen-conserving can contribute to planetary health by reducing carbon emissions. Investment in local innovators to maintain and repair broken oxygen equipment should reduce the financial, human, and environmental costs of device graveyards. Confrontation of the root causes of poor oxygen access will require action on addressing social injustices that drive health inequity more broadly. National medical oxygen systems can be at the forefront of efforts to create the future we want by ensuring the long-term health and sustainability of people and the planet.

### Contributors

## Declaration of interests

HRG declares salary support, research grants, and consultancies from the Royal Children's Hospital Foundation, the Australian National Health and Medical Research Council, the Bill & Melinda Gates Foundation, ELMA Philanthropies, the Asian Development Bank, and Cambridge Economic Policy Associates; declares travel support from the Royal Children's Hospital Melbourne; and holds advisory or leadership roles with the Oxygen for Life Initiative and Lifebox Foundation. CK declares research funding from the Bill & Melinda Gates Foundation, the Swedish Research Council, the US National Institutes of Health, and the Save the Children–GSK partnership, and has an advisory role with the Lifebox Foundation. AER declares a grant from Clinton Health Access Initiative for Commission work (including salary support; GR-02385) and a research grant from the UK National Institute for Health and Care Research (NIHR). LG declares salary support from the Bill & Melinda Gates Foundation and leadership roles with TEAMFund, Rice360, and la Caixa Foundation. HC declares grant funding from NIHR and the Baszucki Brain Research Foundation, consulting fees from WHO, and is Co-Editor in Chief of the *Journal of Global Health*. KC led development of the COPD guidelines for the Latin American Thoracic Society. ME declares research grants from Wellcome and NIHR, and a leadership role with UK Research and Innovation. AGF declares research grants from the Bill & Melinda Gates Foundation, ELMA Philanthropies, a Save the Children–GSK Partnership, the Swedish Research Council, and WHO, and a leadership role with the Oxygen for Life Initiative. AZG declares travel support from the Royal Children's Hospital Melbourne. RL declares travel support from UNICEF. MSL declares research grants from the US Agency for International Development, Moore Foundation, McGovern Foundation, Robert Wood Johnson Foundation, Unitaid, PATH, US Food and Drug Administration, Wellcome Trust, and WHO, and has received payment for presentations from the University of California, Los Angeles and Johns Hopkins University. DL is co-chair of the Global Oxygen Alliance. EDM declares research grants from the US National Institutes of Health, the Bill & Melinda Gates Foundation, the US Agency for International Development, the US Centers for Disease Control and Prevention, Thrasher, Moderna, travel support from WHO, and leadership and advisory roles with the Union, the Lifebox Foundation, and the WHO pneumonia guideline development group. JO declares travel support from UNICEF. SSP declares general salary support from Uppsala University and UNICEF Sweden. HJZ declares research grants from the Bill & Melinda Gates Foundation and the South African Medical Research Council and leadership roles with the Forum of International Respiratory Societies, the Pan African Thoracic Society, and WHO pneumonia guideline development group. MG reports consultancy fees from Cure Kids Foundation, is a co-owner of the non-profit cooperative Azimut 360, declares royalties or licences from a utility model for a trailer for the production of medical oxygen with solar devices, and has received travel and meeting support from Cure Kids Foundation and Azimut 360. SRCH declares salary support from Cure Kids New Zealand. ILL declares consulting fees from the International Centre for Diarrhoeal Disease Research, Bangladesh. SEA declares salary support from the Clinton Health Access Initiative. FS declares salary support from a US Agency for International Development grant. FEK declares grants from Meeting Targets and Maintaining Epidemic Control (awarded to Makerere University School of Public Health; 102533), the Swedish Research Council, Clinton Health Access Initiative (awarded to Makerere University School of Public Health), and DT Global International Development UK (awarded to Makerere University School of Public Health; 21886-001) and holds advisory roles with the Global Oxygen Alliance. TB declares research grants and consultancies from Wellcome Trust, NIHR, UNICEF, the World Bank, the US Agency for International Development, and PATH. All other authors declare no competing interests.
