# Correction to *Lancet Glob Health* 2025; 13: e528–84

**DOI:** 10.1016/S2214-109X(25)00101-9

**Published:** 2025-03-26

*Graham HR, King C, Rahman AE, et al. Reducing global inequities in medical oxygen access: the Lancet Global Health Commission on medical oxygen security.* Lancet Glob Health *2025; **13:** e528–84*—In panel 18 of this Commission, the Global Oxygen Alliance's resource mobilisation target of US$4 billion for 2025–30 was mistakenly described as an annual target. This correction has been made to the online version as of March 26, 2025.

