# Supplementary material for: Reducing global inequities in medical oxygen access: the Lancet Global Health Commission on medical oxygen security
Source: Lancet Glob Health. 2025 Feb 17;13(3):e528–84. doi: 10.1016/S2214-109X(24)00496-0 (PMC11865010; doi:10.1016/S2214-109X(24)00496-0)
Supplement: Supplementary appendix 1 [file mmc1.pdf]

THE LANCET
Global Health

Supplementary appendix 1

This appendix formed part of the original submission and has been peer reviewed. We post it as supplied by the authors.

Supplement to: Graham HR, Carina King C, Rahman AE, et al. Reducing global inequities in medical oxygen access: the *Lancet Global Health* Commission on medical oxygen security. *Lancet Glob Health* 2025; published online Feb 17. https://doi.org/10.1016/S2214-109X(24)00496-0.

APPENDIX 1

Table of Contents

1. Theoretical frameworks and definitions ... 4
1.1 Conceptual frameworks – need, coverage, and access ... 4
1.1.1 Health Service levels ... 6
1.2 Other definitions ... 7
1.2.1 Hypoxaemia and hypoxia infographic ... 7
2. Methodology ... 9
2.1 Literature review methods ... 9
2.1.1 Acute hypoxaemia review ... 9
2.1.2 Long term oxygen therapy need review ... 9
2.1.3 Oxygen service coverage and tools review ... 14
2.1.4 Care packages review ... 18
2.1.5 Cost of oxygen services review ... 19
2.1.6 Role of skin tone in pulse oximetry readings review ... 21
2.1.7 Barriers and facilitators of implementing medical oxygen therapy solutions ... 30
2.2 Needs, Coverage, Costing the Gap estimation ... 33
2.2.1 Estimating global oxygen need ... 33
2.2.2 Estimating current oxygen service coverage ... 74
2.2.3 Estimating the cost gap ... 75
2.3 Country case studies ... 79
2.4 ATMO$_2$S development ... 86
2.5 Thanzi la Onse modelling ... 87
3. Tools and checklists ... 88
3.1 ATMO$_2$S policy scorecard ... 88
3.1.1 ATMO$_2$S definitions ... 88
3.1.2 ATMO$_2$S detailed scorecard ... 89
3.1.3 Intended use of ATMO$_2$S ... 96
3.2 Oxygen Coverage Indicators ... 96
3.2.1 Core oxygen coverage indicators ... 96

3.2.2 Oxygen facility readiness and quality of care indicators 101
3.3 Oxygen-related tools, and standards 107
3.3.1 Mapping of existing oxygen service coverage tools against proposed indicators 107
3.3.2 Additional oxygen-related standards and tools 110
4. Supporting data... 113
4.1 Detailed oxygen need data and additional analyses... 113
4.2 Detailed oxygen and pulse oximetry coverage data 116
4.3 Challenges and solutions coding tree 129
5. Consultations 131
Patients, caregivers, and clinicians 131
Ministries of Health 131
Industry 131
Experts 131
Oxygen Access Collaborators 132
6. References... 133

Tables

Table 1 Long-term oxygen needs in COPD in published literature 10
Table 2 Mean pulse oximetry bias by skin pigmentation and ethnicity 21
Table 3 Included studies in estimating skin pigmentation bias with absolute value of bias....... 24
Table 4 - Search strategy for barriers and facilitators review 30
Table 5 Acute medical conditions requiring medical oxygen therapy...................................... 34
Table 6 Disease burden of acute medical conditions 38
Table 7 Assumptions related to hospitalization needs for acute medical conditions................ 40
Table 8 Assumptions related to hypoxaemia proportion estimates for different acute medical conditions 45
Table 9 Assumptions related to region-specific hypoxaemia proportion estimates for different acute medical conditions 46
Table 10 Estimation of surgery rates by World Bank regions.. 56
Table 11 COPD burden mapping in GBD 57
Table 12 LTOT needs in COPD in published literature 57
Table 13 Minimum oxygen volume assumptions for different acute and chronic disease / conditions and perioperative need 58
Table 14: Justification for the minimum oxygen volume assumptions for different acute and chronic disease / conditions and perioperative need 60
Table 15 Country classifications for analysis, by World Bank region and income classification 68
Table 16 Set-up and assumptions for the Oxygen Service Coverage estimates 74
Table 17: Summary of data used to estimate the cost of oxygen (per litre) 76
Table 18 Calculating the cost to meet the gap in acute medical and surgical oxygen need in LMICs.. 78
Table 19: Country case study academic literature search strategy 81

Table 20: Country case study countries and methodological adaptations 85
Table 21: Calculating the number of biomedical engineers per 10,000 population 97
Table 22 **Core indicators for monitoring of medical oxygen coverage** and access to safe, affordable medical oxygen services globally, nationally (and sub-nationally) 99
Table 23 Key indicators for assessing facility service readiness to provide safe, affordable medical oxygen services... 102
Table 24 Key indicators for **measuring quality of medical oxygen services to patients**, including proposed minimum targets 105
Table 25 Numbers of patients (millions) needing medical oxygen for acute medical, surgical, long-term oxygen therapy, and COVID-19, by incomes and World Bank region*, and minimum volume of oxygen required to meet need (million cubic metres/Nm^3), 2021 113
Table 26 Oxygen need and volume by World Bank region in High-income Countries (HIC) and Low- and Middle-Income Countries (LMIC) 115
Table 27 Trend in estimated people needing oxygen (millions) from 2010 to 2021 using the Global Burden of Disease data for: acute medical (excluding COVID-19), perioperative, and long-term oxygen indications... 115
Table 28 Pulse oximeter availability in LMIC health facilities, meta-estimates (%, 95% CI)..... 116
Table 29 Oxygen availability in LMIC health facilities, meta-estimates (%, 95% CI)................ 119
Table 30 Pulse oximetry coverage to patients in LMIC health facilities, meta-estimates (%, 95% CI) 122
Table 31 Oxygen coverage to patients with hypoxaemia in LMIC health facilities, meta-estimates (%, 95% CI) 125
Table 32 Coding tree for challenges and corresponding solutions....................................... 129

Figures

Figure 1 Effective Coverage Cascade applied to medical oxygen services 5
Figure 2 "Access Framework: Health Technologies for Poor Countries" adapted for medical oxygen systems[1] 5
Figure 3 Forest plot of LTOT prevalence among COPD patients... 11
Figure 4 PRISMA flowchart for 'Prevalence of long-term oxygen therapy' systematic review 13
Figure 5 PRISMA flow diagram for the oxygen service coverage and tools literature review 17
Figure 6: PRISMA flow diagram for the cost-estimate rapid evidence synthesis 20
Figure 7 PRISMA flowchart of effect of skin tone on pulse oximetry readings systematic review 23
Figure 8 PRISMA inclusion for studies in the enables and barriers systematic review.............. 32
Figure 9: Countries selected for case studies 79
Figure 10 Meta-estimates of pulse oximeter and oxygen availability in health facilities and coverage for acutely unwell patients, by decade from 2000 to 2024.................................... 128

Data and more information

Oxygen Commission updates and more information, including additional datasets, publications, resources and a comprehensive pulse oximetry and oxygen library can be found at: https://stoppneumonia.org/latest/lancet-global-health-oxygen-commission/

1. Theoretical frameworks and definitions

1.1 Conceptual frameworks – need, coverage, and access

The Commission relied on two conceptual frameworks – the *Effective Coverage Cascade* and *Access Framework for Health Technologies*[1] - as well as drawing from health systems thinking, including the WHO Health Systems Building Blocks and UNICEF Supply Chain Maturity Model.[2]

We adapted the 'Effective Coverage Cascade' to understand and measure oxygen need, service availability and readiness, and identify the pervasive challenges that need effective solutions. This Effective Coverage Cascade builds upon previous efforts to incorporate need, access, utilisation, and quality aspects of coverage, all centred on equity.[3-5] Following this approach, we have described the Medical Oxygen Service Cascade (Figure 1).

Quantifying the **target population** involves assessment or estimation of oxygen need, defined as *the number of people requiring medical oxygen therapy in a specified period*. Oxygen need includes people not presenting for care (which that can be difficult to measure) and diverse populations. People requiring oxygen services can face multiple barriers at each step of the coverage cascade. They may never attend a facility that could provide oxygen services (**service contact coverage**) either because they don't seek care, or because they attend a small facility but fail to complete referral. This could be due to reasons such as geographical distance, cost, lack of knowledge/awareness, or socio-cultural barriers, and is akin to the first and second 'delays' described in the Three Delays model.[6] They may attend a facility that is not service ready (**input-adjusted coverage**) due to unavailable or nonfunctional equipment (e.g. pulse oximeter, oxygen equipment, electricity), or low workforce capacity. Those who arrive at a facility that has basic oxygen service capacity may still not receive oxygen therapy (**intervention coverage**) or not receive it appropriately (**quality-adjusted coverage**), due to challenges such as missed identification of need, inadequate capacity, competing priorities, and inadequate guidelines or support to achieve high quality clinical practices (e.g. overuse that limits the ability to give to those who actually need it). Patient rejection can further compromise oxygen care (**user adherence-adjusted coverage**) due to challenges such as excessive cost, fear/stigma of oxygen, discomfort, or inadequate patient counselling and support. Ultimately, the goal is to ensure that those who need oxygen receive a clinical benefit (**outcome-adjusted coverage**), such as resolution of respiratory failure and prevention of death and disability. We use the term **oxygen access** to refer to the extent to which people who need oxygen therapy receive it safely and effectively, regarding this as broadly synonymous with quality-adjusted coverage.

Figure 1 Effective Coverage Cascade applied to medical oxygen services

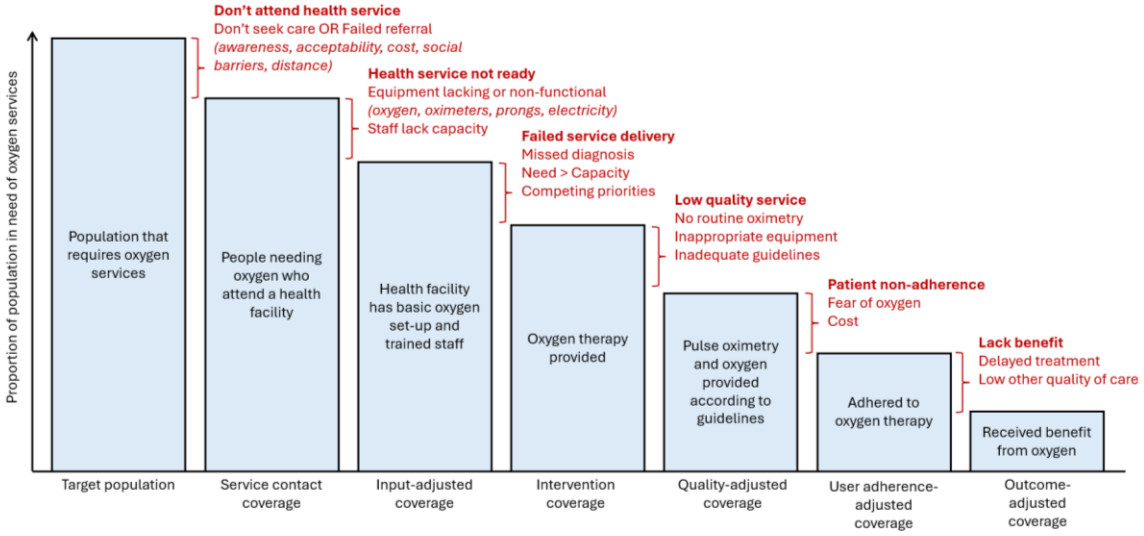

We used the *Access Framework for Health Technologies* to inform the political economy analysis and the development of the ATMO$_2$S and oxygen coverage indicators. This framework centres *availability*, *affordability*, and *Adoption* while explicitly recognising the background *architecture* required for each.[1 7] We adapted the Access Framework for medical oxygen systems (Figure 2).

Figure 2 "Access Framework: Health Technologies for Poor Countries" adapted for medical oxygen systems[1]

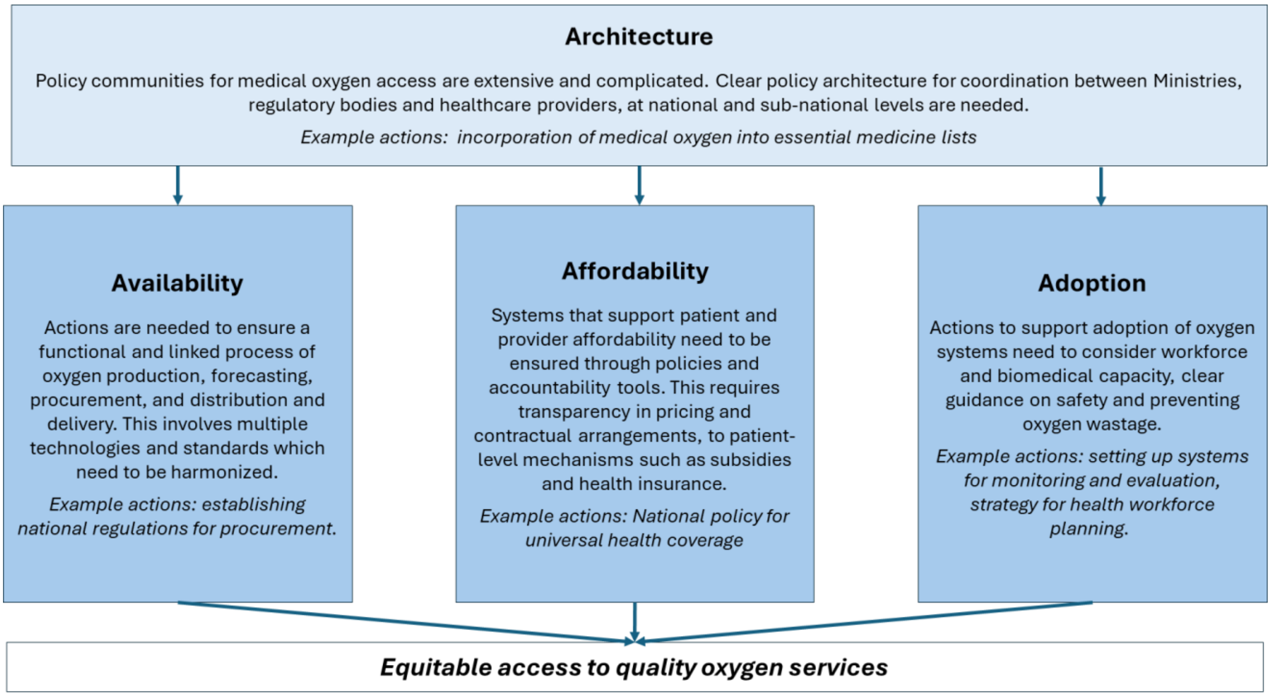

1.1.1 Health Service levels

	**Community**<br>(home, community health workers)		**Level 1 - Primary**<br>(health centre, rural hospital)	**Level 2 - Secondary**<br>(first-level, district, provincial hospital)	**Level 3 - Tertiary**<br>(referral, specialist, teaching hospital)
**Typical infrastructure**	Based in the community, typically through outreach visits and programs during daylight hours.		Outpatient clinic operating during daylight hours with small number of beds (primarily for stabilisation or short observation). May have overnight maternity area.	Inpatient care (~100-300 beds), including emergency department and operating rooms for major and minor procedures, including Caesarean section.	Inpatient care (~300-1,000+ beds), including well equipped operating rooms s and basic intensive care facilities.
**Typical capability**	Provided by community nursing and/or allied health workers.<br>Referral of patients with acute or complex conditions.		Staffing by general paramedical, nurse/midwife staff during daylight hours, with limited availability overnight. Referral of patients requiring admission, surgery, complicated delivery.	Staffing by general medical, nurse/ midwife, paramedical staff 24/7, with access to major specialist staff (e.g. visiting surgeon, anaesthetist, obstetrician, paediatrician). Referral of patients requiring intensive care support or sub-speciality care.	Same as Level 2 with additional surgical and medical subspecialities, specialist nurses, and 24/7 intensive care staffing.
**Typical services**	Focus on health promotion, prevention, and chronic care.		Able to provide care for 90-95% of acute cases, including minor trauma, infections, and basic obstetric care. Provides chronic and preventive care, including antenatal care, nutrition, immunization.	Able to provide acute resuscitation and care for 95-99% of severely ill patients, including Level 1 plus inpatient, surgical, and obstetric care, and some major specialties (e.g. paediatrics, obstetrics).	Same as Level 2 with additional: prolonged care of ventilated patients with multi-organ failure, complex surgery, and some sub-specialties.
**Oxygen services**	No global standard.<br>May include long-term oxygen therapy (LTOT) under a specialist respiratory care program with home pulse oximetry and basic oxygen therapy (i.e. supplemental low-flow oxygen therapy).		No global standard.<br>May include pulse oximetry for screening of severely ill patients with or without basic oxygen therapy (i.e. supplemental low-flow oxygen therapy) for stabilisation or short-stay observation.	Pulse oximetry for assessment and vital signs monitoring; continuous pulse oximetry in operating room and severely ill patients. Basic oxygen therapy for inpatients. Often offer higher level care including CPAP for patients with acute respiratory failure and short-term ventilation in anaesthesia.	Same as Level 2 with additional: non-invasive and mechanical ventilation requiring oxygen-air mixing and blood gas analysis.

Notes: adapted from WHO Emergency and Essential Surgical Care guide, 2012.[8] ICU, intensive care unit; CPAP, continuous positive airway pressure;

1.2 Other definitions

1.2.1 Hypoxaemia and hypoxia infographic

Panel A

Oxygen Delivery To Human Tissues

Successful delivery of oxygen to human tissues (DO_2) is defined by the following factors:

$$DO_2 = CO \times (Hb \times SaO_2 \times 1.34) + (PaO_2 \times 0.003)$$

Where:

Actual O_2 content of blood (or CaO_2) = (Hb x SaO_2 x 1.34) + (PaO_2 x 0.003)

CO = Cardiac output (effective circulation)

Hb = concentration of functional hemoglobin in blood (see Panel B.)

SaO_2 = % saturation of Hb in arterial blood

PaO_2 = partial pressure of O_2 dissolved in arterial blood

Important Concept (cont'd to Panel B)

Hypoxemia refers to 'low **arterial** content or partial pressure of oxygen (PaO_2 or CaO_2)'

vs

Hypoxia refers to a low or inadequate **tissue** concentration or availability of oxygen

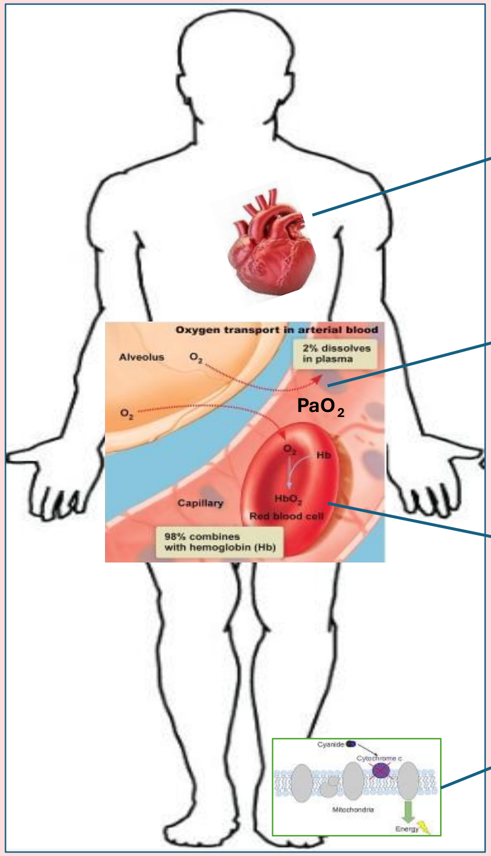

Cause(s) of hypoxia (low tissue oxygen)

Ischemic hypoxia ('pump failure')

Blood flow is too low to deliver oxygen to tissues (due to reduced cardiac output; congestive heart failure, profound hypotension)

Hypoxemic hypoxia (most common)

Actual arterial oxygen content (CaO_2) is too low due to inability to collect O_2 from ambient air (i.e., low PaO_2). Observed with high-altitude or acute and chronic lung disease.

Anemic hypoxia

Actual arterial oxygen content (CaO_2) is too low due to reduced circulating functional Hb (anemia or blood loss) or an excess of a dysfunctional Hb (sickle cell, metHb, carboxyHb etc.) that impairs binding of O_2 to functional Hb

Cytotoxic hypoxia ('impaired tissue uptake')

Tissue uptake of O2 is impaired despite readily available oxygen (i.e., mitochondrial shutdown in sepsis, cyanide poisoning etc.).

Figure 1 (Panel A). Key Concepts Surrounding Successful Delivery of Oxygen to Tissues

Panel B

Measures of Oxygenation / Detection of Hypoxemia

Important Concept (cont'd from Panel A)

Hypoxemia may not always mean tissue hypoxia in any given patient. And tissue hypoxia may occur in the absence of low arterial oxygen content (hypoxemia).

Modern-day bedside measurement devices detect hypoxemia. Yet, the ultimate goal of all bedside clinical care is to prevent tissue hypoxia. Hence, clinicians must recognize the clear utility of these tools, understand what is being measured, employ their correct interpretation for any given clinical context, and continue to value clinical examination to recognize tissue hypoxia (capillary refill time, skin examination) or biochemical markers (lactate).

Pulse Oximetry

Pulse oximetry measures sO_2 non-invasively; representing peripheral functional Hb saturation (SpO_2)

Less commonly, SpO_2 measured via special multiwavelength pulse CO-oximeters can provide information of fractional oxygen saturation (FO_2Hb).

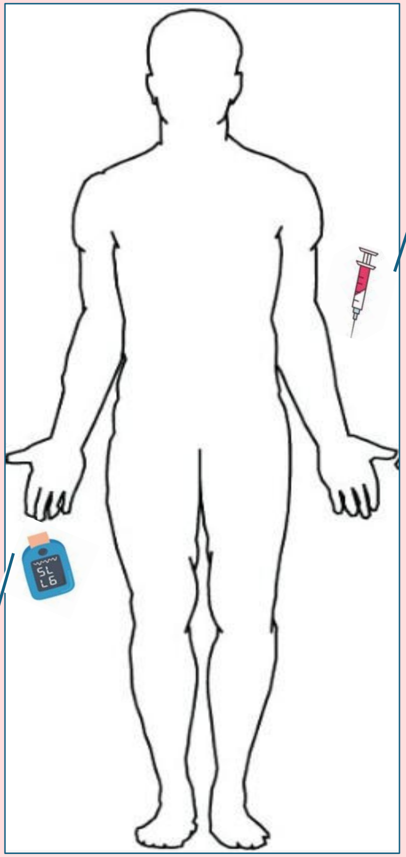

Blood Gas Analysis

Blood gas analysis measures functional Hb saturation (sO_2) when it is further termed SaO_2, SvO_2, $ScvO_2$ (depending on arterial, venous or central venous blood, respectively). Blood gas analysis also provides additional measurements of PaO_2 (partial pressure of dissolved oxygen), and fractional Hb saturation (via multiwavelength co-oximetry analysis).

Functional vs Fractional Oxygen Saturation

Functional oxygen saturation (sO_2) is the % saturation of Hb capable of transporting O_2 (functional Hb). Functional Hb = oxyHb (HbO_2) + deoxyHb (Hb) & sO_2 represents % of HbO_2.

$$sO_2 = \frac{HbO_2}{(HbO_2 + Hb)} \times 100$$

Fractional oxygen saturation (FO_2Hb) measures both functional Hb (Hb species capable of binding oxygen) & non-functional Hb (Hb species incapable of binding oxygen; Met-Hb, CO-Hb, S-Hb).

$$FO_2Hb = \frac{HbO_2}{(Hb + HbO_2 + \text{Met-Hb} + \text{CO-Hb} + \text{S-Hb})} \times 100$$

Figure 1 (Panel B). Measures of Oxygenation (Detection of Hypoxemia)

2. Methodology

2.1 Literature review methods

2.1.1 Acute hypoxaemia review

Data for this came from a separately published systematic review and meta-analysis:

Graham H. et al. (2024) The prevalence of hypoxaemia among paediatric and adult patients presenting to healthcare facilities in low- and middle-income countries: systematic review and meta-analysis.[9]

2.1.2 Long term oxygen therapy need review

Search: To identify medical oxygen need among people with chronic conditions, we conducted a systematic review with the aim to report the proportion of adults with chronic respiratory conditions requiring long-term oxygen therapy (LTOT) globally. The protocol for this review was registered in PROSPERO (CRD42023464405). We conducted the literature search in five databases (Medline, Embase, CINAHL, Web of Science, Scopus) to identify relevant articles published from inception to 2023 (searches conducted on 25 October 2023). Search terms such as "LTOT", "chronic diseases", "adults" were used with relevant keywords (additional details in Panel 1).

Inclusion and exclusion criteria: We included all types of studies, not restricted by geography or language or publication.

- Population: We included all populations with requirement for long-term oxygen therapy (including but not limited to COPD, emphysema, interstitial lung disease).
- Intervention: Long-term oxygen therapy
- Comparator: None
- Outcome: Proportion of patients with COPD who meet criteria for long-term oxygen therapy. Secondary outcomes included the proportion of patients with other chronic respiratory conditions meeting criteria for long-term oxygen therapy.
- Studies that reported oxygen and/or pulse oximeter availability at wards or facilities at any level of the health system (excluding studies that reported data from a single facility).
- Studies that reported oxygen coverage to hypoxaemic populations and/or pulse oximetry coverage including any acutely unwell patient population (excluding pulse oximetry for congenital heart screening and oxygen for long-term therapy).
- Tools used to measure oxygen service coverage, including well-established tools that are intended for broader purposes but do include core oxygen service coverage indicators.

Article screening and data extraction: We used Excel for the title and abstract screening. After the initial title–abstract screening, full-text articles were obtained of all potential studies. Title-abstract screening was independently performed by two reviewers, with any 'yes' votes going through to full-text review. Full-text review for inclusion was independently performed by two reviewers based on specified inclusion/exclusion criteria, and any discrepancies settled by a third reviewer. Data was extracted by two independent reviewers in a pre-specified excel

spreadsheet. Once the team is in consensus, by solving any kind of confusion, the data extraction format was utilized to compile the extracted data. The following data will be extracted- first author name, country, publication year, study setting, participant characteristics, number of participants, altitude, study period, study design, number of people with long-term oxygen therapy need, and health condition. We used the JBI prevalence checklist critical appraisal tool.[10]

Data synthesis: A meta-analysis was conducted to synthesize the results. For the prevalence of people requiring long-term oxygen therapy, a weighted mean and 95% confidence interval was calculated using a random effects model.

The combined searches resulted in 2807 yields after deduplication. After title, abstract and full text screening the final number of articles reporting LTOT proportion on COPD population came to 4. The PRISMA flow diagram is shown in Figure 4.

From these 314 articles, four papers were identified, which reasonably reflected the true needs of LTOT among COPD population. The meta-estimate was 4.67% (95% CI 3.43, 6.09). Table 1 shows the details of the four papers. The forest plot showing the included studies and meta-estimate of LTOT is shown in Figure 3.

Table 1 Long-term oxygen needs in COPD in published literature

Study Title	First Author	Publication year	Study period	Country	Age	Sample Size	LTOT (%)
Home oxygen therapy reduces risk of hospitalisation in patients with chronic obstructive pulmonary disease: a population-based retrospective cohort study, 2005–2012 [11]	Kyoung Hee Cho	2015	2002-2012	South Korea	>=40	36761	3.6
Frequency of comorbidities in chronic obstructive pulmonary disease, and impact on all-cause mortality: A population-based cohort study [12]	Laurent Laforest	2016	2006-2013	France	>=45	4237	8.1
The natural history of community-acquired pneumonia in COPD patients: A population database analysis [13]	Hana Müllerova	2012	1996-2005	UK	>=40	8814	4.81
Patterns and management of chronic obstructive pulmonary disease in urban and rural China: a community-based survey of 25 000 adults across 10 regions [14]	Om P Kurmi	2018	2013-2014	China	38-87	1586	2.77

Figure 3 Forest plot of LTOT prevalence among COPD patients

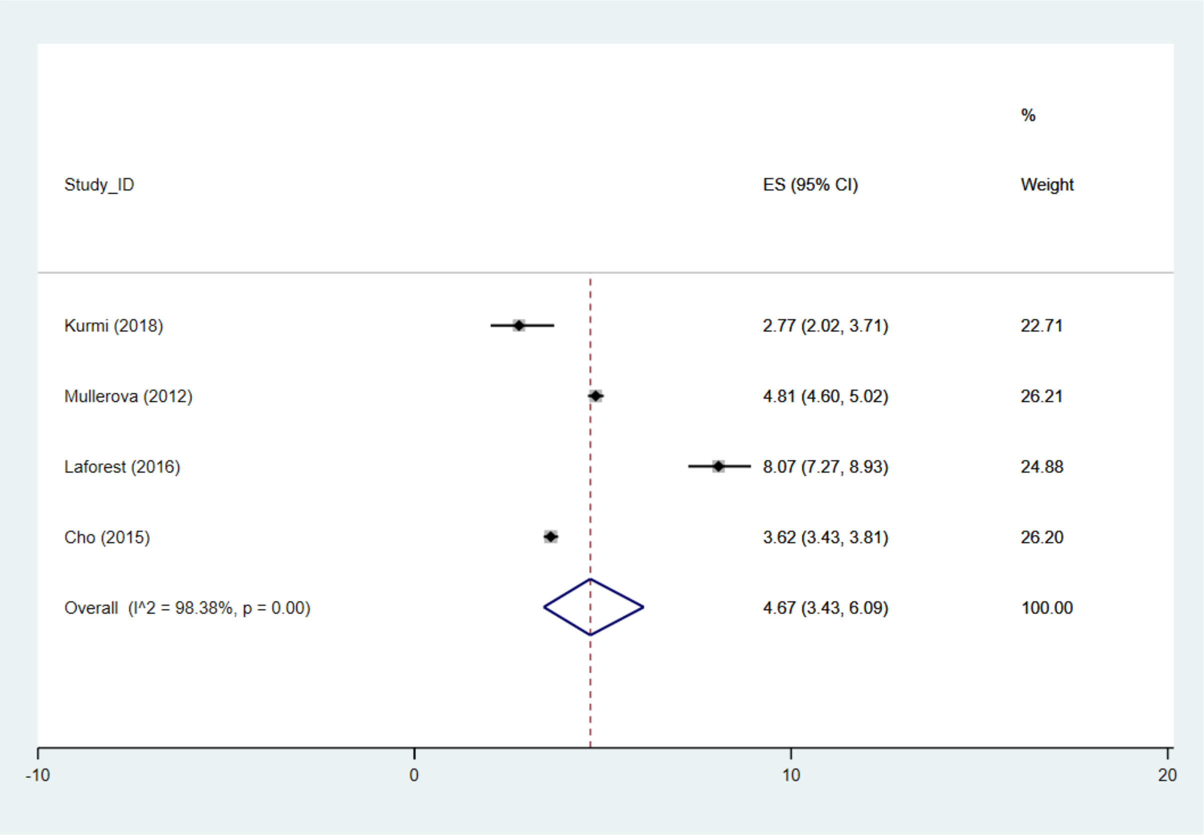

Panel 1 MEDLINE search strategy for the long-term oxygen therapy need literature review

Line	Terms	Results 24/10/2023
1	*lung diseases, interstitial/ or *pulmonary fibrosis/ or *pulmonary disease, chronic obstructive/ or *pulmonary emphysema/	80386
2	Cystic Fibrosis/	40149
3	Bronchiectasis/	8996
4	(COPD or chronic-obstructive-pulmonary-disease).tw,kf.	80938
5	1 or 2 or 3 or 4	166572
6	*Oxygen Inhalation Therapy/	8391
7	*Oxygen/	76495
8	oxygen.tw,kf.	605494
9	(long-term or home).tw,kf,hw.	1301362
10	(6 or 7 or 8) and 9	23728
11	(LTOT or home-oxygen or domiciliary-oxygen).tw,kf.	1996
12	10 or 11	23939
13	(man or men or woman or women or adult or adults or middle-age* or elderly or geriatric* or aged).tw,kf,hw.	9717885
14	exp Adult/	7975058
15	13 or 14	9717889
16	ep.fs.	2136879
17	exp epidemiologic methods/ or exp epidemiologic studies/	7462873
18	(prevalence or incidence* or epidemiol* or survey* or cohort or screening).tw,kf.	3823664
19	16 or 17 or 18	9542151
20	5 and 12 and 15 and 19	923
21	limit 20 to (case reports or comment or editorial or letter or preprint)	17
22	20 not 21	906

Figure 4 PRISMA flowchart for 'Prevalence of long-term oxygen therapy' systematic review

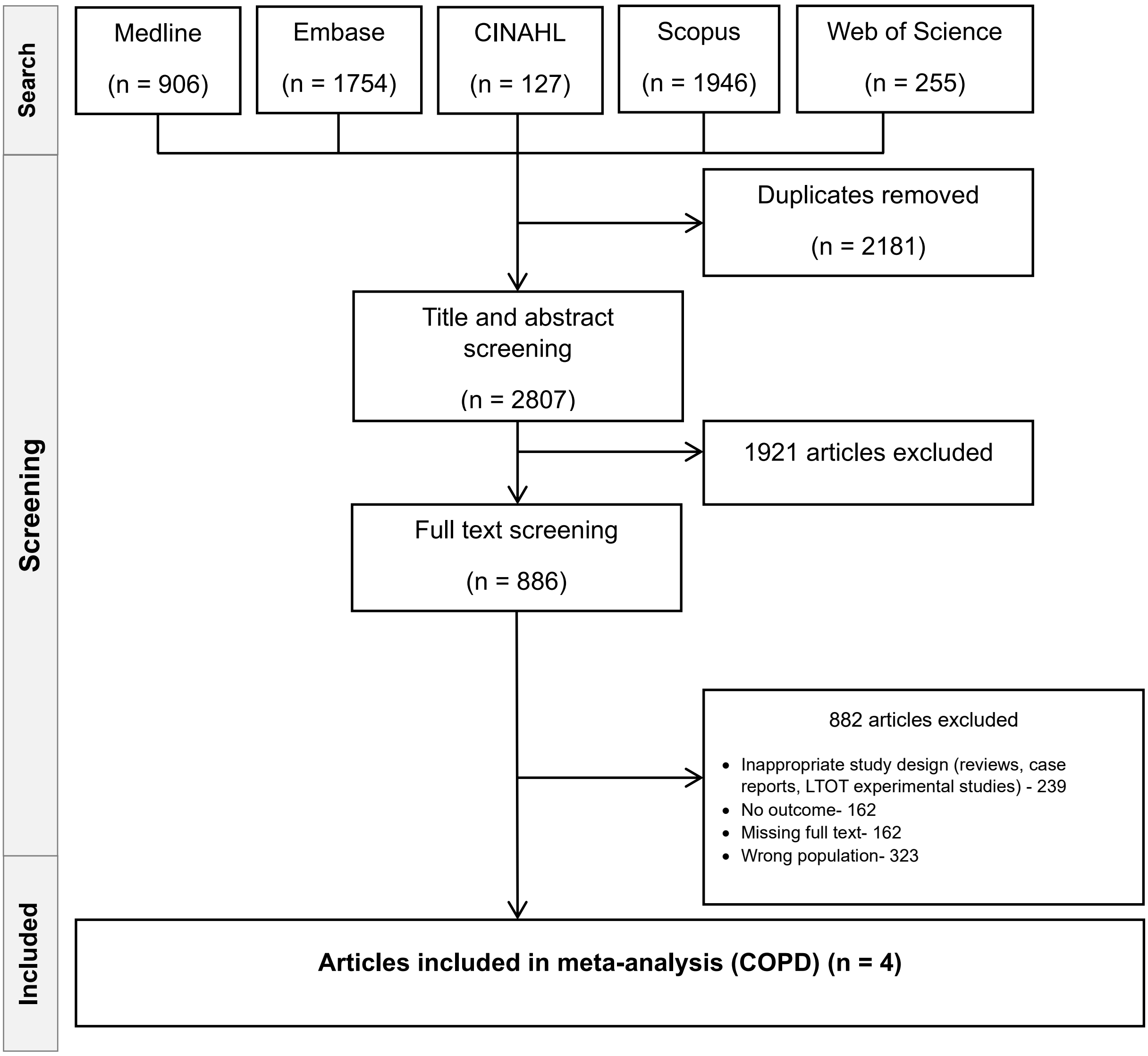

2.1.3 Oxygen service coverage and tools review

Search: We conducted a systematic literature search of academic and grey literature, to capture data on oxygen service coverage (including facility readiness, quality of care, and cost) and associated measurement tools. We searched academic databases (Embase, PubMed, MEDLINE, CINAHL, Lilacs/Lipecs, SciELO) on April 6, 2023 and updated 28 March 2024. We also manually searched for Oxygen Roadmaps and put requests through professional networks for additional published and unpublished data. The broad search terms used were: “oxygen” AND “measurement tool” (additional details in Panel 2). We also searched the reference list of included articles, for any additional relevant publications.

Inclusion and exclusion criteria: We included all types of sources, including original research, reviews, commentaries, reports, position statements and policy documents, and restricted inclusion to the year 2000 onwards with not language restriction. We included:

- Studies that reported oxygen and/or pulse oximeter availability at wards or facilities at any level of the health system (excluding studies that reported data from a single facility).
- Studies that reported oxygen coverage to hypoxaemic populations and/or pulse oximetry coverage including any acutely unwell patient population (excluding pulse oximetry for congenital heart screening and oxygen for long-term therapy).
- Tools used to measure oxygen service coverage, including well-established tools that are intended for broader purposes but do include core oxygen service coverage indicators.

Article screening: Studies were imported into Covidence for title and abstract screening by two independent reviewers with all potentially relevant studies retained for full-text review. Full-text review for inclusion was done by two independent reviewers, with HG serving as third reviewer where consensus failed. Full text access was obtained through the University of Melbourne’s library system.

Data extraction: Data were extracted into an online spreadsheet (Google), including indicators for year, country, World Bank region, income level, setting, age, population description, facility level of care, urban/rural, private/public, ward type, data collection dates, data source, and a long list of outcomes intended to match the proposed oxygen coverage indicators – broadly categorised as service readiness (service access, oxygen and pulse oximeter availability, equipment functionality, maintenance, healthcare worker readiness, biomedical engineering readiness, referral services, safety, supporting infrastructure), quality of care (pulse oximetry coverage, oxygen coverage to hypoxaemic, monitoring, acceptance, healthcare worker capacity) and cost (costs to facility/provider, costs to patients). We used a modified critical appraisal tool adapted from the JBI prevalence checklist.[10]

Data synthesis: We screened over 9000 articles, including 355 in final qualitative synthesis (Figure 5). Quantitative data on pulse oximetry and oxygen availability and service coverage were extracted into a separate Excel dataset for meta-analysis using Stata metaprop command.[15] The remaining data contributed to qualitative synthesis using the oxygen service coverage cascade broad categories.[4] Both quantitative and qualitative synthesis informed the Oxygen Service Coverage Cascade estimates (see Section 4.2). Tools were identified and described narratively.

Panel 2 OVID MEDLINE search strategy for oxygen service coverage and tools literature review

Line	Terms	Results 6/4/2023
1	((oxygen or oximet*) and (Access or coverage or quality or readiness or availability or capacity)).tw,kf,hw.	88107
2	("health services needs and demand"/ or needs assessment/ or "Delivery of Health Care"/) and (oxygen or oximet*).tw,kf,hw.	315
3	(survey or indicator* or metric or surveillance or audit? or instrument? or measure or measuring or tool?).tw,kf,hw.	3246645
4	(critical-care and readiness and survey*).tw,kf,hw.	47
5	exp world health organization/	39074
6	(world-health-organization or WHO).tw,kf.	2758088
7	developing countries/ or resource-limited settings/	80684
8	(austere or (limited adj2 resource*) or (low adj2 resource*) or (transitioning adj econom*) or (third adj world) or LMIC or LMICs or (lami adj countr*) or (transitional adj countr*) or (low adj gdp) or (low adj gnp) or (low adj gross adj domestic) or (low adj gross adj national) or ((emerging or developing or (low adj income) or (middle adj income) or (low adj3 middle) or underdeveloped or under-developed or (less* adj developed) or underserved or under-served or deprived or poor*) and (countr* or nation*1 or econom* or population or world))).tw,kf.	502018
9	exp africa/	321400
10	americas/ or exp caribbean region/ or exp central america/ or latin america/ or mexico/ or exp south america/	295820
11	europe/ or exp europe, eastern/ or exp transcaucasia/	310344
12	antarctic regions/ or exp atlantic islands/ or exp indian ocean islands/ or pacific islands/ or melanesia/ or exp micronesia/ or exp polynesia/	39616
13	New Guinea/	2131
14	asia/ or exp asia, central/ or asia, southeastern/ or borneo/ or cambodia/ or timor-leste/ or indonesia/ or laos/ or malaysia/ or mekong valley/ or myanmar/ or philippines/ or thailand/ or vietnam/ or asia, western/ or bangladesh/ or bhutan/ or exp india/ or middle east/ or afghanistan/ or iran/ or iraq/ or jordan/ or lebanon/ or oman/ or syria/ or turkey/ or yemen/ or nepal/ or pakistan/ or sri lanka/ or china/ or tibet/ or mongolia/ or asia, eastern/ or asia, southern/ or "Democratic People's Republic of Korea"/	646631

15	(Afghanistan or Albania or Algeria or Angola or Antigua or Argentina or Armenia* or Aruba or Azerbaijan or Bahrain or Bangladesh or Barbados or Barbuda or Belarus or Byelarus* or Byelorussian or Belorussian or Belorus* or Belize or Benin or Bhutan or Bolivia or Bosnia or Botswana or Brasil or Brazil or Bulgaria or (Burkina adj Fas*) or (Upper adj Volta) or Burma or Burundi or Cambodia or Khmer or Kampuchea or Cameron* or Cameroon* or (Cape adj Verde) or (Cabo adj Verde) or (Central adj African adj Republic) or Chad or Chile or China or Colombia or Comoros or (Comoro adj Island*) or Comores or Mayotte or Congo or Kongo or (Cook adj Island*) or (Costa adj Rica) or (Cote adj D'ivoire) or Croatia or Cuba or Cyprus or Czech* or Djibouti or Dominica or Dominican or (East adj Timor) or (East adj Timur) or Ecuador or Egypt or El-Salvador or (Equatorial adj Guinea) or Eritrea or Estonia or Ethiopia or Fiji or (French adj Somaliland) or Futuna or Gabon or (Gabonese adj Republic) or Gambia or Gaza or (Georgia* adj Republic) or Ghana or Grenada or Guam or Guatemala or Guinea or Guiana or Guyana or Haiti or Herzeg* or Hercegovina or Honduras or Hungary or India or Indonesia or Iran or Iraq or (Ivory adj Coast) or Jamaica or Jordan or Kazakh* or Kenya or Kiribati or North-Korea or Democratic-People's-Republic-of-Korea or Kosovo or (Kyrgyz adj Republic) or Kyrgyzstan or Kirghizia or Kirghiz or Kirgizstan or Laos or (Lao* adj2 Democratic adj Republic) or (Lao* adj PDR) or Latvia or Lebanon or Lesotho or Basutoland or Liberia or Libya or Lithuania or Macedonia or Madagascar or (Magalasy adj Republic) or Malawi or Malay* or Sabah or Sarawak or Maldives or Mali or (Marshall adj Island*) or Mauritania or Mauritius or (Agalega adj Island*) or Mexico or Micronesia or Moldov* or Mongolia or Montserrat or Montenegro or Morocco or Ifni or Mozambique or Myanma* or Namibia or Nauru or Nepal or (Netherlands adj Antilles) or (Dutch adj Antilles) or (New adj Guinea) or (New adj Caledonia) or Nicaragua or Niue or Niger or Nigeria or (Northern adj Mariana adj Island*) or Nyasaland or Oman or Pakistan or Palau or Panama or (Papua adj New adj Guinea) or PNG or Palestine or Paraguay or Peru or Philipines or Philippines or Phillipines or Phillippines or Poland or (Puerto adj Rico) or Yemen or Romania or Roumania or Rumania or Russia* or Rwanda or Ruanda or (Saint adj Kitts) or (St adj Kitts) or Nevis or (Saint adj Vincent) or (St adj Vincent) or Grenadines or Samoa* or (Navigator adj Island*) or (Saint adj Lucia) or (St adj Lucia) or (Saint adj Helena) or (St adj Helena) or (Sao adj Tome) or Senegal or Serbia or Seychelles or (Sierra adj Leone) or Slovak* or (South adj Africa) or (Solomon adj Island*) or Somalia or (Sri adj Lanka) or Ceylon or Sudan or Surinam* or Swaziland or Eswatini or Syria or Syrian-Arab-Republic or Tajikistan or Tadzhikistan or Tadjikistan or Tadzhik or Tanzania or Thailand or Tibet or Timor-Leste or Togo or (Togolese adj Republic) or Tokelau or Tonga or Trinidad or Tobago or Tunisia or Turkey or Turkmenistan or Turkmen or Tuvalu or Uganda or Ukraine or Uruguay or Urundi or USSR or (Soviet adj Union) or Union-of-Soviet-Socialist-Republic* or Uzbekistan or Vanuatu or (New adj Hebrides) or Venezuela or Vietnam or (Viet adj Nam) or (Wallis adj2 Futuna) or (United adj Arab adj Republic) or (West adj Bank) or (West adj Indies) or Yemen or Yugoslavia or Zaire or Zambia or Zimbabwe or Rhodesia).tw,kf.	1529152
16	(africa or americas or caribbean or (central adj America) or (latin adj America) or (south adj America) or (eastern adj Europe) or Transcaucasia or antarctic or (atlantic adj island*) or (indian adj ocean adj island*) or (pacific adj island*) or polynesia or (central adj asia) or (southeast* adj asia) or (south-east* adj asia) or borneo or mekong or (western adj asia) or (middle adj east) or (far adj east) or (eastern adj asia) or (southern adj asia)).tw,kf.	293678
17	5 or 6 or 7 or 8 or 9 or 10 or 11 or 12 or 13 or 14 or 15 or 16	4989195
18	(1 or 2) and 3 and 17	2189
19	4 and 17	14
20	18 or 19	2203
21	limit 20 to yr="2000 -Current"	2032
	Updated search 28/03/2024 additional records	224

Figure 5 PRISMA flow diagram for the oxygen service coverage and tools literature review

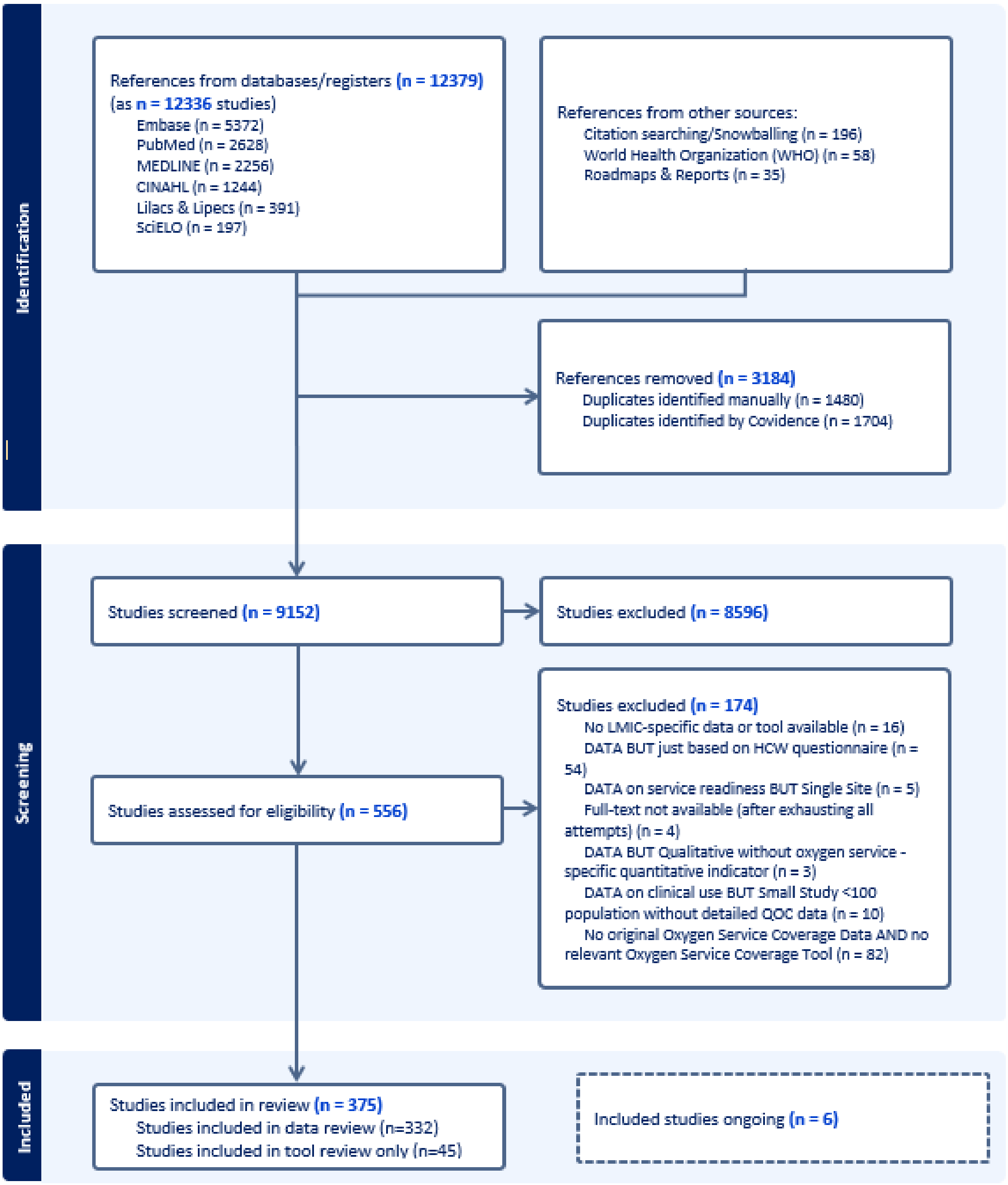

2.1.4 Care packages review

A targeted search of WHO, UNICEF and professional associations was done to find international guidelines for key conditions needing medical oxygen. This resulted in 15 guidelines for acute medical conditions, 2 for chronic conditions, and 1 for COVID-19. Each guideline was graded using a scoring criterion of: Fully Covered, Partially Covered, Not Covered, Not applicable.

Pulse oximetry:

- Lists the potential patients in need of medical oxygen service.
- Acknowledges or mentions considerations or needs unique to specific patient populations such as neonates, pregnant women, patients with chronic illness.
- Intentionally states the clinical indications including hypoxemia, signs and symptoms and underlying disease that may lead to hypoxemia.
- Mentions SpO_2 as a vital sign that should be measured or assessed.
- Guides on the use of pulse oximetry/ SpO_2 to screen, detect hypoxemia, stratify risk for severe illness and to monitor patient prognosis.
- Guides on the proper technique for using the pulse oximeter including placing and use of appropriate probes.
- Guides on how to interpret SpO_2 values and apply them to manage the patient.
- Guides on the healthcare setting where pulse oximetry should be provided and related aspects of context of use.

Oxygen therapy:

- Clearly states which patients need or deserve medical oxygen therapy and highlights the needs of specific patient groups.
- Guides on prescription of medical oxygen including flow rates, patient delivery interfaces and target oxygen saturation levels.
- Mentions monitoring of patients on oxygen therapy – either using pulse oximeters, blood gas analysis, target oxygen saturation levels, signs and symptoms.
- Cautions about potential oxygen toxicity and identifies patients at high risk of oxygen toxicity (neonates, pre-terms) and mentions strategies to prevent it.
- Guides on criteria of weaning the patient off medical oxygen or discontinuing oxygen therapy.
- Guides on some of the oxygen sources that can be used for the expected patients.
- Guides on the proper technique for using oxygen-related medical devices (e.g. bedside oxygen source, flow meter, regulator, alarms, patient delivery interfaces, including how to monitor their proper functioning.
- Guides on the healthcare setting where oxygen therapy should be provided and related aspects of context of use.

Integration of medical oxygen services with other care:

- Delineates the roles of the relevant healthcare providers including nurses, medical doctors, biomedical staff in oxygen therapy.
- Guides on infection control measures relevant for oxygen equipment and devices in the healthcare setting.
- Addresses integration of medical oxygen services with other care patient may require including treating the underlying cause of hypoxemia and referral to a specialized facility.

Presentation and accessibility

- Applies best practice such as use of infographics including flow charts, diagrams, and other illustrations to increase accessibility to diverse audiences.
- Presentation and general structure are user friendly, simple to flow and convenient for practice settings.

2.1.5 Cost of oxygen services review

Search: We conducted a rapid evidence review, using academic and grey literature, to summarise the evidence of cost-effectiveness and cost of oxygen. Academic literature was identified through a search of Web of Science and PubMeb, conducted on the 2nd February 2024, and then through a google scholar search on the 27th March 2024, with the first 100 hits extracted. A Google search was conducted on the 1st May 2024, and the first 100 hits screened for any additional sources. The search terms used were: “oxygen” AND “cost/cost-effectiv*/budget/economic/financ*”. We also searched the reference list of included articles, for any additional relevant publications.

Inclusion and exclusion criteria: We included all types of sources, including original research, reviews, commentaries, reports, position statements and policy documents, and had no restriction by date of publication. We excluded sources that related to wound care, ECMO and other advanced respiratory support, hyperbaric oxygen, CPAP for sleep apnoea, and non-medical uses of oxygen.

Article screening: Academic references were imported into Covidence for title and abstract screening, which was conducted by a single reviewer (CK). Following abstract screening, the remaining publications were full-text screened, and the process documented in Microsoft Excel. Full text access was based on open access through Karolinska Institutet’s library system.

Data extraction: Data were extracted into an Excel spreadsheet, including indicators for year, country, World Bank region, income level, setting, patient group, oxygen source, and oxygen costs (out-of-pocket costs to patient, cost to treat a patient, cost per litre/m^3/cylinder, capital and operational costs of oxygen systems, cost savings or effectiveness of oxygen interventions, and programmatic costs to providers). We did not use a quality assessment tool.

Data synthesis: Overall, 1780 articles were returned from the searches, with 1762 unique studies. After screening and including additional sources from google searches and snowballing, 73 articles were included – Figure 6. The papers were used to write a narrative about cost-effectiveness of oxygen and pulse oximetry for paediatric pneumonia, costs of oxygen systems, and estimate the cost to fill the oxygen need gap (elaborated in Section 2.2.3).

Figure 6: PRISMA flow diagram for the cost-estimate rapid evidence synthesis

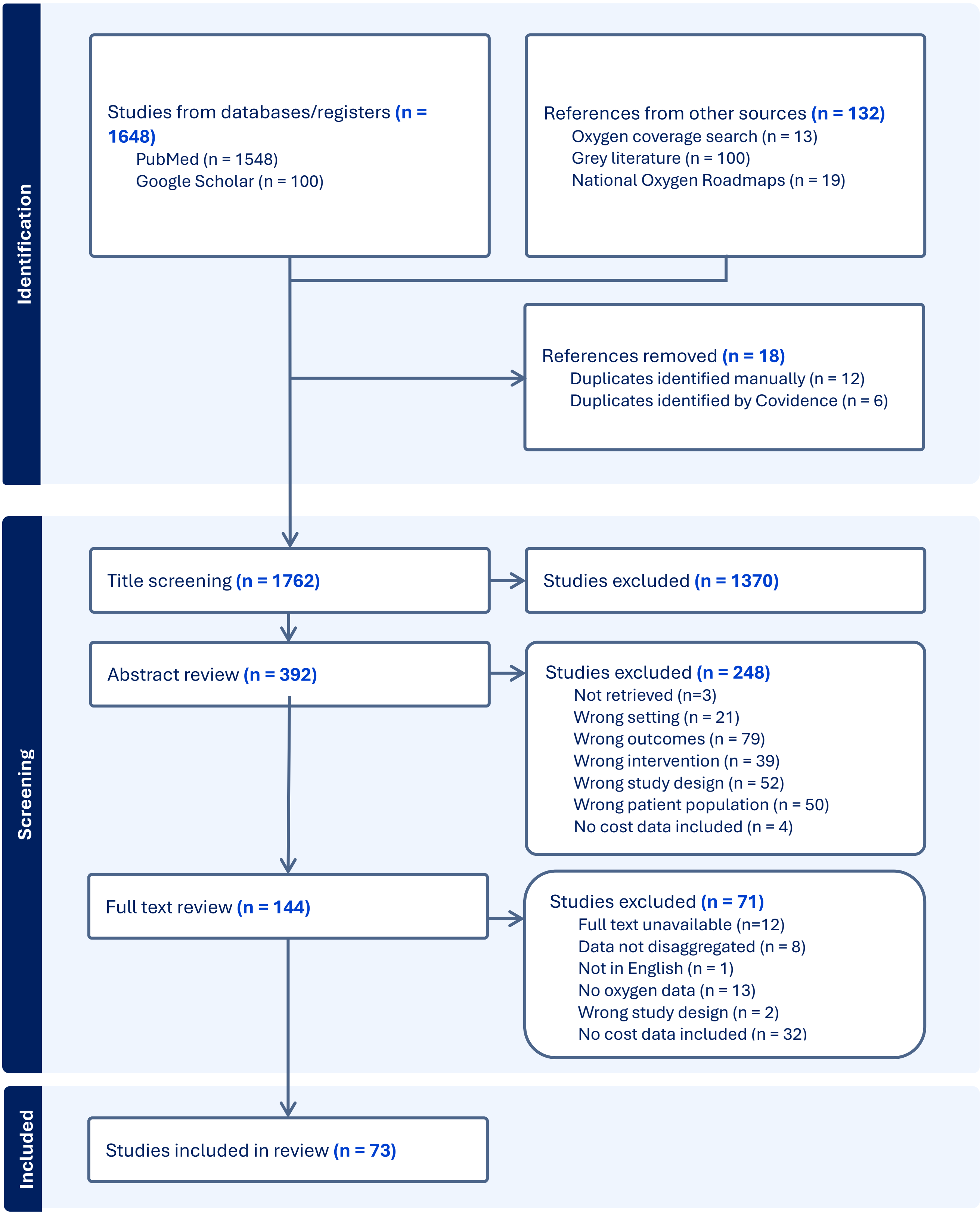

2.1.6 Role of skin tone in pulse oximetry readings review

A comprehensive meta-analysis conducted in 2021 underscored the challenge posed by skin pigmentation in accurate pulse oximetry measurements [16]. When compared to the gold standard measurement of arterial oxygen saturation (SaO_2) obtained via CO-oximetry, pulse oximetry tends to exhibit a systematic bias, particularly in individuals with high skin pigmentation. The pooled mean bias for this group is 1.1%, while specifically among Black/African American individuals, it rises to 1.5%. A subsequent systematic review in 2022 reaffirmed this discrepancy, emphasizing that pulse oximetry readings consistently overestimate oxygen saturation in individuals with darker skin [17]. This overestimation could have critical implications, potentially leading to undetected hypoxemia and associated health risks.

Search: To enhance our understanding, we updated the previous systematic reviews[16 17] by incorporating newer studies published between 2022 and 2023 (search conducted 28 September 2023). Our search included multiple databases, including Medline, Embase, PubMed, CINAHL, and Web of Science, with no language restrictions. Relevant search terms such as “oximetry,” “accuracy,” “overestimation,” and “underestimation” guided our exploration (Panel 3). Additionally, we included findings from two earlier systematic reviews on this topic, both published in 2022.

Analysis: Our analysis encompassed 33 studies, comprising 28 from previous reviews and five newly published studies. These investigations specifically examined the accuracy of peripheral oxygen saturation (SpO_2) measured using pulse oximeters equipped with either reflectance or transmissive probes, comparing them to SaO_2 values obtained via standard CO-oximetry. We considered a diverse range of participants, accounting for varying health complexities, geographical regions, and clinical settings. Key outcomes assessed included bias, precision, accuracy, and agreement. Our analysis was meticulously categorized into two primary dimensions: skin pigmentation (dark, medium, light) and ethnicity (African, Asian/Hispanic/mixed, white/Caucasian). Subsequently, separate meta-analyses were conducted for each subgroup. Regardless of the direction of bias (whether SpO2-SaO2 or SaO2-SpO2), we focused on the absolute mean bias difference.

This review yielded in five articles shown in the PRISMA flow diagram (Figure 7). Summary results below (Table 2) and full list of included studies in Table 3.

Table 2 Mean pulse oximetry bias by skin pigmentation and ethnicity

	Categories	Absolute Mean Bias Difference	Lower CI	Upper CI
**Skin Pigmentation**	Dark (S= 31, N= 8,894)	1.58	1.19	1.97
	Medium (S= 12, N= 11,489)	0.71	0.43	0.99
	Light (S= 26, N= 7,555)	0.79	0.51	1.07
**Ethnicity**	African (S= 26, N= 16,119)	1.99	1.49	2.48
	Asian, Hispanic, Mixed (S=17, N= 6,862)	1.16	0.67	1.65
	Caucasian (S=27, N=58,760)	0.97	0.68	1.25

Panel 3 OVID MEDLINE Search strategy of 'Influence of skin tone on pulse oximetry readings" systematic review

Line	Terms	Results
1	oximetry.mp. [mp=title, book title, abstract, original title, name of substance word, subject heading word, floating sub-heading word, keyword heading word, organism supplementary concept word, protocol supplementary concept word, rare disease supplementary concept word, unique identifier, synonyms, population supplementary concept word, anatomy supplementary concept word]	20493
2	exp Oximetry/	16739
3	(oximet* or oxymet*).ti,ab,kw.	18930
4	(SpO2 or %spo2 or sp o2).tw.	8070
5	2 or 3 or 4	31921
6	(co-oximet* or co-oxymet* or h?emoximet*).ti,ab,kw.	855
7	(blood adj3 (analys* or measure*)).tw.	119958
8	(blood sampl* or gold standard or reference device* or reference instrument* or in-line oximet* or in vitro oximet* or arterial oxygen saturation or arterial oxyhemoglobin saturation or arterial oxyhaemoglobin saturation or arterial blood or arterial puncture or SaO2 or %SaO2 or sa o2).tw.	331459
9	6 or 7 or 8	426217
10	Reproducibility of Results/	465554
11	Validation Study/	109229
12	Evaluation Studies as Topic/	122473
13	Bias/	26792
14	"Sensitivity and Specificity"/	369458
15	Hypoxia/di [Diagnosis]	2418
16	comparative study.pt.	1913099
17	(accura* or inaccura* or overestimat* or over-estimat* or underestimat* or under-estimat* or agreement or root-mean-square or root mean square or RMS or quadratic mean).tw.	1480376
18	(precision or evaluat* or predict* or reliab* or reproducib* or concordance or performance or bias or validat* or error* or erroneous or individual variability or (variability and (analysis or values)) or sensitivity or specificity or failure).tw.	9275801
19	(compar* adj3 (measure* or value*)).tw.	164286
20	(controlled desaturation or paired repeated measure* or method comparison or calibration stud*).ti,ab,kw.	3021
21	(paired readings or paired measurements or "difference of values" or "limits of agreement" or "limits of values" or confidence limits or regression or bland altman).ti,ab,kw.	1075980
22	10 or 11 or 12 or 13 or 14 or 15 or 16 or 17 or 18 or 19 or 20 or 21	11573572
23	5 and 9 and 22	4537
24	limit 23 to (english language and humans and yr="2022 -Current")	196

Figure 7 PRISMA flowchart of effect of skin tone on pulse oximetry readings systematic review

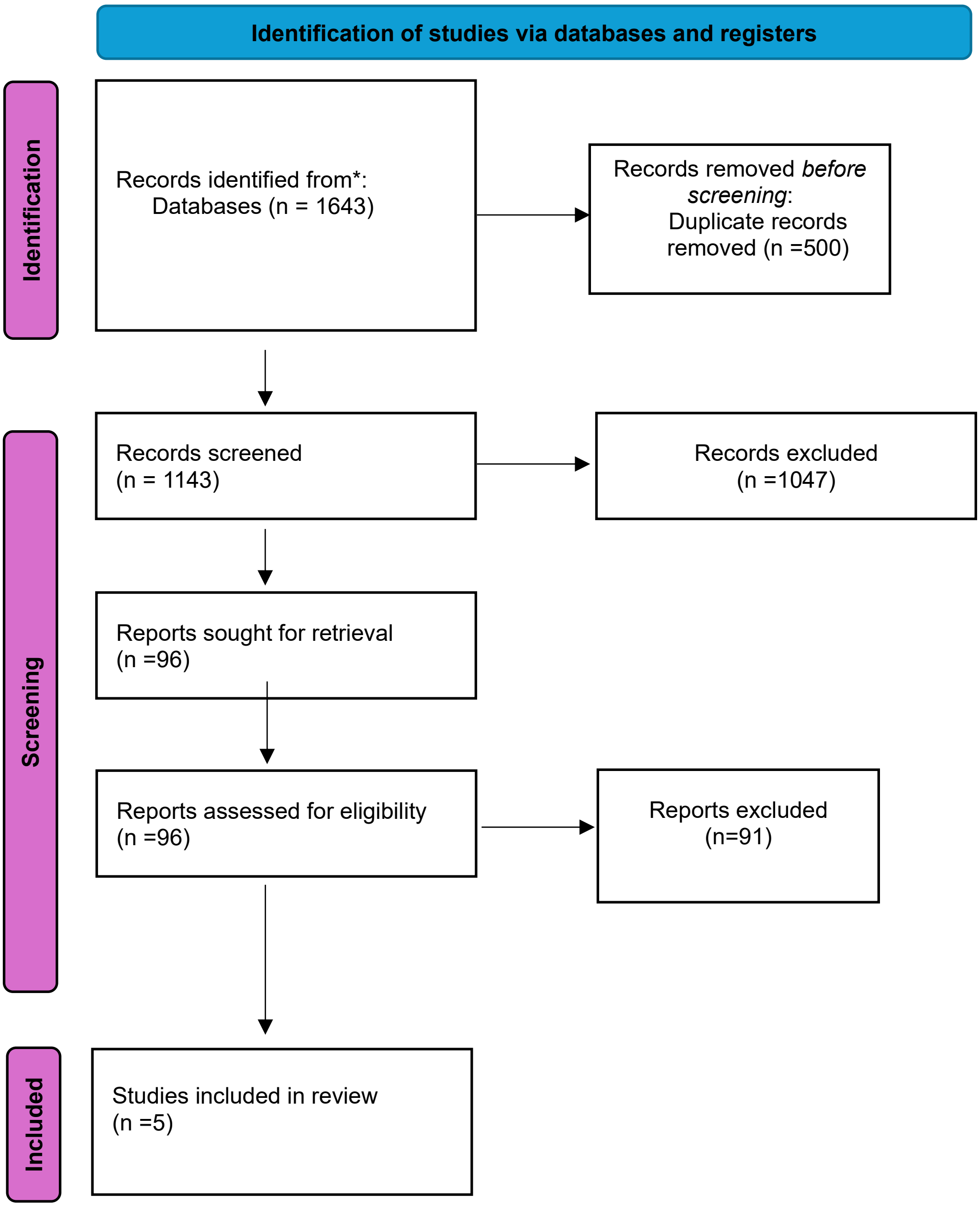

Table 3 Included studies in estimating skin pigmentation bias with absolute value of bias

Source	First Author Last Name	Publication year	Study Title	Study Settings (Region)	Disaggregated Bias Reported for Skin Pigmentation	Dark Skin Pigmentation	Medium Skin Pigmentation	Light Skin Pigmentation	Disaggregated Bias Reported for Ethnicity?	African/ Black	Asian/Hispanic/ Mixed	White/ Caucasian
New	**Ajizan**	2023	OCCULT HYPOXEMIA AND PULSE OXIMETRY PERFORMANCE ACROSS SKIN PIGMENTATION GROUPS	North America	Yes	0.3		0.05	No			
New	**Blanchet**	2023	Accuracy of Multiple Pulse Oximeters in Stable Critically Ill Patients	North America	Yes	1.3,1, 3.3, 0.8		3.1, 0.3, 0.2, 0.9	No			
New	**Fawzy**	2022	Racial and Ethnic Discrepancy in Pulse Oximentry and Delayed Identification of Treatment Eligibility Among Patients With COVID-19	North America	No				Yes	3.4, 3.1, 2.4	1.3, 3.6, 2.9, 4.2, 2.2, 2.3	1.9,1.6, 1.2
New	**Gudelunas**	2022	Low perfusion and missed diagnosis of hypoxemia by pulse oximetry in darkly pigmented skin: a prosective study	North America	Yes	2.1, 1.9	0.6, 1	0.1, 0.9	No			
Previous review	**Henry**	2022	Disparities in hypoxemia detection by pulse oximetry across self-identified racial groups and associations with clinical outcomes	North America	No				Yes		0.43	0.68

Source	First Author Last Name	Publication year	Study Title	Study Settings (Region)	Disaggregated Bias Reported for Skin Pigmentation	Dark Skin Pigmentation	Medium Skin Pigmentation	Light Skin Pigmentation	Disaggregated Bias Reported for Ethnicity?	African/ Black	Asian/Hispanic/ Mixed	White/ Caucasian
New	**Sudat**	2022	Racial disparities in pulse oximeter device inaccuracy and estimated clinical impact on COVID 19 treatment course	North America	No				Yes	2.45		1.53
Previous review	**Wiles**	2022	Effect of patient ethnicity on the accuracy of peripheral pulse oximetry in patients with COVID-19 pneumonitis requiring mechanical ventilation	Europe	No				Yes	1.72	0.96	0.25
Previous review	**Harskamp**	2021	Performance of popular pulse oximeters compared with simultaneous arterial oxygen saturation or clinical-grade pulse oximetry: a cross-sectional validation study in intensive care patients	Europe	No				No			
Previous review	**Vesoulis**	2021	Racial discrepency in pulse oximeter accuracy in preterm infants	North America	No				Yes	1.73		0.72
Previous review	**Brooks**	2020	Transcutaneous oxygen saturation accuracy in critically ill children	Ocenia	No				No			

Source	First Author Last Name	Publication year	Study Title	Study Settings (Region)	Disaggregated Bias Reported for Skin Pigmentation	Dark Skin Pigmentation	Medium Skin Pigmentation	Light Skin Pigmentation	Disaggregated Bias Reported for Ethnicity?	African/ Black	Asian/Hispanic/ Mixed	White/ Caucasian
Previous review	**Pilcher**	2020	A multicentre prospective observational study comparing arterial blood gas values to those obtained by pulse oximeters used in adult patients attending Australian and New Zealand hospitals	Ocenia	Yes	0.1	1.1	1.2	No			
Previous review	**Valbuena**	2020	Racial bias in pulse oximetry measurement among patients about to undergo extracorporeal membrane oxygenation in 2019-2020	North America	No				Yes	1.7	0.3,0.8	0.3
Previous review	**Ebmeier**	2018	A two centre observational study of simultaneous pulse oximetry and arterial oxygen saturation recordings in intensive care unit patients	Ocenia	Yes	2.24, 1.1	0.45, 0.11	0.17, 1.07	No			
Previous review	**Schallom**	2018	Comparison of nasal and forehead oximetry accuracy and pressure injury in critically ill patients	North America	No				No			

Source	First Author Last Name	Publication year	Study Title	Study Settings (Region)	Disaggregated Bias Reported for Skin Pigmentation	Dark Skin Pigmentation	Medium Skin Pigmentation	Light Skin Pigmentation	Disaggregated Bias Reported for Ethnicity?	African/ Black	Asian/Hispanic/ Mixed	White/ Caucasian
Previous review	**Foglia**	2017	The effect of skin pigmentation on the accuracy of pulse oximetry in infants with hypoxemia	North America	Yes	1.6, 5.4		0.2, 3	No			
Previous review	**Harris**	2016	Accuracy of Pulse Oximeters Intended for Hypoxemic Pediatric Patients	North America	No				No			
Previous review	**Ross**	2014	Accuracy of Pulse Oximetry in Children	North America	No				No			
Previous review	**Harris**	2009	Accuracy of a portable pulse oximeter in monitoring hypoxemic infants with cyanotic heart disease	North America	No				No			
Previous review	**Munoz**	2008	Accuracy and reliability of pulse oximetry at different arterial carbon dioxide pressure levels	Europe	No				Yes			0.47
Previous review	**Hinkelbein**	2007	Artificial acrylic finger nails may alter pulse oximetry measurement	Europe	Yes			0.7,0.6,1.1,0.8	Yes			0.7,0.6, 1.1,0.8
Previous review	**Feiner**	2007	Dark skin decreases the accuracy of pulse oximeters at low oxygen saturation: the effects of oximeter probe type and gender	North America	Yes	2.14, 2.04, 0.63, 0.24, 2.13, 1.26	0.99, 0.64, 0.03, 0.34, 0.83, 0.51	0.21, 0.36, 0.95, 0.64, 1.04, 0.52	Yes	2.14, 2.04, 0.63, 0.24, 2.13, 1.26	0.99, 0.64, 0.03, 0.34, 0.83, 0.51	0.21, 0.36, 0.95, 0.64, 1.04, 0.52

Source	First Author Last Name	Publication year	Study Title	Study Settings (Region)	Disaggregated Bias Reported for Skin Pigmentation	Dark Skin Pigmentation	Medium Skin Pigmentation	Light Skin Pigmentation	Disaggregated Bias Reported for Ethnicity?	African/ Black	Asian/Hispanic/ Mixed	White/ Caucasian
Previous review	**Bickler**	2005	Effects of skin pigmentation on pulse oximeter accuracy at low saturation	North America	Yes	0.15, 1.83, 1.29		0.21, 1.15, 0.27	Yes	0.15, 1.83, 1.29		0.21, 1.15, 0.27
Previous review	**Abrams**	2002	Utility of pulse oximetry in the detection of arterial hypoxemia in liver transplant candidates	North America	No				Yes	3.6		3.4
Previous review	**Adler**	1998	Effect of skin pigmentation on pulse oximetry accuracy in the emergency department	North America	Yes	2.2	2.8	2.5	No			
Previous review	**Gabrielczyk**	1988	Pulse oximetry and post operative hypothermia	Europe	No				No			
Previous review	**Avant**	1997	Comparison of accuracy and signal consistency of two reusable pulse oximeter probes in critically ill children	North America	No				Yes	1.35, 1.05		1.45, 1.21
Previous review	**Bothma**	1996	Accuracy of pulse oximetry in pigmented patients	Africa	Yes	1.2, 0.8, 0.6, 1			No			
Previous review	**McGovern**	1996	Comparison of oxygen saturation by pulse oximetry and co-oximetry during exercise testing in patients with COPD	North America	No				Yes			1.7

Source	First Author Last Name	Publication year	Study Title	Study Settings (Region)	Disaggregated Bias Reported for Skin Pigmentation	Dark Skin Pigmentation	Medium Skin Pigmentation	Light Skin Pigmentation	Disaggregated Bias Reported for Ethnicity?	African/ Black	Asian/Hispanic/ Mixed	White/ Caucasian
Previous review	**Lee**	1993	Factors influencing pulse oximetry as compared to functional arterial saturation in multi-ethnic Singapore	Asia	No				Yes		0.82	
Previous review	**Stewart**	1991	Inaccuracy of pulse oximetry in patients with severe tricuspid regurgitation	Asia	No				No			
Previous review	**Zeballos**	1991	Reliability of noninvasive oximetry in black subjects during exercise and hypoxia	North America	Yes	0.4, 2.1, 0.8, 3.5, 4.8, 9.8			Yes	0.4, 2.1, 0.8, 3.5, 4.8, 9.8		
Previous review	**Jubran**	1990	Reliability of pulse oximetry in titrating supplemental oxygen therapy in ventilator-dependant patients	North America	No				Yes	3.3		2.2
Previous review	**Emery**	1987	Skin pigmentation as an influence on the accuracy of pulse oximetry	North America	No				No			

*Some studies show no data as they reported overall bias instead of skin pigmentation/ethnicity disaggregated bias

2.1.7 Barriers and facilitators of implementing medical oxygen therapy solutions

Search: We conducted a comprehensive literature search focused on determinants of implementation outcomes of medical oxygen therapy solutions in two major biomedical databases: EMBASE and MEDLINE (PubMed). These database searches were executed using the EMBASE search engine by Elsevier. Furthermore, we scrutinized the reference lists of selected studies and relevant reviews to identify any additional articles that met the eligibility criteria. We developed a search strategy following an extensive literature review and consultations with both an expert librarian and a search strategist, as well as specialists in the field of medical oxygen solutions. To guarantee thorough coverage, we identified four key search concepts: ‘medical oxygen solutions’, ‘health worker’, ‘health worker-related factors’, and ‘work environment factors.’ The specific search terms corresponding to each of these concepts have been meticulously compiled and are detailed in Table 4**.**

Table 4 - Search strategy for barriers and facilitators review

Domain	Search strategy
Medical oxygen solutions	'oxygen therapy'/exp OR 'oxygen therapy' OR 'oxygen' OR 'pulse oximetry' OR 'medical oxygen' OR 'hypoxemia*' OR 'hypoxaemia*' OR 'oxygen saturation monitoring' OR 'oxygen treatment' OR 'oxygen supply' OR 'oxygen solution*' OR 'hypoxia'
Health worker	'health care personnel' OR 'healthcare personnel' OR 'health worker*' OR 'healthcare worker*' OR 'health workforce'/exp OR 'health workforce' OR 'healthcare professional*' OR 'clinician*' OR 'nurse*' OR 'dentis' OR 'dental surgeon' OR 'paramedic*' OR 'medical student*' OR 'health profession student*' OR 'medical trainee' OR 'dental student*' OR 'medical undergraduate' OR 'pharmacy student*' OR 'nursing student*' OR 'doctor*' OR 'physician'/exp OR 'physician' OR 'internist'/exp OR 'internist' OR 'resident'/exp OR 'resident' OR 'pharmacist*' OR 'biomedical*' OR 'anaesthesiologist'/exp OR 'anaesthesiologist' OR 'anesthesiologist'/exp OR 'anesthesiologist' OR 'midwi*' OR 'health staff' OR 'medical personnel'/exp OR 'medical personnel' OR 'medical officer*' OR 'healthcare provider*' OR 'medical practitioner*' OR 'healthcare team' OR 'health personnel'/exp OR 'health personnel' OR 'health care professional'/exp OR 'health care professional' OR 'clinical medicine'/exp OR 'clinical medicine'
Health worker factors	'knowledge' OR 'awareness' OR 'skill*' OR 'expertise' OR 'proficiency' OR 'attitudes' OR 'beliefs' OR 'perception*' OR 'competency' OR 'qualification' OR 'motivation' OR 'experience*' OR 'motivator*' OR 'drive' OR 'incentive' OR 'capability' OR 'capacity' OR 'behaviour' OR 'opportunity' OR 'efficacy' OR 'barrier*' OR 'practice*' OR 'enabler*' OR 'facilitate*' OR 'continuing professional education' OR 'continuing medical education'
Work environment factors	'work environment' OR 'healthcare setting' OR 'clinical environment' OR 'health facility' OR 'hospital' OR 'clinic' OR 'staffing' OR 'ambulance' OR 'skill mix' OR 'workforce retention' OR 'job turnover' OR 'workload' OR 'job demand' OR 'healthcare readiness' OR 'facility readiness' OR 'infrastructure' OR 'treatment guidelines' OR 'clinical protocols' OR 'patient record systems' OR 'health management*' OR 'recognition systems' OR 'reward systems'

Inclusion and exclusion criteria: We included primary studies or original research that reported implementation outcomes of medical oxygen solutions. The main implementation outcomes assessed were adoption, reach, fidelity, scale up, implementation costs, maintenance or their equivalent or intermediates. The intermediate outcomes included acceptability, appropriateness, feasibility, effectiveness, healthcare worker intermediate implementation outcomes (knowledge, awareness, attitudes, beliefs, competence, perceptions and skills), health facility intermediate implementation

outcomes such as availability and use of medical equipment and accessories and restricted inclusion to the year 2010 onwards.

Article screening: The search results were imported into *Covidence* software, a research tool designed for systematic reviews and meta-analyses, for screening titles, abstracts, full texts, and data extraction [21]. At every stage of screening, whether title, abstract or full text, each article was screened independently by at least two reviewers for inclusion or exclusion. Any conflicts in voting on an article were addressed through reviewer team deliberations and discussions to reach consensus. Full text access for pay walled articles was obtained through library systems at Makerere University, Uganda University of Uppsala, Sweden and Imperial College London, United Kingdom.

Data extraction: We used the COVIDENCE software data extraction function to abstract the following variables from each article. Study setting (country where study was done, study unity, context) Study design (study participants, level of care, sample size, individual study eligibility criteria, intervention studied, study variables), study findings (background characteristics, implementation outcomes or intermediate outcomes or equivalent on health workers, health facility or work environment, barriers and facilitators and determinants, study limitations and conclusions). After data extraction, the data set was downloaded from the *Covidence* software as a Microsoft Excel spreadsheet.

Data management, analysis and synthesis: Data was cleaned in the Microsoft Excel spreadsheet and missing data was retrieved from the text of original articles. Qualitative analysis was done by thematic analysis and grouping into categories. Quantitative data was qualitized and synthesized into barriers or facilitators by category. A detailed account of the systematic review process is available in a systematic review protocol manuscript (Kitutu et al) under review.

Figure 8 PRISMA inclusion for studies in the enables and barriers systematic review

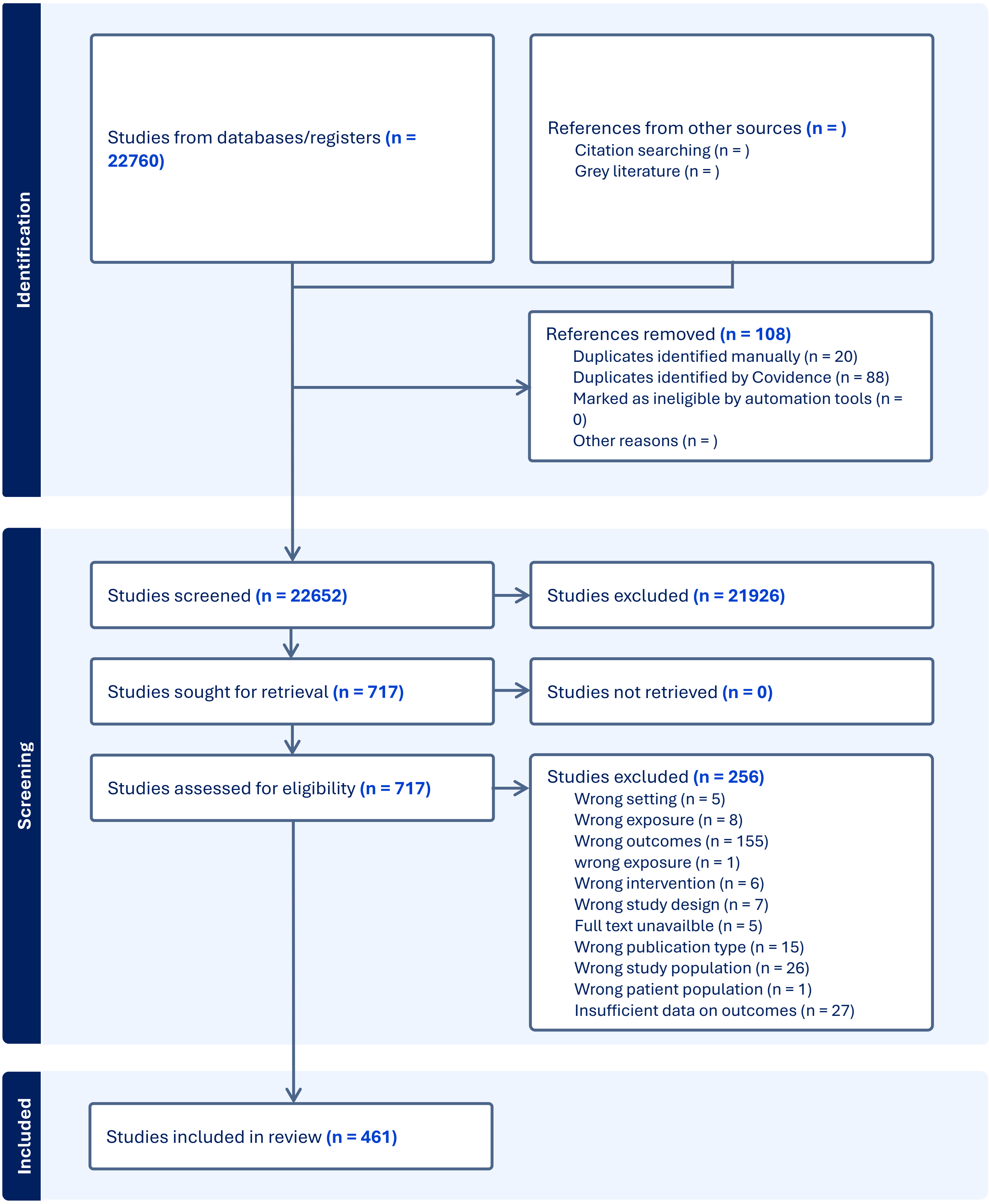

2.2 Needs, Coverage, Costing the Gap estimation

2.2.1 Estimating global oxygen need

To estimate global oxygen needs, we categorized them into two broad categories:

1) Oxygen needs in acute conditions
   a) acute medical conditions
   b) peri-operative period of major surgeries
2) Long-term oxygen therapy in chronic conditions

2.2.1a. Oxygen needs in acute medical conditions

We used the following equation to estimate the yearly oxygen needs.

> **Oxygen need (number of people requiring oxygen therapy) in acute medical conditions = disease burden of acute medical conditions (number) * hospitalization need (%) * hypoxaemia proportion (%) * inflation factors**

We adopted the following steps to estimate the yearly oxygen needs based on the above-mentioned equations.

Step 1: Identify acute medical conditions with oxygen needs

A systematic review was conducted to determine the proportion of hypoxemia in patients admitted to healthcare facilities in low-and-middle-income countries.[9] This review identified 14 conditions/diseases with meta-estimates of hypoxemia proportions: asthma, COVID-19, diarrhoea, encephalitis, HIV/AIDS, lower respiratory infections, malaria, neonatal complications (encephalopathy/birth asphyxia, respiratory infections, sepsis, preterm births), nutritional deficiencies, and trauma/injury. Following consultations with relevant commissioners and advisors as experts, six additional conditions/diseases were identified as frequently or intermittently requiring medical oxygen therapy: cardiovascular diseases, tuberculosis, dengue, measles, pertussis, and typhoid. We considered these 20 conditions/diseases as frequently/occasionally requiring medical oxygen therapy.

These conditions/diseases were aligned with the Global Burden of Disease (GBD) categories (Table 5), except for sepsis. GBD does not consider sepsis as a distinct diseases/condition in children and adults as it is the final common pathway to death due to various infectious diseases and NCDs. We assumed that the sepsis associated with infectious diseases would be accounted for within acute medical conditions identified through the systematic review and expert consultations. However, we included *sepsis (not otherwise classified)* in the list of acute medical conditions to represent sepsis association with non-communicable diseases (NCDs) and other inflammatory conditions.

Conditions/diseases that infrequently/rarely require oxygen therapy were collectively categorized as 'other diseases' outside the list of 20 conditions/diseases identified through the systematic review and expert consultation.

Table 5 Acute medical conditions requiring medical oxygen therapy

sl	Condition/disease	GBD-ID	GBD-Level	GBD terminology (selected category in bold)
1	Asthma	515	Level 3	Level 2: Chronic Respiratory Diseases<br>**Level 3:** Chronic obstructive pulmonary disease, pneumoconiosis, **Asthma (selected)**, Interstitial Lung Disease and Pulmonary Sarcoidosis, Other Chronic Respiratory Diseases<br>Level 4: No relevant disease of the selected level 3 disease
2	Cardiovascular disease (CVD)	491	Level 2	**Level 2: Cardiovascular diseases**<br>Level 3: Rheumatic heart disease, stroke, atrial fibrillation and flutter, endocarditis, pulmonary arterial hypertension, ischemic heart disease, cardiomyopathy and myocarditis, lower extremity peripheral arterial disease, non-rheumatic valvular heart disease
3	COVID 19	1048	Level 3	Level 2: Respiratory infections and tuberculosis<br>**Level 3:** Lower respiratory infections, Upper respiratory infections, Otitis media, **COVID 19 (selected)**, and Tuberculosis<br>Level 4: No relevant disease of the selected level 3 disease
4	Dengue	357	Level 3	Level 2: Neglected tropical diseases and malaria<br>**Level 3:** Malaria, chagas disease, leishmaniasis, African trypanosomiasis, schistosomiasis, cysticercosis, cystic echinococcosis, lymphatic filariasis, onchocerciasis, trachoma, **dengue (selected)**, yellow fever, rabies, intestinal nematode infections, food-borne trematodiases, leprosy, ebola, zika virus, guinea worm disease, other neglected tropical diseases<br>Level 4: No relevant disease of the selected level 3 disease
5	Diarrhoea	302	Level 3	Level 2: Enteric infections<br>**Level 3: Diarrheal diseases (selected)**, typhoid and paratyphoid, invasive non-typhoidal salmonella (iNTS), other intestinal infectious diseases<br>Level 4: No relevant disease of the selected level 3 disease
6	Encephalitis	337	Level 3	Level 2: Other infectious diseases<br>**Level 3:** Meningitis, **encephalitis (selected)**, diptheria, whooping cough, tetanus, measles, varicella and herpes zoster, acute hepatitis, other unspecified infectious diseases<br>Level 4: No relevant disease of the selected level 3 disease
7	HIV/AIDS	298	Level 3	Level 2: HIV/AIDS and sexually transmitted infections<br>**Level 3: HIV/AIDS (selected),** Sexually transmitted infections excluding HIV<br>Level 4: HIV/AIDS - Extensively drug-resistant Tuberculosis, HIV/AIDS - Multidrug-resistant

sl	Condition/disease	GBD-ID	GBD-Level	GBD terminology (selected category in bold)
				Tuberculosis without extensive drug resistance, HIV/AIDS - Drug-susceptible Tuberculosis, HIV/AIDS resulting in other diseases
8	Lower respiratory infection (LRI) / pneumonia	322	Level 3	Level 2: Respiratory infections and tuberculosis<br>**Level 3: Lower respiratory infections (selected),** Upper respiratory infections, Otitis media, COVID 19 and Tuberculosis<br>Level 4: No relevant disease of the selected level 3 disease
9	Malaria	345	Level 3	Level 2: Neglected tropical diseases and malaria<br>**Level 3: Malaria (selected),** chagas disease, leishmaniasis, African trypanosomiasis, schistosomiasis, cysticercosis, cystic echinococcosis, lymphatic filariasis, onchocerciasis, trachoma, dengue, yellow fever, rabies, intestinal nematode infections, food-borne trematodiases, leprosy, ebola, zika virus, guinea worm disease, other neglected tropical diseases<br>Level 4: No relevant disease of the selected level 3 disease
10	Measles and pertussis	341, 339	Level 3	Level 2: Other infectious diseases<br>**Level 3:** Meningitis, encephalitis, diptheria, **pertussis (selected)**, tetanus, **measles (selected)**, varicella and herpes zoster, acute hepatitis, other unspecified infectious diseases<br>Level 4: No relevant disease of the selected level 3 disease
11	Meningitis	332	Level 3	Level 2: Other infectious diseases<br>**Level 3: Meningitis (selected)**, encephalitis, diptheria, whooping cough, tetanus, measles (selected), varicella and herpes zoster, acute hepatitis, other unspecified infectious diseases<br>Level 4: No relevant disease of the selected level 3 disease
12	Neonatal encephalopathy/birth asphyxia	382	Level 4	Level 3: Neonatal Disorders<br>**Level 4:** Neonatal preterm birth, **Neonatal encephalopathy due to birth asphyxia and trauma (selected)**, Neonatal Sepsis and Other Neonatal Infections, Hemolytic Disease and Other Neonatal Jaundice, Other Neonatal Disorders
13	Neonatal lower respiratory infection	322	Level 3	Level 2: Respiratory infections and tuberculosis<br>**Level 3: Lower respiratory infections (selected),** Upper respiratory infections, Otitis media, COVID 19 and Tuberculosis<br>Level 4: No relevant disease of the selected level 3 disease
14	Neonatal preterm birth	381	Level 4	Level 3: Neonatal Disorders<br>**Level 4: Neonatal preterm birth (selected)**, Neonatal encephalopathy due to birth asphyxia and

sl	Condition/disease	GBD-ID	GBD-Level	GBD terminology (selected category in bold)
				trauma, Neonatal Sepsis and Other Neonatal Infections, Hemolytic Disease and Other Neonatal Jaundice, Other Neonatal Disorders
15	Neonatal sepsis	383	Level 4	Level 3: Neonatal Disorders<br>**Level 4:** Neonatal preterm birth, Neonatal encephalopathy due to birth asphyxia and trauma, **Neonatal Sepsis and Other Neonatal Infections,** Hemolytic Disease and Other Neonatal Jaundice, Other Neonatal Disorders
16	Nutritional deficiencies	386	Level 2	**Level 2: Nutritional deficiencies (selected)**<br>Level 3: Protein-energy malnutrition, iodine deficiency, Vitamin A deficiency, dietary iron deficiency, other nutritional deficiencies<br>Level 4: No relevant disease of the selected level 3 disease
17	Trauma/Injury	687	Level 1	**Level 1: Injuries (selected)**<br>Level 2: Transport injuries, unintentional injuries, self-harm and interpersonal violence
18	Tuberculosis	934, 946, 947	Level 4	Level 2: Respiratory infections and tuberculosis<br>Level 3: Lower respiratory infections, Upper respiratory infections, Otitis media, COVID 19 and Tuberculosis<br>**Level 4:** Latent tuberculosis infection, **drug-susceptible tuberculosis (selected), multidrug-resistant tuberculosis without extensive drug resistance (selected), extensively drug-resistant tuberculosis (selected)**
19	Typhoid	958	Level 3	Level 2: Enteric infections<br>**Level 3:** Diarrheal diseases, **typhoid and paratyphoid (selected)**, invasive non-typhoidal salmonella (iNTS), other intestinal infectious diseases<br>Level 4: Typhoid fever, paratyphoid fever
20	Sepsis (not otherwise classified)	NA	NA	Sepsis associated with non-communicable diseases (NCD)
21	Other diseases	NA	NA	All other GBD categories except conditions/diseases identified through systematic review and expert consultations (serial 1-19 of this table)

Step 2: Identifying the disease burden of acute medical conditions

To quantify the burden of acute medical conditions identified in the previous step, we utilized GBD data for 2010, 2013, 2016, 2019, and 2021. For sixteen conditions, including COVID-19, dengue, diarrhoea, encephalitis, lower respiratory infections, malaria, measles, pertussis, meningitis, neonatal encephalopathy due to birth asphyxia/trauma, neonatal preterm birth, neonatal lower respiratory infections, nutritional deficiencies, neonatal sepsis, trauma/injury, typhoid, we determined their burden using incidence estimates, which reflect the number of new cases occurring within a given time frame. GBD ascribe a single aetiology to every incident case, so each illness episode provides one data point (i.e. no double counting for comorbidity). For four other conditions, including HIV/AIDS, cardiovascular diseases (CVD), tuberculosis, and asthma, prevalence estimates were used instead to indicate the total number of existing cases at a given time. Detailed identifiers and levels for each condition/disease are listed in Table 5. Summary results in Table 6.

We categorized the disease burden across the following age groups: neonates (less than 28 days old), children (under 5 years and between 5 to 14 years), and adults (15 to 54 years and 55 years or older). We extracted data for global, regional and country-specific burden of each of the acute medical conditions for each year and age group mentioned above. The regions are defined according to the World Bank's classification: East Asia & Pacific, Europe & Central Asia, Latin America & Caribbean, Middle East & North Africa, North America, South Asia, and Sub-Saharan Africa.

Since GBD does not classify sepsis as a separate disease category, we referred to Rudd et al. (2020) for an age-standardized incidence rate of sepsis related to non-communicable diseases (186 per 100,000 population per year, table 1, page 202) [18]. Using this rate along with GBD's population figures for the specified years and age groups allowed us to estimate incidence rates specific to global, regional, and country levels.

To estimate the burden of "other diseases," we first calculated the total burden of the acute medical conditions listed from 1 to 20 in Table 5. We then subtracted this figure from the total disease burden reported by GBD.

Table 6 Disease burden of acute medical conditions

Disease/ Condition	Age group	Incidence	Prevalence	Deaths
Asthma	<5 years	11,709,792	29,428,788	4,746
	5-14 years	11,542,597	66,292,398	3,463
	15-54 years	9,548,480	103,068,738	76,942
	55+ years	5,063,307	61,689,263	351,042
CVD	15-54 years	14,967,779	155,619,403	2,015,018
	55+ years	49,980,365	443,749,017	17,348,107
COVID	<5years	119,589,049	6,247,821	16,715
	5-14years	463,890,633	23,972,076	25,797
	15-54years	1,368,706,468	1,368,706,468	1,548,610
	55+years	327,531,618	327,531,618	6,296,431
Dengue	<5years	3,472,146	207,066	5,664
	5-14years	12,408,448	740,168	3,243
	15-54years	33,691,305	2,009,907	9,657
	55+years	9,392,287	560,243	10,512
Diarrhoea	<5years	392,778,890	5,825,270	340,429
	5-14years	1,294,505,464	19,364,959	33,816
	15-54years	2,107,369,224	32,182,740	133,054
	55+years	643,923,697	9,903,432	658,098
Encephalitis	<5years	309,231	97,005	19,993
	5-14years	349,179	563,918	7,987
	15-54years	524,062	2,914,671	22,272
	55+years	310,297	1,067,970	41,696
HIV/AIDS	<5years	101,274	294,368	20,594
	5-14years	-	999,590	19,038
	15-54	1,433,576	32,278,641	568,199
	55+ years	110,482	6,464,338	110,248
Lower respiratory infections	<5years	37,828,159	832,724	501,909
	5-14years	32,072,328	743,237	43,764
	15-54	107,809,244	2,366,991	212,428
	55+years	165,897,056	3,626,626	1,424,900
Malaria	<5years	96,303,379	31,749,268	424,386
	5-14years	72,748,881	57,462,343	45,495
	15-54years	76,517,872	76,294,148	157,472
	55+years	3,546,497	8,379,811	120,778
Measles and Pertussis	<5years	10,089,803	935,008	92,619
	5-14years	1,672,029	159,429	13,483
Meningitis	<5years	1,015,606	461,911	91,147
	5-14years	317,128	1,319,240	21,225
	15-54years	569,836	4,251,456	55,898
	55+year	362,287	1,241,040	45,691
Neonatal Encephalopathy/Birth asphyxia	<28 days	1,061,448	50,891	568,255
Neonatal LRI	<28 days	1,436,527	18,974	152,087
Neonatal preterm birth	<28 days	21,553,771	1,511,647	666,803
Neonatal sepsis	<5years	3,880,834	1,370,050	232,656
Nutritional deficiencies	<5years	120,055,302	273,296,047	76,795
	5-14years	111,563,670	303,449,202	7,030
Trauma/injury	<5years	38,735,921	7,994,636	199,926

Disease/ Condition	Age group	Incidence	Prevalence	Deaths
	5-14years	93,833,380	66,411,828	187,402
	15-54years	356,876,393	846,543,514	2,217,408
	55+years	118,343,909	535,400,441	1,738,962
Tuberculosis	<5years	342,720	892,672	55,167
	5-14years	416,581	723,568	15,491
	15-54years	4,981,513	7,968,364	440,562
	55+years	2,666,320	4,908,569	651,575
Typhoid	<5years	2,919,569	174,186	42,945
	5-14years	2,720,708	160,083	28,256
	15-54years	3,057,829	176,998	30,364
	55+years	622,513	35,995	5,895
Sepsis (not otherwise classified)*	<5years	1,224,199	NA	NA
	5-14years	2,517,863	NA	NA
	15-54years	8,171,921	NA	NA
	55+years	2,763,934	NA	NA
Other diseases	<5years	3,049,436,913	213,367,671	2,537,600
	5-14years	5,189,542,991	691,766,203	203,905
	15-54years	15,319,372,255	1,698,789,905	4,288,441
	55+years	4,912,172,233	80,708,693	21,968,126

Step 3: Estimating the need for hospitalization for acute medical conditions

There is some evidence regarding the current practice of hospitalization rates for acute medical conditions. However, these rates may not accurately reflect the 'true need' for hospitalization. Such evidence is often skewed by factors like acceptability, accessibility, availability, and affordability of healthcare services. To ascertain the 'true need' for hospitalization, unhindered by external factors and barriers, we were engaged in consultative discussions with relevant commissioners and advisors as experts. The experts examined the incidence of acute medical conditions alongside corresponding mortality figures, to ascertain the severity of the acute medical conditions. Also, a review of global literature was conducted to compile additional information regarding severity classifications for the selected acute medical conditions. We assigned hospitalization needs to each condition based on this approach (Table 7).

Table 7 Assumptions related to hospitalization needs for acute medical conditions

Disease/ Condition	Hospitalization Need (%) 0-28 days (neonates)	<5 years (young child)	5-14 years (older child)	15-55 years (young adult)	>55 years (older adult)	Justification of the assumption
Asthma	Not applicable	5%	1%	1%	5%	GBD 2021 reports ~0.5 million Asthma deaths. We assumed that the hospitalization need is 20 times the number of deaths. Hence the hospitalization need is 3% of the overall burden. Asthma among younger children and older adults is expected to be more severe. Hence, we assumed the hospitalization need is 5% among younger children, 1% among older children, 1% among younger adults and 5% among older adults.
CVD	Not applicable	Not applicable	Not applicable	25%	25%	GBD 2021 reports ~20 million CVD deaths. Almost half of these deaths would be sudden. We assumed that the hospitalization need is 20 times the number of remaining deaths. Hence the hospitalization need rate is 25% of the overall burden.
COVID-19	Not applicable	1%	0.5%	4%	10%	GBD 2021 reports ~8 million COVID deaths. We assumed that the hospitalization need is 20 times the number of deaths. Hence the hospitalization need rate is 7% of the overall burden. Most of the COVID deaths are among older adults, followed by younger adults, children and younger children. Hence, we assumed the hospitalization need is 1% among children, 0.5% among older children, 4% among younger adults and 10% among older adults.
Dengue	Not applicable	25%	10%	10%	25%	Narvaez et al [19] reports 20% of dengue progress to Dengue Hemorrhagic Fever (DHF) and 10% to Dengue Shock Syndrome (DSS) as 10%. We assumed one-fourth of all DHF and all DSS need hospitalization. Hence the hospitalization need rate is 15% of the overall burden. Dengue is expected to be more severe among younger children and older adults, based on the case fatality rates. Hence, we assumed the hospitalization need is 1% among children, 0.5% among older children, 4% among younger adults and 10% among older adults.
Diarrhoea	Not applicable	5%	2%	2%	5%	Lamberti et al[20] reports (systematic review), 35% of diarrhoea among younger children is moderate and 0.5% is severe. From here we assumed all severe cases and 10-15% of moderate cases require hospitalisation resulting in an average hospitalisation 5% rate. For age groups aged 5 to 55 years we assumed the disease severity to be around half of the other two age groups and assumed the hospitalisation rate to be 2%.

	Hospitalization Need (%)					
**Disease/ Condition**	**0-28 days (neonates)**	**<5 years (young child)**	**5-14 years (older child)**	**15-55 years (young adult)**	**>55 years (older adult)**	**Justification of the assumption**
Encephalitis	Not applicable	100%	100%	100%	100%	GBD 2021 reports ~100k encephalitis deaths. We assumed that the hospitalization need is 20 times the number of deaths, which is more than 100% of the overall burden. Hence, we assumed the hospitalization need is 100% among children, older children, younger adults and older adults.
HIV/AIDS	Not applicable	25%	10%	10%	25%	GBD 2021 reports ~0.75 million HIV deaths. We assumed that the hospitalization need is 20 times the number of deaths. Hence the hospitalization need rate is 15% of the overall burden. HIV/AIDS is expected to be more severe among younger children and older adults, based on the case fatality rates. Hence, we assumed the hospitalization need is 25% among children, 10% among older children, 10% among younger adults and 25% among older adults.
Lower respiratory infection	Not applicable	30%	15%	15%	30%	GBD 2021 reports ~0.5 million LRI deaths among younger children. We assumed that the hospitalization need is 20 times the number of deaths. Hence the hospitalization need is 27% of the overall burden among younger children. McAllister et al. reports that ~16+million younger children were hospitalized in 2015 and it is increasing over time [21]. LRI is expected to be more severe among younger children and older adults, based on the case fatality rates. Hence, we assumed that the hospitalization need is 30% among children, 15% among older children, 15% among younger adults and 30% among older adults.
Malaria	Not applicable	25%	10%	10%	25%	Zeidan et al. report that 12% of malaria among children <15 years are severe [22]. Malaria is expected to be more severe among younger children and older adults, based on the case fatality rates reported by GBD. Hence, we assumed that the hospitalization need is 25% among children, 10% among older children, 10% among younger adults and 25% among older adults.
Measles and pertussis	Not applicable	30%	15%	Not applicable	Not applicable	We used the same assumptions as LRI. Hence we assumed that the hospitalization need is 30% among younger children and 15% among older children.
Meningitis	Not applicable	100%	100%	100%	100%	GBD 2021 reports ~100k encephalitis deaths. We assumed that the hospitalization need is 20 times the number of deaths, which is more than 100% of the overall burden. Hence, we assumed that the hospitalization need is 100% among children, older children, younger adults and older adults.

	Hospitalization Need (%)					
Disease/ Condition	0-28 days (neonates)	<5 years (young child)	5-14 years (older child)	15-55 years (young adult)	>55 years (older adult)	Justification of the assumption
Neonatal Encephalopathy/ Birth asphyxia	100%	Not applicable	Not applicable	Not applicable	Not applicable	GBD 2021 reports ~0.5 million neonatal encephalopathy/birth asphyxia deaths. We assumed that the hospitalization need is 20 times the number of deaths, which is more than the overall disease burden. Hence, we assumed that the hospitalization need is 100% among neonates.
Neonate Lower respiratory infection	100%	Not applicable	Not applicable	Not applicable	Not applicable	GBD 2021 reports ~150 k neonatal LRI deaths. We assumed that the hospitalization need is 20 times the number of deaths, which is more than the overall disease burden. Hence, we assumed that the hospitalization need is 100% among neonates.
Neonatal preterm birth	33%	Not applicable	Not applicable	Not applicable	Not applicable	Ohuma et al. reports that 5% of preterm are <28 week of gestational age (GA), 10% are 28-<32 weeks of gestation. We assume that all of them need hospitalization. Of the remining 85% of preterm (32-<37 weeks of GA), we assume 20% will need hospitalization. Hence, we assume that one-third (33%) of all preterm need hospitalization.
Neonatal sepsis	Not applicable	100%	Not applicable	Not applicable	Not applicable	GBD 2021 reports ~250 k neonatal sepsis deaths. We assumed that the hospitalization need is 20 times the number of deaths, which is more than the overall disease burden. Hence, we assumed that the hospitalization need is 100% among neonates.
Nutritional deficiencies	Not applicable	5%	1%	Not applicable	Not applicable	GBD 2021 reports ~100 k nutritional deficiency deaths. We assumed that the hospitalization need is 20 times the number of deaths. Hence the hospitalization need is 1% of the overall burden. Nutritional deficiency is expected to be more severe among younger children. Hence, we assumed that the hospitalization need is 5% among younger children and 1% among older children.
Other diseases	Not applicable	0.5%	0.5%	0.5%	0.5%	We assumed that the least hospitalization rate for other disease (0.5%) by taking the assumption of hospitalisation rate of children aged 5-14 years with COVID-19
Sepsis (not otherwise classified)*	90%	90%	90%	90%	90%	Any sepsis is severe and need specialised management. Hence, we assumed that the hospitalization need is 90% (almost universal).
Trauma/Injury	Not applicable	30%	15%	15%	30%	GBD 2021 reports ~4.3 million trauma/injury deaths. We assumed that the hospitalization need is 20 times the number of deaths. Hence the hospitalization need rate is 15% of the overall burden. Trauma/injury is expected to be more severe among younger children and older adults, based on the case fatality rates. Hence, we assumed the hospitalization need is 30% among children, 15%

	Hospitalization Need (%)					
Disease/ Condition	0-28 days (neonates)	<5 years (young child)	5-14 years (older child)	15-55 years (young adult)	>55 years (older adult)	Justification of the assumption
						among older children, 15% among younger adults and 30% among older adults.
Tuberculosis	Not applicable	30%	15%	15%	30%	GBD 2021 reports ~1.2 million tuberculosis deaths. We assumed that the hospitalization need is 20 times the number of deaths. Hence the hospitalization need rate is 20% of the overall burden. Tuberculosis is expected to be more severe among younger children and older adults, based on the case fatality rates. Hence, we assumed the hospitalization need is 30% among children, 15% among older children, 15% among younger adults and 30% among older adults.
Typhoid	Not applicable	25%	10%	10%	25%	GBD 2021 reports ~100 k typhoid deaths. We assumed that the hospitalization need is 20 times the number of deaths. Hence the hospitalization need rate is 23% of the overall burden. Typhoid is expected to be more severe among younger children and older adults, based on GBD reported case fatality rates. Hence, we assumed the hospitalization need is 25% among children, 10% among older children, 10% among younger adults and 25% among older adults.

Step 4: Hypoxaemia proportions among patients admitted with acute medical conditions/diseases

We mapped the conditions/diseases reported in the systematic review with the list of acute medical conditions with oxygen needs. For conditions/diseases that were reported in the systematic review (i.e., meta-estimate available), we directly took the hypoxaemia proportion estimate for admitted patients. For conditions that were not reported in the systematic review (i.e., no meta-estimate available), we used a proxy based on expert consultation with commissioners and advisors. Table 8 summarizes the assumptions related to hypoxaemia proportions for each condition/disease and their sources.

We also generated region-specific estimates (meta-analysis) of hypoxaemia proportions for each condition/disease based on the availability of data. For conditions where a region-specific meta-estimate could not be generated, we used the global estimate as proxy. Table 9 summarizes the region-specific assumptions related to hypoxaemia proportions for each condition/disease and their sources.

We mapped the conditions/diseases reported in the acute hypoxaemia systematic review with the list of acute medical conditions with oxygen needs identified in Step 1. For those conditions/diseases that were reported in the systematic review (i.e., meta-estimate available) we directly applied the hypoxemia proportion estimates for hospitalized patients to our calculations. Conversely, for conditions not reported in the systematic review (i.e., meta-estimate not available), we adopted proxy estimates based on expert consultations with commissioners and advisors. These assumptions regarding hypoxemia proportions for each condition/disease, along with their respective sources, are summarized in Table 8.

Further, we conducted a region-specific meta-analysis to ascertain hypoxemia proportions for each condition/disease, contingent upon data availability. In cases where a region-specific meta-estimate were unattainable, we defaulted to utilizing the global estimates. Table 9 provides a summary of the region-specific assumptions pertaining to hypoxemia proportions for each condition/disease, including their sources.

To report the uncertainty intervals for the number of individuals requiring oxygen therapy, we employed the 95% confidence intervals of hypoxemia proportions (systematic review) alongside the upper and lower bounds of disease burden for each condition/ disease (GBD).

Table 8 Assumptions related to hypoxaemia proportion estimates for different acute medical conditions

	Hypoxaemia proportion (%)					Source
Disease/Condition	0-28 days	<5 years	5-14 years	15-55 years	>55 years	
Asthma	Not applicable	49.8%	49.8%	20.4%	20.4%	Graham (2024)
CVD	Not applicable	Not applicable	Not applicable	13.7%	13.7%	Estimate of Trauma/injuries
COVID 19	Not applicable	27.5%	27.5%	44.2%	44.2%	Graham (2024)
Dengue	Not applicable	6.4%	6.4%	6.4%	6.4%	Estimate of Malaria
Diarrhoea	Not applicable	4.1%	4.1%	4.1%	4.1%	Graham (2024)
Encephalitis	Not applicable	13.7%	13.7%	25.3%	25.3%	Graham (2024)
HIV/AIDS	Not applicable	29.5%	29.5%	16.6%	16.6%	Graham (2024)
Lower respiratory infection	Not applicable	34.6%	34.6%	20.4%	20.4%	Graham (2024)
Malaria	Not applicable	6.4%	6.4%	6.4%	6.4%	Graham (2024)
Measles and pertussis	Not applicable	34.6%	34.6 %	Not applicable	Not applicable	Estimate of LRI
Meningitis	Not applicable	13.7%	13.7%	25.3%	25.3%	Graham (2024)
Neonatal Encephalopathy	32.8%	Not applicable	Not applicable	Not applicable	Not applicable	Graham (2024)
Neonate LRI	37.3%	Not applicable	Not applicable	Not applicable	Not applicable	Graham (2024)
Neonatal preterm birth	34.3%	Not applicable	Not applicable	Not applicable	Not applicable	Graham (2024)
Neonatal sepsis	Not applicable	20.6%	20.6%	Not applicable	Not applicable	Graham (2024)
Nutritional deficiencies	Not applicable	14.1%	14.1%	Not applicable	Not applicable	Graham (2024)
Other diseases	Not applicable	4.1%	4.1%	4.1%	4.1%	Estimate of Diarrhoea (lowest hypoxaemia proportion)
Sepsis (not otherwise classified)	Not applicable	25.3%	25.3%	25.3%	25.3%	Graham (2024)
Trauma/injury	Not applicable	4.6%	4.6%	13.7%	13.7%	Graham (2024)
Tuberculosis	Not applicable	34.6%	34.6 %	20.4%	20.4%	Estimate of LRI
Typhoid	Not applicable	6.4%	6.4%	6.4%	6.4%	Estimate of Malaria

Table 9 Assumptions related to region-specific hypoxaemia proportion estimates for different acute medical conditions

Age Category	Disease/Condition	East Asia & Pacific (EAP)	Europe & Central Asia (ECA)	Latin America & Caribbean (LCR)	Middle East & North Africa (MNA)	South Asia (SAR)	North America	Sub Saharan Africa	Global
		Proportion%	Proportion%	Proportion%	Proportion%	Proportion%	Proportion%	Proportion%	Proportion%
		(95% CI)	(95% CI)	(95% CI)	(95% CI)	(95% CI)	(95% CI)	(95% CI)	(95% CI)
Neonate	Neonatal encephalopathy / birth asphyxia	meta-estimate of neonatal encephalopathy globally; **32.8** (16.2, 51.8)	meta-estimate of neonatal encephalopathy globally; **32.8** (16.2, 51.8)	meta-estimate of neonatal encephalopathy globally; **32.8** (16.2, 51.8)	meta-estimate of neonatal encephalopathy globally; **32.8** (16.2, 51.8)	meta-estimate of neonatal encephalopathy globally; **32.8** (16.2, 51.8)	meta-estimate of neonatal encephalopathy globally; **32.8** (16.2, 51.8)	meta-estimate of neonatal encephalopathy globally; **32.8** (16.2, 51.8)	meta-estimate of neonatal encephalopathy globally; **32.8** (16.2, 51.8)
Neonate	Lower respiratory infections	meta-estimate of LRI globally; **37.3** (7.6, 73.5)	meta-estimate of LRI globally; **37.3** (7.6, 73.5)	meta-estimate of LRI globally; **37.3** (7.6, 73.5)	meta-estimate of LRI globally; **37.3** (7.6, 73.5)	meta-estimate of LRI globally; **37.3** (7.6, 73.5)	meta-estimate of LRI globally; **37.3** (7.6, 73.5)	meta-estimate of LRI globally; **37.3** (7.6, 73.5)	meta-estimate of LRI globally; **37.3** (7.6, 73.5)
Neonate	Neonatal preterm birth	meta-estimate of neonatal preterm birth globally; **34.3** (19.5, 50.8)	meta-estimate of neonatal preterm birth globally; **34.3** (19.5, 50.8)	meta-estimate of neonatal preterm birth globally; **34.3** (19.5, 50.8)	meta-estimate of neonatal preterm birth globally; **34.3** (19.5, 50.8)	meta-estimate of neonatal preterm birth globally; **34.3** (19.5, 50.8)	meta-estimate of neonatal preterm birth globally; **34.3** (19.5, 50.8)	meta-estimate of neonatal preterm birth globally; **34.3** (19.5, 50.8)	meta-estimate of neonatal preterm birth globally; **34.3** (19.5, 50.8)
Neonate	Sepsis and other infections	meta-estimate of neonatal sepsis globally; **20.6** (14.3, 27.7)	meta-estimate of neonatal sepsis globally; **20.6** (14.3, 27.7)	meta-estimate of neonatal sepsis globally; **20.6** (14.3, 27.7)	meta-estimate of neonatal sepsis globally; **20.6** (14.3, 27.7)	meta-estimate of neonatal sepsis in South Asia; **21.8** (13.8, 31.1)	meta-estimate of neonatal sepsis globally; **20.6** (14.3, 27.7)	meta-estimate of neonatal sepsis in Sub-Saharan Africa; **15.3** (8.4, 23.9)	meta-estimate of neonatal sepsis globally; **20.6** (14.3, 27.7)

Age Category	Disease/Condition	East Asia & Pacific (EAP)	Europe & Central Asia (ECA)	Latin America & Caribbean (LCR)	Middle East & North Africa (MNA)	South Asia (SAR)	North America	Sub Saharan Africa	Global
		Proportion%	Proportion%	Proportion%	Proportion%	Proportion%	Proportion%	Proportion%	Proportion%
		(95% CI)	(95% CI)	(95% CI)	(95% CI)	(95% CI)	(95% CI)	(95% CI)	(95% CI)
Child	Asthma	meta-estimate of asthma globally; **49.8** (13.4, 86.3)	meta-estimate of asthma globally; **49.8** (13.4, 86.3)	meta-estimate of asthma globally; **49.8** (13.4, 86.3)	meta-estimate of asthma globally; **49.8** (13.4, 86.3)	meta-estimate of asthma globally; **49.8** (13.4, 86.3)	meta-estimate of asthma globally; **49.8** (13.4, 86.3)	meta-estimate of asthma globally; **49.8** (13.4, 86.3)	meta-estimate of asthma globally; **49.8** (13.4, 86.3)
Child	COVID-19	meta-estimate of COVID-19 disease globally; **27.5** (22.0, 33.3)	meta-estimate of COVID-19 disease globally; **27.5** (22.0, 33.3)	meta-estimate of COVID-19 disease globally; **27.5** (22.0, 33.3)	meta-estimate of COVID-19 disease globally; **27.5** (22.0, 33.3)	meta-estimate of COVID-19 disease globally; **27.5** (22.0, 33.3)	meta-estimate of COVID-19 disease globally; **27.5** (22.0, 33.3)	meta-estimate of COVID-19 disease globally; **27.5** (22.0, 33.3)	meta-estimate of COVID-19 disease globally; **27.5** (22.0, 33.3)
Child	Dengue	meta-estimate of malaria globally; **6.4** (4.0, 9.3)	meta-estimate of malaria globally; **6.4** (4.0, 9.3)	meta-estimate of malaria globally; **6.4** (4.0, 9.3)	meta-estimate of malaria globally; **6.4** (4.0, 9.3)	meta-estimate of malaria globally; **6.4** (4.0, 9.3)	meta-estimate of malaria globally; **6.4** (4.0, 9.3)	meta-estimate of malaria in Sub-Saharan Africa; **7.4** (4.8, 10.5)	meta-estimate of malaria globally; **6.4** (4.0, 9.3)
Child	Diarrhoea	meta-estimate of diarrheal disease globally; **4.1** (1.3, 8.0)	meta-estimate of diarrheal disease globally; **4.1** (1.3, 8.0)	meta-estimate of diarrheal disease globally; **4.1** (1.3, 8.0)	meta-estimate of diarrheal disease globally; **4.1** (1.3, 8.0)	meta-estimate of diarrheal disease globally; **4.1** (1.3, 8.0)	meta-estimate of diarrheal disease globally; **4.1** (1.3, 8.0)	meta-estimate of diarrheal disease globally; **4.1** (1.3, 8.0)	meta-estimate of diarrheal disease globally; **4.1** (1.3, 8.0)

Age Category	Disease/Condition	East Asia & Pacific (EAP)	Europe & Central Asia (ECA)	Latin America & Caribbean (LCR)	Middle East & North Africa (MNA)	South Asia (SAR)	North America	Sub Saharan Africa	Global
		Proportion%	Proportion%	Proportion%	Proportion%	Proportion%	Proportion%	Proportion%	Proportion%
		(95% CI)	(95% CI)	(95% CI)	(95% CI)	(95% CI)	(95% CI)	(95% CI)	(95% CI)
Child	Encephalitis	meta-estimate of encephalitis globally; **13.7** (5.1, 25.3)	meta-estimate of encephalitis globally; **13.7** (5.1, 25.3)	meta-estimate of encephalitis globally; **13.7** (5.1, 25.3)	meta-estimate of encephalitis globally; **13.7** (5.1, 25.3)	meta-estimate of encephalitis globally; **13.7** (5.1, 25.3)	meta-estimate of encephalitis globally; **13.7** (5.1, 25.3)	meta-estimate of encephalitis in Sub-Saharan Africa; **7.7** (1.8, 16.8)	meta-estimate of encephalitis globally; **13.7** (5.1, 25.3)
Child	HIV/AIDS	meta-estimate of HIV globally; **29.5** (11.9, 50.7)	meta-estimate of HIV globally; **29.5** (11.9, 50.7)	meta-estimate of HIV globally; **29.5** (11.9, 50.7)	meta-estimate of HIV globally; **29.5** (11.9, 50.7)	meta-estimate of HIV globally; **29.5** (11.9, 50.7)	meta-estimate of HIV globally; **29.5** (11.9, 50.7)	meta-estimate of HIV in Sub-Saharan Africa; **29.5** (11.9, 50.7)	meta-estimate of HIV globally; **29.5** (11.9, 50.7)
Child	Lower respiratory infections	meta-estimate of LRI in East Asia & Pacific; **26.3** (16.6, 37.4)	meta-estimate of LRI globally; **34.6** (30.2, 39.1)	meta-estimate of LRI in Latin America & Caribbean; **45.1** (19.3, 72.4)	meta-estimate of LRI globally; **34.6** (30.2, 39.1)	meta-estimate of LRI in South Asia; **39.1** (33.7, 44.7)	meta-estimate of LRI globally; **34.6** (30.2, 39.1)	meta-estimate of LRI in Sub-Saharan Africa; **34.5** (26.9, 42.5)	meta-estimate of LRI globally; **34.6** (30.2, 39.1)
Child	Malaria	meta-estimate of malaria globally; **6.4** (4.0, 9.3)	meta-estimate of malaria globally; **6.4** (4.0, 9.3)	meta-estimate of malaria globally; **6.4** (4.0, 9.3)	meta-estimate of malaria globally; **6.4** (4.0, 9.3)	meta-estimate of malaria globally; **6.4** (4.0, 9.3)	meta-estimate of malaria globally; **6.4** (4.0, 9.3)	meta-estimate of malaria in Sub-Saharan Africa; **7.4** (4.8, 10.5)	meta-estimate of malaria globally; **6.4** (4.0, 9.3)

Age Category	Disease/Condition	East Asia & Pacific (EAP)	Europe & Central Asia (ECA)	Latin America & Caribbean (LCR)	Middle East & North Africa (MNA)	South Asia (SAR)	North America	Sub Saharan Africa	Global
		Proportion%	Proportion%	Proportion%	Proportion%	Proportion%	Proportion%	Proportion%	Proportion%
		(95% CI)	(95% CI)	(95% CI)	(95% CI)	(95% CI)	(95% CI)	(95% CI)	(95% CI)
Child	Measles and pertussis	meta-estimate of LRI in East Asia & Pacific; **26.3** (16.6, 37.4)	meta-estimate of LRI globally; **34.6** (30.2, 39.1)	meta-estimate of LRI in Latin America & Caribbean; **45.1** (19.3, 72.4)	meta-estimate of LRI globally; **34.6** (30.2, 39.1)	meta-estimate of LRI in South Asia; **39.1** (33.7, 44.7)	meta-estimate of LRI globally; **34.6** (30.2, 39.1)	meta-estimate of LRI in Sub-Saharan Africa; **34.5** (26.9, 42.5)	meta-estimate of LRI globally; **34.6** (30.2, 39.1)
Child	Meningitis	meta-estimate of meningitis globally; **13.7** (5.1, 25.3)	meta-estimate of meningitis globally; **13.7** (5.1, 25.3)	meta-estimate of meningitis globally; **13.7** (5.1, 25.3)	meta-estimate of meningitis globally; **13.7** (5.1, 25.3)	meta-estimate of meningitis globally; **13.7** (5.1, 25.3)	meta-estimate of meningitis globally; **13.7** (5.1, 25.3)	meta-estimate of meningitis in Sub-Saharan Africa; **7.7** (1.8, 16.8)	meta-estimate of meningitis globally; **13.7** (5.1, 25.3)
Child	Nutritional deficiencies	meta-estimate of malnutrition in East Asia and Pacific; **19.3** (13.4, 25.9)	meta-estimate of malnutrition globally; **14.1** (7.7, 21.9)	meta-estimate of malnutrition globally; **14.1** (7.7, 21.9)	meta-estimate of malnutrition globally; **14.1** (7.7, 21.9)	meta-estimate of malnutrition in South Asia; **22.3** (9.4, 38.5)	meta-estimate of malnutrition globally; **14.1** (7.7, 21.9)	meta-estimate of malnutrition in Sub-Saharan Africa; **8.1** (1.2, 19.3)	meta-estimate of malnutrition globally; **14.1** (7.7, 21.9)
Child	Trauma/Injury	meta-estimate of trauma globally; **4.6** (2.9, 6.7)	meta-estimate of trauma globally; **4.6** (2.9, 6.7)	meta-estimate of trauma globally; **4.6** (2.9, 6.7)	meta-estimate of trauma globally; **4.6** (2.9, 6.7)	meta-estimate of trauma globally; **4.6** (2.9, 6.7)	meta-estimate of trauma globally; **4.6** (2.9, 6.7)	meta-estimate of trauma globally; **4.6** (2.9, 6.7)	meta-estimate of trauma globally; **4.6** (2.9, 6.7)

Age Category	Disease/Condition	East Asia & Pacific (EAP)	Europe & Central Asia (ECA)	Latin America & Caribbean (LCR)	Middle East & North Africa (MNA)	South Asia (SAR)	North America	Sub Saharan Africa	Global
		Proportion%	Proportion%	Proportion%	Proportion%	Proportion%	Proportion%	Proportion%	Proportion%
		(95% CI)	(95% CI)	(95% CI)	(95% CI)	(95% CI)	(95% CI)	(95% CI)	(95% CI)
Child	Tuberculosis	meta-estimate of LRI in East Asia & Pacific; **26.3** (16.6, 37.4)	meta-estimate of LRI globally; **34.6** (30.2, 39.1)	meta-estimate of LRI in Latin America & Caribbean; **45.1** (19.3, 72.4)	meta-estimate of LRI globally; **34.6** (30.2, 39.1)	meta-estimate of LRI in South Asia; **39.1** (33.7, 44.7)	meta-estimate of LRI globally; **34.6** (30.2, 39.1)	meta-estimate of LRI in Sub-Saharan Africa; **34.5** (26.9, 42.5)	meta-estimate of LRI globally; **34.6** (30.2, 39.1)
Child	Typhoid	meta-estimate of malaria globally; **6.4** (4.0, 9.3)	meta-estimate of malaria globally; **6.4** (4.0, 9.3)	meta-estimate of malaria globally; **6.4** (4.0, 9.3)	meta-estimate of malaria globally; **6.4** (4.0, 9.3)	meta-estimate of malaria globally; **6.4** (4.0, 9.3)	meta-estimate of malaria globally; **6.4** (4.0, 9.3)	meta-estimate of malaria in Sub-Saharan Africa; **7.4** (4.8, 10.5)	meta-estimate of malaria globally; **6.4** (4.0, 9.3)
Adult	Asthma	meta-estimate of LRI globally; **20.4** (7.7, 37.0)	meta-estimate of LRI globally; **20.4** (7.7, 37.0)	meta-estimate of LRI globally; **20.4** (7.7, 37.0)	meta-estimate of LRI globally; **20.4** (7.7, 37.0)	meta-estimate of LRI globally; **20.4** (7.7, 37.0)	meta-estimate of LRI globally; **20.4** (7.7, 37.0)	meta-estimate of LRI in Sub-Saharan Africa; **18.0** (12.2, 24.7)	meta-estimate of LRI globally; **20.4** (7.7, 37.0)
Adult	COVID-19	meta-estimate of COVID-19 globally: **44.2** (36.6, 51.9)	meta-estimate of COVID-19 globally: **44.2** (36.6, 51.9)	meta-estimate of COVID-19 in Latin America & Caribbean: **52.2** (38.2, 66.0)	meta-estimate of COVID-19 in Middle East & North Africa: **46.4** (35.5, 57.5)	meta-estimate of COVID-19 in South Asia: **39.1** (18.3, 62.1)	meta-estimate of COVID-19 globally: **44.2** (36.6, 51.9)	meta-estimate of COVID-19 in Sub-Saharan Africa: **53.1** (19.3, 85.3)	meta-estimate of COVID-19 globally: **44.2** (36.6, 51.9)

Age Category	Disease/Condition	East Asia & Pacific (EAP)	Europe & Central Asia (ECA)	Latin America & Caribbean (LCR)	Middle East & North Africa (MNA)	South Asia (SAR)	North America	Sub Saharan Africa	Global
		Proportion%	Proportion%	Proportion%	Proportion%	Proportion%	Proportion%	Proportion%	Proportion%
		(95% CI)	(95% CI)	(95% CI)	(95% CI)	(95% CI)	(95% CI)	(95% CI)	(95% CI)
Adult	CVD	meta-estimate of trauma globally; **13.7** (4.8, 26.3)	meta-estimate of trauma globally; **13.7** (4.8, 26.3)	meta-estimate of trauma globally; **13.7** (4.8, 26.3)	meta-estimate of trauma globally; **13.7** (4.8, 26.3)	meta-estimate of trauma globally; **13.7** (4.8, 26.3)	meta-estimate of trauma globally; **13.7** (4.8, 26.3)	meta-estimate of trauma in Sub-Saharan Africa; **13.7** (4.8, 26.3)	meta-estimate of trauma globally; **13.7** (4.8, 26.3)
Adult	Dengue	meta-estimate of child malaria globally; **6.4** (4.0, 9.3)	meta-estimate of child malaria globally; **6.4** (4.0, 9.3)	meta-estimate of child malaria globally; **6.4** (4.0, 9.3)	meta-estimate of child malaria globally; **6.4** (4.0, 9.3)	meta-estimate of child malaria globally; **6.4** (4.0, 9.3)	meta-estimate of child malaria globally; **6.4** (4.0, 9.3)	meta-estimate of child malaria in Sub-Saharan Africa; **7.4** (4.8, 10.5)	meta-estimate of child malaria globally; **6.4** (4.0, 9.3)
Adult	Diarrhoea	meta-estimate of child diarrhoea disease globally; **4.1** (1.3, 8.0)	meta-estimate of child diarrhoea disease globally; **4.1** (1.3, 8.0)	meta-estimate of child diarrhoea disease globally; **4.1** (1.3, 8.0)	meta-estimate of child diarrhoea disease globally; **4.1** (1.3, 8.0)	meta-estimate of child diarrhoea disease globally; **4.1** (1.3, 8.0)	meta-estimate of child diarrhoea disease globally; **4.1** (1.3, 8.0)	meta-estimate of child diarrhoea disease globally; **4.1** (1.3, 8.0)	meta-estimate of child diarrhoea disease globally; **4.1** (1.3, 8.0)
Adult	Encephalitis	Meta-estimate of sepsis globally; **25.3** (14.8, 37.4)	Meta-estimate of sepsis globally; **25.3** (14.8, 37.4)	Meta-estimate of sepsis globally; **25.3** (14.8, 37.4)	Meta-estimate of sepsis globally; **25.3** (14.8, 37.4)	Meta-estimate of sepsis globally; **25.3** (14.8, 37.4)	Meta-estimate of sepsis globally; **25.3** (14.8, 37.4)	Meta-estimate of sepsis globally; **25.3** (14.8, 37.4)	Meta-estimate of sepsis globally; **25.3** (14.8, 37.4)
Adult	HIV/AIDS resulting in other diseases	meta-estimate of HIV globally;	meta-estimate of HIV globally;	meta-estimate of HIV globally;	meta-estimate of HIV globally;	meta-estimate of HIV globally;	meta-estimate of HIV globally;	meta-estimate of HIV in Sub-Saharan Africa;	Meta-estimate of HIV globally;

Age Category	Disease/Condition	East Asia & Pacific (EAP)	Europe & Central Asia (ECA)	Latin America & Caribbean (LCR)	Middle East & North Africa (MNA)	South Asia (SAR)	North America	Sub Saharan Africa	Global
		Proportion%	Proportion%	Proportion%	Proportion%	Proportion%	Proportion%	Proportion%	Proportion%
		(95% CI)	(95% CI)	(95% CI)	(95% CI)	(95% CI)	(95% CI)	(95% CI)	(95% CI)
		**16.6** (11.6, 22.4)	**16.6** (11.6, 22.4)	**16.6** (11.6, 22.4)	**16.6** (11.6, 22.4)	**16.6** (11.6, 22.4)	**16.6** (11.6, 22.4)	**16.6** (11.6, 22.4)	**16.6** (11.6, 22.4)
Adult	Lower respiratory infections	meta-estimate of LRI globally; **20.4** (7.7, 37.0)	meta-estimate of LRI globally; **20.4** (7.7, 37.0)	meta-estimate of LRI globally; **20.4** (7.7, 37.0)	meta-estimate of LRI globally; **20.4** (7.7, 37.0)	meta-estimate of LRI globally; **20.4** (7.7, 37.0)	meta-estimate of LRI globally; **20.4** (7.7, 37.0)	meta-estimate of LRI in Sub-Saharan Africa; **18.0** (12.2, 24.7)	meta-estimate of LRI globally; **20.4** (7.7, 37.0)
Adult	Malaria	meta-estimate of child malaria globally; **6.4** (4.0, 9.3)	meta-estimate of child malaria globally; **6.4** (4.0, 9.3)	meta-estimate of child malaria globally; **6.4** (4.0, 9.3)	meta-estimate of child malaria globally; **6.4** (4.0, 9.3)	meta-estimate of child malaria globally; **6.4** (4.0, 9.3)	meta-estimate of child malaria globally; **6.4** (4.0, 9.3)	meta-estimate of child malaria in Sub-Saharan Africa; **7.4** (4.8, 10.5)	meta-estimate of child malaria globally; **6.4** (4.0, 9.3)
Adult	Meningitis	Meta-estimate of sepsis globally; **25.3** (14.8, 37.4)	Meta-estimate of sepsis globally; **25.3** (14.8, 37.4)	Meta-estimate of sepsis globally; **25.3** (14.8, 37.4)	Meta-estimate of sepsis globally; **25.3** (14.8, 37.4)	Meta-estimate of sepsis globally; **25.3** (14.8, 37.4)	Meta-estimate of sepsis globally; **25.3** (14.8, 37.4)	Meta-estimate of sepsis globally; **25.3** (14.8, 37.4)	Meta-estimate of sepsis globally; **25.3** (14.8, 37.4)
Adult	Trauma/Injury	meta-estimate of trauma globally; **13.7** (4.8, 26.3)	meta-estimate of trauma globally; **13.7** (4.8, 26.3)	meta-estimate of trauma globally; **13.7** (4.8, 26.3)	meta-estimate of trauma globally; **13.7** (4.8, 26.3)	meta-estimate of trauma globally; **13.7** (4.8, 26.3)	meta-estimate of trauma globally; **13.7** (4.8, 26.3)	meta-estimate of trauma in Sub-Saharan Africa; **13.7** (4.8, 26.3)	meta-estimate of trauma globally; **13.7** (4.8, 26.3)

Age Category	Disease/Condition	East Asia & Pacific (EAP)	Europe & Central Asia (ECA)	Latin America & Caribbean (LCR)	Middle East & North Africa (MNA)	South Asia (SAR)	North America	Sub Saharan Africa	Global
		Proportion%	Proportion%	Proportion%	Proportion%	Proportion%	Proportion%	Proportion%	Proportion%
		(95% CI)	(95% CI)	(95% CI)	(95% CI)	(95% CI)	(95% CI)	(95% CI)	(95% CI)
Adult	Tuberculosis	meta-estimate of LRI globally; **20.4** (7.7, 37.0)	meta-estimate of LRI globally; **20.4** (7.7, 37.0)	meta-estimate of LRI globally; **20.4** (7.7, 37.0)	meta-estimate of LRI globally; **20.4** (7.7, 37.0)	meta-estimate of LRI globally; **20.4** (7.7, 37.0)	meta-estimate of LRI globally; **20.4** (7.7, 37.0)	meta-estimate of LRI in Sub-Saharan Africa; **18.0** (12.2, 24.7)	meta-estimate of LRI globally; **20.4** (7.7, 37.0)
Adult	Typhoid	meta-estimate of child malaria globally; **6.4** (4.0, 9.3)	meta-estimate of child malaria globally; **6.4** (4.0, 9.3)	meta-estimate of child malaria globally; **6.4** (4.0, 9.3)	meta-estimate of child malaria globally; **6.4** (4.0, 9.3)	meta-estimate of child malaria globally; **6.4** (4.0, 9.3)	meta-estimate of child malaria globally; **6.4** (4.0, 9.3)	meta-estimate of child malaria in Sub-Saharan Africa; **7.4** (4.8, 10.5)	meta-estimate of child malaria globally; **6.4** (4.0, 9.3)
All ages	Sepsis (non-infections) *	Meta-estimate of sepsis globally; **25.3** (14.8, 37.4)	Meta-estimate of sepsis globally; **25.3** (14.8, 37.4)	Meta-estimate of sepsis globally; **25.3** (14.8, 37.4)	Meta-estimate of sepsis globally; **25.3** (14.8, 37.4)	Meta-estimate of sepsis globally; **25.3** (14.8, 37.4)	Meta-estimate of sepsis globally; **25.3** (14.8, 37.4)	Meta-estimate of sepsis globally; **25.3** (14.8, 37.4)	Meta-estimate of sepsis globally; **25.3** (14.8, 37.4)
Other Diseases		meta-estimate of child diarrhoea disease globally; **4.1** (1.3, 8.0)	meta-estimate of child diarrhoea disease globally; **4.1** (1.3, 8.0)	meta-estimate of child diarrhoea disease globally; **4.1** (1.3, 8.0)	meta-estimate of child diarrhoea disease globally; **4.1** (1.3, 8.0)	meta-estimate of child diarrhoea disease globally; **4.1** (1.3, 8.0)	meta-estimate of child diarrhoea disease globally; **4.1** (1.3, 8.0)	meta-estimate of child diarrhoea disease globally **4.1** (1.3, 8.0)	meta-estimate of child diarrhoea disease globally; **4.1** (1.3, 8.0)

**Green denotes meta-estimate available, yellow denotes not enough data available to conduct a meta-analysis, grey denotes no datapoints.*

Step 5: Inflation factors

5a Moderate hypoxaemia:

In our estimation of oxygen needs, we used the hypoxaemia proportions of patients admitted with acute medical conditions from the systematic review by Graham et al. (2024). The review used a cut-off of SpO_2< 90% for defining hypoxaemia. While WHO also recommends this threshold, it is important to note that individuals with moderate hypoxaemia (SpO_2 between 90%-93%/94%) face a significantly higher mortality risk compared to those with normoxaemia (SpO_2>98%). Given that a considerable number of patients presenting with moderate hypoxaemia may require medical oxygen therapy, their inclusion in our estimation of oxygen needs was considered essential.

To understand the burden of moderate hypoxaemia across various conditions/diseases and age groups, we examined several studies. McCollum et al. reported that out of 3,848 outpatient children aged 3 to 11 months with suspected pneumonia, 2.7% had hypoxaemia (SpO_2< 90%), while 8% had moderate hypoxaemia (SpO_2 90-93%) [23]. Hooli et al. found that among 1,853 children younger than 2 months presenting with cough or shortness of breath, 14.7% had hypoxaemia (SpO_2< 90%), 11.5% had moderate hypoxaemia (SpO_2 90-92%) [24]. In adults with COVID-19, Acar et al. observed that out of 709 patients, 9.3% suffered from hypoxaemia (SpO_2< 89%), and 30.2% had moderate hypoxaemia (SpO_2 89-94%)[25]. Additional evidence from Padmaprakash et al. indicated that among 1,536 hospitalized COVID-19 patients aged 15 years or older, 0.8%, had SpO_2<75%, 4.3% had SpO_2 75-90% and 3.4% had SpO_2 90-94% [26]. Krebs et al.'s study on traumatic brain injury patients aged ten years or older revealed that 4% had hypoxaemia (SpO_2< 90%), and 13.3% had moderate hypoxaemia (SpO_2 90-94%) [27]. King et al.'s research on children aged twelve years or younger referred for any cause showed that 8.6% had hypoxaemia (SpO_2< 90%), and an additional 13.1% had moderate hypoxaemia (SpO_2< 90-93%) [28]. Wasingya-Kasereka et al.'s study on adult hospitalized patients due to various causes found that 6.3% had hypoxaemia (SpO_2< 90%), and 21.8%, had moderate hypoxaemia (SpO_2< 90-94%) [29]. Lastly, Junge et al.'s examination of hospitalized children for any reason reported that 6% had hypoxaemia (SpO_2< 90%), while 13% had moderate hypoxaemia (SpO_2 90-94%) [30].

Given the substantial evidence demonstrating the high burden of moderate hypoxaemia, we adjusted our estimates conservatively by an additional 20% to account for the oxygen needs associated with this condition.

5b serial assessments:

Most of the papers that report hypoxaemia proportion typically focus on hypoxaemia at admission and often overlook the dynamic nature of oxygen requirements, which can escalate post-admission. Cunningham et al.'s study indicates that while approximately 40% of patients exhibit hypoxaemia upon admission, the demand for oxygen therapy increases to about 65% during their hospital stay [31]. To address the risk of underestimating oxygen needs due to the lack of subsequent serial assessments, we introduced an inflation factor of 10%; this adjustment accounts for normoxaemic patients who may develop or experience hypoxaemia after admission.

2.2.1b Oxygen needs in peri-operative care

To estimate the oxygen needs during the perioperative period for major surgeries, we employed the following formula:

Oxygen needs during peri-operative period = Population (number) * major surgery rate * proportion of major surgeries requiring oxygen during the perioperative period

Population: We used GBD 2021 population estimate (without any age filter). We used global population for generating global estimate and region (World Bank) specific population for generating region specific estimates.

Surgery rate: We used the rates reported in the Lancet Surgery Commission. We mapped the regions reported in the Lancet Surgical Commission with the relevant World Bank regions used in the oxygen commission reporting. Then we re-estimated the World Bank region specific surgery rates (Table 10).

Proportion of surgeries requiring oxygen: We assumed **70%** of **all major surgeries** will require oxygen during peri-operative period. The explanation is as follows:

- The Lancet Surgical Commission (table 2; page 576) reports the global and region-specific surgical rates. [32] They have used the original work done by Rose and colleagues for generating these estimates. [33] Rose and colleagues defined **surgery as any procedure requiring general or neuraxial anaesthesia in the operating theatre** (page s15). [33] The global surgery need is 321,505,362 per year, of which 12.04% (38,720,343) are due to maternal conditions (supplementary material, appendix 1, page 2). We assumed that almost all the surgeries due to maternal complications can be performed through neuraxial anaesthesia. Of note, this estimate does not include surgeries or anaesthetics performed outside of the operating theatre or in the outpatient settings (e.g. colonoscopies with anaesthesia care). Thus, our estimate is likely a conservative underestimation of oxygen needs for surgery and anaesthesia care worldwide.
- We conducted a desk review to locate published studies of general anaesthesia versus neuraxial rates globally and found limited data. There was wide variation of this rate between different income level countries with a trend toward higher prevalence of general anaesthesia in high-income countries compared to low-and middle-income countries. Countries in this income group such as India have reported rates at about 40% neuraxial anaesthesia, with other LMICs like Uganda reporting similar rates (50%). [34 35] On the contrary, high-income countries report a higher rate of general anaesthesia compared to neuraxial anaesthesia. Examples include China, UK and Chile where the rates of general anaesthesia are 80%, 77% and 70% respectively. [36-38] The extent to which higher general anaesthesia rates in high-income countries reflects factors like patient preference or availability of resources was uncertain and unable to be quantified.
- The commission informally engaged with numerous anaesthesia experts across multiple countries, continents and income levels to inquire about anaesthesia practice patterns
- For each anaesthetic done under neuraxial, we assumed that no oxygen is necessarily needed, though recognize oxygen must always be available and not infrequently will be utilized during this anaesthesia.

<u>Oxygen volume assumption:</u>

To estimate the yearly oxygen volume required for peri-operative care during surgeries, we employed the following equation:

Oxygen volume (in one year) in acute medical, surgical or chronic conditions = Amount of oxygen needed in one minute (litres per minute) * minutes per hour * number of hours needed per day (hours) * number of days needed (days) * number of people requiring oxygen (number)

Following consultation with global experts in anaesthesia, the following assumption was taken:

- For each surgery done under general anaesthesia, we assumed that average case duration was **2.5 hours** and would likely receive at least **2 litres per minute** of oxygen for the duration of the case. We considered there is likely to be wide variability in practice across settings due to factors including provider preference, access to compressed medical air, and variable access to types of anaesthesia machine or CO_2 absorbent materials, among other factors.
- For each surgery done under general anaesthesia, we assumed that while in post-operative recovery, each patient was likely to receive oxygen for at least **0.5 hours** and likely at a rate of approximately **4 litres per minute** of oxygen. We considered wide variability in practice patterns, resource availability, needs of different patients and no clear standard of care.
- Thus, for the purpose of estimating oxygen needs in the immediate perioperative period we assumed **(2.5h*60min/h*2LPM) +(0.5h*60min/h*4LPM) = 420 litres per case.**

Table 10 Estimation of surgery rates by World Bank regions

WB region	Population (millions)	Surgery need (millions)	Surgery rate (surgery need/ population)
East Asia & Pacific	1612	68.8	0.0427
Europe & Central Asia	822	42.7	0.0519
Latin America & Caribbean	590	22.2	0.0376
Middle East & North Africa	446	19.8	0.0444
North America	340	15.8	0.0465
South Asia	2223	97.7	0.0444
Sub-Saharan Africa	859	53.3	0.0620
Global	6893	321.3	0.0466

2.2.1c Oxygen needs in chronic conditions

Long-term oxygen therapy (LTOT) is a treatment for patients with chronic respiratory diseases who have low levels of oxygen in their blood. It involves the use of supplemental oxygen for a significant number of hours each day, typically over 15 hours, to improve survival and quality of life. Conditions such as chronic obstructive pulmonary disease (COPD), emphysema, pulmonary fibrosis, bronchiectasis, and cystic fibrosis may necessitate LTOT. In this review, we concentrate on the requirements for LTOT in patients with COPD, as current literature indicates the most substantial benefit from LTOT is observed within this demographic [39 40].

To estimate the LTOT needs in COPD, we employed the following formula:

Population (COPD) prevalence (number) * Proportion of LTOT (%/100)

We adopted the following steps execute the formula:

Step 1 Identify population prevalence

We used the global, regional (World Bank regions) and country specific prevalence of COPD of people aged ≥ 40 years reported in GBD (Table 11).

Table 11 COPD burden mapping in GBD

Disease/ Condition	GBD-ID	GBD-Level	GBD terminology (selected category in bold)
COPD	509	Level 3	Level 2: Chronic Respiratory Diseases<br>**Level 3: Chronic obstructive pulmonary disease (selected)**, pneumoconiosis, Asthma, Interstitial Lung Disease and Pulmonary Sarcoidosis, Other Chronic Respiratory Diseases<br>Level 4: No relevant disease of the selected level 3 disease

Step 2 Estimate LTOT proportions in COPD

We conducted a systematic review to estimate the proportion of LTOT needs among people with COPD. The review was registered in PROSPERO (CRD42023464405). From the review, four papers were identified, which reasonably reflected the true needs of LTOT among COPD population (Table 12). The meta-estimate was 4.67% (95% CI 3.43, 6.09).

Table 12 LTOT needs in COPD in published literature

Study Title	Country	Age	Sample Size	LTOT (%)
Home oxygen therapy reduces risk of hospitalisation in patients with chronic obstructive pulmonary disease: a population-based retrospective cohort study, 2005–2012 [11]	South Korea	>=40	36761	3.6
Frequency of comorbidities in chronic obstructive pulmonary disease, and impact on all-cause mortality: A population-based cohort study [12]	France	>=45	4237	8.1
The natural history of community-acquired pneumonia in COPD patients: A population database analysis [41]	UK	>=40	8814	4.81
Patterns and management of chronic obstructive pulmonary disease in urban and rural China: a community-based survey of 25 000 adults across 10 regions [14]	China	38-87	1586	2.77

2.2.1d Minimum oxygen volume required to meet needs estimation

To estimate the minimum annual oxygen volume required to meet needs for acute medical conditions, peri-operative care during surgeries and long term oxygen therapy in COPD, we employed the following equation:

Oxygen volume (in one year) in acute medical, surgical or chronic conditions = Amount of oxygen needed in one minute (litres per minute) * 60 (minutes) * number of hours needed per day (hours) * number of days needed (days) * number of people requiring oxygen (number)

Assumptions for the amount of oxygen and time needed for its provision were derived from clinical guidelines, limited published data, and finalised through expert consultations. The assumptions taken for each disease/condition in shown in Table 13. Importantly, these assumptions are based on efficient clinical use of oxygen. As such, the resulting estimates reflect the *minimum oxygen volume required to meet need,* assuming no wastage or inefficiencies in the clinical use of oxygen, or in upstream oxygen production, transport, distribution, or supply. Given the limited actual data for these assumptions we have not attempted to calculate confidence intervals or adjust for this uncertainty in the volume needs calculations.

Table 13 Minimum oxygen volume assumptions for different acute and chronic disease / conditions and perioperative need

Type	Disease/Condition	Assumption	Litres per episode
Acute medical oxygen need	Asthma	Child: 1.3 LMP * 3 days<br>Adult: 4 LMP * 3 days	Child: 5,616<br>Adult: 17,280
Acute medical oxygen need	CVD	Adult: 3 LMP* 2 days	Adult: 8640
Acute medical oxygen need	Dengue	Child: 1.3 LMP * 2 days<br>Adult: 4 LMP * 2 days	Child: 3,744<br>Adult: 11,520
Acute medical oxygen need	Diarrhoea	Child: 1.3 LMP * 2 days<br>Adult: 4 LMP * 2 days	Child: 3,744<br>Adult: 11,520
Acute medical oxygen need	Encephalitis	Child: 1.3 LMP * 3 days<br>Adult: 4 LMP * 3 days	Child: 5,616<br>Adult: 17,280
Acute medical oxygen need	HIV/AIDS	Child: 1.3 LMP * 3 days<br>Adult: 4 LMP * 3 days	Child: 5,616<br>Adult: 17,280
Acute medical oxygen need	Lower respiratory infection	Child: 1.3 LMP * 3 days<br>Adult: 4 LMP * 3 days	Child: 5,616<br>Adult: 17,280
Acute medical oxygen need	Malaria	Child: 1.3 LMP * 2 days<br>Adult: 4 LMP * 2 days	Child: 3,744<br>Adult: 11,520
Acute medical oxygen need	Measles and pertussis	Child: 1.3 LMP * 2 days	Child: 3,744
Acute medical oxygen need	Meningitis	Child: 1.3 LMP * 3 days<br>Adult: 4 LMP * 3 days	Child: 5,616<br>Adult: 17,280
Acute medical oxygen need	Neonatal Encephalopathy/Birth asphyxia	Neonate: 0.75 LPM * 2 days	Neonate: 2,160
Acute medical oxygen need	Neonate Lower respiratory infection	Neonate: 0.75 LPM * 2 days	Neonate: 2,160
Acute medical oxygen need	Neonatal preterm birth	Neonate: 0.75 LPM * 3.5 days	Neonate: 3,780
Acute medical oxygen need	Neonatal sepsis	Neonate: 0.75 LPM * 2 days	Neonate: 2,160

Acute medical oxygen need	Nutritional deficiencies	Child: 1.3 LMP * 2 days	Child: 3,744
Acute medical oxygen need	Other diseases	Child: 1.3 LMP * 2 days<br>Adult: 4 LMP * 2 days	Child: 3,744<br>Adult: 11,520
Acute medical oxygen need	Sepsis (not otherwise classified) *	Child: 1.3 LMP * 3 days<br>Adult: 4 LMP * 3 days	Child: 5,616<br>Adult: 17,280
Acute medical oxygen need	Trauma/Injury	Child: 1.3 LMP * 2 days<br>Adult: 4 LMP * 2 days	Child: 3,744<br>Adult: 11,520
Acute medical oxygen need	Tuberculosis	Child: 1.3 LMP * 3 days<br>Adult: 4 LMP * 3 days	Child: 5,616<br>Adult: 17,280
Acute medical oxygen need	Typhoid	Child: 1.3 LMP * 2 days<br>Adult: 4 LMP * 2 days	Child: 3,744<br>Adult: 11,520
Acute medical oxygen need	COVID 19	MV: 3.2 LMP * 12 days and afterwards 5 LMP * 4 days<br>HFNC: 40 LMP *3 days and afterwards 5 LMP * 4 days<br>Non-ICU: 5 LMP * 4 days	Child: 11,722<br>Adult: 35,165
Acute surgical (perioperative) oxygen need	Major Surgery with General Anaesthesia	All: (2.5 hours per day * 2 litre per minute) +(0.5 hours per day * 4 litre per minute) * 1 day	All: 420
Chronic oxygen need	COPD*	Adult: 1 litre per minute * 15 hours per day * 365 days	Adult: 328,500

Table 14: Justification for the minimum oxygen volume assumptions for different acute and chronic disease / conditions and perioperative need

Type	Disease/Condition	Justification of assumption
Acute medical oxygen need	Asthma	The World Health Organization (WHO) recommends an oxygen flow rate of 1-2 LPM for infants and 1-4 LPM for older children [42]. Conversely, Graham et al. (2019) observed in 12 hospitals in Nigeria that the flow rate was 1.0–1.5 LPM for younger children and 1.0–2.0 LPM for older children [43]. The duration of oxygen therapy was 2.8 days, with minimal variation across age groups. Children with acute lower respiratory infections (ALRI) required longer oxygen therapy (mean 3.2 days, median 2.5 days, IQR 1.5–3.5). Based on these observations, children received approximately 5000 litres of oxygen. However, Howie et al., in an MRC-funded hospital in Gambia, reported that each hypoxemic child received an average of 16000 litres of oxygen [44]. Additionally, Rojas Reyes et al. reported an oxygen flow rate of 1-2 LPM for children with lower respiratory infections (LRI) [45].<br><br>Based on this evidence, we adopted a conservative assumption of 1.3 LPM for 3 days for children with LRI or pneumonia. We also assumed that the required flow rate for adults with LRI or pneumonia would be three times that of children, i.e., 4 LPM for 3 days.<br><br>Since we do not have any specific evidence on asthma, we assumed the oxygen flow rate and duration would be same for LRI and asthma.
Acute medical oxygen need	CVD	The American College of Cardiology Foundation/American Heart Association guideline reported that patients with ST elevated myocardial infarction be given 2-4 litre/min oxygen [46]. From this estimate we took a conservative assumption of 3LPM for 2 days for all cardiovascular diseases.<br><br>Based on this evidence, we adopted a conservative assumption of 3 LPM for 2 days for adults with cardiovascular diseases.
Acute medical oxygen need	Dengue	The World Health Organization (WHO) recommends an oxygen flow rate of 1-2 LPM for infants and 1-4 LPM for older children [42]. Conversely, Graham et al. (2019) observed in 12 hospitals in Nigeria that the flow rate was 1.0–1.5 LPM for younger children and 1.0–2.0 LPM for older children [43]. The duration of oxygen therapy was 1.5 days (IQR 0.5–3.5), with minimal variation across age groups. Based on these observations, children received approximately 5000 litres of oxygen. However, Howie et al., in an MRC-funded hospital in Gambia, reported that each hypoxemic child received an average of 16000 litres of oxygen [44].<br><br>Cam et al. reported administering oxygen via a face mask with a reservoir bag at a flow rate of 6–8 LPM in paediatric patients with dengue shock syndrome [47]. Based on this evidence, we adopted a conservative assumption (noting that not all dengue patients advance to severe cases such as dengue shock syndrome or dengue haemorrhagic fever) of 1.3 LPM

		for 2 days for children with dengue. We also assumed that the required flow rate for adults with dengue would be three times that of children, i.e., 4 LPM for 2 days.
Acute medical oxygen need	Diarrhoea	The World Health Organization (WHO) recommends an oxygen flow rate of 1-2 LPM for infants and 1-4 LPM for older children [42]. Conversely, Graham et al. (2019) observed in 12 hospitals in Nigeria that the flow rate was 1.0–1.5 LPM for younger children and 1.0–2.0 LPM for older children [43]. The duration of oxygen therapy was 1.5 days (IQR 0.5–3.5), with minimal variation across age groups. Based on these observations, children received approximately 5000 litres of oxygen. However, Howie et al., in an MRC-funded hospital in Gambia, reported that each hypoxemic child received an average of 16000 litres of oxygen [44].<br><br>Based on this evidence, we adopted a conservative assumption of 1.3 LPM for 2 days for children with diarrhoea. We also assumed that the required flow rate for adults with diarrhoea would be three times that of children, i.e., 4 LPM for 2 days.
Acute medical oxygen need	Encephalitis	The World Health Organization (WHO) recommends an oxygen flow rate of 1-2 LPM for infants and 1-4 LPM for older children [42]. Conversely, Graham et al. (2019) observed in 12 hospitals in Nigeria that the flow rate was 1.0–1.5 LPM for younger children and 1.0–2.0 LPM for older children [43]. The duration of oxygen therapy was 2.8 days with minimal variation across age groups. Children with acute lower respiratory infections (ALRI) required longer oxygen therapy (mean 3.2 days, median 2.5 days, IQR 1.5–3.5). Based on these observations, children received approximately 5000 litres of oxygen. However, Howie et al., in an MRC-funded hospital in Gambia, reported that each hypoxemic child received an average of 16000 litres of oxygen [44]. Additionally, Rojas Reyes et al. reported an oxygen flow rate of 1-2 LPM for children with lower respiratory infections (LRI) [45].<br><br>Based on this evidence, we adopted a conservative assumption of 1.3 LPM for 3 days for children with LRI or pneumonia. We also assumed that the required flow rate for adults with LRI or pneumonia would be three times that of children, i.e., 4 LPM for 3 days.<br><br>Since we do not have any specific evidence on encephalitis, we assumed the oxygen flow rate and duration would be same for LRI and encephalitis.
Acute medical oxygen need	HIV/AIDS	The World Health Organization (WHO) recommends an oxygen flow rate of 1-2 LPM for infants and 1-4 LPM for older children [42]. Conversely, Graham et al. (2019) observed in 12 hospitals in Nigeria that the flow rate was 1.0–1.5 LPM for younger children and 1.0–2.0 LPM for older children [43]. The duration of oxygen therapy was 2.8 days, with minimal variation across age groups. Children with acute lower respiratory infections (ALRI) required longer oxygen therapy (mean 3.2 days, median 2.5 days, IQR 1.5–3.5). Based on these observations, children received approximately 5000 litres of oxygen. However, Howie et al., in an MRC-funded hospital in Gambia, reported that each hypoxemic child received an average of

		16000 litres of oxygen [44]. Additionally, Rojas Reyes et al. reported an oxygen flow rate of 1-2 LPM for children with lower respiratory infections (LRI) [45].<br><br>Based on this evidence, we adopted a conservative assumption of 1.3 LPM for 3 days for children with LRI or pneumonia. We also assumed that the required flow rate for adults with LRI or pneumonia would be three times that of children, i.e., 4 LPM for 3 days.<br><br>Since we do not have any specific evidence on HIV/AIDS, we assumed the oxygen flow rate and duration would be same for LRI and HIV/AIDS.
Acute medical oxygen need	Lower respiratory infection	The World Health Organization (WHO) recommends an oxygen flow rate of 1-2 L/min for infants and 1-4 L/min for older children [42]. Conversely, Graham et al. (2019) observed in 12 hospitals in Nigeria that the flow rate was 1.0–1.5 L/min for younger children and 1.0–2.0 L/min for older children [43]. The duration of oxygen therapy was 2.8 days with minimal variation across age groups. Children with acute lower respiratory infections (ALRI) required longer oxygen therapy (mean 3.2 days, median 2.5 days, IQR 1.5–3.5). Based on these observations, children received approximately 5000 liters of oxygen. However, Howee et al., in an MRC-funded hospital in Gambia, reported that each hypoxemic child received an average of 16000 liters of oxygen [44]. Additionally, Rojas Reyes et al. reported an oxygen flow rate of 1-2 L/min for children with lower respiratory infections (LRI) [45].<br><br>Based on this evidence, we adopted a conservative assumption of 1.3 L/min for 3 days for children with LRI or pneumonia. We also assumed that the required flow rate for adults with LRI or pneumonia would be three times that of children, i.e., 4 L/min for 3 days.
Acute medical oxygen need	Malaria	The World Health Organization (WHO) recommends an oxygen flow rate of 1-2 LPM for infants and 1-4 LPM for older children [42]. Conversely, Graham et al. (2019) observed in 12 hospitals in Nigeria that the flow rate was 1.0–1.5 LPM for younger children and 1.0–2.0 LPM for older children [43]. The duration of oxygen therapy was 1.5 days (IQR 0.5–3.5), with minimal variation across age groups. Based on these observations, children received approximately 5000 litres of oxygen. However, Howie et al., in an MRC-funded hospital in Gambia, reported that each hypoxemic child received an average of 16000 litres of oxygen [44].<br><br>Based on this evidence, we adopted a conservative assumption of 1.3 LPM for 2 days for children with malaria. We also assumed that the required flow rate for adults with malaria would be three times that of children, i.e., 4 LPM for 2 days.
Acute medical oxygen need	Measles and pertussis	The World Health Organization (WHO) recommends an oxygen flow rate of 1-2 LPM for infants and 1-4 LPM for older children [42]. Conversely, Graham et al. (2019) observed in 12 hospitals in Nigeria that the flow rate was 1.0–1.5 LPM for younger children and 1.0–2.0 LPM for older children [43]. The duration of oxygen therapy was 2.8 days, with minimal variation

		across age groups. Based on these observations, children received approximately 5000 litres of oxygen. However, Howie et al., in an MRC-funded hospital in Gambia, reported that each hypoxemic child received an average of 16000 litres of oxygen [44].<br><br>Based on this evidence, we adopted a conservative assumption of 1.3 LPM for 2 days for children with measles and pertussis.
Acute medical oxygen need	Meningitis	The World Health Organization (WHO) recommends an oxygen flow rate of 1-2 LPM for infants and 1-4 LPM for older children [42]. Conversely, Graham et al. (2019) observed in 12 hospitals in Nigeria that the flow rate was 1.0–1.5 LPM for younger children and 1.0–2.0 LPM for older children [43]. The duration of oxygen therapy was 2.8 days with minimal variation across age groups. Children with acute lower respiratory infections (ALRI) required longer oxygen therapy (mean 3.2 days, median 2.5 days, IQR 1.5–3.5). Based on these observations, children received approximately 5000 litres of oxygen. However, Howie et al., in an MRC-funded hospital in Gambia, reported that each hypoxemic child received an average of 16000 litres of oxygen [44]. Additionally, Rojas Reyes et al. reported an oxygen flow rate of 1-2 LPM for children with lower respiratory infections (LRI) [45].<br><br>Based on this evidence, we adopted a conservative assumption of 1.3 LPM for 3 days for children with LRI or pneumonia. We also assumed that the required flow rate for adults with LRI or pneumonia would be three times that of children, i.e., 4 LPM for 3 days.<br><br>Since we do not have any specific evidence on meningitis, we assumed the oxygen flow rate and duration would be same for LRI and meningitis.
Acute medical oxygen need	Neonatal Encephalopathy/Birth asphyxia	The WHO Handbook of oxygen therapy for children recommends that neonates be given oxygen via nasal prongs with a flow rate of 0.5-1 L/min [42]. Conversely, Graham et al. (2019) observed in 12 hospitals in Nigeria that the flow rate was 0.5-1.0 LPM for neonates [43]. The duration of oxygen therapy was 1.5 days (IQR 0.5–3.5), with minimal variation across age groups. Moreover, the paper reports that the median flowrate of oxygen for neonatal encephalopathy was 1.0 LPM (IQR 0.5-1.0) and the median duration was 1.5 days (IQR-1.5-3.5).<br><br>Based on this evidence, we adopted a conservative assumption of 0.75 LPM for 2 days for neonatal encephalopathy.
Acute medical oxygen need	Neonate Lower respiratory infection	The WHO Handbook of oxygen therapy for children recommends that neonates be given oxygen via nasal prongs with a flow rate of 0.5-1 L/min [42]. Conversely, Graham et al. (2019) observed in 12 hospitals in Nigeria that the flow rate was 0.5-1.0 LPM for neonates [43]. The duration of oxygen therapy was 1.5 days (IQR 0.5–3.5), with minimal variation across age groups.<br><br>Based on this evidence, we adopted a conservative assumption of 0.75 LPM for 2 days for neonates with LRI.

Acute medical oxygen need	Neonatal preterm birth	The WHO Handbook of oxygen therapy for children recommends that neonates be given oxygen via nasal prongs with a flow rate of 0.5-1 L/min [42]. Conversely, Graham et al. (2019) observed in 12 hospitals in Nigeria that the flow rate was 0.5-1.0 LPM for neonates [43]. The duration of oxygen therapy was 1.5 days (IQR 0.5–3.5), with minimal variation across age groups. Moreover, the paper reports that the median flowrate of oxygen for preterm birth was 1.0 LPM (IQR 0.5-1.0) and the median duration was 2.5 days (IQR-1.5-4.5).<br><br>Based on this evidence, we adopted a conservative assumption of 0.75 LPM for 3.5 days for preterm birth.
Acute medical oxygen need	Neonatal sepsis	The WHO Handbook of oxygen therapy for children recommends that neonates be given oxygen via nasal prongs with a flow rate of 0.5-1 L/min [42]. Conversely, Graham et al. (2019) observed in 12 hospitals in Nigeria that the flow rate was 0.5-1.0 LPM for neonates [43]. The duration of oxygen therapy was 1.5 days (IQR 0.5–3.5), with minimal variation across age groups. Moreover, the paper reports that the median flowrate of oxygen for neonatal sepsis was 1.0 LPM (IQR 0.5-1.0) and the median duration was 1.5 days (IQR-1.5-3.5).<br><br>Based on this evidence, we adopted a conservative assumption of 0.75 LPM for 2 days for neonatal sepsis.
Acute medical oxygen need	Nutritional deficiencies	The World Health Organization (WHO) recommends an oxygen flow rate of 1-2 LPM for infants and 1-4 LPM for older children [42]. Conversely, Graham et al. (2019) observed in 12 hospitals in Nigeria that the flow rate was 1.0–1.5 LPM for younger children and 1.0–2.0 LPM for older children [43]. The duration of oxygen therapy was 1.5 days (IQR 0.5–3.5), with minimal variation across age groups. Based on these observations, children received approximately 5000 litres of oxygen. However, Howie et al., in an MRC-funded hospital in Gambia, reported that each hypoxemic child received an average of 16000 litres of oxygen [44]. Moreover, a guideline issued by the Ministry of Health of Republic of Rwanda states that children with malnutrition needs to be administered oxygen at a rate of 1 to 2 LPM[48].<br><br>Based on this evidence, we adopted a conservative assumption of 1.3 LPM for 2 days for children with nutritional deficiencies.
Acute medical oxygen need	Other diseases	The World Health Organization (WHO) recommends an oxygen flow rate of 1-2 LPM for infants and 1-4 LPM for older children [42]. Conversely, Graham et al. (2019) observed in 12 hospitals in Nigeria that the flow rate was 1.0–1.5 LPM for younger children and 1.0–2.0 LPM for older children [43]. The duration of oxygen therapy was 1.5 days (IQR 0.5–3.5), with minimal variation across age groups. Based on these observations, children received approximately 5000 litres of oxygen. However, Howie et al., in an MRC-funded hospital in Gambia, reported that each hypoxemic child received an average of 16000 litres of oxygen [44].

		Based on this evidence, we adopted a conservative assumption of 1.3 LPM for 2 days for children with other diseases. We also assumed that the required flow rate for adults with other diseases would be three times that of children, i.e., 4 LPM for 2 days.
Acute medical oxygen need	Sepsis (not otherwise classified) *	The World Health Organization (WHO) recommends an oxygen flow rate of 1-2 LPM for infants and 1-4 LPM for older children [42]. Conversely, Graham et al. (2019) observed in 12 hospitals in Nigeria that the flow rate was 1.0–1.5 LPM for younger children and 1.0–2.0 LPM for older children [43]. The duration of oxygen therapy was 2.8 days, with minimal variation across age groups. Children with acute lower respiratory infections (ALRI) required longer oxygen therapy (mean 3.2 days, median 2.5 days, IQR 1.5–3.5). Based on these observations, children received approximately 5000 litres of oxygen. However, Howie et al., in an MRC-funded hospital in Gambia, reported that each hypoxemic child received an average of 16000 litres of oxygen [44]. Additionally, Rojas Reyes et al. reported an oxygen flow rate of 1-2 LPM for children with lower respiratory infections (LRI) [45].<br><br>Based on this evidence, we adopted a conservative assumption of 1.3 LPM for 3 days for children with LRI or pneumonia. We also assumed that the required flow rate for adults with LRI or pneumonia would be three times that of children, i.e., 4 LPM for 3 days.<br><br>Moreover, The British Thoracic Society guideline suggests that a reservoir mask at 15 LPM be given to septic patients pending the availability of reliable oximetry readings [49].<br><br>Based on this evidence, we assumed the oxygen flow rate and duration would be same for LRI and sepsis (not otherwise classified) *.
Acute medical oxygen need	Trauma/Injury	The World Health Organization (WHO) recommends an oxygen flow rate of 1-2 LPM for infants and 1-4 LPM for older children [42]. Conversely, Graham et al. (2019) observed in 12 hospitals in Nigeria that the flow rate was 1.0–1.5 LPM for younger children and 1.0–2.0 LPM for older children [43]. The duration of oxygen therapy was 1.5 days (IQR 0.5–3.5), with minimal variation across age groups. Based on these observations, children received approximately 5000 litres of oxygen. However, Howie et al., in an MRC-funded hospital in Gambia, reported that each hypoxemic child received an average of 16000 litres of oxygen [44].<br><br>Based on this evidence, we adopted a conservative assumption of 1.3 LPM for 2 days for children with trauma/injury. We also assumed that the required flow rate for adults with trauma/injury would be three times that of children, i.e., 4 LPM for 2 days.
Acute medical oxygen need	Tuberculosis	The World Health Organization (WHO) recommends an oxygen flow rate of 1-2 LPM for infants and 1-4 LPM for older children [42]. Conversely, Graham et al. (2019) observed in 12 hospitals in Nigeria that the flow rate was 1.0–1.5 LPM for

		younger children and 1.0–2.0 LPM for older children [43]. The duration of oxygen therapy was 2.8 days with minimal variation across age groups. Children with acute lower respiratory infections (ALRI) required longer oxygen therapy (mean 3.2 days, median 2.5 days, IQR 1.5–3.5). Based on these observations, children received approximately 5000 litres of oxygen. However, Howie et al., in an MRC-funded hospital in Gambia, reported that each hypoxemic child received an average of 16000 litres of oxygen [44]. Additionally, Rojas Reyes et al. reported an oxygen flow rate of 1-2 LPM for children with lower respiratory infections (LRI) [45].<br><br>Based on this evidence, we adopted a conservative assumption of 1.3 LPM for 3 days for children with LRI or pneumonia. We also assumed that the required flow rate for adults with LRI or pneumonia would be three times that of children, i.e., 4 LPM for 3 days.<br><br>Since we do not have any specific evidence on tuberculosis, we assumed the oxygen flow rate and duration would be same for LRI and tuberculosis.
Acute medical oxygen need	Typhoid	The World Health Organization (WHO) recommends an oxygen flow rate of 1-2 LPM for infants and 1-4 LPM for older children [42]. Conversely, Graham et al. (2019) observed in 12 hospitals in Nigeria that the flow rate was 1.0–1.5 LPM for younger children and 1.0–2.0 LPM for older children [43]. The duration of oxygen therapy was 1.5 days (IQR 0.5–3.5), with minimal variation across age groups. Based on these observations, children received approximately 5000 litres of oxygen. However, Howie et al., in an MRC-funded hospital in Gambia, reported that each hypoxemic child received an average of 16000 litres of oxygen [44].<br><br>Based on this evidence, we adopted a conservative assumption of 1.3 LPM for 2 days for children with typhoid. We also assumed that the required flow rate for adults with typhoid would be three times that of children, i.e., 4 LPM for 2 days.
Acute medical oxygen need	COVID 19	Regarding COVID, we assumed that out of all COVID-related admissions, one-fourth would require ICU admission, while the rest would be treated in general wards [50]. Among ICU admissions, one-fifth would need Mechanical Ventilation, and the remainder would receive High-Flow Nasal Cannula (HFNC) treatment [51]. On average, COVID patients on mechanical ventilation would require 3.2 litres per minute (LPM) of oxygen for 12 days and patients treated with HFNC would need 40 LPM (1:3 oxygen to air ratio) 3 days [51]. In non-ICU setting, we assumed that adult COVID patients would require a higher flow rate of oxygen than adult pneumonia patients. Therefore, in non-ICU setting (originally admitted or stepped down from MV and HFNC), COVID patients would require 5 LPM of oxygen for 4 days [51].<br><br>Consequently, for every 100 COVID patients admitted with hypoxemia, the total oxygen need would be 3,516,480 litres. Thus, the assumed oxygen requirement per adult COVID patient admitted with hypoxemia is 35,165 litres. We also

		assumed that child COVID would have one third of the adult COVID oxygen needs. Therefore, the assumed oxygen requirement per child COVID patient admitted with hypoxemia is 11,722 litres.
Acute surgical (perioperative) oxygen need	Major Surgery with General Anaesthesia	For each major surgery done under general anaesthesia, we assumed that average case duration was 2.5 hours and would likely receive at least 2 litres per minute of oxygen for the duration of the case.<br><br>For each major surgery done under general anaesthesia, we assumed that while in post-operative recovery, each patient was likely to receive oxygen for at least 0.5 hours and likely at a rate of approximately 4 litres per minute of oxygen.<br><br>Thus, for the purpose of estimating oxygen needs in the immediate perioperative period we assumed (2.5h*60min/h*2LPM) +(0.5h*60min/h*4LPM) = 420 litres per case.
Chronic oxygen need	COPD*	The Medical Research Council and American Thoracic Society guidelines recommends administering LTOT at a rate of 2 LPM for 15 hours [52]. We took a conservative assumption of 1 LPM for 1 year, as COPD is a chronic disease and patients need oxygen for an extended time.

Table 15 Country classifications for analysis, by World Bank region and income classification

Country	World Bank Region	World Bank Income	HIC-LMIC
Afghanistan	South Asia (SAR)	LOW INCOME COUNTRY	LMIC
Albania	Europe & Central Asia (ECA)	UPPER MIDDLE-INCOME COUNTRY	LMIC
Algeria	Middle East & North Africa (MNA)	LOWER MIDDLE-INCOME COUNTRY	LMIC
American Samoa	East Asia & Pacific (EAP)	HIGH INCOME COUNTRY	HIC
Andorra	Europe & Central Asia (ECA)	HIGH INCOME COUNTRY	HIC
Angola	Sub-Saharan Africa	LOWER MIDDLE-INCOME COUNTRY	LMIC
Antigua and Barbuda	Latin America & Caribbean (LCR)	HIGH INCOME COUNTRY	HIC
Argentina	Latin America & Caribbean (LCR)	UPPER MIDDLE-INCOME COUNTRY	LMIC
Armenia	Europe & Central Asia (ECA)	UPPER MIDDLE-INCOME COUNTRY	LMIC
Australia	East Asia & Pacific (EAP)	HIGH INCOME COUNTRY	HIC
Austria	Europe & Central Asia (ECA)	HIGH INCOME COUNTRY	HIC
Azerbaijan	Europe & Central Asia (ECA)	UPPER MIDDLE-INCOME COUNTRY	LMIC
Bahamas	Latin America & Caribbean (LCR)	HIGH INCOME COUNTRY	HIC
Bahrain	Middle East & North Africa (MNA)	HIGH INCOME COUNTRY	HIC
Bangladesh	South Asia (SAR)	LOWER MIDDLE-INCOME COUNTRY	LMIC
Barbados	Latin America & Caribbean (LCR)	HIGH INCOME COUNTRY	HIC
Belarus	Europe & Central Asia (ECA)	UPPER MIDDLE-INCOME COUNTRY	LMIC
Belgium	Europe & Central Asia (ECA)	HIGH INCOME COUNTRY	HIC
Belize	Latin America & Caribbean (LCR)	UPPER MIDDLE-INCOME COUNTRY	LMIC
Benin	Sub-Saharan Africa	LOWER MIDDLE-INCOME COUNTRY	LMIC
Bermuda	North America	HIGH INCOME COUNTRY	HIC
Bhutan	South Asia (SAR)	LOWER MIDDLE-INCOME COUNTRY	LMIC
Bolivia	Latin America & Caribbean (LCR)	LOWER MIDDLE-INCOME COUNTRY	LMIC
Bosnia Herzegovina	Europe & Central Asia (ECA)	UPPER MIDDLE-INCOME COUNTRY	LMIC
Botswana	Sub-Saharan Africa	UPPER MIDDLE-INCOME COUNTRY	LMIC
Brazil	Latin America & Caribbean (LCR)	UPPER MIDDLE-INCOME COUNTRY	LMIC
Brunei Darussalam	East Asia & Pacific (EAP)	HIGH INCOME COUNTRY	HIC
Bulgaria	Europe & Central Asia (ECA)	HIGH INCOME COUNTRY	HIC
Burkina Faso	Sub-Saharan Africa	LOW INCOME COUNTRY	LMIC
Burundi	Sub-Saharan Africa	LOW INCOME COUNTRY	LMIC
CÃ´te d'Ivoire	Sub-Saharan Africa	LOWER MIDDLE-INCOME COUNTRY	LMIC
Cabo Verde	Sub-Saharan Africa	LOWER MIDDLE-INCOME COUNTRY	LMIC
Cambodia	East Asia & Pacific (EAP)	LOWER MIDDLE-INCOME COUNTRY	LMIC
Cameroon	Sub-Saharan Africa	LOWER MIDDLE-INCOME COUNTRY	LMIC
Canada	North America	HIGH INCOME COUNTRY	HIC
Central African Rep.	Sub-Saharan Africa	LOW INCOME COUNTRY	LMIC

Country	World Bank Region	World Bank Income	HIC-LMIC
Chad	Sub-Saharan Africa	LOW INCOME COUNTRY	LMIC
Chile	Latin America & Caribbean (LCR)	HIGH INCOME COUNTRY	HIC
China	East Asia & Pacific (EAP)	UPPER MIDDLE-INCOME COUNTRY	LMIC
Colombia	Latin America & Caribbean (LCR)	UPPER MIDDLE-INCOME COUNTRY	LMIC
Comoros	Sub-Saharan Africa	LOWER MIDDLE-INCOME COUNTRY	LMIC
Congo	Sub-Saharan Africa	LOWER MIDDLE-INCOME COUNTRY	LMIC
Cook Islands	East Asia & Pacific (EAP)	HIGH INCOME COUNTRY	HIC
Costa Rica	Latin America & Caribbean (LCR)	UPPER MIDDLE-INCOME COUNTRY	LMIC
Croatia	Europe & Central Asia (ECA)	HIGH INCOME COUNTRY	HIC
Cuba	Latin America & Caribbean (LCR)	UPPER MIDDLE-INCOME COUNTRY	LMIC
Cyprus	Europe & Central Asia (ECA)	HIGH INCOME COUNTRY	HIC
Czechia	Europe & Central Asia (ECA)	HIGH INCOME COUNTRY	HIC
DPR of Korea	East Asia & Pacific (EAP)	LOW INCOME COUNTRY	LMIC
DR Congo	Sub-Saharan Africa	LOW INCOME COUNTRY	LMIC
Denmark	Europe & Central Asia (ECA)	HIGH INCOME COUNTRY	HIC
Djibouti	Middle East & North Africa (MNA)	LOWER MIDDLE-INCOME COUNTRY	LMIC
Dominica	Latin America & Caribbean (LCR)	UPPER MIDDLE-INCOME COUNTRY	LMIC
Dominican Republic	Latin America & Caribbean (LCR)	UPPER MIDDLE-INCOME COUNTRY	LMIC
Ecuador	Latin America & Caribbean (LCR)	UPPER MIDDLE-INCOME COUNTRY	LMIC
Egypt	Middle East & North Africa (MNA)	LOWER MIDDLE-INCOME COUNTRY	LMIC
El Salvador	Latin America & Caribbean (LCR)	UPPER MIDDLE-INCOME COUNTRY	LMIC
Equatorial Guinea	Sub-Saharan Africa	UPPER MIDDLE-INCOME COUNTRY	LMIC
Eritrea	Sub-Saharan Africa	LOW INCOME COUNTRY	LMIC
Estonia	Europe & Central Asia (ECA)	HIGH INCOME COUNTRY	HIC
Eswatini	Sub-Saharan Africa	LOWER MIDDLE-INCOME COUNTRY	LMIC
Ethiopia	Sub-Saharan Africa	LOW INCOME COUNTRY	LMIC
Fiji	East Asia & Pacific (EAP)	UPPER MIDDLE-INCOME COUNTRY	LMIC
Finland	Europe & Central Asia (ECA)	HIGH INCOME COUNTRY	HIC
France	Europe & Central Asia (ECA)	HIGH INCOME COUNTRY	HIC
Gabon	Sub-Saharan Africa	UPPER MIDDLE-INCOME COUNTRY	LMIC
Gambia	Sub-Saharan Africa	LOW INCOME COUNTRY	LMIC
Georgia	Europe & Central Asia (ECA)	UPPER MIDDLE-INCOME COUNTRY	LMIC
Germany	Europe & Central Asia (ECA)	HIGH INCOME COUNTRY	HIC
Ghana	Sub-Saharan Africa	LOWER MIDDLE-INCOME COUNTRY	LMIC
Greece	Europe & Central Asia (ECA)	HIGH INCOME COUNTRY	HIC
Greenland	Europe & Central Asia (ECA)	HIGH INCOME COUNTRY	HIC
Grenada	Latin America & Caribbean (LCR)	UPPER MIDDLE-INCOME COUNTRY	LMIC
Guam	East Asia & Pacific (EAP)	HIGH INCOME COUNTRY	HIC

Country	World Bank Region	World Bank Income	HIC-LMIC
Guatemala	Latin America & Caribbean (LCR)	UPPER MIDDLE-INCOME COUNTRY	LMIC
Guinea	Sub-Saharan Africa	LOWER MIDDLE-INCOME COUNTRY	LMIC
Guinea-Bissau	Sub-Saharan Africa	LOW INCOME COUNTRY	LMIC
Guyana	Latin America & Caribbean (LCR)	HIGH INCOME COUNTRY	HIC
Haiti	Latin America & Caribbean (LCR)	LOWER MIDDLE-INCOME COUNTRY	LMIC
Honduras	Latin America & Caribbean (LCR)	LOWER MIDDLE-INCOME COUNTRY	LMIC
Hungary	Europe & Central Asia (ECA)	HIGH INCOME COUNTRY	HIC
Iceland	Europe & Central Asia (ECA)	HIGH INCOME COUNTRY	HIC
India	South Asia (SAR)	LOWER MIDDLE-INCOME COUNTRY	LMIC
Indonesia	East Asia & Pacific (EAP)	UPPER MIDDLE-INCOME COUNTRY	LMIC
Iran (Islamic Republic)	Middle East & North Africa (MNA)	LOWER MIDDLE-INCOME COUNTRY	LMIC
Iraq	Middle East & North Africa (MNA)	UPPER MIDDLE-INCOME COUNTRY	LMIC
Ireland	Europe & Central Asia (ECA)	HIGH INCOME COUNTRY	HIC
Israel	Middle East & North Africa (MNA)	HIGH INCOME COUNTRY	HIC
Italy	Europe & Central Asia (ECA)	HIGH INCOME COUNTRY	HIC
Jamaica	Latin America & Caribbean (LCR)	UPPER MIDDLE-INCOME COUNTRY	LMIC
Japan	East Asia & Pacific (EAP)	HIGH INCOME COUNTRY	HIC
Jordan	Middle East & North Africa (MNA)	LOWER MIDDLE-INCOME COUNTRY	LMIC
Kazakhstan	Europe & Central Asia (ECA)	UPPER MIDDLE-INCOME COUNTRY	LMIC
Kenya	Sub-Saharan Africa	LOWER MIDDLE-INCOME COUNTRY	LMIC
Kiribati	East Asia & Pacific (EAP)	LOWER MIDDLE-INCOME COUNTRY	LMIC
Kuwait	Middle East & North Africa (MNA)	HIGH INCOME COUNTRY	HIC
Kyrgyzstan	Europe & Central Asia (ECA)	LOWER MIDDLE-INCOME COUNTRY	LMIC
Lao PDR	East Asia & Pacific (EAP)	LOWER MIDDLE-INCOME COUNTRY	LMIC
Latvia	Europe & Central Asia (ECA)	HIGH INCOME COUNTRY	HIC
Lebanon	Middle East & North Africa (MNA)	LOWER MIDDLE-INCOME COUNTRY	LMIC
Lesotho	Sub-Saharan Africa	LOWER MIDDLE-INCOME COUNTRY	LMIC
Liberia	Sub-Saharan Africa	LOW INCOME COUNTRY	LMIC
Libya	Middle East & North Africa (MNA)	UPPER MIDDLE-INCOME COUNTRY	LMIC
Lithuania	Europe & Central Asia (ECA)	HIGH INCOME COUNTRY	HIC
Luxembourg	Europe & Central Asia (ECA)	HIGH INCOME COUNTRY	HIC
Madagascar	Sub-Saharan Africa	LOW INCOME COUNTRY	LMIC
Malawi	Sub-Saharan Africa	LOW INCOME COUNTRY	LMIC
Malaysia	East Asia & Pacific (EAP)	UPPER MIDDLE-INCOME COUNTRY	LMIC
Maldives	South Asia (SAR)	UPPER MIDDLE-INCOME COUNTRY	LMIC
Mali	Sub-Saharan Africa	LOW INCOME COUNTRY	LMIC
Malta	Middle East & North Africa (MNA)	HIGH INCOME COUNTRY	HIC
Marshall Islands	East Asia & Pacific (EAP)	UPPER MIDDLE-INCOME COUNTRY	LMIC

Country	World Bank Region	World Bank Income	HIC-LMIC
Mauritania	Sub-Saharan Africa	LOWER MIDDLE-INCOME COUNTRY	LMIC
Mauritius	Sub-Saharan Africa	UPPER MIDDLE-INCOME COUNTRY	LMIC
Mexico	Latin America & Caribbean (LCR)	UPPER MIDDLE-INCOME COUNTRY	LMIC
Micronesia (Federated States of)	East Asia & Pacific (EAP)	LOWER MIDDLE-INCOME COUNTRY	LMIC
Monaco	Europe & Central Asia (ECA)	HIGH INCOME COUNTRY	HIC
Mongolia	East Asia & Pacific (EAP)	LOWER MIDDLE-INCOME COUNTRY	LMIC
Montenegro	Europe & Central Asia (ECA)	UPPER MIDDLE-INCOME COUNTRY	LMIC
Morocco	Middle East & North Africa (MNA)	LOWER MIDDLE-INCOME COUNTRY	LMIC
Mozambique	Sub-Saharan Africa	LOW INCOME COUNTRY	LMIC
Myanmar	East Asia & Pacific (EAP)	LOWER MIDDLE-INCOME COUNTRY	LMIC
Namibia	Sub-Saharan Africa	UPPER MIDDLE-INCOME COUNTRY	LMIC
Nauru	East Asia & Pacific (EAP)	HIGH INCOME COUNTRY	HIC
Nepal	South Asia (SAR)	LOWER MIDDLE-INCOME COUNTRY	LMIC
Netherlands	Europe & Central Asia (ECA)	HIGH INCOME COUNTRY	HIC
New Zealand	East Asia & Pacific (EAP)	HIGH INCOME COUNTRY	HIC
Nicaragua	Latin America & Caribbean (LCR)	LOWER MIDDLE-INCOME COUNTRY	LMIC
Niger	Sub-Saharan Africa	LOW INCOME COUNTRY	LMIC
Nigeria	Sub-Saharan Africa	LOWER MIDDLE-INCOME COUNTRY	LMIC
Niue	East Asia & Pacific (EAP)	HIGH INCOME COUNTRY	HIC
North Macedonia	Europe & Central Asia (ECA)	UPPER MIDDLE-INCOME COUNTRY	LMIC
Northern Mariana Isl	East Asia & Pacific (EAP)	HIGH INCOME COUNTRY	HIC
Norway	Europe & Central Asia (ECA)	HIGH INCOME COUNTRY	HIC
Oman	Middle East & North Africa (MNA)	HIGH INCOME COUNTRY	HIC
Pakistan	South Asia (SAR)	LOWER MIDDLE-INCOME COUNTRY	LMIC
Palau	East Asia & Pacific (EAP)	UPPER MIDDLE-INCOME COUNTRY	LMIC
Palestine	Middle East & North Africa (MNA)	UPPER MIDDLE-INCOME COUNTRY	LMIC
Panama	Latin America & Caribbean (LCR)	HIGH INCOME COUNTRY	HIC
Papua New Guinea	East Asia & Pacific (EAP)	LOWER MIDDLE-INCOME COUNTRY	LMIC
Paraguay	Latin America & Caribbean (LCR)	UPPER MIDDLE-INCOME COUNTRY	LMIC
Peru	Latin America & Caribbean (LCR)	UPPER MIDDLE-INCOME COUNTRY	LMIC
Philippines	East Asia & Pacific (EAP)	LOWER MIDDLE-INCOME COUNTRY	LMIC
Poland	Europe & Central Asia (ECA)	HIGH INCOME COUNTRY	HIC
Portugal	Europe & Central Asia (ECA)	HIGH INCOME COUNTRY	HIC
Puerto Rico	Latin America & Caribbean (LCR)	HIGH INCOME COUNTRY	HIC
Qatar	Middle East & North Africa (MNA)	HIGH INCOME COUNTRY	HIC
Republic of Korea	East Asia & Pacific (EAP)	HIGH INCOME COUNTRY	HIC
Republic of Moldova	Europe & Central Asia (ECA)	UPPER MIDDLE-INCOME COUNTRY	LMIC
Romania	Europe & Central Asia (ECA)	HIGH INCOME COUNTRY	HIC

Country	World Bank Region	World Bank Income	HIC-LMIC
Russian Federation	Europe & Central Asia (ECA)	HIGH INCOME COUNTRY	HIC
Rwanda	Sub-Saharan Africa	LOW INCOME COUNTRY	LMIC
Saint Kitts and Nevis	Latin America & Caribbean (LCR)	HIGH INCOME COUNTRY	HIC
Saint Lucia	Latin America & Caribbean (LCR)	UPPER MIDDLE-INCOME COUNTRY	LMIC
St Vincent Grenadines	Latin America & Caribbean (LCR)	UPPER MIDDLE-INCOME COUNTRY	LMIC
Samoa	East Asia & Pacific (EAP)	LOWER MIDDLE-INCOME COUNTRY	LMIC
San Marino	Europe & Central Asia (ECA)	HIGH INCOME COUNTRY	HIC
Sao Tome & Principe	Sub-Saharan Africa	LOWER MIDDLE-INCOME COUNTRY	LMIC
Saudi Arabia	Middle East & North Africa (MNA)	HIGH INCOME COUNTRY	HIC
Senegal	Sub-Saharan Africa	LOWER MIDDLE-INCOME COUNTRY	LMIC
Serbia	Europe & Central Asia (ECA)	UPPER MIDDLE-INCOME COUNTRY	LMIC
Seychelles	Sub-Saharan Africa	HIGH INCOME COUNTRY	HIC
Sierra Leone	Sub-Saharan Africa	LOW INCOME COUNTRY	LMIC
Singapore	East Asia & Pacific (EAP)	HIGH INCOME COUNTRY	HIC
Slovakia	Europe & Central Asia (ECA)	HIGH INCOME COUNTRY	HIC
Slovenia	Europe & Central Asia (ECA)	HIGH INCOME COUNTRY	HIC
Solomon Islands	East Asia & Pacific (EAP)	LOWER MIDDLE-INCOME COUNTRY	LMIC
Somalia	Sub-Saharan Africa	LOW INCOME COUNTRY	LMIC
South Africa	Sub-Saharan Africa	UPPER MIDDLE-INCOME COUNTRY	LMIC
South Sudan	Sub-Saharan Africa	LOW INCOME COUNTRY	LMIC
Spain	Europe & Central Asia (ECA)	HIGH INCOME COUNTRY	HIC
Sri Lanka	South Asia (SAR)	LOWER MIDDLE-INCOME COUNTRY	LMIC
Sudan	Sub-Saharan Africa	LOW INCOME COUNTRY	LMIC
Suriname	Latin America & Caribbean (LCR)	UPPER MIDDLE-INCOME COUNTRY	LMIC
Sweden	Europe & Central Asia (ECA)	HIGH INCOME COUNTRY	HIC
Switzerland	Europe & Central Asia (ECA)	HIGH INCOME COUNTRY	HIC
Syrian Arab Republic	Middle East & North Africa (MNA)	LOW INCOME COUNTRY	LMIC
Taiwan	East Asia & Pacific (EAP)	HIGH INCOME COUNTRY	HIC
Tajikistan	Europe & Central Asia (ECA)	LOWER MIDDLE-INCOME COUNTRY	LMIC
Thailand	East Asia & Pacific (EAP)	UPPER MIDDLE-INCOME COUNTRY	LMIC
Timor-Leste	East Asia & Pacific (EAP)	LOWER MIDDLE-INCOME COUNTRY	LMIC
Togo	Sub-Saharan Africa	LOW INCOME COUNTRY	LMIC
Tokelau	East Asia & Pacific (EAP)	UPPER MIDDLE-INCOME COUNTRY	LMIC
Tonga	East Asia & Pacific (EAP)	UPPER MIDDLE-INCOME COUNTRY	LMIC
Trinidad and Tobago	Latin America & Caribbean (LCR)	HIGH INCOME COUNTRY	HIC
Tunisia	Middle East & North Africa (MNA)	LOWER MIDDLE-INCOME COUNTRY	LMIC
Turkey	Europe & Central Asia (ECA)	UPPER MIDDLE-INCOME COUNTRY	LMIC
Turkmenistan	Europe & Central Asia (ECA)	UPPER MIDDLE-INCOME COUNTRY	LMIC

Country	World Bank Region	World Bank Income	HIC-LMIC
Tuvalu	East Asia & Pacific (EAP)	UPPER MIDDLE-INCOME COUNTRY	LMIC
Uganda	Sub-Saharan Africa	LOW INCOME COUNTRY	LMIC
Ukraine	Europe & Central Asia (ECA)	LOWER MIDDLE-INCOME COUNTRY	LMIC
United Arab Emirates	Middle East & North Africa (MNA)	HIGH INCOME COUNTRY	HIC
United Kingdom	Europe & Central Asia (ECA)	HIGH INCOME COUNTRY	HIC
United Rep Tanzania	Sub-Saharan Africa	LOWER MIDDLE-INCOME COUNTRY	LMIC
United States America	North America	HIGH INCOME COUNTRY	HIC
US Virgin Islands	Latin America & Caribbean (LCR)	HIGH INCOME COUNTRY	HIC
Uruguay	Latin America & Caribbean (LCR)	HIGH INCOME COUNTRY	HIC
Uzbekistan	Europe & Central Asia (ECA)	LOWER MIDDLE-INCOME COUNTRY	LMIC
Vanuatu	East Asia & Pacific (EAP)	LOWER MIDDLE-INCOME COUNTRY	LMIC
Venezuela	Latin America & Caribbean (LCR)	UPPER MIDDLE-INCOME COUNTRY	LMIC
Viet Nam	East Asia & Pacific (EAP)	LOWER MIDDLE-INCOME COUNTRY	LMIC
Yemen	Middle East & North Africa (MNA)	LOW INCOME COUNTRY	LMIC
Zambia	Sub-Saharan Africa	LOWER MIDDLE-INCOME COUNTRY	LMIC
Zimbabwe	Sub-Saharan Africa	LOWER MIDDLE-INCOME COUNTRY	LMIC

2.2.2 Estimating current oxygen service coverage

We used the Effective Coverage cascade as the overarching framework for analysis,[4] seeking to quantify the number of people needing oxygen services at each point in the cascade. To populate the estimates we used:

- Needs estimates as described previously (2.2.1), disaggregated by World Bank region.
- Meta-estimates on (i) oxygen availability and (ii) pulse oximeter availability at facilities, disaggregated by ward where possible (presented in the Commission Report with additional details in Supporting data section below).
- Meta-estimates on (i) oximetry coverage to acutely unwell patients and (ii) oxygen coverage to hypoxaemic patients, disaggregated by patient population where possible (presented in the Commission Report with details in Supporting data section below).
- Qualitative synthesis on oxygen service coverage barriers and quality of care.
- Supporting data from published literature (detailed below). This included reliance on the WHO Global health Observatory (GHO) data on care-seeking for children with Acute Respiratory Infection (ARI), as the only standardised reporting of care-seeking available globally.

We constructed an Excel-based model combining this data with additional assumptions, as outlined in Table 16. Initial estimates were discussed at the Commissioner meeting in March 2024, with assumptions tested and refined with additional input from the broader group of Advisors. This generated oxygen service coverage for acute medical conditions and for perioperative care. We then applied the oxygen service coverage estimate for acute medical conditions for people in LMICs with the total LMIC population to estimate the number of people in LMICs without access to medical oxygen services globally.

Table 16 Set-up and assumptions for the Oxygen Service Coverage estimates

Estimate	Assumptions	Data sources
Target population	Derived from Needs (page 33), by World Bank region. Subsequent calculations were all done at a regional level, then summed across all regions for Global (LMIC) estimate.	See Needs Estimates (page 33).
Service contact coverage	% people needing oxygen for acute medical conditions who do not seek care.<br>• SSA 7%<br>• SA 4%<br>• EAP 3%<br>• LAC 3%<br>• MENA 4%<br>• ECA 2%<br>• Surgery 59%<br><br>% people who seek care at Level 1, Level 2, Level 3:<br>• SSA 36%, 50%, 7%<br>• SA 38%, 50%, 8%<br>• EAP 38%, 50%, 9%<br>• LAC 38%, 50%, 9%<br>• MENA 38%, 50%, 8%<br>• ECA 38%, 51%, 9%	WHO Global Health Observatory (GHO) data on care-seeking for children with Acute Respiratory Infection (ARI) – with gap reduced 10-fold to reflect greater care-seeking by severely ill patients. Surgical data from the Lancet Surgery 2030 Commission.[53]<br><br>Triangulated WHO GHO data with:<br>• Prevalence of hypoxaemia in children with ARI in community is <1%, primary care ~9%, hospital ~34%[9]<br><br>• Multi-country study in SSA: median 63% (range14-81%) of hypoxaemic children attending Level 2/3 had

	• Surgery 0%, 20%, 21%<br><br>Of those that seek care at Level 1, % who complete referral to Level 2/3:<br>• SSA 60%<br>• SA 70%<br>• EAP 70%<br>• LAC 80%<br>• MENA 80%<br>• ECA 90%<br>• Surgery N/A	attended Level 1. (unpublished data from Valériane Leroy, AIRE study)<br><br>• Data from studies in SSA: ~60% (range 20% to 100%) of hypoxaemic patient at Level 1 attend Level 2/3 facility.[28 54-57]<br><br>• Data from representative sample of patients at Level 1, 2, 3 in Uganda: one-third of children and adults with hypoxaemia attend Level 1.[54]
Input-adjusted coverage	Uses meta-estimate of oxygen availability at facility, by region. Calculated separately for Level 2 and Level 3 facilities using their respective estimates.	See additional results table from meta-analysis (Table 29)
Intervention coverage	Uses meta-estimate of oxygen coverage to acutely hypoxaemic patients, by region. Calculated separately for Level 2 and Level 3 facilities using their respective estimates.<br><br>Discounted if oximetry is not routinely used:<br>• by 40% if oximetry coverage is <25%<br>• by 15% if oximetry coverage is <50%	See additional results table from meta-analysis (Table 31)
Quality-adjusted coverage	Discounted if qualitative synthesis suggests limitations in timeliness, appropriateness, interruptions, or inadequate monitoring:<br>• by 75% if severe<br>• by 50% if moderate<br>• by 25% if mild	See qualitative synthesis narrative in main report.

SSA, Sub-Saharan Africa; SA, South Asia; EAP, East Asia and Pacific; LAC, Latin America and Caribbean; MENA, Middle East and North Africa; ECA, Europe and Central Asia.

2.2.3 Estimating the cost gap

Data on the cost of medical oxygen was extracted from academic and grey literature, National oxygen road maps, and sourced from individuals working in medical oxygen production. Academic literature was identified from both the Oxygen Service Coverage (section 2.1.3) and Cost of Oxygen Services (section 2.1.5) reviews. Cost of oxygen was extracted as reported by the paper as the: cost per litre, cost per cylinder, cost per m^3, cost per patient treated, or proportion of overall admission cost.

For the cost per cylinder and cost per m^3, we converted this into cost per litre of medical oxygen. In cases where the cylinder size was not reported, we assumed they were using a J cylinder which includes 6800 litres of oxygen. All costs, where applicable, were converted to USD, using the average annual exchange rate from the year of publication, and then adjusted to 2024 USD costs by applying a 2.56% annual inflation rate (the average from 2000-2023) from the year of publication. For data that reported the cost for production only (i.e. did not consider any of the

distribution to facilities), we inflated the cost by 30%. The average cost per litre of oxygen was generated for each World Bank Region, and the overall average used for regions with less than 5 data points - Table 17.[44 58-71]

Table 17: Summary of data used to estimate the cost of oxygen (per litre)

		Number of data points	Average cost 2024 USD/L
Region	East Asia & Pacific	6	0.0049
	Europe & Central Asia	4	0.0023
	South Asia	4	0.0008
	Sub-Saharan Africa	34	0.0051
	North America	0	
	Latin American and the Caribbean	0	
Income	Low income	11	0.0034
	Lower middle income	33	0.0052
	Upper middle income	0	
	High income	4	0.0024
**OVERALL**		48	0.0045

We used the estimated *minimum volume of oxygen required to meet need* from Section 2.2.1d, and the multiplied this by the regional coverage gap – calculated as the difference between the 'target population' (i.e. all those who need oxygen) and the 'quality-adjusted coverage' (i.e. all those who receive quality oxygen services). This gave the minimum volume for unmet need in litres for each region, restricted to LMIC countries only (i.e. we assumed that high-income countries did not have any unmet need). This was multiplied by the regional cost per litre of oxygen as paid by a facility presented in Table 17, to give the *minimum cost to fill the coverage gap*.

Given the minimum volume estimate was based on recommended treatment as per Table 13, we inflated the cost by 95% to reflect actual practice where there are inefficiencies in the system, clinical wastage, and additional consumables. These figures were based on examples from literature and clinical experience within the Commission. For inefficiencies in the system, this refers to leakages in oxygen delivery systems, as well as losses during production, distribution and storage (including major failures).[72] Anecdotally, leakages are regarded as common, with a study by Howie et al. (2009) finding leakage rates between 10-80% for cylinders, depending on the type and quality of the system.[44] We therefore conservatively inflated the minimum oxygen cost by 25% to reflect the mix of high-leakage prone piped systems versus concentrators. For clinical wastage, we applied a 50% inflation, to reflect routine practices of higher flowrates for longer periods than recommended, and treatment of patients without a clinical need for oxygen.[73] Studies in India and Nigeria both reported this magnitude of oxygen saving following quality improvement programmes.[66 74] For consumables, the median oxygen cost to treat patients with non-COVID-19 acute medical conditions in LMICs from published literature was $US 40. A 20% inflation factor was applied, assuming that approximately $US 8 is attributed to consumables, including presence of pulse oximeters, nasal cannulas, masks and staff time. A breakdown by region is presented in Table 18. We then triangulated our cost per patient treated, against published examples as a sense check (Panel 4).

Panel 4: Triangulating the cost of treating patients with oxygen

For a case of paediatric pneumonia, the minimum oxygen need is calculated as 5616 litres (Table 13). Treated in the Sub-Saharan African region, we calculated the cost of minimum unmet need for the oxygen as:

$$5616*0.0051 = \$US\ 28.64$$

When this is inflated for system inefficiency, wastage and consumables, we estimate the real-world cost to be:

$$28.64 + (28.64 * 0.25) + (28.64 * 0.50) * (28.64 + 0.20) = \$US\ 55.85$$

Costs reported in the published literature vary: $38-57 (Hill, 2009 - Gambia), $13–75 (Howie 2009 - Gambia), $88 (Kortz, 2017 - Malawi), $39 (Graham, 2016 – Nigeria), $26 (Huang, 2021 – Uganda). Our estimate falls within the IQR of these values.

Table 18 Calculating the cost to meet the gap in acute medical and surgical oxygen need in LMICs

		Acute medical								Surgical						
	a	b	c	d	e	f	g		h	i	j	k	l	m	
Region	Cost per litre of oxygen *(USD)*	Minimum need *million ltr*	Coverage gap	Cost of unmet need (a*b*c) *million USD*	System efficiency (d*0.25) *million USD*	Clinical wastage (d*0.5) *million USD*	Consum-ables (d*0.2) *million USD*	Total cost (d+e+f+g) *million USD*	Minimum need *million ltr*	Coverage gap	Cost of unmet need (a*h*i) *million USD*	System efficiency (j*0.25) *million USD*	Clinical wastage (j*0.5) *million USD*	Consum-ables (j*0.2) *million USD*	Total cost (j+k+l+m) *million USD*
Middle East & North Africa	0.0045	22854.1	43.6%	$93	$23	$46	$19	$180	5276.0	32.2%	$16	$4	$8	$3	$31
Europe & Central Asia	0.0045	30226.5	46.7%	$64	$16	$32	$13	$125	3814.5		$12	$3	$6	$2	$23
Latin America & Caribbean	0.0045	38607.3	34.0%	$202	$51	$101	$40	$395	6939.1		$21	$5	$11	$4	$41
Sub-Saharan Africa	0.0051	99492.2	8.9%	$803	$201	$401	$161	$1 565	21456.9		$74	$18	$37	$15	$144
East Asia & Pacific	0.0049	136998.6	26.0%	$816	$204	$408	$163	$1 591	26835.4		$88	$22	$44	$18	$172
South Asia	0.0045	194170.5	21.8%	$1 244	$311	$622	$249	$2 426	24809.1		$76	$19	$38	$15	$148
**Overall**	**0.0045**	**522349.1**	**23.0%**	**$3 221**	**$805**	**$1 611**	**$644**	**$6 282**	**41875.6**		**$287**	**$71**	**$143**	**$57**	**$559**

2.3 Country case studies

We used a problem-based political economy analysis (PEA)[75] to understand the following aspects in relation to national oxygen systems: power, stakeholder roles and decision-making processes, governance and accountability mechanisms, regulation and safety, and financing.

Country selection: We purposively selected countries to act as case studies, based on the following considerations: geographical and income diversity; highlight different experiences and aspects of political economy of oxygen systems; members of the Commission have access to key stakeholders. The shortlisting of countries was done through nominations from Commissioners and Advisory Group members, which included the case study focus and practical information on resources available to conduct the study. A shortlist of 11 countries was compiled from which six were decided by the Political Economy working group (Nigeria, Malawi, Peru, Bangladesh, India, Sweden) – Figure 9. Uganda was added later, as we noted a gap in focus on supply chain management.

Figure 9: Countries selected for case studies

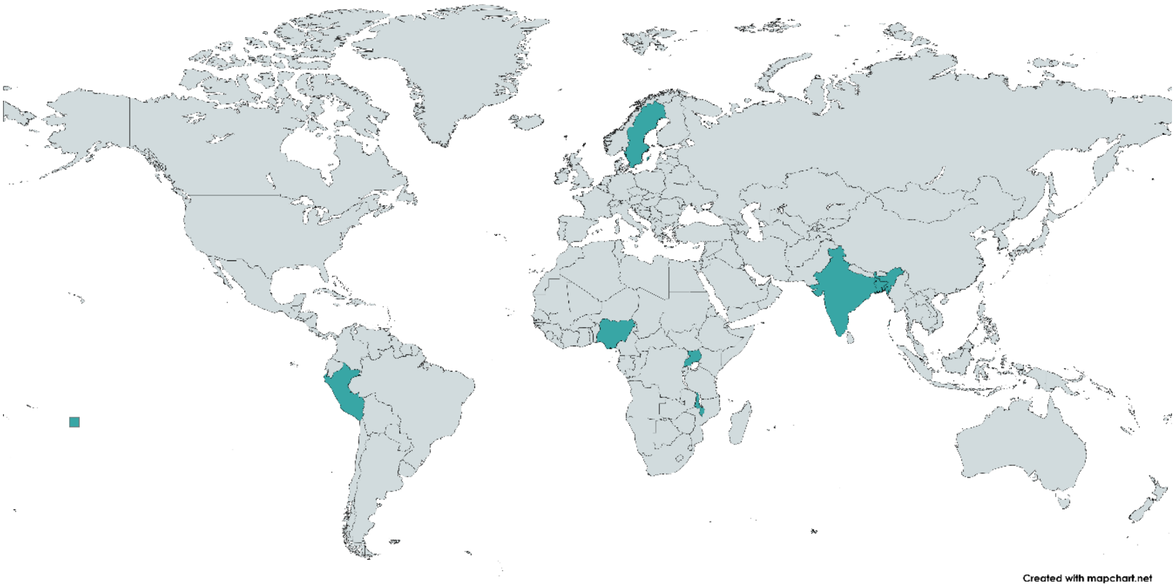

PEA design and methods: Each case study was led by the Commissioner from that country and coordinated by Carina King. A standardised protocol and reporting template was provided to each Commissioner, who then adapted the protocol to reflect their local context and case study focus. The reporting template included three sections: 1) Country context, including demographics, economy, and epidemiology, the health system, and COVID-19 pandemic; 2) Oxygen supply and clinical use landscape; 3) Political economy themes. In general, case studies were based on a desk review, and qualitative key informant interviews – further details are presented in Table 20.

Academic literature was identified through a search in: Medline, Embase, Web of Science and Global Index Medicus. The search was conducted on the 3rd April 2023, and was not updated. The search strategy was developed in Medline (Ovid) in collaboration with librarians at the Karolinska Institutet University Library. For each search concept Medical Subject Headings (MeSH-terms) and free text terms were identified. The search was then translated, in part using Polyglot Search Translator,[76] into the other databases. The strategies were peer reviewed by another librarian prior to execution. No language restriction was applied, and databases were

searched from inception. De-duplication was done using the method described by Bramer et al.,[77] and an additional step was added to compare DOIs.

The full search results are presented in Table 19. The search was run separately for each country, and resulting hits saved as individual country files, which were shared with the case study team in each country for screening and data extraction. Grey literature, national policy documents and budget reports were sourced from online databases, stakeholders and searches of key government, NGO, and CSO webpages. The specific strategy for this was develop by the case study team in each country. The following inclusion and exclusion criteria were applied across all case studies, and then further refined for each country:

- Inclusion criteria:
  - Presents information on the process, adoption, financing, impact or implementation of oxygen systems
  - Published since 2000
  - Any study type with original data, commentaries, and opinion pieces
- Exclusion criteria:
  - Publications where full text cannot be accessed
  - Articles referring solely to industrial oxygen applications

Key informant interviews were planned to gain further insights from a range of stakeholder perspectives. Between 6-15 stakeholders were purposively selected by the case study team in each country, prioritising diversity in roles, interest and power, and including the following groups: government, industry, patient groups, and professional bodies. If there was existing work on-going in this space, or recently completed projects that involved interviews, then new key informant interviews were not conducted.

Interviews were either conducted by teleconference, or in person. Where permitted, interviews were audio-recorded. Given the prominent role of Commissioners in National oxygen initiatives, issues of power, conflicts and bias were critical to reflect upon when determining who would conduct interviews, and for all case study interviews, they were conducted by local research assistants. Interviews were conducted in local languages, and quotes used in the case study reports and Commission were translated to English. All audio and transcription files were stored and archived locally, and only draft and final reports shared with the Executive Committee, Commissioners and Advisory Group.

Synthesizing evidence: While the case studies were conducted individually and analysed their data separately, we used a comparative case study approach to explore cross-cutting lessons.[78] Once all the first drafts of the case studies were completed, one researcher read through each of them multiple times for familiarisation, and then extracted he case study themes and related quotes into an Excel file. These were considered the unit of analysis, which were then summarised into categories and themes. Carina King conducted the data extraction, coding and generated the themes. This was then checked by Amy Gray, and the coding was updated through email exchange discussions. The interpretation and overall thematic areas were presented to Commissioners in a presentation in March 2024, which included 6 of the 7 case study leads.

Table 19: Country case study academic literature search strategy

Interface and search details	Field labels
Interface: Ovid MEDLINE(R) and Epub Ahead of Print, In-Process & Other Non-Indexed Citations and Daily<br>Date of Search: 31 March 2023<br>Number of hits: 3620<br>Comment: In Ovid, two or more words are automatically searched as phrases; i.e. no quotation marks are needed	Field labels<br>• exp/ = exploded MeSH term<br>• / = non exploded MeSH term<br>• .ti,ab,kf. = title, abstract and author keywords<br>• adjx = within x words, regardless of order<br>• * = truncation of word for alternate endings

Ovid MEDLINE(R) ALL <1946 to March 30, 2023>

1	exp Oxygen Inhalation Therapy/	28246
2	exp Oximetry/	16591
3	Oxygen/sd	107
4	Oxygen/st	32
5	Oxygen/tu	3680
6	oxygen*.ti,ab,kf.	671667
7	oximet*.ti,ab,kf.	16396
8	Medical oxygen.ti,ab,kf.	116
9	or/1-8	691298
10	COVID-19/ or exp COVID-19 Testing/ or COVID-19 Vaccines/ or SARS-CoV-2/	222928
11	(coronavirus/ or betacoronavirus/ or coronavirus infections/) and (disease outbreaks/ or epidemics/ or pandemics/)	40224
12	(nCoV* or 2019nCoV or 19nCoV or COVID19* or COVID or SARS-COV-2 or SARSCOV-2 or SARS-COV2 or SARSCOV2 or SARS coronavirus 2 or Severe Acute Respiratory Syndrome Coronavirus 2 or Severe Acute Respiratory Syndrome Corona Virus 2).ti,ab,kf.	335614
13	(longCOVID* or postCOVID* or postcoronavirus* or postSARS*).ti,ab,kf.	81
14	((coronavirus* or corona virus* or betacoronavirus*) adj3 (pandemic* or epidemic* or outbreak* or crisis)).ti,ab,kf.	15253
15	or/10-14	347854
16	9 or 15	1029755
17	Sweden/	81195
18	(sweden* or swedish).ti,ab,kf.	84949
19	Bangladesh/	14154
20	bangladesh*.ti,ab,kf.	20510
21	Malawi/	6627
22	(Malawi* or Nyasaland).ti,ab,kf.	9360
23	Nigeria/	33632
24	Nigeria*.ti,ab,kf.	42246
25	Peru/	10654
26	(peru or peruv*).ti,ab,kf.	17061
27	exp India/	117961
28	india*.ti,ab,kf.	202744
29	or/17-28	448196
30	exp "Health Care Economics and Organizations"/	1677889
31	exp Guidelines as Topic/	172708
32	exp Legislation as Topic/	174737
33	exp Policy/	191489

34	Decision Making/	104139
35	Decision Making, Shared/	1853
36	Decision Making, Organizational/	11238
37	exp Industry/	349838
38	exp Health Planning/ or Delivery of Health Care/	463497
39	(policy* or policies).ti,ab,kf.	361907
40	(legislat* or law*).ti,ab,kf.	200096
41	(social control or regulation* or guideline*).ti,ab,kf.	1493264
42	decision making.ti,ab,kf.	189918
43	((drug or health or healthcare or health care or medical) adj2 (sector* or industr* or market* or financ* or planning* or program* or reform*)).ti,ab,kf.	142218
44	or/30-43	3980854
45	16 and 29 and 44	3620

Interface: Embase

Date of Search: 3 April 2023

Number of hits: 3953

Comment: Emtree is the controlled vocabulary in Embase

Field labels

- /exp = exploded Emtree term
- /de = non exploded Emtree term
- ti,ab,kw = title, abstract and author keywords
- NEAR/x = within x words, regardless of order
- * = truncation of word for alternate endings

No. Query Results

#41	#13 AND #27 AND #40	3953
#40	#28 OR #29 OR #30 OR #31 OR #32 OR #33 OR #34 OR #35 OR #36 OR #37 OR #38 OR #39	561915
#39	india*:ti,ab,kw	277211
#38	'india'/exp OR 'indian'/de	219130
#37	peru:ti,ab,kw OR peruv*:ti,ab,kw	21910
#36	'peru'/de OR 'peruvian'/de	16326
#35	nigeria*:ti,ab,kw	52676
#34	'nigeria'/de OR 'nigerian'/de	47503
#33	malawi*:ti,ab,kw OR nyasaland:ti,ab,kw	10935
#32	'malawi'/de OR 'malawian'/de	9772
#31	bangladesh*:ti,ab,kw	24417
#30	'bangladesh'/de OR 'bangladeshi'/de	21929
#29	sweden*:ti,ab,kw OR swedish:ti,ab,kw	110823
#28	('sweden'/de OR swedish) AND 'citizen'/de	0
#27	#14 OR #15 OR #16 OR #17 OR #18 OR #19 OR #20 OR #21 OR #22 OR #23 OR #24 OR #25 OR #26	4226178
#26	((drug OR health OR healthcare OR 'health care' OR medical) NEAR/2 (sector* OR industr* OR market* OR financ* OR planning* OR program* OR reform*)):ti,ab,kw	173477
#25	'decision making':ti,ab,kw	259190
#24	'social control':ti,ab,kw OR regulation*:ti,ab,kw OR guideline*:ti,ab,kw	1988646
#23	legislat*:ti,ab,kw OR law*:ti,ab,kw	238114
#22	policy*:ti,ab,kw OR policies:ti,ab,kw	424491
#21	'health care delivery'/de	206325
#20	'health care planning'/exp	112231
#19	'industry'/exp	365675
#18	'decision making'/exp	455296
#17	'policy'/exp	325726

#16	'law'/exp	123509
#15	'practice guideline'/de	533695
#14	'health care cost'/exp	334863
#13	#6 OR #12	785625
#12	#/ OR #8 OR #9 OR #10 OR #11	402215
#11	((coronavirus* OR 'corona virus*' OR betacoronavirus*) NEAR/3 (pandemic* OR epidemic* OR outbreak* OR crisis)):ti,ab,kw	15164
#10	longcovid*:ti,ab,kw OR postcovid*:ti,ab,kw OR postcoronavirus*:ti,ab,kw OR postsars*:ti,ab,kw	7747
#9	ncov*:ti,ab,kw OR 2019ncov:ti,ab,kw OR 19ncov:ti,ab,kw OR covid19*:ti,ab,kw OR covid:ti,ab,kw OR 'sars cov 2':ti,ab,kw OR 'sarscov 2':ti,ab,kw OR 'sars cov2':ti,ab,kw OR sarscov2:ti,ab,kw OR 'sars coronavirus 2':ti,ab,kw OR 'severe acute respiratory syndrome coronavirus 2':ti,ab,kw OR 'severe acute respiratory syndrome corona virus 2':ti,ab,kw	369309
#8	('coronavirinae'/de OR 'betacoronavirus'/de OR 'coronavirus infection'/de) AND ('epidemic'/de OR 'pandemic'/de)	11515
#7	'coronavirus disease 2019'/de OR 'covid-19 testing'/exp OR 'sars-cov-2 vaccine'/de OR 'severe acute respiratory syndrome coronavirus 2'/de	322811
#6	#1 OR #2 OR #3 OR #4 OR #5	400226
#5	oximet*:ti,ab,kw	23791
#4	oxygen*:ti,ab,kw	821258
#3	'oxygen'/de	257430
#2	'oximetry'/exp	35192
#1	'oxygen therapy'/exp	104359

Interface: Clarivate Analytics (Web of Science Core Collection)
Editions = A&HCI, ESCI, SCI-EXPANDED, SSCI
Date of Search: 3 April 2023
Number of hits: 4290

Field labels

- TS/Topic = title, abstract, author keywords and Keywords Plus
- NEAR/x = within x words, regardless of order
- * = truncation of word for alternate endings

Note: the *Exact search*-function was used for all the searches

#	Search Query	Results
1	"TS=(oxygen*)"	1360186
2	"TS=oximet*"	17384
3	"#1 OR #2"	1367747
4	"TS=(nCoV* OR 2019nCoV OR 19nCoV OR COVID19* OR COVID OR SARS-COV-2 OR SARSCOV-2 OR SARS-COV2 OR SARSCOV2 OR ""SARS coronavirus 2"" OR ""Severe Acute Respiratory Syndrome Coronavirus 2"" OR ""Severe Acute Respiratory Syndrome Corona Virus 2"")"	410468
5	"TS=(longCOVID* OR postCOVID* OR postcoronavirus* OR postSARS*)"	187
6	"TS=((coronavirus* OR ""corona virus*"" OR betacoronavirus*) NEAR/2 (pandemic* OR epidemic* OR outbreak* OR crisis))"	19278
7	"#4 OR #5 OR #6"	414464
8	"#3 OR #7"	1773100
9	"TS=(policy* OR policies)"	1104049
10	"TS=(legislat* OR law*)"	872196
11	"TS=(""social control"" OR regulation* OR guideline*)"	2070976
12	"TS=""decision making"""	414003

13	"TS=((drug OR health OR healthcare OR ""health care"" OR medical) NEAR/1 (sector* OR industr* OR market* OR financ* OR planning* OR program* OR reform*))"	131861
14	"#9 OR #10 OR #11 OR #12 OR #13"	4298656
15	"TS=(sweden* OR swedish)"	152988
16	"TS=bangladesh*"	41142
17	"TS=(Malawi* OR Nyasaland)"	16742
18	"TS=Nigeria*"	70492
19	"TS=(peru OR peruv*)"	47350
20	"TS=india*"	503100
21	"#15 OR #16 OR #17 OR #18 OR #19 OR #20"	819351
22	"#8 AND #14 AND #21"	4290

Interface: Global Index Medicus web interface<br>https://www.globalindexmedicus.net/<br>Date of Search: 3 April 2023<br>Number of hits: 667	Field labels<br>• Tw = Title, Abstract, Subject

(Oxygen OR oximet* OR "covid 19" OR corona*) AND (Policy OR Policies OR Regulaton* OR guideline* OR Legislat* OR law* OR reform* OR finance* OR industr* OR market* OR sector* Or planning OR program*) AND (Sweden* OR Swedish OR Bangladesh* OR Malawi* OR Nyasaland OR Nigeria* OR Peru OR Peruv* OR india*)

Table 20: Country case study countries and methodological adaptations

Country	Region	Income level	PEA focus	Academic references returned	Key informant interviews	Ethical approval
Bangladesh	South Asia	LMIC	Policy adoption of pulse oximetry into IMCI	779 references	14 interviews conducted	Research Review Committee and Ethics Review Committee of icddr,b (ref: PR 23139)
India	South Asia	LMIC	Data systems for oxygen planning	5209 references	Not conducted	*Not required*
Malawi	Sub-Saharan Africa	LIC	Financing a National action plan	157 references	Not conducted	*Not required*
Nigeria	Sub-Saharan Africa	LMIC	Moving from a National plan to sub-national implementation	756 references	8 interviews conducted	University of Ibadan Research Ethics Committee (ref: UI/EC/23/0181)
Peru	Latin America and Caribbean	UMIC	The role of people and power in oxygen access	631 references	13 interviews conducted	Ethical Review Board of the Universidad Peruana Cayetano Heredia Peru (ref: SIDISI 211379)
Sweden	Europe and Central Asia	HIC	Governance of oxygen in relation to elderly care	703 references	9 interviews conducted	Swedish Ethics Authority (ref: 2023-01793-01)
Uganda	Sub-Saharan Africa	LIC	Oxygen solutions, people and products, including medical oxygen supply chains	709 references	16 interviews conducted	Makerere School of Health Sciences Research Ethics Committee (refs: MakSHSREC-2023-598, MakSHSREC-2023-600, HS3444ES)

HIC = high-income country; UMIC = upper-middle income country; LMIC = lower-middle income country; LIC = low-income country. IMCI = Integrated Management of Childhood Illness guidelines.

2.4 ATMO$_2$S development

The WHO Executive Board resolution on medical oxygen (Ref EB152(4), 1 February 2023) was adopted at the 76th World Health Assembly on the 26th May 2023.[79] This resolution represents a commitment of Member States to establish and implement national oxygen strategies that ensure essential oxygen services are accessible to all people who need them. The resolution contains 20 actions which member states should take, covering production, distribution, and effective clinical use of quality assured medical oxygen at all levels of the healthcare system for both routine and surge/emergency events. Member States are obligated to report back the World Health Assembly (WHA) in 2026, 2028 and 2030.

The Commission therefore set out to support Member States and the WHO with a tool for tracking policy progress, that integrates with best practice indicators for assessing quality oxygen access (*see also the main Commission Report*). The **A**ccess **T**o **M**edical **O**xygen **S**corecard – ATMO$_2$S, aims to both address National level reporting needs to the WHA and support Ministries of Health in setting priorities for their efforts to increase access to medical oxygen. Using a policy scorecard approach has been successful for supporting and monitoring progress for other resolutions, in particular the WHO Framework Convention on Tobacco Control.[80]

Drafting ATMO$_2$S

The starting point for drafting the ATMO$_2$S scorecard was the WHO resolution, where we mapped the 20 Member State actions to the Frost & Reich (2008) framework domains of architecture, availability, affordability and adoption.[81] This mapping was conducted by a smaller group of Executive Members and Commissioners, and then shared within the Political Economy working group for feedback, where we went through four iterative versions of the scorecard, and presented to the wider Commissioners and Advisors group.

In this process we had consultations with WHO and ALMA – the African Leaders Malaria Alliance. WHO consultations were conducted to ensure ATMO$_2$S is aligned with their planned support for Member States and does not duplicate efforts. ALMA provides a valuable example of a scorecard that has been successful in maintaining engagement and commitment from leaders.[82] Discussions with ALMA provided several critical lessons around country ownership and buy-in, building in the ability for the scorecard to evolve over time, and prompting considerations of trust in data.

Finalising and piloting ATMO$_2$S

The 4th iteration of ATMO$_2$S was then taken to consultations with Member States, using two approaches (detailed in section 5). This process allowed Ministry of Health officials to provide feedback on both the overall approach proposed, and the individual scorecard items and scoring mechanisms. We updated individual items based on this feedback. We also asked if Ministries of Health would be willing to complete the scorecard as a pilot – providing input on the clarity of scorecard items and ease of applying the scoring criteria. Overall, five Ministries of Health provided feedback of completed scorecards. We did a final review of ATMO$_2$S following this small pilot, to produce the final tool presented in the **3.1.2 ATMO2S detailed scorecard** section.

2.5 Thanzi la Onse modelling

The model results presented in Panel 7 in the main Commission Report describes the cost-effectiveness of oxygen and routine pulse oximetry strategies under practical health system conditions. These "real-word" conditions used in the model were derived from empirical data from Malawi (i, ii) or sourced from clinical experts (iii, iv), and are as follows: i) HCW diagnostic accuracy for acute lower respiratory tract infections of 75%; ii) HCWs use pulse oximetry, if available, on 90% of children; iii) 85% of children with severe pneumonia complete a referral for inpatient care; iv) 60% of children with oral antibiotics treatment failure seek/receive follow-up care.

The oxygen systems costs include a mix of PSA, cylinders and concentrators, as well as key equipment (monitoring pulse oximeters, ventilators, monitors, resuscitation and suction devices) and distribution costs (e.g. procurement of trucks for cylinder distribution). The mix of sources and costs for these were based on those set out in the Malawi National Oxygen Roadmap, and checked with a group of experts with specific knowledge of the Malawian oxygen ecosystem. For the cost-effectiveness analysis, the equivalent annual costs for the analysis year (2024) were used, and an assumed 25.9% of the oxygen costs were incurred by children with ALRI.

The cost of routine pulse oximetry was estimated by assuming one device per health worker, with the unit cost determined by the number of patients seen at the outpatient department per health worker per day.

The model also included the additional costs for antibiotics, outpatient consultations and inpatient bed-days, associated with changes in care based on diagnosis of pneumonia and hypoxaemia detection. These values were based on the 2015 International Medical Products Price Guide (MSH), and 2010 WHO-CHOICE estimates, inflated to 2024 value. In all scenarios, antibiotic availability was assumed to 100%, and care-seeking for acute lower respiratory tract infections was assumed to be 100%.

3. Tools and checklists

3.1 ATMO$_2$S policy scorecard

3.1.1 ATMO$_2$S definitions

Architecture: The WHO resolution provides guidance for necessary institutional architecture to support a quality medical oxygen system that meets need. It encourages member states to develop policies, plans, rules and standards which ensure a policy environment for all agencies participating in the oxygen system to ultimately support access. The policy communities for medical oxygen access are extensive and require policy engagement and coordination across national and sub-national levels, and between Ministries. Actions here include development of national policies, incorporation of oxygen into national pharmacopoeia and into essential medicine lists and guide upstream public and private agencies to build workforce capacity in quality control, production, distribution, inventory management and use of oxygen and related technologies. This domain is focused at national level policy documents and planning.

Availability: The WHO resolution outlines actions to address oxygen availability including production, forecasting, procurement, and distribution and delivery of quality assured medical oxygen. Several technologies are needed across the production, distribution and delivery cascade, and standards for quality assurance across supply chains (including safe storage and transportation) are expected. This domain initially focusses on establishing national level infrastructure for setting up procurement and distribution systems, including regulation. Once a system is more mature, this domain should be supported with indicators of functional supply.

Affordability: The WHO resolution encourages member states to increase and sustain adequate domestic financing, and promote mutual assistance and support to improve access, use and affordability for medical oxygen across public and private sector agencies. Actions and systems that support end-user affordability, are encouraged from member states and other oxygen industry stakeholders. These start from transparency in pricing and contractual arrangements with industrial actors, to patient-level mechanisms such as subsidies and health insurance. This domain is targeted at National level procedures and policies for universal health coverage, and establishing sustainable financing systems that cover both providers and patients.

Adoption: Broadening the use of oxygen across all healthcare contexts including primary care settings and across relevant therapeutic protocols is part of the Universal Health Coverage agenda. The WHO resolution calls for "*public awareness of the life-saving role of medical oxygen as a treatment for many conditions*" and wide-application of hypoxaemia diagnostic technologies (e.g., pulse oximetry). The WHO resolution also calls for integration of medical oxygen into the infrastructure plans of medical establishments such as hospitals and health centres. Actions to support adoption of oxygen systems at national and sub-national level are encouraged and include end-user and workforce capacity development programmes that extend to biomedical technicians and engineers. It is crucial that member state approaches consider their needs and priorities for effective adoption. The appropriate use of medical oxygen is signposted in the WHO resolution, including safety (such as mitigation of the risk of toxicity, especially in neonates), and preventing wastage. This domain initially focuses on setting up systems for monitoring and evaluation, which can be targeted at national or sub-national levels, and a national level strategy for health workforce planning. These items should be tracked at sub-national levels, evidenced through facility, ward and patient level indicators.

3.1.2 ATMO$_2$S detailed scorecard

Scorecard item	***Definitions and notes***	***Sources of evidence***	***Scoring***
O$_2$ Architecture			
1.1 We include medical oxygen in national **essential medicine lists** (or an equivalent e.g., pharmacopoeia).		The national level essential medicines list, or where this is not available, a national pharmacopoeia or formulary.<br>*Source document required*	3 = Medical oxygen is included within a national essential medicine list, or equivalent.<br>2 = Medical oxygen is included within a national medicine list, but is not considered essential, or contradicts international standards.<br>1 = Medical oxygen is not included in any essential medicine or equivalent list.
1.2 We have included medical oxygen into **national strategic and operational health plans** (e.g., as a National Oxygen Strategy) which includes a realistic costing and financing plan.	This can include both incorporation into a broader strategic health planning document, or as a standalone oxygen roadmap / plan. If planning to develop a national plan, various tools are available to support. Realistic costing and financing should identify where funds are coming from, and be justified with data on oxygen need.	A national level document or strategy that covers the production, distribution and financing of medical oxygen, which covers the current time period (e.g. Oxygen Roadmap, Oxygen Scale-Up plan, Strategic Health Plan)<br>*Source document(s) required*	3 = Fully costed national oxygen strategy for the current time period is available<br>2 = A strategy document exists, but it lacks core elements, such as costing or distribution planning.<br>1 = Medical oxygen is not currently covered by any national health strategy or plan
1.3 We include medical oxygen in our approved national **emergency and disaster preparedness and response plans** and drills.	Emergency and disaster response, in particular Pandemic preparedness plans (PPP) and drills should allow for a range of scenarios, including a respiratory pandemic (such as influenza). However, oxygen need and planned response should be considered beyond respiratory pandemic scenarios.	A PPP which covers the current time period is in place, and medical oxygen is included within this.<br>*Source document(s) required*	3 = A PPP plan exists, and medical oxygen is adequately considered across multiple scenarios.<br>2 = A PPP plan exists, but medical oxygen is insufficiently addressed (e.g. lack of surge planning)<br>1 = A PPP plan is not in place, or if it is, medical oxygen is not included in the planning and response.
1.4 We have **coordination** mechanisms in place across state and non-state actors to support effective partnerships, evidence use, advocacy and leveraged capabilities to	State actors should be considered more broadly than the Ministry of Health. Non-state actors can include: NGOs, multilateral and bilateral organisations, private and industry and charities, CSO, academia. Consider	Qualitative score assignment, with example coordination mechanisms to consider: MOUs with partners; oxygen taskforce has been convened; guidance on managing medical donations; data sharing procedures; prioritisation based	3 = Several currently active mechanisms exist to support coordination, with a person(s)/agency responsible for national oxygen coordination.<br>2 = Some coordination mechanisms in place, but their ability to impact oxygen access is limited (e.g. lack reach, partially active, lack leadership)

increase and sustain oxygen access.	systems for integrating donations into the national oxygen system.	on needs assessments and local research.<br>*Source document(s) required*	1 = No clear mechanisms to support effective coordination of oxygen at a national level
Scorecard item	***Definitions and notes***	***Sources of evidence***	***Scoring***
O_2 Availability			
2.1 We can produce or acquire sufficient medical oxygen supply to meet need during **routine** conditions.	Seasonal variation in oxygen need is included under 'routine conditions'.	Ideally for items 2.1 and 2.2 countries will have a nationally representative situational analysis and resource mapping which can demonstrate both production capacity, effective distribution, and oxygen need in the prior calendar year. Where data on these indicators are not available, score is qualitatively assigned, considering any sub-national/ad-hoc data, for example stock-outs, consumption, hospitalisations.<br>**Coverage and service readiness indicators available.**	3 = Systems are in place to track and report medical oxygen production, distribution and need, and that supply has met need over the prior calendar year.<br>2 = Systems in place to track and report medical oxygen production, distribution and need but supply has inconsistently met need over the prior calendar year.<br>1 = Lack of systems in place to determine if oxygen supply has been sufficient, or multiple instances of oxygen being unavailable to meet needs in the prior calendar year.
2.2 We can promptly increase medical oxygen supply to meet **surges** in demand in national and sub-national-level demands (e.g. outbreaks and disasters).	Major surges are context and temporally dependent and definitions vary by context and scenario. Example scenarios include: multiple overlapping seasonal viruses exceeding routine forecasts; a respiratory pandemic; a major trauma incident with increased critical care/surgery; natural disaster with damaged infrastructure.	Qualitative score assignment, supported by guidelines and policy documents. Example policies, plans and guidelines to consider include pandemic preparedness and response (PPR) plan that covers the current time period; disaster response planning; legal frameworks that allow for temporary conversion of industrial to medical oxygen; mechanisms for emergency release of government funds.<br>*Source document(s) required*	3 = Medical oxygen is included in plans and guidelines for various different scenarios that would require surge capacity, supply has met need over the prior calendar year<br>2 = Medical oxygen is included in plans and guidelines, but for limited scenarios or with insufficient coverage for surge capacity, or supply has inconsistently met need over the prior calendar year<br>1 = Guidelines and policies to support surge capacity are not available or multiple instances of oxygen being unavailable to meet needs in the prior calendar year..
2.3 We have an up-to-date **quantification** of national	This currently focuses on a national level approach to forecasting medical	A system for quantifying oxygen need, allowing for changes in production	3 = Quantification of medical oxygen need is up to date, and includes sub-national variation

medical oxygen need and this is linked to production and procurement.	oxygen need, but sub-national variation should be considered when building this capacity.	capacity and seasonal variation, should be available. The score is qualitatively assigned, considering performance of forecasting and sub-national application.<br><br>**Coverage and service readiness indicators available.**	2 = Quantification of medical oxygen need is conducted, but is out of date or does not link to production and procurement capacity<br><br>1 = Quantification of medical oxygen need is not conducted.
2.4 We have guidance documents and **contracts** to connect production (e.g. gas companies, distributors) and end-user organisations (e.g. hospitals, district health boards) at national and sub-national levels.	Beyond contracts should also be the legal mechanisms that ensure the obligations of contracts are met.	Guidelines for negotiating contracts exist at the National level, and all levels of the health system are covered by this. Evidence of service level agreements can be used where available, and should consider maintenance, training and distribution models.<br><br>*Source document(s) required*<br><br>**Service readiness indicator available.**	3 = Guidelines for negotiating contracts exist at the National level, and all levels of the health system are covered by this<br><br>2 = Guidelines for negotiating contracts exist, but they do not consider different health system level needs<br><br>1 = Guidelines for negotiating contracts do not exist at the National level
2.5 We have regulatory processes for ensuring medical oxygen and oxygen related devices meet **quality and safety standards** and accountability mechanisms for those responsible for supply.	This includes both commercial medical oxygen, industrial oxygen and donations of medical oxygen of equipment.	Regulatory instruments and standards can be demonstrated at relevant National agencies (e.g. drug and medical device authorities) in the first instance. Evidence that they are functional and being implemented in the next level. Facility level processes should also be considered, when assigning the score qualitatively.<br><br>*Source document(s) required*<br><br>**Service readiness indicator available.**	3 = Regulatory instruments and standards for medical oxygen equipment exist and there is active oversight in the supply chain<br><br>2 = Regulatory instruments and standards for medical oxygen equipment exist but they are insufficient or lack oversight<br><br>1 = Regulatory standards for medical oxygen equipment are not present
2.6 We have mechanisms for **safe distribution** of medical oxygen that cover all facilities, to ensure minimal gaps in availability at the point of care	This includes plans for transport and storage at facilities and should include both logistics and safety considerations.	Occupational and environmental health standards are available and cover medical oxygen distribution. Score is assigned qualitatively and can be	3 = Occupational and environmental health cover medical oxygen distribution and distribution systems cover all facilities<br><br>2 = Occupational and environmental health cover medical oxygen distribution but there are gaps in distribution systems

for all patients in need of medical oxygen.		supported by data from sources such as incidence reports and stock-outs.<br><br>*Source document(s) required*<br><br>**Service readiness indicator available.**	1 = Medical oxygen distribution mechanisms are not established, or are not covered by environmental and occupational safety standards
Scorecard item	***Definitions and notes***	***Sources of evidence***	***Scoring***
O_2 Affordability			
3.1 We have **budget** allocations for the key priorities within national medical oxygen plans and are able to access and release funds in a timely way.	Key considerations are how different funding sources are being used, what areas are being prioritised, differentiating between capital and recurrent costs across the production, distribution and delivery, and whether there are accountability mechanisms.	National, sub-national, district or facility budget documents (depending on the way oxygen is procured) should be the basis of the scoring, with both the amount allocated to oxygen systems and the amount spent in the last full budget year considered.<br><br>*National health budget required*	3 = Medical oxygen systems were fully covered in the annual health budget, and funds were largely realised.<br><br>2 = Medical oxygen systems were only partially included in the annual health budget (i.e. key components of the oxygen plan were missing), and funds were not fully realised.<br><br>1 = Medical oxygen systems were not included in the annual health budget, or funds allocated for oxygen were not realised.
3.2 Our **procurement** and tendering processes for medical oxygen supplies, services and devices are transparent and reflect a competitive market price.	This should consider both the processes used for tendering oxygen services, and how they are applied to ensure a transparent and competitive market.	National level guidance on public tenders should be available. Other evidence that should be qualitatively discussed are presence of freedom of information laws for government contracts; price transparency from medical oxygen companies; presence and application of laws that prevent monopolies.<br><br>*Support document(s) required*	3 = National policies on public tenders exist, and mechanisms to ensure they are applied in a transparent and competitive way are functional.<br><br>2 = National policies on public tenders exist, but supportive mechanisms to ensure a competitive market are not available or functional.<br><br>1 = National policies on public tenders do not exist or are insufficient.
3.3 We have mechanisms in place that ensure the provision of medical oxygen services at the point of care is **affordable to providers** and uses appropriate technologies (devices, spare parts, consumables, servicing).	This focuses on how affordable it is for healthcare providers to access an appropriate mix of oxygen technologies for patient care.	Qualitative score assignment, considering the presence of a national formulary for medical devices and drugs, which has an appropriate equipment mix and variations in price points for different use cases. The use of Health Technology Assessments is considered.	3 = National formulary for medical devices and drugs exists and has a systematic process for inclusion of appropriate oxygen supplies and equipment.<br><br>2 = National formulary for medical devices and drugs exists, but key oxygen equipment is missing, or includes inappropriate equipment for the setting.

		Source document(s) required<br>**Coverage and quality of care indicators available.**	1 = National formulary does not exist.
3.4 We have mechanisms in place that ensure oxygen is **affordable to all patients** who need it (e.g. insurance, financial assistance, subsidies).	This is patient centred and should consider universal health coverage policies and financial protections.	Evidence of policies that include universal health coverage, and financial protections (including insurance, subsidies) should be provided and qualitatively discussed. Where available data on out-of-pocket and catastrophic health expenditure should be considered.<br>**Coverage and quality of care indicators available.**	3 = National health system is free at the point of care, or comprehensive financial protections are in place and include oxygen services.<br>2 = Oxygen services can incur out of pocket costs to patients, which are only partially covered or only for parts of the population.<br>1 = Oxygen services are not covered by national insurance or financial protection policies, and can result in catastrophic health expenditure.
O_2 Adoption			
4.1 We have sufficient trained and equipped **healthcare workers** to provide medical oxygen services safely and effectively to patients.	Sufficient refers to existing WHO standard for health workforce of 5 healthcare workers per 1000 population (which is a general standard not specific to oxygen), and should further consider how these health workers are distributed in the health system to ensure oxygen access. While this focuses on a national level, addressing sub-national variation needs to be considered.	Evidence on the number of currently working healthcare workers at a National level. Should also consider whether oxygen and pulse oximetry is included as a core competency in the curriculum for organisations which confer clinical degrees and qualifications.<br>**Coverage, quality of care and service readiness indicators available.**	3 = A minimum of 5 healthcare workers per 1000 population, and these are distributed effectively at sub-national and health system levels, with training on oxygen and pulse oximetry.<br>2 = Below 5 healthcare workers per 1000 population OR training in oxygen and oximetry is not routinely conducted.<br>1 = Below 5 healthcare workers per 1000 AND training in oxygen and oximetry is not routinely, or ever, conducted, or data on healthcare worker density is not available
4.2 We have sufficient trained biomedical **engineers / technicians**, with access to appropriate equipment and supplies, to manage medical oxygen technology safely and effectively.	Sufficient biomedical engineers do not currently have a WHO standard, but the Commission is proposing 0.5 per 10,000 population as a minimum standard. While this focuses on a national level, addressing sub-national variation needs to be considered, as well as systems for different levels of	Evidence on the number of trained and currently working biomedical engineers and technicians at a National level. Where available, facility level coverage can also be used to evidence sufficient coverage. The presence of a capacity-building strategy should also be considered.	3 = A minimum of 0.4 biomedical engineers per 10,000 population, and these are distributed effectively at sub-national, and health system level.<br>2 = Below 0.4 biomedical engineers per 10,000 population, OR considerable sub-national variation in biomedical engineer workforce. Presence of a strategic plan for increasing workforce capacity.

	the health system, and oxygen technology types.	**Coverage and service readiness indicators available.**	1 = Data on biomedical engineer density is not available or is below 0.4 per 10,000 population AND considerable sub-national variation in biomedical engineer workforce. No strategic plan for increasing workforce capacity.
4.3 We have indicators for **monitoring and evaluating** medical oxygen systems, including financing, supply, need, patient access, and quality of care (safety & appropriateness) at national, sub-national and health facility levels.	This item considers both the systems for monitoring oxygen systems, but also the evaluation of oxygen systems based on monitoring data.	Evidence of an M&E framework which includes all dimensions of medical oxygen systems and covers the current time period should be available. The quality of data, extent to which it has been adopted (e.g. indicators included within HMIS systems) and application of this for evaluation (e.g. annual reports) should be qualitatively discussed.<br><br>*M&E framework required*	3 = M&E framework exists and covers all aspects of the medical oxygen system, and has been adopted into routine data systems, with annual evaluation reports.<br><br>2 = M&E framework exists, but it does not cover all elements of the medical oxygen system, or has not been translated into implementation.<br><br>1 = Medical oxygen systems are not currently included in a national health M&E framework.
4.4 We have reliable **public health information** being communicated to the population about medical oxygen services available	The types of materials and their purpose will be context dependent and should respond to local needs – it is important to consider what is relevant in your context. Examples could include: information targeted at the general population to promote uptake of referrals for oxygen treatment; information for healthcare workers to support effective patient communication; tackling mis-information.	Evidence of public health materials that have been checked/updated within the prior 12 months should be provided and scored through qualitative discussion considering their relevance to context, and timeliness. Examples of source materials include: web page with information, awareness campaigns, posters and materials for HCWs.<br><br>*Source material(s) required*	3 = Up to date public health materials available (in multiple languages where appropriate) and tailored to the different needs of communities, patients and providers.<br><br>2 = Public health materials are available, but are out of date, not tailored to the specific needs of the country, or are inaccessible to part of the population.<br><br>1 = Public health materials on medical oxygen are not available through the Ministry of Health or Public Health Agency.
4.5 We have systems in place to ensure **appropriate use** of oxygen among those who need it.	Systems refers to guidelines that guide clinical care, and accountability mechanisms that ensure quality of care (including safe use of medical oxygen). Appropriate use of medical oxygen refers to both the safe delivery of medical oxygen to the patient, and the	Where possible, the score should be data driven. Without data, evidence of systems should be used and discussed qualitatively. Example evidence sources include: national clinical guidelines for medical oxygen use (adapted to different cadres and health system levels); facility	3 = Guidelines exist for clinical use of medical oxygen, and systems for ensuring these are applied safely and effectively are in place.<br><br>2 = Guidelines exist for the clinical use of medical oxygen, but systems/data for ensuring they are safely and effectively used are lacking.

	diagnosis and monitoring of patients using pulse oximetry.	audits on equipment; patient surveys; adverse event data (e.g. neonatal retinopathy).<br>*Source document(s) required*<br>**Quality of care indicators available**	1 = No formal systems or data available to demonstrate appropriate use of oxygen

3.1.3 Intended use of ATMO$_2$S

ATMO$_2$S uses the first person "we" to indicate National level government and was done to promote ownership by member states. The scorecard is intended to be completed and reported by Member States, with the National/Federal Ministry of Health taking the lead and coordinating other relevant government institutions (such as the Ministry of Finance, and Medicine Regulation bodies). Unless otherwise stated, all items in the scorecard should be filled according to the availability of currently relevant policies, plans regulations and guidelines (i.e., they cover the calendar year in which the scorecard is being reported) or use data/budgets from the last full calendar or financial year.

Several indicators reply on qualitatively assessing country capacity in the first instance, acknowledging that many countries will not yet have data systems in place to quantitatively track progress. It is therefore the intention that ***lack of data does not preclude a country from completing ATMO$_2$S***. As countries medical oxygen systems mature, ATMO$_2$S is planned to evolve with it (e.g. if all countries have included oxygen in their essential medicine list or pharmacopeia, then this item can be retired).

We intend for countries ATMO$_2$S results to be made publicly available to support transparency, and more strategic planning from global partners to respond to areas of weakness. However, it is ***not the intention ATMO$_2$S scores to be used in ranking countries against each other***, given the issue raised about different levels of maturity between systems. This would not provide a useful comparison and may discourage open and self-critical reflection.

3.2 Oxygen Coverage Indicators

Selection of oxygen coverage indicators required balancing desirability with feasibility, recognising that some of the ideal or preferred indicators are difficult to obtain outside of research projects and relevant proxies must be used for routine program monitoring and health information system integration. The proposed indicators are intended to be adapted to meet user needs and capacity and should be integrated into existing data collection systems as much as possible (i.e. not duplicating or creating a separate oxygen data system). In keeping with the overall focus of the Commission, the indicators focus on people needing basic oxygen services with pulse oximetry monitoring. These do not cover all the broader health systems requirements for provision of oxygen service coverage but do capture some key aspects (e.g. health workforce, power supply). While these have been framed as indicators for oxygen services, many are equally relevant for other services. We encourage users to seek linkages, and to recognise the utility in oxygen-focussed indicators as useful signal functions for assessing the readiness and quality of other services (e.g. emergency, critical care, paediatric, neonatal, anaesthetic, surgical services).[83-85]

3.2.1 Core oxygen coverage indicators

The *core oxygen coverage* indicators are intended to give an overall picture of the strength of medical oxygen services, capturing multiple aspects of the effective coverage cascade (service contact/accessibility, input-adjusted coverage, and quality-adjusted coverage) - Table 22. They are intended for use in strategic planning, prioritisation, and accountability at subnational, national, and global level.

The **'priority items'** reflect the most important indicators for understanding whether oxygen services are reaching the people who need them. These are indicators that we recommend are integrated into routine health and logistic management information systems. We consider pulse oximetry coverage (the proportion of acutely unwell admitted patients that have documented SpO_2 reading) as the single best indicator of oxygen service coverage at a patient level, recognising it is highly correlated with appropriate oxygen use. We recognise that capturing data on oxygen coverage to hypoxaemic patients is desirable but more challenging, requiring individually matched data on SpO_2 and oxygen use, and high pulse oximetry coverage to provide accurate capture of hypoxaemic patients.

The *'additional items'* provide important information for planning, including demand forecasting, costing, workforce planning, and impact estimates. These may require additional (non-routine) data sources, or modifications to existing data tools, such as workforce administrative data or patient surveys.

Much of these data can be obtained from existing or adapted data collection tools or sources and efforts to obtain these data should be integrated with broader facility readiness, disease incidence, and clinical care data systems. Additional data can be obtained from adapted facility readiness and quality of care assessments (see below).

As much as possible we have selected targets that are consistent with existing global standards or consistently used within the oxygen systems literature. In the absence of global standards for biomedical engineering (BME) workforce we propose a target of ≥0.4 per 10,000 population, which roughly equates to approximately 1 per 100 beds. We derived this using data from the WHO Data Observatory,[86] by calculating the median BME per 10,000 population and median BME per hospital bed, amongst countries meeting the following criteria: 1) complete data on number of BMEs, doctors, nurses and midwives, and hospital beds per 10,000 population in 2017; 2) the country had met the WHO criteria of >44.5 doctors, nurses and midwives per 10,000 population; 3) the BME density was stable across the values reported in 2017, 2015 and 2014 (i.e. did not fluctuate more than 20% between years); 4) none of the years reported a BME density of <0.05 which indicated unreliable data. We used the year 2017 as the most recent year when data on BME workforce was published. Table 21 shows the results of this selection.

Table 21: Calculating the number of biomedical engineers per 10,000 population

Region	Country (income status)	Doctors, nurses, midwives per 10,000 population	Hospital beds per 10,000 population	BMEs per 10,000 population	BME per 100 hospital beds
Latin America & the Caribbean	Chile (HIC)	146.6	21.2	0.36	1.7
	Trinidad and Tobago (HIC)	84.6	24.1	0.29	1.2
	Argentina (UMIC)	65.7	50.2	0.34	0.7
	Mexico (UMIC)	53.2	9.9	0.24	2.4
Europe & Central Asia	Austria (HIC)	122.9	73.7	0.91	1.2
	Denmark (HIC)	145.6	26.2	0.79	3.0
	Estonia (HIC)	100.0	46.1	0.46	1.0
	France (HIC)	145.6	62.3	0.09	0.1
	Greece (HIC)	96.9	42.3	0.28	0.7
	Hungary (HIC)	100.9	70.2	0.41	0.6

	Iceland (HIC)	191.6	30.6	1.68	5.5
	Ireland (HIC)	177.7	29.9	0.69	2.3
	Romania (HIC)	103.5	68.5	0.63	0.9
	Slovenia (HIC)	129.2	44.3	0.84	1.9
	Spain (HIC)	98.1	29.7	0.22	0.7
	Switzerland (HIC)	218.4	46.5	0.14	0.3
	Georgia (UMIC)	116.8	40.0	0.67	1.7
	Montenegro (UMIC)	76.8	38.8	0.24	0.6
Middle East & North Africa	Israel (HIC)	90.3	31.6	2.34	7.4
**Median**				**0.41**	**1.2**

Table 22 **Core indicators for monitoring of medical oxygen coverage** and access to safe, affordable medical oxygen services globally, nationally (and sub-nationally)

	Definition	Target	Example data source
**Pulse Oximetry Coverage**	Proportion of patients presenting to hospital with acute illness or undergoing surgery with SpO_2 documented on triage/admission (or during non-emergency surgery).	Target 50% of countries tracking this by 2030.<br>Target >80%	Clinical audit (+/- routine tracking) of documented pulse oximetry usage.
**Oxygen Production and Storage Capacity**	Mean (and maximum) monthly production volume (Nm3) of medical oxygen, and storage capacity, of each production facility (PSA/VSA or ASU).<br><br>Number of medical oxygen production facilities (PSA/VSA or ASU), including hospital-based facilities.	Target 80% of countries tracking this by 2030.<br><br>Individualised country targets.	National/regional survey of oxygen production facilities (+/- routine tracking)
**Pulse Oximetry and Oxygen Availability**	Number and proportion of acute ward areas in health facilities with a functional pulse oximeter and oxygen supply sufficient to meet patient need in the past month.<br><br>Number and proportion of acute ward areas (as above) that have experienced unavailability of oxygen to a patient who needed it in the past month.	100%<br><br>0%	Facility readiness survey, reported by facility (or ward) head and verified by direct observation (+/- routine tracking).
Pulse Oximetry and Oxygen Service Accessibility	Proportion of the population that can access, within two hours, a health facility that provides low-flow oxygen services with pulse oximetry monitoring.<br><br>Alternative: Proportion of facilities providing basic oxygen services	Target 100%	Health facility mapping with markup of facilities that should provide basic oxygens services (+/- oxygen service readiness).
Hypoxaemia prevalence	Proportion of patients attending a health facility with hypoxaemia (SpO_2<90%) on triage/admission.	Target 50% of countries tracking this by 2030.<br>No target.	Clinical audit (+/- routine tracking) of documented SpO_2.
Oxygen Coverage	Proportion of patients with hypoxaemia (SpO_2<90%) on triage/admission to a health facility who receive oxygen therapy within one hour.	Target 50% of countries tracking this by 2030.<br>Target >80%	Clinical audit (+/- routine tracking) of documented SpO_2 and oxygen usage.
Hypoxaemia-related Mortality	Proportion of patients attending a health facility with hypoxaemia (SpO_2<90%) who die before discharge, or within 30 days.	Target 50% of countries tracking this by 2030.<br>Individualised country targets.	Clinical audit (+/- routine tracking) of documented SpO_2 and outcome.

Clinical Workforce	Number of doctors, nurses, and midwives, per 10,000 population.	≥45 per 10,000 population[87]	Workforce administrative data. Ref: WHO Workforce 2030.[87]
Biomedical Engineering Workforce	Number of biomedical engineers (defined broadly as per WHO[88]) per 10,000 population)	≥0.4 per 10,000 population	In the absence of existing targets we propose a new target based on current coverage and expert consensus (see Table 21 and text explanation).
Protection against Catastrophic Health Expenditure	Proportion of patients receiving medical oxygen services with out-of-pocket expenditure on oxygen services greater than 1% of total annual household expenditure or income.<br><br>Alternatives:<br>Proportion of the population receiving medical oxygen services with household expenditure on health greater than 10% of total household expenditure or income (SDG 2.8.2).<br><br>Proportion of the population receiving medical oxygen services pushed below the $1.90 a day poverty line by household health expenditures (SDG 2.8.2).<br><br>Mean/median out-of-pocket cost for oxygen services incurred by people receiving medical oxygen services.	<5%	Facility readiness survey using patient billing price list for oxygen services.<br><br>Alternatives:<br>Integrated into household survey on out-of-pocket costs for health-care, including oxygen-related fraction.

These indicators are most useful when used and interpreted together as no single indicator provides an adequate representation of oxygen-related service provision in isolation. All targets should be adapted to the local context and given a timeline. Items in **bold** are regarded as highest in terms of priority and feasibility, while others are highly desirable but may be more challenging to assess.

ASU, air separation unit for cryogenic production of liquid oxygen;HRH, human resources for health; PSA/VSA, pressure- or vacuum-swing adsorption oxygen plant; WHO, World Health Organization;. All targets should be adapted to the local context and given a timeline.

3.2.2 Oxygen facility readiness and quality of care indicators

We propose two supplemental sets of indicators for assessing *oxygen service readiness* and *quality of oxygen therapy* in more detail, which are ideally done together. These provide a selection of indicators that capture more detailed infrastructure, input, and process data on how medical oxygen systems are functioning.

The *oxygen service readiness* indicators (Table 23) inform and expand on service readiness items in the core oxygen service coverage indicators and are intended for use in service availability and readiness assessments, local quality improvement initiatives, and research activities. These drill down on the input-adjusted coverage aspect of the effective coverage cascade. The **priority items** reflect essential aspects of equipment availability and workforce capacity at a facility and ward level that should be considered for any readiness assessment. The *additional items* provide important information on common challenges to service readiness, including cost, equipment functionality, availability of normative guidance, and access to spare parts.

The *quality of oxygen therapy* indicators (Table 24) inform and expand on clinical items in the core oxygen service coverage indicators and are intended for use in clinical quality improvement, capacity building, and research activities. These drill down on the quality-adjusted and user adherence-adjusted aspects of the effective coverage cascade. The **priority items** reflect essential elements of oxygen care that should be considered whenever measuring oxygen-related clinical care. These data should be available using a clinical audit of vital signs and documentation, but may require improvement to clinical documentation processes and tools (e.g. standardised observation chart). The *additional items* provide important information on the quality of care (e.g. appropriateness, rational use) and common challenges to providing high quality care (e.g. acceptability, cost) but may not be so easy to get from retrospective audits.

Table 23 Key indicators for assessing facility service readiness to provide safe, affordable medical oxygen services

These data can be self-reported by facility staff and used for self-assessment and monitoring. For higher data quality, data should be obtained from the most qualified person and confirmed through direct observation by an independent observer. Technology functionality requires direct testing.

	Definition	Target	Comment
**Service availability**	Proportion of acute care ward areas (including emergency department, inpatient wards, and operating theatre) with pulse oximetry and low-flow oxygen service availability.	Target >90%	This is a composite of pulse oximetry and oxygen availability.
**Pulse oximeter availability**	Number and ratio of functional pulse oximeters to beds (or patients), by ward.	Low-acuity wards: ≥1:10<br>Mixed-acuity wards: ≥1:5<br>High-acuity wards: ≥1:2 (ideally 1:1 continuous)	Quality of device is important. Functionality require ability to get a reading at the time of assessment.
**Oxygen availability**	Number and ratio of functional oxygen delivery points to beds (or patients), by ward.[1]	Low-acuity wards: ≥1:10<br>Mixed-acuity wards: ≥1:5<br>High-acuity wards: ≥1:1	Quality of device is important. Functionality requires medical oxygen provision at the time of assessment.
**Clinical health workforce**	Ratio of nurses to patients (or beds), by ward.	Low-acuity wards: ≥1:10 day shift, 1:20 night shift<br>Mixed-acuity wards: ≥1:7 day shift, 1:14 night shift<br>High-acuity wards: ≥1:4 day shift, 1:8 night shift	Nurses (or similar cadre) should be adequately trained and skilled to use pulse oximetry and oxygen safely and appropriately.
**Biomedical engineering workforce**	Number of biomedical engineering workforce with responsibility and capacity to manage pulse oximeters, medical oxygen sources/distribution systems, delivery devices and other oxygen-related technologies.	Tertiary: >2 onsite<br>Secondary: minimum 1 onsite<br>Primary: minimum 1 on call<br><br>The national target of 0.4 per 10,000 population roughly equates to 1.2 per 100 beds and 1 per 250 HCWs.	Biomedical engineer (or similar cadre) should be adequately trained and skilled to maintain pulse oximeter and oxygen equipment safely and appropriately.
Oxygen supply stockout	Number (and proportion) of days in past month that oxygen supplies (including backup) or delivery equipment has been unavailable for a patient who needed it in this ward area.	Target 0%	Primary oxygen supply source should work 24/7 in extreme environmental conditions.

[1] Oxygen delivery points can include wall oxygen outlets, flowmeter assembly outlets, or individual delivery from oxygen cylinders or concentrators.

Backup oxygen supply	Proportion of wards with access to secondary and/or backup oxygen supply in case of failure of primary source	Target 100%	Back-up supply should be active automatically or with minimal human involvement or time delay.
Out-of-pocket cost to patients	Oxygen-related out-of-pocket costs as a proportion of total out-of-pocket costs for a prototypical care episode (e.g. 3-day admission with pneumonia, C-section delivery)	Target: <25%	Costs should be reasonable for the local health financing context and always avoid catastrophic health expenditure.
Cost and Contracts	Presence of up-to-date contracts for oxygen supply +/- maintenance with relevant supplier(s) (governmental or non-governmental).<br><br>Proportion of total annual facility budget spent on direct oxygen supply costs, including:<br>- cost of cylinder refill, including delivery<br>- cost of oxygen equipment purchases (cylinders, concentrators)<br>- cost of spare parts/maintenance<br>- cost of liquid oxygen supply	Target: 100%<br><br>No target	All facilities should be covered by a long-term oxygen supply plan, regardless of the financier or supplier detail. These indicators are intended to provide additional clarity on management processes for oxygen.
Technology management and safety	Presence of pulse oximeters and medical oxygen sources and distribution equipment in *facility equipment inventory*.<br><br>Presence of *maintenance schedule, oxygen analyser and basic tools* for cleaning, preventive maintenance, and simple repairs on oxygen equipment.<br><br>Number and proportion of pulse oximeters | oxygen concentrators | oxygen distribution systems (including manifolds and piping) that have received preventive or corrective maintenance in the previous 6 months.<br><br>Number and proportion of major oxygen sources tested for oxygen purity in past 6 months.<br><br>Presence of oxygen-related safety precautions for all oxygen devices and locations (e.g. secure housing for cylinders, fire	Target 100%	All facilities should have oxygen-related technology integrated into equipment management plans. These indicators are intended to provide additional clarity on equipment management processes for oxygen.

	awareness and extinguisher, ramps and wheels for moving cylinders, safety checks on oxygen outlets/manifolds/etc.).		
Clinical use of oxygen guidelines	Proportion of wards with clinical guideline or protocol containing oxygen-related guidance.	Target 100%	Ideally oxygen-related clinical guidance is integrated into core clinical protocols and available on the ward (digital or print).
Higher respiratory support	Proportion of wards providing higher respiratory support (e.g. NICU, ICU, OT) with capacity to mix air-oxygen for individual patients	Target 100%	Reported by ward doctor, confirmed by direct observation.
Technology functionality	Number and proportion of *pulse oximeters* that are functional and ready for use (power on, probe attached), by ward.<br><br>Number and proportion of wall oxygen outlets that are functional and ready for use (connected to oxygen supply with regulator/flowmeter apparatus attached), by ward.<br><br>Number and proportion of *oxygen cylinders* that are functional and ready for use (not empty with regulator/flowmeter apparatus attached), by ward.<br><br>Number and proportion of *oxygen concentrators* that are functional and ready for use (power on, produce oxygen purity ≥85%), by ward.	Target 100% of those in active use and >80% of all those in facility.	Direct observation of presence and basic functionality.
Supporting infrastructure	Number of power outages per month, by ward.<br>Mean and longest, duration of power outages over the previous 3 months, by ward.		Reported by head of ward, confirmed with engineering lead.

These indicators are most useful when used and interpreted together as no single indicator provides an adequate representation of oxygen-related service provision in isolation. All targets should be adapted to the local context and given a timeline. Items in **bold** are regarded as highest in terms of priority and feasibility, while others are highly desirable but may be more challenging to assess.

HCW, healthcare worker; ICU, intensive care unit; NICU, neonatal intensive care unit; OT, operating theatre.

Table 24 Key indicators for **measuring quality of medical oxygen services to patients**, including proposed minimum targets

	**Definition**	**Target**	**Comment**
**Assessment for hypoxaemia**	Proportion of patients presenting with acute illness or undergoing surgery with SpO_2 documented on triage/admission (or during surgery).	Target >80%	Typically obtained from clinical audit of documented vital signs and clinical care.
**Timeliness**	Proportion of patients with hypoxaemia (SpO_2<90%) on admission who receive oxygen therapy within 1 h.	Target >80%	
**Monitoring**	Proportion of patients receiving oxygen therapy who have SpO_2 documented at least 3 times per day (and continuously for patients receiving additional respiratory support or undergoing surgery).	Target >90%	
Hypoxaemia prevalence	Proportion of patients admitted to a health facility with hypoxaemia (SpO_2<90%) on triage/admission.	No target – reflects magnitude of oxygen need	
Appropriate delivery	Proportion of patients receiving oxygen therapy who receive it via an appropriate delivery device and flowrate (as per guidelines)	Target >80%	
Rational use	Proportion of patients receiving oxygen therapy who have severe or moderate hypoxaemia (SpO_2<94%) or other documented indication.	Target >80%	
Cessation	Proportion of patients receiving oxygen therapy who have documented SpO_2≥90% within 2 h of final cessation.	Target >90%	
Hypoxaemia-related outcomes	Proportion of patients admitted with hypoxaemia (SpO_2<90%) who die before discharge, or 30 days.<br><br>Proportion of patients admitted with hypoxaemia (SpO_2<90%) who (i) are transferred out, (ii) discharge against medical advice, (iii) discharge well.	Target 50% of countries tracking this by 2030. Individualised country targets.	
Quality of services to patients who die	Proportion of patients who die, who have had pulse oximetry measurement on triage/admission, received oxygen if hypoxaemic, and had pulse oximetry monitoring at least 3 times per day.	Target >90%	
Safety in at-risk groups	Proportion of patient-hours spent with SpO_2 in target range for at-risk populations (preterm neonates, adults with COPD, others as per guidelines)	Target >50%	This may require additional clinical tracking chart or continuous oximetry data.

Acceptability and adherence	Proportion of patients receiving oxygen therapy or with hypoxaemia (SpO2<90%) on admission who refuse oxygen or discharge against medical advice.<br><br>Alternative:<br>Proportion of patients receiving oxygen therapy or with hypoxaemia (SpO2<90%) on admission who complete the full recommended course of treatment	Target: <5%<br><br><br><br><br>Target >90%	This may require data from patient exit survey.
Out-of-pocket cost to patients	Oxygen-related out-of-pocket costs as a proportion of total out-of-pocket costs for a given care episode.<br><br>Median out-of-pocket cost for oxygen-related services incurred by patients requiring oxygen therapy	Target: <25%<br><br><br>Target: N/A (locally defined)	This may require data from cashier review or patient exit survey.
Workforce capacity	Proportion of healthcare workers who have received training on pulse oximetry and low-flow oxygen therapy within the previous 5 years (pre-service or in-service, including integrated in other training modules).<br><br>Proportion of healthcare workers demonstrate competency in use of pulse oximetry and basic oxygen therapy according to guidelines.	Target >90%	This requires some sort of healthcare worker survey or observed practice.

These indicators are most useful when used and interpreted together as no single indicator provides an adequate representation of the quality of oxygen service provision in isolation. All targets should be adapted to the local context and given a timeline. Items in **bold** are regarded as highest in terms of priority and feasibility, while others are highly desirable but may be more challenging to assess.

COPD, chronic obstructive pulmonary disease; N/A, not applicable.

3.3 Oxygen-related tools, and standards

3.3.1 Mapping of existing oxygen service coverage tools against proposed indicators

The following table maps existing data collection tools against the Commission's proposed oxygen service coverage indicators.

Indicator category			Oxygen coverage (Table 22)										Oxygen Service Readiness (Table 23)														Oxygen quality of care indicators (Table 24)											
Indicator			**Priority**			*Additional*							**Priority**					*Additional*									**Priority**			*Additional*								
Survey	F: facility survey C: clinical audit; -Ward FW OPD ED Ped Neo Ad CC OT Mat*	Total questions	**Pulse oximetry coverage**	**Production & storage capacity**	**Availability & stockout of O_2**	*Accessibility to medical O_2 services*	Hypoxaemia incidence	*O_2 coverage*	*Hypoxaemia-related mortality*	*Clinical health workforce*	*Engineering health workforce*	*Catastrophic health expense*	**Service availability**	**Pulse oximeter availability**	**Oxygen availability**	**Clinical health workforce**	**Engineering health workforce**	*Oxygen supply stockout*	*Out-of-pocket cost to patients*	*Cost and Contracts*	*Technology management and safety*	*Clinical use of oxygen guidelines*	*Backup oxygen supply*	*Higher respiratory support*	*Technology functionality*	*Supporting infrastructure*	**Assessment for hypoxaemia**	**Timeliness**	**Monitoring**	*Appropriate delivery*	*Rational use*	*Cessation*	*Quality of services to patients who die*	*Safety in at-risk groups*	*Acceptability and adherence*	*Out-of-pocket cost to patients*	*Workforce capacity*	***Number of indicators tool assesses***
**Critical Care/Emergency/Trauma/Surgery/Anesthesia Services assessment tools**																																						
AFEM ECAT [89]	F-CC	71																																				0
UCSF/CHESA CC FLA[90]	F-CC	70																																				4
< ECCS tool [91]	F-All	1057																																				7
WHO/IATSIC G_EsTC [92]	F-FW	260																																				0
WHO NA&EF 4EEEER[93]	F-ER	28																																				0
# WHO TSAAEESC[94-97]	F-FW	185																																				4
+WHO/AFEM HEAT [98]	F-FW ED	223																																				4
WHO/PGSSC /LCoGS SAT [99 100]	F- FW, OT	180																																				6
~SOS PIPES [101-103]	F-OT	105																																				1

WFSA_AFAT [104]	F-OT, CC	240	6
**COVID-19 readiness assessment tools**			
†WHO C19 RHRC [105]	F	98	0
†WHO BMES C-19[106]	F-All	123	3
**Pediatric essential emergency and critical care**			
EnvScan ObsScan [107]	F&C-ED, Ped	457 131	9
**Pediatric services quality of care**			
WHO IQPC appendix 4[108]	C-Ped	29	1
WHO IPC Annex 8 indicators[109]	F -Ped	106	2
WHO EURO HC4C[110]		>200	9
**Reproductive/Maternal/Obstetric, neonatal, child, adolescent health assessment tool**			
WHO/SEARO AT4HC [111]	F-ED/ Mat/Ped/Neo	>200	2
WHO/EURO HC4M&NB [112]	F-Neo Mat	>200	6
AMDD NA EmONC			
**Broad facility assessment tool**			
World Bank SDI	F-		0
WHO HHFA [113]	F	>500	5
WHO SARA [114]	F	>500	3
WHO HeRAMS [115]	F	134	2
DHS Prog SPA [116]	F	955	5
WHO/UNICEF PHCMF&I[117]	F	96	1
**Oxygen focused tool**			
§ EPIC/USAID O_2 &C-19 tools[118]	F-CC, Ped	150 115	7
WHO SEARO IMAI O_2 [119]	F-CC	135	11

‡CHAI/CHAI MOXY [120]	F&C-All	23 19																																				17
O_2 SICW [121]	F-Ped	130																																				9
^PATH O_2DT BAS[122]	F	260																																				8
FreO_2 Uganda [123]	F&C	140 55																																				17
Nigeria OIP [69 74 124 125]	F&C	40 189																																				20
INSPIRING [126 127]	F&C-ED,Ped	41 27																																				14
Nabwire 2018[128]	F-Ped	30																																				5
**Number of tools that assess indicator**			7	4	13	4	5	4	2	9	1	3	13	8	12	17	5	4	3	2	3	3	12	6	3	13	5	0	4	5	3	1	2	0	2	3	7	

* Facility-wide (FW); Outpatient (OPD); Emergency (ED); Paediatric/children's (Ped); Neonatal/nursery/special care/NICU (Neo); Adult/medical (Ad); Critical care/HDU/ICU/CCU (CC); Operating Theatre/surgical theatre/recovery (OT); Maternity/obstetric/delivery/newborn resuscitation areas (Mat).

< see also Emergency Care Capacity Score (ECCS) [129]

\# - Part of the WHO Integrated Management for Emergency & Essential Surgical Care (IMEESC) toolkit; see also Harvard Humanitarian Initiative survey[130] Vanderbilt University Medical survey[131] , INTACT[132] and SaLTS[131] which are based on the WHO Global Initiative for Emergency and Essential Surgical Care (GIEESC) TSAAEESC

\+ - see also Malawi Emergency and Critical Care survey (MECC)[133]; and Emergency Care Capacity Score (ECCS)[134] which are based on the WHO/AMEF HEAT

~ - see also Pedi-Pipes, Neuro-PIPES[135] and INTACT[132 135] which are based on the SOS PIPES

† A module from the WHO Suite of health service capacity assessments in the context of the COVID-19 pandemic (Interim guidance 2 November 2020)[136] which replaces WHO Harmonized health service capacity assessment in the context of the COVID-19 pandemic (Interim guidance 31 May 2020) [137]; and contains other modules that mention oxygen 1. Diagnostics, therapeutics, vaccine readiness, and other health products for COVID-19; and 2. Continuity of essential health services: Facility assessment tool

§ EPIC Oxygen and COVID-19 Response Rapid Assessment Tool: Hospital Facilities and Oxygen and COVID-19 Response Rapid Assessment Tool: Primary Care Facilities are mapped in this table, there are two other tools Liquid Oxygen (LOX) Systems: Assessment Tool A – USAID Mission Survey and Liquid Oxygen (LOX) Systems: Assessment Tool B – National Stakeholders

‡ The Clinton Health Access Initiative (CHAI) Facility Assessment Tool: Facility Oxygen Availability Assessment and Patient Record Review Tool: Inpatient Case Note Review tool have both been adapted for CHAI MOXY and various assessments in Cambodia, Ethiopia, India, Kenya, Laos PDR, Liberia, Nigeria, Rwanda, and Uganda.

^ A component of the PATH Oxygen Delivery Toolkit: Resources to plan and scale medical oxygen

3.3.2 Additional oxygen-related standards and tools

Checklists/Standards/Guidelines

- World Health Organization Oxygen therapy for children: a manual for health workers 2016
- World Health Organization Generic Essential Emergency Equipment list 2012
- World Health Organization WHO Model List of Essential Medicines for Children - 9th list, 2023
- World Health Organization WHO Model List of Essential Medicines - 23rd list, 2023
- World Health Organization Safe Childbirth checklist 2015
- World Health Organization Surgical Safety Checklist 2009
- World Health Organization Guidelines for Safe Surgery 2009
- World Health Organization Pocket Book of Hospital Care for Children second edition, 2013
- World Health Organization Standards for improving the quality of care for small and sick newborns in health facilities 2020
- World Health Organization Standards for improving the quality of care for children and young adolescents in health facilities 2018
- World Health Organization Standards for improving quality of maternal and newborn care in health facilities 2016
- World Health Organization IMAI district clinician manual: hospital care for adolescents and adults. Guidelines for the Management of common illnesses with limited resources 2011
- World Health Organization WHO Package for Essential Non-communicable Diseases (PEN) 2020
- World Health Organization Clinical care of severe acute respiratory infections – Tool kit 2022
- World Health Organization & UNICEF The integrated Global Action Plan for Prevention and Control of Pneumonia and Diarrhoea 2013
- World Health Organization Paediatric emergency triage, assessment and treatment: care of critically-ill children 2016
- Centers for Disease Control and Prevention (CDC) Comprehensive Hospital Preparedness Checklist for Coronavirus Disease 2019
- World Health Organization & International Committee of the Red Cross Basic Emergency Care: approach to the acutely ill and injured 2018
- World Health Organization & World Federation of Societies of Anaesthesiologists International Standards for Safe Practice of Anaesthesia 2018
- Association of Anaesthetists of Great Britain & Ireland Recommendations for standards of monitoring during anaesthesia and recovery 2021
- Summary of WHO symposium on meeting the global needs for oxygen and respiratory care: recent learning, current knowledge and future direction, 4 October 2023, Toronto, Canada

Procurement/Forecasting/Estimating/ Technical guidance/Supply chain monitoring tools:

- PATH oxygen delivery toolkit Resources to plan and scale medical oxygen
  - Oxygen is Essential: A Policy and Advocacy Primer
  - Health Facility Standards Guide

- Consumption Tracking Tool
    - ODT_Consumption_Tracking_Tool
- Procurement Guide
- Quantification and Costing Tools
    - Quantification and Costing Tool: Oxygen Delivery Sources Excel-based tool
    - Quantification and Costing Tool: Pulse Oximetry Devices Excel-based tool
    - Quantification and Costing Tools for Oxygen and Pulse Oximetry
- Reference Pricing Guide
- Electricity Planning Guide
- Asset Management Guide
- Global Financing Facility Medical Oxygen Investment Guide

- PATH Pulse oximetry primer May 2024
- WHO COVID-19 Essential Supplies Forecasting Tool (COVID-ESFT) v4.1 (replaced COVID-19 Essential Supplies Forecasting Tool v4.0)
- WHO Priority medical devices list for the COVID-19 response and associated technical specifications 2020
- Oxygen Supply and Demand Calculator – Open Critical Care [World Federation of Societies of Anaesthesiologists (WFSA), University of California, San Francisco, Open Critical Care, Sustaining Technical and Analytical Resources (STAR) project]
    - O_2 Demand
    - **O_2 Supply**
    - SpO_2 to PaO_2
    - Cylinder Duration
    - Cylinder Size
    - Consumables & Cost **Beta**
- Oxygen system planning tool – UNICEF
- Oxygen Market Dashboard – UNICEF
- Medical gas consumption calculator v9.20 by Rob Chatburn
- Medical gas flow limitations calculator v1.21 by Rob Chatburn
- Predicting Surge Requirements for Medical Gas Consumption by Rob Chatburn
- Quality Assurance Practices for Medical Oxygen Systems Technical Resource for Distribution- and Facility-Level Medical Oxygen Systems US Agency for International Development (USAID) Medicines, Technologies, and Pharmaceutical Services (MTaPS) Program 2023
- WHO-UNICEF Technical specifications and guidance for oxygen therapy devices 2019
- WHO MEDEVIS https://medevis.who-healthtechnologies.org/ 2024
- WHO Oxygen Needs-Gap Facility Estimator 2024
- WHO Foundations of medical oxygen systems 2023
    - WHO Foundations of medical oxygen systems annex A: Technical considerations for the procurement of oxygen generator plants
    - WHO Foundations of medical oxygen systems annex B: Site evaluation for oxygen generator plants
    - WHO Foundations of medical oxygen systems annex C: Site readiness for oxygen generator plants
    - WHO Foundations of medical oxygen systems annex D: Commissioning report for oxygen generator plants
    - WHO Foundations of medical oxygen systems annex E: Oxygen supplier's mapping

- WHO Developing key performance indicators for the medical oxygen ecosystem through Delphi consensus 2023 KPIs for monitoring global investments in medical oxygen
- WHO Technical consultation on oxygen access scale-up for COVID-19 2021
- WHO Technical specifications for invasive and non-invasive ventilators for COVID-19: interim guidance 2020
- WHO Technical specifications for pressure swing adsorption (PSA) oxygen plants. Geneva 2020
- WHO Technical specifications for oxygen concentrators 2015
- WHO Interagency List of Priority Medical Devices for Essential Interventions for Reproductive, Maternal, Newborn and Child Health 2016
- WHO Access to Oxygen Initiative
    - Estimating electrical operational costs of an on-site oxygen generation system
    - Estimate total cost of ownership, including capital and operational costs, of a newly acquired oxygen generation system along its lifespan
    - Generate a preventive maintenance calendar and estimate the time to next required maintenance for an on-site oxygen generation system, including the diesel generator
    - Assess the actual and expected operational performance of the booster compressor, determine the volume of oxygen produced and translate to number of cylinders filled
    - Conversion and equivalence of common metrics / units used when measuring oxygen volume
- American Society for Health Care Engineering (ASHE) Medical Air and Oxygen Capacity Assessment Tool
- PAHO Good practices in the rational and effective use of oxygen 2021
- Build Health International Oxygen Technical Support
- Global Oxygen Alliance - Working toward affordable, equitable medical oxygen for everyone who needs it
- Medical Oxygen - Unitaid
- Home - Access to Oxygen Resource Library (a2o2resources.org)

4. Supporting data

4.1 Detailed oxygen need data and additional analyses

The following section contains additional analysis on oxygen need based on Global Burden of Disease data for 2021. Analyses of other years is available on request.

Table 25 Numbers of patients (millions) needing medical oxygen for acute medical, surgical, long-term oxygen therapy, and COVID-19, by incomes and World Bank region*, and minimum volume of oxygen required to meet need (million cubic metres/Nm³), 2021

	Acute medical			Surgical			Long-term			Total			COVID-19		
**People needing oxygen (millions)**															
	#	LB	UB	#	LB	UB	#	LB	UB	**#**	**LB**	**UB**	#	LB	UB
**GLOBAL**	105.4	39.6	211.0	259.0	230.0	288.4	9.2	5.9	13.5	373.6	275.5	512.9	52.4	25.1	89.8
**HICs**	18.3	6.1	37.7	46.8	42.4	51.1	2.9	1.9	4.2	68.1	50.4	93.1	8.7	6.3	11.8
**LMICs**	87.1	33.5	173.3	212.2	187.6	237.3	6.2	4.0	9.3	305.5	225.1	419.8	43.7	18.8	78.1
East Asia & Pacific	21.0	7.3	43.0	63.9	58.6	69.2	2.8	1.8	4.2	87.8	67.7	116.4	4.5	3.0	6.4
Europe & Central Asia	2.7	0.9	5.4	9.1	7.8	10.4	0.3	0.2	0.5	12.1	8.9	16.2	3.0	1.4	5.3
Latin America & Caribbean	6.8	2.3	14.1	16.5	14.5	18.5	0.6	0.4	0.9	23.9	17.2	33.5	5.9	3.5	9.0
Middle East & North Africa	3.9	1.4	7.8	12.6	10.9	14.2	0.3	0.2	0.4	16.8	12.5	22.5	3.6	1.6	6.3
North America	-	-	-	-	-	-	-	-	-	-	-	-	-	-	-
South Asia	32.1	12.5	63.3	59.1	51.8	66.7	1.8	1.2	2.7	93.0	65.5	132.8	15.4	6.3	28.1
Sub-Saharan Africa	20.6	9.0	39.6	51.1	44.0	58.3	0.4	0.2	0.6	72.0	53.2	98.4	11.3	3.1	22.9
**Minimum oxygen volume to meet need (million cubic metres/Nm³)**															
	#	LB	UB	#	LB	UB	#	LB	UB	**#**	**LB**	**UB**	#	LB	UB
**GLOBAL**	1,132.0	418.6	2,264.2	114.8	101.9	127.8	3,178.8	2,044.2	4,683.8	4,425.5	2,564.7	7,075.7	1,913.2	910.8	3,284.8
**HICs**	200.1	66.3	411.6	20.7	18.8	22.7	1,015.1	664.7	1,472.7	1,236.0	749.8	1,907.0	319.5	230.7	433.2
**LMICs**	931.9	352.3	1,852.5	94.0	83.1	105.1	2,163.6	1,379.5	3,211.1	3,189.6	1,814.9	5,168.7	1,593.7	680.1	2,851.7

East Asia & Pacific	238.8	81.9	488.1	28.3	26.0	30.7	978.6	620.8	1,458.5	1,245.7	728.7	1,977.2	162.5	107.4	233.6
Europe & Central Asia	28.2	9.8	57.1	4.0	3.4	4.6	108.3	69.0	161.4	140.5	82.3	223.1	111.2	52.1	195.0
Latin America & Caribbean	71.7	24.2	147.9	7.3	6.4	8.2	215.6	136.3	322.8	294.6	166.9	478.8	216.8	129.6	329.1
Middle East & North Africa	38.4	13.7	77.3	5.6	4.8	6.3	95.9	59.9	145.1	139.8	78.4	228.7	132.8	57.1	231.8
North America	-	-	-	-	-	-	-	-	-	-	-	-	-	-	-
South Asia	371.8	136.6	745.8	26.2	23.0	29.6	639.7	415.3	932.4	1,037.7	574.9	1,707.8	562.3	224.9	1,028.9
Sub-Saharan Africa	183.0	86.1	336.4	22.6	19.5	25.8	125.6	78.2	190.9	331.2	183.7	553.1	408.1	108.9	833.3

LB, lower bound; UB, upper bound. Minimum volume of oxygen required to meet need is calculated using data on recommended and usual flow rates and duration for various conditions and assumes no inefficiencies in oxygen use and no wastage or inefficiencies in upstream oxygen production, supply, and distribution. *Data for World Bank regions presents data for LMICs within those regions only.

Table 26 Oxygen need and volume by World Bank region in High-income Countries (HIC) and Low- and Middle-Income Countries (LMIC)

Region	Acute Oxygen Needs (in million population): Acute medical and surgical (excluding COVID-19)						Acute Oxygen Volume (in Nm^3): Acute medical and surgical (excluding COVID-19)					
	HIC Oxygen Need			LMIC Oxygen Need			HIC Oxygen Volume			LMIC Oxygen Volume		
	#	LB	UB	#	LB	UB	#	LB	UB	#	LB	
East Asia & Pacific	10.1	7.6	13.7	84.9	65.9	112.2	34.7	13.2	68.8	267.1	107.9	518.7
Europe & Central Asia	33.6	24.9	45.8	11.7	8.7	15.7	109.2	42.3	214.4	32.2	13.3	61.7
Latin America & Caribbean	1.3	0.9	1.8	23.3	16.8	32.6	4.6	1.7	9.0	79.0	30.7	156.0
Middle East & North Africa	2.9	2.1	3.9	16.5	12.3	22.1	8.1	3.2	15.7	43.9	18.5	83.6
North America	17.3	12.9	23.7	-	-	-	64.2	24.5	126.3	-	-	-
South Asia	-	-	-	91.2	64.3	130.1	-	-	-	398.0	159.5	775.4
Sub-Saharan Africa	0.006	0.004	0.008	71.6	53.0	97.9	0.015	0.007	0.033	205.7	105.6	362.2

Table 27 Trend in estimated people needing oxygen (millions) from 2010 to 2021 using the Global Burden of Disease data for: acute medical (excluding COVID-19), perioperative, and long-term oxygen indications

	Acute medical (excluding COVID-19)			Perioperative			Long-term oxygen therapy		
**Year**	Estimate	Lower bound	Upper bound	Estimate	Lower bound	Upper bound	Estimate	Lower bound	Upper bound
**2010**	104.7	43.3	200.1	227	222.4	231.3	6.9	4.5	10.0
**2013**	104.8	42.8	200.5	235	230.2	241.0	7.5	4.9	10.9
**2016**	105.9	42.6	203.1	244	238.0	250.6	8.1	5.3	11.8
**2019**	107.5	42.7	207.1	253	246.1	259.7	8.8	5.7	12.7
**2021**	105.4	39.6	211.0	259	230.0	288.4	9.2	6.0	13.4

4.2 Detailed oxygen and pulse oximetry coverage data

The following section contains additional analysis on pulse oximeter and oxygen availability and use, including data that informed our oxygen coverage estimates.

Table 28 Pulse oximeter availability in LMIC health facilities, meta-estimates (%, 95% CI)

Pulse oximeter availability generally reported without consideration of functionality, adequacy, or appropriateness to use-case, thereby over-estimating actual oximeter availability. See main report for additional discussion of facility oxygen readiness.

POX availability	All Acute	OPD / triage	Paediatric	Neonatal	Adult ward	Maternity	Emergency	Operating theatre	Intensive care
Overall	19% (18-19)<br>N=158 (101*)	15% (15-16)<br>N=53 (18)	40% (36-45)<br>N=30 (14)	75% (70-80)<br>N=18 (7)	59% (54-63)<br>N=16 (6)	70% (66-74)<br>N=5 (4)	80% (76-84)<br>N=28 (10)	89% (87-92)<br>N=46 (26)	97% (95-99)<br>N=25 (10)
Primary	10% (10-11)<br>N=33 (31)	10% (10-11)<br>N=33 (31)	NA	NA	NA	NA	NA	NA	NA
Secondary	54% (53-56)<br>N=91 (79)	42% (40-44)<br>N=22 (18)	35% (31-39)<br>N=22 (14)	69% (63-75)<br>N=12 (7)	50% (45-55)<br>N=8 (4)	69% (65-73)<br>N=3	75% (71-79)<br>N=15 (10)	86% (83-89)<br>N=29 (22)	85% (80-90)<br>N=13 (6)
Tertiary	83% (79-86)<br>N=36 (30)	53% (44-63)<br>N=14 (10)	91% (80-99)<br>N=8 (3)	76% (67-85)<br>N=7 (3)	92% (84-98)<br>N=8 (4)	75% (71-79)<br>N=3	96% (88-100)<br>N=13 (8)	99% (94-100)<br>N=17 (15)	99% (97-100)<br>N= 16 (9)
**Sub-Saharan Africa**									
Overall	17% (16-17)<br>N=109 (71)	14% (13-15)<br>N=33 (11)	32% (27-36)<br>N=19 (9)	65% (57-72)<br>N=13 (5)	60% (54-66)<br>N=9 (3)	100% (72-100)<br>N=1	83% (78-88)<br>N=19 (10)	86% (82-89)<br>N=27 (15)	99% (97-100)<br>N=14 (7)
Primary	7% (6-8)<br>N=21 (20)	7% (6-8)<br>N=21 (20)							
Secondary	46% (44-48)<br>N=66 (57)	33% (31-35)<br>N=14 (11)	28% (23-32)<br>N=15 (9)	61% (54-69)<br>N=9 (5)	46% (39-52)<br>N=4 (2)	no data	76% (71-81)<br>N=10 (7)	82% (79-86)<br>N=19 (13)	96% (90-100)<br>N=6 (4)
Tertiary	80% (74-86)<br>N=23 (19)	46% (34-58)<br>N=8 (5)	90% (75-99)<br>N=4 (1)	50% (33-68)<br>N=5 (2)	96% (88-100)<br>N=5 (3)	100% (72-100)<br>N=1	100% (94-100)<br>N=9 (6)	100% (94-100)<br>N=8 (6)	100% (97-100)<br>N=9 (7)
**South Asia**									
Overall	23% (22-25)<br>N=21 (15)	21% (20-22)<br>N=12 (5)	67% (54-80)<br>N=2 (1)	91% (85-96)<br>N=3 (2)	55% (45-64)<br>N=2 (1)	49% (39-59)<br>N=2	69% (60-78)<br>N=2 (1)	92% (86-97)<br>N=6 (4)	93% (88-96)<br>N=4 (3)
Primary	16% (15-17)<br>5 studies	16% (15-17)<br>5 studies							

POX availability	All Acute	OPD / triage	Paediatric	Neonatal	Adult ward	Maternity	Emergency	Operating theatre	Intensive care
Secondary	73% (70-76) N=10	71% (67-74) N=5	63% (49-75) N=1	98% (90-100) N=2	53% (43-62) N=1	48% (38-58) N=1	68% (59-76) N=1	91% (84-96) N=2	71% (62-80) N=2
Tertiary	96% (91-99) N=6	66% (50-80) N=3	88% (53-98) N=1	82% (72-89) N=1	78% (45-94) N=1	62% (31-86) N=1	71% (36-92) N=1	95% (82-100) N=4	98% (94-100) N=3
**East Asia / Pacific**									
Overall	70% (67-73) N=12 (8)	66% (37-92) N=2 (1)	70% (59-80) N=9 (5)	10% (0-36) N=2 (1)	59% (49-68) N=5 (2)	56% (27-81) N=1	83% (73-92) N=5 (2)	85% (55-100) N=4 (3)	79% (65-91) N=7(3)
Primary	63% (55-71) N=1	63% (55-71) N=1							
Secondary	70% (67-73) N=8 (7)	60% (39-78) N=1	64% (53-75) N=6 (5)	0% (0-26) N=1	56% (46-65) N=3 (2)	56% (27-81) N=1	81% (71-90) N=3 (2)	77% (41-100) N=3	77% (62-90) N=5 (3)
Tertiary	81% (59-97) N=3 (2)	100% (21-100) N=1	94% (70-100) N=3 (2)	100% (44-100) N=1	72% (46-94) N=2 (1)	no data	86% (59-100) N=2 (1)	100% (44-100) N=1	90% (75-99) N=4 (2)
**Latin America / Caribbean**									
Overall	9% (8-11) N=15 (10)	10% (8-11) N= 6 (2)	no data	no data	no data	no data	63% (32-90) N=2 (1)	99% (94-100) N=9 (5)	no data
Primary	9% (7-10) N=5 (4)	9% (7-10) N=5 (4)							
Secondary	84% (75-91) N=7	39% (32-45) N=2					50% (19-81) N=1	99% (92-100) N=5	
Tertiary	96% (73-100) N=3	50% (23-77) N= 2					75% (36-94) N=1	99% (86-100) N=4	
**Middle East / North Africa**									
Overall	no data	no data	no data	no data	no data	no data	no data	no data	98% (91-100) N=1
Primary									
Secondary									
Tertiary									98% (91-100) N=1
**Eastern European / Central Asia**									
Overall	no data	no data	no data	no data	no data	no data	no data	no data	no data

POX availability	All Acute	OPD / triage	Paediatric	Neonatal	Adult ward	Maternity	Emergency	Operating theatre	Intensive care
Primary									
Secondary									
Tertiary									

*N=# is number of datasets in meta-estimate; (#) is number of unique studies contributing to meta-estimate; OPD, outpatient department. "All acute" includes all general ward areas and excludes operating theatre and intensive care units. Facility level: Level 1 (primary care clinic); Level 2 (general/district hospital); Level 3 (tertiary/teaching hospital).

Table 29 Oxygen availability in LMIC health facilities, meta-estimates (%, 95% CI)

Oxygen availability generally reported without consideration of functionality, adequacy, or appropriateness to use-case, thereby over-estimating actual oxygen availability. See main report for additional discussion of facility oxygen readiness.

O2 availability	All Acute	OPD / triage	Paediatric	Neonatal	Adult ward	Maternity	Emergency	Operating theatre	Intensive care
Overall	24% (23-24) N=280 (171*)	17% (16-17) N=58 (21)	43% (41-46) N=45 (22)	77% (73-81) N=18 (7)	73% (69-77) N=18 (6)	36% (34-38) N=28 (16)	88% (85-90) N=33 (13)	93% (90-95) N=41 (23)	97% (94-98) N=25 (11)
Primary	12% (11-12) N=67 (65)	12% (11-12) N=67 (65)	NA	NA	NA	21% (19-23) N=11	NA	NA	NA
Secondary	58% (57-59) N=153 (135)	38% (37-40) N=25 (21)	44% (42-47) N=29 (20)	70% (66-75) N=12 (7)	69% (64-73) N=9 (5)	53% (50-56) N=13	84% (81-87) N=18 (13)	91% (88-94) N=27 (20)	93% (88-96) N=10 (6)
Tertiary	86% (83-88) N=50 (43)	66% (56-75) N=14 (10)	94% (85-99) N=11 (6)	65% (59-71) N=8 (4)	88% (80-94) N=9 (5)	95% (91-89) N=5	96% (89-100) N=15 (10)	94% (86-99) N=14 (12)	99% (96-100) N=15 (11)
**Sub-Saharan Africa**									
Overall	20% (19-20) N=201 (129)	12% (12-13) N=38 (14)	59% (56-63) N=28 (17)	70% (65-75) 13 (5) studies	64% (59-69) N=12 (5)	25% (23-27) N=19 (11)	82% (78-86) N=23 (10)	89% (85-92) N=28 (16)	97% (95-99) N=18 (9)
Primary	8% (8-9) n=46 (45)	8% (8-9) n=46 (45)				16% (14-18) N=8			
Secondary	52% (51-53) N=115 (104)	26% (24-27) N=17 (14)	63% (59-67) N=19 (15)	67% (61-72) N=9 (5)	59% (53-66) N= 6 (4)	39% (35-42) N=8	78% (73-82) N=13 (10)	87% (84-91) N=20 (14)	92% (87-96) N=7 (5)
Tertiary	68% (63-72) N=32 (29)	63% (51-75) N=8 (5)	87% (76-96) N=6 (4)	51% (44-59) N=6 (3)	80% (70-88) N=6 (4)	72% (60-83) N=3	95% (87-100) N=10 (7)	84% (68-96) N=8 (6)	100% (98-100) N=11 (9)
**South Asia**									
Overall	36% (34-37) N=38 (26)	28% (26-29) N=12 (5)	100% (98-100) N=2 (1)	93% (87-97) N=3 (2)	99% (95-100) N=2 (1)	87% (79-93) N=4 (2)	96% (91-99) N=2 (1)	99% (95-100) N=5 (3)	93% (88-97) N=3 (2)
Primary	23% (21-24) N=12	23% (21-24) N=12				43% (24-63) N=1			
Secondary	81% (79-83) N=18	74% (71-77) N=5	100% (92-100) N=1	93% (84-99) N=2	97% (92-99) N=1	94% (86-99) N=2	93% (88-97) N=1	98% (94-100) N=2	95% (86-98) N=1
Tertiary	100% (98-100) N=7	71% (56-84) N=3	100% (68-100) N=1	91% (83-96) N=1	100% (70-100) N=1	100% (68-100) N=1	100% (65-100) N=1	98% (84-100) N=3	92% (85-97) N=2

O2 availability	All Acute	OPD / triage	Paediatric	Neonatal	Adult ward	Maternity	Emergency	Operating theatre	Intensive care
**East Asia / Pacific**									
Overall	67% (63-70) N=17 (10)	95% (72-100) N=2 (1)	85% (75-92) N=9 (5)	45% (17-74) N=2 (1)	65% (55-74) N=4 (1)	no data	100% (100-100) N=4 (1)	100% (70-100) N=1	86% (65-99) N=4 (1)
Primary	59% (52-66) N=2	59% (52-66) N=2							
Secondary	66% (62-69) N=10 (9)	85% (64-95) N=1	78% (68-87) N=6 (5)	27% (10-57) N=1	54% (44-63) N=2 (1)		100% (98-100) N=2 (1)	100% (70-100) N=1	74% (49-94) N=2 (1)
Tertiary	100% (93-100) N=5 (2)	100% (21-100) N=1	100% (86-100) N=3 (2)	100 N=1	100% (95-100) N=2 (1)		100% (93-100) N=2 (1)	no data	100% (74-100) N=2 (1)
**Latin America / Caribbean**									
Overall	17% (16-19) N=17 (11)	12% (10-13) N=6 (2)	no data	no data	no data	99% (96-100) N=1	46% (17-76) N=2 (1)	100% (94-100) N=7 (4)	no data
Primary	10% (8-11) N=6 (5)	10% (8-11) N=6 (5)				no data			
Secondary	100% (99-100) N=8	55% (48-61) N=2				99% (96-100) N=1	42% (14-76) N=1	99% (91-100) N=4	
Tertiary	100% (100-100) N=4	50% (23-77) N=2				99% (96-100) N=1	50% (19-81) N=1	100% (91-100) N=3	
**Middle East / North Africa**									
Overall	27% (25-30) N=4 (2)	no data	27% (25-30) N=4 (2)	no data	no data	57% (52-62) N=4 (2)	no data	no data	100% (94-100) N=1
Primary	no data					77% (69-84) N=2			
Secondary	26% (23-29) N=2		26% (23-29) N=2			47% (41-53) N=2			no data
Tertiary	no data		no data			no data			100% (94-100) N=1
**Eastern European / Central Asia**									
Overall	19% (2-44) N=2 (1)	no data	19% (2-44) N=2 (1)	no data	no data	no data	no data	no data	no data
Primary	no data								

O2 availability	All Acute	OPD / triage	Paediatric	Neonatal	Adult ward	Maternity	Emergency	Operating theatre	Intensive care
Secondary	16% (6-38)<br>N=1		16% (6-38)<br>N=1						
Tertiary	100% (34-100)<br>N=1		100% (34-100)<br>N=1						

*N=# is number of datasets in meta-estimate; (#) is number of unique studies contributing to meta-estimate; OPD, outpatient department. “All acute” includes all general ward areas and excludes operating theatre and intensive care units. Facility level: Level 1 (primary care clinic); Level 2 (general/district hospital); Level 3 (tertiary/teaching hospital).

Table 30 Pulse oximetry coverage to patients in LMIC health facilities, meta-estimates (%, 95% CI)

Pulse oximetry coverage generally reported as whether patients had a documented pulse oximetry reading, irrespective of timeliness or appropriateness of ongoing oximetry monitoring, thereby over-estimating actual pulse oximetry coverage. See main report for additional discussion of oxygen quality of care.

POX coverage	All Acute	OPD / triage	Paediatric	Neonatal	Adult ward	Maternity	Emergency	Operating theatre	Intensive care
Overall	28% (28-29) N=93 (33)	0% (0-0) N=13 (5)	13% (12-13) N=25 (10)	6% (5-6) N=13 (6)	43% (43-44) N=23 (12)	27% (27-28) N=2	70% (69-71) N=19 (7)	91% (90-91) N=6 (5)	78% (72-83) N=11 (1)
Primary	0% (0-0) N=8 (5)	0% (0-0) N=8 (5)	NA	NA	NA	0% (0-5) N=1	NA	NA	NA
Secondary	19% (19-19) N=52 (23)	0% (0-1) N=4 (1)	12% (12-13) N=16 (9)	6% (5-6) N=9 (6)	29% (28-29) N=15	no data	72% (70-73) N=8 (3)	85% (83-86) N=3	79% (66-90) N=5 (1)
Tertiary	54% (53-54) N=37 (14)	97% (85-99) N=1	30% (28-32) N=9 (4)	71% (64-79) N=4 (1)	70% (69-71) N=11 (6)	28% (27-29) N=1	69% (68-70) N=11 (6)	92% (92-83) N=3	77% (70-83) N=6 (1)
**Sub-Saharan Africa**									
Overall	25% (25-25) N=63 (19)	0% (0-0) N=10 (4)	12% (11-12) N=19 (9)	7% (6-7) N=9 (3)	54% (54-55) N=12 (5)	28% (27-29) N=1	59% (58-60) N=13 (5)	67% (64-71) N=2	66% (55-75) N=8(1)
Primary	0% (0-0) N=6 (4)	0% (0-0) N=6 (4)				no data			
Secondary	14% (13-14) N=33 (15)	0% (0-0) N=3 (1)	12% (12-12) N=12 (8)	6% (5-6) N=6 (3)	8% (7-9) N=6 (3)		70% (68-71) N=6 (3)	54% (47-60) N=1	63% (47-79) N=4 (1)
Tertiary	47% (47-48) N=25 (9)	97% (85-99) N=1	21% (19-23) N=7 (4)	70% (63-77) N=3 (1)	71% (70-72) N=6 (3)	28% (27-29) N=1	50% (49-51) N=7 (4)	73% (69-77) N=1	67% (54-79) N=4 (1)
**South Asia**									
Overall	22% (21-23) N=6	0% (0-1) N=1	76% (65-84) N=1	5% (3-6) N=3	24% (23-25) N=1	no data	no data	no data	no data
Primary	0% (0-1) N=1	0% (0-1) N=1							
Secondary	23% (22-23) N=4			5% (3-6) N=3	24% (23-25) N=1				
Tertiary	76% (65-84) N=1		76% (65-84) N=1	no data	no data				

POX coverage	All Acute	OPD / triage	Paediatric	Neonatal	Adult ward	Maternity	Emergency	Operating theatre	Intensive care
**East Asia / Pacific**									
Overall	46% (44-47) N=16 (2)	51% (37-65) N=1	23% (21-24) N=6 (2)	67% (21-94) N=1	79% (77-81) N=4 (1)	no data	85% (82-88) N=4 (1) studies	93% (92-94) N=2 (1)	83% (76-89) N=3 (1)
Primary	no data								
Secondary	34% (32-35) N=9 (3)	51% (37-65) N=1	15% (14-17) N=4 (2)		79% (76-82) N=2 (1)		83% (79-87) N=2 (1)	96% (90-98) N=1	100% (82-100) N=1
Tertiary	84% (82-86) N=7 (1)		84% (80-87) N=2 (1)	67% (21-94) N=1	79% (76-83) N=2 (1)		88% (84-91) N=2 (1)	93% (92-93) N=1	79% (72-86) N=2
**Latin America / Caribbean**									
Overall	86% (85-87) N=4	no data	no data	no data	55% (52-58) N=2	no data	93% (92-94) N=2	no data	no data
Primary	no data								
Secondary	55% (52-58) N=2				55% (52-58) N=2		no data		
Tertiary	85% (84-86) N=3				15% (12-19) N=1		93% (92-94) N=2		
**Middle East / North Africa**									
Overall	62% (57-66) N=3	0% (0-5) N=1	no data	no data	76% (72-80) N=2	0% (0-5) N=1	no data	99% (95-100) N=1	no data
Primary	0% (0-5) N=1	0% (0-5) N=1				0% (0-5) N=1			
Secondary	76% (72-80) N=2				76% (72-80) N=2			no data	
Tertiary	81% (76-86) N=1				81% (76-86) N=2	no data		99% (95-100) N=1	
**Eastern European / Central Asia**									
Overall	95% (91-97) N=1	no data	no data	no data	95% (91-97) N=1	no data	no data	no data	no data
Primary	no data								
Secondary					95% (91-97) N=1				

POX coverage	All Acute	OPD / triage	Paediatric	Neonatal	Adult ward	Maternity	Emergency	Operating theatre	Intensive care
	95% (91-97%)<br>N=1								
Tertiary	no data				no data				

*N=# is number of datasets in meta-estimate; (#) is number of unique studies contributing to meta-estimate; OPD, outpatient department. “All acute” includes all general ward areas and excludes operating theatre and intensive care units. Facility level: Level 1 (primary care clinic); Level 2 (general/district hospital); Level 3 (tertiary/teaching hospital).

Table 31 Oxygen coverage to patients with hypoxaemia in LMIC health facilities, meta-estimates (%, 95% CI)

Oxygen coverage generally reported as whether patients with SpO_2<90% received oxygen therapy, with resultant under-representation of facilities with low pulse oximetry coverage and therefore over-estimating actual oxygen coverage. This is also reported irrespective of timeliness, appropriateness, interruptions, or other aspects of oxygen care quality, additionally over-estimating actual oxygen coverage. See main report for additional discussion of oxygen quality of care.

O2 coverage	All Acute	OPD / triage	Paediatric	Neonatal	Adult ward	Maternity	Emergency	Operating theatre	Intensive care
Overall	58% (57-59) N=102 (23)	17% (4-34) N=4 (1)	32% (31-33) N=23 (10)	84% (82-85) N=13 (7)	75% (73-77) N=42 (8)	1% (0-16) N=9	77% (73-80) N=11 (3)	no data	99% (93-100) N=21 (2)
Primary	50% (28-72) N=1	50% (27-72) N=1	NA	NA	NA	no data	NA	NA	NA
Secondary	45% (44-46) N=45 (12)	0% (0-6) N=2 (1)	30% (28-31) N=13 (7)	85% (83-87) N=8 (6)	79% (73-84) N=15 (3)	0% (0-17) N=2 (1)	89% (85-93) N=5 (1)		90% (61-100) N=5 (2)
Tertiary	79% (77-80) N=57 (15)	67% (21-94) N=1	88% (84-92) N=11 (6)	83% (81-85) N=5 (2)	74% (72-76) N=27 (7)	6% (0-32) N=7 (1)	64% (59-70) N=6 (3)		99% (93-100) N=16 (2)
**Sub-Saharan Africa**									
Overall	57% (57-58) N=83 (20)	17% (4-34) N=4 (1)	32% (31-33) N=19 (10)	80% (79-81) N=10 (5)	77% (75-79) N=34 (8)	1% (0-18) N=8 (1)	73% (68-77) N=8 (2)		98% (87-100) N=13 (2)
Primary	50% (28-72) N=1	50% (27-72) N=1	NA	NA	NA	no data			
Secondary	45% (44-46) N=37 (10)	0% (0-6) N=2 (1)	31% (29-32) N=11 (7)	81% (80-83) N=6 (4)	81% (75-86) N=12 (3)	0% (0-17) N=2 (1)	88% (83-92) N=4 (1)		93% (62-100) N=3 (2)
Tertiary	79% (78-80) N=46 (14)	67% (21-94) N=1	88% (84-92) N=9 (6)	79% (77-81) N=4 (2)	76% (73-78) N=22 (7)	9% (0-41) N=6 (1)	57% (51-63) N=4 (2)		99% (89-100) N=10 (2)
**South Asia**									
Overall	97% (89-100) N=4	no data	93% (85-98) N=2	15% (0-78) N=2	no data	no data	no data		no data
Primary	no data								
Secondary	15% (0-78) N=2		no data	15% (0-78) N=2					
Tertiary	93% (85-98) N=2		93% (85-98) N=2						

O2 coverage	All Acute	OPD / triage	Paediatric	Neonatal	Adult ward	Maternity	Emergency	Operating theatre	Intensive care
**East Asia / Pacific**									
Overall	90% (82-97) N=11 (1)	no data	66% (36-91) N=4 (1)	100% (21-100) N=1	74% (57-88) N=4 (1)	no data	93% (86-98) N=2 (1)		100% (89-100) N=1
Primary	no data								
Secondary	91% (79-99) N=5 (1)		66% (15-100) N=2 (1)	no data	55% (23-85) N=2 (1)		95% (82-99) N=1		100% (34-100) N=1
Tertiary	90% (78-98) N=6 (1)		65% (30-94) N=2 (1)	100% (21-100) N=1	81% (62-95) N=2 (1)		91% (77-97) N=1		98% (86-100) N=2 (1)
**Latin America / Caribbean**									
Overall	93% (86-98) N=2	no data	93% (84-97) N=1	no data	no data	no data	90% (70-97) N=1		no data
Primary	no data								
Secondary	no data								
Tertiary	93% (86-98) N=2		93% (84-97) N=1				90% (70-97) N=1		
**Middle East / North Africa**									
Overall	30% (19-40) N=5 (1)	no data	no data	no data	32% (21-44) N=4 (1)	0% (0-56) N=1	no data		5% (76-100 N= 5 (1)
Primary	no data								
Secondary	57% (25-84) N=1				57% (25-84) N=1	no data			0% (0-79) N=1
Tertiary	27% (16-40) N=4 (1)				30% (18-42) N=3 (1)	0% (0-56) N=1			97% (79-100) N=4 (1)
**Eastern European / Central Asia**									
Overall	no data	no data	no data	no data	no data	no data	no data		no data
Primary									
Secondary									
Tertiary									

*N=# is number of datasets in meta-estimate; (#) is number of unique studies contributing to meta-estimate; OPD, outpatient department. "All acute" includes all general ward areas and excludes operating theatre and intensive care units. Facility level: Level 1 (primary care clinic); Level 2 (general/district hospital); Level 3 (tertiary/teaching hospital).

Figure 10 Meta-estimates of pulse oximeter and oxygen availability in health facilities and coverage for acutely unwell patients, by decade from 2000 to 2024

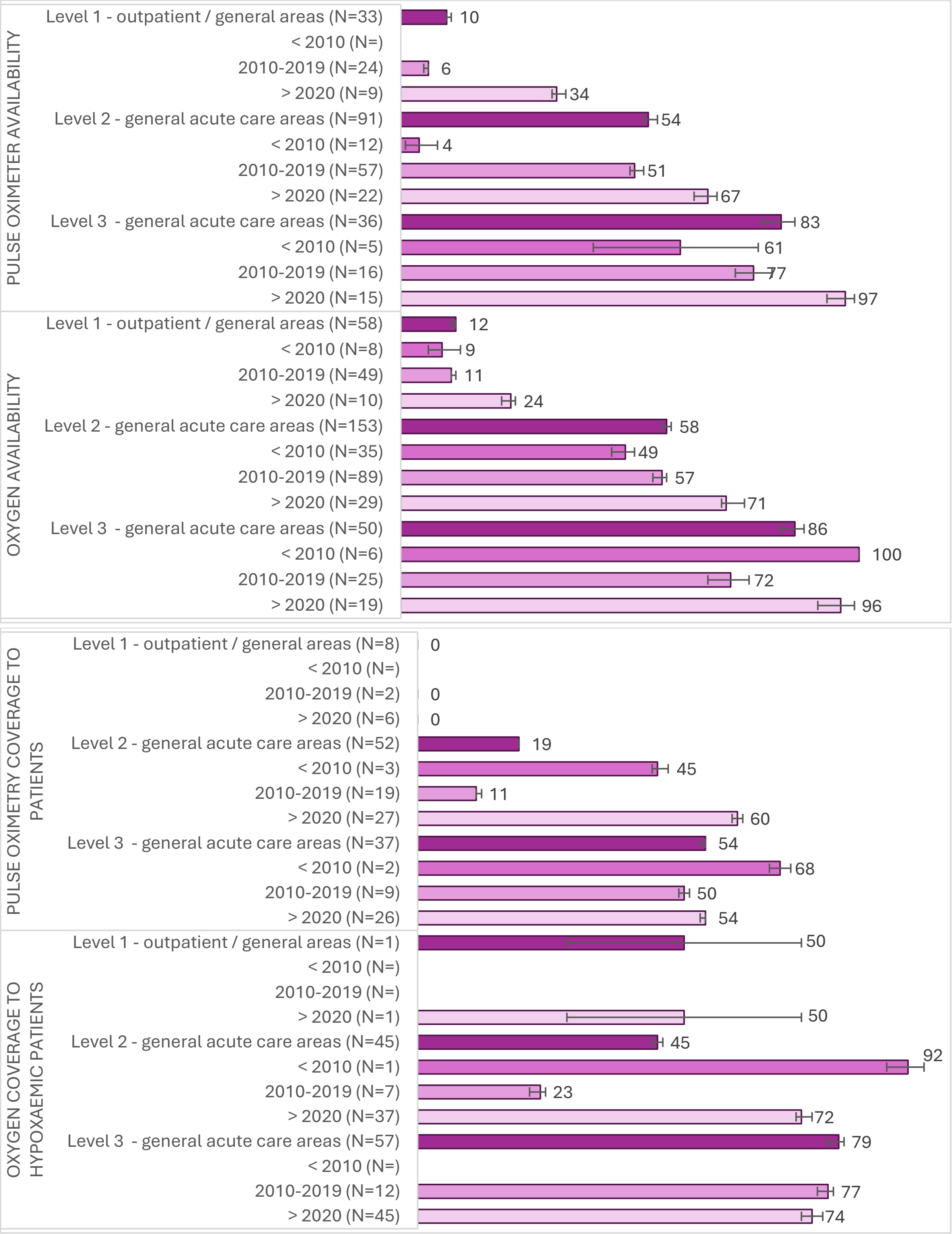

*Post-2020 data mostly comes from countries with active oxygen systems strengthening programs currently underway. Pulse oximetry coverage refers to the proportion of acutely unwell patients who had a pulse oximeter reading documented (variously reported at presentation, admission, any time). Oxygen coverage refers to the proportion of acutely unwell patients with hypoxaemia (variously reported based on SpO_2 or clinical signs) who received oxygen (in any modality). General acute care areas analysis excludes operating theatres and intensive-care units.

4.3 Challenges and solutions coding tree

At the start of the Commission process, the Cascade of Care (Figure 1) was mapped to 6 'wicked challenges' and a further wicked challenge emerged during the work. Solutions emerged from multiple sources – literature, consultations and case studies. These were synthesised into 4 global themes, with sub-themes, that address these 7 wicked challenges.

Table 32 Coding tree for challenges and corresponding solutions

Wicked challenge	Solution theme	Solution sub-themes	Evidence source
Weak hypoxaemia detection and referral pathways limit access	Linking patients to care through pulse oximetry at every health facility	Make oxygen an essential part of Universal Health Coverage	Barriers systematic review
		Work with communities to change misconceptions and fears of accessing oxygen services	Patient perspectives scoping review
		Include primary care services in oxygen system planning	TLO model
		Make pulse oximeters better	Consultations<br>Skin pigmentation review
Low HCW capacity, opportunity, and motivation to provide oxygen therapy		Empower healthcare workers to use pulse oximeters and oxygen	Barriers systematic review
		Integrate oxygen into all relevant care packages	Care package targeted review
		Sustained support to make SpO2 a vital sign for quality oxygen care	Barriers systematic review
Unreliable and costly medical oxygen supplies	Building resilient medical oxygen production, distribution and delivery systems	Embrace mixed-source oxygen supply systems	Barriers systematic review
		Foster locally appropriate oxygen distribution and maintenance models	Case studies<br>Barriers systematic review
		Make energy for health reliable and affordable (and cleaner)	Case studies<br>Cost rapid review
		Establish infrastructure for surge capacity and rapid scale-up	
		Collect and use oxygen data for timely decision making	
Weak biomedical systems for oxygen-related devices		Strengthen biomedical service capacity, including workforce, essential tools, and processes	Barriers systematic review
		Include oxygen-related devices and considerations in health tech management plans.	Barriers systematic review
Lack of strategic planning and action on medical oxygen systems	Coordinating the management of medical oxygen systems	Develop costed National Oxygen Plans	Case studies
		Integrate oxygen into pandemic preparedness and emergency response	Consultations
		Establish Oxygen desk at national and sub-national level	Nigeria case study
		Adopt ATMO$_2$S and core indicators	
Weak broader health systems infrastructure, workforce, and systems		Evidence champions and local data, innovation, learning	Case studies
		Promote funding for holistic oxygen systems improvement	Case studies<br>Consultations

Weak governance in the face of a strong industry fosters sub-optimal market conditions	Strengthening medical oxygen regulations and markets	Adopt a universal definition of medical oxygen to prevent 'policy traps'	Peru case study
		Increase price transparency for oxygen supply and services	Case studies
		Partner with industry to improve oxygen access	Consultations
		Improve oxygen-related standards and regulation to better service LMICs	Consultations<br>Barriers systematic review

5. Consultations

To ensure that the *Lancet Global Health* Commission on Medical Oxygen Security was informed by stakeholders who were not represented among Commissioners and Advisors, the Executive Committee undertook targeted consultations with patients, caregivers, and clinicians, Ministries of Health, industry, and select experts, and conducted monthly webinars that were open to all "oxygen access collaborators."

Patients, caregivers, and clinicians

Video recordings and written testimony was collected from nine patients, eight family carers, and eight clinicians from Africa, Asia, and Latin America. Each testimony was securely stored in a cloud folder accessible to Commissioners and the Executive Committee, together with signed Consent Forms from each speaker. Over 20 hours of testimony was reviewed with quotable comments collected into a separate document for use in the Commission report. Video testimonies were shared with the *Lancet Global Health* for compilation in a short video to be released with the final report. These stories aimed to give voice to those affected by lack of access to oxygen, to ensure the Commission was grounded in real-life experiences.

Ministries of Health

To solicit the views of governments on the Access to Medical Oxygen Scorecard (ATMO_2S) as a tool to measure national progress to the WHO Increasing Access to Medical Oxygen Resolution, the Commission invited 40 Ministries of Health representing all WHO regions to test the tool. Due to limited engagement with this outreach, the Commission hosted a booth at the WHO National Oxygen Scale-Up Framework Meeting in Senegal from 14 to 16 May 2024 with the express purpose of engaging more Ministries of Health in the Scorecard. Representatives from 62 member states attended, and 14 signed up for an additional ATMO_2S deep dive.

Industry

A webinar was hosted on 30 November 2023 for 54 of 300 invited representatives of the medical oxygen industry, including liquid oxygen, PSA/VSA plant, mobile concentrator, pulse oximeter, and respiratory therapy manufacturers. Invitees were also given the opportunity to submit short written testimony to the Commission by January 2024 and 11 submissions were received. In addition, industry members who wanted to participate more actively in the work of the Commission were encouraged to join the monthly "oxygen access collaborator" calls.

Experts

Twelve virtual meetings were held with experts who were invited to speak to the Commission on specific topics of concern or up-coming research findings, including (in alphabetical order), Disease Control Priorities 4 team, International Organization for Standardization (ISO) Technical Committee on Anesthetic and Respiratory Equipment (TC121), International Primary Care Respiratory Group, Lancet Global Health Surgery Commission, Partners in Health, Tools for Integrated Management of Childhood Illness (TIMCI), Improving the Identification of Respiratory Distress in Children (AIRE), and The Integrated Sustainable Childhood Pneumonia and Infectious disease Reduction in Nigeria (INSPIRING) research teams, and the World Health Organization (WHO). Multiple meetings were conducted with the WHO Emergencies, Medical Devices, and Child Health teams in the context of the WHO Increasing Access to Medical Oxygen Resolution,

the National Oxygen Scale-Up Framework Meeting, and the revision to the IMCI Guidelines as they relate to the use of pulse oximetry in the management of childhood pneumonia.

Additional individual experts who briefed the Commission included Sue Horton from the University of Waterloo, Anders Nordstrom, former Global Health Ambassador for Sweden, Robert Neighbor, CEO of Diamedica, and Jeffrey Perlman, Chief of Newborn Medicine, NewYork-Presbyterian / Weill Cornell Medical Center.

Oxygen Access Collaborators

Beginning in December 2022, the Commission invited 1,000 stakeholders to join monthly calls to receive updates on the Commission's progress, to share their feedback, and to raise any other issues they felt pertinent to the Commission's work. Following each call, a newsletter was shared summarizing ways the collaborators could take action to contribute to the Commission's work. Video recordings of each call were distributed and written summaries of the feedback from collaborators was shared with the Commissioners at their quarterly meetings. This group is open for interested individuals to join, and includes representatives from across industry, academia, implementation partners, multi-lateral and funding agencies, and Ministries of Health.

To ensure wide accessibility to the work of the Commission, the Every Breath Counts Coalition maintained a webpage dedicated to its progress and an active social media presence on X and LinkedIn.[138]

6. References

1. Frost LJ, Reich MR. Creating access to health technologies in poor countries. *Health Aff (Millwood)* 2009;28(4):962-73. doi: 10.1377/hlthaff.28.4.962 [published Online First: 2009/07/15]
2. Indicators A. Monitoring the building blocks of health systems. *Geneva, Switzerland: WHO Document Production Services* 2010
3. Marsh AD, Muzigaba M, Diaz T, et al. Effective coverage measurement in maternal, newborn, child, and adolescent health and nutrition: progress, future prospects, and implications for quality health systems. *Lancet Glob Health* 2020;8(5):e730-e36. doi: 10.1016/s2214-109x(20)30104-2
4. Amouzou A, Leslie HH, Ram M, et al. Advances in the measurement of coverage for RMNCH and nutrition: from contact to effective coverage. *BMJ global health* 2019;4(Suppl 4):e001297. doi: 10.1136/bmjgh-2018-001297 [published Online First: 2019/07/13]
5. Shengelia B, Tandon A, Adams OB, et al. Access, utilization, quality, and effective coverage: an integrated conceptual framework and measurement strategy. *Soc Sci Med* 2005;61(1):97-109. doi: 10.1016/j.socscimed.2004.11.055 [published Online First: 2005/04/26]
6. Kruk ME, Gage AD, Arsenault C, et al. High-quality health systems in the Sustainable Development Goals era: time for a revolution. *Lancet Glob Health* 2018;6(11):e1196-e252. doi: 10.1016/s2214-109x(18)30386-3
7. Graham HR, Olojede OE, Bakare AA, et al. Measuring oxygen access: lessons from health facility assessments in Lagos, Nigeria. *BMJ global health* 2021;6(8) doi: 10.1136/bmjgh-2021-006069
8. WHO. Guide to infrastructure and supplies and various levels of health care facilities: Emergency and Essential Surgical Care (EESC). Geneva: World Health Organization, 2012.
9. Graham HR, Jahan E, Subhi R, et al. The prevalence of hypoxaemia among paediatric and adult patients in healthcare facilities in low- and middle-income countries: systematic review and meta-analysis. *Lancet Global Health* 2025;(in press)
10. Munn Z, Moola S, Riitano D, et al. The development of a critical appraisal tool for use in systematic reviews addressing questions of prevalence. *Int J Health Policy Manag* 2014;3(3):123-8. doi: 10.15171/ijhpm.2014.71 [published Online First: 20140813]
11. Cho KH, Kim YS, Nam CM, et al. Home oxygen therapy reduces risk of hospitalisation in patients with chronic obstructive pulmonary disease: a population-based retrospective cohort study, 2005-2012. *BMJ Open* 2015;5(11):e009065. doi: 10.1136/bmjopen-2015-009065 [published Online First: 20151130]
12. Laforest L, Roche N, Devouassoux G, et al. Frequency of comorbidities in chronic obstructive pulmonary disease, and impact on all-cause mortality: A population-based cohort study. *Respir Med* 2016;117:33-9. doi: 10.1016/j.rmed.2016.05.019 [published Online First: 20160520]
13. Müllerova H, Chigbo C, Hagan GW, et al. The natural history of community-acquired pneumonia in COPD patients: a population database analysis. *Respir Med* 2012;106(8):1124-33. doi: 10.1016/j.rmed.2012.04.008 [published Online First: 2012/05/25]
14. Kurmi OP, Davis KJ, Hubert Lam KB, et al. Patterns and management of chronic obstructive pulmonary disease in urban and rural China: a community-based survey of 25 000 adults across 10 regions. *BMJ Open Respir Res* 2018;5(1):e000267. doi: 10.1136/bmjresp-2017-000267 [published Online First: 2018/03/14]
15. Nyaga VN, Arbyn M, Aerts M. Metaprop: a Stata command to perform meta-analysis of binomial data. *Archives of Public Health* 2014;72:1-10.
16. Shi C, Goodall M, Dumville J, et al. The accuracy of pulse oximetry in measuring oxygen saturation by levels of skin pigmentation: a systematic review and meta-analysis. *BMC Med* 2022;20(1):267. doi: 10.1186/s12916-022-02452-8 [published Online First: 20220816]
17. Cabanas AM, Fuentes-Guajardo M, Latorre K, et al. Skin Pigmentation Influence on Pulse Oximetry Accuracy: A Systematic Review and Bibliometric Analysis. *Sensors (Basel)* 2022;22(9) doi: 10.3390/s22093402 [published Online First: 2022/05/21]
18. Rudd KE, Johnson SC, Agesa KM, et al. Global, regional, and national sepsis incidence and mortality, 1990-2017: analysis for the Global Burden of Disease Study. *Lancet* 2020;395(10219):200-11. doi: 10.1016/S0140-6736(19)32989-7
19. Narvaez F, Gutierrez G, Pérez MA, et al. Evaluation of the traditional and revised WHO classifications of Dengue disease severity. *PLoS Negl Trop Dis* 2011;5(11):e1397. doi: 10.1371/journal.pntd.0001397 [published Online First: 2011/11/17]

20. Lamberti LM, Fischer Walker CL, Black RE. Systematic review of diarrhea duration and severity in children and adults in low- and middle-income countries. *BMC Public Health* 2012;12(1):276. doi: 10.1186/1471-2458-12-276
21. McAllister DA, Liu L, Shi T, et al. Global, regional, and national estimates of pneumonia morbidity and mortality in children younger than 5 years between 2000 and 2015: a systematic analysis. *Lancet Glob Health* 2019;7(1):e47-e57. doi: 10.1016/s2214-109x(18)30408-x [published Online First: 2018/12/01]
22. Zeidan Z, Kojal H, Habour A, et al. Clinical and epidemiological features of severe malaria in children in four hospitals in Sudan. *East Mediterr Health J* 2006;12(6):783-91. [published Online First: 2007/03/06]
23. McCollum ED, Ahmed S, Roy AD, et al. Risk and accuracy of outpatient-identified hypoxaemia for death among suspected child pneumonia cases in rural Bangladesh: a multifacility prospective cohort study. *Lancet Respir Med* 2023;11(9):769-81. doi: 10.1016/s2213-2600(23)00098-x [published Online First: 2023/04/11]
24. Hooli S, King C, Zadutsa B, et al. The Epidemiology of Hypoxemic Pneumonia among Young Infants in Malawi. *Am J Trop Med Hyg* 2020;102(3):676-83. doi: 10.4269/ajtmh.19-0516 [published Online First: 2020/01/24]
25. Acar HC, Can G, Karaali R, et al. An easy-to-use nomogram for predicting in-hospital mortality risk in COVID-19: a retrospective cohort study in a university hospital. *BMC Infect Dis* 2021;21(1):148. doi: 10.1186/s12879-021-05845-x [published Online First: 2021/02/07]
26. Padmaprakash KV, Vardhan V, Thareja S, et al. Clinical characteristics and clinical predictors of mortality in hospitalised patients of COVID 19 : An Indian study. *Med J Armed Forces India* 2021;77(Suppl 2):S319-s32. doi: 10.1016/j.mjafi.2021.01.009 [published Online First: 2021/08/03]
27. Krebs E, Gerardo CJ, Park LP, et al. Mortality-Associated Characteristics of Patients with Traumatic Brain Injury at the University Teaching Hospital of Kigali, Rwanda. *World Neurosurg* 2017;102:571-82. doi: 10.1016/j.wneu.2017.03.001 [published Online First: 2017/03/25]
28. King C, Zadutsa B, Banda L, et al. Prospective cohort study of referred Malawian children and their survival by hypoxaemia and hypoglycaemia status. *Bull World Health Organ* 2022;100(5):302-14B. doi: 10.2471/BLT.21.287265 [published Online First: 2022/05/07]
29. Wasingya-Kasereka L, Nabatanzi P, Nakitende I, et al. Oxygen use in low-resource settings: An intervention still triggered by intuition. *Resusc Plus* 2020;4:100056. doi: 10.1016/j.resplu.2020.100056 [published Online First: 2021/07/06]
30. Junge S, Palmer A, Greenwood BM, et al. The spectrum of hypoxaemia in children admitted to hospital in The Gambia, West Africa. *Trop Med Int Health* 2006;11(3):367-72. doi: 10.1111/j.1365-3156.2006.01570.x [published Online First: 2006/03/24]
31. Cunningham S, Rodriguez A, Adams T, et al. Oxygen saturation targets in infants with bronchiolitis (BIDS): a double-blind, randomised, equivalence trial. *Lancet* 2015;386(9998):1041-8. doi: 10.1016/s0140-6736(15)00163-4
32. Meara JG, Leather AJ, Hagander L, et al. Global Surgery 2030: evidence and solutions for achieving health, welfare, and economic development. *Int J Obstet Anesth* 2016;25:75-8. doi: 10.1016/j.ijoa.2015.09.006 [published Online First: 2015/11/26]
33. Rose J, Weiser TG, Hider P, et al. Estimated need for surgery worldwide based on prevalence of diseases: a modelling strategy for the WHO Global Health Estimate. *Lancet Glob Health* 2015;3 Suppl 2(Suppl 2):S13-20. doi: 10.1016/S2214-109X(15)70087-2 [published Online First: 2015/05/01]
34. Ramachandran S, Malhotra N, Velayudhan S, et al. Regional anaesthesia practices in India: A nationwide survey. *Indian J Anaesth* 2021;65(12):853-61. doi: 10.4103/ija.ija_803_21 [published Online First: 2022/03/01]
35. Albutt K, Punchak M, Kayima P, et al. Operative volume and surgical case distribution in Uganda's public sector: a stratified randomized evaluation of nationwide surgical capacity. *BMC Health Serv Res* 2019;19(1):104. doi: 10.1186/s12913-019-3920-9 [published Online First: 2019/02/08]
36. Sury MR, Palmer JH, Cook TM, et al. The state of UK anaesthesia: a survey of National Health Service activity in 2013. *Br J Anaesth* 2014;113(4):575-84. doi: 10.1093/bja/aeu292 [published Online First: 2014/09/23]
37. Yang Q, Xie K, Xiong L. Anaesthesiology in China: present and future. *Br J Anaesth* 2019;123(5):559-64. doi: 10.1016/j.bja.2019.08.004 [published Online First: 2019/09/24]
38. Corvetto M, McCready M, Cook C, et al. [Regional anesthesia practice in Chile: an online survey]. *Rev Esp Anestesiol Reanim* 2010;57(4):209-13. doi: 10.1016/s0034-9356(10)70206-1 [published Online First: 2010/05/27]

39. Górecka D, Gorzelak K, Sliwiński P, et al. Effect of long-term oxygen therapy on survival in patients with chronic obstructive pulmonary disease with moderate hypoxaemia. *Thorax* 1997;52(8):674-9. doi: 10.1136/thx.52.8.674 [published Online First: 1997/08/01]
40. Chaouat A, Weitzenblum E, Kessler R, et al. A randomized trial of nocturnal oxygen therapy in chronic obstructive pulmonary disease patients. *Eur Respir J* 1999;14(5):1002-8. doi: 10.1183/09031936.99.14510029 [published Online First: 1999/12/22]
41. Mullerova H, Chigbo C, Hagan GW, et al. The natural history of community-acquired pneumonia in COPD patients: a population database analysis. *Respir Med* 2012;106(8):1124-33. doi: 10.1016/j.rmed.2012.04.008 [published Online First: 2012/05/25]
42. Oxygen therapy for children. Geneva, Switzerland, 2016.
43. Graham H, Bakare AA, Ayede AI, et al. Hypoxaemia in hospitalised children and neonates: A prospective cohort study in Nigerian secondary-level hospitals. *EClinicalMedicine* 2019;16:51-63. doi: 10.1016/j.eclinm.2019.10.009 [published Online First: 2019/12/14]
44. Howie SR, Hill S, Ebonyi A, et al. Meeting oxygen needs in Africa: an options analysis from the Gambia. *Bull World Health Organ* 2009;87(10):763-71. doi: 10.2471/blt.08.058370
45. Rojas-Reyes MX, Granados Rugeles C, Charry-Anzola LP. Oxygen therapy for lower respiratory tract infections in children between 3 months and 15 years of age. *Cochrane Database Syst Rev* 2014;2014(12):Cd005975. doi: 10.1002/14651858.CD005975.pub3 [published Online First: 2014/12/11]
46. O'Gara PT, Kushner FG, Ascheim DD, et al. 2013 ACCF/AHA guideline for the management of ST-elevation myocardial infarction: a report of the American College of Cardiology Foundation/American Heart Association Task Force on Practice Guidelines. *Circulation* 2013;127(4):e362-425. doi: 10.1161/CIR.0b013e3182742cf6 [published Online First: 2012/12/19]
47. Cam BV, Tuan DT, Fonsmark L, et al. Randomized comparison of oxygen mask treatment vs. nasal continuous positive airway pressure in dengue shock syndrome with acute respiratory failure. *J Trop Pediatr* 2002;48(6):335-9. doi: 10.1093/tropej/48.6.335 [published Online First: 2003/01/11]
48. PROTOCOL FOR THE MANAGEMENT OF ACUTE MALNUTRITION: Ministry of Health, Republic of Rwanda, 2018.
49. O'Driscoll BR, Howard LS, Earis J, et al. BTS guideline for oxygen use in adults in healthcare and emergency settings. *Thorax* 2017;72(Suppl 1):ii1-ii90. doi: 10.1136/thoraxjnl-2016-209729 [published Online First: 2017/05/17]
50. Guan WJ, Ni ZY, Hu Y, et al. Clinical Characteristics of Coronavirus Disease 2019 in China. *N Engl J Med* 2020;382(18):1708-20. doi: 10.1056/NEJMoa2002032 [published Online First: 2020/02/29]
51. Huth SF, Rothkopf A, Smith L, et al. Variability of oxygen requirements in critically ill COVID-19 patients. *J Glob Health* 2024;14:05012. doi: 10.7189/jogh.14.05012 [published Online First: 2024/02/23]
52. Long term domiciliary oxygen therapy in chronic hypoxic cor pulmonale complicating chronic bronchitis and emphysema. Report of the Medical Research Council Working Party. *Lancet* 1981;1(8222):681-6. [published Online First: 1981/03/28]
53. Meara JG, Leather AJM, Hagander L, et al. Global Surgery 2030: evidence and solutions for achieving health, welfare, and economic development. *The Lancet* 2015;386(9993):569-624. doi: 10.1016/s0140-6736(15)60160-x
54. Graham HR, Kamuntu Y, Miller J, et al. Hypoxaemia prevalence and management among children and adults presenting to primary care facilities in Uganda: A prospective cohort study. *PLOS Glob Public Health* 2022;2(4):e0000352. doi: 10.1371/journal.pgph.0000352
55. Olojede OE, Falade AG, Bakare AA, et al. Understanding pulse oximetry adoption in primary healthcare facilities in Nigeria: a realist process evaluation of the INSPIRING-Lagos stabilisation room project. *BMJ Public Health* 2024;2(2) doi: 10.1136/bmjph-2024-001211
56. Colbourn T, Falade AG, Graham HR, et al. Pulse oximetry and oxygen services for under-five children with community-acquired pneumonia attending primary and secondary level health facilities in Lagos, Nigeria (INSPIRING-Lagos): a pre-implementation and post implementation study. *BMJ Public Health* 2024;2(2) doi: 10.1136/bmjph-2024-001210
57. Hedible GB, Anago G, Lenaud S, et al. What were the challenges and needs before implementing routine pulse oximetry in IMCI consultations at primary health centres in West Africa? Baseline site assessment of the operational AIRE project, 2020. *medRxiv* 2024 doi: 10.1101/2024.10.14.24315436
58. Barasa E, Kairu A, Ng'ang'a W, et al. Examining unit costs for COVID-19 case management in Kenya. *BMJ Global Health* 2021;6(4):e004159. doi: 10.1136/bmjgh-2020-004159

59. Bradley BD, Light JD, Ebonyi AO. Implementation and 8-year follow-up of an uninterrupted oxygen supply system in a hospital in The Gambia. ... *of Tuberculosis and* ... 2016
60. Davidescu AA, Apostu SA, Stanciu-Mandruleanu C. Shedding Light on the Main Characteristics and Perspectives of Romanian Medicinal Oxygen Market. *Healthcare (Basel)* 2021;9(2) doi: 10.3390/healthcare9020155 [published Online First: 2021/02/07]
61. Duke T, Peel D, Wandi F, et al. Oxygen supplies for hospitals in Papua New Guinea: a comparison of the feasibility and cost-effectiveness of methods for different settings. *P N G Med J* 2010;53(3-4):126-38.
62. Graham HR, Olojede OE, Bakare AA, et al. Measuring Oxygen Access: lessons from health facility assessments in Nigeria. medRxiv, 2021.
63. Kizza D, Mushumbamwiza H, Ndwandwe S, et al. Financial Implications of Tariffs for Medical Oxygen on Rwandan Public Hospitals' Finance Management During the Coronavirus Epidemic. *Glob Health Sci Pract* 2022;10(5) doi: 10.9745/ghsp-d-22-00058 [published Online First: 20221031]
64. Manhas V, Rawat M, Kaurav YS, et al. Cost analysis of different medical oxygen sources for a healthcare facility in India. *Indian Journal of Anaesthesia* 2024;68(4)
65. Nyende S, Conroy A, Opoka RO, et al. Solar-powered oxygen delivery: Study protocol for a randomized controlled trial. *Trials* 2015;16:297. doi: http://dx.doi.org/10.1186/s13063-015-0814-y
66. Parmar J, Pawar V, Warathe A, et al. Rationalising oxygen usage in a level II special newborn care unit in Madhya Pradesh, India. *BMJ Open Qual* 2021;10(Suppl 1) doi: 10.1136/bmjoq-2021-001386
67. Smith V, Changoor A, McDonald C, et al. A Comprehensive Approach to Medical Oxygen Ecosystem Building: An Implementation Case Study in Kenya, Rwanda, and Ethiopia. *Glob Health Sci Pract* 2022;10(6) doi: 10.9745/ghsp-d-21-00781 [published Online First: 20221221]
68. Tesfaye SH, Loha E, Johansson KA, et al. Cost-effectiveness of pulse oximetry and integrated management of childhood illness for diagnosing severe pneumonia. *PLOS Global Public Health* 2022;2(7):e0000757. doi: 10.1371/journal.pgph.0000757
69. Graham HR, Ayede AI, Bakare AA, et al. Oxygen for children and newborns in non-tertiary hospitals in South-west Nigeria: A needs assessment. *Afr J Med Med Sci* 2016;45(1):31-49.
70. Malawi Ministry of H. Malawi National Medical Oxygen Ecosystem Roadmap 2021-2026. Lilongwe, Malawi, 2021.
71. Gray AZ, Morpeth M, Duke T, et al. Improved oxygen systems in district hospitals in Lao PDR: a prospective field trial of the impact on outcomes for childhood pneumonia and equipment sustainability. *BMJ Paediatrics Open* 2017;1(1):e000083. doi: 10.1136/bmjpo-2017-000083
72. Schumacher Shawn D, Brockwell Russell C, Andrews JJ, et al. Bulk Liquid Oxygen Supply Failure. *Anesthesiology* 2004;100(1):186-89. doi: 10.1097/00000542-200401000-00032
73. Bakare AA, Graham H, Ayede AI, et al. Providing oxygen to children and newborns: a multi-faceted technical and clinical assessment of oxygen access and oxygen use in secondary-level hospitals in southwest Nigeria. *International Health* 2020;12(1):60-68. doi: 10.1093/INTHEALTH/IHZ009
74. Graham HR, Bakare AA, Ayede AI, et al. Oxygen systems to improve clinical care and outcomes for children and neonates: A stepped-wedge cluster-randomised trial in Nigeria. *PLoS Med* 2019;16(11):e1002951. doi: 10.1371/journal.pmed.1002951 [published Online First: 20191111]
75. Rodríguez DC, Balaji LN, Chamdimba E, et al. Political economy analysis of subnational health management in Kenya, Malawi and Uganda. *Health Policy and Planning* 2023;38(5):631-47. doi: 10.1093/heapol/czad021
76. Clark JM, Sanders S, Carter M, et al. Improving the translation of search strategies using the Polyglot Search Translator: a randomized controlled trial. *J Med Libr Assoc* 2020;108(2):195-207. doi: 10.5195/jmla.2020.834 [published Online First: 20200401]
77. Bramer WM, Giustini D, de Jonge GB, et al. De-duplication of database search results for systematic reviews in EndNote. *J Med Libr Assoc* 2016;104(3):240-3. doi: 10.3163/1536-5050.104.3.014
78. Goodrick D. Comparative case studies: Methodological briefs-Impact evaluation No. 9, 2014.
79. Wolrd Health Organisation. WHA76.3 Resolution: Increasing Access to Medical Oxygen Geneva, Switzerland: Wolrd Health Organisation,, 2023.
80. Smith CE, Hill SE, Amos A. Impact of population tobacco control interventions on socioeconomic inequalities in smoking: a systematic review and appraisal of future research directions. *Tobacco Control* 2021;30(e2):e87-e95.
81. Frost LJ, Reich MR. Access: how do good health technologies get to poor people in poor countries?: Harvard Center for Population and Development Studies 2008.
82. Mswati IK, III. An Africa free of malaria. *The Lancet* 2019;394(10203):988-89. doi: 10.1016/S0140-6736(19)31952-X

83. Gabrysch S, Civitelli G, Edmond KM, et al. New signal functions to measure the ability of health facilities to provide routine and emergency newborn care. *PLoS Med* 2012;9(11):e1001340. doi: 10.1371/journal.pmed.1001340 [published Online First: 2012/11/16]
84. Rahman AE, Banik G, Mhajabin S, et al. Newborn signal functions in Bangladesh: identification through expert consultation and assessment of readiness among public health facilities-study protocol using Delphi technique. *BMJ Open* 2020;10(9):e037418. doi: 10.1136/bmjopen-2020-037418 [published Online First: 2020/09/03]
85. Tembo T, Chongwe G, Vwalika B, et al. Signal functions for emergency obstetric care as an intervention for reducing maternal mortality: a survey of public and private health facilities in Lusaka District, Zambia. *BMC pregnancy and childbirth* 2017;17(1):288. doi: 10.1186/s12884-017-1451-0 [published Online First: 2017/09/08]
86. Global Health Observatory Data Repository (Region of the Americas). Biomedical engineering and technician personnel: Data by country. In: World Health Organisation, ed., 2018.
87. WHO. Global strategy on human resources for health: workforce 2030. Geneva: World Health Organization (WHO), 2016.
88. WHO. Human resources for medical devices: the role of biomedical engineers. WHO Medical device technical series. Geneva: World Health Organization (WHO), 2017.
89. Bae C, Pigoga JL, Cox M, et al. Evaluating emergency care capacity in Africa: an iterative, multicountry refinement of the Emergency Care Assessment Tool. *BMJ Glob Health* 2018;3(5):e001138. doi: 10.1136/bmjgh-2018-001138 [published Online First: 20181015]
90. University of California San Francisco (UCSF), Center for Health Equity in Surgery and Anesthesia (CHESA). UCSF-CHESA Critical Care Facility Level Assessment Tool
91. Baker T, Lugazia E, Eriksen J, et al. Emergency and critical care services in Tanzania: a survey of ten hospitals. *BMC Health Serv Res* 2013;13:140. doi: 10.1186/1472-6963-13-140 [published Online First: 20130416]
92. Mock C, Lormand JD, Goosen J, et al. Guidelines for essential trauma care. Geneva. In: World Health Organization, ed., 2004.
93. World Health Organization. Needs Assessment and Evaluation Form for Resource Limited Health Care Facility (Annexe 3) of WHO Workshop on Integrated Management on Emergency and Essential Surgical Care (IMEESC) in collaboration with Ministry of Health, Kyrgyzstan, 2005.
94. World Health Organization. Global Initiative for Emergency and Essential Surgical Care (GIEESC). Tool for Situational Analysis to Assess Emergency and Essential Surgical Care (TSAAEESC). In: Organization WH, ed., 2010.
95. Osen H, Chang D, Choo S, et al. Validation of the World Health Organization tool for situational analysis to assess emergency and essential surgical care at district hospitals in Ghana. *World J Surg* 2011;35(3):500-4. doi: 10.1007/s00268-010-0918-1
96. World Health Organization. WHO integrated management for emergency and surgical care (IMEESC toolkit [electronic format]. Geneva: World Health Organization, 2011.
97. Kushner AL, Cherian MN, Noel L, et al. Addressing the Millennium Development Goals from a surgical perspective: essential surgery and anesthesia in 8 low- and middle-income countries. *Arch Surg* 2010;145(2):154-9. doi: 10.1001/archsurg.2009.263
98. World Health Organization. Hospital Emergency Unit Assessment Tool (HEAT) 2017.
99. Lin Y, Raykar NP, Saluja S, et al. Identifying essential components of surgical care delivery through quality improvement: An updated surgical assessment tool. *Int J Surg* 2020;82:103-07. doi: 10.1016/j.ijsu.2020.08.002 [published Online First: 20200815]
100. World Health Organization. Surgical Assessment Tool.
101. Surgeons OverSeas. PIPES Surgical Assessment
102. Surgeons OverSeas. Pedi PIPES Assessment of Pediatric Surgical Capacity
103. Markin A, Barbero R, Leow JJ, et al. Inter-rater reliability of the PIPES tool: validation of a surgical capacity index for use in resource-limited settings. *World J Surg* 2014;38(9):2195-9. doi: 10.1007/s00268-014-2522-2
104. World Federation of Societies of Anaesthesiologists. WFSA Anaesthesia Facility Assessment Tool (AFAT) v1.1. In: (WFSA), ed., 2018.
105. World Health Organization. Rapid hospital readiness checklist for COVID-19. Geneva: World Health Organization, 2020.
106. World Health Organization. Biomedical equipment for COVID-19 case management: inventory tool: harmonized health service capacity assessments in the context of the COVID-19 pandemic: interim guidance, 25 June 2020. Geneva: World Health Organization, 2020.

107. Fung JST, Hwang B, Dunsmuir D, et al. A 2-Phase Survey to Assess a Facility's Readiness for Pediatric Essential Emergency and Critical Care in Resource-Limited Settings: A Literature Review and Survey Development. *Pediatr Emerg Care* 2022;38(10):532-39. doi: 10.1097/PEC.0000000000002826 [published Online First: 20220818]
108. World Health Organization. Improving quality of paediatric care in small hospitals in developing countries : report of a meeting, Geneva, 19-21 June 2000. Geneva: World Health Organization, 2001.
109. World Health Organization. Indicators and standards for paediatric hospital care, Annex 8 of Improving paediatric referral care in the context of child survival activities and IMCI : review of processes to improve paediatric care in small hospitals in developing countries : report of a global meeting, Denpasar, Indonesia, 15-19 January 2007. Geneva: World Health Organization, 2008.
110. World Health Organization. Regional Office for Europe. Hospital care for children: quality assessment and improvement tool: a systematic standard based participatory approach. 2nd Edition. Copenhagen: World Health Organization. Regional Office for Europe., 2015:125 p.
111. World Health Organization. South East Asia Region (SEARO). Assessment tool for hospital care: improving the quality of care for reproductive, maternal, neonatal, child and adolescent health in South-East Asia. New Delhi: World Health Organization. Regional Office for South-East Asia 2016.
112. World Health Organization. Regional Office for Europe. Hospital care for mothers and newborn babies: quality assessment and improvement tool a systematic standard based participatory approach, 2nd edition. In: WHO Regional Office for Europe., ed., 2014.
113. World Health Organization. Harmonized health facility assessment (HHFA): Combined questionnaire (Availability, Readiness, and Management and Finance) Core and additional questions. V2.1. In: WHO, ed., 2023.
114. World Health Organization. Service availability and readiness assessment (SARA). v2.2, 2015.
115. World Health Organization. Health Resources and Services Availability Monitoring System (HeRAMS).
116. The DHS Program. Service Provision Assessment (SPA). In: Demographic and Health Serveys TDP, USAID, ed., 2022.
117. World Health Organization (WHO), United Nations Children's Fund (UNICEF). Web Annex. Technical specifications. In: Primary health care measurement framework and indicators:

monitoring health systems through a primary health care lens. In: (UNICEF) WHOatUNCsF, ed. Geneva, 2022.
118. Meeting Targets and Maintaining Epidemic Control (EpiC). Assessing the medical oxygen ecosystem: tools from national to primary health care levels. In: FHI 360., ed. Durham (NC), US, 2022.
119. World Health Organization, South East Asia Region (SEARO), Integrated Management of Adult and Adolescent Illness (IMAI) Alliance. SEARO-IMAI Oxygen Hospital Survey tool 2020.
120. Clinton Health Access Initiative (CHAI). Facility Oxygen Equipment Availability Assessment. In: Assessment CEOE, ed., 2022.
121. La Vincente SF, Peel D, Carai S, et al. The functioning of oxygen concentrators in resource-limited settings: a situation assessment in two countries. *Int J Tuberc Lung Dis* 2011;15(5):693-9. doi: http://dx.doi.org/10.5588/ijtld.10.0544
122. PATH. Baseline Assessment Survey. Oxygen Delivery Toolkit: Resources to plan and scale medical oxygen, 2020.
123. Bagayana S, Subhi R, Moore G, et al. Technology to improve reliable access to oxygen in Western Uganda: study protocol for a phased implementation trial in neonatal and paediatric wards. *BMJ Open* 2022;12(6):e054642. doi: 10.1136/bmjopen-2021-054642 [published Online First: 2022/06/30]
124. Bakare AA, Graham H, Ayede AI, et al. Providing oxygen to children and newborns: a multi-faceted technical and clinical assessment of oxygen access and oxygen use in secondary-level hospitals in southwest Nigeria. *Int Health* 2020;12(1):60-68. doi: 10.1093/inthealth/ihz009
125. Graham HR, Ayede AI, Bakare AA, et al. Improving oxygen therapy for children and neonates in secondary hospitals in Nigeria: study protocol for a stepped-wedge cluster randomised trial. *Trials* 2017;18(1):502. doi: 10.1186/s13063-017-2241-8
126. Graham HR, Olojede OE, Bakare AA, et al. Measuring oxygen access: lessons from health facility assessments in Lagos, Nigeria. *BMJ Glob Health* 2021;6(8) doi: 10.1136/bmjgh-2021-006069
127. Graham HR, Olojede OE, Bakare AAA, et al. Pulse oximetry and oxygen services for the care of children with pneumonia attending frontline health facilities in Lagos, Nigeria (INSPIRING-Lagos): study protocol for a mixed-methods evaluation. *BMJ Open* 2022;12(5):e058901. doi: 10.1136/bmjopen-2021-058901 [published Online First: 2022/05/03]

128. Nabwire J, Namasopo S, Hawkes M. Oxygen Availability and Nursing Capacity for Oxygen Therapy in Ugandan Paediatric Wards. *J Trop Pediatr* 2018;64(2):97-103. doi: 10.1093/tropej/fmx033
129. Coyle RM, Harrison H-L. Emergency care capacity in Freetown, Sierra Leone: a service evaluation. *BMC Emergency Medicine* 2015;15(1):2. doi: 10.1186/s12873-015-0027-4
130. Solis C, Leon P, Sanchez N, et al. Nicaraguan surgical and anesthesia infrastructure: survey of Ministry of Health hospitals. *World J Surg* 2013;37(9):2109-21. doi: 10.1007/s00268-013-2112-8
131. Iverson KR, Ahearn O, Citron I, et al. Development of a surgical assessment tool for national policy monitoring & evaluation in Ethiopia: A quality improvement study. *Int J Surg* 2020;80:231-40. doi: 10.1016/j.ijsu.2020.03.025 [published Online First: 20200319]
132. Wong EG, Gupta S, Deckelbaum DL, et al. The International Assessment of Capacity for Trauma (INTACT): an index for trauma capacity in low-income countries. *J Surg Res* 2014;190(2):522-7. doi: 10.1016/j.jss.2014.01.060 [published Online First: 20140204]
133. Sonenthal PD, Masiye J, Kasomekera N, et al. COVID-19 preparedness in Malawi: a national facility-based critical care assessment. *Lancet Glob Health* 2020;8(7):e890-e92. doi: 10.1016/S2214-109X(20)30250-3 [published Online First: 20200525]
134. Bredow Z, Corbett Z, Tarawally MM, et al. Emergency care capacity in Sierra Leone: A multicentre analysis. *Afr J Emerg Med* 2024;14(1):58-64. doi: 10.1016/j.afjem.2024.01.003 [published Online First: 20240206]
135. Ploss B, Abdelgadir J, Smith ER, et al. Pilot Use of a Novel Tool to Assess Neurosurgical Capacity in Uganda. *World Neurosurg* 2017;108:844-49 e4. doi: 10.1016/j.wneu.2017.08.045 [published Online First: 20170818]
136. World Health Organization. Suite of health service capacity assessments in the context of the COVID-19 pandemic, interim guidance, 2 November 2020. Geneva: World Health Organization, 2020.
137. World Health Organization. Harmonized modules for health facility assessment modules in the context of the COVID-19 pandemic: interim guidance, 31 May 2020. Geneva: World Health Organization, 2020.
138. Every Breath Counts Coalition. Lancet Global Health Oxygen Commission 2022 [Available from: https://stoppneumonia.org/latest/lancet-global-health-oxygen-commission/ accessed 01/05/2024 2024.
