# Supplementary material for: Reducing global inequities in medical oxygen access: the Lancet Global Health Commission on medical oxygen security
Source: Lancet Glob Health. 2025 Feb 17;13(3):e528–84. doi: 10.1016/S2214-109X(24)00496-0 (PMC11865010; doi:10.1016/S2214-109X(24)00496-0)
Supplement: Supplementary appendix 2 [file mmc2.pdf]

THE LANCET
Global Health

Supplementary appendix 2

This appendix formed part of the original submission and has been peer reviewed. We post it as supplied by the authors.

Supplement to: Graham HR, Carina King C, Rahman AE, et al. Reducing global inequities in medical oxygen access: the *Lancet Global Health* Commission on medical oxygen security. *Lancet Glob Health* 2025; published online Feb 17. https://doi.org/10.1016/S2214-109X(24)00496-0.

THE LANCET Global Health
COMMISSION ON MEDICAL OXYGEN SECURITY

Appendix 2: Country Case Studies

Table of Contents

Bangladesh 2
India 24
Malawi 45
Nigeria 65
Sweden 92
Uganda 108

The Peru country case study can be found here: Garcia PJ, Kitutu FE, Guzman JM, Najarro L, Ssengooba S, King C. Challenges in the medical oxygen ecosystem of Peru: a political economy analysis. medRxiv 2025; https://doi.org/10.1101/2025.01.02.25319915

THE LANCET Global Health
COMMISSION ON MEDICAL OXYGEN SECURITY

Country Case Study: Bangladesh

A success story of integrating pulse oximetry into IMCI

Ahmed Ehsanur Rahman[1], Shafiqul Ameen[1], Sabit Saad Shafiq[1], Aniqa Tasnim Hossain[1], Shams El Arifeen[1], Carina King[2]

1: International Centre for Diarrheal Disease Research, Bangladesh (icddr,b); **2:** Department of Global Public Health, Karolinska Institutet, Sweden

Case study focus

This case study focuses on the incorporation and expansion of pulse oximetry within the Integrated Management of Childhood Illnesses (IMCI) programme in Bangladesh. The effectiveness of IMCI heavily depends upon the diagnostic proficiency of healthcare providers, to mitigate missed and misdiagnoses and the administration of inappropriate treatments.[1] It is noteworthy that hypoxaemia is prevalent in over 30% of children diagnosed with pneumonia – a key focus condition of IMCI.[2,3] And pulse oximetry, a non-invasive method for estimating and monitoring peripheral oxygen saturation (SpO_2) at the point of care, is the simplest way to measure hypoxaemia.[4,5] The integration of pulse oximetry in IMCI settings can therefore significantly improve the classification accuracy of pneumonia.[5] In recognition of the importance of pulse oximetry, the World Health Organisation (WHO) revised the IMCI guidelines in 2014 to incorporate pulse oximetry in the assessment of pneumonia symptoms in children aged 2-59 months, supplementing clinical assessment.[6,7] The impact of this integration is substantiated by a study conducted in Malawi, which reported that 69% of hypoxaemic children at health centres would have been deemed ineligible for referral if assessed solely based on clinical assessments without pulse oximetry.[8]

Presently, however the WHO's 2014 IMCI guidelines include pulse oximetry only as a footnote, suggesting that if an oximeter is available, it should be used as part of the clinical pneumonia assessment. The 2024 recommendations have not changed this. The large-scale adoption and implementation of pulse oximetry within IMCI has so far been limited due to various concerns and barriers. This case study, therefore, focuses on the successful example of Bangladesh, where IMCI is the primary healthcare strategy for children presenting with pneumonia. The Bangladeshi government has made joint efforts to expand the scope of IMCI healthcare units by including pulse oximetry. This initiative holds significant relevance for Bangladesh, and other low to middle-income countries (LMICs) can glean valuable insights from it. The case study underscores the potential of pulse oximetry as a critical tool in improving the diagnosis and treatment of pneumonia within the IMCI framework.

Country Context

Demography, economy, and epidemiology

The national source of causes of deaths for all ages in Bangladesh is the Sample Vital Registration System (SVRS). However, SVRS does not follow the World Health Organization (WHO) standard tool for verbal autopsy to report causes of death distribution,[9] and it does not follow the International Classification of Diseases (ICD-10) codes for assigning causes – somewhat limiting comparisons to other settings.[10] According to the most recent all age causes of death data from SVRS 2021, five causes contributed to around half of all deaths in Bangladesh:[11] heart attack (23%); cause-specific mortality rate (CSMR) 103 per 100,000 population); respiratory disease (12%; CSMR 52 per 100,000 population), brain stroke (10%; CSMR 45 per 100,000 population), asthma (6%; CSMR 29 per 100,000 population) and pneumonia (5%; CSMR 24 per 100,000 population) - Figure 1.

Data from other large population surveys and Health and Demographic Surveillance Sites (HDSS) in Matlab, Chakariya, Baliakandi, Sitankundu and Dhaka have reported similar top causes of death, with strokes, heart disease and respiratory diseases consistently in the top 5. Of particular note for this case study, key causes of death linked to oxygen needs such as respiratory disease, asthma and pneumonia consistently account for over 1 in 5 deaths. [11,12]

Indicator	Recent value	Data source
Total population	165 million	Population and Housing Census 2022 [13]
Total under-five population	15,590,973	Population and Housing Census 2022 [13]
Under-five mortality	31 deaths per 1,000 live births	BDHS 2022 [14]
Life expectancy (m:f)	Overall: 72.4 years<br>Male:70.8 years<br>Female: 74.2 years	SVRS 2022 [15]
GDP per capita	2,688 US$	The World Bank [16]
Healthcare expenditure per capita	54 US$	BNHA 1997-2020 [17]
Income status	Lower-middle-income Country	The World Bank [18]

Table 1: Overview of Bangladesh's demography and economy

The Bangladesh Maternal Mortality and Health Care Survey (BMMS) 2016 revealed that around 6,577 women die annually in Bangladesh due to maternal causes – Figure 2. Two top causes haemorrhage (31%) and eclampsia (23%) accounted for half of the maternal deaths.[19] Indirect causes, including stroke, cancer, heart disease, and asthma, contributed to 21% of the maternal deaths.[20] The Bangladesh Demographic and Health Survey (BDHS) 2022 is a nationally representative survey which provide causes of under-five deaths.[14] This survey uses the WHO's 2016 verbal autopsy questionnaire, and trained physicians assigned the causes of 502 under five deaths using ICD-10 codes.[10] According to the BDHS 2022, two-thirds of the under-five deaths are caused by pneumonia, prematurity and low-birth-weight (LBW) and birth asphyxia,[14] with pneumonia contributing 24% of the under-five deaths (approximately 26,000 deaths). Prematurity and LBW (22%) accounted for 25,000 deaths and birth asphyxia (18%) accounted for 21,000 under-five deaths (Figure 3).

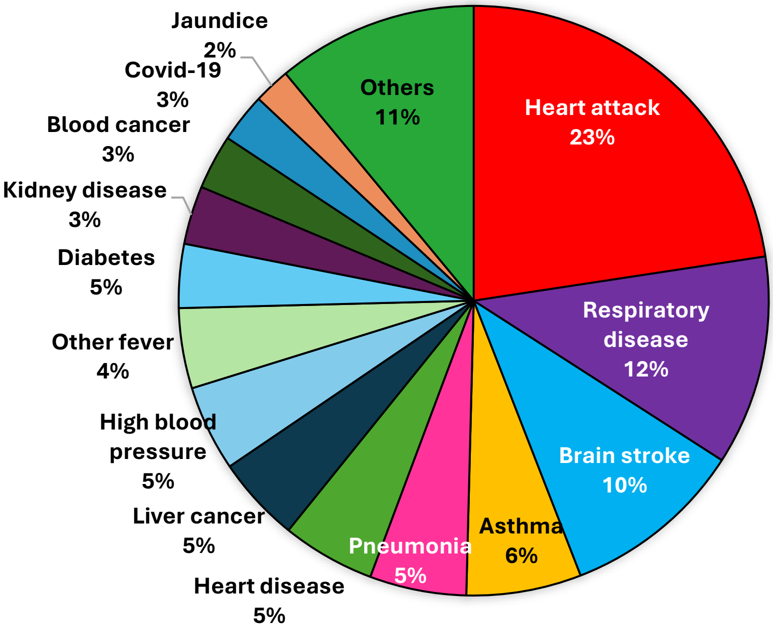

Figure 1: Causes of deaths in Bangladesh, presented in percentages from the 2021 SVRS

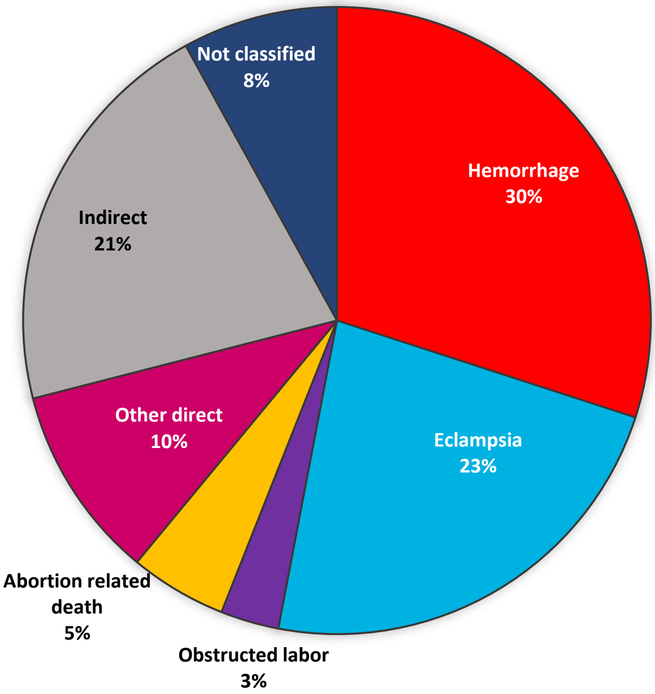

Figure 2: Causes of maternal deaths, presented in percentage from BMMS 2016 (n=175)

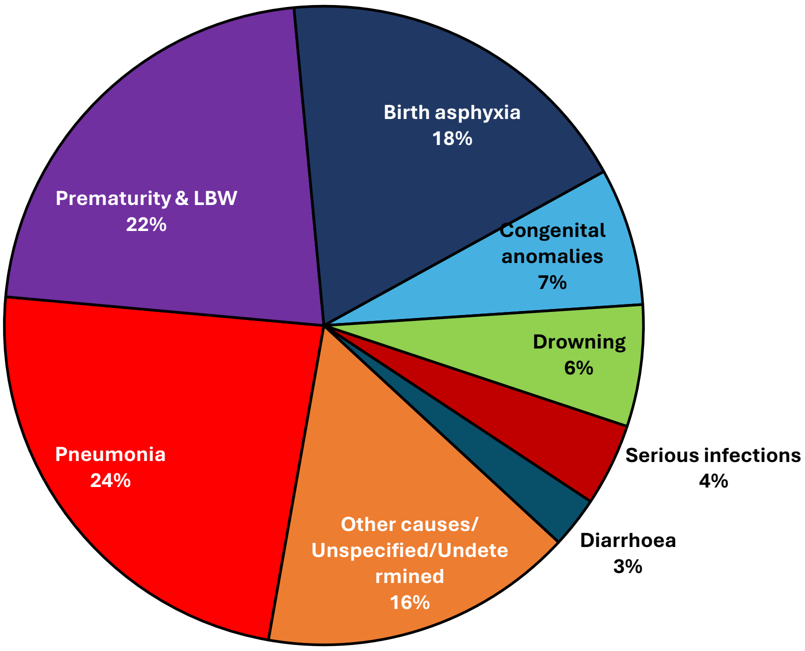

Figure 3: Causes of under-five deaths, presented in percentage from BDHS 2022 (n=502)

Hypoxaemia burden

Hypoxaemia, based on the WHO definition of SpO_2<90%, is a common complication of pneumonia and other acute lower respiratory infections (ALRIs). The most recent global estimate of the prevalence of hypoxaemia for the low- and middle-income countries was 31% among all children with WHO-classified pneumonia.[3] The pooled estimate from the five papers from Bangladesh in this systematic review estimated 52% of children with WHO-classified pneumonia were hypoxaemic (Figure 4), considerably higher than the global estimate. However, 3 of the 5 studies were based on hospitalised patients, and one study included both hospitalised and non-hospitalised patients, therefore representing a severely sick population. A study conducted in outpatient clinics of Bangladesh reported 3% of children aged 3–11 months with suspected pneumonia had hypoxaemia based on the WHO-recommended cut-off of SpO_2<90%. Another 8% of the children had an SpO_2 of 90-93%.[21] Another secondary analysis of data from 2646 patients admitted to icddr,b-Dhaka Hospital, a secondary level referral hospital located in Dhaka, conducted in 2021, reported a high hypoxaemia prevalence of 40% among children hospitalised with severe pneumonia on admission.[22]

The seasonal variation in hypoxaemia prevalence between 2014 and 2017 observed in the icddr,b-Dhaka Hospital found no notable seasonal pattern, although the average annual prevalence increased from 32% in 2014 to 51% in 2017 (Figure 5). There could be several factors contributing to this. The introduction of pneumococcal conjugate vaccines (PCVs) and Haemophilus influenzae type b (Hib) vaccines in national immunization programs across several countries, including Bangladesh, has significantly reduced the incidence of serious bacterial pneumonias. Consequently, respiratory syncytial virus (RSV) and other viruses have become more dominant pathogens causing severe pneumonia in children. This shift in pneumonia aetiology may impact clinical features, including hypoxemia. Approximately 20% of hospitalized children with RSV-associated acute LRIs exhibit hypoxemia.[23] The changes in hypoxemia prevalence could also be attributed to improved pneumonia care-seeking practices in Bangladesh. Parents are now more aware of pneumonia-related symptoms and complications, leading to better identification of complicated cases through hospital-based assessments.[24]

Among these children, 6% died during the hospital stay, and 9% were referred to higher-level facilities due to clinical deterioration. Hypoxaemia was found to be the strongest predictor of mortality with 11 times higher odds of death in these children.[22]

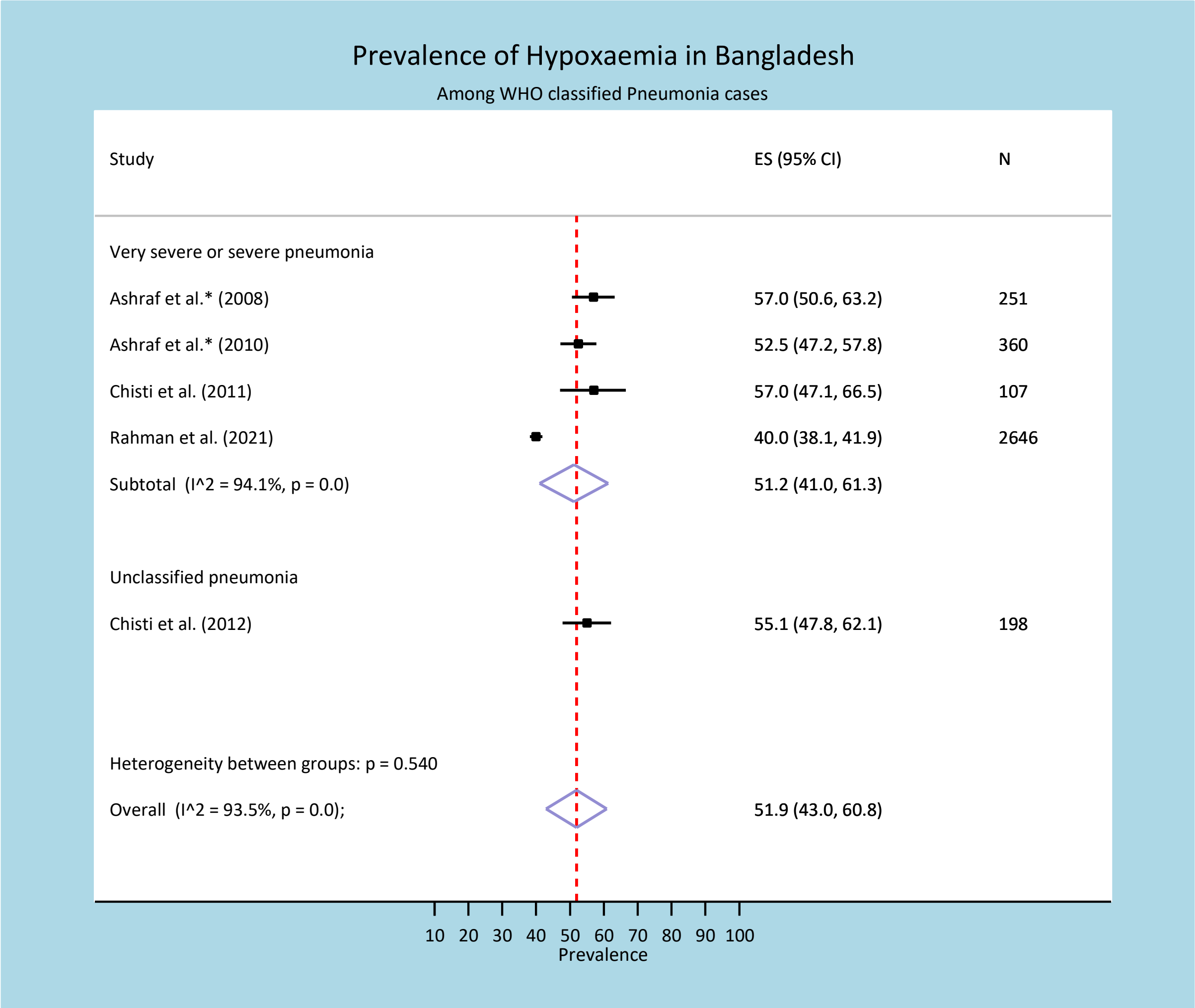

Figure 4: Hypoxaemia prevalence among children with WHO-classified pneumonia in Bangladesh by clinical severity. *Studies where SpO_2 cut-off is greater than 90%.

An observational study was also conducted at a secondary level referral district hospital in Kushtia of Bangladesh on children aged 0-59 months who were admitted to the paediatric inpatient unit or visited the emergency department (*unpublished data*). Among the 2,025 indoor patients, the prevalence of hypoxaemia was 42% among children aged 2 to 59 months with critical illness or clinical severe infection, and 40% among fast breathing pneumonia patients of 0 to 59 days, compared to 18% among children of same age without any signs of pneumonia. For children aged 2 to 59 months, 11% of admitted children with severe or non-severe pneumonia had hypoxaemia, compared to 4% among admitted children without pneumonia (Figure 6). This analysis again found hypoxaemia to be the strongest predictor of mortality with 3 times higher odds of death.

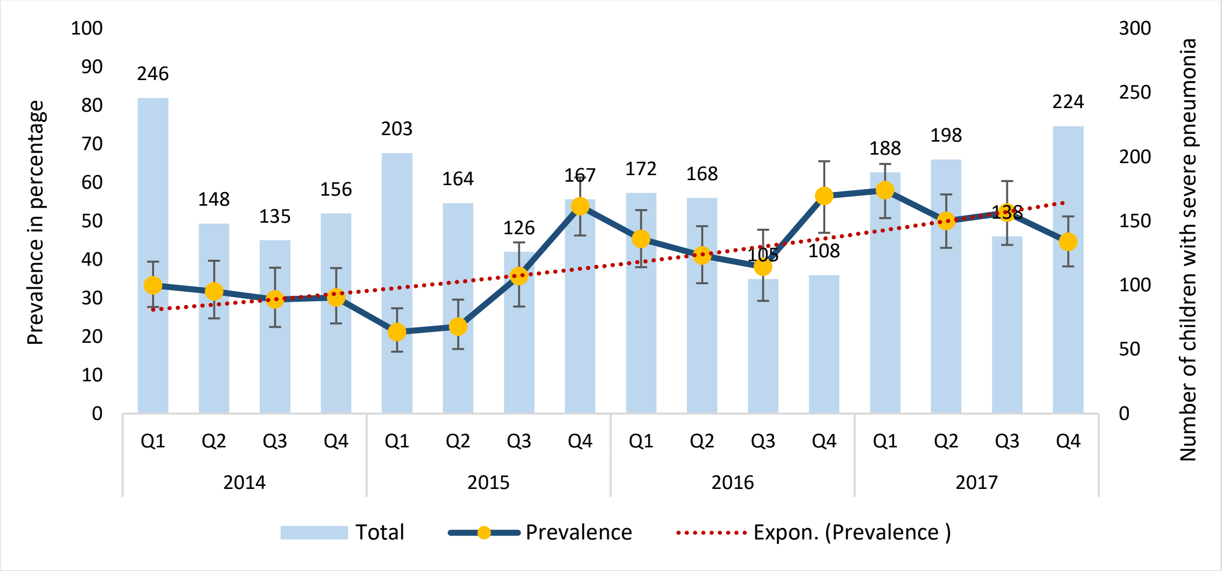

Figure 5: Hypoxaemia prevalence among children aged 2-59 months admitted to icddr,b Dhaka Hospital with WHO-defined severe pneumonia between 2014 and 2017

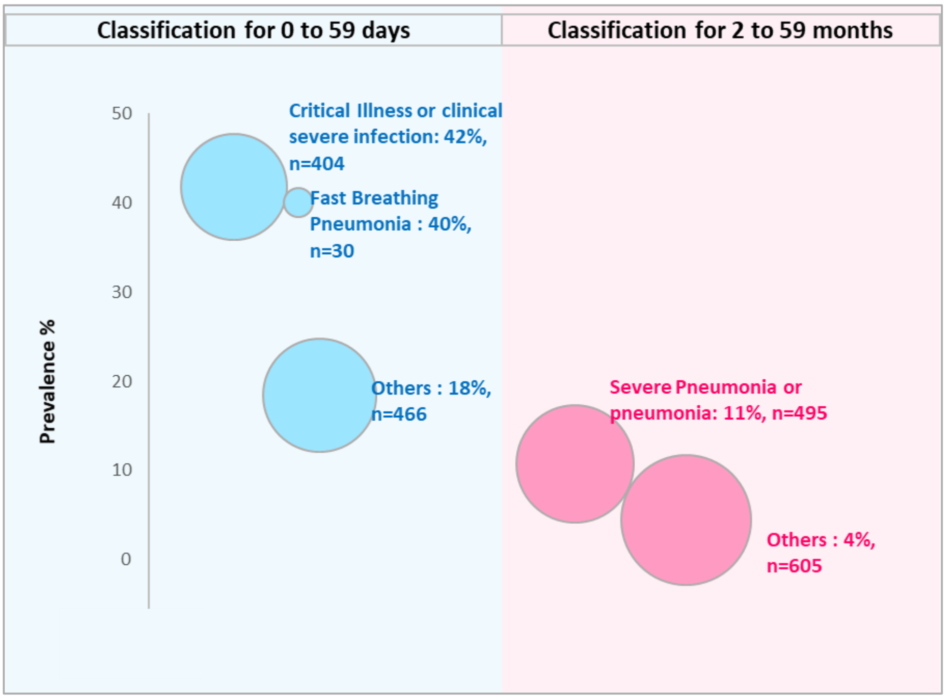

Figure 6: Prevalence of hypoxaemia among children with pneumonia disaggregated by age and clinical severity at Kushtia District Hospital in Bangladesh

Health system

The administrative structure of Bangladesh is hierarchical, cascading from divisions to villages. Each division is subdivided into districts, then upazilas (sub-districts), and down to unions and villages. There are a total of seven divisions, 64 districts, 495 upazilas, and approximately 4671 unions and 87,320 villages in Bangladesh. The healthcare system in Bangladesh adopts a pluralistic approach, involving a diverse range of stakeholders. These include the government, profit-driven private sector entities, non-profit organizations, and international development agencies.[25]

The Ministry of Health and Family Welfare (MOH&FW) oversees the health system. It has two major wings: The Health Services Division, which manages clinical and public health services, and the Medical Education and Family Welfare Division, which focuses on family planning work, training and capacity development, and enhancing research programmes.[25] Under the MOH&FW, there are five regulatory bodies and nine implementing agencies. The two major implementing agencies are the Director General of Health Services (DGHS) and the Directorate General of Family Planning (DGFP).[25] All six levels of the Bangladeshi government—national, divisional, district, sub-district, union, and village—are under the supervision of the Directorate General of Health Services and the Directorate General of Family Planning.

The capacity of secondary-level district hospitals ranges from approximately 100 to 250 beds. Beneath this, facilities known as Upazila health Complexes have 30-50 beds,[25] and at the union level, Union Health and Family Welfare Centres (UH&FWCs) facilities are responsible for providing fundamental preventative and curative treatment, and are maintained by Sub-Assistant Community Medical Officers (SACMO). Family Welfare Visitors (FWV) are those who are primarily concerned with the reproductive and maternal health of the communities they visit. Community Health Care Providers (CHCP) are responsible for the operation of Community Clinics (CCs) and provide basic healthcare, as well as health education and family planning services.

Position	Number
Total DGHS Staff	78,227
Sanctioned Posts	115,272
Doctors	26,791
Community Healthcare Providers (CHCPs)	13,948
Sub-assistant Community Medical Officers (SACMOs)	3,616
Health Inspectors (HIs)	893
Assistant Health Inspectors (AHIs)	3,433

Table 2: Workforce available in healthcare [25]

Population per registered physician	1,410
Health Workforce	Per 10,000 population
Registered Physician	7.10
Doctors working under DGHS	1.59
Medical technologist	0.34
Community and domiciliary health workers under DGHS	2.17

Table 3: Overview of Bangladesh Population-Health Workforce ratio DHGS 2020 [25]

The Government of Bangladesh provides financial support to the public healthcare system, and subsidises care. Patients are also obliged to pay modest out-of-pocket payments, particularly for outpatient services. Bangladesh is struggling with one of the largest burdens of out-of-pocket expenses globally, with more than 63% of overall health costs incurred by households, resulting in financial fragility. Access to health services has been found to be unequal across geographic location, income level, gender, and there is a clear gap between urban and rural areas.[25] In June 2023, the Bangladesh National Parliament approved the budget for FY2023-24, with an increased allocation for the health sector. The health sector received 5% of the full budget, representing a 27.9% increase from the previous year.[26] Additionally, the Government initiated free healthcare services to those living below the poverty line to protect them from the financial hardship of illness. This policy is reflective of the Government of Bangladesh's ambition of universal health coverage for all by 2032.[27]

Private healthcare in Bangladesh spans profit-oriented enterprises, non-profit organizations (NGOs), and informal practitioners, such as village doctors and various unqualified providers. The private sector offers a diverse range of health facilities, from individual practitioners' offices to high-end tertiary-level hospitals adhering to international standards.[25] Bangladesh's private health business has expanded rapidly in recent years, improving public health. As of June 2020, Bangladesh had 5,577 registered private hospitals and clinics, and 10,727 private diagnostic centres. There are a total of 91,537 beds in registered private hospitals and clinics.[25] The majority of targeted commodities are manufactured and marketed by local pharmaceutical companies, and the majority of Bangladeshis obtain their prescriptions privately. Privately purchased drugs are the most common source of narcotics for disadvantaged people in rural and urban areas.

The Bangladesh National Newborn Health Programme and Integrated Management of Childhood Illness Programme (NNHP & IMCI) launched a significant initiative between 2020 and 2021 to improve access to medical oxygen.[28] This was aimed at enhancing care for newborns and children, led by the government, with support from UNICEF. As part of this initiative, the General Hospital in Tangail and the Medical College in Chattogram were upgraded, including the establishment of a liquid oxygen (LOX) facility and infrastructural improvements, and training was provided to doctors and nurses to effectively manage hypoxemia in children. Additionally, new oxygen indicators were added to the District Health Information System (DHIS2) for structured reporting. The initiative expanded to cover a total of 13 of 64 districts. Its success led the Government to acknowledge the need for national guidelines and training modules, and as a result, oxygen therapy has been included in the operational plan for the next sector programme, aiming to enhance the overall quality of paediatric care standards.

COVID-19

On March 08, 2020, the Institute of Epidemiology, Disease Control and Research (IEDCR) declared the first confirmed case of COVID-19 in Bangladesh.[29-31] Up to January 2024, there were 2,047,051 cases (Figure 7) and 29,481 deaths (Figure 8) officially reported in Bangladesh.[32] A nationwide lockdown was imposed on March 26, 2020 which extended until August 31, 2020.[33,34] Concurrently, the government introduced a TK.1.04 trillion (approximately USD 9.4 billion) package to mitigate COVID-19's financial impact, encompassing cash, food, subsidized food, tax relief, and policy support for firms of all sizes. Awareness-raising measures were implemented, and various mobile applications were launched during the lockdown.[35]

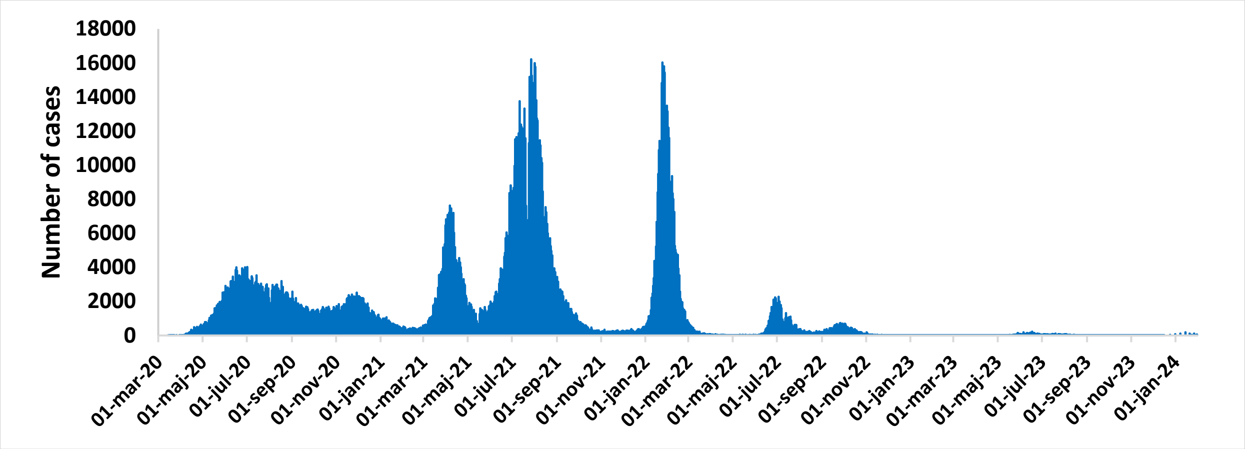

Figure 7: Trend in COVID-19 cases in Bangladesh (# of cases)

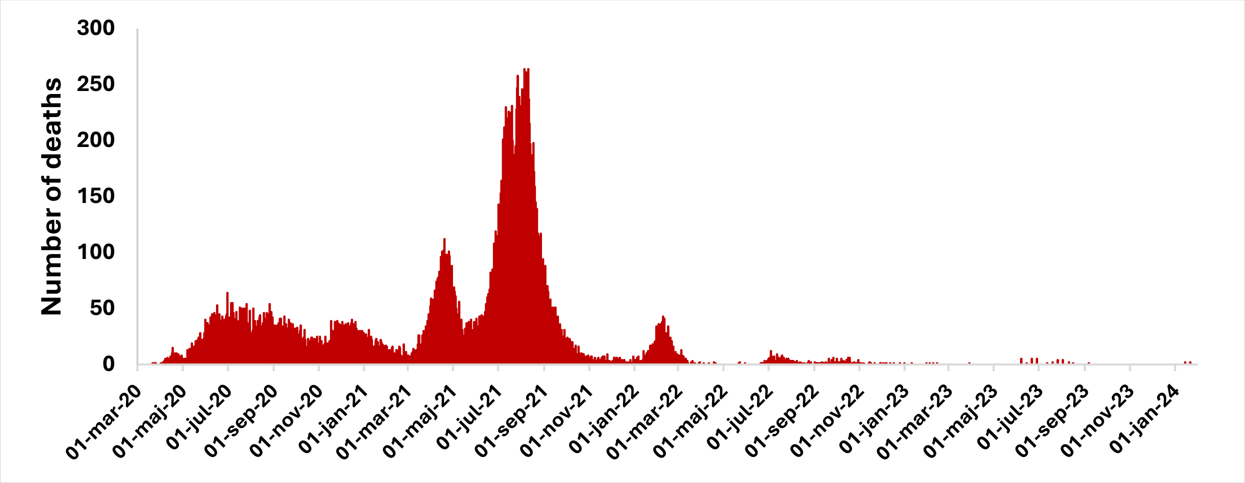

Figure 8: Trend in COVID-19 deaths in Bangladesh (# of deaths)

The Government of Bangladesh also developed a National Preparedness and Response Plan for COVID-19.[36] According to this plan, triage and screening facilities were made available for respiratory diseases in all hospitals, with special areas designated for identifying suspected cases. These centres then referred to the hospitals designated for managing COVID-19 patients, and suspected cases with comorbid conditions and critical cases were referred to specialised hospitals designated for advanced management of COVID-19 patients. In addition, all government hospitals had isolation units to quarantine patients, and special isolation wards for this purpose.

With the support of global partners such as the WHO, Serum Institute of India, and countries including Japan and Bulgaria, Bangladesh administered 160 million COVID-19 vaccine doses by January 2022 and fully immunized more than 70% of its population.[37] This was achieved through various channels, including purchases, donations, and the COVAX programme.[38,39] In February 2022, a large-scale vaccination initiative, supported by WHO and UNICEF, vaccinated 17 million people, significantly increasing the country's overall immunization rate. Dhaka, the capital, received the highest number of COVAX vaccine doses.[40] More than 90% of Bangladesh's population had received at least one COVID-19 vaccine dose (150,049,129 people) by 2024, with 131,182,263 having a second, 65,6732,743 a third, and 569,825 a fourth dose.[37,41]

Oxygen supply and clinical use landscape in Bangladesh

In Bangladesh, the regulation of oxygen is a collaborative effort involving various national and international entities. The Directorate General of Health Services (DGHS) under the Ministry of Health and Family Welfare (MOH&FW) leads this initiative, with significant contributions from the National Electro-Medical Equipment Maintenance Workshop and Training Centre (NEMEMW&TC) and the Central Medical Store Depot (CMSD). Other government bodies, such as the Public Works Department (PWD), City Corporations, and municipalities, also play crucial roles in maintaining the oxygen system. Development partners, such as UNICEF, WHO, USAID, The World Bank, ADB, the UK Foreign, Commonwealth & Development Office, and the Government of Canada have made significant contributions to this cause.

The availability and distribution of medical-grade oxygen is reliant on the nation's domestic production capabilities, which are based on the generation of liquid oxygen (LOX). This LOX is transported from manufacturing locations using tankers and is subsequently stored in Vacuum Insulated Evaporator (VIE) tanks at healthcare facilities. These VIE tanks enable a centralized supply of oxygen within hospitals and clinics. The country is equipped with 76 VIE tanks, dispersed across a range of healthcare institutions, from specialized and tertiary hospitals to district-level health centres. Additionally, numerous health facilities are equipped with onsite oxygen generating plants, with both Pressure Swing Adsorption (PSA) and Vacuum Swing Adsorption (VSA) technologies. Notably, the surge of the COVID-19 pandemic catalysed the establishment of over 100 oxygen generating plants, considerably enhancing capacity.

The regulatory framework governing the establishment and operation of medical oxygen plants mandates securing a license from the Director General of Drug Administration (DG-DA). A critical precondition for this licensing process is obtaining a gas purity certification from the Chemical Engineering Department of the Bangladesh University of Engineering and Technology (BUET). As of now, four LOX production and supply entities have acquired the necessary licensing, including Linde Bangladesh Ltd, which operates two facilities, Spectra Oxygen Ltd, Bangladesh Industrial Gas Limited, and Islam Oxygen Limited.

Furthermore, the importation and use of oxygen concentrators, cylinders, and manifold oxygen cylinder systems, while not directly licensed by the DG-DA, are subject to safety regulations requiring a fire safety certificate from the Department of Explosives within the Ministry of Energy and Mineral Resources. Prior to initiating the operational licensing application with the DG-DA, potential medical oxygen producers must first secure a gas purity certification from BUET's Chemical Engineering Department. The establishment and maintenance of Medical Gas and Vacuum Pipeline Systems in healthcare facilities are guided by the "PWD Standard Operating Procedure 2018 For Electro-Medical Works," as outlined by the Public Works Department. This procedural manual serves as a cornerstone for ensuring the safe and effective installation of medical gas systems. In response to the increased demand during the COVID-19 pandemic, an additional 14 local industrial LOX manufacturers were granted No Objection Certificates (NOCs) by local authorities, after obtaining the purity certificate from local authorities to supply oxygen to various facilities (Table 4).

During the COVID-19 pandemic, several companies also increased their LOX production capacity. For example, GPH Ispat’s Green factory in Chattogram, the largest oxygen plant in Bangladesh, produces 300 metric tons of medical oxygen daily - 10% in liquid form for medical use. Abul Khair Group’s Ispat unit in Chattogram produces 260 metric tons of oxygen daily, dedicating 30-40 tons for medical-grade oxygen. SPECTRA Oxygen Limited, which has been operational since 1999, supplies both liquid and gaseous medical gases. Linde Bangladesh, with two plants in Chittagong and Narayangonj, produces 90 metric tons of liquid oxygen daily. Meghna Group recently obtained a license and started producing 40 metric tons of liquid oxygen. KSRM produces 24,000 cubic meters of oxygen daily for cylinder use.

Sl.No.	Name and Address of the Manufacturers	NOC Issue date	Status Capacity
1	M / S Abul Khair Steel Melting Ltd. Sitalpur, Sitakunda, Chottogram	29-7-2020	Functional 260 Ton / Day
2	M / S D R Industries Ltd. 175, Muradpur, Modanpur, Bandar, Narayanganj	09-08-2020	Functional 19 Ton / Day
3	Union Oxygen Limited Lakhon Khola, Bander, Narayanganj	04-05-2021	Functional 9.26 Ton / Day
4	AK Oxygen Ltd. Barpa, Rupganj, Narayanganj	10-12-2020	Functional 1200m³ / Hour
5	Padma Oxygen Ltd, Thangrband, Modhyapara, Kaliakaoir, Gazipur	17-01-2021	Functional 8 Ton / Day
6	Sonargoan Steel Fabricate Ltd., Meghna Industrial Economic Zone, Tipordi, Sonargoan, Narayanganj	06-04-2021	Functional 56.8 Ton / Day
7	Arcade SS Oxygen Ltd., Ashulia, Savar, Dhaka	21-04-2021	Functional
8	Refat Oxygen Company (Pvt) Ltd., Kunda South, Keraniganj, Dhaka	27-04-2021	Functional 150m³ / Hour
9	Linde Bangladesh Ltd., Shipyard, Khulna	28-04-2021	Functional 1960m³ / Hour
10	Kabir Oxygen Limited, Johanabad, Bhatiary, Sitakunda, Chottogram	28-04-2021	Functional 1.6 MTon / Day
11	Spectra Oxygen Ltd., 64 Isali, Udhuli , Shebaloy , Manikganj, (Additionally during Covid crisis on rental, Basis at Shalauddin Oxygen Ltd , Kanchpur ,Narayanganj)	28-04-2021	Functional 400m³ / Hour
12	Diamond Steel Product Co. (Pvt) Ltd., Kanchpur, Sonargoan, Narayanganj	12-05-2021	Functional 1400m³ / Day
13	North Bengal Oxygen Plant, Majira Bypass, Dompukur, Shajahanpur, Bogura	03-08-2021	Functional 130m³ / Year
14	Associated Oxygen Limited, Khadampara, Madambibir Hat, Bhatiary, Sitakunda, Chottogram	03-08-2021	Functional 9800m³ / Day

Table 4: List of medical oxygen manufacturers received DGDA's NOC (as of January 2024)[42]

Until November 2021, the Central Medical Store Depot (CMSD) had procured or was in the process of procuring 50 VSA oxygen plants, and 52 PSA plants. The CMSD is responsible for supplying oxygen cylinders, currently covering 353 upazila-level hospitals, while the Ministry of Health and Family Welfare procured 3,136 oxygen concentrators and installed 76 liquid medical oxygen VIE tanks at different health facilities. Currently, 172 facilities have a central oxygen supply system, either by a manifold system, VIE tank, or oxygen plant.[42]

The National Electro-Medical Equipment Maintenance Workshop and Training Centre (NEMEW&TC), operating under the MOH&FW, oversees the maintenance of medical equipment, including oxygen systems in public health facilities. This responsibility extends to 596 facilities of varying tiers, from district hospitals and medical college hospitals to specialized and upazila health complexes, managed by a team of 62 staff members, including 14 biomedical experts. To ensure oxygen safety, the MOH&FW is considering the introduction of an accreditation system for hospital standards, which would encompass oxygen standards and safety measures. Given the limited resources in public facilities, it is crucial to improve the quality of care by effectively managing hypoxaemia in neonates, children, and adults. To address this issue, the Directorate General of Health Services (DGHS) introduced the "National Guidelines on Use of Oxygen Therapy for Management of Newborns and Paediatric Hypoxaemia" in 2020. This comprehensive guideline, developed by the National Neonatal Health Programme (NNHP) and Integrated Management of Childhood Illness (IMCI) programme under the DGHS, provides a framework for managing newborn and paediatric hypoxaemia.[43] However, there were no specific guidelines developed for adult patients, apart from the guidelines on oxygen therapy for COVID-19.

Sl . No.	Funding Support	Number of plants	Type
1	Government	3	PSA 500 litre / min
2	Government	40	20 VSA and 20 PSA
3	Global Fund	29	PSA
4	ADB	30	VSA
Total		102	

Table 5: Oxygen Generating Plants Procured by MOHFW (till November 2021) [42]

Introduction of pulse oximetry into IMCI settings

Introduction of IMCI in Bangladesh - a history of early adoption

The Government of Bangladesh has prioritized the reduction of respiratory infections, diarrhoea, and malnutrition among children under five years of age. This decision was influenced by the positive results from a multi-country evaluation of the IMCI guidelines recommended by the WHO in 1997.[44] In response to these findings, the Government established a national steering committee under the Deputy Programme Manager of the Control of Diarrhoeal Disease Programme.[45] The committee's mandate was to introduce IMCI services in Bangladesh. This led to the implementation of the Facility-Based Integrated Management of Childhood Illness (FB-IMCI) programme in 2001, marking a significant milestone in the country's healthcare journey.[44,46] The success of the FB-IMCI programme led to its expansion in 2004, resulting in substantial improvements in healthcare quality and utilization rates. By the end of 2014, the Government in partnership with various development partners, had expanded IMCI services to all 64 districts and over 420 upazila health complexes (Figure 9).[47,48] Recognizing the unique needs of its rural population, the Government introduced a Community-Based IMCI (CB-IMCI) strategy in 2003.[49] The NNHP & IMCI programme of DGHS, responsible for implementing IMCI services across the country, played a crucial role in this process.

In 2018, icddr,b, with technical assistance from the University of Edinburgh, initiated a research project to introduce pulse oximetry in routine IMCI settings. As part of this project, icddr,b organized a series of workshops with the NNHP & IMCI programme to sensitize them regarding the role of pulse oximetry in managing childhood illnesses. Consequently, the NNHP & IMCI programme agreed to lead the process of integrating pulse oximetry in IMCI settings of Bangladesh.[49] In the sensitization workshops, icddr,b presented the underlying problem related to pneumonia management in Bangladesh, pulse oximetry as a possible solution along with the experience of implementing pulse oximetry in other relevant contexts. This comprehensive approach convinced the NNHP & IMCI programme to take the leadership role in integrating pulse oximetry in IMCI settings of Bangladesh.

The central role of Government and research collaborations

icddr,b conducted a comprehensive desk review of IMCI-related documents and organized interviews with key informants to identify 15 stakeholder organizations at the national level and 16 at the district level. These stakeholders encompass government health programs, professional societies, UN agencies, and a range of non-government organizations (NGOs) both locally and internationally, as well as health service providers. Following this, icddr,b collaborated with the NNHP & IMCI team to organize a workshop aimed at mapping stakeholders based on a Power-Interest matrix. This exercise led to the identification of organizations with high power and high interest at both national and district levels, which were deemed crucial for the process of integrating pulse oximetry in IMCI settings. At the national level, high power and high interest were held by DGHS, WHO, Save The Children, UNICEF, and

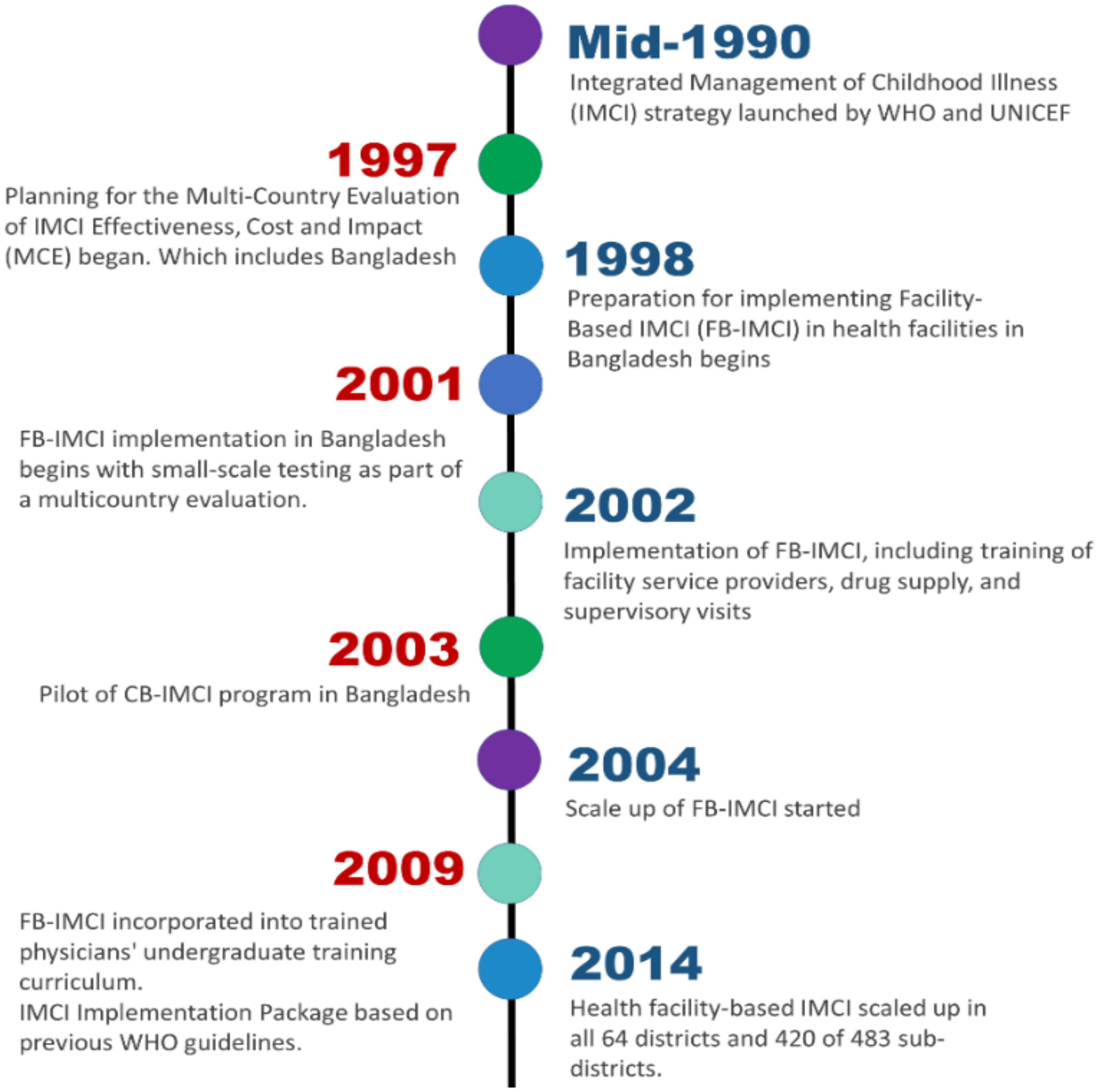

Figure 9: Journey of IMCI in Bangladesh

icddr,b. At the district level, the high power-high interest group comprised of the Civil Surgeon (district level health manager), Upazila Health and Family Planning Officer (sub-district level health manager), doctors, nurses, Sab assistant community medical officers (Paramedics), and the Resident Medical Officer (RMO) – Figure 10. It is notable that in sub-districts, where responsibility for enacting on policies takes place, the power firmly lies with Government structures and the presence of international and national NGOs are virtually non-existent. While at the National level, the prominent role of icddr,b signifies the importance with which research and evidence-informed decision making has held to date in IMCI programming.

In addition to the mapping, a thirteen-member working group was established, chaired by the Programme Manager of the NNHP & IMCI programme. This group comprised members from DGHS, UN organizations, and development partners. The working group decided to update the overall national IMCI guideline and IMCI implementation package based on the existing global guidelines, rather than solely focusing on the introduction of pulse oximeters. They also recommended conducting implementation research to test the feasibility of introducing pulse oximetry in routine IMCI settings. This decision emphasizes the continued value placed on evidence and evidence-generation and reflects long-standing collaborations.

Upon reviewing the WHO guidelines from 2009 and 2014, the group decided to adopt and update the criteria classification of pneumonia in children. After careful consideration of the contextual relevance, rationale, and operational feasibility, the group incorporated these changes into the national IMCI guidelines. This led to an extensive update of the National IMCI Implementation Package, which includes the IMCI chart booklet, IMCI service registers, referral forms, training manuals, reporting forms, monitoring checklist, drug list, job aids, equipment, logistic and medicine list, trainer's guide, student handbook, and manager toolkit. A total of 24 documents were updated, with pulse oximetry included in the updated IMCI implementation package. The NNHP & IMCI programme also organised orientation sessions with IMCI master trainers on the updated IMCI chart booklet and training manuals. The master trainers carefully reviewed the IMCI chart booklet and training manuals and provided feedback. Subsequently, feedback was received and incorporated from the members of National Newborn Technical Working Committee for Newborn Health and the National IMCI Technical Working Committee, the highest-level technical committees regarding newborn and child health in Bangladesh (Figure 11).

Centring evidence in policy decision-making

In order to determine whether it would be possible to incorporate pulse oximetry into IMCI, the NNHP & IMCI programme, in collaboration with icddr,b, conducted implementation research.[50] Kushtia district was selected for the "District Implementation Model" initiative to introduce pulse oximetry in routine IMCI settings because of its significant patient turnover and long-standing IMCI services. Eleven health facilities including district hospital, upazila health complexes and union level health facilities were chosen as the study sites. The IMCI service providers in these health facilities received training on the updated IMCI Implementation Package prior to the start of the implementation evaluation. Pulse oximeters were also introduced in the IMCI corners of these selected health facilities. This study assessed WHO implementation outcome variables such as adoption, feasibility, fidelity, appropriateness, acceptability and sustainability based on a pre-set benchmark for each indicator. The study revealed that the IMCI service providers almost universally used the pulse oximeter on all eligible children. The IMCI service providers were successful in conducting pulse oximetry within a short period of implementation, with good accuracy and minimum challenges. Pulse oximetry was also well accepted by the caregivers.

The success was largely attributed to the early and strategic involvement of stakeholders, including IMCI service providers. They were sensitized and actively participated in all stages, from the design and development to the implementation of pulse oximetry in routine IMCI. Continuous engagement via training, support supervision, and performance appraisal workshops resulted in a sense of ownership and acquisition of necessary technical skills among the IMCI service providers, which have likely contributed to the positive findings. These positive findings in turn convinced the policy makers of Bangladesh to scale-up pulse oximetry to the whole country, using lessons learnt. The process of scale up included training of the IMCI service providers on updated IMCI implementation package, procurement of pulse oximeters using government funding and integration of pulse oximetry related indicators in the routine DHIS2. One of the key informants stated:

> *"The pulse oximeter's integration has been successful, enabling accurate detection of severe pneumonia in babies with low oxygen levels. Despite the challenges faced by our staff, this tool has improved our diagnostic capabilities and patient care. While data accuracy is not yet perfect, we anticipate gradual improvements."*

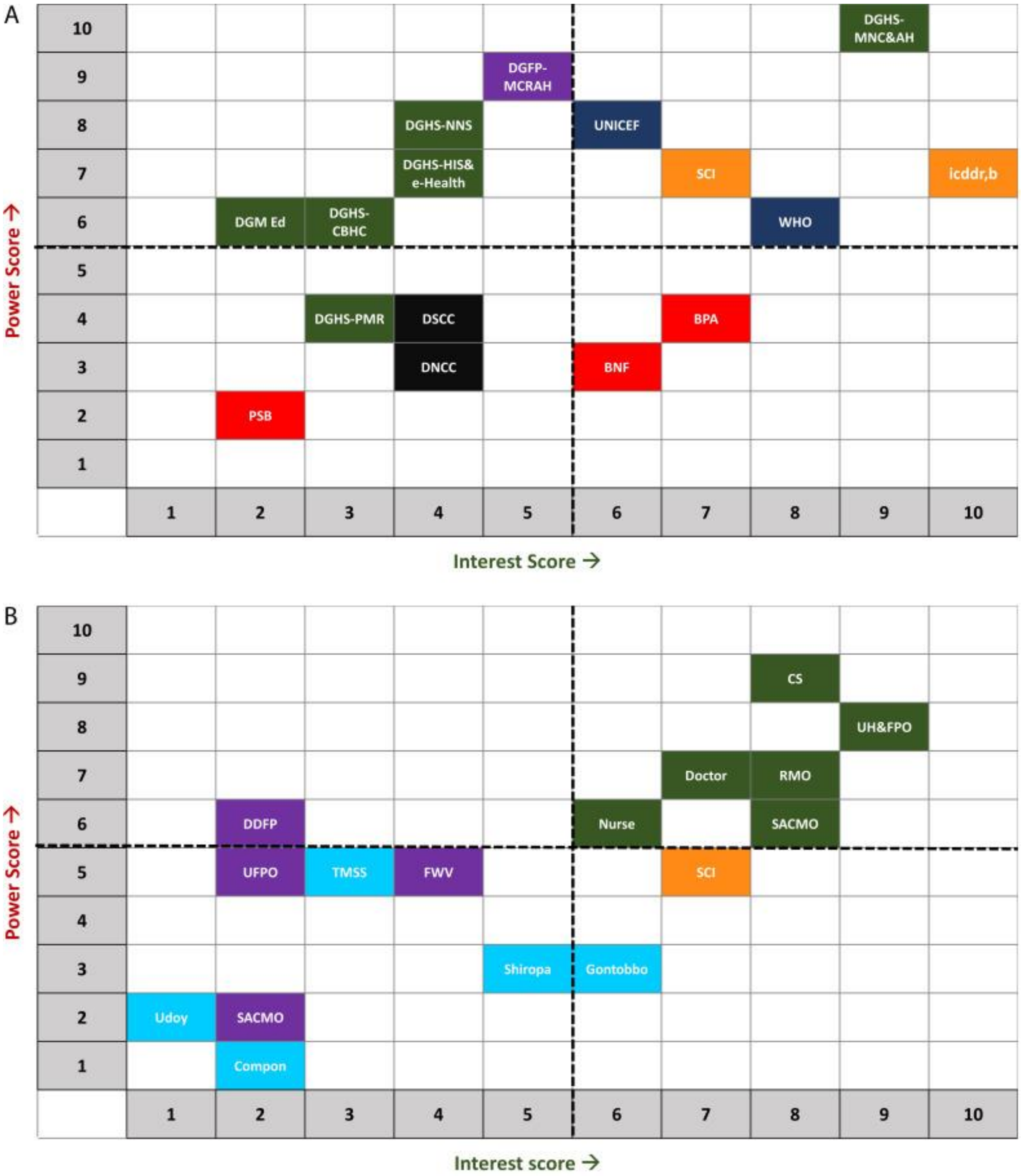

BNF-Bangladesh Neonatal Federation, **BPA**-Bangladesh Pediatric Association, **PSB**- Perinatal Society of Bangladesh, **DGFP-MCRAH**- Directorate General of Family Planning- Maternal, Child, Reproductive and Adolescent Health, **DGHS-CBHC**- Directorate General of Health Services-Community Based Health Care- Operational Plan, **DGHS-HIS& e-Health**- Directorate General of Health Services- Health Information System and e-Health- Operational Plan, **DGHS-MNC&AH**- Directorate General of Health Services-Maternal Newborn Child and Adolescent Health, **DGHS-NNS**- Directorate General of Health Services-National Nutrition Services- Operational Plan, **DGHS-PMR**- Directorate General of Health Services-Planning, Monitoring and Research-Operational Plan, **SCI**- Save the Children, **UNICEF**- United Nations International Children's Emergency Fund, **WHO**- World Health Organization, **DNCC**- Dhaka North City Corporation, **DSCC**-Dhaka South City Corporation, **DGMEd**- Directorate General of Medical Education, **icddr,b**- International Centre for Diarrhoeal Disease Research
CS-Civil Surgeon, **DDFP**-Deputy Director Family Planning, **FWV**-Family Welfare Visitor, **RMO**-Residential Medical Officer, **SACMO**- Assistant Community Medical Officer, **TMSS**- Thengamara Mohila Sobuj Sangha, **UFPO**- Upazila Family Planning Officer, **UH&FPO**- Upazila Health and Family Planning Officer.

Directorate general of health services
Directorate general of family planning
Professional body
City corporation
UN organization
National/ International NGO
Local NGO

Figure 10: Power-Interest mapping of national (A) and district level (B) stakeholders[50]

The importance of a comprehensive training strategy

Since December 2019, NNHP and IMCI program in Bangladesh have been working on a comprehensive training initiative. This initiative has successfully trained 4,872 service providers, including doctors, nurses, and paramedics, on the updated IMCI Implementation Package, with a particular emphasis on the use of pulse oximetry. The program operates across various training venues, including Dhaka and several medical colleges outside the capital. Each venue hosts a pool of 5-6 experienced individuals who further train healthcare providers at designated facilities. The training duration has been optimized over time, with doctors now receiving 5 days of training, nurses, SACMOs, and paramedics for 6 days, and basic health workers for 2 days. One of the key informants mentioned that,

> *"This isn't a one-time event, but a continuous cycle of initial training, refresher courses, and mentoring. As knowledge can fade, regular refreshers are necessary. This ongoing learning not only maintains up-to-date information but also motivates our service providers, addressing a key deficiency."*

The programme ensures continuity of learning through annual training and refresher courses, catering to both new recruits and existing staff. Funding for these training programs is sourced from development partners and the government budget, with government-funded sessions typically held from February to June. International organizations such as UNICEF and WHO support the IMCI training programs. Annual performance appraisal meetings engage data-involved personnel, including doctors, nurses, SACMOs, basic health workers, and statisticians from Upazila Health Complexes. These individuals receive training on IMCI datasets and updates from the Management Information System.

Procurement of pulse oximeters with government funding

The NNHP & IMCI programme decided to do phase-wise scale up of pulse oximeters, where in the first phase, pulse oximeters will be procured for district hospitals and upazila health complexes. In the second phase pulse oximeters will be procured for union level health facilities. As part of the decision for national scale-up, pulse oximeters are procured through Central Medical Store Depot (CMSD) using government funds from the programme budget, with no specific model prioritized. Instead, purchases are made based on specifications that fit within the budget. One of the respondents mentioned that,

> *"Our purchases are made according to the Public Procurement Act, with CMSD as our procurement agency. We are required to buy items from suppliers who fit within our allocated budget. We didn't prefer a specific pulse oximeter model, but we procured it based on the implementation research conducted in Kushtia. However, due to budget constraints, we procured the minimum which our government could afford."*

Over the course of two fiscal years, 2020-21 and 2021-22, the programme procured two thousand handheld pulse oximeters with child probes (model: Yongkang YK-820A). Despite challenges such as the lack of a fixed budget and the need to ensure the procurement of high-quality pulse oximeters with child probes, the NNHP & IMCI programme has successfully distributed these pulse oximeters to health facilities at both district hospitals and upazila health complexes. The NNHP & IMCI programme also kept provision of procurement of pulse oximeters in the next health sector programme (2024 to 2029) with plans to introduce pulse oximetry in the union level health facilities in the second phase.

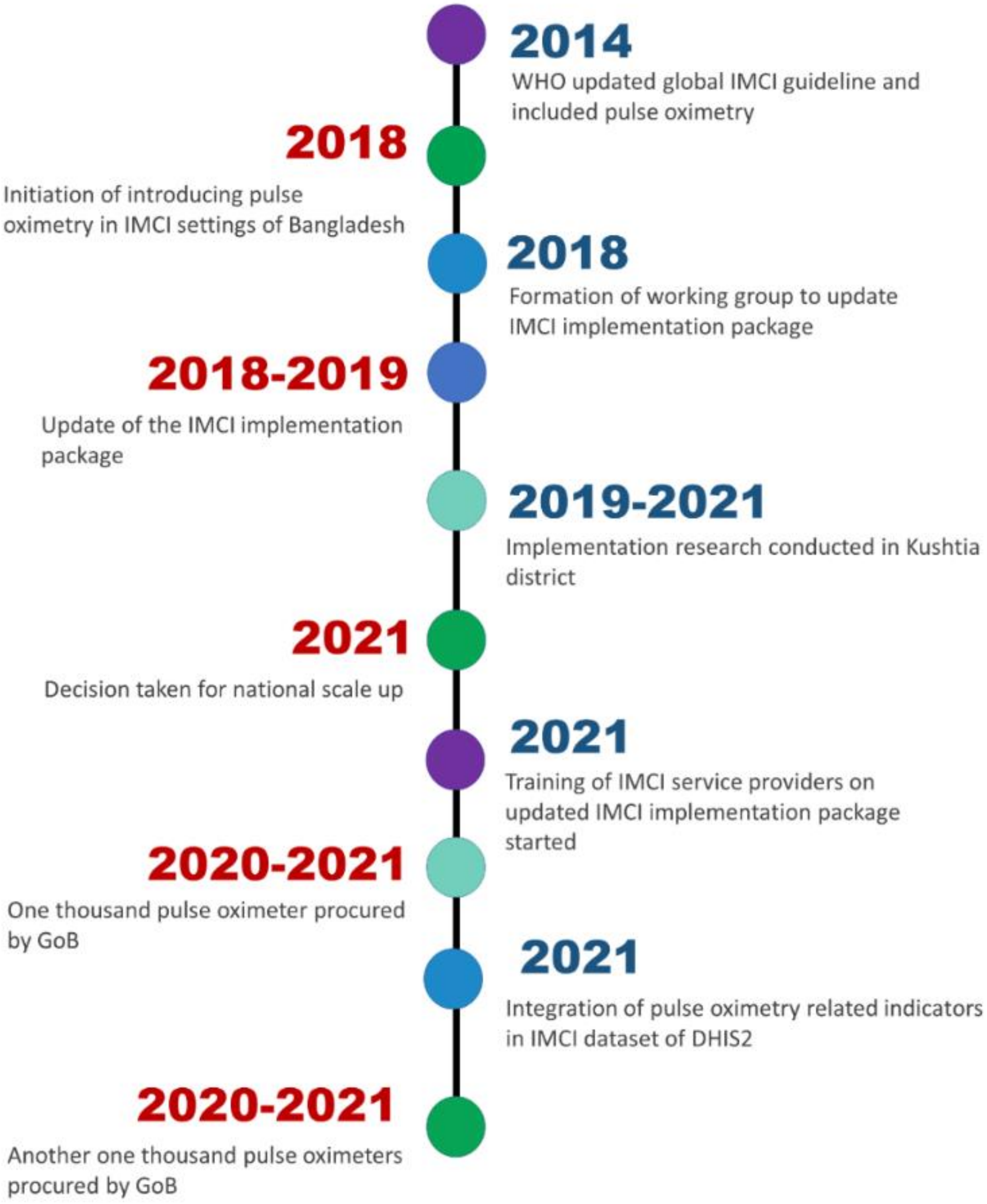

Figure 11: Journey of introducing pulse oximetry in IMCI services of Bangladesh

Integration of indicators in DHIS2

The NNHP & IMCI programme organised meetings with the Management Information System (MIS) of DGHS to update the DHIS2 IMCI dataset. This led to an agreement between NNHP & IMCI programme and MIS to introduce a New IMCI dataset in DHIS2 including indicators for hypoxaemia detection. Currently, the NNHP & IMCI programme is arranging regular trainings and workshops to improve the quality of the new IMCI dataset. One deputy programme manager of the NNHP & IMCI programme is assigned to ensure the quality of new IMCI dataset. One of the key informants mentioned that,

> *"In January 2021, the IMCI dataset was introduced in DHIS2 throughout Bangladesh. Initially, there were some mistakes with data being uploaded to the old dataset. However, series of orientation arranged on the new system for health managers and statisticians of the health facilities responsible for data entry, the transition has been successful. Now, all data, including pulse oximeter-related information, is being received via the new IMCI dataset in DHIS2."*

The impact of COVID-19

The COVID-19 pandemic resulted in cessation of the implementation activities due to a government-imposed lockdown in March 2020. Following a five-month intermission, the NNHP & IMCI programme demonstrated initiative by resuming activities and ensuring the timely completion of the implementation. Health managers were directed to reactivate all events, adhering to a revised plan that incorporated necessary precautions to mitigate the spread of the virus. However, the COVID-19 pandemic resulted in increased awareness regarding the impact of low oxygen saturation in blood and usefulness of pulse oximetry among the service providers and caregivers.

Key messages

- It is more likely for the policy makers to be convinced on taking any decision if the underlying problem, possible solution along with the experience of that solution in other relevant contexts is presented before them.
- The overall process of integrating pulse oximetry in IMCI settings were led by NNHP & IMCI programme with catalytic support from icddr,b and other development partners. The country ownership and government leadership facilitated the process of introduction and scale up of pulse oximetry in Bangladesh
- The extensive stakeholder mapping exercise was crucial in identifying the key stakeholders regarding child health services in Bangladesh. To successfully introduce pulse oximetry in Bangladesh, it was important to involve and engage key stakeholders at every stage of the process. This ensured that all relevant parties were informed and invested in the initiative, which in turn increased the likelihood of success
- The overall update the national IMCI guideline based on the existing evidence and not only focusing on inclusion of pulse oximetry created the momentum for successful integration of pulse oximetry in IMCI settings
- Setting a benchmark prior to conducting the implementation research created a sense of accountability among the service provide

Additional methods information:

This case study was conducted based on the information received from key informants' interviews and the information collected from the documents mentioned by the key informants. We first purposively approached the Hospital Services Management programme, who are responsible for ensuring oxygen security in the hospitals of Bangladesh, and the NNHP & IMCI programme of DGHS, who are responsible for implementing IMCI services across the country. We collected information of the personnels who were involved in the process of pulse oximetry integration in IMCI settings of Bangladesh from the NNHP & IMCI programme. A total of 14 participants were identified and all of them were interviewed.

Programme Manager of Hospital Service Management (HSM)
Ex-Programme Manager of NNHP & IMCI
Programme Manager of NNHP & IMCI
Deputy Programme Manager, Monitoring and Data Quality, NNHP & IMCI
Deputy Programme Manager, Training and Child Injury, NNHP & IMCI
Deputy Programme Manager, Newborn Health, NNHP & IMCI
Deputy Programme Manager, Monitoring & Data Quality
Deputy Programme Manager, Coordination & Logistics
Deputy Programme Manager, Admin & Finance
Deputy Programme Manager, Newborn Health
Former Advisor, Pneumonia Centinel Commitment (PCC) Project, Save the Children
Advisor, Save the Children
Health Specialist, World Health Organization
NPO, MNCAH, WHO Bangladesh

Table 6: Key informant interview participants

References

1. Carai S, Kuttumuratova A, Boderscova L, et al. Review of Integrated Management of Childhood Illness (IMCI) in 16 countries in Central Asia and Europe: implications for primary healthcare in the era of universal health coverage. *Arch Dis Child* 2019; **104**(12): 1143-9.
2. Lazzerini M, Sonego M, Pellegrin MC. Hypoxaemia as a mortality risk factor in acute lower respiratory infections in children in low and middle-income countries: systematic review and meta-analysis. *PloS one* 2015; **10**(9): e0136166.
3. Rahman AE, Hossain AT, Nair H, et al. Prevalence of hypoxaemia in children with pneumonia in low-income and middle-income countries: a systematic review and meta-analysis. *The Lancet Global Health* 2022; **10**(3): e348-e59.
4. Rahman AE, Mhajabin S, Dockrell D, Nair H, El Arifeen S, Campbell H. Managing pneumonia through facility-based integrated management of childhood management (IMCI) services: an analysis of the service availability and readiness among public health facilities in Bangladesh. *BMC health services research* 2021; **21**: 1-13.
5. Floyd J, Wu L, Hay Burgess D, Izadnegahdar R, Mukanga D, Ghani AC. Evaluating the impact of pulse oximetry on childhood pneumonia mortality in resource-poor settings. *Nature* 2015; **528**(7580): S53-S9.
6. World Health Organization. Integrated management of childhood illness - Chart booklet. 2014. https://www.who.int/publications/m/item/integrated-management-of-childhood-illness---chart-booklet-(march-2014)2023).
7. World Health Organization. Revised WHO classification and treatment of childhood pneumonia at health facilities. 2014. https://www.who.int/publications/i/item/97892415078132023).
8. McCollum ED, King C, Deula R, et al. Pulse oximetry for children with pneumonia treated as outpatients in rural Malawi. *Bulletin of the World Health Organization* 2016; **94**(12): 893.
9. World Health Organisation. Verbal autopsy standards: ascertaining and attributing causes of death tool. 2024. https://www.who.int/standards/classifications/other-classifications/verbal-autopsy-standards-ascertaining-and-attributing-causes-of-death-tool.
10. Jetté N, Quan H, Hemmelgarn B, et al. The development, evolution, and modifications of ICD-10: challenges to the international comparability of morbidity data. *Medical care* 2010; **48**(12): 1105-10.
11. Bangladesh Bureau of Statistics. Bangladesh Sample Vital Statistics 2021. 2021. https://drive.google.com/file/d/1HfirfErmcD6XEVDbgH0ubKe_TRyprrHM/view2024.
12. Ali A, Mahin Al N, Md Mahabubur R, et al. Adult mortality trends in Matlab, Bangladesh: an analysis of cause-specific risks. *BMJ Open* 2023; **13**(9): e065146.
13. Bangladesh Bureau of Statistics. Population & Housing Census 2022 Preliminary Report. 2022. https://sid.portal.gov.bd/sites/default/files/files/sid.portal.gov.bd/publications/01ad1ffe_cfef_4811_af97_594b6c64d7c3/PHC_Preliminary_Report_(English)_August_2022.pdf2024.
14. National Institute of Population Research and Training and ICF. Bangladesh Demographic and Health Survey 2022. 2023. https://dhsprogram.com/pubs/pdf/PR148/PR148.pdf2024.
15. Bangladesh Bureau of Statistics. Bangladesh Sample Vital Statistics 2020 Key Findings. 2023. https://bbs.portal.gov.bd/sites/default/files/files/bbs.portal.gov.bd/page/b343a8b4_956b_45ca_872f_4cf9b2f1a6e0/2023-06-14-11-32-83dd2f50d6a81a25ae1233e77e85b7f6.pdf
16. Bank TW. Bangladesh. 2022. https://data.worldbank.org/country/BD.
17. Health Economics Unit. Bangladesh National Health Accounts 1997-2020, 1997-2020.
18. The World Bank. World Bank Country and Lending Groups. https://datahelpdesk.worldbank.org/knowledgebase/articles/906519-world-bank-country-and-lending-groups.

19. Hossain AT, Siddique AB, Jabeen S, et al. Maternal mortality in Bangladesh: Who, when, why, and where? A national survey-based analysis. *Journal of Global Health* 2023; **13**.
20. Haider MM, Siddique AB, Jabeen S, et al. Levels, trends, causes, place and time of, care-seeking for, and barriers in preventing indirect maternal deaths in Bangladesh: An analysis of national-level household surveys. *Journal of Global Health* 2023; **13**.
21. McCollum ED, Ahmed S, Roy AD, et al. Risk and accuracy of outpatient-identified hypoxaemia for death among suspected child pneumonia cases in rural Bangladesh: a multifacility prospective cohort study. *The Lancet Respiratory Medicine* 2023.
22. Rahman AE, Hossain AT, Chisti MJ, et al. Hypoxaemia prevalence and its adverse clinical outcomes among children hospitalised with WHO-defined severe pneumonia in Bangladesh. *J Glob Health* 2021; **11**: 04053.
23. Shi T, McAllister DA, O'Brien KL, et al. Global, regional, and national disease burden estimates of acute lower respiratory infections due to respiratory syncytial virus in young children in 2015: a systematic review and modelling study. *The Lancet* 2017; **390**(10098): 946-58.
24. National Institute of Population, Research, Training, Niport, Ministry of Health Family Welfare, ICF. Bangladesh Demographic and Health Survey 2017-18. Dhaka, Bangladesh: NIPORT/ICF, 2020.
25. Directorate General of Health Services-Ministry of Health and Family Welfare- Government of Bangladesh. Bangladesh Health Bulletin 2020. 2022. https://dghs.portal.gov.bd/sites/default/files/files/dghs.portal.gov.bd/page/8983ee81_3668_4bc3_887e_c99645bbfce4/2022-09-20-12-31-58c5a0b12e3aad087eaa26c3ce0f1a7b.pdf.
26. Centre for Policy Dialogue. National Budget 2023-24: Summary, Health. 2023. https://cpd.org.bd/resources/2023/08/Health-budget-2023-24.pdf.
27. Mostari S, Mohona NP. Can Bangladesh achieve universal health coverage? 2023. https://a2i.gov.bd/can-bangladesh-achieve-universal-health-coverage/#:~:text=With%20only%202.5%25%20of%20the,coverage%20(UHC)%20by%202032.
28. UNICEF. Improving newborn and pediatric quality of care by strengthening access to safe use of oxygen, pulse oximetry, and infection prevention and control measures. 2021. https://www.unicef.org/media/122526/file/UNICEF_Bangladesh_case%20study_Scaling_oxygen_access_for_newborns_and_children_2021.pdf.
29. Standard TB. First coronavirus cases detected in Bangladesh. *The Business Standard (2020, March 8)* 2020.
30. Islam MT, Talukder AK, Siddiqui MN, Islam T. Tackling the COVID-19 pandemic: The Bangladesh perspective. *J Public Health Res* 2020; **9**(4): 1794.
31. Saha P, Gulshan J. Systematic Assessment of COVID-19 Pandemic in Bangladesh: Effectiveness of Preparedness in the First Wave. *Front Public Health* 2021; **9**: 628931.
32. Worldometer. COVID-19 CORONAVIRUS PANDEMIC. 2020. https://www.worldometers.info/coronavirus/ (accessed 31 March 2020).
33. Islam MS, Tusher TR, Roy S, Rahman M. Impacts of nationwide lockdown due to COVID-19 outbreak on air quality in Bangladesh: a spatiotemporal analysis. *Air Quality, Atmosphere & Health* 2021; **14**: 351-63.
34. Mina FB, Billah M, Rahman MS, et al. COVID-19: transmission, diagnosis, policy intervention, and potential broader perspective on the rapidly evolving situation in Bangladesh. *J Adv Biotechnol Exp Ther* 2020; **3**(4): 18-29.
35. Akanda AAM, Ahmed R. How successful Bangladesh is in controlling the coronavirus pandemic? *Bulletin of the National Research Centre* 2020; **44**: 1-8.
36. Azad AK. National Preparedness and Response Plan for COVID-19 Bangladesh: Directorate General of Health Services, Health Service Division, Ministry of Health and Family Welfare, Bangladesh, 2020.

37. Nazmunnahar, Ahamed B, Haque MA, et al. COVID-19 vaccination success in Bangladesh: Key strategies were prompt response, early drives for vaccines, and effective awareness campaigns. *Health Sci Rep* 2023; **6**(5): e1281.
38. Sarker, Mohammad N, Ghosh, Sourav. A Study on the Key Elements of Bangladesh's Vaccine Diplomacy During COVID-19. *Journal of Asian and African Studies* 2023: 00219096231179659.
39. UNICEF. UNICEF: 190 million COVID-19 vaccines delivered under COVAX. 2022. https://www.unicef.org/bangladesh/en/press-releases/unicef-190-million-covid-19-vaccines-delivered-under-covax.
40. UNICEF. Bangladesh's COVID-19 vaccination rate has soared in a year. 2022. https://www.unicef.org/stories/bangladesh-covid-19-vaccination-rate-has-soared.
41. DGHS. COVID-19 Vaccination Dashboard for Bangladesh. http://103.247.238.92/webportal/pages/covid19-vaccination-update.php.
42. UNICEF. Bangladesh National Oxygen Landscape Report.
43. Kabir ANME, Afroze S. National Guidelines on Use of Oxygen Therapy for Management of Newborn and Paediatric Hypoxemia; 2020.
44. El Arifeen S, Blum LS, Hoque DM, et al. Integrated Management of Childhood Illness (IMCI) in Bangladesh: early findings from a cluster-randomised study. *Lancet* 2004; **364**(9445): 1595-602.
45. Binagwaho A, Udoh K, Ntawukuriryayo T, et al. Exemplars in Under-5 Mortality: Bangladesh Case Study. 2019.
46. Lawn J, Kerber K. Opportunities for Africa's Newborns: Practical data, policy and programmatic support for newborn care in Africa. *Partnership for Maternal, Newborn and Child Health, Cape Town* 2006; **32**.
47. Bryce J, Victora CG, Habicht J-P, Black RE, Scherpbier RW. Programmatic pathways to child survival: results of a multi-country evaluation of Integrated Management of Childhood Illness. *Health policy and planning* 2005; **20**(suppl_1): i5-i17.
48. Arifeen SE, Hoque DE, Akter T, et al. Effect of the Integrated Management of Childhood Illness strategy on childhood mortality and nutrition in a rural area in Bangladesh: a cluster randomised trial. *The Lancet* 2009; **374**(9687): 393-403.
49. Ahsan KZ, Streatfield PK, Ijdi R-E-, Escudero GM, Khan AW, Reza M. Fifteen years of sector-wide approach (SWAp) in Bangladesh health sector: an assessment of progress. *Health Policy and Planning* 2016; **31**(5): 612-23.
50. Rahman AE, Jabeen S, Fernandes G, et al. Introducing pulse oximetry in routine IMCI services in Bangladesh: a context-driven approach to influence policy and programme through stakeholder engagement. *Journal of Global Health* 2022; **12**.

THE LANCET Global Health
COMMISSION ON MEDICAL OXYGEN SECURITY

Country Case Study: India

Establishing a National Medical Oxygen Grid in India

Varun Manhas[1] and Ramanan Laxminarayan[1]

1. One Health Trust, India

Case study focus

The COVID-19 pandemic highlighted and exacerbated the deficiencies of the medical oxygen system in India, presenting a unique and urgent opportunity to solve a critical problem of our time. The pandemic had a catastrophic effect not just because of the rates of contagion and the virulence of the disease but also because of the drastic scarcity in life-saving resources such as medical oxygen. Pre-COVID-19, hospitals in India relied heavily on third-party vendors to meet their oxygen demand as they could not manufacture it locally. The increase in oxygen demand during the pandemic led to black-marketing and hoarding,[1] and desperate requests were raised on social media for medical oxygen. At times, hospitals also sought judicial orders against the supplying agencies and the government to replenish their oxygen supplies.[2]

Historically medical oxygen demand, supply, and consumption has not been monitored, making it hard to plan both for routine care provision, but also surge capacity needs. While some hospitals developed an “oxygen policy” during COVID-19 to: (i) monitor the current and future demand and consumption; (ii) ensure adequate supply with rational use, and maintenance of equipment; and (iii) conducting regular audits.[3] To address the supply chain challenges and mitigate risks associated with disrupted or insufficient supply, one of the most critical aspects is to have a supply chain data system that can support informed decision-making and greater operational efficiencies. This case study outlines the concept of a national medical oxygen grid (NMOG), a state-of-the-art IT platform, which aims to ensure the supply of high-quality medical oxygen in routine and surge scenarios, and smooth out fluctuations in demand.

Country Context

Demography, economy, and epidemiology

Along with overtaking China as the world's most populous country in 2023, India is undergoing transitions on multiple fronts - economic, demographic, and epidemiological. This vast and diverse country is being presented with various challenges and opportunities as it seeks to transform its health sector. Since transitioning from a low-income to a lower-middle-income country in 2009, India has seen improvements in life-expectancy and increased health expenditure (Table 1).

Development Indicators	Historical Value (Year)	Current Value (Year)
Total population	1,291,132,063 (2013)	1,428,627,663 (2023)
Total under-five population	125,709,000 (2013)	113,049,137.5 (2023)
Under-five mortality	55 deaths per 1,000 live births (2011)	30.6 deaths per 1,000 live births (2021)
Life expectancy (m:f)	67.0 : 70.1 years (2013)	70.5 : 73.6 years (2023)
Gross Domestic Product	$US 1.83 trillion (2012)	$US 3.39 trillion (2022)
Healthcare expenditure	$US 44.90 per capita (2010)	$US 56.63 per capita (2020)
Income status	Low income (2009)	Lower middle income (2023)

Table 1: Demography and economy of India[4,5]

In India, non-communicable diseases account for 56% of all deaths and were the leading cause of death between 2017 and 2019 (Figure 1). Communicable, maternal, perinatal, and nutritional conditions constitute another 22% of deaths during the same period. Overall, cardiovascular disease (29%) was the leading cause of death. Respiratory diseases also accounted for a substantial proportion of deaths in all the regions, with the highest burden reported in the Central region (10%) followed by the Northern region (9%) and the lowest burden in the North-Eastern region (6%). Age and gender distributions of deaths in India due to respiratory infections and respiratory diseases between 2017 and 2019 can be found in Table 2, and demonstrate a dual burden of infections in young children and the elderly.[6] With a current Sustainable Development Goal (SDG) index score of 63.45, the country is working towards achieving all its SDGs, with action on poverty and improving health, but India still ranks 112th globally on this metric and therefore needs to accelerate action.[7]

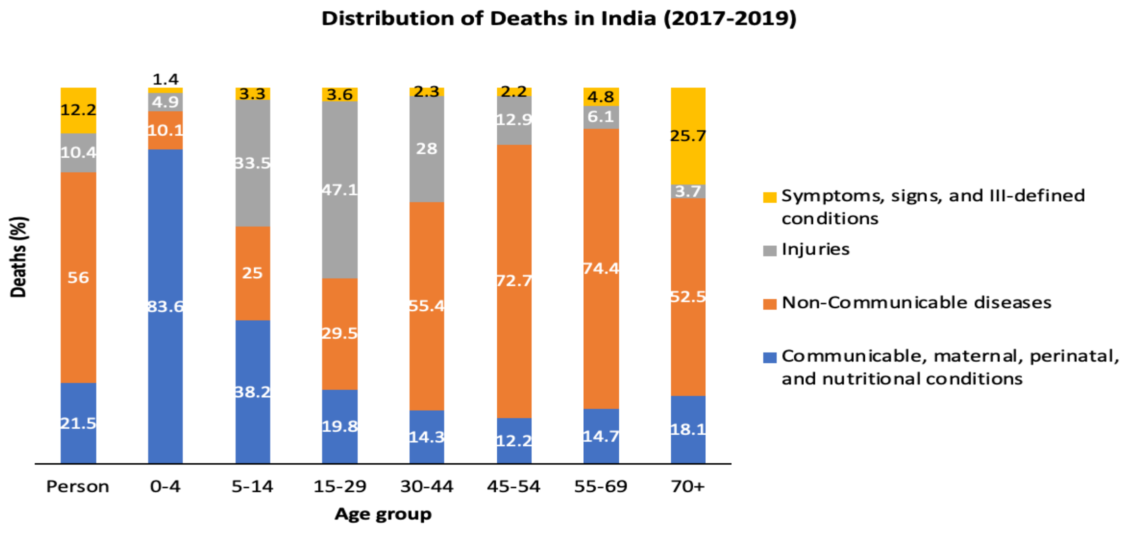

Figure 1: Distribution of deaths in India (2017-2019)[6]

		Percentage of deaths							
	**Cause of death**	**Overall**	**0-4**	**5-14**	**15-29**	**30-44**	**45-54**	**55-69**	**70+**
Overall	Respiratory infections	3.6	17.4	6.3	1.0	0.9	1.2	1.9	3.7
	Respiratory diseases	7.3	0.3	0.9	1.4	2.2	4.3	8.9	10.5
Male	Respiratory infections	3.2	16.8	6.0	0.6	0.7	1.0	1.7	3.7
	Respiratory diseases	7.0	0.3	0.8	1.1	1.8	3.7	8.4	11.0
Female	Respiratory infections	4.2	18.1	6.7	1.6	1.4	1.7	2.3	3.8
	Respiratory diseases	7.7	0.2	0.9	1.9	3.1	5.6	9.6	9.9

Table 2: Distribution of deaths in India due to respiratory infections and diseases between 2017 and 2019[6]

Health system

In India, services in the public health system include both curative and preventive services. Health is a State subject, and therefore each State operates its own health facilities, organized under the Department of Health and Family Welfare (DoHFW), headed by a Minister of Health and Family Welfare and a Minister for Medical Education. The Health Secretariat within the DoHFW is administered by Principal Secretaries and Commissioners of Health and Family Welfare Services and Medical Education. These are officers of the Indian Administrative Services (IAS) and are supported by other administrative officials. Moreover, the activities of the National Health Mission (NHM) at the state-level are carried out through State Health Societies (SHS).

The Central Government, through the Ministry of Health and Family Welfare (MoHFW), oversees policymaking, planning, guiding, assisting, evaluating, and coordinating the work with state health authorities. To achieve nationally or internationally desirable health goals, the Central Government also finances national health schemes such as Ayushman Bharat, PM-JAY, Free Drugs and Diagnostics Service Initiative. This is to help ensure adequate coverage of health services and consistency in performance across different states. Currently, the MoHFW has two independent departments: (i) the Department of Health and Family Welfare (DoHFW); and (ii) the Department of Health Research. The Ayurveda, Yoga, Naturopathy, Unani, Siddha, and Homeopathy (AYUSH) services, which was earlier a department under the MoHFW, has now been established as a separate ministry (Figure 2). The departments are staffed by IAS officials, technical advisors, and administrative staff, supported by a network of public-funded autonomous research and training institutions, and technical advisory bodies.

Apart from health facilities operated and funded by the State and National Ministries of Health, public sector employers such as the Ministry of Defense and the Ministry of Railways, also provide health services through institutions directly owned, financed, and managed by them. The All India Institute of Medical Sciences (AIIMS), India's main tertiary-level health facility in the public sector, is funded directly by the Central Government, and is the highest-ranking tertiary care institution in India.

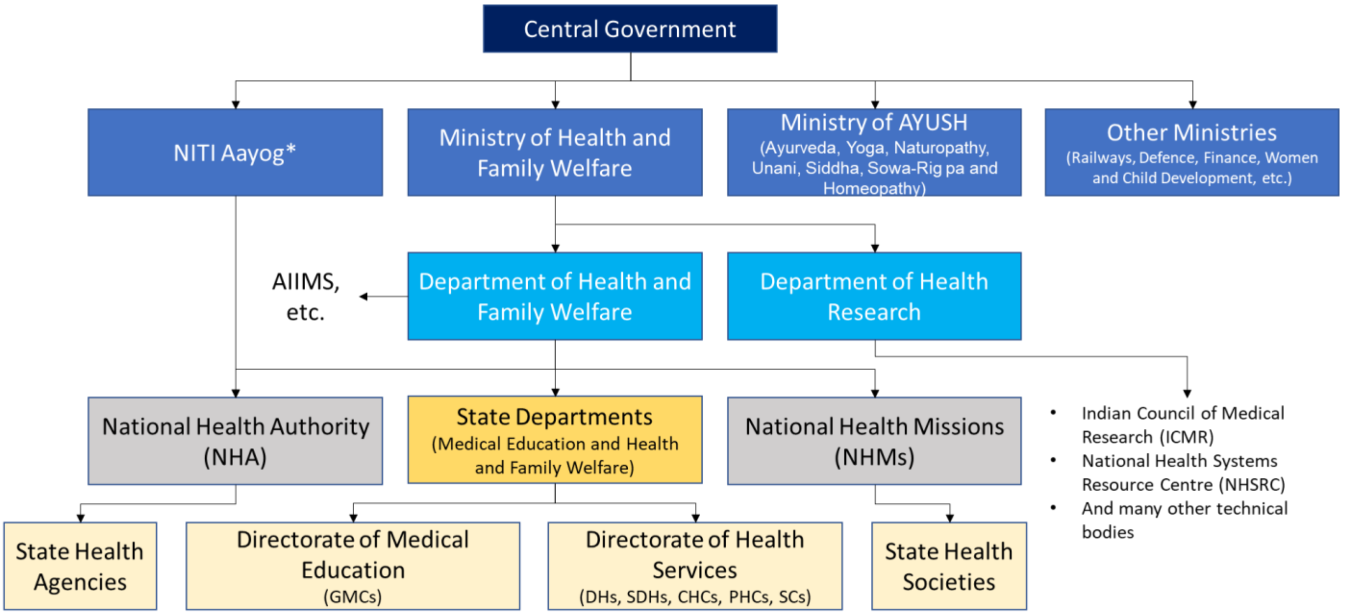

Figure 2: Governance structure for healthcare in India.[8] AIIMS: All India Institute of Medical Sciences; GMCs: Government Medical Colleges; DHs: District Hospitals; SDHs: Sub-Divisional Hospitals; CHCs: Community Health Centres; PHCs: Primary Health Centres; SCs: Sub-health Centres. *NITI Aayog is the premier policy think tank of the Government of India, providing directional and policy inputs.

Public sector health services in India are organized as a three-tier hierarchical system, comprising of the following levels: primary - subcenters and primary health centers (PHCs); secondary - community health centers (CHCs), sub-divisional hospitals (SDHs), and district hospitals (DHs); tertiary - medical colleges and teaching hospitals.[8] The number of functioning government health facilities in 2019 can be found in Figure 3. The private sector in India also plays a major role in providing inpatient and outpatient services, diagnostic services, and human resources for health, and in the pharmaceutical sector. It was reported that India had 43,486 private hospitals in 2020, of which 344 were private medical college hospitals.[8,9] The organization of the private sector in India is diverse, including for-profit, not-for-profit, charitable and religious organizations. About 70% of all outpatient visits and 58% of hospitalization visits occur in private facilities.

The healthcare workforce in India was estimated to be over 6 million in 2019 (Figure 4). To make it easier to regulate and implement standard practices in private healthcare facilities, they are regulated by the Clinical Establishments (Registration and Regulation) Act, 2010 along with government healthcare facilities – the one exception are hospitals owned, controlled, or managed by the Armed Forces.[8] Due to human resource challenges within the public health sector (e.g. absenteeism, low motivation, corruption), alongside shortages or unavailability of essential medicines and diagnostic services, patients switch to private providers despite higher out-of-pocket (OOP) costs or insurance financing. Insurance schemes (privately purchased or publicly funded) provide partial or complete coverage for hospitalization at empaneled hospitals (i.e. those where people can seek medical care for free of cost with governments supporting costs); however, outpatient care at private hospitals is mostly paid for out of pocket.

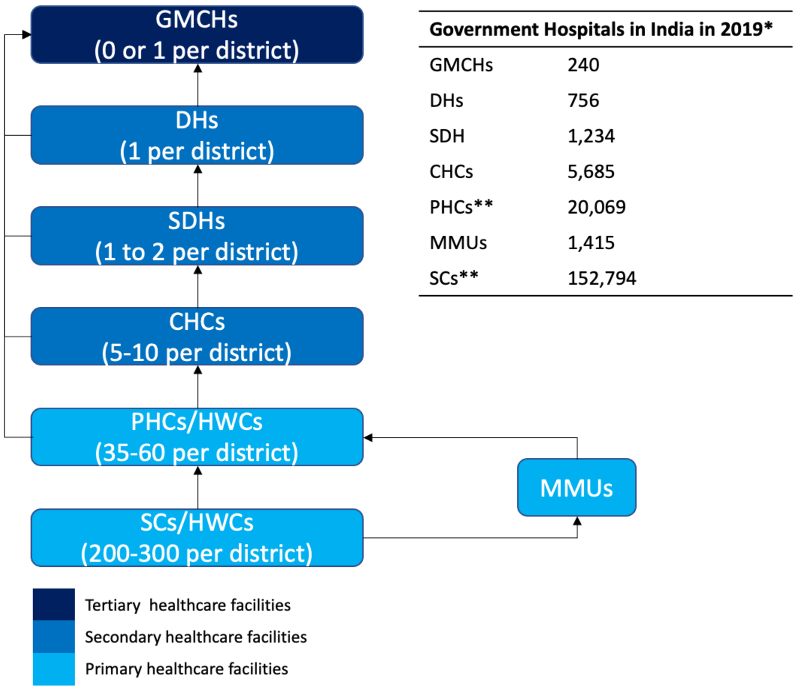

Government Hospitals in India in 2019*	
GMCHs	240
DHs	756
SDH	1,234
CHCs	5,685
PHCs**	20,069
MMUs	1,415
SCs**	152,794

Figure 3: Government healthcare facilities in India in 2019.[8] GMCHs: Government Medical College Hospitals; DHs: District Hospitals; SDHs: Sub-Divisional Hospitals; CHCs: Community Health Centres; PHCs: Primary Health Centres; MMUs: Mobile Medical Units; SCs: Sub-health centres. * In addition, Indian Railways and Employees' State Insurance Corporation (ESIC) run 128 and 155 hospitals, respectively. ** Some are being converted into Health and Wellness Centres (HWCs).

To tackle rising OOP expenditure for the care-seekers, several tax-funded health insurance programmes have been initiated since the mid-2000s. In 2018, a new national scheme, Pradhan Mantri Jan Arogya Yojana (PM-JAY), that integrated the health insurance schemes of several state governments under one umbrella was launched. The PM-JAY, with a focus on inpatient services, seeks to cover 500 million people from poor, and economically and socially disadvantaged groups, with an annual benefit package entitlement of INR 500,000 ($US 6,100) to households, including over 1500 treatment packages provided free to patients in need.[8,10–12]

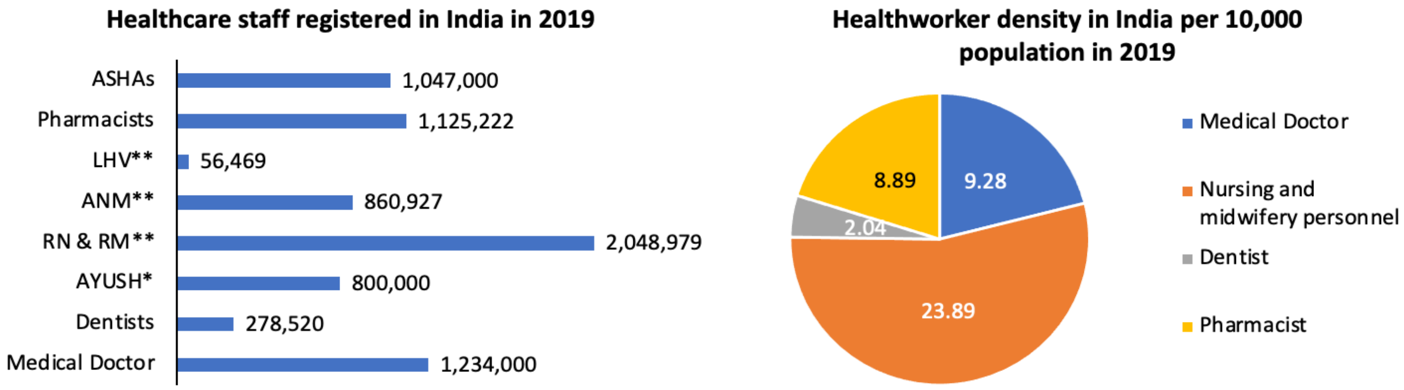

Figure 4: Healthcare workforce in India in 2019.[8] AYUSH: Ayurveda, Yoga and Naturopathy, Unani, Siddha, and Homeopathy; RN & RM: Registered Nurse and Registered Midwife; ANM: Auxiliary Nurse Midwife; LHV: Lady Health Visitor; ASHAS: Accredited Social Health Activists. * Data from 2018; ** Data from 2017

COVID-19

The first case of COVID-19 in India was reported on January 27, 2020 in Kerala.[13] As of May 2023, over 45 million cases had been reported, with a mortality rate of 1.2%, with Maharashtra having the highest infections. However, reports based on excess mortality indicate far more cases and deaths than have been officially reported.[14] The peak of the first COVID-19 wave was observed around mid-September 2020, with a peak daily oxygen demand of around 3,095 metric tonnes (MT). The second wave of infections peaked around early May 2021, with peak daily demand reaching over 11,000 MT - over threefold higher than the first wave, and the struggle to meet this oxygen demand become a defining moment in the pandemic globally.

Over 927 million samples have been tested for COVID-19, and over 2.2 billion vaccination doses have been administered since 16 January 2021.[15] To effectively manage the pandemic, the Government of India took steps to swiftly strengthen various aspects of healthcare services, such as surveillance and diagnostics, immunization and vaccinations, hospital bed capacity and equipment infrastructure, medical oxygen supply chain, data systems, and policies, guidelines and capacity building (Figure 5).[16–19] In addition, during the COVID-19 pandemic, $US 1.1 billion (₹ 8257.88 crores) and $US 2.8 billion (₹ 23,123 crores) were released by the Central government to the States as financial assistance under the Emergency COVID Response Plan (ECRP) in Phase 1 and Phase 2, respectively.

Under the Prime Minister's Citizen Assistance & Relief in Emergency Situation (PMCARES) fund, a sum of $US 115 million (₹ 933.63 crores) was allocated between June 2020 and June 2021 to procure 1,222 pressure swing adsorption (PSA) plants with a total oxygen manufacturing capacity of 1,750 metric tonnes.[16,18] Finally, the Central government and State governments received financial and technical support from a range of private, non-profit, multilateral and bilateral organizations, including: World Health Organization (WHO), United Nations Children's Fund (UNICEF), World Bank, United States Agency for International Development (USAID), Bill & Melinda Gates Foundation (BMGF), Clinton Health Access Initiative (CHAI), PATH, Jhpiego, Oxygen for India - a global initiative convened by the One Health Trust, Give India, and the Swasth Alliance, and finally support from many other corporate social responsibility partners.

Given India's size and large global diaspora, it played a large role in the fate of the pandemic. India's pandemic preparedness plan was largely abandoned in the face of a real pandemic and to an extent, the response was driven by political priorities and lockdowns.[20] There were several challenges in implementing the International Critical Care Guidelines for COVID-19 on infection control and using respirator masks.[21] Despite the significant health and economic impact, the pandemic offered an opportunity to invest in the public health infrastructure such as capacity building, public health research, and surveillance, which have been neglected over the past few decades. For instance, the increase in usage of non-invasive ventilation (NIV) using high flow nasal cannula (HFNC) as the initial mode for hypoxic respiratory failure when compared with other methods of NIV was highlighted.[22,23] The quality and regulation standards for the use of ventilators were also defined.[24]

Various strategies and principles were adopted by hospitals to mitigate challenges in administration, hospital space organization, management of staff and supplies, maintenance of standard of care, and specific COVID-19 care and ethics during the pandemic.[25,26] Additionally, substantial growth was observed for e-pharmacy businesses during the pandemic which have the potential to improve access to medicines, but come with public health challenges such as the sale of prescription-only medicines without prescription, the sale of substandard and

falsified medicines, and risks of consumer fraud and data privacy.[27] Mathematical modelling was developed to support optimal management of immunisation, diagnostic testing, patient caseload and medical oxygen supply. Examples of this include a model to ensure quick sample transport at minimum cost and to support optimal utilisation of testing laboratories;[28] a 10-day forecasts of the number of daily confirmed cases;[29] and a prediction equation using weight and height to estimate oxygen demand in the Asian-Indian population.[30] Furthermore, a pandemic management strategy comprising integrated satellite remote sensing, geographic information system (GIS), and local knowledge-based approach to effectively tackle the contagion of this disease was used in some areas.[31]

However, on the other side, an increase in the incidence of fires in Indian hospitals was reported during COVID-19 due to increased use of oxygen and alcohol-based disinfectants, high-power demand, increase in inflammable material in wards and limited fire safety infrastructure. Therefore, several changes in the equipment maintenance and management practices in Indian hospitals is required along with conformity with the Indian fire safety regulations.[32,33]

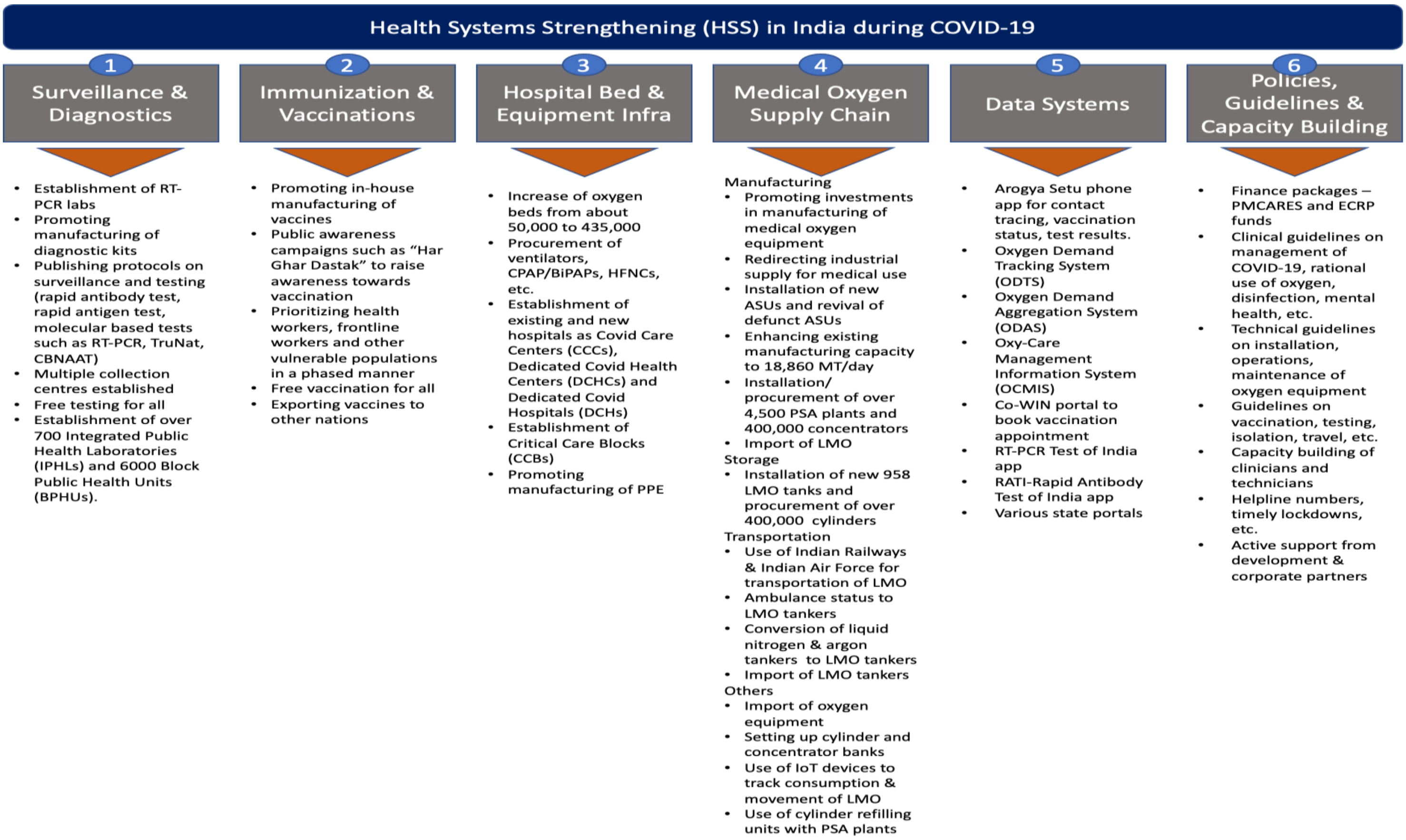

Figure 5: Strengthening of healthcare systems in India during COVID-19.[16–19] RT-PCR: Reverse Transcription – Polymerase Chain Reaction; CBNAAT: Cartridge Based Nucleic Acid Amplification Test; ICU: Intensive Care Unit; CPAP: Continuous Positive Airway Pressure; BiPAP: Bilevel Positive Airway Pressure; HFNC: High Flow Nasal Cannula; ASU: Air Separation Unit; PSA: Pressure Swing Adsorption; LMO: Liquid Medical Oxygen; IoT: Internet of Things; PMCARES: Prime Minister's Citizen Assistance & Relief in Emergency Situation; ECRP: Emergency COVID Response Plan.

Oxygen supply and clinical use landscape in India

Before COVID-19, India's daily liquid oxygen manufacturing capacity was about 9,000-10,000 MT/day, of which about 10% was utilized for medical purposes. Considering future surges in oxygen demand, the capacity was enhanced to 18,860 MT/day by December 2021 by increasing production from existing liquid oxygen plants, repairing defunct liquid oxygen plants, commissioning of PSA plants and procurement of oxygen concentrators. Since September 2020, over 4500 PSA plants have been installed, and if functional, would have a combined manufacturing capacity of about 4000 MT/day. The central and state funds, as well as donations from development partners, international agencies, and corporates, helped to install these 4,500 PSA plants across various public health facilities ranging from government medical colleges, district hospitals, civil hospital and some community health centres. In addition, the estimated 100,000 concentrators in the country would contribute to about 1000 MT/day. In India, INOX Air Products and Linde Gases have a combined market share of 60% in liquid oxygen manufacturing. Most of the liquid oxygen manufactured in the country is utilized by large-scale industries, such as the steel and electronics industry (25-35%). There are 1,600 liquid oxygen tankers owned by private manufacturers for transporting liquid oxygen.[34]

With a single-day COVID-19 peak of active cases of 3.6 million people, the demand for oxygen in India in May 2021 was about 11,200 MT/day. The manufacturing capacity at the time was 9,446 MT/day, leading to a considerable deficit of 1750 MT.[18,35] The country faced multiple challenges in the constituent parts of the oxygen supply chain linked to: 1) production; 2) transportation; 3) storage - Figure 6.

The regulation of medical oxygen in India involves multiple different actors and government departments. The Petroleum and Explosives Safety Organization (PESO), under the Department of Promotion of Industry and Internal Trade, Ministry of Commerce and Industry, regulates and monitors the installation and commissioning of liquid oxygen manufacturing units. PESO serves as the nodal agency for regulating the safety of hazardous substances such as explosives, compressed gas, and petroleum. Using the Gas Cylinder Rules 2016 and Static and Mobile Pressure Vessels (Unfired) Rules 2016, it regulates the installation and commissioning of LMO tanks, booster units to refill cylinders with PSA plants and the use of liquid oxygen cylinders and gaseous cylinders in a hospital setting.[36,37] Oxygen concentrators being medical devices, their registration and use is overseen by Central Drugs Standard Control Organization (CDSCO) under the Directorate General of Health Services, MoHFW.[38] Moreover, oxygen being an essential medicine, medical oxygen suppliers require a license based on the Drugs and Cosmetics Act of 1940, which gives the Indian Pharmacopoeia legal status to regulate purity requirements for medical oxygen. The National Accreditation Board for Testing and Calibration Laboratories (NABL) is a Constituent Board of the Quality Council of India that approves laboratories to test the quality of medical oxygen delivered to patients, primarily through PSA plants.[34] During the pandemic, cylinder and LMO refilling costs fluctuated based on the demand, and so the National Pharmaceutical Pricing Authority capped the price of medical oxygen. But this did not cover transportation costs levied by the vendors.[39] The sites for PSA plant installation across the hospitals were prepared mostly by either the National Highways Authority of India (NHAI) under the Ministry of Road Transport and Highways or Central Public Works Department (CPWD) under the Ministry of Urban Development. On the other hand, PSA plants were installed by Defence Research and Development Organisation (DRDO) under the Ministry of Defence, Ministry of Petroleum & Natural Gas, Ministry of Power, Ministry of Coal, Ministry of Railways or Central Medical Services Society (CMSS) under MoHFW.[40]

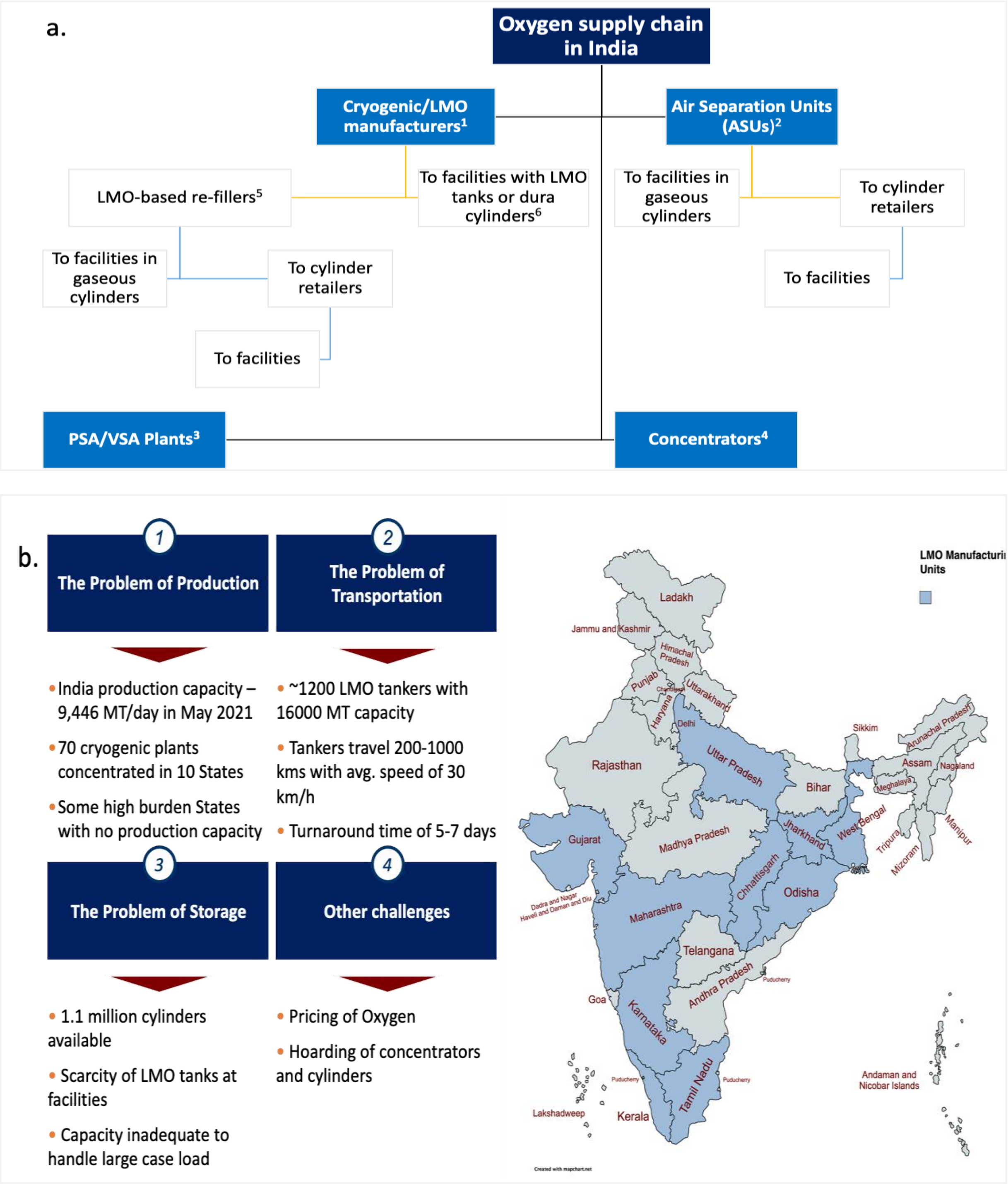

Figure 6: (a) Medical oxygen supply chain and (b) the challenges associated with it in India[34]

[1]Cryogenic/LMO manufacturers: Using a cryogenic air separation unit, oxygen is stored in liquid form with a purity of >99%.

[2]ASUs: Using a cryogenic air separation unit, oxygen is stored in gaseous form with a purity of >99%.

[3]PSA/VSA plants: Using pressure or vacuum swing adsorption technology operating at ambient temperatures, generates oxygen with a purity of 93±3%.

[4]Concentrators: Using PSA technology operating at ambient temperatures, generates oxygen with a purity of >82%, connected directly to patients.

[5]LMO-based re-fillers: They receive oxygen in liquid form from cryogenic/LMO manufacturers, convert it into gaseous form, and fill it into cylinders

[6]Dura cylinders: Mobile LMO tanks that are connected directly to MGPS and may be refilled on or off-site.

In India, the most common storage sources of medical oxygen in healthcare facilities are compressed gas cylinders and LMO tanks, while oxygen concentrators and PSA plants are the most common forms of production. In tertiary care hospitals, LMO tanks and PSA plants typically act as primary sources of oxygen by providing oxygen through the medical gas pipeline system, whereas gaseous cylinders and concentrators act as secondary and reserve supplies, respectively. PSA plants and cylinders are the primary sources of oxygen in secondary care hospitals, whereas only cylinders are the primary source in primary care hospitals. An ideal hospital journey for a patient in India requiring oxygen therapy can be seen in Figure 7.[41]

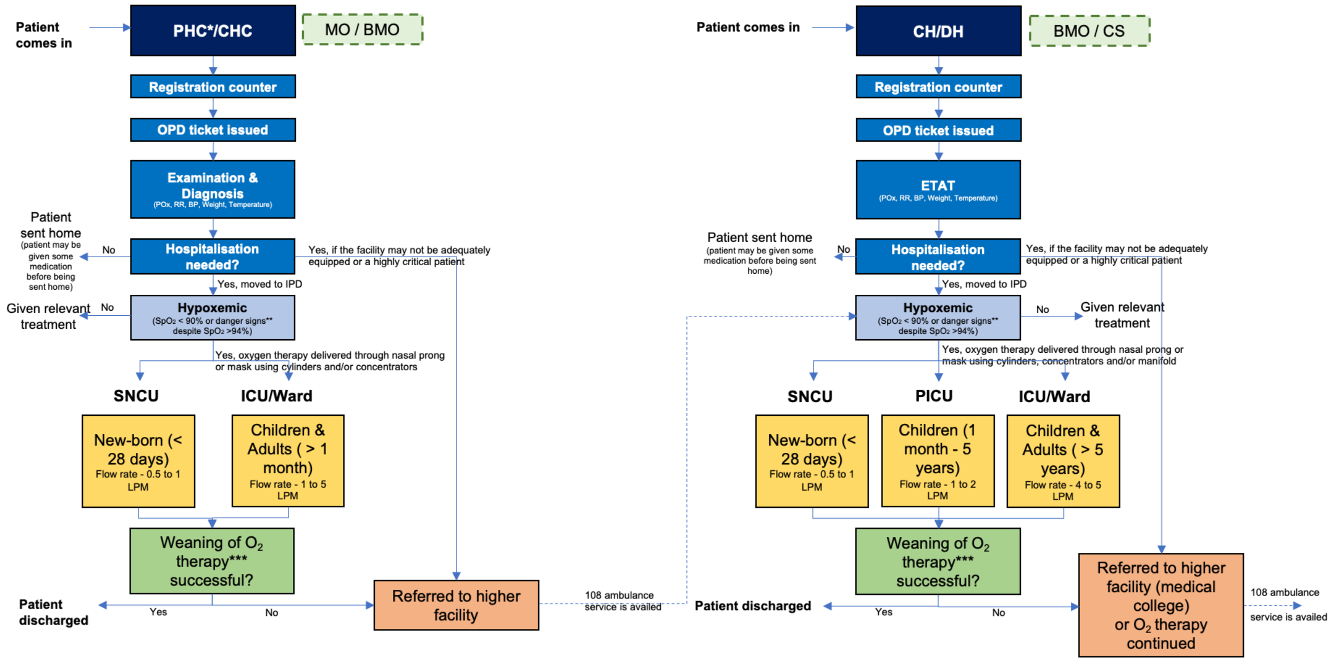

Figure 7: Hospital journey of a patient requiring oxygen therapy in India.
BMO: Block Medical Officer; BP: Blood Pressure; CH: Civil Hospital/Sub-divisional Hospital (SDH); CHC: Community Health Centre; CMHO: Chief Medical & Health Officer; CS: Civil Surgeon; DH: District Hospital; ETAT: Emergency Triage Assessment and Treatment; HR: Human Resource; ICU: Intensive Care Unit; IPD: In Patient Department; LPM: Litres Per Minute; MO: Medical Officer; OPD: Out Patient Department; PHC: Primary Health Centre; PICU: Paediatric Intensive Care Unit; POx: Pulse Oximetry**;** RR: Respiratory Rate; SNCU: Special Newborn Care Unit. * PHCs are mostly delivery points where new-borns and mothers are primary receivers of oxygen therapy. ** Danger signs are lethargy, unconsciousness, nasal flaring, vomiting, or convulsion. *** Weaning is successful if SpO_2 is within desired range after stopping therapy for 15 mins and rechecked after 1h

During the COVID-19 pandemic, MoHFW regulated the use of oxygen for COVID-19 management and issued guidelines for management of COVID-19 in adults and children. It categorized patients into three groups: 1) 80% of cases are mild and do not require oxygen; 2) 17% of cases are moderate and can be managed on non-ICU beds, and 50% of these may require oxygen at 10 LPM; 3) 3% of cases are severe ICU cases requiring oxygen at 24 LPM.[42,43] In addition, various State health departments published their own guidelines for medical oxygen use.[44,45]

After the COVID-19 pandemic, returning to routine service delivery (i.e. lower oxygen demand), has thrown up a new challenge for hospitals and State administrators. Many State governments and other stakeholders have expressed concerns over the 'high costs' incurred by keeping PSA

plants operational, including electricity bills, costs of fuel for diesel generator sets and technically qualified and trained HR to operate them safely. Due to lower utilisation, they are now also being referred to as "white elephants". Allowing them to lie idle could mean letting these plants become defunct over time, which would be a waste of the estimated $US 730 million (₹ 6000 crore) investment that had gone into installing and commissioning them. To ensure the maximum utilisation of PSA plants as a source of medical oxygen, an Oxygen Technical Advisory Committee (TAC) has been formed under the stewardship of the National Health Systems Resource Centre (NHSRC). Their mandate is to provide: guidelines on the purity of oxygen being delivered by PSA plants and their regular testing, the requirement of medical gas pipeline system (MGPS) infrastructure with PSA plants, mixing of PSA plant generated oxygen with LMO and their usage in ICUs, refilling cylinders with PSA plants, operational cost comparison with other sources of oxygen, use of solar power as a source of energy for their operations, audits and mock drills. The TAC consists of experts from various domains within the oxygen ecosystem, including representatives from the NHSRC, health commissioners, oxygen nodal officers from state health departments, medical experts, professors, industry experts (PSA plant and LMO manufacturers), oxygen quality testing experts, and engineers.

The need for a national medical oxygen grid (NMOG)

The COVID-19 pandemic highlighted the impact of mHealth and Industry 4.0 technologies (such as the Internet-of-Things (IoT), big data and artificial intelligence) based-applications in pandemic responses especially in LMICs. With minimum human interface, they make the healthcare system more resilient and provide significant benefits to underserved populations.[1] During COVID-19, these digital applications augmented accessibility, communication, and public health practices through telemedicine, automated GPS and Bluetooth-based contact and quarantine tracing, and real-time information dissemination; however, it also raised questions on maintaining personal privacy. The uptake of these applications was enhanced due to high smart phone penetration and digital literacy, and availability of free or subsidized internet data; thus, promoting greater flow of information without significant financial implications.[28,46–50] As the Indian subcontinent is highly susceptible to natural disasters, a disaster supply chain structure was proposed that integrates information and digital technologies for multi-agency information sharing, coordination and decision making for implementation of better response practices.[51] The list of digital solutions used in the Indian State of Kerala during the COVID-19 pandemic response can be found in Appendix 1.[47,52]

The Indian Central Government took multiple steps to strengthen the oxygen supply chain to tackle the health crisis and prevent the deaths of many COVID-19 patients (Figure 5). These included efforts for improving oxygen production, enhancing tanker availability to optimize logistics, improving oxygen storage at the last mile, and easing procurement norms. In addition, anticipating the challenges in medical oxygen management during the second wave of the pandemic, the MoHFW expedited the development and deployment of the Oxygen Demand Aggregation System (ODAS) as well as the Oxy-Care Management Information System (OCMIS) as comprehensive IT solutions to aggregate the estimated demand of oxygen from health facilities across the country. They have proven to be invaluable assets that allowed the district, state, and national authorities to make meaningful decisions based on the aggregated data to optimize faster delivery of oxygen. However, these systems also have several limitations (Figure 8), as follows:

- Need for a computer or laptop to enter the data.
- Realtime access to internet to submit the data.
- High data entry burden on a single nodal person through one user ID per facility.
- No demand-based predictive and decision analytics available at the facility level.

In the post COVID-19 scenario, always ensuring the availability of oxygen in adequate quantities regardless of demand fluctuations is critical. In a vast and densely populated country such as India, ensuring oxygen availability is even more significant as the health system is expanding rapidly, but is fragmented. The supply chain challenges in India are not unique to medical oxygen as other industries such as electricity and oil and gas, also have very different points of production and consumption with significantly varying demand. The risks associated with such a setup could be mitigated by establishing interconnected grids which comprises of networks of producers and consumers to facilitate efficient and timely delivery of a product. [34]

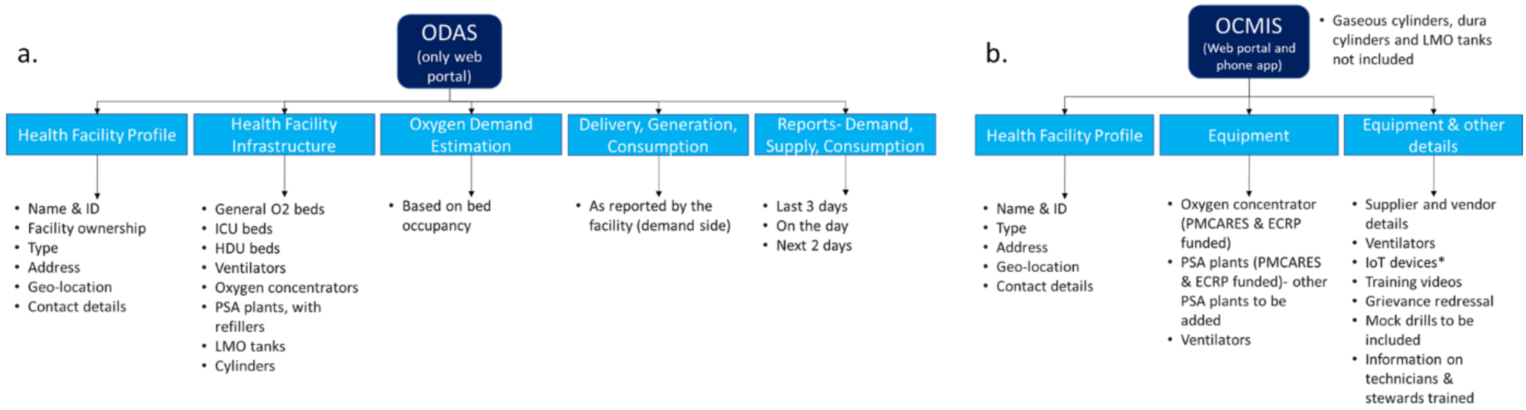

Figure 8: (a) Oxygen Demand Aggregation System (ODAS). (b) Oxy-Care Management Information System (OCMIS)

ICU: Intensive Care Unit; HDU: High Dependency Unit; PMCARES: Prime Minister's Citizen Assistance & Relief in Emergency Situations; ECRP: Emergency COVID Response Plan; PSA: Pressure Swing Adsorption; IoT: Internet of Things. *IoT devices: They are pieces of hardware, such as sensors that are programmed for certain applications and can transmit data over the internet or other networks.

A National Medical Oxygen Grid (NMOG) could solve this need, and is be based on four principles:

i) preference for creating large storage capacity
ii) preference for creating an interconnected network to allow asset reallocation based on demand
iii) preference for public–private partnerships
iv) working towards achieving oxygen self-sufficiency.

The grid mechanism would be enabled by a robust IT platform, which would include both traditional manual and technology-enabled (IoT device-based) automatic data collection.[34] The technology-enabled data inputs may be captured through specific sensors for pressure, quality, level, and flow rate. They may also involve other technologies such as GPS coordination and QR code scanning to overcome the challenges associated with laborious manual data entry. A detailed comparison between the NMOG IT platform and the oxygen management systems used during COVID-19 in India are presented in Table 3. The IT platform would ensure coordination between different stakeholders and help monitor resource prioritization and allocation during a

crisis.[34] Moreover, with a governance framework at the facility, district, state, and national levels, the IT grid would provide the following functions and features (Figure 9):

- 7-day demand prediction tool to estimate the demand for medical oxygen and different types of hospital beds (low flow vs high flow) based on the CAGR of data of the recent past. This could also notably act as an outbreak early warning system if demand considerably exceeds predicted trends.
- Cost comparison tool for optimal utilization of oxygen sources such as LMO, PSA plants, and cylinders.
- Oxygen metric unit conversion tool to report demand/supply/consumption in metric tonnes (MT) or any other desired metric system.
- Differences in estimated vs actual consumption tool to promote rational use of oxygen.
- Global capacity building repository tool on clinical and technical aspects of medical oxygen.
- Decision analytics tool to support asset allocation and patient management.

Metrics	ODAS	OCMIS	NMOG
**Technology**			
Intuitive user interface			✓
Master-satellite users			✓
Personalised GUI			✓
Web portal	✓	✓	✓
Phone app		✓	✓
**Features & Functions**			
Facility profile creation	✓	✓	✓
Oxygen infrastructure management	✓	✓	✓
Bed infrastructure management	✓		✓
Disease-wise patient management			✓
Mock drill management		✓	✓
Supplier management		✓	✓
Order placing & management (demand side)	✓	✓	✓
Order placing & management (supply side)			✓
Predictive analysis of oxygen demand, bed demand and disease-specific patient caseload			✓
Decision analytics on asset allocation and patient management			✓
Oxygen source operational cost analysis tool			✓
Rational use of oxygen tool			✓
Oxygen unit conversion tool			✓
Oxygen knowledge products repository			✓
Map based data visualisation			✓
**Help & support**			
User manual	✓	✓	✓
Video tutorial			✓
Accessible technical support/ grievance redressal mechanism		✓	✓

Table 3: Comparison of NMOG IT platform with the oxygen management systems used during COVID-19 in India

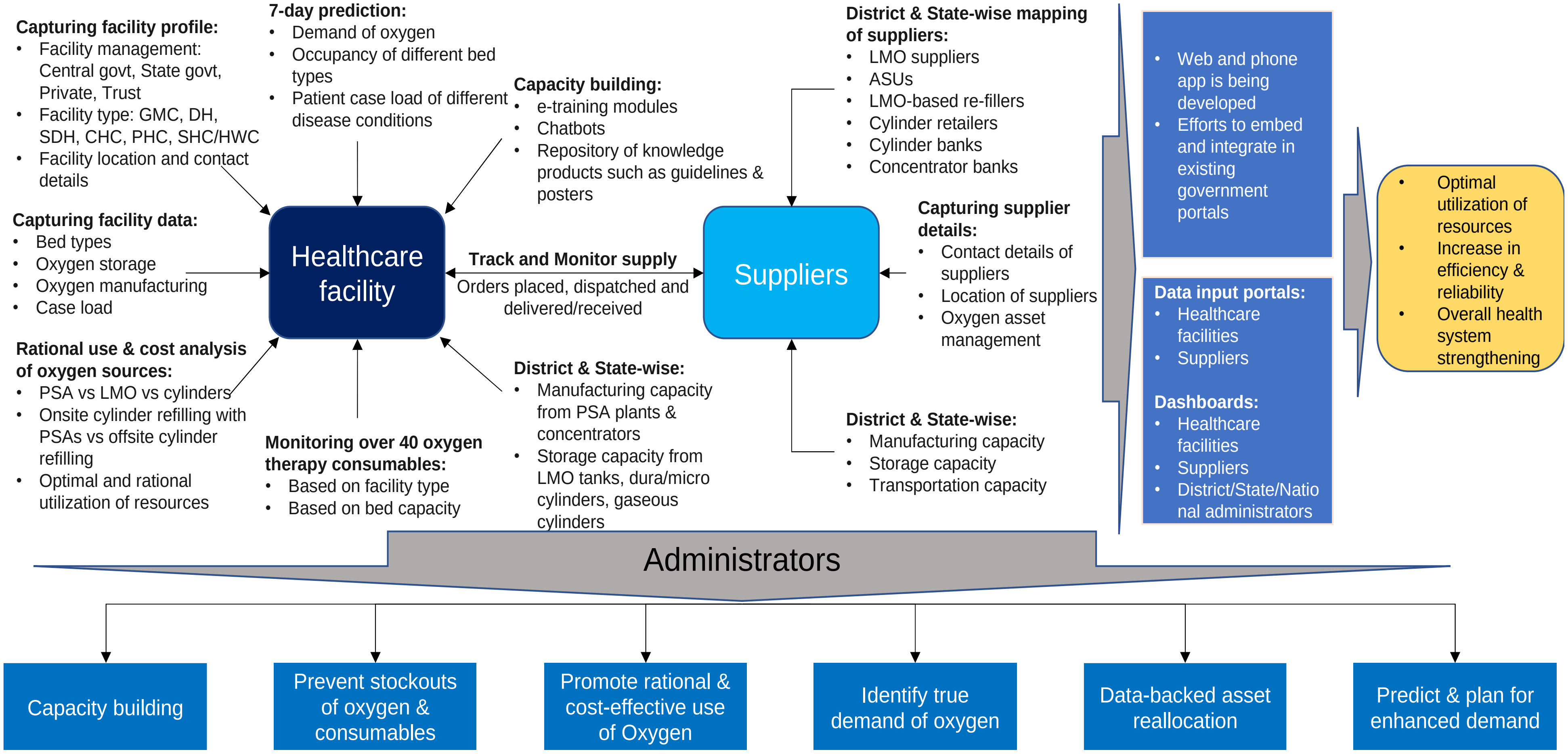

Figure 9: NMOG's functions and features

In addition, to further improve data entry on the NMOG portal, the following features have been included:

- Accessibility from desktop or laptop, and also from a phone app (both android and iOS version).
- Supports data entry in offline mode through a phone app when internet connection may not be temporarily available; however, would need internet connection before the end of the day for submission to the main server else the data for the day would be marked as blank.
- Concept of Master-Satellite data entry operator has been implemented:
  - Master data entry operator:
    - Would review, validate and submit the data to the main server.
    - Can also enter the data.
    - Can create and delete multiple satellite data entry operators each of whom would be tasked to manage an oxygen asset(s) or a ward(s) in the hospital.
  - Satellite data entry operator:
    - Would enter and submit data for asset(s) being managed by them.

Key messages

- The medical production capacity in India was rapidly scaled during COVID-19, but as demand has returned to normal, challenges of how to maintain new oxygen production equipment have emerged.
- To strengthen the medical oxygen supply chain, strategic storage capacity should be enhanced, along with leveraging technologies such as IoT, GPS, and QR codes for data entry, and asset management and (re)allocation.
- NMOG provides an effective solution to integrate data from multiple sources, to better distribute oxygen supplies to meet demand, through forecasting demand, consumption, supply, and storage patterns.
- To support the development and nationwide implementation of the NMOG, other structural support systems, such as developing policies and guidelines on the uptake and utilisation of NMOG, along with strengthened advocacy, communications, and partnerships with all the relevant stakeholders, are needed required.

Additional methods information

Academic and grey literature were screened. The grey literature sources searched were: Government of India circulars, notifications, press releases, advisories, public orders, regulations and guidelines, reports (or data) from MoHFW website, World Bank, Unicef, WHO, and other development organisations.

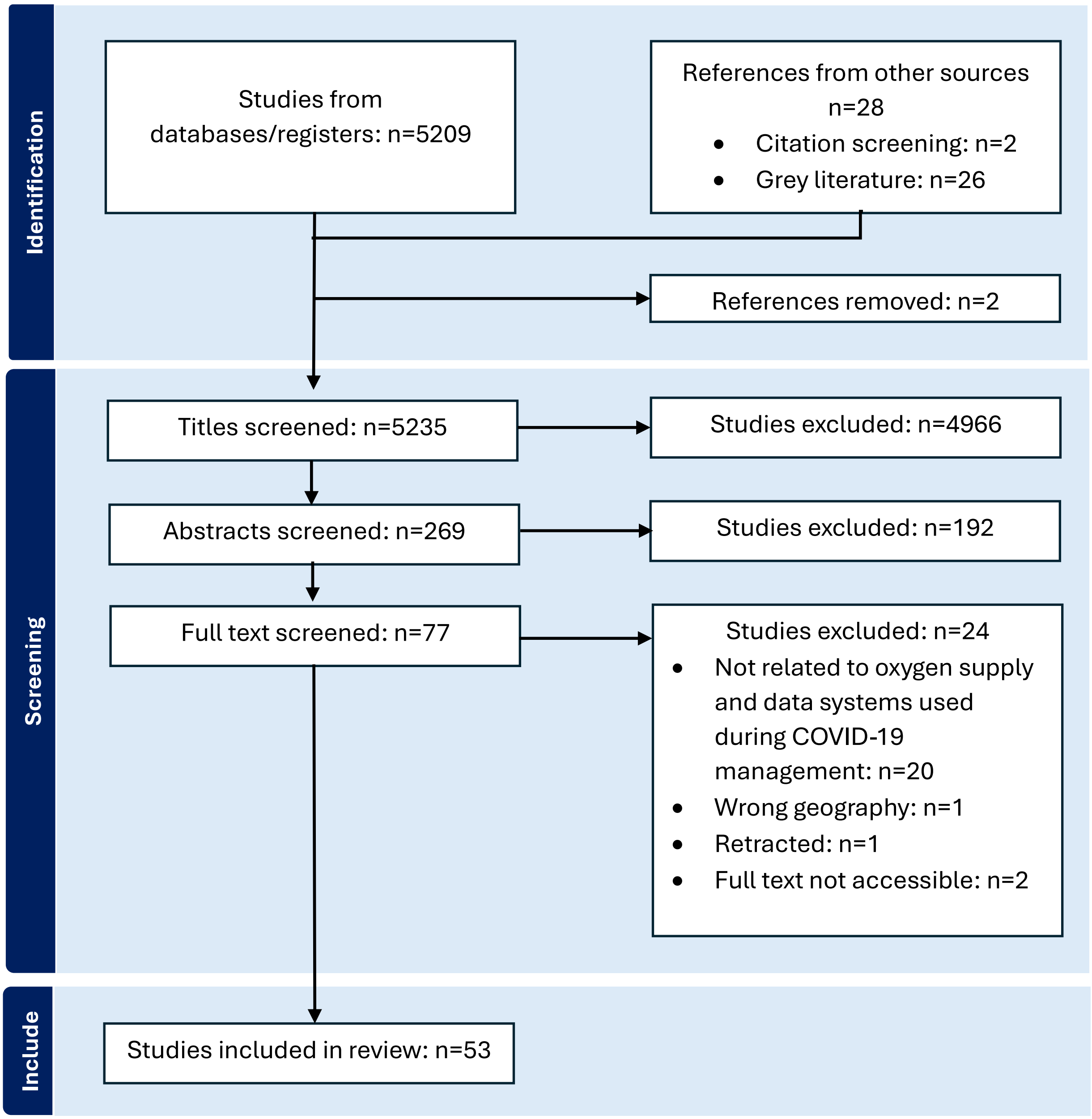

Figure 10: Academic and grey literature inclusion

Appendix I: Digital solutions used in Kerala for COVID-19 management

#	Solution name	Description	Solution type
1.	Arogya Mitra	Provides information on Kerala's COVID-19 response activities	Web portal
2.	Arogya Setu	Provides information on risks, best practices and relevant COVID-19 advisories	Mobile app
3.	Arogyakeralam	Kerala's National Health Mission (NHM) portal, which includes COVID19 related information	Web portal
4.	BeSafe Tracking	Tracks an individual by device/phone Global Positioning System (GPS)	Mobile app
5.	BlueTeleMed	Tele-counselling and telemedicine	Mobile app
6.	Break the Chain campaign	Conveying important COVID-19 containment messages through animation	Online campaign
7.	Break the Chain diary	Presents a route map of places visited	Mobile app
8.	Chiri (smile) telecall	Tele-counselling for children's mental health involving frontline health workers (ASHAs, Anganwadi workers)	Helpline/tele-call
9.	CoronaSafe Network—quiz	A multilingual quiz on COVID-19 myths and protective measures	Web portal
10.	CoronaSafe Network	An open-source disaster management platform	Web portal
11.	Covid Safety	Bluetooth and GPS-enabled tracker to track proximity to a COVID-19 positive person	Mobile app
12.	COVID-19 Jagratha	Real-time surveillance, care and support for people affected or quarantined by COVID-19	Mobile app/ web portal
13.	Department of Health & Family Welfare	Information on state health department activities, data visualisation on COVID-19 status, online training modules and communication material	Web portal
14.	DISHA-1056	A toll-free 24/7 telehealth helpline providing physical and mental health guidance, counselling, and information	Web portal/helpline
15.	Doctor just a phone call away	Tele-counselling and telemedicine for police personnel	Helpline, WhatsApp or video consultation
16.	Emergency Response Support System 112 Service	Provides rapid assistance in response to citizen 'distress signals' in the form of voice calls, SMSs, email, and web requests	Helpline
17.	eSanjeevani OPD	National tele-consultation service	Mobile app/web portal
18.	GoK Dashboard	Government of Kerala dashboard visualising COVID-19 status including daily reporting, quarantine report, test results and hot spots	Web portal
19.	GoK- Direct Kerala	Shares announcements, updated guidelines, and safety tips for visitors to Kerala	Mobile app
20.	Kerala Battles COVID	Consolidation of COVID-19 management updates for public accessibility	Web portal

21.	Kerala Health Disease Surveillance	Disease surveillance app using phone location; also provides awareness on COVID-19	Mobile app
22.	Kerala Police Home Quarantine Assistance	Coordinates the delivery of non-clinical services to people	Mobile app
23.	Kerala Sannadha Sena (volunteers)	Enrols and coordinates community volunteers	Mobile app/web portal
24.	Kerala Superhero app	Tracks the near real-time location of volunteer assets like ambulance drivers, delivery crews and medical personnel	Mobile app
25.	Koode	Enables people under home quarantine to self-report and collate health details daily	Mobile app
26.	Koode helpline	Tele-counselling by Ayurveda doctors	Helpline
27.	Local self-government Kerala pandemic dashboard	Provides information on local government COVID-19 activities and services	Web portal
28.	Ottakkalla Oppamundu ('not alone with you')	Provides psychosocial support to children	Helpline
29.	People Move	Tracks and delivers non-clinical services to people	Mobile app
30.	Pol-App	Kerala police information programme	Mobile app
31.	PRASANTHI	Free service provision or directory	Helpline
32.	Project Eagle Eye	Drone tracking of lockdown violations	Drones
33.	Shops app	Online shopping	Mobile app
34.	WhatsApp chatbot	Provision of COVID-19 information and directory to services	Mobile app
35.	Kerala health online training	Educational videos about COVID-19 on YouTubes	Mobile app/web portal

ASHAs: accredited social health activists; SMSs: short message services.

References

1 Gowda NR, Siddharth V, Kumar P, Vikas H, Swaminathan P, Kumar A. Constrained medical oxygen supply chain in India during COVID-19: Red-tapism, the elephant in the room? *Disaster Med Public Health Prep* 2023; **17**: e296.

2 Baheti AD, Nayak P. Covid-19 in India: Oxygen shortages and a real world trolley problem. *bmj* 2022; **376**.

3 Tiwari T, Upadhyaya DN, Dheer Y, Singh GP, Tiwari S. Formulation of an oxygen policy to ensure adequate supply of oxygen reserves during the second wave of COVID-19 pandemic. *Med Gas Res* 2023; **13**: 39.

4 UNICEF Data: Monitoring the situation of children and women. https://data.unicef.org/ (accessed April 24, 2023).

5 World Development Indicators. https://data.worldbank.org/indicator (accessed April 24, 2023).

6 Cause of Death Statistics 2017-2019. Office of the Registrar General, Ministry of Home Affairs, Government of India, 2023 https://censusindia.gov.in/nada/index.php/catalog/44752 (accessed April 24, 2023).

7 Sachs JD, Lafortune G, Fuller G, Drumm E. Implementing the SDG stimulus. *Sustain Dev Rep* 2023.

8 Selvaraj S, Karan K A, Srivastava S, Bhan N, & Mukhopadhyay I. India health system review. New Delhi: World Health Organization, Regional Office for South-East Asia, 2022.

9 Jaffrelot C, Jumle V. Private Healthcare in India: Boons and Banes. Institut Montaigne https://www.institutmontaigne.org/en/expressions/private-healthcare-india-boons-and-banes#:~:text=India%20has%20a%20total%20of,59%2C264%20ICUs%2C%20and%2029%2C631%20ventilators. (accessed April 25, 2023).

10 National Health Mission. Ministry of Health & Family Welfare, Government of India https://nhm.gov.in/index1.php?lang=1&level=1&sublinkid=1213&lid=167 (accessed April 25, 2023).

11 Gupta I, Patel N. International Health Care System Profiles: India. The Commonwealth Fund https://www.commonwealthfund.org/international-health-policy-center/countries/india (accessed April 25, 2023).

12 Pradhan Mantri Jan Arogya Yojana (PM-JAY). National Health Authority https://nha.gov.in/PM-JAY.html (accessed April 25, 2023).

13 Andrews M, Areekal B, Rajesh K, *et al.* First confirmed case of COVID-19 infection in India: A case report. *Indian J Med Res* 2020; **151**: 490.

14 14.9 million excess deaths associated with the COVID-19 pandemic in 2020 and 2021. World Health Organization, 2022.

15 COVID-19. Government of India https://www.mygov.in/covid-19/ (accessed May 3, 2023).

16 Ministry of Health & Family Welfare 2020 ACHIEVEMENTS. Press Information Bureau, Ministry of Health and Family Welfare, Government of India, 2020 https://www.pib.gov.in/PressReleasePage.aspx?PRID=1684546 (accessed May 3, 2023).

17 INITIATIVES & ACHIEVEMENTS-2021. Press Information Bureau, Ministry of Health and Family Welfare, Government of India, 2022 https://pib.gov.in/PressReleseDetailm.aspx?PRID=1787361 (accessed May 3, 2023).

18 Centre undertakes multiple initiatives to enhance Oxygen availability, distribution and storage infrastructure. Press Information Bureau, Ministry of Commerce & Industry, Government of India, 2021 https://pib.gov.in/Pressreleaseshare.aspx?PRID=1717459 (accessed May 3, 2023).

19 PIB'S BULLETIN ON COVID-19. Press Information Bureau, Ministry of Information and Broadcasting, Government of India, 2021 https://pib.gov.in/PressReleasePage.aspx?PRID=1720925 (accessed May 3, 2023).

20 Purushothaman U, Moolakkattu JS. The politics of the COVID-19 pandemic in India. *Soc Sci* 2021; **10**: 381.
21 Tirupakuzhi Vijayaraghavan BK, Nainan Myatra S, Mathew M, *et al*. Challenges in the delivery of critical care in India during the COVID-19 pandemic. *J Intensive Care Soc* 2021; **22**: 342–8.
22 Jose A, Vivekananthan S, Sivakumar M. Comparison of HFNC and NIV Use in Hypoxic Respiratory Failure in COVID-19 Pneumonia Patients: A Retrospective Study. *Indian J Crit Care Med* 2022; : S105–6.
23 Sekar L, Sehgal IS, Kajal K, *et al*. Factors Associated With Non-invasive Oxygen Therapy Failure in COVID-19 Pneumonia: A Single Center, Retrospective Study in a Tertiary Hospital in North India. *Cureus* 2022; **14**.
24 Pandey N, Thakkar A, Walia R. Quality and Regulation Standards for Ventilator as Medical Devices in India. *Int J Res Pharm Sci* 2022; : 246–53.
25 Jog S, Kelkar D, Bhat M, *et al*. Preparedness of acute care facility and a hospital for COVID-19 pandemic: what we did! *Indian J Crit Care Med Peer-Rev Off Publ Indian Soc Crit Care Med* 2020; **24**: 385.
26 Nibudey A, Vidya Baliga S. Preparing hospitals in India for covid-19 pandemic. *Int J Res Pharm Sci* 2020; : 333–41.
27 Miller R, Wafula F, Onoka CA, *et al*. When technology precedes regulation: the challenges and opportunities of e-pharmacy in low-income and middle-income countries. *BMJ Glob Health* 2021; **6**: e005405.
28 Devi Y, Patra S, Singh SP. A location-allocation model for influenza pandemic outbreaks: A case study in India. *Oper Manag Res* 2021; : 1–16.
29 Chakraborty T, Ghosh I. Real-time forecasts and risk assessment of novel coronavirus (COVID-19) cases: A data-driven analysis. *Chaos Solitons Fractals* 2020; **135**: 109850.
30 John N, Thangakunam B, Devasahayam AJ, Peravali V, Christopher DJ. Maximal oxygen uptake is lower for a healthy Indian population compared to white populations. *J Cardiopulm Rehabil Prev* 2011; **31**: 322–7.
31 Kanga S, Sudhanshu, Meraj G, Farooq M, Nathawat M, Singh SK. Reporting the management of COVID-19 threat in India using remote sensing and GIS based approach. *Geocarto Int* 2022; **37**: 1337–44.
32 Malviya AK, Mulchandani M, Singh J, Singh A, Gupta A. Increasing Hospital Fires During the COVID-19 Pandemic in India: Are the Current Policies and Infrastructure Adequate? *J Patient Saf* 2022; **18**: e869–70.
33 Paliwal B, Kothari N, Purohit A. Oxygen Cylinder Fire during the COVID-19 Pandemic. *Indian J Crit Care Med Peer-Rev Off Publ Indian Soc Crit Care Med* 2022; **26**: 974.
34 Aggarwal A, Bhushan I, Mahurkar D, Mehta R, Roy S, Laxminarayan R. Blueprint for a National Medical Oxygen Grid in India. Bengaluru, India: One Health Trust, 2022.
35 Mirza M, Verma M, Sahoo SS, Roy S, Kakkar R, Singh DK. India's Multi-Sectoral Response to Oxygen Surge Demand during COVID-19 Pandemic: A Scoping Review. *Indian J Community Med Off Publ Indian Assoc Prev Soc Med* 2023; **48**: 31.
36 Gas Cylinder Rules. 2016. https://peso.gov.in/web/gas-cylinder-rules-2016.
37 Static and Mobile Pressure Vessels (Unfired) (SMPV(U)) Rules. 2016. https://peso.gov.in/web/smpv-u-rules-2016 (accessed May 6, 2023).
38 Advisory regarding Oxygen Concentrators suitable for COVID-19 case management in Home settings. 2021; published online May 16. https://cdsco.gov.in/opencms/resources/UploadCDSCOWeb/2018/UploadPublic_NoticesFiles/Advisory%20on%20Oxygen%20Concentrators.pdf (accessed Oct 3, 2023).
39 National Pharmaceutical Pricing Authority, Department of Pharmaceuticals, Ministry of Chemicals and Fertilizers, Government of India. NPPA steps in to cap price of Liquid Medical Oxygen and Medical Oxygen cylinders. 2020; published online Sept 26. https://pib.gov.in/PressReleasePage.aspx?PRID=1659266.

40 The Government has Sanctioned 1563 PSA Oxygen Generation Plants. Press Information Bureau, Ministry of Health and Family Welfare, Government of India, 2021 https://pib.gov.in/PressReleseDetailm.aspx?PRID=1780145 (accessed May 6, 2023).
41 Kapoor G, Jain A, Gandhi C, *et al*. Guidance document on use of medical oxygen [Manuscript in preparation]. Bengaluru, India: One Health Trust.
42 CLINICAL GUIDANCE FOR MANAGEMENT OF ADULT COVID-19 PATIENTS. 2023; published online Jan 5. https://www.mohfw.gov.in/pdf/ClinicalGuidanceforManagementofAdultCOVID19Patientsupdatedason05thjan2023.pdf (accessed May 7, 2023).
43 Revised Comprehensive Guidelines for Management of COVID-19 in Children and Adolescents (below 18 years). 2022; published online Jan 20. https://www.mohfw.gov.in/pdf/RevisedComprehensiveGuidelinesforManagementofCOVID19inChildrenandAdolescentsbelow18years.pdf (accessed May 7, 2023).
44 SOP for Medical Oxygen Use and Fire Safety for Public and Private Hospitals of Madhya Pradesh. 2021; published online May. https://media.path.org/documents/SOP_MP_Medical_Oxygen_Use__Fire_Safety_Guidelines.pdf (accessed May 7, 2023).
45 Medical Oxygen Management System. 2021; published online Oct. https://media.path.org/documents/Guidebook_on_Medical_Oxygen_Management_System_Meghalaya_Med_Res.pdf (accessed May 7, 2023).
46 Winkie MJ, Nambudiri VE. A tale of two applications: lessons learned from national LMIC COVID applications. *J Am Med Inform Assoc* 2023; **30**: 781–6.
47 Ummer O, Scott K, Mohan D, Chakraborty A, LeFevre AE. Connecting the dots: Kerala's use of digital technology during the COVID-19 response. *BMJ Glob Health* 2021; **6**: e005355.
48 Annamalai L, Arulraj M, Nagamani P, Jai Shankar G. Geo-Information Communication Technology (Geo-ICT) Framework to Prevent Spread of Corona Virus Disease (COVID-19). *J Indian Soc Remote Sens* 2022; **50**: 1163–75.
49 KEK V, Nadeem SP, Meledathu Sunil S, Suresh G, Sanjeev N, Kandasamy J. Modelling the strategies for improving maturity and resilience in medical oxygen supply chain through digital technologies. *J Glob Oper Strateg Sourc* 2022; **15**: 566–95.
50 Dash SP. The impact of IoT in healthcare: global technological change & the roadmap to a networked architecture in India. *J Indian Inst Sci* 2020; **100**: 773–85.
51 Dash BP, Dixit V. Disaster supply chain with information and digital technology integrated in its institutional framework. *Int J Prod Res* 2022; : 1–20.
52 Sahasranamam S, Soundararajan V. Innovation ecosystems: what makes them responsive during emergencies? *Br J Manag* 2022; **33**: 369–89.

THE LANCET Global Health
COMMISSION ON MEDICAL OXYGEN SECURITY

Country Case Study: Malawi

Financing a medical oxygen system

Michael Krautmann[1], Raphael Kayamankadzanja[1], Carina King[2], Mirwais Rahimzai[3], Tisungane Mvalo[4], Lisa Smith[1]

1. Programs and Innovation, Market Dynamics, PATH, USA; 2. Department of Global Public Health, Karolinska Institutet, Sweden; 3. FHI360, USA; 4. University of North Carolina Project Malawi, Malawi.

Case study focus

This case study focuses on the financing of medical oxygen in Malawi, following the COVID-19 pandemic. In 2021, Malawi produced its first National Oxygen Ecosystem Road Map, which included a fully costed plan for national scale-up of oxygen services. The costed plan was developed using Ministry of Health Planning Department costing guidelines and was estimated at approximately US$76 million to cover gaps in equipment and supplies to meet the projected national demand. In this case study we aim to describe the extent to which the planned financing has been achieved, key barriers and successes in ensuring funding is allocated to oxygen, and understand the stakeholders involved in this process.

Country Context

Demography, economy, and epidemiology

Malawi is a nation of 20.4 million people located in southern Africa. Population growth is a modest 2.6% per year. Approximately 18% of the population, equating to 3.7 million people, are children under the age of five. Almost 82% of the population lives in rural areas, while only 14% of the population has access to electricity. Malawi is classified by the World Bank as a low-income country, with a per-capita GDP of USD $645. Annual GDP growth is relatively low at 0.9% for 2022.[1] The Malawian economy has been characterized by high inflation in recent years, averaging 21% in 2022 and reaching 33%% in November 2023 at the time of writing.[2]

Total healthcare spending in Malawi was $44 per capita as of 2019, with 57% coming from external development assistance, 24% coming from government spending, and 18% coming from private/out-of-pocket spending.[3] This has translated into a life expectancy of 63 years (60 for males, 68 for females) in 2022, and an under-five mortality rate of 39 per 1000 live births.[1] The top five causes of mortality across all ages are: 1) HIV/AIDS; 2) neonatal disorders; 3) acute lower respiratory infections; 4) tuberculosis; 5) diarrheal diseases[4] – the top causes all have oxygen needs.

Currently, the reported burden of chronic respiratory conditions such as COPD is low, accounting for 1.2% of annual deaths.[4] However, presence of abnormal lung function is as high as 40% in the general adult population, indicating a high undiagnosed burden of non-communicable respiratory morbidity.[5] Reliable data on the number of surgeries is lacking, but there is evidence of a large unmet surgical need and therefore, oxygen needs for surgical care (both intra-operative and post-operative) and critical care patients should increase as surgical capacity expands.[6]

Health system

Health system in Malawi is organized at three levels – primary, secondary, and tertiary – which are linked by a referral system. These health services are delivered through a network of public, private-for-profit, and faith-based providers, as shown in Table 1.[7]

- The primary care level consists of (i) community healthcare workers (named Health Surveillance Assistants), who provide integrated community case management care for children under-five, and (ii) health centers, which are generally staffed by nurses and medical assistants and provide outpatient care. Respiratory care services at the primary care level focus on screening patients for referral to higher-level facilities and may also include basic oxygen delivery for maternal care. Functional pulse oximeters are inconsistently available at this level of care.
- Secondary care is delivered at district hospitals, which provide general inpatient care and limited high-dependency care. Oxygen services at this level should be available for limited paediatric and adult high-dependency units, surgery and maternal and newborn care.
- Tertiary care is provided by 4 central hospitals that are distributed geographically throughout the country. Oxygen services at this level should be available for ICU and critical care, surgery, general wards, maternal and newborn care and chronic care.

	Primary health posts, dispensaries, health centres	Secondary referral hospitals	Tertiary referral hospitals	Total
Government/public	625	45	4	674
Christian Health Association of Malawi (CHAM)	136	51	0	187
Private for-profit	359	17	0	376
Other (mostly NGO)	173	7	0	180
**Total**	1293	120	4	1417

Table 1: Distribution of health facilities in Malawi

The government operates the largest number of health facilities nationwide, including all four tertiary referral hospitals. In government facilities, all health services – including oxygen delivery – are provided free of charge. The Christian Health Association of Malawi (CHAM), a faith-based non-profit organization, is the second largest individual healthcare provider. CHAM facilities complement public facilities and are partially subsidized by the Ministry of Health, which prioritizes funding for CHAM facilities located more than eight kilometers from the nearest public facility. Small user fees may be charged for oxygen services at these facilities.

Within these facilities, Malawi generally faces a healthcare workforce shortage, with 1.49 health workers per 1,000 population, compared to the WHO target of 4.45 per 1,000.[8] Figure 1 from a recent study underpinning the Malawi Human Resources for Health Strategic Plan, shows the vacancy rates (i.e., workforce shortage rates) against Ministry of Health targets for specific heath worker cadres.[8] All cadres face a staffing shortage, and the most severe shortages exist for lab assistants, pharmacists, and pharmacy technicians and assistants. Notable in this figure is the absence of biomedical engineers as a cadre of the healthcare workforce.

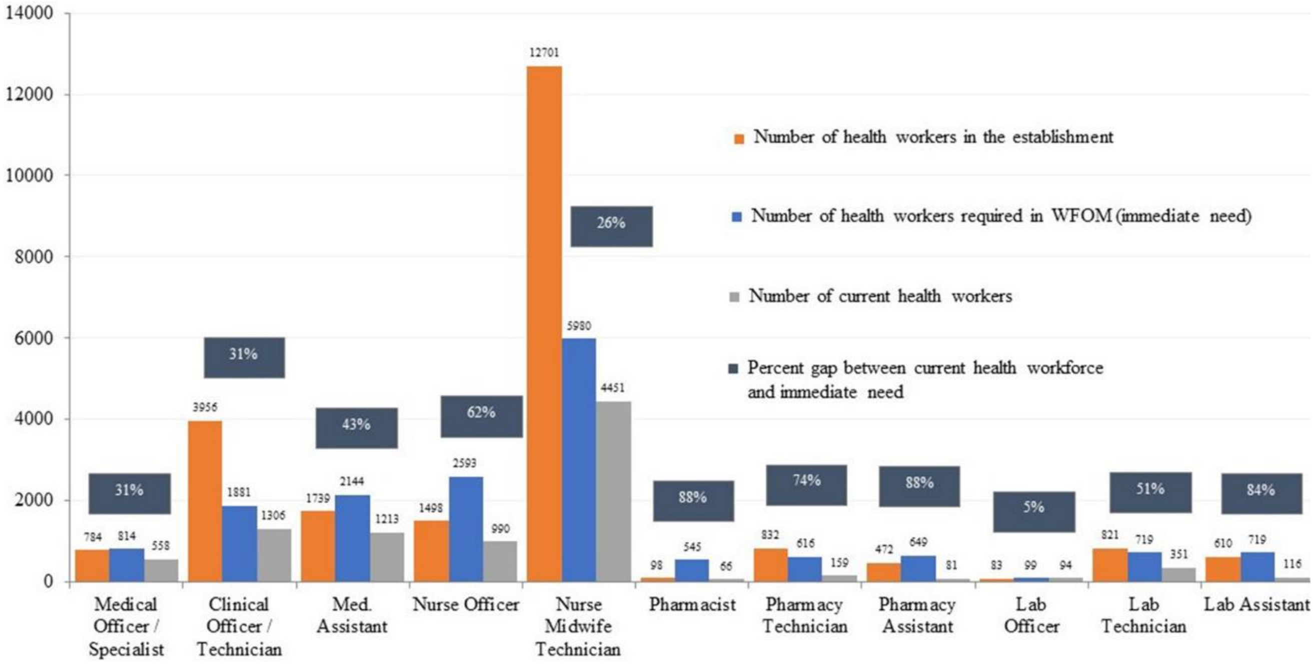

Figure 1: Current workforce, modelled workforce needs, and workforce targets set by MOH establishment (Berman et al 2022)[8]

COVID-19

Malawi registered its first case of COVID-19 on April 2, 2020. As of September 2022, Malawi had four waves of COVID-19 with 87,981 confirmed cases, resulting in 2,680 deaths (for a case fatality rate of 3.0%). The first wave of the pandemic began in April 2020 and caused a reported 6,039 cases. A second wave hit in mid-December 2020, causing a reported 28,807 cases. A third wave emerged in June 2021 and continued until the end of September 2021 and was responsible for a reported 27,129 cases. The fourth wave emerged in December 2021 with a reported 23,366 cases (Figure 2).[9]

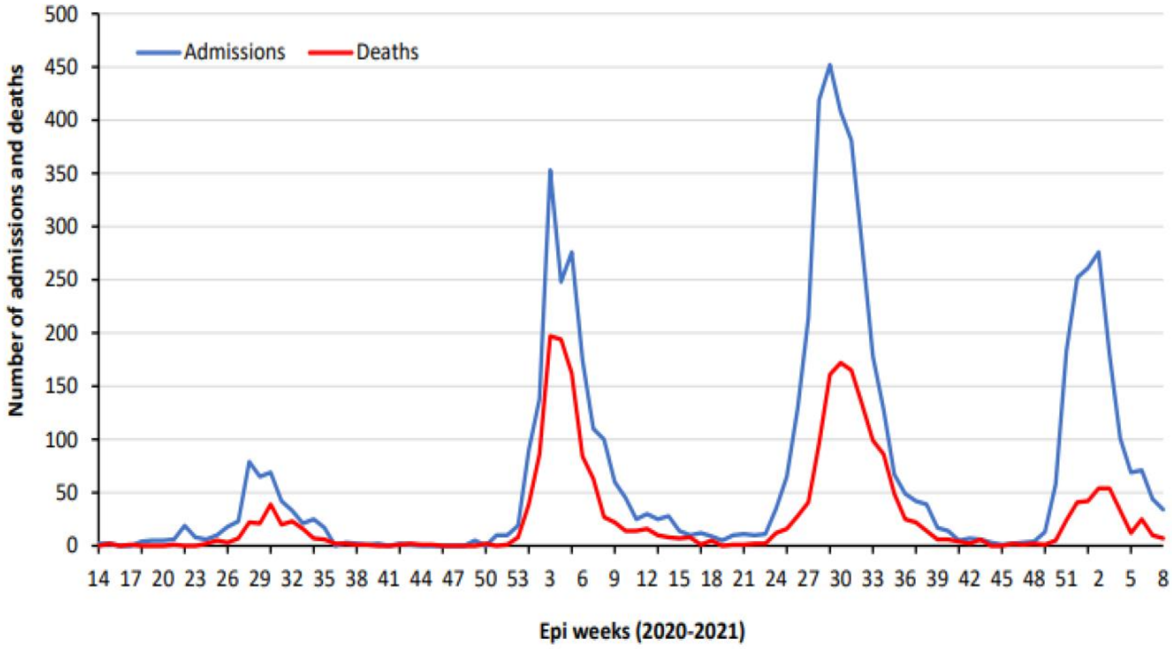

Figure 2: Hospital admissions and Deaths for COVID-19 in Malawi from April 2020 – December 2021 (PHIM surveillance unit).[9]

The third wave saw the highest number of hospitalizations, while the second wave recorded the highest number of deaths from COVID-19. Admitted COVID-19 patients receiving oxygen varied by wave as well, with fewest requiring oxygen during the 4th Omicron wave (17% vs 62% in wave 3).[10] Deaths from COVID-19 affected males more than females (64.2% of those who died were male), and tended to occur in the older population groups, with a median age of 61 years.[9] The government of Malawi's response to the COVID-19 pandemic included bolstering the healthcare workforce by recruiting an additional 5,622 health workers, including 179 medical doctors, 325 nursing officers, 86 clinical officers and 233 laboratory personnel. The government also declared a national emergency in March 2020 and again in January 2021, which limited individual travel and required schools to close in an attempt to curtail the spread of the virus but stopped short of enforcing a complete lockdown.

These states of emergency led to a reduction in access to essential health services and challenges in delivering routine care.[11] For HIV services, studies showed a significant reduction in demand for HIV testing, voluntary medical male circumcision, and pre-exposure prophylaxis.[12] For maternal, newborn and child health (MNCH) services, the COVID-19 response led to reductions in outpatient department consultations, institutional deliveries, and reductions in antenatal care visits.[13,14] A reduction in overall outpatient visits was also reported.[15]

Oxygen supply and clinical use landscape in Malawi

Oxygen supply and demand

As part of the 2021 National Oxygen Ecosystem Roadmap development, the Ministry of Health estimated the national oxygen need to be approximately 1.4 billion liters per year (Table 2).[16] This was estimated using the UNICEF Oxygen System Planning tool.[17]

Facility type	Number of health facilities	Number of hypoxemic cases per year	Monthly oxygen demand (L)	Monthly oxygen demand in "J" cylinders (6,800 L)	Annual oxygen demand (L)	Annual oxygen demand in "J" cylinders (6,800 L)
Central hospital	4	100,579	16,942,200	2,492	206,130,000	30,313
Community hospital	2	1,107	181,350	27	2,206,500	324
District hospital	25	171,868	29,561,790	4,347	359,668,380	52,892
Health centre	457	618,396	39,472,350	5,805	480,246,900	70,625
Mission hospital	27	55,471	20,682,090	3,041	251,632,080	37,005
Rural hospital	25	59,497	8,316,270	1,223	101,181,420	14,880
**Total**	**541**	**1,006,918**	**115,156,050**	**16,935**	**1,401,065,280**	**206,039**

Table 2: Oxygen demand from the Medical Oxygen Ecosystem roadmap

These estimates represent a routine care scenario and do *not* factor in surges in oxygen need due to spikes in COVID-19 cases that were present at the time of planning, or surge needs that will occur in the future. These surges were significant enough to cause widespread oxygen shortages, especially during the second and third COVID-19 waves in Malawi.

Medical oxygen supply in Malawi comes in several forms. The primary bulk liquid oxygen supplier is AFROX Malawi, which has two liquid oxygen storage tanks in the central and southern region of Malawi. AFROX imports liquid oxygen from South Africa and converts to it to gas in country. Health facilities then buy oxygen cylinder refills, with procurement coordinated by individual facilities, who pay directly to AFROX based on the number of cylinders consumed. Most cylinders found in public facilities have historically been procured via this method. A biomedical equipment survey conducted by PATH in 2021 found a low availability of cylinders in many facilities.[18] However, this is likely an underestimate of current availability, and doesn't necessarily reflect newer cylinder distribution models, given the numerous new procurements done during the COVID-19 pandemic.

Additional bulk oxygen capacity is available via Pressure Swing Adsorption (PSA) oxygen plants that have been installed in a number of public sector hospitals by the Ministry of Health and its partners. As of April 2023, ten active PSA plants are co-located at health facilities across the country, with nine more planned or awaiting procurement and installation. These PSA plants are located at mostly district hospitals and Central hospitals. Only one community hospital has a PSA plant on site. As these PSA plants become functional, a shift towards a 'hub and spoke' model coordinated by larger hospitals may occur. In terms of clinical use, most cylinders are used directly at the bedside of patients.

Oxygen concentrators also play a major role in oxygen delivery in Malawi, even in facilities that have other bulk oxygen production capacity. A biomedical equipment survey in 2022 reached 490 health facilities and documented the availability of oxygen concentrators (Table 3), and pulse oximeters (Table 4).[19] This report highlighted that availability of concentrators and pulse oximeters remains a major challenge, particularly at the lowest levels of the health system, and

that even when equipment is available it is often non-functional. A major challenge identified was facilities receiving devices from numerous sources, both before and during the COVID-19 pandemic, and as a result facilities end up managing multiple different brands and models of the same device type. For example, this survey identified 50 unique concentrator manufacturers and 49 pulse oximeter manufacturers;[19] many of these devices have specialized training needs but lack adequate documentation. Another challenge is that this equipment are often not equally distributed at a sub-national and sub-district level, resulting in inequitable access.[20]

Facility Type	Number of facilities included	Average number of concentrators per facility	Percent of concentrators that are functional	Percent of facilities with at least one functional concentrator	Average availability gap vs. MOH standards
Central Hospitals	4	167	74%	100%	34%
District Hospitals	24	26	71%	88%	74%
Rural/Community Hospitals	2	1.5	100%	50%	94%
Health Centres	460	0.7	86%	24%	85%
**Total**	**490**	**1630**	**76%**	**28%**	**84%**

Table 3: Availability of oxygen concentrators in public-sector facilities in Malawi [19]

Facility Type	Number of facilities included	Average number of oximeters per facility	Percent of oximeters that are functional	Percent of facilities with at least one functional oximeter	Average availability gap (%) vs. MOH standards
Central Hospitals	4	120.8	89%	100%	0%
District Hospitals	24	20.5	98%	83%	48%
Rural/Community Hospitals	2	0	--	0%	100%
Health Centres	460	1	97%	18%	68%
**Totals**	**490**	**1434 units total**	**95%**	**21%**	**66%**

Table 4: Availability of pulse oximeters in public-sector facilities in Malawi [19]

Clinical use of oxygen in Malawi

Regulation and oversight of medical oxygen lie primarily with the Pharmacy Medicines Regulatory Authority (PMRA). However, standards that regulate oxygen production and delivery exist only in draft form as of December 2023. The Ministry of Health, through its Directorate of Clinical Services, has been primarily responsible for developing clinical guidelines and training resources for healthcare workers.

In Malawi, 42% of all infant deaths occur within the neonatal period, and 14% of all newborns have low birth weight, putting them at greater risk of complications and death.[21] Birth asphyxia and intrapartum complications necessitating newborn resuscitation, prematurity and low birth weight, and severe infections such as pneumonia and sepsis account for most newborn deaths—and all of these may manifest with hypoxemia. Previous studies have found the prevalence of hypoxia in this population to range from 8% to 28%.[22]

Among children under-five, hypoxemia is the primary fatal complication of pneumonia, affecting 13.3% of children with pneumonia according to the WHO.[23] The UN Inter-Agency Group on Child Mortality Estimates conclude that about 26,000 children died in Malawi in 2021; the Malawi MOH estimates that 11% (2,860) of these deaths were attributed to pneumonia.[24] A study by McCollum et al. in five hospitals found that 5.3% of hospitalized Malawian children under 15 years old were hypoxemic and, while there was more hypoxemia among children with respiratory illness, it was also spread across children with other diagnoses, such as malaria and sepsis.[25] Pulse oximetry was not routinely used, and only 22.5% of children needing supplemental oxygen, as defined by WHO guidelines, were given oxygen.[25] A recent analysis in Malawi has also reported implementation of WHO guidelines without pulse oximetry will miss high risk cases of pneumonia, highlighting the need for routine pulse oximetry to improve outcomes.[26]

Oxygen is also used among surgical patients. Hypoxemia occurs in about 30% of all surgical cases in the early post-operative period, and it is recommended that all surgical cases receive supplemental oxygen, which has been demonstrated to reduce the incidence of postoperative hypoxemia. Data for this group in Malawi specifically was not found. Similarly, very few studies have estimated the prevalence of hypoxemia in the rest of the population in this setting. In a previous study in two hospitals, 4% of admitted adults were found to be hypoxemic, with only 11% were receiving oxygen treatment at the time of assessment.[27] A study from 2019 reported that COPD prevalence was 0.9% in the population.[28] However, we found no published reports on oxygen use among this patient population outside the hospital, and lack of data about chronic respiratory disease in Malawi has been reported as a key gap previously.[29]

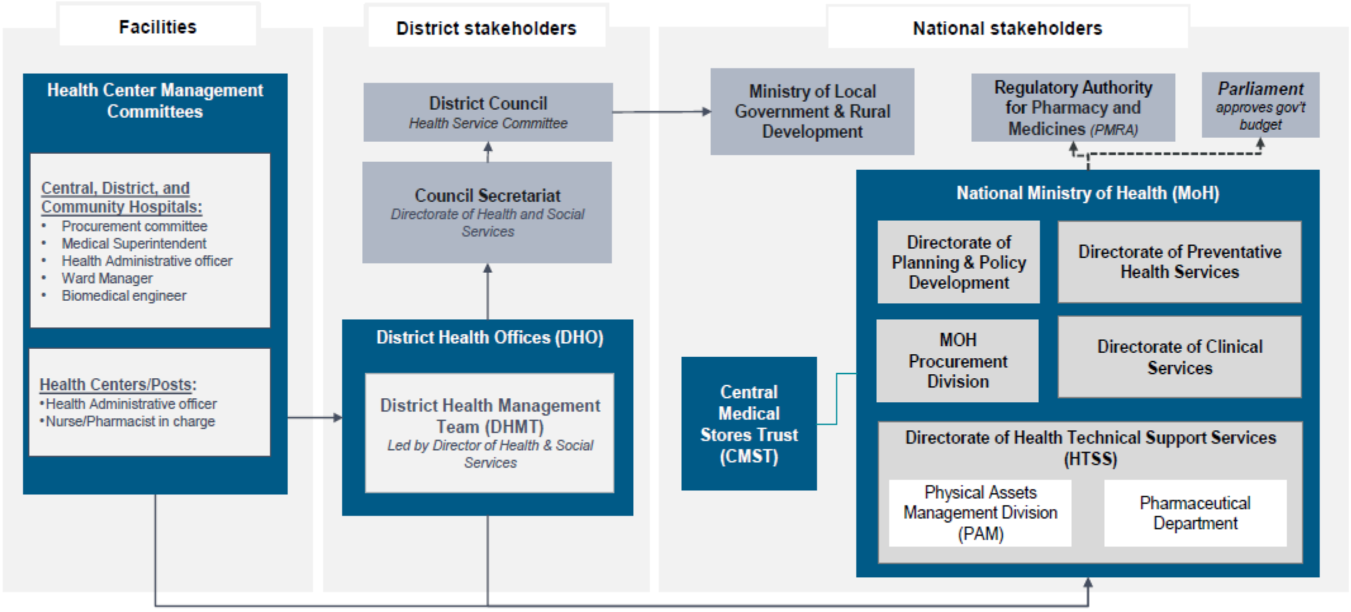

Figure 3: Public sector medical devices stakeholders in Malawi

Financing an oxygen system

Organisation of the system

Governance of the Malawian health system has been nominally decentralized since the passage of the Local Government Act of 1998. Primary and secondary healthcare delivery are managed by Health Center Management Committees, who are in turn overseen by District Health Management Teams, and ultimately by District Councils and their leadership (Figure 3).[30]

In practice, however, these decentralization reforms were never fully implemented, and have been reversed in some cases. Local governments account for less than 5% of total government spending,[31] so district health budgets are heavily dependent on central-level transfers, which often come earmarked and with little opportunity for district input. Since 2005, District Council leadership has been appointed by central-level entities (the Office of the President and Cabinet, and the Ministry for Local Government, Unity, and Culture) rather than by local elections which was the case between 1998 and 2005.[32,33] For these reasons, de facto power and decision-making authority within the health system remains highly centralized, as depicted in the government stakeholder influence map in Figure 4.

For respiratory care equipment, the Ministry of Health's Physical Assets Management (PAM) Division, which sits within the Directorate of Health Technical Services (HTSS) has the broadest scope of responsibility and authority of any individual stakeholder. They are responsible for most aspects of medical device management, including planning and conducting procurement, determining device allocation and arranging distribution, and overseeing maintenance and repair of devices. However, these responsibilities intersect with other MoH departments in important ways, leading to some overlap and an evolving division of roles between departments. For example:

- While PAM prepares budget requests for medical device procurement and operation (based on compiled submissions from the DHMTs), final funding decisions and allocations are made by the MoH Planning Department.
- PAM must also work in coordination with the MoH Procurement Division for any procurements that they manage directly; the Procurement Division's mandate is to ensure adherence to key transparency and competitiveness regulations.
- PAM is also not the only agency who can conduct procurements; the Central Medical Stores Trust (CMST), which sits outside the MoH, has historically focused on pharmaceutical products, but has increasingly taken on procurement of medical device consumables and smaller medical devices. This leads to a blurring of lines between PAM's and CMST's procurement duties.
- While respiratory care devices are managed under PAM, oxygen as a drug, falls under the purview of the MoH Pharmacy Division as it is considered an essential medicine. Both divisions fall under the same directorate (HTSS), but this dynamic has the potential to complicate management decisions and information sharing related to oxygen delivery. For example, the Pharmacy division has a robust supply chain management system for medicines, but that system has not been extended to medical devices in part because of the separation of roles between the two divisions.

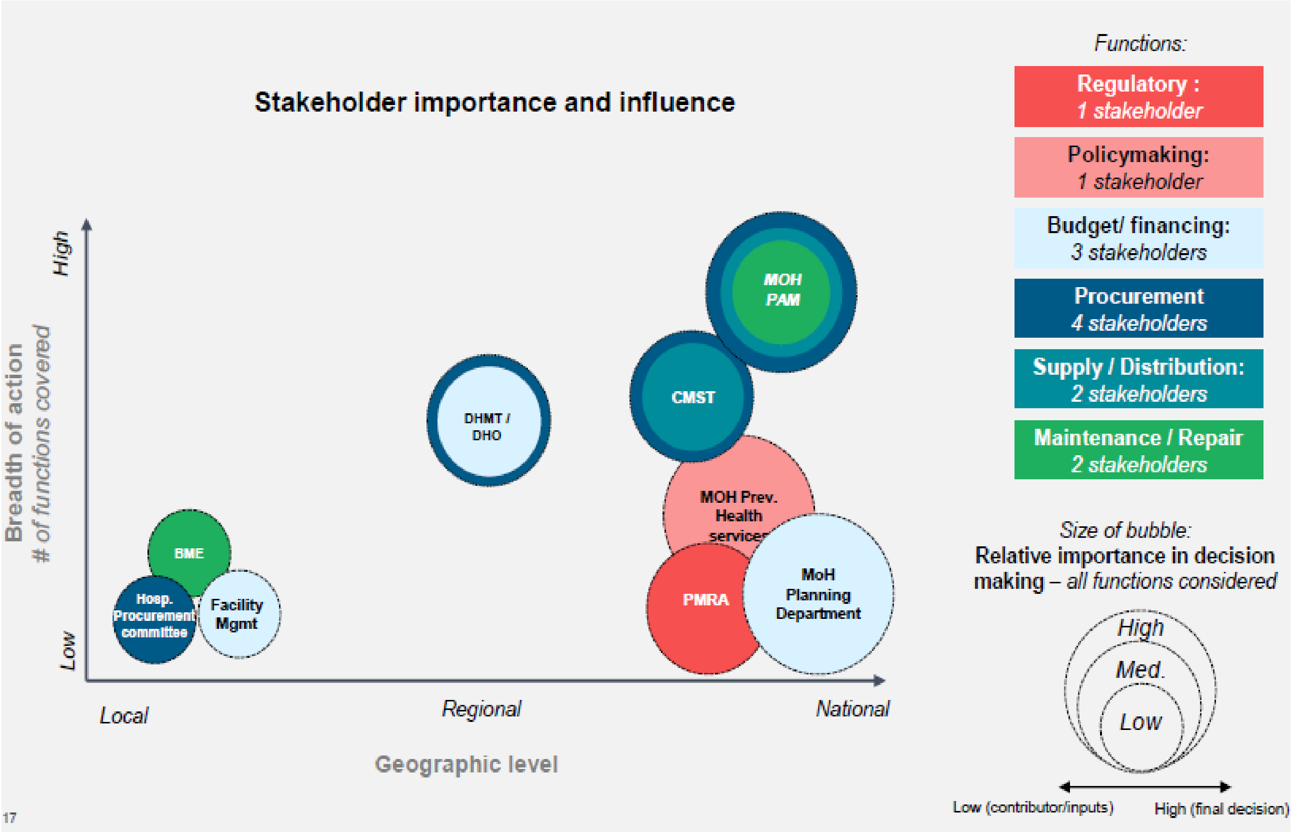

Figure 4: Malawi public sector stakeholder influence map

In addition, there are a large number of private, non-profit, and donor organizations that play a role in oxygen and respiratory care delivery. A non-exhaustive list of key organisations, their relative power and interests are shown in Table 5. Malawi's stakeholder structure and political economy were a critical influence in shaping the government's response to COVID-19, the passage of the Oxygen Ecosystem Roadmap, and ultimately the financing of oxygen in general.

Engagement across this broader group of stakeholders occurs primarily through the National Oxygen Taskforce. At the height of the COVID-19 pandemic the Taskforce met bi-weekly to identify specific partner and health system needs, coordinate oxygen-related activities, and endorsing key policy documents. The Taskforce represented a critical venue for developing, revising, and championing the National Oxygen Ecosystem Roadmap, and had continued to meet (albeit less frequently) to address ongoing oxygen access challenges.

Stakeholder	Type	Power and Interest
Health Center Management Committees – Central District and Community Hospitals*	Public sector	Have authority within health facility and the catchment areas. Can advocate for more resources to area, but also distribute resources towards oxygen within their budget allocation.
National Ministry of Health – Directorate of Clinical Services*	Public Sector	Responsible for providing leadership in all hospital-based care. Advocates for oxygen needs, can aggregate demand for oxygen, can advocate for more financing towards oxygen allocation.
National Ministry of Health – Directorate of Health Technical Support Services (HTSS)*	Public Sector	Responsible for equipment procurement guidance, maintenance of equipment, service contract management, supply chain of diagnostics and other drugs. Can advocate for more procurement of oxygen equipment.
PATH*	NGO	Technical partner with broader visibility in oxygen ecosystem, expertise of development of roadmaps, costing and developing implementation arrangements. Strong advocacy skills to lobby for more oxygen resources.
FHI 360*	NGO	Supporting MoH in implementation of liquid oxygen in country.
Clinton Health Access Initiative (CHAI)*	NGO	Supporting MoH in implementation of liquid oxygen in country.
Catholic Relief Services (CRS)*	NGO	Supports training of HCWs in COVID-19 case management and oxygen delivery.
Medecins Sans Frontiers*	NGO	Supports in training, testing of COVID and maintenance of medical equipment.
Build Health International*	NGO	Supporting maintenance and training of BMEs in PSA plants.
NEST 360	NGO	Technology partner supporting neonatal care. Donates and trains medical engineers and end users of technologies in patient care.
Right to Care	NGO	Supports donations of oxygen equipment and training of HCW.
BMGF	Donor	Major donor of respiratory care and oxygen interventions.
USAID*	Donor	Major donor of respiratory care and oxygen interventions especially investments in liquid oxygen.
USG	Donor	Major donor of respiratory care and oxygen interventions especially liquid oxygen.
UNICEF*	Donor, multilateral	Supports training of HCW, and supply chain of RCE commodities. Supporting procurement of a PSA plant.
World Bank	Donor	Finance institution that provides loans and grant support for health care goods and services; provided emergency investments during the pandemic to support response efforts.
WHO*	Donor, multilateral	Provides technical support for clinical management and health systems.
Afrox	Private company	Private supplier of medical oxygen. Holds majority share of private market for medical oxygen.
Mission Pharma	Private company	Private supplier of medical equipment and consumables. Offers training, installation, and maintenance/repair services in Malawi.
Pulse Medics Equipment	Private company	Private supplier of Airsep concentrators, Airsep PSA plants, and installers of medical gas piping systems.

Table 5: Oxygen-specific stakeholders in Malawi. *Regular participants in the Malawi Oxygen Taskforce. NGO = non-governmental organization.

Centralized decision-making enabled oxygen champions

Prior to the COVID-19 pandemic, the Malawi Ministry of Health had already been involved in a variety of oxygen and respiratory care-related research, innovations, investments, and projects. For example:

- The Child Lung Health Program, funded by the International Union Against Tuberculosis and Lung Disease and the Bill & Melinda Gates Foundation, standardized inpatient case management for severe forms of pneumonia in children[34] and provided oxygen concentrators to paediatric units in Malawi central and district hospitals between 2002 and 2004.[35]
- The set-up of oxygen for surgical care, with a nationwide implementation of tailored anaesthetic systems was first done in the 1980's and roll-out of pulse oximeters with Lifebox in 2014.[36–38]
- Queen Elizabeth Central Hospital was one of the original partners involved in developing and testing the Pumani bubble CPAP device in 2012.[39] Queen Elizabeth Central Hospital, Kamuzu Central Hospital and the University of Malawi College of Medicine have all led a variety of CPAP research in subsequent years. This research also involved international institutions like Rice University, Baylor University, Johns Hopkins University, University College London, and University Medical Centre Utrecht in the Netherlands.[39–41]
- In 2015, The Paediatric and Child Health Association of Malawi partnered with the MoH to develop an extensive Care of Infants and Newborns training curriculum, which included substantial focus on oxygen.[42]
- The NEST360° partnership has also been operating in Malawi since 2019, introducing a package of newborn respiratory care technologies and supporting pre-service and in-service education for clinicians and biomedical engineers.

These prior investments related to oxygen access ensured that key leaders within the Ministry of Health and clinicians in the largest teaching hospitals were knowledgeable about critical oxygen gaps and needs, had positioned themselves as champions for those needs, and had developed effective working relationships with local and international academic and policy organizations. When the COVID-19 pandemic hit, these champions already had well-established credibility and experience engaging with external partners and donors.

The government's centralized power structure also played a key role in amplifying these champions' voices during the COVID-19 pandemic. As noted above, despite a nominal push for decentralization, de facto decision-making power remains concentrated among a few national-level stakeholders. For oxygen, this core decision-making group included most of the champions from earlier research and investment, and their voices and views carried outsized influence because there were so few other stakeholders involved.

The Oxygen Taskforce, which was established during the pandemic and was critical to organizing the COVID-19 response and passing the Oxygen Ecosystem Roadmap, was a key manifestation of this centralized power structure. The Taskforce served as a central forum to map out and coordinate various partner interventions, and for those partners to identify strategic, mutually beneficial areas of collaboration. For example, PATH, UNICEF, and Partners in Health were able to use the Taskforce to coordinate the launch of both the Oxygen Ecosystem Roadmap and the complementary Emergency and Critical Care Strategy. The Taskforce also served as an accountability mechanism for all stakeholders involved. For example, the Roadmap's implementation framework identified specific actions, responsible organizations, and timelines

for completion that were revisited frequently during Taskforce meetings. Finally, the Taskforce served as a forum for reinforcing and building deep and personal working relationships across key stakeholder organizations, ensuring more effective communication and collaboration.

However, despite its overall effectiveness there are some downsides to Malawi's centralized decision-making structure. While key experts and champions had influential voices, the centralization of power led to a very top-down approach to implementing key interventions, with less-than-ideal involvement of healthcare workers and leaders. For example, although district leaders and district hospital staff are part of the Oxygen Taskforce and were invited to all of the meetings, attendance among sub-national participants was much lower, due in large part to the high workload during the COVID-19 pandemic. As a result, the ideas and concerns of local-level staff may not have been captured reliably for key Taskforce decisions.

Additionally, a centralized power structure for oxygen in Malawi has proven challenging to maintain in a global health landscape, given diverse interests and priorities (often driven by other competing centralized decision structures) from within the Oxygen Taskforce membership. For example, the Oxygen Roadmap did not originally contain plans to invest in liquid oxygen within the first five years, due to a lack of piping and absorptive capacity in health facilities and the lack of a liquid oxygen production facility within Malawi (and the associated supply risks of importing liquid oxygen from a neighbouring country). Nevertheless, liquid oxygen investments are underway, because of available funding through a broader, multi-country liquid oxygen initiative. Such divergent priorities will likely continue to crop up, putting pressure on the government's centralized structure and plans.

Pre-existing data enabled rapid decision-making, but systems are needed for planning

The large body of local oxygen-related research mentioned above also provided the Ministry of Health with a variety of useful data at the outset of the pandemic, and in many cases the Ministry of Health was able to turn that data into quick decisions and actions. For example, clinical research in hypoxemia prevalence impact of oxygen treatments in pneumonia patients, and value of pulse oximetry was critical to helping to advocate for the importance of oxygen, using evidence-based research.[43–46] The fact that Malawian MoH officials comprised many of the research co-authors only improved how quickly the research could be put into action.

In addition, PATH had conducted a biomedical equipment survey for oxygen equipment in January of 2020 (the activity was part of a multi-country effort that was planned prior to the pandemic) and updated that initial data with a rapid assessment in June of 2020.[18] Other equipment-focused assessments included: the Malawi Service Provision Assessments from 2014;[47] NEST360's initial facility evaluations focused on availability of oxygen equipment in district hospital NICUs; and the Malawi Emergency and Critical Care (MECC) survey.[48] Together, these data sources helped the MoH quickly understand where there were supply gaps in oxygen equipment and convey the need to donor agencies. This is reflected in funding applications like the government's application to the Global Fund COVID-19 Response Mechanism.

On the other hand, routine monitoring and reporting systems were *not* already in place, and the government faced challenges in managing data flow and took time to establish reporting systems. One particularly acute challenge arose around tracking equipment donations. Public health facilities could receive donations directly from private parties, and the government did not have a system in place to track what was being donated where. As a result, the MoH could not effectively allocate incoming equipment because it couldn't update its initial equipment gap

assessments with information about new donations. Additionally, for oxygen specifically, there was a high occurrence of well-meaning donors buying and donating industrial oxygen cylinders, which cannot be used in a medical setting. Had there been a proper system in place for coordination and tracking of donations, there could have been stronger messaging on proper cylinder specifications – and could have allowed the MoH to advocate for its oxygen priorities more effectively.

Another data challenge was the continued prevalence of paper-based clinical record systems. Handwritten patient records are difficult to read, aggregate, and analyse in real time. As a result, only basic statistics were recorded and reported to the central level, such as the total number of patient admissions per week or per month. Detailed records about COVID-19 patient outcomes weren't being used to assess what was happening at the patient level. These data and reporting challenges were amplified by shifts in the broader government power and funding structure resulting from the COVID-19 pandemic response. Once a national disaster was declared the Department of Disaster Management Affairs took charge of coordinating pandemic response and routing large financial donations from both the Government and external donor agencies.[49]

A National COVID-19 taskforce was also formed, with each participating Ministry (including the MoH) responsible for developing their own activities to support COVID-19 response. While this was a sensible COVID-19 response framework, it led to new and additional steps in the data and decision-making flow for key leaders in the MoH, especially the technical working groups that were traditionally responsible for things like clinical policy or budgeting/financing. In part due to these new reporting and decision-making structures, it was hard to track and manage spending from donors like the IMF and World Bank,[50] and difficult for MoH leaders to secure typical resources needed to distribute donated or newly purchased goods from the central level to the health facilities.

Catalyzing oxygen investment through policy action

The launch and dissemination of the National Oxygen Roadmap offers important lessons on the use of policy action to stimulate investment. First, as part of the launch process, the MoH planned an extensive advocacy campaign using several dissemination channels:

- Invited the Chair of the Health Committee of Parliament to review the roadmap and give the keynote speech at the Roadmap launch event itself.
- Engaged the World Health Organization, a key dissemination partner in planning the main launch event.
- Presented the Roadmap and costed implementation plan to a consortium of the main health donors in Malawi.
- Engaged news media, including an article printed in Nyasa Times, a prominent local online news platform.
- Travelled to the Districts to disseminate the roadmap and implementation plan; timed visits to coincide with district implementation plans and budget requests; also created template implementation plans to enable their quick completion.
- Included printed copies of the Roadmap with each respiratory care equipment delivery.
- Engaged the Global Fund's principal recipient throughout the roadmap development as part of the oxygen taskforce; scheduled advocacy meetings once there was detailed information on key respiratory care access gaps and fundable activities in the implementation plan.

These launch and dissemination efforts created several advantages in stimulating oxygen investment. First and perhaps most importantly, the specific, delineated implementation plan and associated cost estimates made it easy for partners and donors to identify investment opportunities that aligned with their mission and funding situations. For example, the Global Fund and World Bank saw and responded to the need for additional bulk oxygen production; once the PSA plants they funded are operational, Malawi is expected to sufficient bulk oxygen production to meet the needs estimated in the Roadmap. The Roadmap also helped implementing partners demonstrate to their donors how individual projects mapped back to the overarching goals of improving oxygen access. This approach of using a national strategic plan to direct coordinated action has previous success stories in Malawi.[51]

Additionally, the presence of MoH leaders who can speak to the Roadmap in detail has begun to drive important changes to internal policies and procedures. The PAM Directorate, for example, was able to successfully expand staffing for biomedical engineers to ensure that they would be stationed in all hospitals slated to receive PSA plants in the Roadmap. The Clinical Services Directorate has made significant progress in securing expansion of oxygen services at the health clinic level.

That said, funding for oxygen in Malawi remains a work in progress. As of June 2023, only about 30% of the Roadmap had been fully financed, leading to many concerning gaps. For example, while capital investments such as PSA plant procurements have been covered by the World Bank and Global Fund, the on-going and consumable costs, such as maintenance of those PSA plants is not well funded after their current service contracts expire. Additionally, some oxygen funding went to uses outside of the initial Roadmap implementation plan, in many cases driven by divergent organizational interests. The liquid oxygen example mentioned above, for example, as well as PSA plants installed in smaller facilities not on the Roadmap's list of facilities.

A midline assessment of the Roadmap, will be a key opportunity to track progress toward implementation, and adapt the approach based on evolving priorities, new assessments, and the experiences and lessons described above. The goal of this assessment is to refocus government, donor, and partner attention on the most important gaps and priorities, and in doing so accelerate the path to greater financing for oxygen needs.

Key messages

- Centralised decision-making structures in Malawi benefitted from having oxygen champions that had a history of working in this space – meaning the existing data on hypoxaemia burden and the importance of oxygen were already well known when COVID-19 hit.
- However, central coordinating bodies lacked local representation from districts and were subject to different stakeholder priorities.
- Systems for tracking equipment procurement and distribution are critical and need to include equipment donations that occur outside for formal Government procurement structures. Without this, planning for a national oxygen system is challenging.
- Disseminating the national oxygen roadmap amongst Ministers, District management, donors and the public has raised awareness and led to positive action, but key funding gaps still remain.

Additional methods information

The information in this case study was assembled in two phases – firstly for an internal report conducted by PATH in 2022 which involved academic and grey literature searches and key informant interviews. This was then complemented with a narrow rapid review of academic literature on the political economy of oxygen.

Desk-based review

An initial literature search used Google, Google Scholar, and PubMed to identify relevant academic and grey literature in several categories:

1. Academic literature documenting the long ***history of respiratory care research in Malawi*** by the Ministry of Health, universities, and teaching hospitals. Key search terms included "Malawi" + ["respiratory distress" or "hypoxemia" or "pneumonia" or "respiratory care" or "oxygen" or "oxygen access" or "bubble CPAP" or "ventilation" or "pulse oximetry"].

2. Academic or grey literature focused on the ***COVID-19 pandemic and the government's response effort***, specifically the impact on respiratory care systems. Key search terms included "Malawi" + ["COVID-19" or "coronavirus" or "pandemic"] + ["impact" or "response" or "preparedness"].

3. Academic or grey literature focused specifically on ***analyzing health sector stakeholder influence in Malawi***. Key search terms included "Malawi" + ["health" or "health sector"] + ["influence mapping" or "political economy analysis" or "decentralization"]. Almost all resulting literature focused on political decentralization in Malawi, and its impact on various sectors including healthcare.

4. Official ***government strategy and policy documents*** outlining broad, "on-paper" stakeholder responsibilities.

5. Academic and grey literature ***assessments of individual health sector functions*** in Malawi, such as health financing or procurement. Key search terms included "Malawi" + ["health" or "health sector"] + ["financing" or "procurement" or "supply chain" or "management"]

Key informant interviews

For the stakeholder importance and influence mapping, PATH conducted a series of key informant interviews from January to May of 2022 with administrative and technical leaders whose roles span key aspects of oxygen and medical device management (e.g., supply and distribution, financing, procurement, service delivery, and maintenance). Key informant interviews lasted between 30 minutes and 1 hour, and focused on several general topic areas within each interviewee's domain of expertise:

- What are the typical processes within that domain (e.g., financing, procurement, maintenance) and how do they apply to medical device management?
- Who are the key stakeholders they interact with on a regular basis with respect to medical device management, and what are those interactions like?
- How would they characterize the power dynamics and incentives for each of these key stakeholders?

Position and Organization	Relevance
Chief Procurement Officer, Ministry of Health	Highlight MOH role in medical device procurement
Procurement Officer, Central Medical Stores Trust	Role of CMST in medical device and oxygen procurement; relationship with PAM
Director, JM Diagnostics	Private sector distributor perspective on medical device supply and relationships with public sector stakeholders
Chief Operations Officer, Medical Consultants Africa Ltd	Private sector distributor perspective on medical device supply and relationships with public sector stakeholders
Deputy Director, Physical Assets Management Division, MOH	Detail PAM role in oxygen and medical device financing, procurement, and maintenance
Acting Head, Department of Emergency and Clinical Services, MOH	Illustrate the MOH Pharmacy division's role in oxygen management, and relationship with other MOH entities
Medical Council of Malawi	General overview of medical device stakeholders; role of civil society organizations
Pediatric and Child Health Association	General overview of medical device stakeholders; role of civil society organizations
Chief Economist, Planning Department, MOH	Detail the strategic role of MOH Planning Department; interaction with Ministry of Finance
Principal Economist, Planning Department, MOH	Detail tactical role of MOH Planning Department; interactions with PAM and other operational stakeholders
Principal Economist, Planning Department, MOH	Detail tactical role of MOH Planning Department; interactions with PAM and other operational stakeholders
Regional Management Unit engineer, PAM, MOH	General overview of medical device and oxygen management at subnational level; role of stakeholders in financing and directing device maintenance
Regional Management Unit engineer, PAM, MOH	General overview of medical device and oxygen management at subnational level; role of stakeholders in financing and directing device maintenance
Director of Programs, CHAM	CHAMS offers 40% of health services in Malawi and trains 80% of health workforce.
Marketing Manager, AFROX	Largest/Majority private supplier of medical oxygen in Malawi.

Table 6: Key informant interview participants

Rapid scoping review

A more formal rapid scoping review was also conducted, using a standardized approach across the case studies - the results of the search are presented in the Prisma diagram below.

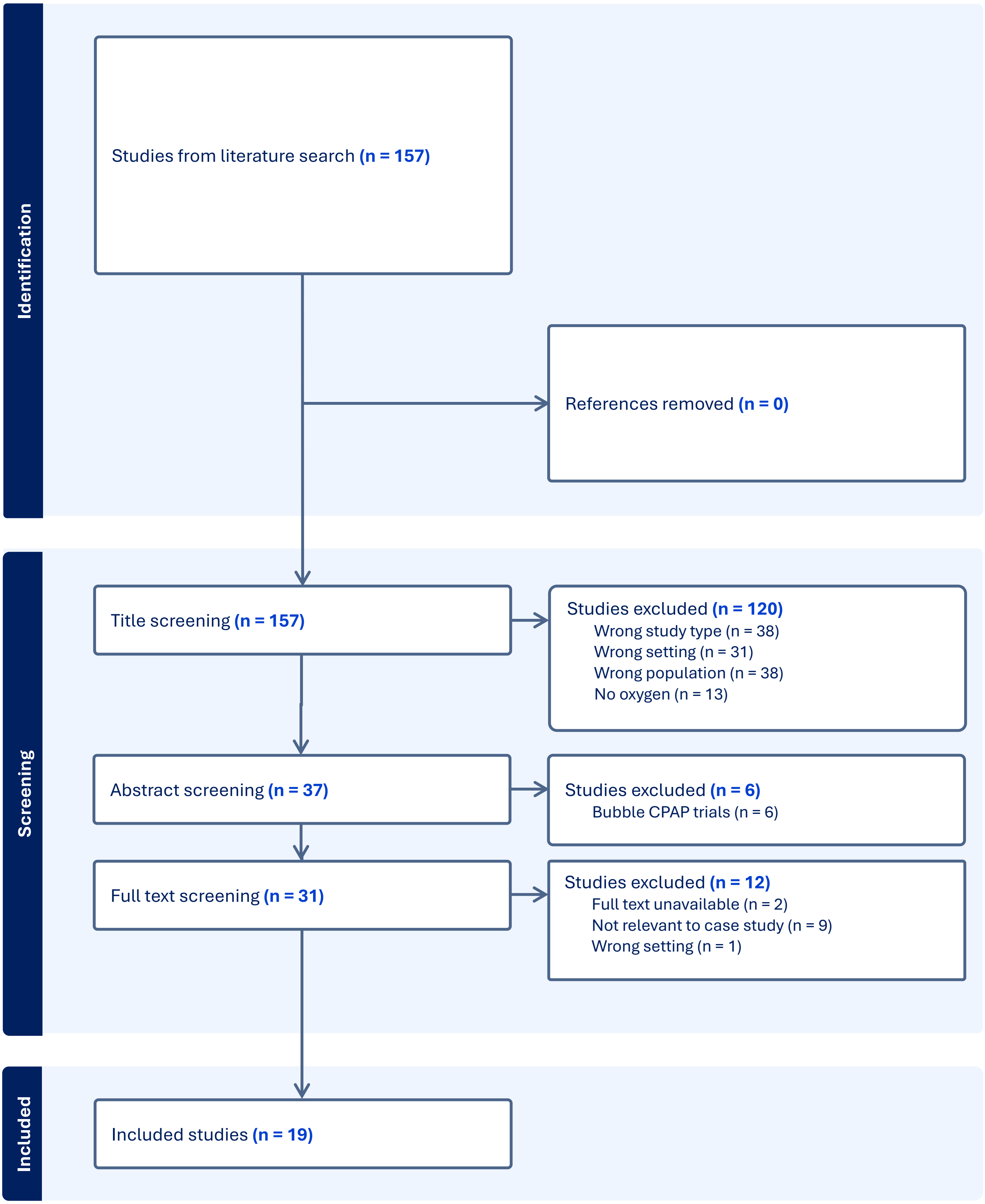

Figure 5: Rapid review literature inclusion

References

1 World Bank. Malawi Country Profile. 2023; published online Dec 26. https://data.worldbank.org/country/malawi (accessed Dec 26, 2023).

2 Malawi National Statistical Office. Malawi Consumer Prices Indices (CPI) Dashboard: November 2023. 2023; published online Dec 26. http://www.nsomalawi.mw/index.php?option=com_content&view=article&id=186&Itemid=37#:~:text=Malawi%20Consumer%20Price%20Indices%20Dashboard&text=The%20year%20on%20year%20inflation,percent%20and%2016.1%20percent%2C%20respectively (accessed Dec 26, 2023).

3 Micah AE, Bhangdia K, Cogswell IE, *et al.* Global investments in pandemic preparedness and COVID-19: development assistance and domestic spending on health between 1990 and 2026. *Lancet Glob Health* 2023; **11**: e385–413.

4 Institute for Health Metrics and Evaluation (IHME). GBD Compare Data Visualization. Seattle, WA: IHME, University of Washington. 2023; published online Dec 26. https://vizhub.healthdata.org/gbd-compare/ (accessed Dec 26, 2023).

5 Nightingale R, Jary H, Meghji J, *et al.* Non-communicable respiratory disease in Malawi: a systematic review and meta-analysis. *Malawi Med J* 2020; **32**: 64–73.

6 Varela C, Young S, Groen R, Banza L, Mkandawire NC, Viste A. Untreated surgical conditions in Malawi: A randomised cross-sectional nationwide household survey. *Malawi Medical Journal* 2017; **29**: 231.

7 Malawi Ministry of Health. Malawi Master Health Facility Registry. 2023. https://documents1.worldbank.org/curated/en/496011611551081262/pdf/Malawi-Master-Health-Facility-List.pdf (accessed Dec 26, 2023).

8 Berman L, Prust ML, Maungena Mononga A, *et al.* Using modeling and scenario analysis to support evidence-based health workforce strategic planning in Malawi. *Hum Resour Health* 2022; **20**: 34.

9 Presidential Taskforce on Coronavirus. The Coronavirus pandemic in Malawi: Trailing the waves. Lilongwe, 2022.

10 Anscombe C, Lissauer S, Thole H, *et al.* A comparison of four epidemic waves of COVID-19 in Malawi; an observational cohort study. *BMC Infect Dis* 2023; **23**: 79.

11 Phiri MM, MacPherson EE, Panulo M, *et al.* Preparedness for and impact of COVID-19 on primary health care delivery in urban and rural Malawi: a mixed methods study. *BMJ Open* 2022; **12**: e051125.

12 Thekkur P, Tweya H, Phiri S, *et al.* Assessing the Impact of COVID-19 on TB and HIV Programme Services in Selected Health Facilities in Lilongwe, Malawi: Operational Research in Real Time. *Trop Med Infect Dis* 2021; **6**: 81.

13 Shapira G, Ahmed T, Drouard SHP, *et al.* Disruptions in maternal and child health service utilization during COVID-19: analysis from eight sub-Saharan African countries. *Health Policy Plan* 2021; **36**: 1140–51.

14 Chimhuya S, Neal SR, Chimhini G, *et al.* Indirect impacts of the COVID-19 pandemic at two tertiary neonatal units in Zimbabwe and Malawi: an interrupted time series analysis. *BMJ Open* 2022; **12**: e048955.

15 Fejfar D, Andom AT, Msuya M, *et al.* The impact of COVID-19 and national pandemic responses on health service utilisation in seven low- and middle-income countries. *Glob Health Action* 2023; **16**: 2178604.

16 Malawi Ministry of Health. Malawi National Medical Oxygen Ecosystem Roadmap 2021-2026. Lilongwe, Malawi, 2021 https://stoppneumonia.org/wp-content/uploads/2022/02/Malawi-National-Medical-Oxygen-Ecosystem-Roadmap-Final-Final.pdf (accessed Dec 26, 2023).

17 UNICEF Office for Innovation. Oxygen System Planning Tool: Demand estimation and recommendations to plan oxygen delivery from source to patient. 2023; published online

Dec 26. https://www.unicef.org/innovation/oxygen-system-planning-tool (accessed Dec 26, 2023).
18 PATH. Biomedical Equipment for COVID-19 Case Management: Malawi Facility Survey Report. Seattle, WA, 2021 https://www.path.org/our-impact/resources/biomedical-equipment-covid-19-case-management-malawi-facility-survey-report/ (accessed Dec 26, 2023).
19 PATH. Malawi National Medical Equipment Baseline Inventory Report 2022. Seattle, WA, 2023 https://www.path.org/our-impact/resources/malawi-national-medical-equipment-baseline-inventory-report/ (accessed Dec 26, 2023).
20 King C, Dube A, Zadutsa B, *et al*. Paediatric Emergency Triage, Assessment and Treatment (ETAT)–preparedness for implementation at primary care facilities in Malawi. *Glob Health Action* 2021; **14**. DOI:10.1080/16549716.2021.1989807.
21 Healthy Newborn Network Website. Malawi-KMC-National-Guidelines, 2009. https://www.google.com/search?q=Healthy+Newborn+Network+Malawi+National+KMC+guidelines&rlz=1C1GCEU_enMW965MW965&oq=Healthy+Newborn+Network+Malawi+National+KMC+guidelines&aqs=chrome..69i57j33i160.23146j0j7&sourceid=chrome&ie=UTF-8 (accessed May 19, 2023).
22 King C, Zadutsa B, Banda L, *et al*. Prospective cohort study of referred Malawian children and their survival by hypoxaemia and hypoglycaemia status. 2022 1;100(5):302-314. DOI: 10.2471/BLT.21.287265
23 Organization WH. Oxygen therapy for children: a manual for health workers. Geneva: World Health Organization, 2016 https://apps.who.int/iris/handle/10665/204584.
24 UN Inter-Agency Group for Child Mortality Estimation(IGME) website. Malawi Under Five mortality rate. 2021.
25 McCollum ED, Bjornstad E, Preidis GA, Hosseinipour MC, Lufesi N. Multicenter study of hypoxemia prevalence and quality of oxygen treatment for hospitalized Malawian children. *Trans R Soc Trop Med Hyg* 2013; **107**: 285–92.
26 Hooli S, Makwenda C, Lufesi N, *et al*. Implication of the 2014 World Health Organization Integrated Management of Childhood Illness Pneumonia Guidelines with and without pulse oximetry use in Malawi: A retrospective cohort study. *Gates Open Res* 2023; **7**: 71.
27 Kayambankadzanja RK, Schell CO, Mbingwani I, Mndolo SK, Castegren M, Baker T. Unmet need of essential treatments for critical illness in Malawi. *PLoS One* 2021; **16**: e0256361.
28 Njoroge MW, Mjojo P, Chirwa C, *et al*. Changing lung function and associated health-related quality-of-life: A five-year cohort study of Malawian adults. *EClinicalMedicine* 2021; **41**: 101166.
29 Mulupi S, Ayakaka I, Tolhurst R, *et al*. What are the barriers to the diagnosis and management of chronic respiratory disease in sub-Saharan Africa? A qualitative study with healthcare workers, national and regional policy stakeholders in five countries. *BMJ Open* 2022; **12**: e052105.
30 Masefield SC, Msosa A, Grugel J. Challenges to effective governance in a low income healthcare system: a qualitative study of stakeholder perceptions in Malawi. *BMC Health Serv Res* 2020; **20**: 1142.
31 OECD, United Cities and Local Governments (UCLG). OECD Regional Country Profiles - Malawi. 2016; published online Oct. https://www.oecd.org/regional/regional-policy/profile-Malawi.pdf (accessed Dec 26, 2023).
32 O'Neil T, Cammack D, Kanyongolo E, *et al*. Fragmented governance and local service delivery in Malawi. London, UK, 2014 https://cdn.odi.org/media/documents/8943.pdf (accessed Dec 26, 2023).
33 Rodríguez DC, Balaji LN, Chamdimba E, *et al*. Political economy analysis of subnational health management in Kenya, Malawi and Uganda. *Health Policy Plan* 2023; **38**: 631–47.

34 Lazzerini M, Sonego M, Pellegrin MC. Hypoxaemia as a Mortality Risk Factor in Acute Lower Respiratory Infections in Children in Low and Middle-Income Countries: Systematic Review and Meta-Analysis. *PLoS One* 2015; **10**: e0136166.
35 Enarson P, La Vincente S, Gie R, Maganga E, Chokani C. Implementation of an oxygen concentrator system in district hospital paediatric wards throughout Malawi. *Bull World Health Organ* 2008; **86**: 344–8.
36 Scott DA, McDougall R. The effective introduction of Lifebox pulse oximetry to Malawi. *Anaesthesia* 2017; **72**: 675–7.
37 Albert V, Mndolo S, Harrison EM, O'Sullivan E, Wilson IH, Walker IA. Lifebox pulse oximeter implementation in Malawi: evaluation of educational outcomes and impact on oxygen desaturation episodes during anaesthesia. *Anaesthesia* 2017; **72**: 686–93.
38 Pedersen J, Nyrop M. Anaesthetic equipment for a developing country. *Br J Anaesth* 1991; **66**: 264–70.
39 Brown J, Machen H, Kawaza K, *et al*. A High-Value, Low-Cost Bubble Continuous Positive Airway Pressure System for Low-Resource Settings: Technical Assessment and Initial Case Reports. *PLoS One* 2013; **8**: e53622.
40 Walk J, Dinga P, Banda C, *et al*. Non-invasive ventilation with bubble CPAP is feasible and improves respiratory physiology in hospitalised Malawian children with acute respiratory failure. *Paediatr Int Child Health* 2016; **36**: 28–33.
41 McCollum ED, Mvalo T, Eckerle M, *et al*. Bubble continuous positive airway pressure for children with high-risk conditions and severe pneumonia in Malawi: an open label, randomised, controlled trial. *Lancet Respir Med* 2019; **7**: 964–74.
42 O'Hare B, Kawaza K, Mzikamanda R, Molyneux L. 2016 Care of the infant and newborn in Malawi: the COIN course participants manual. St Andrews, UK: University of St. Andrews, 2017 http://misssophie.net/guidelines/data/set_1010/html/_chapters.htm (accessed Dec 26, 2023).
43 Hooli S, King C, Zadutsa B, *et al*. The epidemiology of hypoxemic pneumonia among young infants in Malawi. *American Journal of Tropical Medicine and Hygiene* 2020; **102**. DOI:10.4269/ajtmh.19-0516.
44 McCollum ED, King C, Deula R, *et al*. Pulse oximetry for children with pneumonia treated as outpatients in rural Malawi. *Bull World Health Organ* 2016; **94**. DOI:10.2471/BLT.16.173401.
45 King C, Zadutsa B, Banda L, *et al*. Prospective cohort study of referred Malawian children and their survival by hypoxaemia and hypoglycaemia status. *Bull World Health Organ* 2022; **100**. DOI:10.2471/BLT.21.287265.
46 Sylvies F, Nyirenda L, Blair A, Baltzell K. The impact of pulse oximetry and Integrated Management of Childhood Illness (IMCI) training on antibiotic prescribing practices in rural Malawi: A mixed-methods study. *PLoS One* 2020; **15**: e0242440.
47 Malawi Ministry of Health, ICF International. Malawi Service Provision Assessment (MSPA) 2013-14. Lilongwe, Malawi and Rockville, Maryland, USA, 2014 https://dhsprogram.com/pubs/pdf/SPA20/SPA20%5BOct-7-2015%5D.pdf (accessed Dec 26, 2023).
48 Sonenthal PD, Masiye J, Kasomekera N, *et al*. COVID-19 preparedness in Malawi: a national facility-based critical care assessment. *Lancet Glob Health* 2020; **8**: e890–2.
49 Mzumara GW, Chawani M, Sakala M, *et al*. The health policy response to COVID-19 in Malawi. *BMJ Glob Health* 2021; **6**. DOI:10.1136/bmjgh-2021-006035.
50 Nayupe SF, Munharo S, Mbulaje P, Banda C, Lucero-Prisno DE. Covid-19 and fund mismanagement in Malawi: A major challenge to its effective pandemic containment. *Health Sci Rep* 2022; **5**: e546.
51 Yoon I, Twea P, Heung S, *et al*. Health Sector Resource Mapping in Malawi: Sharing the Collection and Use of Budget Data for Evidence-Based Decision Making. *Glob Health Sci Pract* 2021; **9**: 793–803.

THE LANCET Global Health
COMMISSION ON MEDICAL OXYGEN SECURITY

Country Case Study: Nigeria

From national policy to local implementation

Adegoke G. Falade,[1,2] *Abiodun A Sogbesan,*[2,3] *Ayobami A Bakare,*[3,4] *Adejumoke I Ayede,*[1,2] *Carina King*[3]

1. Department of Paediatrics, College of Medicine, University of Ibadan, Nigeria; **2**. Department of Paediatrics, University College Hospital, Nigeria; **3**. Department of Global Public Health, Karolinska Institutet, Sweden; **4**. Department of Community Medicine, University College Hospital, Nigeria.

Case study focus

The Nigeria government was one of the first countries to publish a national strategy for oxygen. In 2016, the Federal Ministry of Health, collaborated with stakeholders to develop the National Policy on Medical Oxygen, and subsequently the National Strategy for the Scale-up of Medical Oxygen in Health Facilities 2017-2022. The National Policy on Medical Oxygen laid the foundation for the national strategy, emphasizing a comprehensive approach to managing patients and improving access to life-saving oxygen in Nigeria. The comprehensive 5-year roadmap, aimed to address the high morbidity and mortality from hypoxaemia among children in Nigeria, and serves as a roadmap for engaging stakeholders across the medical oxygen ecosystem, including oxygen generation, distribution, administration, and maintenance, to guide increased access to medical oxygen supply systems in Nigeria. This case study focuses on understanding the political environment surrounding medical oxygen in Nigeria, assessing the extent to which national-level Federal Government policies have been adopted and implemented at State and local levels across Nigeria. We explore enabling factors contributing to effective policy enactment, and challenges that have limited uptake. This is particularly crucial when contextualized within the complex framework of the Nigeria Health System, a mixed system of public, private and donor-funded delivery through primary, secondary and tertiary health facilities.

Country Context

Demography, economy and epidemiology

Indicator	Value in 2022/2023	Data source
Total population	223.8 million	United Nations Population Fund, 2022[1]<br>UNICEF, 2023[2]
Total under-five population	35.9 million	UNICEF, 2023[2]
Under-five mortality	102 per 1,000 live births<br><br>111 per 1,000 live births	National Bureau of Statistics, 2022[3]<br>UNICEF, 2023[2]
Life expectancy (m:f)	68 years: 78 years	United Nations Population Fund, 2022[1]
GDP	$US 2280 per capita in Q1 2023 3	International Monetary Fund, 2023[4]
Healthcare expenditure	$US 70 per capita	The World Bank, 2023[5]
Income status	Lower-middle income	The World Bank 2023[6]
Neonatal mortality rate	34 per 1,000 live births	National Bureau of Statistics, 2022[3]

Table 1: Key metrics and indicators for Nigeria

Approximately 40% of Nigerians live below the national poverty line of $382 per year, and 31% living below the international extreme poverty line of $2.15 per person per day.[7] Economic inequalities exist in Nigeria, with poverty clustered in the Northern States and in rural areas of the country. Of those living below the international poverty line, 79% are living in Northern States and 87% live in rural areas.[7] The top causes of mortality across all ages in Nigeria are neonatal conditions, acute lower respiratory infections, diarrhoeal diseases, tuberculosis, and malaria.[8] The three leading causes of death in children under-five in Nigeria are malaria, diarrhoea, and pneumonia,[9] responsible for 64% of mortality in this group.[2] Nigeria is not on track to achieve SDG 3.2 by 2030 (Figure 1).[10]

Hypoxaemia contributes to over a million preventable deaths in low-income countries every year.[11] In Nigeria, an estimated 625,000 deaths occur annually from diseases associated with hypoxaemia,[12] however, reliable data about the hypoxaemia burden and oxygen need for different disease conditions is largely lacking. Notably, hypoxaemia is prevalent among hospitalized children in Nigeria, increasing the odds of death by 6-fold in neonates and 8-fold in under-five children.[13] Approximately 13 million persons in Nigeria, including children, adolescents and adults, have clinical asthma,[14] and evidence show a low rate of oxygen saturation monitoring at admission and during the course of treatment for patients with acute asthma in Nigeria. Specifically, only 5% of patients with acute asthma have an SpO_2 measured upon admission, and while supplemental oxygen was administered to 31% of them, none of these decisions were informed by an oxygen saturation measurement.[15] A study in 2012 indicated that 57% of endoscopists in Nigeria monitor oxygen saturation during sedation, with half (51%) utilizing supplemental oxygen for diagnostic gastrointestinal endoscopy.[16] For chronic obstructive pulmonary diseases (COPD), a review by Ale *et al.* (2022) provided the first national estimates for the prevalence of COPD in Nigeria, reporting a median prevalence of 9.2% (interquartile range, IQR=7.6–10.0).[17] However, the contribution of COPD to burden of hypoxaemia in Nigeria has not been determined.

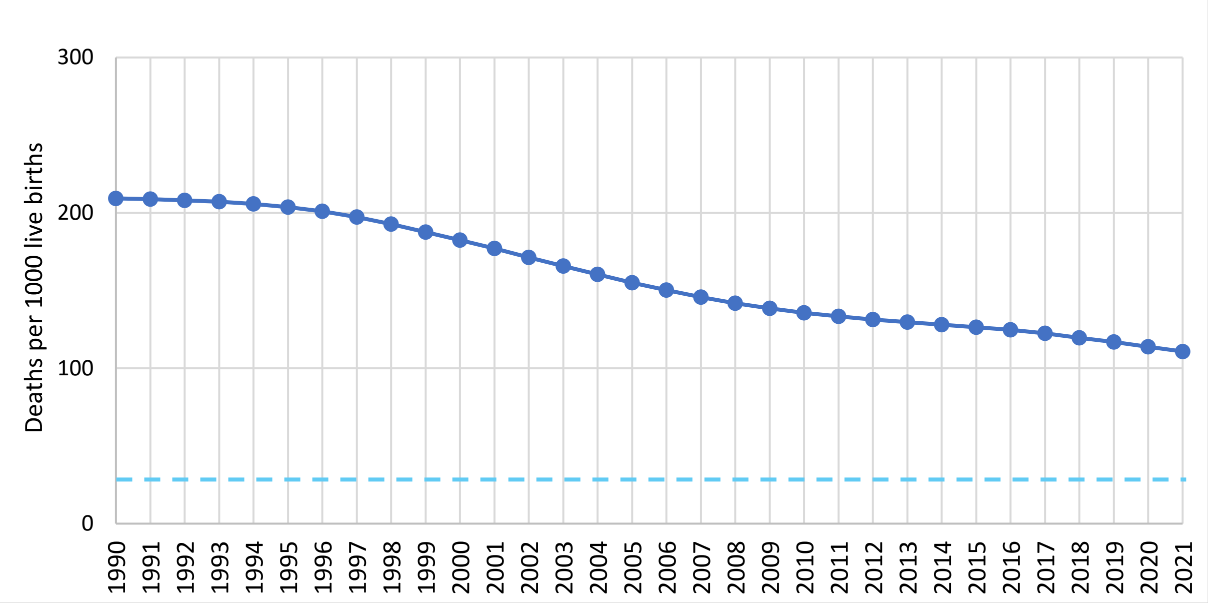

Figure 1: Trend in the under-five mortality rate in Nigeria. Source: UNICEF Data (2023): Monitoring the situation of children and women - Nigeria. https://data.unicef.org/country/nga/

Health system

The Nigerian healthcare system is public sector driven, with a substantial private sector involvement in service provision.[18] The Nigerian healthcare system operates with tertiary, secondary and primary levels of care. Secondary- and tertiary-level health facilities are predominant in urban areas, while primary healthcare (PHC) facilities mostly serve rural areas. The Federal government has a responsibility to organize tertiary health services through the network of teaching hospitals and federal medical centres while, the State government is primarily responsible for secondary health services but may also provide tertiary services when financial capacity is available. The local government provides primary health services through health posts and clinics, primary health centres and comprehensive health centres – Figure 2. Patients are referred from the primary health care which is the first entry point of care to other higher levels of care.[18]

Private health facilities are classified based on their structure and the services they provide.[19] There is no regulation that appropriately classifies which level of healthcare private facilities belong as structures and designs of private facilities vary across settings. The private healthcare providers in Nigeria are broadly clinics, maternity homes, and hospitals with ownership including individuals, faith-based and other civil society organisation.[19] The Department of Hospital Services of the Federal Ministry of Health coordinates all interventions for medical oxygen and medical oxygen systems in the country, providing strategic guidance to government, partners, and stakeholders in the oxygen landscape.[20]

There are around 38,500 operational health facilities in Nigeria, including hospitals and clinics enumerated by the Federal Ministry of Health. Of these health facilities 0.4% are tertiary hospitals, 14% are secondary facilities, and 85% are primary facilities. The majority of the health facilities (74%) are publicly owned.[21] Despite having the highest proportion of health workers in Africa, accounting for 26% (0.94 million) of the continents workforce, Nigeria's health workforce density of 1.95 per 1000 population is still below the WHO threshold of 4.45 health workers per

1000 population.[22,23] There are 39 doctors to 100,000 population (0.4 doctors per 1000 population) and 148 nurse/midwife to 100,000 population in Nigeria (1.5 nurses and midwives per 1000 population).[24] However, the human resource for health in Nigeria has seen significant reduction recently with migration of healthcare workers to high income countries.

Health financing comes from several sources including, but not limited to, tax revenue, out-of-pocket payments, donor funding, and health insurance (social and community).[25] However, out-of-pocket expenditure accounts for 78% of total health expenditure in the country, with high risk of catastrophic and impoverishing health expenditure for many households – especially for the large number in the population with no insurance packages.[24,26] Health insurance covers less than 5% of the population,[26] with these individuals primarily covered through health insurance from their employers; privately purchased insurance is uncommon.[27]

The low budget for health by the Nigerian government has been a major challenge in achieving universal health coverage. This is reflected in the failure to achieve the 2001 Abuja Declaration where African leaders pledged a commitment to increase budgetary allocation to 15%.[28,29] Competing programmes or activities have been given as a reason for not meeting this target. The situation is worse at the State and local government levels, where even less is allocated to health. Since the COVID-19 pandemic, the 36 State governments responded by increasing investment towards health. Between 2020 and 2022, only Kaduna and Sokoto states consistently achieved the target of 15% health expenditure, and 23 states failed to reach the target even once during this period, despite increasing their nominal spending on health.[30] In the fiscal years 2021 and 2022, 13 States (Akwa Ibom, Anambra, Benue, Edo, Enugu, Jigawa, Kano, Katsina, Kebbi, Lagos, Niger, Rivers, and Yobe) consistently increased their budgetary allocation for health; Yobe, Imo, and Bayelsa States more than doubled their 2020 budgetary levels in 2022.[30] Nevertheless, despite documented poor health outcomes thirteen State governments, notably Ebonyi and Plateau State, decreased fiscal allocations to the healthcare sector in 2022.[30]

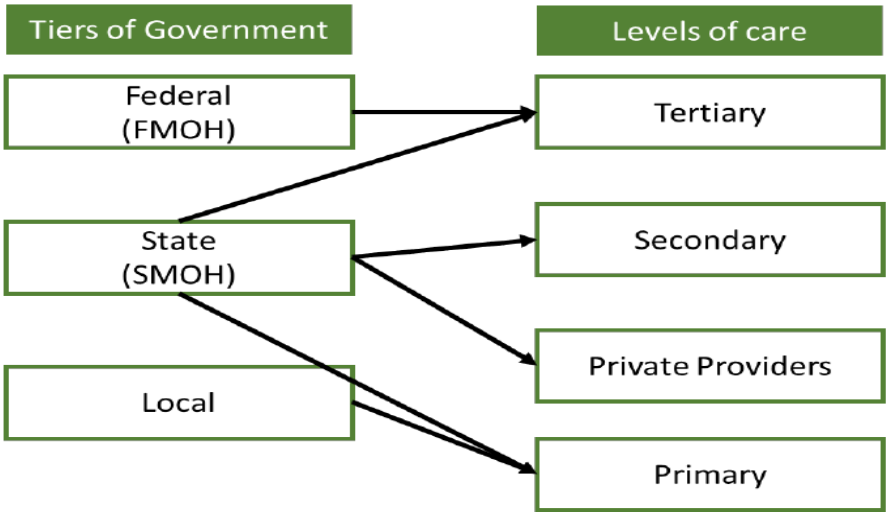

Figure 2: Health system structure in Nigeria. Source: National Strategy for the Scale-up of Medical Oxygen in Health Facilities 2023-2027.

During the COVID-19 pandemic, the Nigerian government officially enacted the new National Health Insurance Act (NHIA) 2022 on May 19, 2022 which expands coverage to over 83 million poor and vulnerable people.[31,32] The NHIA Act was signed into bill to ensure universal health coverage with health insurance now mandatory for all Nigerians by the government.[31] On 1 August 2022, the WHO Country Representative announced their pledge to aid Nigeria in accelerating the operationalization of the NHIA Act including the Vulnerable Group Fund (VGF) through provision of sustainable health financing support for universal health coverage and health security.[32] They reiterated that the support to be provided is in line with WHO's goal of making sure that all individuals and communities receive the quality health services they need without suffering financial hardship.[32]

Under the past administration of President Muhammed Buhari's government, budgetary allocation for health in Nigeria surpassed a trillion naira for the first time in 2023, constituting 5.8% of the 2023 total budget.[28] In the subsequent term under the leadership of President Bola Tinubu, the 2024 health budget was set to ₦1.5 trillion (approximately US $2 billion), representing 5.5% of the total budget. Despite the increase in total amount from 2023, the value of the 2024 budget is lower in real-terms due to changes in the official Naira exchange rate, a 28% year-on-year inflation rate, and population growth.[33] Furthermore, the 2024 budget continues to fall short of the Abuja declaration, representing only a third of the recommended 15% of the total budget which would have amounted to ₦4.1 trillion.[28,33] On January 11, 2024, the Federal Government revised the foreign exchange benchmark to ₦800 per dollar in the 2024 budget, considering the Naira's average performance and global dynamics.[34] Consequently, the 2024 health budget was revised down to represent US $1.9 billion. The incumbent President Bola Tinubu-led administration has expressed its commitment to increasing the annual budgetary allocation to the health sector to 10% of the country's total budget. However, this commitment is yet to be reflected in the first national budget set by the administration, with promises of additional increments contingent on judicious management.[28]

COVID-19

As of the 26th July 2023, a total of 266,675 cases of COVID-19 had been confirmed in Nigeria and 3,155 deaths recorded.[35] Nigeria received the first shipment of 3.9 million doses of the Oxford-AstraZeneca vaccine through the COVAX facility in March 2021.[37,38] In response to the COVID-19 pandemic, the Coalition Against COVID-19 (CACOVID), a private sector-led organization in collaboration with the Federal Government, the Nigeria Centre for Disease Control (NCDC) and the WHO was launched on March 26, 2020. The coalition aimed to bolster the government's response and address the challenges posed by the spread of COVID-19 in Nigeria.[39,40] CACOVID's mandate included pooling resources from various industries to offer technical and operational support, as well as providing financial assistance and fostering advocacy through robust awareness campaigns.

Playing a pivotal role in enhancing the country's healthcare infrastructure, CACOVID strategically established medical facilities, including testing, isolation, and treatment centres, equipped with Intensive Care Units (ICUs) and molecular testing laboratories. This was implemented across all six geopolitical zones in Nigeria to strengthen the nation's ability to manage and respond to the challenges posed by the COVID-19 pandemic.[39] This initiative earned the coalition recognition as the third-largest contributor globally to the fight against COVID-19 virus. By the time the Coalition concluded its efforts in 2023, it had mobilised 62 billion naira, established 39 isolation centres in all 36 States and the FCT of Abuja, testing

supplies for almost one million tests, food for 10 million vulnerable individuals, oxygen and oxygen cylinders for the most affected states, support for vaccines delivery and distribution, and assistance in reopening the economy during lockdown.

However, the pandemic negatively affected the healthcare system, particularly in the area of medical oxygen supply. Health facilities in Nigeria that were grappling with pre-existing deficiencies in their oxygen systems were further burdened – Table 2. The Federal Ministry of Health, led by the Minister of Health, recognized this critical need and took proactive measures. These included repairing non-functional Pressure Swing Adsorption (PSA) plants nationwide and collaborating with industrial oxygen producers to repurpose their facilities for the production of medical-grade oxygen, aiming to bolster the country's oxygen production capacity.[42]

However, research findings paint a picture of inadequate oxygen access. Findings from public primary and secondary facilities, and private facilities in Lagos, Nigeria, found none of the facilities were equipped to meet minimum oxygen demands.[41] The demand for medical oxygen increased by seven-fold in Lagos Stata, the epicentre of the pandemic, and hospitals in the capital city of Abuja were on the brink of running out of supplemental oxygen during the second wave.[43] Tertiary hospitals, such as University of Nigeria Teaching Hospital Enugu, Southeastern Nigeria and Lagos University Teaching Hospital, reported acute shortages of oxygen at some points during the pandemic.[44] According to the Chief Medical Director of Lagos University Teaching Hospital, several months after the pandemic began, the PSA plant at their facility was upgraded on request; however, the facility used over 120 oxygen cylinders a day and oxygen demand overwhelmed their PSA plant. He stressed that patients in the wards required a high flow of oxygen, and all admitted patients needed oxygen, meaning cylinders often ran out.[44]

Anecdotal evidence from COVID-19 patients highlighted the immense struggle healthcare workers faced in managing patients in public hospitals, due to the scarcity of oxygen.[43] Wealthier COVID-19 patients sought treatment in private hospitals where medical oxygen was more readily available but at a higher cost.[43,44] The national health insurance package did not cover the management of COVID-19, making it difficult for the working poor to afford the unexpected costs of testing and treatment at private facilities.[45] On 6th August 2022, UNICEF announced an 18-month partnership with IHS Nigeria – a private company specializing in telecommunication infrastructure, to support the Federal Ministry of Health in strengthening oxygen supply in hospitals across eight States in Nigeria citing the potential consequence of limited access to medical oxygen for critically ill patients with severe COVID-19 and pneumonia.[46]

Table 2 presents findings on the availability of oxygen and/or pulse oximetry in various clinical settings before and during the pandemic, with limited access and infrastructural gaps evident, especially in rural and paediatric care settings. In addition, there were challenges in continuous monitoring and cost barriers affecting quality of care before pandemic. During the pandemic, published data shows interventions and stakeholder awareness initiatives improved oxygen access in paediatric care; however, challenges in oxygen access persisted, cost remained a barrier to treatment, and ongoing efforts are crucial to address disparities and ensure sustained enhancements in healthcare infrastructure. The pandemic underscored the importance of adaptive interventions and health system strengthening to meet the evolving demands for oxygen therapy.

Authors	Patient/health workers group	Setting	Key findings
		Pre COVID-19	
Mokuolu and Ajayi (2002)	Neonates	Neonatal unit	Oxygen concentrators were recommended as cost-effective for neonatal units in Nigeria. Demonstrated significant cost savings with concentrators compared to cylinder.[51]
Desalu *et al.* (2011)	Asthma patients	Tertiary hospitals	Limited availability of pulse oximeters in teaching hospitals in Nigeria.[52]
Ogunbosi *et al.* (2011)	Children under 15 years	Paediatric emergency room at a tertiary hospital	About 32.2% of children received oxygen therapy in the emergency room.[53]
Orimadegun *et al.* (2011)	Paediatricians	Healthcare centres in Nigeria	Limited knowledge and access to pulse oximeters among paediatricians.[54]
Nwokediuko and Obienu (2012)	GI endoscopists	Scientific conference	Only 57.1% of endoscopists monitored oxygen saturation during sedation.[16]
Henry et al. (2012)	Surgeons	41 private, rural hospitals in southern Nigeria	44.5% of private, rural hospitals had a pulse oximeter.[55]
Abdulraheem *et al.* (2015)	Neonates referred and transported to facility	Tertiary hospital	Study on neonates revealed 66.2% had hypoxemia, and failure to administer oxygen during transport was associated with hypoxemia.[56]
Desalu *et al.* (2016)	Patients with acute exacerbation of asthma	Two tertiary hospitals in a state in Southwestern Nigeria	Inadequate measurement of oxygen saturation and limited use of continuous monitoring in asthma care.[15]
Iroezindu *et al.* (2016)	Adults with community-acquired pneumonia	Four major tertiary care hospitals in South-East Nigeria	Need for supplemental oxygen identified as an independent predictor of in-hospital mortality among adult patients with pneumonia.[57]
Graham *et al.* (2016)	Children and newborns	Non-tertiary hospitals in south-west Nigeria	Structural, technical, and clinical barriers to safe and effective oxygen therapy were identified in non-tertiary hospitals.[58]
CHAI (2017)	Patients in health facilities in Nigeria, with a specific focus	Hospitals and paediatric wards in Nigeria.	Oxygen and pulse oximeter availability in health facilities were assessed. Only 55% of 169 hospitals provided oxygen therapy,

	on paediatric wards.		with only 17% in paediatric wards. Pulse oximeters were available in 11% of hospitals. In the emergency paediatric unit of Barau Dikko Teaching Hospital, 80% percent of the concentrators were initially found to deliver oxygen at very low concentrations. After routine maintenance and repair by trained engineers, all concentrators were reported to be working optimally.[59]
Aneji *et al.* (2020)	HCW and hospital administrators	Hospitals involved in the bCPAP program	Survey of hospitals indicated that only 64.3% reported the presence of pulse oximeters.[61]
King *et al.* (2020)	Stakeholders involved in paediatric pneumonia	National, Jigawa and Lagos states	Stakeholders identified insufficient and inadequate access to essential equipment, including oxygen and pulse oximeters, in paediatric pneumonia management.[60]
During COVID-19			
Bakare *et al.* (2020)	Children and neonates	12 Secondary health facilities in southwest Nigeria	Cross-sectional facility assessment revealed varied availability and functionality of oxygen equipment in secondary health facilities.[62]
Briggs and Eneh (2020)	HCWs providing obstetrics and newborn services	28 PHC Centres in Port Harcourt, South-South Nigeria.	Only 5 out of 28 primary health care centres in Port Harcourt, Nigeria, had oxygen cylinders, and none had oxygen readily available for use.[63]
Walker *et al.* (2020)	Neonates	Three hospitals in southwest Nigeria	Prospective study on the effectiveness of intermittent pulse oximetry in guiding oxygen therapy in neonates in a low-resource setting.[64]
Banke-Thomas *et al.* (2021)	Pregnant women with COVID-19	Tertiary hospital in Lagos, Southwestern Nigeria	Hospital-based cost analysis revealed medical oxygen as a major cost driver for managing severe COVID-19 symptoms.[65]
Graham *et al.* (2021)	Children and neonates	58 health facilities in Lagos state including primary, secondary, government, and	Case study on oxygen access in health facilities in Lagos state, Nigeria, revealed disparities in availability, cost, and use.[66]

		private health facilities	
Graham *et al.* (2021)	Children with severe pneumonia, severe malaria, and severe diarrhoea	12 hospitals in South-west Nigeria	Stepped-wedge cluster randomized trial showed improved quality of care for children with severe pneumonia and severe malaria with enhanced oxygen systems.[67]
Ogunbiyi *et al.* (2021)	Children and adults	30 public and private intensive care units	Cross-sectional survey of 30 public and private intensive care units in 6 geo-political zones of Nigeria revealed varying costs for ICU admission and challenges in equipment functional.[68]
Bolu *et al.* (2022)	Children and adults	National end-of-year review of findings of the Presidential Task Force on Health	End-of-year review of Nigeria's response to the COVID-19 pandemic, highlighting the challenges and improvements in healthcare infrastructure.[69]
Graham *et al.* (2022)	Children and neonates	12 secondary health facilities in four states (Oyo, Ondo, Osun, and Ogun) in south-west Nigeria.	Prospective evaluation of the sustainability and effectiveness of improved oxygen systems in secondary health facilities in southwestern Nigeria.[70]
Kalu *et al.* (2022)	Children and adults	205 health care facilities in 18 LGAs, across the three senatorial districts of Cross River State	Descriptive cross-sectional study on the availability of Basic Life Support devices and essential drugs in health care facilities in Cross River State, Nigeria.[71]
Okeke *et al.* (2022)	HCWs	Literature review of responses to COVID-19 in Nigerian health facilities	Rapid assessment of the health system's response to COVID-19 in Nigeria, highlighting the training of healthcare workers and acquisition of ventilators and oxygen concentrators.[72]
CHAI (2023)	Biomedical engineers and HCWs involved in oxygen equipment	Assessment in health facilities across five Nigerian states	CHAI collaborated with LUTH for national training of biomedical engineers, resulting in peer-led mentoring programs for equipment maintenance.[73]

Table 2: Evidence of the presence or absence of oxygen/oximetry in various clinical settings. HCW = healthcare workers

Oxygen supply and clinical use landscape in Nigeria

The findings from the first comprehensive health facility assessment for medical oxygen was conducted across all 36 states and the Federal Capital Territory between June and September 2022. It was conducted by the Federal MOH supported by FHI360, UNICEF and Clinton Health Access Initiative (CHAI) and included 6,786 health facilities, comprising primary health centres, secondary and tertiary health facilities, specialized hospitals and private hospitals. The assessment identified 39 PSA plants in secondary and tertiary facilities in Nigeria, of which 30 (77%) were functional (22 in tertiary hospitals, 8 in secondary facilities). While additional PSA plants exist in the country, ownership lies within the private sector, which operates various types of public private partnership (PPP) models with health facilities and often operate hub-and-spoke models to support adjacent health facilities. Examples of PSA plants supplying through PPP models include: the PSA plant provided by CHAI at the Infectious Disease Hospital in Lagos; the Life Bank PSA plant in Orozo LGA, Nassarawa state, installed in 2021 through a PSA plant franchising model; Healthport's solar powered micro-PSA plant installed and operational in the 50-bed hospital, Harvey Health Centre and Maternity in Yaba, Lagos.

The COVID-19 pandemic presented an opportunity to acquire an additional 122 PSA plants, with plans for installation spanning across the six geopolitical zones. The procurement of these plants holds significant importance, given that many regions in the country lack access to private sector supply channels. Notably, the Federal Government of Nigeria procured 38 of these plants to ensure coverage across all 36 states and FCT (with two plants for Lagos state). Additionally, 9 plants were obtained through UNICEF, and another 75 via the Global Fund COVID-19 response mechanism (C19-RM). As of early 2024, 12 of the 75 C19-RM PSA plants were installed.[78]

Oxygen cylinders and oxygen concentrators at the bedside are the most common oxygen sources in most health facilities in Nigeria. Some larger secondary and tertiary health facilities have bedside oxygen piping systems and on-site PSA plants of varying capacities and efficiency. Unfortunately, many have become redundant and inefficient due to poor maintenance practices. The 2022 national assessment identified a total of 5,741 oxygen concentrators, of which 76% were functional, and 49% had a 5L/minute maximum capacity. Comparing these findings with previous smaller assessments, a relatively consistent trend in concentrator functionality emerges, with 72% functionality reported in 12 secondary facilities across four states (Oyo, Ondo, Osun and Ogun),[70] and 64% in 58 health facilities (28 private and 30 public facilities) in Lagos.[41] Both National and the smaller assessments highlighted maintenance issues, broken concentrators and retired and broken cylinders.

For liquid oxygen (LOX), Industrial and Medical Gases (IMG) and Air Liquide are the key suppliers, concentrated in the southern region of the country. These suppliers also produce other industrial gases for private businesses including bottling companies, mining and oil sectors. The national assessment found only two facilities with functional LOX storage capacity: the National Hospital Abuja and Jos University Teaching Hospital, with a total storage capacity of 18,000L of oxygen. Facilities operating the LOX system perennially experience refilling challenges with an estimated delay of 3-4 weeks between order and delivery. With support from the Global Fund C19-RM, USAID and FHI360, the country is enhancing its LOX capacity. This involves acquiring an additional 18 LOX tanks (12 funded through the C19-RM and 6 through USAID). This expansion will boost the storage capacity 10-times, up to approximately 188,800 litres, with two tanks located in each of the six geopolitical zones.

There were 8,824 pulse oximeters in the National assessment, averaging approximately 1 pulse oximeter per facility, with a higher concentration in secondary and tertiary facilities. Overall, 77% of these assessed pulse oximeters were found to be functional, and 51% of the functional pulse oximeters were fingertip pulse oximeters (Table 3). A comparison with previous smaller assessments suggests a higher overall functionality rate for pulse oximeters in the national survey,[41,62] but similar percentage of fingertip pulse oximeters.[41]

Type	Functional	Total	Health facilities				
			Primary	Secondary	Tertiary	Specialized hospitals	Private
Table-top	Functional	1431 (76%)	309 (64%)	697 (81%)	388 (77%)	0 (0%)	37 (79%)
	Non-functional	460 (24%)	175 (36%)	161 (19%)	114 (23%)	0 (0%)	10 (21%)
Hand-held	Functional	1728 (70%)	291 (40%)	1012 (85%)	369 (78%)	1 (100%)	55 (82%)
	Non-functional	725 (30%)	429 (60%)	182 (15%)	102 (22%)	0 (0%)	12 (18%)
Fingertip	Functional	3638 (81%)	476 (75%)	2037 (83%)	1044 (81%)	6 (100%)	75 (74%)
	Non-functional	842 (19)	157 (25%)	415 (17%)	244 (19%)	0 (0%)	26 (26%)

Table 3: Pulse Oximeter Access from the Federal Ministry of Health National Oxygen System Assessment (2022) –

Among frontline healthcare workers trained in the last 5 years, many facilities assessed provided training to nurses (30%) and doctors (28%) within the last 1-3 years. However, only 132 (16%) assessed public secondary and tertiary health facilities reported having the capacity for equipment maintenance and installation. Both the national and subnational assessments underscore the persisting challenge of maintenance practices for oxygen equipment. While the national assessment provides a broad overview of pulse oximeter distribution and functionality across facilities, the subnational assessments offer detailed insights into specific factors like coverage, changes over time, and the presence of guidelines. Nevertheless, both the national survey and the smaller assessments indicated a clear need for the adoption and implementation of guidelines and policies to ensure proper use and management of oxygen sources and equipment at national and sub-national levels.

From a National Plan to local implementation

Early adopters of a National Oxygen Plan

Before the adoption of the WHO executive board draft resolution by the 76th World Health Assembly in May 2023,[75] the Nigerian government had already published National Policy on Medical Oxygen in 2016, and a National Strategy for the Scale-up of Medical Oxygen in Health Facilities 2017-2022.[76] Its development garnered attention for medical oxygen and interest from the wider government, leading to investments in medical oxygen systems before COVID-19 arrived. A National multi-stakeholder coordinating platform, United for Oxygen (U4O), was developed in the same year. U4O is chaired by the Oxygen Desk of the Department of Hospital Services of the Federal Ministry of Health. This platform includes stakeholders such as the Federal MOH, academics, implementation partners and private sectors, and is responsible for providing reports on the implementation status to the government and President.

Three years after the launch of the first roadmap, the world was hit by the COVID-19 pandemic, substantially increasing oxygen demand and reshaping the medical oxygen landscape. This crisis prompted unprecedented investments in oxygen systems by the Nigerian government, donor agencies, and implementing partners,[20] contributing to gains such as the establishment of oxygen coordinating platforms at sub-national levels, procurement of oxygen commodities and consumables to meet demand, conduct of rapid assessments and national quantification exercises, and increased awareness of oxygen as an essential medicine.

Following the expiration of the 5-year validity period of the first strategy roadmap, the Federal Government and its partners revised the strategy in 2022, incorporating lessons learned to respond to COVID-19 context – launching the new National Strategy for the Scale-up of Medical Oxygen in Health Facilities (2023 – 2027). The new strategy builds on the implementation gap in the previous document, with a costed implementation plan that includes dissemination and a performance tracking framework. The National Clinical Guidelines on Oxygen Use was developed just prior to the revision of the National strategy in 2022. These guidelines provide specific recommendations to various healthcare professionals, encompassing the clinicians, biomedical engineers, administrators, and health officers. They aim to guide these professionals in the appropriate treatment and care of patients requiring oxygen, and on the appropriate screening of hypoxaemia in patients visiting all tiers of the healthcare system and handling of oxygen equipment (including diagnostic equipment and oxygen delivery devices). This document proves particularly valuable by offering guidance on management of hypoxaemia in all patient groups including neonates, children and adults. Furthermore, they assist individual health facilities in selecting the appropriate oxygen delivery systems.[77]

Multi-stakeholder coordination

With the launch of the second National Oxygen Strategy in 2022, roles of stakeholders in implementation, particularly at the subnational level, were clearly defined. The established subnational oxygen coordinating platforms and oxygen desks now play pivotal role, guided by clear terms of reference that align with the overarching National Strategy for the Scale-up of medical oxygen. Their establishment aimed to address the need for improved inventory coordination, enhanced visibility of oxygen delivery services, and more effective management of resources.

This United for Oxygen multi-stakeholder coordinating platform played a pivotal role in aligning the National Strategy's goals with the broader healthcare landscape, ensuring a unified approach towards achieving the strategy's objectives. At the subnational level, the coalition leveraged support from State Ministries of Health (SMOH), State Health Care Boards, and Hospital Management Boards for implementing the strategy. However, significant challenges emerged in the strategy's implementation, including low adoption and prioritization, inadequate financing mechanisms leading to oxygen scarcity, and high oxygen prices, particularly at subnational levels. These challenges were apparent in health facilities struggling to meet oxygen demand and an inequitable delivery of medical oxygen to patients.[41] Weak equipment maintenance services, undefined oxygen production operating models and public private partnership structures, low health capacity, and lack of quality data were identified as persistent gaps. Table 4 describes key stakeholders and stakeholder groups related to medical oxygen in Nigeria.

Partner	Role and Area of Collaboration
Federal MOH	• The National Oxygen Desk sits in the FMOH. Provides overall Oxygen programme coordination in the country through the national and subnational coordinating mechanisms.<br>• The National Oxygen Desk maintains a national database to map and coordinate oxygen investments and interventions.<br>• Increasing domestic resource mobilization for oxygen systems through advocacy, budgets etc.<br>• Convenes biannual National Oxygen Coordination Meeting with state oxygen desk officers and other U4O partners.<br>• Second edition was held in August 2023.
United for Oxygen (U4O)	• National multi-stakeholder coordinating platform for oxygen in Nigeria, set up to align approaches and provide strategic guidance to government, donors and partners implementing medical oxygen-related interventions at national and sub-national levels.<br>• Facilitated the review of the National Oxygen Strategy and will be driving implementation.
Bill and Melinda Gates Foundation	• Provided direct funding to CHAI support for the C-19 response in Lagos state, including installation of a PSA plant in the C-19 treatment facility and establish and rolling out oxygen triage centres as part of the C-19 response.<br>• Support through direct funding to CHAI for respiratory care systems strengthening for the C-19 response across 8 states in Nigeria.<br>• Providing direct funding supply to CHAI to strengthen oxygen systems nationally and in five states through the MOXY project.
Unitaid	• Unitaid has funded CHAI through two grants to improve oxygen systems for the C-19 response through direct capital and equipment investments and in system strengthening to build resilient oxygen systems:<br>• *Grant One Emergency Procurements*: Emergency procurement of 11 200kVA generators to support new PSAs plants procured by the Government of Nigeria across 11 Tertiary Health Facilities in the country<br>• *Grant Two Resilient Oxygen Systems*: Supported C-19 oxygen systems strengthening work across 6 states in Nigeria and the FCT, including building oxygen systems and support to revise the National Oxygen Strategy; training of Biomedical Engineers (BMEs) on repair and maintenance of PSA oxygen plants; training of HCWs on hypoxaemia management (45 Trainers and 1,296 HCWs); procurement of tool kits for trained BMEs and repair drives in ~130HFs across 5 states.<br>• *Optimizing equipment and supply*: Roll out of facility assessments in 578 facilities and supporting national oxygen gap assessments
Global Fund	• The Global Fund, through the C19-RM, has supported investments in strengthening oxygen and respiratory care systems for the C19 response.<br>• As PRs to the C19-RM grant, the National Aids Control Agency (NACA) and the NTBLCP have supported CHAI, WHO and other partners to expand supply through direct investments in equipment procurement, installation of PSA plants and liquid oxygen tanks as well as the necessary systems support to optimize these investments.

	• Under this grant, CHAI is undertaking: Oxygen piping expansion in 44 tertiary facilities; Nationwide COVID-19 and hypoxemia case management trainings for ~1700 HCWs from secondary and tertiary facilities; Scale up of subnational Oxygen coordinating mechanisms across the country; BME trainings and repair drives across 36 states and the FCT
WHO	• WHO is a member of the U4O coalition and provided support for oxygen for the C19 response and for overall systems strengthening.<br>• Collaborated with CHAI to update the national C-19 guidelines and training materials to include hypoxemia.<br>• Supported a national Training of Trainers on COVID-19 and hypoxemia case management for case managers.<br>• Worked collaboratively with CHAI and the FMOH to support dissemination of the revised national strategy at the sub-national level
UNICEF	• Active member of the U4O platform<br>• Joined and supported the review of the National Oxygen strategy along with CHAI and other partners<br>• Supporting the government to expand oxygen supply through the procurement and installation of 9 PSA plants in 9 HFs<br>• Successfully included oxygen in IMCI training packages that have been used to roll out IMCI trainings for HCWs<br>• Working closely with CHAI to define priority indicators for oxygen and in rolling appropriate HMIS systems for oxygen<br>• Worked closely with CHAI to adapt the OSPT tool and roll out national supply assessments that were used to develop national gap quantifications
FHI360	• FHI-360 receives funding through USAID's EPIC program for direct oxygen infrastructure investments and for overall oxygen systems strengthening.<br>• Providing overall TA support to the C19-RM grant across three thematic areas including for Oxygen<br>• Through this grant, FHI is procuring and installing 6 liquid oxygen tanks and are providing overall support for the filling of 2 existing LOX tanks. This also includes oxygen piping expansion in these prioritized facilities<br>• Jointly supported the revision of the National Oxygen Strategy with CHAI, and working with CHAI to establish a working group to review all investments and supply in order to develop appropriate business plans and support oxygen market shaping efforts
Oxygen for Life Initiative (OLI)	• Provides support for review of clinical guidelines and training resources<br>• Support the FMOH with HCW capacity building efforts<br>• Engaged by CHAI to directly support the revision of the National Oxygen Strategy

Table 4: Key medical oxygen stakeholders in Nigeria

The timeliness of COVID-19 for the first strategic plan

Nigeria's first national roadmap on medical oxygen preceded the COVID-19 pandemic, and at the Federal level, there were some positive gains, especially the creation of a budget line for medical oxygen. Pre-COVID, the Federal government was conducting regional trainings, rather than State level, due to limited funds. Awareness of the policy document at that time was poor

due to a lack of funding and pre-defined dissemination plan. Nevertheless, the policy proved valuable during the pandemic for resource mapping and gap quantification.

The COVID-19 pandemic was a game changer in medical oxygen security in Nigeria. Firstly, there was re-orientation of stakeholders on the importance of medical oxygen—a shift from end-of-life therapy to an essential medication that should be always available. The pandemic created awareness about the existing medical oxygen roadmap, spawned interest in many partners on medical oxygen, and consequently increased investment in the medical oxygen system not limited to procurement and installation of oxygen plants and capacity building of healthcare workers including biomedical engineers and technicians. Post-COVID-19, there is a belief that access to oxygen has improved. The following are testimonies from some of the key stakeholders we interviewed:

> *"When the pandemic came, surprisingly Nigeria is one the very few African countries that had a strategy on ground, even though we had the strategy, the reality is that how much were you using the strategy, that's the question, but you know, since the pandemic came, awareness, a lot of awareness has been created and a lot of people got to know about the strategy"* Participant 7

> *"From 2016, 2017, 2018, if you look at the conversation around oxygen and the management of oxygen, it has significantly improved because check around and see, 2017 and 2018, most of our health care workers actually never knew how to manage oxygen, but you can see, if you're going to take a statistics now across the health facilities, you'll find out that on an average on yearly basis, over a hundred to a hundred and fifty health care workers have been trained either by UNICEF, WHO, or by CHAI across the country."* Participant 6

> *"Having medical oxygen security post-COVID became an issue that the government, health facilities, policymakers started to pay attention to."*
> Participant 3

This underscores the importance of proactive planning, effective dissemination, and strategic allocation of resources in healthcare. It also highlights how a crisis can serve as a catalyst for positive change, resulting in increased investments and improvements in healthcare systems. The focus on medical oxygen, a critical resource during the pandemic, serves as a case study for the broader principles of preparedness and response to public health emergencies.

Multiple actors, with competing agendas, can hinder effective financing

Sources of funding for medical oxygen in Nigeria include governments, private sector, donor agencies and philanthropists. Publicly owned medical oxygen equipment and technologies are procured by the Federal and State Ministries or donors.[20,76] However, divergent opinions exist on the major financier of medical oxygen. On one hand, the government is regarded as the major financier of medical oxygen systems, yet funding from the government was limited prior to the COVID-19 pandemic. This is attributed to insufficient advocacy for oxygen scale-up and low prioritization of medical oxygen at national and sub-national levels.[20] On the other hand, donor agencies are perceived as the major source of funds for oxygen, with government expenditure on medical oxygen only increasing after the COVID-19 pandemic.

Nevertheless, release of appropriated funds by the government for oxygen related activities was highlighted as a major problem—erratic and insufficient. Government funding for health is

generally below the international benchmarks. Security challenges in the country were identified as a competing priority for government funding, sometimes resulting in conflicts between governments and partners. Evidence suggests that facilities across States occasionally partner with private sector players and financing systems to sustain supply of medical oxygen through various PPP arrangements.[20] However, out-of-pocket expenditure was not mentioned as part of financing mechanism for medical oxygen, despite direct costs of oxygen to patients being common place, and a major barrier to effective oxygen delivery. One participant highlighted that funding opportunities from private individuals (philanthropists) have not been fully explored.

> "*Affordability is a major issue. So, I work in a facility where most ailment care is out of pocket. If the parents of a child do not have the funds, then they cannot get oxygen even if it is available, so affordability is key for the provision of oxygen.*" Participant 3

> *"If there is a possibility of engaging NGOs or people who are well to do in the society that can donate for oxygen provision. Possibility of involving people that can donate freely, so if you have people in the society that can be donating money for the procurement of oxygen."* Participant 1

However, a crucial milestone was reached during the 64th National Council of Health in November 2023. The approval of a memo titled "*Medical Oxygen Investment (Pressure Swing Adsorption Plant, Liquid Oxygen Tank and Other Medical Oxygen Accessories) Sustainability*" signifies a step towards establishing a policy framework for sustaining investment in medical oxygen. The National Council of Health resolution mandates collaborative efforts between the Federal Ministry of Health, related agencies, parastatals, and the State Ministries of Health to: *"Secure a dedicated account for medical oxygen investment which shall be managed by the Federal Government of Nigeria and to also develop and implement a Standard Operation Procedure (SOP) for the management of these equipment".*[79,80]

Oxygen desks as an accountability solution, but sustainability needs to be prioritised

The Department of Hospital Services of the Federal MOH coordinates medical oxygen and medical oxygen service interventions in Nigeria, offering strategic guidance to the government, partners, and stakeholders.[20] Various government ministries, departments and agencies are involved in regulation of medical oxygen in the country. Specifically, the Federal Ministry of Health, Standard Organization of Nigeria, and the National Agency for Food and Drug Administration and Control (NAFDAC). Participants highlighted that the roles of these bodies are not clearly defined, and there is lack of synergy among them. These bodies are also hindered by inadequate funding and insufficient personnel.

Within the Federal MOH, there is an 'oxygen desk' tasked with the responsibility to coordinate, supervise and implement oxygen related activities. The desk superintends procurement and installation processes for oxygen plants and ensures that they conform with international standards. The concept of the oxygen desk has been expanded to the State level, where they are responsible for assessing oxygen gaps, demand and supply, as well as advocating for medical oxygen in their respective States. At the time of the national strategy review, oxygen desks were established in 34 of 36 States, with coordinating fora in 16 through support from partners.[20] However, by the end of 2023, oxygen desks and multi-sectoral oxygen coordinating forums have been successfully established in all states in the country, operating under the oversight of the U4O coordinating platform.

Prior to 2021, when the coordination platforms or desks responsible for the management of medical oxygen systems at the State Ministries of Health were non-existent, there was a notable gap in accountability. These included poorly coordinated inventory, lack of visibility of oxygen delivery services and management of resources.[20] At subnational level, oxygen desks aim to bridge this accountability gap by improving inventory coordination and effective resource management. As a result, state oxygen desks play a crucial role in the adoption, adaptation, and domestication of policy documents and strategies at the subnational level. This is particularly vital due to the heterogeneity in contexts across Nigeria. However, one participant we interviewed expressed the desire for State oxygen desks to be government funded, rather than externally supported and donor driven. To further empower State oxygen desks and coordinating platforms, essential tools, resources, and capacity-building initiatives are being provided, to ensure they offer strategic support for oxygen programming at state level.

Healthcare workers were also identified as key players of accountability mechanisms for medical oxygen. It is the responsibility of the healthcare workers to ensure that the patients receive medical oxygen in appropriate dose, delivery method and required duration.

> *"So, oxygen security in Nigeria is a responsibility of every health worker as well as all the partners involved in oxygen support and intervention... Then we also have the biomedical engineers, and then we also have the end users, those are the health workers."* Participant 2

> *"Well, I guess both the physician and the nurses, we try to make sure the child gets it. We try to overcome every obstacle and make sure the child gets it and then we monitor. So, I think it's everybody's responsibility in terms of both doctors and nurses."* Participant 3

Beyond oxygen availability, other challenges continue to inhibit equitable access

While there is a growing recognition of the importance of medical oxygen and its improved availability, other barriers to oxygen provision at the facility level persist, relating to financing,[58,65,81] infrastructure (e.g., power supply and poor oxygen/oximetry equipment),[54,58,60,61,82] knowledge gaps,[54,61,72,83] and the need for and implementation of comprehensive guidelines and policies – Table 5.[52,64]

The cost of medical oxygen is consistently identified as a significant barrier, both in routine healthcare and in the context of managing conditions like COVID-19.[58,65,81] The financial burden extends to both healthcare facilities and patients. Beyond oxygen availability on the ward, oxygen needs to be affordable to patient. One participant stressed the need for free healthcare services for children and called for policy to ensure unhindered provision of medical oxygen for patients in need during emergencies irrespective of caregiver's ability to pay.

> "*For the care of children, especially in emergencies, policy should be stated that allows the free provision of oxygen, medical oxygen, for the care of children.*" Participant 3

During the COVID-19 pandemic there were concerns regarding the huge cost of medical oxygen including the financial implications of managing severe COVID-19 cases.[65,81] Post pandemic, medical oxygen remains expensive to patients. There are currently no clear strategies to make oxygen free or affordable, though efforts to include it in the national insurance scheme are ongoing.

Besides oxygen cost, inadequate infrastructure and equipment pose significant challenges. These include issues with power supply, lack of clinical guidelines, and limited access to essential equipment like pulse oximeters and oxygen concentrators, and other consumables and ancillary oxygen equipment, particularly in non-tertiary hospitals.[54,58,60,61,82] Non-availability of the appropriate medical oxygen equipment at the point of care is a source of stress for healthcare workers. There are also challenges in implementing comprehensive oxygen therapy programmes, including the lack of piped oxygen, limited on-site oxygen generation capabilities, and challenges in the supply chain for oxygen cylinders and concentrators.[61] There was a recurring theme of knowledge gaps among healthcare providers regarding acute oxygen therapy in the literature. This includes misconceptions about oxygen as a drug, prescribing practices, and appropriate device usage.[54,61,72,83]

Reference	Population	Setting	Main findings
Orimadegun *et al.* (2011)	Paediatricians	Various children healthcare centres in Nigeria	The nationwide survey assessed paediatricians' knowledge of pulse oximetry, revealing gaps in training and knowledge among healthcare providers. It also highlighted equipment availability challenges in healthcare centres. Most respondents reported that they had no pulse oximeters in their neonatal care units (79%) and emergency units (70%).[54]
Desalu *et al.* (2011)	Asthma patients	Tertiary hospitals	The cross-sectional study assessed facilities' readiness for asthma care, pointing out challenges in adherence to internationally endorsed standards. It indicated gaps in the availability of essential equipment (38.2% had pulse oximeter) and clinical guidelines.[52]
Graham *et al.* (2016)	Children and neonates	Non-tertiary hospitals in south-west Nigeria	The needs assessment of non-tertiary hospitals highlighted structural, technical, and clinical barriers to safe and effective oxygen therapy, emphasizing issues with power supply, equipment, and the high cost of oxygen.[58]
Aneji *et al.* (2020)	HCWs and hospital administrators	Hospitals involved in the bCPAP program	A qualitative study assessing the implementation of a bCPAP program revealed challenges in oxygen infrastructure. However, the hands-on training had a positive impact on staff development, indicating the importance of training in improving healthcare practices.[61]
Walker *et al.* (2020)	Neonates	Three hospitals in southwest Nigeria	The prospective validation study evaluated the effectiveness of intermittent pulse oximetry in guiding oxygen therapy in neonates, emphasizing the need for more frequent monitoring to improve oxygen targeting and potentially prevent harm from ROP and BPD , particularly in low-resource settings.[64]

Shittu *et al.* (2020)	HCWs	Health facilities in Lagos and Jigawa states, Nigeria	The mixed-methods study conducted facility audits, surveys and focus group discussion, indicating the availability of oxygen and pulse oximeters in secondary care facilities. However, challenges in primary facilities were identified, emphasizing disparities in infrastructure.[82]
King *et al.* (2020)	Stakeholders involved in paediatric pneumonia	National, Jigawa and Lagos states	The concurrent mixed-methods study explored stakeholder perspectives on paediatric pneumonia, emphasizing insufficient equipment, particularly oxygen and pulse oximeters. The study also noted challenges related to the power supply, a critical aspect during the COVID-19 pandemic.[60]
Banke-Thomas *et al.* (2021)	Pregnant women with COVID-19	Tertiary hospital in Lagos, Southwestern Nigeria	The hospital-based cost analysis during the COVID-19 pandemic highlighted medical oxygen as a major cost driver, emphasizing the financial implications of managing severe COVID-19 cases.[65]
Desalu *et al.* (2022)	HCWs	Public tertiary hospital in the Middle Belt region in Nigeria	The cross-sectional study among healthcare providers revealed knowledge gaps in acute oxygen therapy. It also highlighted barriers in oxygen delivery, such as a shortage of supply, inadequate delivery devices, power outages, and out-of-pocket costs.[83]
Adeoti *et al.* (2022)	Patients and caregivers	Tertiary hospital in Ado Ekiti, southwestern Nigeria	The study in Ekiti State University Teaching Hospital emphasized the perception of patients and caregivers, highlighting beliefs in the benefits of oxygen therapy and concerns about its cost. This speaks to financing challenges and the need for addressing patient perspectives.[81]

Table 5: Challenges and barriers to the availability and effective use of oxygen and oximetry. HCW = healthcare worker

Key Messages

- Nigeria was one of the first countries to establish a national oxygen strategy. While the launch of this policy had started to mobilise action, the COVID-19 pandemic provided the stimulus needed for widespread awareness and resource mobilisation.
- Medical oxygen systems involve a wide range of actors. As a solution, Nigeria has established Oxygen Desks at the Federal and State levels to support coordination and monitoring of oxygen supply and need.
- Despite medical oxygen receiving political focus, wider health system issues limit their impact, with poor infrastructure, healthcare worker capacity and high out of pocket costs for patients, preventing equitable access.

Additional methods information

Desk-based review

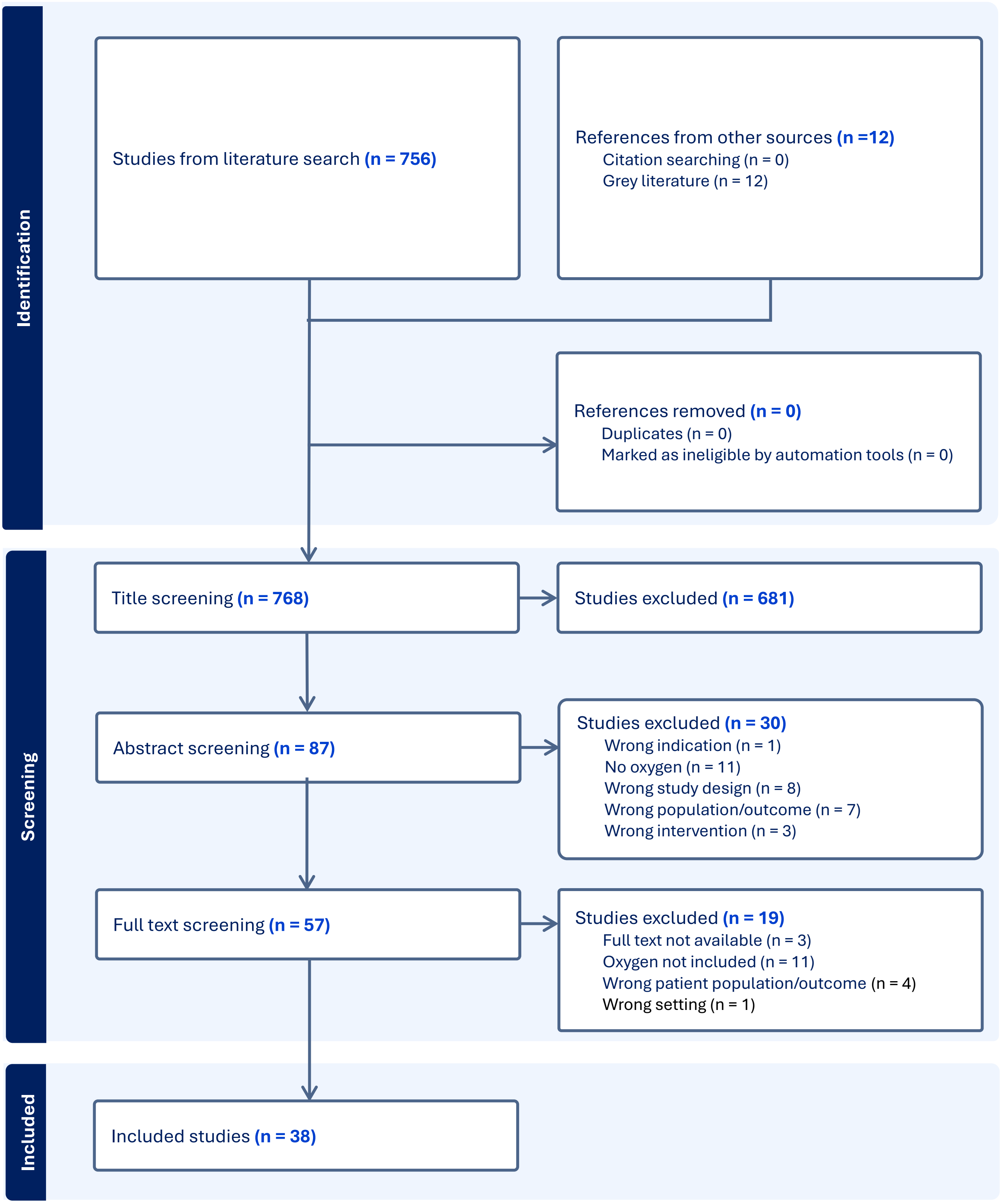

Figure 3: Academic and grey literature inclusion

Key informant interviews

Participants were purposively sampled to ensure at least one representative is included from the following groups: government, non-governmental organizations and professional body. A process of stakeholder mapping using the Lancet Oxygen Commission's networks, from academic and grey literature, and snowballing informed the participant selection. We ensured a diversity in roles, interest and power amongst the participants.

Individuals were contacted initially by email from Professor A. G. Falade, or anyone assigned by him. Participants were emailed up to three times to try and make initial contact and then followed with a phone call or approached in person at a meeting (e.g., Academic conference). In situations where none of these approaches were successful, the participant was considered as a "non-respondent" and an alternative participant was approached.

	Organisation	Role	Gender
1	University of Medical Sciences, Teaching Hospital, Akure, Ondo State.	Biomedical Technologist	Female
2	Federal Ministry of Health, Department of Hospital Services	Principal Medical Officer	Male
3	University College Hospital, Ibadan	Paediatric Cardiologist, Senior Lecturer/ Consultant	Female
4	UNICEF	Health Specialist	Female
5	CHAI	Public Health Analyst	Female
6	Kaduna State Ministry of Health	Biomedical Engineer	Male
7	Jos University Teaching Hospital	Public Health Physician	Male
8	FHI360	Background in Medicine and Public Health	Male

Table 6: Key informant participants

References

1. United Nations Population Fund. World Population Dashboard -Nigeria. Published 2022. Accessed July 29, 2023. https://www.unfpa.org/data/world-population/NG
2. UNICEF. UNICEF Data: Monitoring the situation of children and women - Nigeria. UNICEF Data. Published 2023. Accessed July 29, 2023. https://data.unicef.org/country/nga/
3. National Bureau of Statistics (NBS). Nigeria 2021 Multiple Indicator Cluster Survey (MICS) & National Immunization Coverage Survey (NICS): Survey Findings Report. 2022;(August).
4. International Monetary Fund. IMF Data Mapper. Published 2023. Accessed July 29, 2023. https://www.imf.org/external/datamapper/profile/NGA
5. The World Bank. Current health expenditure per capita (current US$) - Nigeria. Published 2023. Accessed July 29, 2023. https://data.worldbank.org/indicator/SH.XPD.CHEX.PC.CD?locations=NG
6. The World Bank. World Bank Country and Lending Groups – World Bank Data Help Desk. Published 2023. Accessed September 11, 2023. https://datahelpdesk.worldbank.org/knowledgebase/articles/906519-world-bank-country-and-lending-groups
7. World Bank Group. Poverty & Equity Brief: Africa Western & Central - Nigeria. 2023;(April).
8. World Health Organization. Global Health Estimates 2020: Deaths by Cause, Age, Sex, by Country and by Region, 2000-2019. Published 2020. Accessed September 11, 2023. https://www.who.int/data/gho/data/themes/mortality-and-global-health-estimates/ghe-leading-causes-of-death
9. Odejimi A, Quinley J, Eluwa GI, et al. Causes of deaths in neonates and children aged 1 – 59 months in Nigeria : verbal autopsy findings of 2019 Verbal and Social Autopsy study. *BMC Public Health*. Published online 2022:1-15. doi:10.1186/s12889-022-13507-z
10. United Nations. SDG Indicators — Global indicator framework for the Sustainable Development Goals and targets of the 2030 Agenda for Sustainable Development. Published 2023. Accessed July 29, 2023. https://unstats.un.org/sdgs/indicators/indicators-list/
11. Clinton Health Access Initiative. Oxygen Therapy. Published 2023. Accessed September 11, 2023. https://www.clintonhealthaccess.org/our-programs/oxygen/
12. Federal Ministry of Health. National Policy on Medical Oxygen in Health Facilities. Published online 2017. http://www.health.gov.ng/doc/NATIONAL POLICY ON MEDICAL OXYGEN.pdf
13. Graham H, Bakare AA, Ayede AI, et al. Hypoxaemia in hospitalised children and neonates: A prospective cohort study in Nigerian secondary-level hospitals. *EClinicalMedicine*. 2019;16:51-63. doi:10.1016/j.eclinm.2019.10.009
14. Ozoh OB, Aderibigbe SA, Ayuk AC, et al. The prevalence of asthma and allergic rhinitis in Nigeria: A nationwide survey among children, adolescents and adults. Published online 2019. doi:10.1371/journal.pone.0222281
15. Desalu OO, Adeoti AO, Ogunmola OJ, Fadare JO, Kolawole TF. Quality of acute asthma care in two tertiary hospitals in a state in South Western Nigeria: A report of clinical audit. *Niger Med J*. 2016;57(6):339-346. http://ovidsp.ovid.com/ovidweb.cgi?T=JS&PAGE=reference&D=pmnm3&NEWS=N&AN=27942102
16. Nwokediuko SC, Obienu O. Sedation practices for routine diagnostic upper gastrointestinal endoscopy in Nigeria. *World J Gastrointest Endosc*. 2012;4(6):260-265. doi:10.4253/wjge.v4.i6.260
17. Ale BM, Ozoh OB, Gadanya MA, et al. Estimating the prevalence of COPD in an African country: evidence from southern Nigeria. *J Glob Heal reports*. 2022;6. doi:10.29392/001C.38200
18. World Health Organization. Primary health care systems (PRIMASYS): case study from

Nigeria. *Geneva*. Published online 2017:1-16. https://apps.who.int/iris/rest/bitstreams/1346150/retrieve
19. National Primary Health Care Development Agency (NPHCDA). Minimum Standards for Primary Health care in Nigeria. *Nphcda*. Published online 2010:1-71.
20. Federal Ministry of Health. *National Strategy for the Scale-up of Medical Oxygen in Health Facilities 2023-2027*.; 2023.
21. Federal Ministry of Health. Nigeria Health Facility Registry. Published 2023. Accessed July 30, 2023. https://hfr.health.gov.ng/statistics/tables
22. Ahmat A, Okoroafor SC, Kazanga I, et al. The health workforce status in the WHO African Region: findings of a cross-sectional study. *BMJ Glob Heal*. 2022;7:1-8. doi:10.1136/bmjgh-2021-008317
23. World Health Organization. Global strategy on human resources for health: Workforce 2030. *Who*. Published online 2016:64. Accessed January 6, 2024. https://www.who.int/hrh/resources/global_strategy_workforce2030_14_print.pdf?ua=1
24. Federal Ministry of Health. National Health Accounts 2017. 2019;(April). https://www.health.gov.ng/doc/FINAL-VERSION-NHA-2017.pdf
25. Uzochukwu BSC, Ughasoro MD, Etiaba E, Okwuosa C, Envuladu E, Onwujekwe OE. Health care financing in Nigeria: Implications for achieving universal health coverage. *Niger J Clin Pract*. 2015;18(4):437-444. doi:10.4103/1119-3077.154196
26. Aregbeshola BS, Khan SM. Predictors of Enrolment in the National Health Insurance Scheme Among Women of Reproductive Age in Nigeria. *Int J Heal policy Manag*. 2018;7(11):1015-1023. doi:10.15171/IJHPM.2018.68
27. Sasu DD. Health insurance coverage in Nigeria 2018, by type and gender. Statista. Published February 1, 2022. Accessed January 6, 2024. https://www.statista.com/statistics/1124773/health-insurance-coverage-in-nigeria-by-type-and-gender/
28. Adebowale-Tambe N. Tinubu to increase health allocation to 10% of total budget - Aide. Premium Times Nigeria. Published July 25, 2023. Accessed September 29, 2023. https://www.premiumtimesng.com/news/top-news/611918-tinubu-to-increase-health-allocation-to-10-of-total-budget-aide.html
29. Odunyemi AE. The Implications of Health Financing for Health Access and Equity in Nigeria. *Healthc Access*. Published online August 26, 2021. doi:10.5772/INTECHOPEN.98565
30. One. Post-Pandemic Health Financing by State Governments in Nigeria, 2020 to 2022. Published online 2022.
31. Ajala-Damisa D, Agbaoye K. From A Scheme to an Authority - 5 Things You Need to Know About the New NHIA Act. Nigeria Health Watch. Published May 25, 2022. Accessed September 17, 2023. https://articles.nigeriahealthwatch.com/from-a-scheme-to-an-authority-5-things-you-need-to-know-about-the-new-nhia-act/
32. World Health Organization. WHO supports Nigeria in operationalizing the National Health Insurance Authority Act 2022. Published August 1, 2022. Accessed September 17, 2023. https://www.afro.who.int/countries/nigeria/news/who-supports-nigeria-operationalizing-national-health-insurance-authority-act-2022
33. Onyekpere E. Review of the 2024 federal health budget proposal (1). Punch Newspaper. Published December 18, 2023. Accessed January 14, 2024. https://punchng.com/review-of-the-2024-federal-health-budget-proposal-1/?utm_source=auto-read-also&utm_medium=web
34. Angbulu S. Why FG chose N800/dollar exchange rate for 2024 budget. Punch Newspaper. Published January 12, 2024. Accessed January 14, 2024. https://punchng.com/why-fg-chose-n800-dollar-exchange-rate-for-2024-budget-minister/
35. World Health Organization. Nigeria: WHO Coronavirus Disease (COVID-19) Dashboard With Vaccination Data. Published 2023. Accessed August 1, 2023.

https://covid19.who.int/region/afro/country/ng
36. Federal Ministry of Health. Health Minister: First Case Of COVID-19 Confirmed In Nigeria. Published February 28, 2020. Accessed August 1, 2023. https://www.health.gov.ng/index.php?option=com_k2&view=item&id=613:health-minister-first-case-of-covid-19-confirmed-in-nigeria
37. Olawale G. COVID-19 : Experts worry over challenges as Nigeria rolls out vaccines. Published online 2021:1-6.
38. UNICEF Nigeria. UNICEF Nigeria on Twitter: History is made! Published March 11, 2021. Accessed August 1, 2023. https://twitter.com/UNICEF_Nigeria/status/1367809774100877319
39. CACOVID. Private sector coalition against COVID-19. Published 2020. Accessed January 8, 2024. https://www.cacovid.org/
40. Business Day. CACOVID: Nigeria's private-sector response to COVID-19 -. Businessday NG. Published April 15, 2020. Accessed January 8, 2024. https://businessday.ng/coronavirus/article/cacovid-nigerias-private-sector-response-to-covid-19/
41. Graham HR, Olojede OE, Bakare AA, et al. Measuring oxygen access: Lessons from health facility assessments in Lagos, Nigeria. *BMJ Glob Heal*. 2021;6(8):1-10. doi:10.1136/bmjgh-2021-006069
42. Adepoju P. How COVID-19 laid bare Africa's medical oxygen crisis. Devex. Published March 22, 2022. Accessed September 16, 2023. https://www.devex.com/news/how-covid-19-laid-bare-africa-s-medical-oxygen-crisis-102845
43. George L, Akwagyiram A. "Oxygen, oxygen, oxygen": Nigeria battles shortages amid COVID-19 surge. Reuters. Published February 2, 2021. Accessed September 16, 2023. https://www.reuters.com/business/healthcare-pharmaceuticals/oxygen-oxygen-oxygen-nigeria-battles-shortages-amid-covid-19-surge-2021-02-02/
44. Njoku L, Daniel E, Edward O. Oxygen crisis hits Nigeria's COVID-19 response scheme — Nigeria. The Guardian Nigeria News. Published January 7, 2021. Accessed September 16, 2023. https://guardian.ng/news/oxygen-crisis-hits-nigerias-covid-19-response-scheme/
45. Aregbeshola BS, Folayan MO. Nigeria's financing of health care during the COVID-19 pandemic: Challenges and recommendations. *World Med Heal Policy*. 2022;14(1):195. doi:10.1002/WMH3.484
46. UNICEF Nigeria. UNICEF and IHS Nigeria partner to expand access to oxygen for pneumonia and COVID-19 patients. Published August 26, 2022. Accessed September 16, 2023. https://www.unicef.org/nigeria/press-releases/unicef-and-ihs-nigeria-partner-expand-access-oxygen-pneumonia-and-covid-19-patients
47. United Nations Development Programme. UN procures ventilators and other medical supplies to boost the Government of Nigeria's response to COVID-19. Published April 14, 2020. Accessed August 1, 2023. https://www.undp.org/nigeria/press-releases/un-procures-ventilators-and-other-medical-supplies-boost-government-nigeria's-response-covid-19
48. Federal Ministry of Health. FG Distributes Oxygen Concentrators & Ventilators To Various Health Institutions, Trains 176 Specialists. Published September 11, 2020. Accessed August 1, 2023. https://health.gov.ng/index.php?option=com_k2&view=item&id=765:fg-distributes-oxygen-concentrators-ventilators-to-various-health-institutions-trains-176-specialists
49. Nigeria Centre for Disease Control. NCDC COVID-19 Case Update. Published March 23, 2020. Accessed August 1, 2023. https://twitter.com/NCDCgov/status/1242025530070687750?lang=en
50. Federal Ministry of Health. COVID-19: US Donates 200 Life Saving Ventilators To Nigeria. Published August 11, 2020. Accessed August 1, 2023. https://health.gov.ng/index.php?option=com_k2&view=item&id=739:covid-19-us-

donates-200-life-saving-ventilators-to-nigeria
51. Mokuolu OA, Ajayi OA. Use of an oxygen concentrator in a Nigerian neonatal unit: economic implications and reliability. *Ann Trop Paediatr*. 2002;22(3):209-212. http://ovidsp.ovid.com/ovidweb.cgi?T=JS&PAGE=reference&D=med4&NEWS=N&AN=12369483
52. Desalu OO, Onyedum CC, Iseh KR, Salawu FK, Salami AK. Asthma in Nigeria: are the facilities and resources available to support internationally endorsed standards of care? *Health Policy*. 2011;99(3):250-254. doi:10.1016/j.healthpol.2010.10.006
53. Ogunbosi BO, Orimadegun AE, Carson S. Clinical features associated with oxygen use in children presenting to emergency care in Ibadan, Nigeria. *Am J Respir Crit Care Med*. 2011;183(1). https://www.embase.com/search/results?subaction=viewrecord&id=L70847738&from=export
54. Orimadegu AE, Ogunbosi BO, Akinbami FO. Knowledge and Views of Paediatricians about Pulse Oximetry: a Nationwide Online Survey in Nigeria. *Afr j respir Med*. 2011;7(1):14-18. http://www.africanjournalofrespiratorymedicine.com/articles/september_2011/AJRM Sept pp 14-18.pdf http://www.africanjournalofrespiratorymedicine.com/articles/september_2011/AJRM20Sept20pp2014-18.pdf
55. Henry JA, Windapo O, Kushner AL, Groen RS, Nwomeh BC. A survey of surgical capacity in rural southern Nigeria: opportunities for change. *World J Surg*. 2012;36(12):2811-2818. doi:10.1007/s00268-012-1764-0
56. Abdulraheem MA, Orimadegun AE, Tongo O. Pre- and intra-transport neonatal care oxygen saturation hypoxemia among referred neonate in a tertiary hospital in Nigeria. *Am J Respir Crit Care Med*. 2015;191. https://www.embase.com/search/results?subaction=viewrecord&id=L72049701&from=export
57. Iroezindu MO, Isiguzo GC, Chima EI, et al. Predictors of in-hospital mortality and length of stay in community-acquired pneumonia: a 5-year multi-centre case control study of adults in a developing country. *Trans R Soc Trop Med Hyg*. 2016;110(8):445-455. doi:10.1093/trstmh/trw057
58. Graham HR, Ayede AI, Bakare AA, et al. Oxygen for children and newborns in non-tertiary hospitals in South-west Nigeria: A needs assessment. *Afr J Med Med Sci*. 2016;45(1):31-49.
59. CHAI. A government-led approach to increasing access to oxygen in Nigeria. Published November 2017. Accessed December 23, 2023. https://www.clintonhealthaccess.org/blog/government-led-approach-accessing-oxygen-nigeria/
60. King C, Iuliano A, Burgess RA, et al. A mixed-methods evaluation of stakeholder perspectives on pediatric pneumonia in Nigeria-priorities, challenges, and champions. *Pediatr Pulmonol*. 2020;55 Suppl 1:S25-S33. doi:10.1002/ppul.24607
61. Aneji C, Hartman T, Olutunde O, et al. Implementing bubble continuous positive airway pressure in a lower middle-income country: A nigerian experience. *Pan Afr Med J*. 2020;37:1-9. doi:10.11604/pamj.2020.37.10.24911
62. Bakare AA, Graham H, Ayede AI, et al. Providing oxygen to children and newborns: a multi-faceted technical and clinical assessment of oxygen access and oxygen use in secondary-level hospitals in southwest Nigeria. *Int Health*. 2020;12(1):60-68. doi:10.1093/inthealth/ihz009
63. Briggs DC, Eneh AU. Preparedness of primary health care workers and audit of primary health centres for newborn resuscitation in Port Harcourt, Rivers State, Southern Nigeria. *Pan Afr Med J*. 2020;36:68. doi:10.11604/pamj.2020.36.68.22164
64. Walker PJB, Bakare AA, Ayede AI, et al. Using intermittent pulse oximetry to guide

neonatal oxygen therapy in a low-resource context. *Arch Dis Child Fetal Neonatal Ed*. 2020;105(3):316-321. doi:10.1136/archdischild-2019-317630
65. Banke-Thomas A, Makwe CC, Balogun M, Afolabi BB, Alex-Nwangwu TA, Ameh CA. Utilization cost of maternity services for childbirth among pregnant women with coronavirus disease 2019 in Nigeria's epicenter. *Int J Gynaecol Obstet*. 2021;152(2):242-248. doi:10.1002/ijgo.13436
66. Graham HR, Olojede OE, Bakare AA, et al. Measuring oxygen access: lessons from health facility assessments in Lagos, Nigeria. *BMJ Glob Heal*. 2021;6(8):6069. doi:10.1136/BMJGH-2021-006069
67. Graham HR, Maher J, Bakare AA, et al. Oxygen systems and quality of care for children with pneumonia, malaria and diarrhoea: Analysis of a stepped-wedge trial in Nigeria. *PLoS One*. 2021;16(7 July). doi:10.1371/journal.pone.0254229
68. Ogunbiyi O, Sanusi A, Osinaike B, Yakubu S, Rotimi M, Fatungase O. An overview of intensive care unit services in Nigeria. *J Crit Care*. 2021;66:160-165. doi:10.1016/j.jcrc.2021.07.007
69. Bolu O, Mustapha B, Ihekweazu C, et al. Effect of Nigeria Presidential Task Force on COVID-19 Pandemic, Nigeria. *Emerg Infect Dis*. 2022;28(13):S168-S176. doi:10.3201/eid2813.220254
70. Graham HR, Bakare AA, Ayede AI, et al. Cost-effectiveness and sustainability of improved hospital oxygen systems in Nigeria. *BMJ Glob Heal*. 2022;7(8). doi:10.1136/bmjgh-2022-009278
71. Kalu QN, Edentekhe TA, Omoronyia OE, Nakanda BE, Archibong AA. State-wide situation analysis of availability of basic resuscitation devices and essential drugs in primary and secondary healthcare facilities in Cross River State, Nigeria. *Pan Afr Med J*. 2022;42:225. doi:10.11604/pamj.2022.42.225.35452
72. Okeke C, Uzochukwu B, Onyedinma C, Onwujekwe O. An assessment of Nigeria's health systems response to COVID-19. *Ghana med j*. 2022;56(3 suppl):74-84. doi:10.4314/gmj.v56i3s.9
73. CHAI. Biomedical engineers peer-led mentorship program increases workforce capacity and access to oxygen in Akwa Ibom State, Nigeria. Published March 28, 2023. Accessed December 23, 2023. https://www.clintonhealthaccess.org/blog/biomedical-engineers-increase-medical-oxygen-access-in-nigeria/
74. Kitutu FE, Rahman AE, Graham H, others. Announcing the Lancet Global Health Commission on medical oxygen security. *Lancet Glob Heal*. 2022;10:11.
75. Word Health Organization. WHO Resolution: Increasing access to medical oxygen. *WHO*. Published online May 2023.
76. Federal Republic of Nigeria. *National Strategy for the Scale-up of Medical Oxygen in Health Facilities 2017 - 2022.*; 2017.
77. Federal Ministry of Health. *National Clinical Guidelines on Oxygen Use*.; 2022.
78. Federal Ministry of Health. National Strategy For the Scale-up of Medical Oxygen in Health Facilities 2017 - 2022. *Fed Minist mof Heal*. Published online 2017.
79. Nigeria Medical World. FEDERAL MINISTRY OF HEALTH & SOCIAL WELFARE COMMUNIQUE ISSUED AT THE END OF THE 64TH NATIONAL COUNCIL OF HEALTH (NCH) MEETING HELD AT AB FOUNDATION CIVIL CENTRE, ADO-EKITI FROM 13TH - 17TH NOVEMBER, 2023. Published November 30, 2023. Accessed January 16, 2024. https://medicalworldnigeria.com/post/Federal-Ministry-of-Health-and-Social-Welfare-Communique-Issued-at-the-End-of-the-64th-National-Council-of-Health-NCH-Meeting-Held-at-AB-Foundation-Civil-Centre-Ado-Ekiti-from-13th-17th-November-2023?pid=67684
80. Nigeria Health Watch. Federal Ministry of Health in Collaboration with USAID funded EpiC Project Hosts a National Dialogue on Medical Oxygen Security. Published 2023. Accessed January 16, 2024. https://articles.nigeriahealthwatch.com/federal-ministry-of-health-in-

collaboration-with-usaid-funded-epic-project-hosts-a-national-dialogue-on-medical-oxygen-security/
81. Adeoti A, Desalu O, Elebiyo T, Aremu O. Misconception on oxygen administration among patients and their caregivers in Ado Ekiti, Nigeria. *Ann Afr Med*. 2022;21(3):269-273. doi:10.4103/aam.aam_63_21
82. Shittu F, Agwai IC, Falade AG, et al. Health system challenges for improved childhood pneumonia case management in Lagos and Jigawa, Nigeria. *Pediatr Pulmonol*. 2020;55 Suppl 1:S78-S90. doi:10.1002/ppul.24660
83. Desalu OO, Ojuawo OB, Adeoti AO, et al. Doctors' and Nurses' Knowledge and Perceived Barriers Regarding Acute Oxygen Therapy in a Tertiary Care Hospital in Nigeria. *Adv Med Educ Pract*. 2022;13:1535-1545. doi:10.2147/AMEP.S378533

THE LANCET Global Health
COMMISSION ON MEDICAL OXYGEN SECURITY

Country Case Study: Sweden

Coordinating access to medical oxygen in an aging population

Ann Liljas[1], *Carina King*[1], *Stefan Swartling Peterson*[1]

1. Department of Global Public Health, Karolinska Institutet, Sweden

Case study focus

The focus of the Swedish case study is the provision, access, demand and supply of medical oxygen to the ageing population of Sweden, with a particular focus on how oxygen is managed in home-based and care home settings. Sweden has a long history of home and care home-based oxygen treatment, used primarily amongst older patients with COPD. During the COVID-19 pandemic, this group of institutionalised elderly patients was particularly affected by the coronavirus, alongside widespread narratives that oxygen was unavailable, withheld, or not clinically useful within these settings.[1,2] This raised questions about the governance and adaptability of clinical policies for eldercare, where residents have complex vulnerabilities and multiple organisational structures to coordinate. This case study aims to provide insights into both how long-term oxygen therapy can be equitably sustained in one of the oldest populations in the world, but also how care home settings should be considered in pandemic preparedness and response planning.

Country Context

Demography, economy and epidemiology

In Sweden, the leading cause of death is circulatory diseases which accounted for one third (33%) of all deaths in 2018. Of these, 12% were due to ischemic heart disease and 6% caused by stroke. In the same year, cancers accounted for one quarter (25%) of all deaths, of which lung cancer (4%) was the most common. Two in five (38%) Swedish adults have at least one chronic condition and this increases with age: among adults aged over 65 years, 55% have one or more chronic conditions.[1] However, Sweden is below the EU average for avoidable hospital admissions due to chronic illnesses (including COPD), reflecting functional outpatient and community care structures, and overall lower burden of these illnesses than other high-income settings.[1]

In 2019, one third (34%) of all deaths were attributed to behavioral risk factors of which tobacco accounted for 15%. Air pollution, defined as fine particulate matter and ozone exposure, accounted for only 1%.[1] Sweden has one of the oldest populations in the world, with an average life expectancy of 83 years. One in five (20%) adults are aged 65 years and over. Subsequently there is a high and increasing demand for health and social care, as well as medical treatments for chronic conditions, to meet the health and care needs of an ageing population.

Indicator	Value	Data source (year)
Total population	10.6 million	UNPF (2023)
Total under-five population	576,367	SCB (2023)
Under-five mortality	2 deaths per 1,000 live births	UNICEF (2022)
Life expectancy (m:f)	82 years: 85 years	UNPF (2023)
GDP	65,84 thousand US Dollars per capita as at first quarter of 2023	IMF (2023)
Healthcare expenditure	6,914.91 USD per capita (in 2021)	The World Bank (2021)
Income status	High income country	The World Bank (2023)
COPD mortality	22.9 deaths per 100 000 inhabitants (in 2021)	Statista (2021)

Table 1. Summary of Sweden's demography, economy and epidemiology

Health system

Sweden is a welfare state in which health and social care are largely tax funded and publicly organized and provided. The guiding principle is to make services available to anyone in need of care, regardless of economic status. Sweden's universal care system is decentralized with 21 regions which locally collect taxes on income to cover costs for primary healthcare, specialized healthcare and rehabilitation. This is then further decentralized to 290 municipalities which oversee prevention and health promotion, and social care primarily for older people (Table 2). The State's role is typically to decide on policy aims and directives through legislation and financial incentives, which regions and municipalities then enact, and the State provides smaller contributions to the funding of health and social care. Small out-of-pocket fees for outpatient care apply to adults under 85 years (approximately 15-50 USD per visit). Through policy and funding, the State also influences health promotion and prevention that is delivered largely by municipalities, with care for elderly being an example of this. Municipalities are (by

law) not employing physicians, and municipalities therefore need to allocate physicians to the care they organize – including elderly care homes. In the last few decades, older people in need of both healthcare and social care have been moved to care homes. More recently however, care homes are being replaced by home-based social and health care.[2]

	Sweden		
	State	Region	Municipality
**Specialized healthcare**			
Policy	X		
Funding	X	X	
Organizing		X	
**Primary healthcare**			
Policy	X	X	
Funding	X	X	
Organizing		X	
**Prevention and health promotion**			
Policy	X		X
Funding	X		X
Organizing		(X)	X
**Rehabilitation (medical)**			
Policy	X		
Funding		X	
Organizing		X	
**Social care for older people**			
Policy	X		
Funding	X		X
Organizing			X

Table 2. Areas and levels of responsibilities for health and social care for older people in Sweden[2]

Whilst the care system is universal, it is supplied by both public and private providers, and each patient has the right to choose their primary care clinic and social care provider. Private care providers run 15 of the country's 100 hospitals and 45% of the 1,200 primary care clinics, primarily in urban areas, and 23% of the social care services. The private companies are allocated tax money to provide the care, with no differences in out-of-pocket payments incurred by patients between public and private care providers.[3,4] There is a wide variation in the proportion of elderly care hours provided by private care providers between regions, ranging from 62% in Stockholm region to 2% in Norbotten region in 2016.[4]

In Sweden, medical oxygen is classified as a medical treatment that requires advanced medical healthcare. All medical treatments used in Sweden are approved by the independent government agency the Swedish Medical Products Agency. The process of approving medical treatments are regulated in law (reference: 2015:315). Medical oxygen is approved for use as a treatment for multiple diseases and conditions, with policies regarding what diseases to be treated based on research and best practice and developed and regularly revised by the National Board of Health and Welfare and the Swedish Medical Products Agency. Similar to the Swedish Medical Products Agency, the National Board of Health and Welfare is an independent

government agency that licenses healthcare workers, develops policy and guidelines, and disseminates information to other agencies, professionals and the general public. The National Board of Health and Welfare also maintains some of the national health data registries and official statistics.[5] The Ministry of Health and Social Affairs is responsible for delivering social welfare, public health, healthcare and care for the elderly. Within this Ministry, the unit for healthcare and medical products has the function of compiling information, including medical oxygen, to provide guidance to the government.[6]

According to the Swedish Healthcare Act (reference: 2017:30), healthcare providers are obliged to have capacity for providing medical equipment needed according to the Swedish Medicine Agencies regulations, including medical treatment such as oxygen. The policy guideline HSLF-FS 2023:32 developed and distributed by the National Board of Health and Welfare covers administration and prescription of medical oxygen, and policy LVFS 2003:11 refers to medical products approved by the Swedish Medical Products Agency.[7]

COVID-19

Sweden, and particularly the capital, Stockholm, was hard hit by the COVID-19 pandemic from March 2020 onwards. By 23 June 2023, there had been 622,903 confirmed cases of COVID-19 in Region Stockholm – a population of approximately 2.4 million people. As of the 11th September 2023, there had been 19,475 deaths nationwide of which 2,133 deaths occurred in Stockholm municipality.[8] By February 2023, 88% of the adult population (18 years and over) had received at least one dose of COVID-19 vaccine. Vaccine coverage is the highest among the oldest: 97% of adults aged 80 years and over have taken at least one dose of COVID-19 vaccine.[9]

In Sweden, the response to the COVID-19 pandemic differed from that of many other countries by having recommendations rather than regulations and lockdowns. The recommendations aimed at limiting the spread of the virus in society by keeping a physical distance to other people, focusing on hand hygiene, and specific recommendations aimed at shielding vulnerable populations. Due to the high mortality risk, those aged 70 years and over were strongly urged to maintain social distancing, and visits to care homes for older adults were banned during certain time periods.[10] There were widespread reports of care home staff lacking basic PPE equipment and challenges around staff illness, shortages and gaps in training.[11,12] These were given as reasons for the high COVID-19 incidence and mortality within care homes, particularly in Stockholm, and when compared to neighboring Nordic countries.[13]

Sweden rapidly increased their intensive care unit (ICU) bed capacity, and while there were examples of capacity being stretched, they were never overwhelmed.[14] This may in part be explained by different approaches to clinical management, especially in the elderly population. Firstly, Sweden had an oxygen saturation threshold of ≤90% for commencing oxygen treatment in COVID-19 patients within their clinical guidelines - lower than many other high-case burden countries, where cut-offs up to ≤95% were used.[15] Secondly, there were multiple reports in popular media and from personal testimonials that treatment of COVID-19 amongst care home residents should focus on symptom relief and palliative care, rather than admission to hospital for oxygen therapy or intensive care.[12,16,17] While arguments around the need for oxygen and the appropriateness of oxygen treatment amongst these frail patients were raised,[16,18] guidelines for pulse oximetry use for hypoxaemia detection in this setting were not found.

Oxygen supply and clinical use landscape in Sweden

Medical oxygen supply

In the early 2000s, there were national negotiations followed by agreements on medical oxygen supply with mainly two operators: Linde and Air Liquide. These large international providers mainly supply oxygen to the industrial market and the medical oxygen market forms only a small proportion of the oxygen they deliver in Sweden. The companies each provide about 50% of the medical oxygen consumed in the country. The operators are responsible for delivering, re-filling and replacing, and collecting empty oxygen tanks and supplying and repairing concentrators. Despite efforts to obtain information on these contracts and the volumes of oxygen delivered through freedom of interest requests, we were unable to interpret or discern the meaning of the information provided. In recent years, the operators reported that the request for portable medical oxygen has increased – triangulating with national register data on longer-term oxygen need (see Figure 1). Nonetheless, the procedure for the delivery and collection of oxygen tanks and concentrators of different sizes was described by the industrial key informant as the same i.e. concentrators are collected and returned to hospitals or pharmacies. The oxygen providers offer training to pharmacy staff in how to handle medical oxygen.

In addition, Sweden exports medical oxygen. In 2022 Sweden was estimated to have exported 8.5 million cubic meters of bulk liquid oxygen – mostly to Denmark and Norway and was the 18th largest exporter of concentrators and ventilators by market value globally.[19]

Clinical prescribing

In Sweden, all use of medical oxygen by individuals is regulated and requires a medical prescription. Medical oxygen is purchased and paid for by the regions and ordered online by clinical staff using the region's website. Oxygen equipment is collected at a local pharmacy or delivered to the individual by healthcare workers. Users are required to return the concentrators to the pharmacy after use. Within clinical units that prescribe long-term oxygen therapy for home use,[20] a specific cadre of nurse or technician specialized oxygen nurse, or oxygen technician, is responsible for the set-up and follow-up of patients as described by Ekstrom et al. below.

> "*When a patient fulfills LTOT [long term oxygen therapy] criteria and is identified as eligible by the responsible specialist, contact is established with the specialized oxygen nurse(s) at the responsible clinical unit who manages the practical aspects of treatment and follow-up. The oxygen nurses in most cases hand over the oxygen equipment and information to the patient in the home, check the home condition regarding risk factors for fire including smoking, gas stove, and open fireplaces, and arranges for home adaptation by an occupational therapist as needed*" (Ekstrom et al., 2017)[21]

Monitoring outpatient oxygen use – Swedevox

In 1987, a national register of patients receiving long term oxygen therapy (LTOT) was established – Swedevox, and was expanded to include those on long term mechanical ventilation in 1996, and CPAP for the period of 2010 - 2021.[22] The register includes approximately 85% of patients receiving LTOT in Sweden and as of end 2022, 30745 patients have been included in the Swedevox register, from 48 clinical units that are able to prescribe LTOT. The absolute number of patients receiving LTOT has been slowly increasing, and more recently with a dramatic rise in

2021-2022 (Figure 1). The age of starting LTOT has increased from 66 years in 1987 to 76 years in 2022.[23] Reasons given for the more dramatic increase in 2021-2022 is prescriptions for oxygen during exertion/exercise following COVID-19 – although evidence to support this treatment is questioned by the Swedevox report, and the COVID-19 pandemic was also linked to poorer reporting practices for the 2021-2022 period.[23] The background trend of increasing LTOT prior to COVID-19 likely reflects the concurrently ageing and growing population of Sweden.

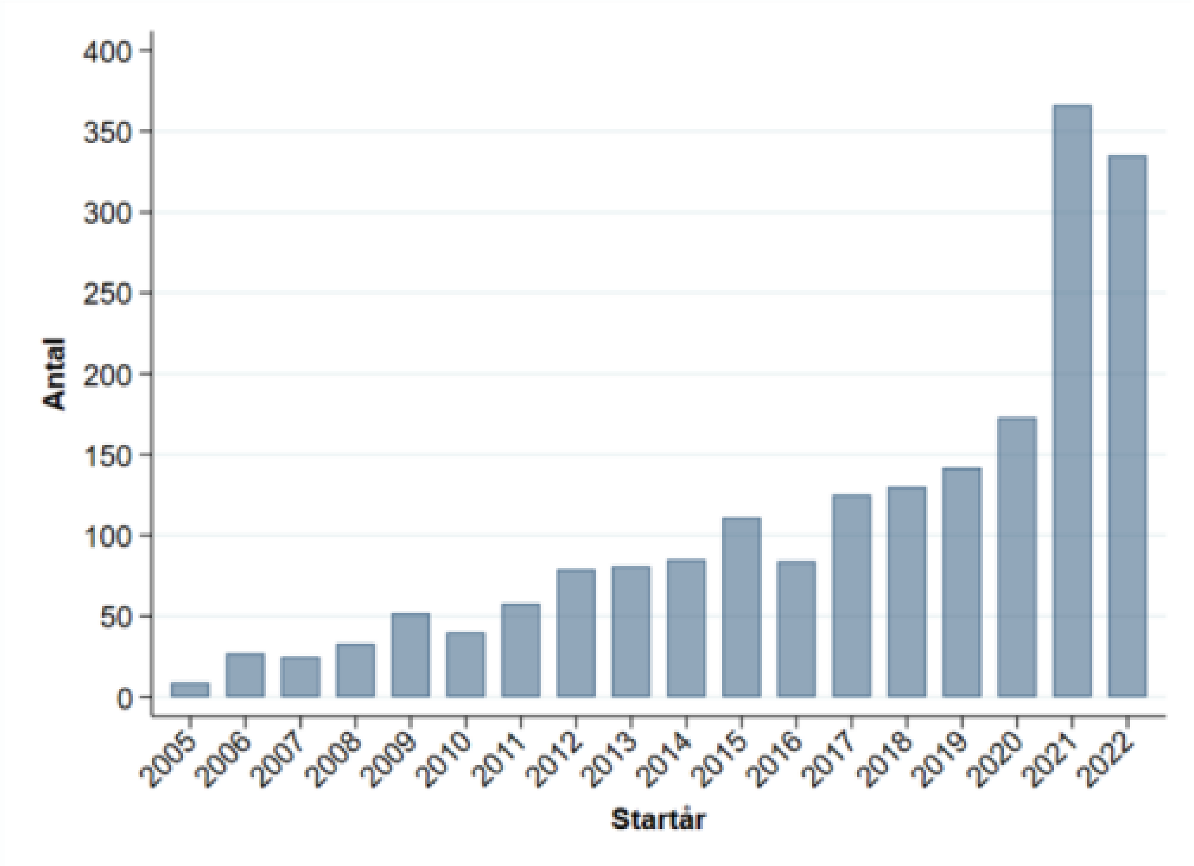

Figure 1: Trend in long term oxygen therapy prescriptions in Sweden from the 2022 Swedevox Annual Report (antal = number; startår = starting year)[23]

At the time of establishment, 85% of LTOT was being delivered by oxygen concentrators, with the National Lung Organisation responsible for supervising delivery of equipment by commercial companies.[24] The predominance of concentrators has remained and has been shown as considerably cheaper than the alternative of liquid oxygen for these patients,[25] although associated with more adverse effects on quality of life.[26] The Swedevox register has proven useful in monitoring long-term trends in home-based oxygen use,[21] as well as evaluating policy impacts on oxygen need, how evidence is being put into clinical practice, and oxygen safety in non-clinical settings.[27,28] It has also highlighted regional variations in prescription practices – especially for mechanical ventilation in palliative care.[23]

Access to medical oxygen for an ageing population

Oxygen stakeholders in Sweden

As healthcare is publicly provided there is no private market for medical oxygen in Sweden. Key stakeholders include the responsible ministry and public agencies such as the Ministry of Health and Social Affairs, the National Board of Health and Welfare, and the Swedish Medical Products Agency. According to those interviewed, in Sweden, medical oxygen is not on the political agenda and there is no lobbyism, yet the market has been dominated by two large suppliers for an extended period. The Ministry of Health and Social Affairs has confirmed that there are no discussions or ongoing work around medical oxygen. Sweden has an active research community in the clinical use of medical oxygen and generating evidence for clinical guidelines both locally and internationally.[23]

> *"Currently there is very little discussion about medical oxygen partly because the option [to provide medical oxygen] exists and that COVID-19, which caused the debate, and vaccination… one has gained more knowledge about treatment of COVID-19 and so such discussion is little nowadays."* Medical doctor working with multiple care homes for older adults.

Effective public health can reduce the need for long-term oxygen therapy

Overall, the need for medical oxygen among older adults in care homes is low. According to a medical doctor with management responsibilities for about 40% of the 400 care homes across Stockholm, it is very unusual that older adults residing in care homes need medical oxygen. A couple of medical doctors further said that medical oxygen was rarely used during the pandemic for this patient group, referring to research that showed breathlessness was found in 73% of patients dying in hospitals but only in 35% of older adults dying in care homes. The authors posited that breathlessness was less common in those who died in care homes since they mostly died within the first 7-8 days of the disease that is characterized by fever, cough, and pronounced fatigue, but not by pneumonia. The paper concluded that many older adults who died in care homes passed away before COVID-19 had entered their lungs and acute respiratory distress syndrome had developed.[18] The implication of this being that oxygen was not needed.

> *"There is research concluding that they [older adults in care homes with COVID-19] were too frail. There was a difference between healthy older adults who died from having their lungs infected by the coronavirus and those frail who died because they were close to death anyway as they were so frail, very little was needed, they never got particularly ill before they died."* Medical doctor specialized in geriatrics.

However, relying on breathlessness as an indicator of hypoxaemia, and therefore oxygen need, was questioned – and doesn't align with the commonly reported phenomenon of 'happy hypoxaemia' occurring in patients without other signs of respiratory distress. As one frontline care worker expressed:

> *"Could we give oxygen? I started to think … I myself did not have problem with breathing, only once I felt that it was difficult to breathe. Then we discovered that many did not have good oxygen saturation even though they did not have difficulty with breathing. They die in silence. That's when we started to use the pulse*

oximeter much more at the care home and began to think why we weren't allowed to administer oxygen" (Kabir et al., 2020)[12]

According to Air Liquide, one of the two main suppliers of oxygen to the Swedish care sector and a main supplier to other Nordic countries, Denmark has three times as many individuals in need of medical oxygen as Sweden. Yet the total population of Sweden is twice as large as the population of Denmark. This is probably explained by different trends in smoking,[23] only 6% of Swedish adults smoke cigarettes daily yet snus, a smokeless, moist powder tobacco product, is commonly used.[29] Regular smoking for a long time increases the risk of lung diseases such as COPD that can require medical oxygen in older age.[30] Both the Swedevox registry and Air Liquide report links the proportion of smokers in the 1950s and 60s with today's medical oxygen demand,[23] with Danes starting smoking cigarettes a decade later than Swedes, and younger generations being less likely to smoke. Similar to the Swedevox registry, Denmark also established a medical oxygen register in 1994, allowing long-term tracking of home-oxygen use and outcomes.[31] Interestingly, the oxygen usage data is provided by the oxygen suppliers and linked to patient medical records – providing a nice example of industry collaboration for monitoring oxygen demand. A key difference between initiating LTOT in Denmark and Sweden is the role of smoking – in Sweden it is a counter-indication, also possibly contributing to the lower prevalence of LTOT prescriptions.[28] This has highlighted a safety concern, with higher numbers of burn injuries in this group in Denmark compared to Sweden.[28]

This re-emphasizes that the adage "prevention is better than cure", or rather in this case "...better than treatment" should also be integrated into oxygen policy and planning. Nonetheless, even if the demand for medical oxygen to patients in home-based care or care homes has decreased compared to neighboring countries and remained low until very recently,[23] the demand of medical oxygen in hospitals was reported to have remained the same.

Decentralization can create and exacerbate inequities

In non-pandemic circumstances, those in need of medical oxygen have it prescribed in hospital by specialist units, and in some cases will need to go to the hospital to receive their medical oxygen treatment. However, in some regions, medical staff from the hospital are part of a team trained in delivering advanced medical care in the home setting and can therefore provide medical oxygen in the patient's home - this mechanism was particularly common during the COVID-19 pandemic. In rural areas of Sweden, distances to the nearest hospital are long, sometimes making it challenging and tiresome for the patient when advance care teams are not present.

"We have to trust the home help providers [who visit older adults more often than the home care nurse] to report on anything that's not normal [regarding the patient's oxygen supply], which has worked so far." Medically responsible nurse operating in a rural area.

Similarly, older adults who live in a care home and have been prescribed medical oxygen may have it delivered by a member of staff from the advanced medical home care team. Whilst care home nurses may have a background in advanced healthcare and are trained in caring for patients with medical oxygen, this is not universal across staff. Limited competence and capacity to provide medical oxygen in the older patient's home raises questions on inequity and possible weaknesses to the care system that may negatively influence the individual's life satisfaction.

These differences between regions could be explained by the decentralized system. Some regions also provide additional services such as mobile healthcare teams that conduct out-reach for urgent calls and carry smaller medical oxygen cylinders that can be provided to patients residing in their own homes or care homes while they wait for the ambulance to arrive. In Stockholm for example, since COVID-19 medical staff from the advanced home healthcare teams have continued in their role of providing medical oxygen in care homes and in patients' homes; however, it is unclear whether this will continue in the long term. This has generated discussions on how to ensure that medical oxygen can be provided rapidly to older adults in care homes, and how to ensure this is done equitably given the coordination needed between regions and municipalities. In particular, in other parts of Sweden, the difference between having to undertake long journeys to hospital for medical oxygen treatment versus providing such treatment in the home is of importance to the individual.

Delivering medical oxygen without medicalizing the setting.

As mentioned previously, Sweden has one of the oldest populations in the world. In the last few decades, the concept of 'ageing in place' has guided policies around where care for older adults should happen. 'Ageing in place' refers to the idea that older people should remain living at home for as long as possible. Subsequently, the number of beds in hospitals and, particularly, long-term care institutions have been dramatically reduced and the proportion of older adults who remain living at home has increased.[32] Today, a place in a care home is usually not offered until the older person's social care needs exceed what can be reasonably be offered by carer visits, and therefore care home residents in Sweden tend to be very frail with multimorbidity and often have dementia. They need help to undertake everyday activities, with nurse assistants and social care workers running 24-hour services. Nurses are onsite during the daytime, and a medical doctor undertakes routine visits weekly or more often depending on the older adults' medical needs. The poor health of care home residents was reported by several interviewees, including the difficulty in providing medical oxygen treatment to someone with dementia.

Before a major national care reform in the 1990s, medical oxygen was supplied to older adults in long-term care through piped oxygen, with outlets by each bedside. Today this is very unusual and has mainly been replaced by oxygen concentrators prescribed to the individual and provided on-site or in hospital. This also applies to independent individuals. Hence, today there is no difference in how oxygen is supplied to individuals residing in care homes or to individuals living in their own homes. Generally, spare or back-up medical oxygen is not stored in care homes as only individuals who have been prescribed medical oxygen receive it. Several of those interviewed felt this system was good given the risks associated with storing oxygen, such as explosions. It was also mentioned that the home-like environment of today's care homes is highly valued by the older residents and medical oxygen supply in each care home apartment would make the environment more hospital-like. Some medical doctors interviewed elaborated that it would be hard to motivate the costs of having oxygen stored in care homes, given they did not anticipate it would be used often.

> *"Sometimes patients don't want to leave the care home to go to the hospital for care, and in such situations we try to provide the care here, including for example providing medical oxygen. Trying to meet the patient's wishes is incredibly important. But having it [medical oxygen] stored in the care home just because someone may need it every other year, one could discuss how resource efficient that would be."* Medical doctor working with multiple care homes for older adults.

During COVID-19 a key challenge raised was the multiple different private providers that are contracted by the municipalities to provide home-based and care-home services – with shortfalls in coordination, communication and infection control.[33,34] The intention to maintain a homely environment, despite residents having complex medical needs, may explain why these settings were largely overlooked in Sweden's pandemic preparedness planning:

> *"In the Swedish pandemic plan eldercare in general was noted but not further elaborated upon. The level of elaboration in the plan does not correspond to the organizational and systemic intricacies of eldercare, in terms of municipal and regional variations and roles of national authorities, as well as the need for cooperation due to a complex structure"* (Rapeli et al., 2023)[35]

Challenges in providing high flow oxygen in the hospital setting.

According to the informant from Air Liquide, the challenge has never been to produce enough oxygen - not even during the COVID-19 pandemic, but to have it delivered all the way to the patient. They stated that the oxygen pipelines in hospitals, and historically those in care homes, are very narrow and restrict the flow. This was identified by them as a problem during the pandemic as several patients simultaneously required a very high flow of oxygen. Air Liquide reported portable cylinders and concentrators to be inadequate to meet the demand during the pandemic and at some hospitals they provided an additional bulk storage tank next to the regular tank outside the building. Notably, a study from a regional hospital in Sweden reported a mean flow rate of 3L/min – considerably lower than the WHO estimated rate of 10L/min, and within the capacity of most concentrators to deliver.[36] However, this provides a useful lesson for pandemic preparedness, suggesting that planned treatment centres, surge facilities and settings with high numbers of ICU numbers should have a physical environment that supports surge capacity solutions (e.g. safe location for additional bulk liquid oxygen storage tanks, or the ability to upgrade pipelines for higher flow rates).

The informant at Air Liquide further reported that when there is a crisis, the medical oxygen suppliers prioritize delivery of medical oxygen over industrial oxygen. This was not considered a challenge as only a small amount of the oxygen produced is medical oxygen. The informant at the oxygen supplier further reported that in such situations hospitals are prioritized. Therefore, the hospital setting needs to be considered in addition to the care home and home care settings.

> *"In a crisis, hospitals are prioritized. The industry... a paper mill, consumes in one month [the same amount of oxygen as] what a hospital consumes during the entire year. So that's not a problem. The dimensions... a paper mill can stop its production. Hospitals are prioritized, yes they are."* Industry employee

Dividing the oxygen supplied between different settings could help. The informant at Air Liquide explained that in Norway, during the pandemic, oxygen tanks were placed at hospitals whereas portable oxygen was primarily given to those who live in geographically hard to reach areas, or who are unable to be transported to hospital. However, this would require both capacity in terms of enough healthcare staff (during COVID-19 many healthcare staff were ill too) and the right competence of such staff. Some informants thought that increased staff competence is always a positive thing yet referred to other areas where staff competence is lacking such as palliative care and dementia – areas of greater interest and importance to themselves. This may illustrate the competition medical oxygen seems to be facing if put on the agenda.

Key messages

- Older adults in need of medical oxygen should, if possible, receive medical oxygen where they reside. Thus, portable medical oxygen solutions that are tailored to different use cases (e.g. mobile advance care teams, or LTOT at home) are key to ensuring oxygen access for an ageing population.
- Strengthening staff capacity and competence in care homes, where staff often consist of a multi-disciplinary mix of clinical and non-clinical cadres, to provide medical oxygen is important to be able to deliver medical oxygen where the patient resides.
- Given home and care home-based settings are complex, and include complex patients, ensuring all frontline workers are incorporated into pandemic preparedness planning is needed – and was criticized as a gap in the COVID-19 response.
- As all citizens have equal rights to care irrespective of where they live, providing care in relation to the patient's wishes and where they reside also means needing to have the capacity to offer the same alternatives to those who live in rural areas.
- Smoking prevention initiatives are important to reduce long-term oxygen needs as populations age.

Additional methods information:

Desk-based review:

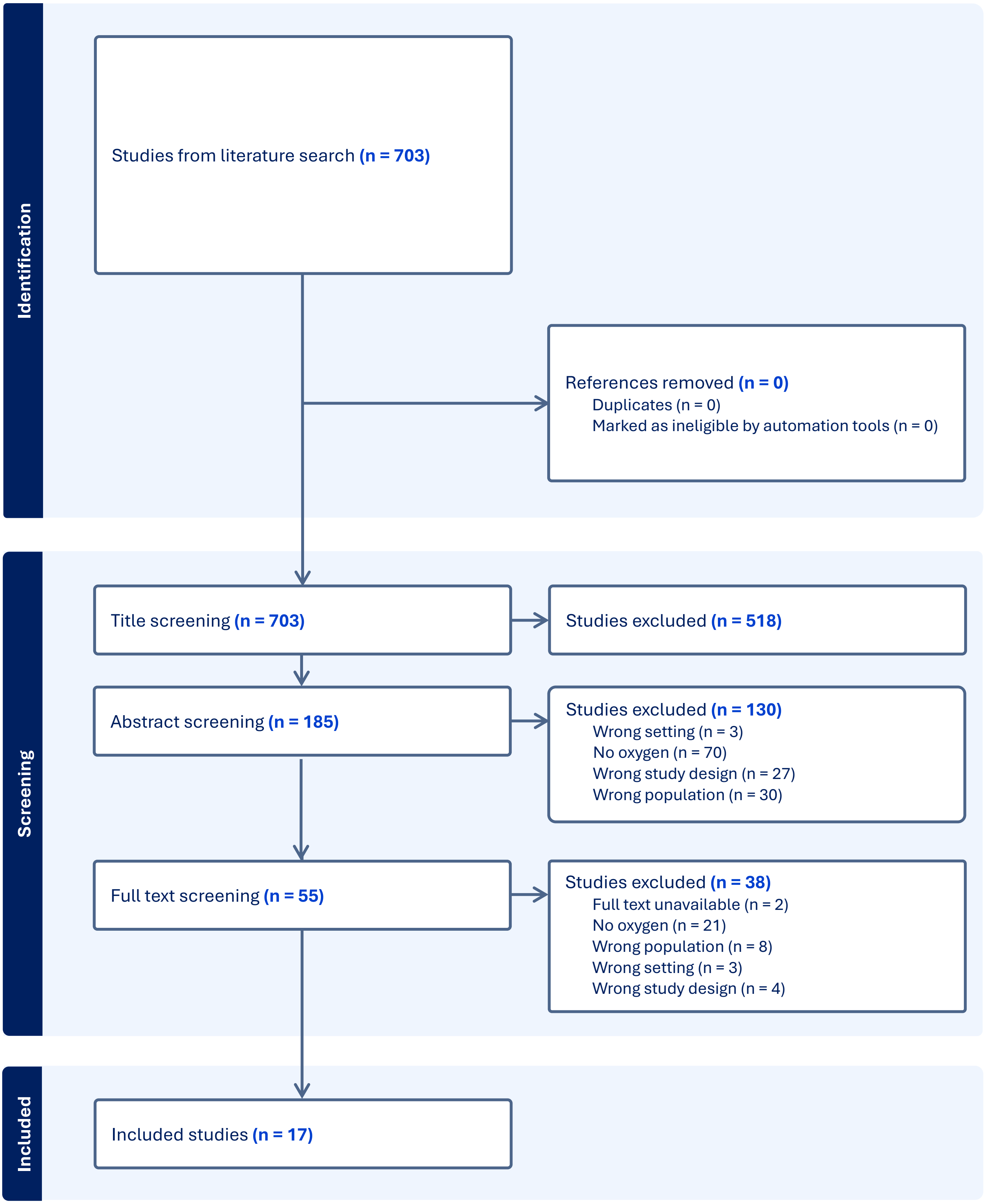

Sources of grey literature included searching for information on the following organization webpages between May and September 2023:

Organization	Description	Website
1177	Official website on information about illnesses, care and health	www.1177.se
Andningssviktregistret Swedevox	The national register for patients on long-term oxygen therapy	https://www.ucr.uu.se/swedevox/
Astma- och allergiförbundet	The Asthma and Allergy Association	https://astmaoallergiforbundet.se/
Linde	A major oxygen supplier	www.linde-gas.se
Tandvårds- och läkemedelsförmånsverket (TLV)	Government agency on dental and pharmaceutical benefits	www.tlv.se
Sveriges Kommuner och Regioner (SKR) Läkemedelskommittéer	Committees for medical drugs at the Swedish Association of Local Authorities and Regions	https://skr.se/skr/halsasjukvard/vardochbehandling/lakemedelkommunerregioner/kontaktuppgifterlakemedelsfragor/lakemedelskommitteer.1934.html
Vårdhandboken	Guidelines on healthcare and medicines	www.vardhandboken.se

Table 3: List of webpages included in the grey literature search

Key informant interviews:

Individuals approached for this project were purposively sampled based on their expertise. Only very limited information can be provided about them to avoid the identification of these individuals. An additional two medical doctors who work on the organization of care reported briefly on the distribution of medical oxygen via email. Contact with a civil servant at the Government Agency on Dental and Pharmaceutical Benefits did not generate sufficient information to estimate provision/consumption of medical oxygen. Five people/organizations/companies contacted did not respond to our requests for interviews despite multiple attempts to contact them via email and by phone.

Civil servant at the Ministry of Health and Social Affairs
Coordinator of health and social care at an employer organization
Medical doctor specialized in geriatrics
Medical doctor specialized in geriatrics and advanced healthcare in the home setting
Medical doctor specialized in palliative care
Medical doctor working with multiple care homes
Member of the Corona commission
Member of staff at oxygen supplier Air Liquide
Medically responsible nurse

Table 4: List of interviewed key informants

References

1 OECD, European Observatory on Health Systems and Policies. State of Health in the EU: Sweden Country Health Profile 2021. Brussles, 2021 https://health.ec.europa.eu/system/files/2021-12/2021_chp_sv_english.pdf (accessed Jan 10, 2024)

2 Agerholm J, Pulkki J, Jensen NK, *et al*. The organisation and responsibility for care for older people in Denmark, Finland and Sweden: outline and comparison of care systems. *Scand J Public Health* 2023; : 140349482211371.

3 International Trade Administration. Healthcare Technologies Resource Guide - Sweden. https://www.trade.gov/healthcare-resource-guide-sweden (accessed Jan 12, 2024).

4 Jacob Öljemark. Äldreomsorg i privat regi. EkonomiFakta. 2023; published online Aug 18. https://www.ekonomifakta.se/Fakta/Foretagande/offentlig-sektor/aldreomsorg-i-privat-regi/ (accessed Jan 12, 2024).

5 Tikkanen R, Osborn R, Mossialos E, Djordjevic A, Wharton GA. International Health Care System Profiles: Sweden. New York, USA, 2020 https://www.commonwealthfund.org/international-health-policy-center/countries/sweden (accessed Jan 12, 2024).

6 Regeringskansliet. Socialdepartementets organisation. 2021; published online Nov 22. https://www.regeringen.se/sveriges-regering/socialdepartementet/socialdepartementets-organisation/ (accessed Jan 12, 2024).

7 The Care Manual. Oxygenbehandling: Referenser och regelverk. 2022; published online May 2. https://www.vardhandboken.se/vard-och-behandling/lakemedelsbehandling/oxygenbehandling/referenser-och-regelverk/ (accessed Jan 12, 2024).

8 Swedish National Board on Health and Welfare. Statistics on COVID-19. 2022; published online Oct 21. https://www.socialstyrelsen.se/en/statistics-and-data/statistics/statistics-on-covid-19/ (accessed Jan 11, 2024).

9 Public Health Agency of Sweden. COVID-19 statistik och analyser. Bekräftade fall i Sverige. 2023; published online Dec 22. https://www.folkhalsomyndigheten.se/smittskydd-beredskap/utbrott/aktuella-utbrott/COVID-19/statistik-och-analyser/bekraftade-fall-i-sverige/ (accessed Jan 11, 2024).

10 Tegnell A. The Swedish public health response to COVID-19. *APMIS* 2021; **129**: 320–3.

11 Bergqvist M, Bastholm-Rahmner P, Gustafsson LL, *et al*. How much are we worth? Experiences of nursing assistants in Swedish nursing homes during the first wave of COVID-19. *Int J Older People Nurs* 2023; **18**. DOI:10.1111/opn.12498.

12 Kabir ZN, Boström A-M, Konradsen H. In Conversation with a Frontline Worker in a Care Home in Sweden during the COVID-19 Pandemic. *J Cross Cult Gerontol* 2020; **35**: 493–500.

13 Sepulveda ER, Stall NM, Sinha SK. A Comparison of COVID-19 Mortality Rates Among Long-Term Care Residents in 12 OECD Countries. *J Am Med Dir Assoc* 2020; **21**: 1572-1574.e3.

14 Paterlini M. Covid-19: Sweden considers tougher restrictions as ICU beds near capacity. *BMJ* 2020; : m4833.

15 Mansab F, Donnelly H, Kussner A, Neil J, Bhatti S, Goyal DK. Oxygen and Mortality in COVID-19 Pneumonia: A Comparative Analysis of Supplemental Oxygen Policies and Health Outcomes Across 26 Countries. *Front Public Health* 2021; **9**. DOI:10.3389/fpubh.2021.580585.

16 Nilsson L, Andersson C, Sjödahl R. COVID-19 as the sole cause of death is uncommon in frail home healthcare individuals: a population-based study. *BMC Geriatr* 2021; **21**: 262.

17 Brusselaers N, Steadson D, Bjorklund K, *et al*. Evaluation of science advice during the COVID-19 pandemic in Sweden. *Humanit Soc Sci Commun* 2022; **9**: 91.

18 Strang P, Bergström J, Lundström S. Symptom Relief Is Possible in Elderly Dying COVID-19 Patients: A National Register Study. *J Palliat Med* 2021; **24**: 514–9.
19 World Integrated Trade Solutions. Medical ventilators; CPAP; BiPap; Oxygen concentrators; (901920) exports by country in 2022. 2024; published online Jan 12. https://wits.worldbank.org/trade/comtrade/en/country/ALL/year/2022/tradeflow/Exports/partner/WLD/nomen/h5/product/901920 (accessed Jan 12, 2024).
20 Ekström M, Andersson S, Emtner M, *et al.* Riktlinjer LTOT i hemmet: Nationellt kvalitetsregister för långtidsbehandling med oxygen (LTOT), respirator (LTMV) eller CPAP. 2021 https://www.ucr.uu.se/swedevox/behandlingsriktlinjer/dokumentation/riktlinjer-oxygen-2021-2/viewdocument/568 (accessed Jan 12, 2024).
21 Ekström M, Ahmadi Z, Larsson H, *et al.* A nationwide structure for valid long-term oxygen therapy: 29-year prospective data in Sweden. *Int J Chron Obstruct Pulmon Dis* 2017; **Volume 12**: 3159–69.
22 About Swedevox. 2024. https://www.ucr.uu.se/swedevox/about-swedevox (accessed Jan 12, 2024).
23 Ekström M, Palm A, Hegardt F. Årsrapport från Andningssviktregistret 2022: Andningssviktregistret Swedevox Nationellt kvalitetsregister för långtidsbehandling med oxygen (LTOT), respirator (LTMV) eller CPAP. 2023.
24 Fauroux B, Howard P, Muir J. Home treatment for chronic respiratory insufficiency: the situation in Europe in 1992. The European Working Group on Home Treatment for Chronic Respiratory Insufficiency. *European Respiratory Journal* 1994; **7**: 1721–6.
25 Andersson A, Strom K, Brodin H, *et al.* Domiciliary liquid oxygen versus concentrator treatment in chronic hypoxaemia: a cost-utility analysis. *European Respiratory Journal* 1998; **12**: 1284–9.
26 Björklund F, Ekström M. Adverse Effects, Smoking, Alcohol Consumption, and Quality of Life during Long-Term Oxygen Therapy: A Nationwide Study. *Ann Am Thorac Soc* 2022; **19**: 1677–86.
27 Tanash H, Ekström M, Huss F. The risk of burn injury during long-term oxygen therapy: a 17-year longitudinal national study in Sweden. *Int J Chron Obstruct Pulmon Dis* 2015; : 2479.
28 Tanash H, Ringbaek T, Huss F, Ekström M. Burn injury during long-term oxygen therapy in Denmark and Sweden: the potential role of smoking. *Int J Chron Obstruct Pulmon Dis* 2017; **Volume 12**: 193–7.
29 Public Health Agency of Sweden. Tobacco and nicotine products. 2022; published online Aug 22. https://www.folkhalsomyndigheten.se/the-public-health-agency-of-sweden/living-conditions-and-lifestyle/andtg/tobacco/ (accessed Jan 10, 2024).
30 Backman H, Vanfleteren L, Lindberg A, *et al.* Decreased COPD prevalence in Sweden after decades of decrease in smoking. *Respir Res* 2020; **21**: 283.
31 Ringbaek TJ, Lange P. The impact of the Danish Oxygen Register on adherence to guidelines for long-term oxygen therapy in COPD patients. *Respir Med* 2006; **100**: 218–25.
32 Spasova S, Baeten R, Coster S, Ghailani D, Peña-Casas R, Vanhercke B. Challenges in long-term care in Europe - A study of national policies 2018. Brussels, 2018 https://ec.europa.eu/social/main.jsp?catId=738&langId=en&pubId=8128&furtherPubs=yes (accessed Jan 10, 2024).
33 Baxter R, Jemberie WB, Li X, *et al.* COVID-19: Opportunities for interdisciplinary research to improve care for older people in Sweden. *Scand J Public Health* 2021; **49**: 29–32.
34 Fernemark H, Skagerström J, Seing I, Hårdstedt M, Schildmeijer K, Nilsen P. Working conditions in primary healthcare during the COVID-19 pandemic: an interview study with physicians in Sweden. *BMJ Open* 2022; **12**: e055035.
35 Rapeli M, Carlstedt J, Hergeirsdóttir R, Guðmundsson HS, Björngren Cuadra C, Hatakka I. Three Nordic countries responding to COVID-19 – Eldercare perspectives. *International Journal of Disaster Risk Reduction* 2023; **84**: 103442.

36 Hvarfner A, Al-Djaber A, Ekström H, *et al*. Oxygen provision to severely ill COVID-19 patients at the peak of the 2020 pandemic in a Swedish district hospital. *PLoS One* 2022; **17**: e0249984.

THE LANCET Global Health
COMMISSION ON MEDICAL OXYGEN SECURITY

Country Case Study: Uganda

The evolution of the medical oxygen supply chain

Freddy Eric Kitutu[1,2,3] Angella Nabakooza Kigongo[3], Brenda Nakimuli[3], Marion Birungi[3], Freddie Ssengooba[3], Henry Zakumumpa[3]

1. Department of Pharmacy, Makerere University School of Health Sciences, Uganda; **2.** Department of Women's and Children's Health, International Child Health and Migration, Uppsala University, Sweden; **3.** Makerere University School of Public Health, Kampala, Uganda

Case study focus

Medical oxygen systems are complex, covering oxygen production, distribution, storage, and patient delivery. Each of these steps in the supply chain can involve different technologies, multiple public and private actors, and various regulations and standards. Therefore, understanding how these components integrate into an effective and efficient system is critical for ensuring medical oxygen security across the health system. A need more pronounced in a context where the different components and pieces of the desired medical oxygen system are actively put together as they provide medical oxygen to patients who need it. This case focuses on the current state of medical oxygen supply chain at multiple levels of the Ugandan health system while tracing the evolutionary journey taken since the pre-COVID19 period in 2018. It aims to provide insight into whether the recent investments in increasing the countries medical production capacity has translated into improved patient access through a strengthened oxygen system.

Country Context

Demography, economy, and epidemiology

Uganda is a landlocked country in East Africa with a population of approximately 45.9 million based on the 2024 census,[1] and maintains one of the highest population growth rates in the world at 2.9%. The country has achieved substantial gains in economic development and improvements in health outcomes over recent decades, including reductions in under-5 mortality at 52 deaths per 1000 live births, infant mortality at 36, and neonatal mortality at 22 and pregnancy related mortality ratio of 228 deaths per 100,000 live births.[2,3] However, it remains one of the least developed countries in the world with as gross national income per capita of $US 974 in 2022.[4] Poverty levels are high with 20.3% and 41.2% of the population below the national and international poverty line, respectively.[4] Uganda's health system bears a substantial burden of preventable and poverty-related diseases including malaria, HIV/AIDS, and tuberculosis.

In the financial year of 2022/2023, neonatal conditions were the leading cause of health facility deaths across all age groups, accounting for 10.3% of mortality, followed by malaria (7.4%), pneumonia (5.3%), anaemia (3.9%), road traffic injuries from motorcycles and vehicles (2.3%), and septicaemia (1.9%). In the same period, the leading causes of hospital admission were malaria, pneumonia, anaemia, septicaemia, neonatal conditions, road traffic accidents and other injuries, complications of pregnancy and obstructed labour, sickle cell disease and asthma in descending order of magnitude.[4]

These conditions are commonly associated with hypoxaemia (low blood oxygen) and other complications for which prompt detection and management with pulse oximetry and oxygen therapy is a basic standard of care.[5,6] Low oxygen levels in the blood, occurs in various acute and chronic disease conditions throughout the life course, [5,6] significantly contributing to their severity and mortality. It affects patients regardless of age, sex, aetiology, or geographical region. Over the last three fiscal years, the major causes of institutional deaths have not significantly changed. However, despite remaining as the leading cause of mortality, there's been a notable 36.8% decrease in absolute deaths from neonatal conditions, dropping from 5,899 in 2021/22 to 3,730 in 2022/23. This improvement has been attributed to focused interventions, such as the perinatal death notification and reviews, establishment of Neonatal Intensive Care Units (NICUs), and a mentorship program.[4]

Indicator	Recent value	Data source (year)
Total population	45,791,461	Uganda Bureau of Statistics (2022)
Total under-five population	7,921,923	Uganda Bureau of Statistics (2022)
Under-five mortality rate	52/1,000 live births	Uganda DHS (2022)
Maternal mortality ratio	189/100,000 live births	Uganda DHS (2022)
Life expectancy at birth (m:f)	60:65	World bank (2021)
GDP	$US 45.6 billion	World bank (2023)
Healthcare expenditure per capita	$US 22	WHO (2022/2023)
Income status (income per capita)	$US 1046	Uganda Bureau of Statistics (2022)
Gross National Income per Capita	$US 840	World Bank (2021)

Table 1: Description of key population health indicators in Uganda. DHS = Demographic and Health Survey; $US = US dollars

Health system

Uganda has a decentralized health system, comprised of both public and private health providers. The Ministry of Health is responsible for setting policies and strategic direction, while Local Governments are responsible for service delivery. The Districts and Health Sub-Districts are responsible for leadership in the planning and management of health services, supervision and quality assurance, provision of technical, logistic and capacity development support. Uganda's has a tiered health system based on the health services scope they provide and the catchment area they are intended to serve. On the lower rung of the formal health system are health centre level II to level IV; then General hospital, Regional Referral hospital and National Referral hospital. For the public facilities, the National and Regional Referral Hospitals report to the Central Government; while General Hospitals and Health Centres (II—IV), report to the Local Governments. Uganda has a total of 6,404 Health facilities and special clinics. 48.0% (n=3084) of health facilities are Government owned, 15.0% (947) are private not-for-profit and the remaining 37.0% (n=2373) are private-for-profit facilities. At community level, volunteers chosen within the community by the residents form a village health team that largely provides health promotion services and community sensitization and mobilization.

In terms of financing, there are various mechanisms through which health services are funded in Uganda. These include public expenditure, private expenditures (that primarily consists of out-of- pocket payments and health insurance) and external donor support.[7] Government allocations to health have fallen over time, in 2018/19, the government allocated 7.0% of the national budget to the health sector which is far below the 2001 Abuja declaration target of 15%. From the $US 144 per capita annual budget allocation, approximately US$10 per capita was allocated to the health sector. This allocation to health is also significantly lower than the estimated requirement of US$86 per capita needed to provide essential health services in low-income countries.[8] In 2019, the proportion of the current health expenditure in the country showed that the Government of Uganda was only funding 17.2% of the health sector budget.[7] The low government funding led to gaps in the quality of services provided and as a result, hindered access to services within the public health system. The domestic private health expenditure in the form of out-of-pocket payments and health insurance was at 41.4%.[9] The Parliament of Uganda passed the National Health Insurance bill as a private members bill amidst protests by the Ministry of Health. However, the President refused to assent to the bill and returned it to parliament. Presently, health insurance has a very low penetration amongst the population with approximately 5% of private health expenditure going towards private health insurance schemes. The low insurance coverage is attributed to several reasons; there is no mandatory requirement for health insurance and majority of the population are not afforded the opportunity to benefit from it, those in the informal sector who are the largest population group. Existing health insurance schemes are promoted as employee benefits by a handful of employers.[9]

The National Scale-up of Medical Oxygen Implementation Plan (2018-2022) in Uganda was a substantial initiative aimed at improving the availability and accessibility of medical oxygen across the country.[10] The expiration of this plan marked an important moment to reflect on its accomplishments and the challenges that remain. The plan required a significant financial investment, totalling approximately $US 1.2million (4,509,305,909 Ugandan Shillings). The Ugandan Government committed a substantial portion of this budget, approximately $US 8.6 million (32,669,838,631 Uganda Shillings), demonstrating a strong governmental commitment to healthcare infrastructure and the well-being of its citizens. However, the plan also encountered a notable funding gap of $US 3.1 million (11,839,467,278 Uganda Shillings). This gap highlights the challenges in mobilizing adequate resources for such crucial healthcare initiatives. A successor plan for 2023-2027 has now been published.[11]

COVID-19

Uganda reported its first case of COVID-19 on 21st March 2020. The country thereafter experienced a rapid rise in the number of cases to 52, most of whom were imported cases from international quarantine. By 6th May 2020, the country had recorded 100 cases, most of which (89%) were imported amongst truck drivers. As of June 2021, Uganda was experiencing a second wave of the pandemic and more non-pharmaceutical interventions were reinstated to control spread. By 23rd January 2023, Uganda had registered 171,983 cases and 3,632 deaths. Uganda implemented a number of measures to control the pandemic including imposing lockdowns in March 2020. The events as they unfolded are summarized in Figure 1. The COVID-19 pandemic underscored the insufficient affordability and sustainability of medical oxygen on a national scale, where the extraordinarily high clinical demands at hospitals surpassed the available oxygen supply, leading to preventable deaths. Those challenges were exacerbated by existing issues within the healthcare system, including inadequate human resources, financial constraints, infrastructural deficiencies, and supply chain and logistical problems, an additional reminder that increasing access to medical oxygen to those who need it is a complex problem.

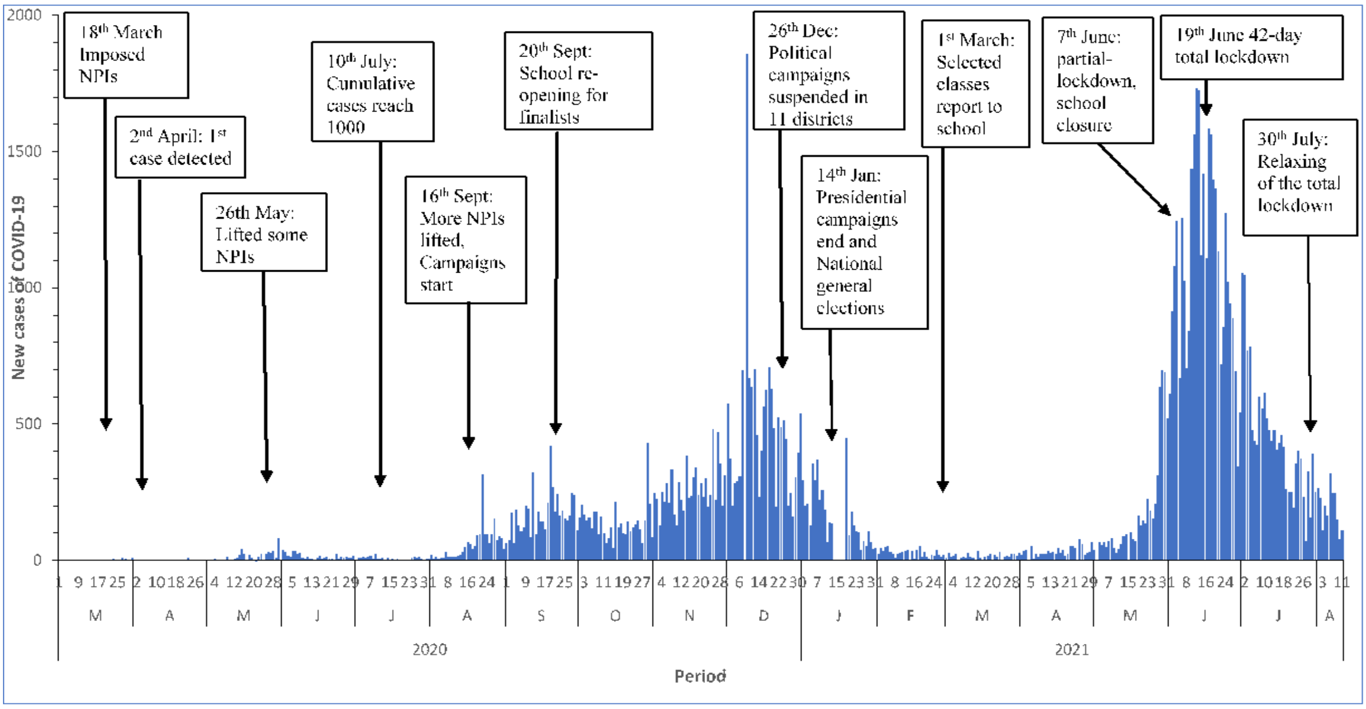

Figure 1: Timeline of key COVID-19 events

The medical oxygen supply chain of Uganda

The Uganda Ministry of Health launched its first National Medical Oxygen Scale-up Plan in 2018. Before this, medical oxygen was not a national priority, and access remained limited. Until 2016, oxygen concentrators were the primary source, leaving patients without high-flow oxygen when demand was high or during power outages.

The 2018 plan introduced a mixed-source model incorporating oxygen concentrators, Pressure Swing Adsorption (PSA) generators, and limited liquid oxygen. Several PSA generators were

installed, a process accelerated by the COVID-19 pandemic. Currently, 33 PSA generators operate nationwide, with capacities ranging from 15m^3/hour to 100m^3/hour. By June 2024, 27 additional PSA generators were in various stages of installation. Two liquid oxygen cryogenic tanks now serve as backup supply. Oxygen concentrators remain part of the national system.

However, 75% of medical oxygen is still supplied via cylinders refilled at PSA plants in regional hospitals, central medical stores, and an accredited private steel industry. Distribution follows a hybrid model: a hub-and-spoke system where cylinders are refilled at regional hospitals and a milkman system where the central stores exchange filled cylinders with empty ones at health facilities.

Multiple sources of medical oxygen

This case study found that at the tertiary level of care, Pressure Swing Absorption (PSA) oxygen generators are a principal source of medical oxygen production, more so in government funded health facilities. The Ugandan Government has established PSA plants at major public Regional Referral Hospitals (RRHs) across the country, with over 17 PSA plants established at the tertiary level of care in Uganda's across major geographic sub-regions.

Interview participants indicated that the public PSA plants established at sub-national level across Uganda are intended as regional supply hubs for lower-level facilities including general hospitals and health centres level IV, III and II. The PSA plants are expected to produce enough medical oxygen to serve the Regional Referral Hospitals where the greatest demand for the medical oxygen is expected, while simultaneously acting as a supply hub for the lower-level facilities within their catchment via oxygen cylinders. RRHs have greater demand for medical oxygen due to having multiple stand-alone care units such as Accidents and Emergency wards, high dependency units and specialized care service units such as intensive care units and oncology units.

For the private not-for-profit sector, the Joint Medical Stores (JMS) - a specialized medicine and health technologies agency founded in 1979 by the Catholic and Protestant churches as a complimentary procurement, warehousing and distribution player to the publicly funded National Medical Stores. In August 2022, JMS established PSA oxygen generators in Kampala, the administrative, commercial and capital city of Uganda to address the prevalent challenge of inadequate access to medical grade oxygen in Uganda, exacerbated by the COVID19 pandemic. JMS supplies medical oxygen primarily to health facilities accredited to the Uganda Catholic and Protestant Medical Bureaux (also referred to as private not-for-profit health facilities) principally through oxygen cylinders. It is important to note that a handful of tertiary private not-for-profit facilities in Uganda such as Nsambya Hospital located in the Ugandan capital and Lacor Hospital in Northern Uganda also have on-site PSA plants. For private-for-profit facilities, the JMS is a major source of medical oxygen such as in the case of Nakasero Hospital, a top-tier private hospital, in the Ugandan capital.

> *"With regard to PNFP [private not-for-profit] facilities, Joint Medical Stores is the main supplier. JMS has a PSA plant with a production capacity of 38 nanometer cubed. They have two plants both with a capacity of 19 nanometer cubed which gives us 38 nanometer cubed. That is what JMS contributes to the national production pool. The other two PNFP plants which we have in Uganda we have is the one with a capacity of 15 nanometer cubed at Nsambya Hospital and Holy Innocents a paediatric hospital in Mbarara in South Western Uganda. There is also one based at Lacor Hospital in Northern Uganda with a capacity of 11 nanometer cubed. That is the capacity we have in the private not for profit sector."* Ministry of Health Official

Interviewees reported that Roofings Uganda Limited, one of the largest steel manufacturers in Uganda, produces high volumes of medical oxygen and offers it to public facilities at no charge on-site once empty cylinders are provided for refilling. Since the second wave of the COVID-19 pandemic in Uganda in March 2020, Roofings Uganda has been a major supplier to the National Medical Stores (NMS) which re-distributes the oxygen through cylinders to facilities in need across Uganda.

> *"We have to give credit to Roofings Uganda. During the second wave of the COVID-19 pandemic they provided us with 35,000 oxygen cylinders. They supplied these cylinders to over 168 facilities across Uganda for both public and private facilities. Also NMS was getting their supply for national distribution initially from Roofings actually that was before they opened their own PSA plant in February 2024."*
> Biomedical engineer, Ministry of Health

Our national-level informants also revealed that there were a handful of private firms based in the Ugandan capital which produced medical oxygen on demand such as Oxygen Uganda which largely produced industrial oxygen but could produce medical grade oxygen on order. Our interviewees also reported that the National Medical Stores had already completed construction of a liquid oxygen plant with the capacity to 'produce 25 oxygen cylinders per hour'. This was intended as a backup for the established PSA plants countrywide. However, this plant had not yet started oxygen production.

> *"As a country we budgeted that we shall have a 60,000 liters' capacity liquid oxygen tanker at National Medical Stores as a backup for all our PSA plants. As a country, if our PSA plants break down, you need uninterrupted oxygen supply. The liquid oxygen plant we have is what we call chimerical oxygen, it is refilled with supplies from outside of Uganda once a year and when it is full and we assume the evaporation rate will be 2% lost in a calendar year"* Ministry of Health worker

Oxygen concentrators emerged as the second most important source of medical oxygen at the point of service delivery based on the facilities we visited. Health workers indicated they elect to use concentrators due to their sheer simplicity owing to their ready-to-use attribute. Several of the concentrators we observed in the facilities we visited were developed for home-use and were not intended for multiple users, as they were being used in hospital settings. We only found a few that were meant for multiple users. Nurses reported that concentrators frequently broke down due to overuse and due to having a poor maintenance regime.

Cylinders as the backbone of distribution

We found that in Uganda, the medical oxygen distribution system is principally cylinder-based. Bedside cylinders are the principal medical oxygen storage unit and the main distribution vehicle. Refills for medical oxygen by private and public producers of medical oxygen is predominantly via bedside cylinders. Whenever there are stock-outs, public or private facilities ask for refills by providing empty cylinders to producers or suppliers of medical oxygen. Roofings Limited offers free medical oxygen supplies to public and private facilities on condition that they provide empty cylinders and are willing to meet the costs of transportation of refilled cylinders. Our findings suggest that oxygen supplies are indeed freely available at Roofings, but that facilities are frequently unable to meet the financial cost of transportation of refilled oxygen cylinders. A recent case involved Hoima RRH in Western Uganda:

> *"There instances where Regional Referral Hospitals approach us for emergency supplies of medical oxygen when they experience stock outs. However, it is common to invite them to pick the oxygen from here in Kampala but you find they don't have the money for transport. For instance, Hoima RRH made a requisition*

with us but it wasn't honored in time because they appear to lack operational funds. I am told the problem emanated from Ministry of Finance delaying to release operational funds to them. So those kinds of delays are common but not t oxygen is actually available in plenty but transportation is usually the challenge." Program Manager, National Medical Stores

In the majority of the facilities we visited across both private and public facilities, bedside cylinders were, by far, the most common source of medical oxygen on hospital wards. There was a paucity of piping systems for distributing oxygen across the various service points within the facilities we visited, and hence the importance of bedside cylinders in getting the commodity to the patients who need it. Even in facilities with PSA plants, bedside cylinders were the default source of medical oxygen on the wards. The over reliance on oxygen cylinders as the primary source of storage and distribution in Uganda has put immense pressure on the supply of empty cylinders. There were complaints raised by oxygen producers such as JMS and NMS on the quality of empty cylinders presented to them for refills ranging from old age and broken nasals.

"I think we should push for more availability of oxygen cylinders because some of these cylinders are now obsolete and it wears their integrity and sometimes with poor handling they can break, the nasals can break. If there could be a way in which they can purchase more durable and long- lasting oxygen cylinders that would be a very good thing. For example, hospitals can bring about 100 empty cylinders and then about 10 or 12 of them are faulty. So, if the government can purchase more cylinders that would be good. And also, if there could be training programmes for engineers to be to get more knowledge on how to maintain and repair cylinders." Plant Operator, National Medical Stores

During the process of transporting oxygen cylinders for refills many of them suffer damage due to "*manhandling*" and lack of practice guidelines for safe transportation of medical oxygen cylinders. Few facilities have specialized trucks for carrying the cylinder in the upright position and many are lost or suffer pilferage during transit to production sites.

"To some extent poor handling of cylinders while they are being ferried to suppliers is causing loss of cylinders and reducing their availability for refill because sometimes when the trollies aren't enough, the technicians at facilities roll the cylinders down on the floor and that's where sometimes the nodes break off. So, it's a challenge. There are vehicles that are specialized in carrying cylinders in an upright and safe position, that should be procured but funding is a common challenge. At NMS we procured specialized oxygen transport vehicles because during transportation the knobs can snap off and break. This can be dangerous because this is pressurized air." Program Manager, National Medical Stores

Operational constraints of running PSA plants

Interviews with national-level informants revealed that over the previous five years the Ugandan Government had been implementing a deliberate strategy of increasing the number of PSA plants, particularly at the tertiary level of care that predated the COVID-19 pandemic. As presented earlier, at the level of public Regional Referral Hospitals, PSA plants were a principal source of medical oxygen, with 17 PSA plants established across Uganda's major sub-regions. Select RRHs such as Mbarara RRH and Fort Portal RRH each had two PSA plants each. At three National Referral Hospitals of Mulago, Kawempe and Kiruddu based in the Ugandan capital, stand-by PSA plants had been set up by private companies as emergency backup. National-level informants indicated that medical oxygen demands were much higher at the tertiary level facilities compared to the secondary level, given the presence of more ward areas, including

intensive care units. Consumption patterns varied across the different service points within a hospital, but the average consumption was described by a participant:

> *'Medical oxygen is utilized in each of these particular units for example in the OPD you realize the average is about five meters per minute and the rationale here is that commonly in OPD the cases being handled are using a nasal prong and looking at the oxygen flow by nasal prong we are going to find it at zero such that one to five liters per minute is what is used. If the patient is not responding, then you need to escalate to a higher gadget like a simple face mask or you need to escalate to a partial venturi mask or escalate to a nanometer mask which is going to be using around 15 liters per minute. If we take average consumption per patient, then its five liters per minute'* Health worker at study health facility

Frequent breakdowns of newly established PSA plants were consistently reported in the national print media over the previous two years. At Kabale RRH in South Western Uganda, the lone PSA plant had been broken down for close to six months at the time of the study. As a result, Kabale RRH had to procure medical oxygen more than 400 kilometers away, in the capital of Kampala, using cylinders. Interviews with informants highlighted that the paucity of spare parts due to the diverse brands in service was a major constraint and that it was common for breakdowns to last weeks due to the lack of spare parts which often had to be imported. Health facilities were contractually bound to order spares for equipment and associated accessories from the original foreign suppliers. External suppliers engaged 'local agents' to service this equipment. This arrangement denied operational experience of servicing and repairing medical oxygen equipment by a technical cadre on the public sector payroll.

A key finding of this case study was that PSA plants did not have recurrent budgets for routine maintenance, servicing and repair. Recurrent costs such as electricity supply for PSA plants were not provided for in operations budgets for facilities.

> *"There are several operational challenges associated with running PSA plants at facility level. While setting up PSA plants, the Ministry of Health did not include maintenance budgets for these plants. When the plants break down, hospitals incur the cost of bringing in a maintenance engineer but there is often no budget line for this in public facilities. Some regional referral hospitals such as Kabale RRH have two plants which increases maintenance costs. Then some hospital PSA plants have failed due to of lack servicing agreements with equipment suppliers. Once they commission the plant the hospital is left to its own devices. There is no after sales support."* Ministry of Health official.

A plant operator at a newly established PSA plant run by NMS with a capacity of producing 30 oxygen cylinders per day elicited that the maintenance requirements for PSA plants were out of reach of most public facilities with constrained recurrent budgets. This was unlike the NMS which is autonomous with a much larger operational budget compared to a typical public facility in Uganda.

> *"The maintenance schedules are demanding. We have a weekly servicing routine, monthly and every after six months and after a year according to the manuals. So weekly we make sure that we change the water in the cooling tank, the one that cools the booster compressor that fills the cylinders with oxygen and then we also do dusting because even when the place is well ventilated sometimes dust comes in so we have to dust the filters. There is a sponger filter where air cooling the compressor that we need to dust with a blower. General cleaning of equipment is weekly. We do general inspections on a regular basis. We have a maintenance guide that we follow."* PSA Plant Operator, National Medical Stores

At the Joint Medical Stores, it was reported that the monthly electricity bill for running the PSA plant there is more $10,000 which was hardly affordable for public facilities with multiple competing recurrent needs.

Even when hospitals had operational PSA plants, oxygen delivery systems had been found to be a 'weak link'. Having elaborate piping systems that distributed the medical oxygen to the various service points within a hospital complex was a teething challenge in many public facilities. Leakages in the piping systems were common. A related challenge was that of secondary accessories that moved medical oxygen from production to consumption, including basics such as nasal prongs. Accompanying equipment accessories such as flow meters were often in short supply. The shortage of pulse oximeters had been identified as having a ripple effect on oxygen supply management.

The print media articles in the Uganda national press we reviewed also identified a challenge with the number of cylinders that hospital-based PSA systems can have access to. Most of the cylinders seemed to be tied to centralised oxygen systems such as NMS. That is to say, the cylinders were exchangeable with NMS (and maybe JMS) and few were available outside this network. These also leaked out to industrial use such as in the cottage wielding industry. Attempts to colour code them and restrict the leakage of cylinders to the industrial users was yet to yield results.

Documentation and procedures for running an oxygen system are insufficient

Interviews with health workers across the facilities we visited suggested that documentation of medical oxygen use was haphazard. We found that medical oxygen use was not provided for in the current health information systems architecture of Uganda. Documenting medical oxygen consumption trends was not provided for in routinely collected data using the Ministry of Health - health management information system (HMIS) at health facility level. Our findings revealed that consumption patterns were rarely documented at the frontline level of service delivery which negatively impacted the data available to inform the planning and implementation of the entire medical oxygen supply chain. There were no paper-based or digital platforms that allowed for capturing data on medical oxygen demand and supply at routine points of care.

Within health facilities in Uganda, there appeared to be no formal standardized medical oxygen information management systems for unit operations like procurement, ordering, storage, intra-facility distribution and use in patients. We found multiple regimes for the management of medical oxygen supply chains at different facilities. The cadres of health workers involved in the medical oxygen supply chain management were diverse. Overall, we found that nurses were the most frequently cited cadre regarding medical oxygen decision making at the bottom stream end of the supply chain. Often, nurses managed the requisition for the commodity at the point of service delivery. However, most requisitions for oxygen were made largely out of medical emergencies on the various wards such as NICUs, ICUs or labour suites rather through systematic procurement planning.

> *"The standard requisitioning is usually done by the nurse who puts in a requisition to the store keeper or such other staff. But most of these orders come in as emergency orders. They are not routine orders. But our PSA plant has been down for some time now. So, we have actually been placing orders to National Medical Stores."* Facility in-charge

Even at the level of tertiary hospitals we found vast variations in the availability of specialized personnel for managing medical oxygen supply chains. At Mbarara RRH in South Western Uganda, we found that a qualified biomedical engineer was on the staff. The biomedical engineer oversaw requisitions for medical oxygen and was instrumental in running the two PSA

plants at Mbarara RRH. However, this was the exception rather than the rule in the rest of the tertiary hospitals we visited. At Fort Portal RRH in mid-western Uganda, there was no qualified biomedical engineer or trained plant operator. Staff who managed PSA plants were in their roles on an ad-hoc basis and didn't have specialized training for the roles in which they were deployed. At the secondary level of service delivery, such as at general hospitals, the level of specialization of personnel for medical oxygen was even lower.

Our qualitative interviews revealed the diverse cadres of specialized staff required to run and operate PSA plants one of the many functions of managing medical oxygen supply chains.

> *"With regard to the required staff responsible for running an oxygen plant, you need plant operators, you need a plant maintenance team, you need an estates and you need a mechanical engineer. So the staff you need includes a civil engineer, an electrical engineer, a plumbing technician. You need a monitoring team to ensure that the quality of oxygen produced meets the standards"* Plant Operator, National Medical Stores

In terms of processes or procedures for requisitioning for medical oxygen, we found that there were wide variations in practices in our sample of facilities. Overall, it is a nurse who places orders for the commodity with a 'storeman' although the term to describe such personnel may vary across facilities. We found that even in facilities which have on-site PSA plants, the process of requisition still involved a nurse or clinician placing an order with some personnel in the facility managing medicines and commodities who either did not a proper job description or did not have the requisite knowledge, skills and capacity building opportunities or both.

Key messages

Prioritizing medical oxygen on policy agendas

- Medical oxygen needs to be prioritized in the public health sector agenda by increasing financial commitments in annual recurrent and capital budgeting at the national, sub-national and facility levels.
- Robust budgeting requires health information systems that capture trends in consumption. This will necessitate integrating medical oxygen data capture into routinely collected data in district health information systems (DHIS-2).
- There is need to need for civil society advocacy in order to sustain medical oxygen issues on the public sector agenda amidst the array of competing needs across health conditions in Uganda.

Health workforce gaps

- Health workforce gaps in medical oxygen security emerged as a cross-cutting constraint in the case of Uganda. Particularly, dependence on expertise from foreign-based equipment suppliers in servicing and repairing PSA plants emerged as a major issue. High-impact interventions such as peer to peer mentorships, regionally based support teams and workshops could be worthwhile considerations.
- From a biomedical engineering perspective, building a critical mass of qualified biomedical engineers that are competent in medical oxygen equipment management, including providing tertiary training institutions-level attachments, should be a priority.
- Delegating responsibility for the maintenance and servicing of PSA plants across the country to the National Medical Stores would enable build technical expertise and attract dedicated budgets given its unique status in Uganda's health commodity supply chain.

- Policy and programming interventions for medical oxygen security in Uganda were heavily donor-funded and donor-driven which calls for urgent consideration of more predictable and government led health financing based on efficient and optimal use of public resources.

Centralization of oxygen governance systems

- The centralization of oxygen production and distribution appears to stem from differential funding arrangements for central production relative to hospital-based PSA plant systems. NMS and JMS are paid for the oxygen production (JMS can sell its product) from government or the private sector.
- Public facilities with PSAs have found it cheaper to collect "free" oxygen cylinders from NMS, than producing oxygen on-site due to high operational costs. This model also favours NMS that stands to gain from supplying (and therefore billing) higher volumes.
- The issue of parallel investments in centralization production, and on-site hospital-PSAs with competing incentives need harmonization at an ‘upstream’ level.
- There is need to engender a broader health systems strengthening approach to medical oxygen supply chains in terms of governance arrangements and financing pathways.

Additional methods information

This case study is based on empirical data collected from 10 key informant interviews – 4 female and 6 male - of purposively selected participants, review of extracts of publications in the national media dailies and review of reports of four consultative workshops held by the national medical oxygen coordinating task force between January and June 2024. The participants in the interviews were selected for maximum variation to enable the study to explore and dig deeper into different views of the realities of the medical oxygen supply chain in Uganda as shown below.

Representatives from health sector partners engaged in supporting the Ministry of Health on the National Implementation Plan for Medical Oxygen Scale-up
Non-state practitioners in the medical oxygen supply chain ecosystem
Activist from a Civil Society Organization engaged on health advocacy
Actors in the public sector on the medical oxygen supply chain ecosystem
Ministry of Health officials
Practicing health worker at one of the study health facilities

Table 2: Key informant interview participants

References

1. Uganda Bureau of Statistics. The National Population and Housing Census 2024 – Preliminary Report. Kampala, Uganda. 2024.
2. UN IGME. United National Inter-Agency Group for Child Mortality Estimation (UN IGME). United Nations Inter-agency Group for Child Mortality Estimation (UN IGME); 2019.
3. Uganda Bureau of Statistics, ICF International. Uganda Demographic and Health Survey 2023. Kampala, Uganda: Uganda Bureau of Statistics (UBOS) and ICF International, 2023.
4. Uganda Bureau of Statistics. The 2022 Statistical Abstract. Kampala, Uganda: 2022.
5. Duke T, Graham SM, Cherian MN, Ginsburg AS, English M, Howie S, et al. Oxygen is an essential medicine: a call for international action. Int J Tuberc Lung Dis. 2010;14(11):1362-8. PubMed PMID: 20937173; PubMed Central PMCID: PMCPMC2975100UKMS33165.
6. WHO. World Health Organization Model List of Essential Medicines: 21st List. Geneva, Switzerland: World Health Organization (WHO); 2019.
7. Margini, F., Anooj Pattnaik, Tapley Jordanwood, Angellah Nakyanzi, and Sarah Byakika, Case Study: The Initial COVID-19 Response in Uganda. 2020, Washington, DC: ThinkWell and Ministry of Health Uganda.1. Uganda Bureau of Statistics. The 2022 Statistical Abstract. Kampala, Uganda, 2022.
8. Mcintyre D, Meheus F, Røttingen JA. What level of domestic government health expenditure should we aspire to for universal health coverage? Health Econ Policy Law. 2017 Apr;12(2):125-137. doi: 10.1017/S1744133116000414. PMID: 28332456.
9. Ministry of Health Uganda. National Health Accounts 2016-2019. Kampala, Uganda. 2019
10. MOH-Uganda. National Scale up of Medical Oxygen Implementation Plan. Kampala, Uganda: The Republic of Uganda Ministry of Health, 2018.
11. Uganda Ministry of Health, National Implementation Plan for Medical Oxygen Scale up 2023/24 – 2027/28. Kampala, Uganda. 2023.
